# Nucleophilic trifluoromethoxylation of alkyl halides without silver

**DOI:** 10.1038/s41467-020-14598-1

**Published:** 2020-02-06

**Authors:** Yan Li, Yang Yang, Jinrui Xin, Pingping Tang

**Affiliations:** State Key Laboratory and Institute of Elemento-Organic Chemistry, College of Chemistry, Nankai University, Tianjin, 300071 China

**Keywords:** Synthetic chemistry methodology, Medicinal chemistry, Reaction mechanisms

## Abstract

The biological properties of molecules containing the trifluoromethoxy group have made these compounds important targets in pharmaceuticals and agrochemicals, yet their preparation is still a substantial challenge. Herein, we present a practical nucleophilic trifluoromethoxylation of alkyl halides with (*E*)-*O*-trifluoromethyl-benzaldoximes (TFBO) as a trifluoromethoxylation reagent in the absence of silver under mild reaction conditions. The trifluoromethoxylation reagent TFBO is easily prepared and thermally stable, and can release CF_3_O^−^ species in the presence of a base. Furthermore, broad scope and good functional group compatibility are demonstrated by application of the method to the late-stage trifluoromethoxylation of alkyl halides in complex small molecules.

## Abstract (web summary)

Trifluoromethyl ethers are important bioactive targets in pharmaceuticals and agrochemicals, however, their synthesis is often not straightforward. Here, the authors disclose a reagent for the nucleophilic trifluoromethoxylation of alkyl halides without silver and under mild conditions.

## Introduction

A growing number of fluorine-containing organic compounds have widespread application in the fields of pharmaceuticals, pesticides and materials because of irreplaceable properties of fluoride^1–5^. The incorporation of fluorine-containing groups has been an efficient strategy for the design of new drugs and agrochemical. In recent years, the late-stage and selective fluorination reaction of organic molecules has received significant attention, especially the trifluoromethoxylation reaction, which is one of the most important research hotspots, as the trifluoromethoxy group’s electron-withdrawing effects and high lipophilicity (Hansch parameter π_x_ = 1.04)^6–10^. However, the trifluoromethoxylation reaction remain limitations and challenges, such as limited trifluoromethoxylation reagents and instability of trifluoromethoxide anion, which impede its development and application^11–14^.

**Fig. 1:**
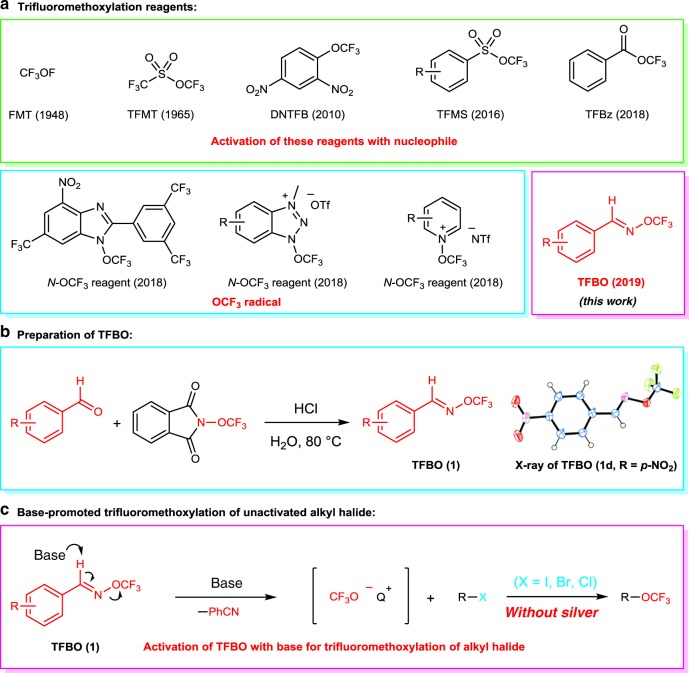
Reaction design. **a** Trifluoromethoxylation reagents. **b** Preparation of (*E*)-*O*-trifluoromethyl-benzaldoximes (TFBO). **c** The current method for nucleophilic trifluoromethoxylation of alkyl halides without silver.

The synthesis of trifluoromethyl ethers can be achieved by indirect strategies and direct strategies. The indirect strategies include the nucleophilic fluorination of ether groups^15–17^ and electrophilic trifluoromethylation of hydroxyl groups^18–23^, which suffered from poor substrate scope, harsh reaction conditions and use of highly toxic reagents. The direct strategies are the direct introduction of the trifluoromethoxy group into organic compounds with trifluoromethoxylation reagents^24^. For example, *tris*(dimethylamino)sulfonium trifluoromethoxide (TASOCF_3_) was used as a trifluoromethoxylation reagent by Ritter’s group to achieve the direct trifluoromethoxylation of aryl stannanes and aryl boronic acids with equivalent silver^25^. Liu group reported a palladium-catalyzed intramolecular aminotrifluoromethoxylation of alkenes with AgOCF_3_ or CsOCF_3_ as the trifluoromethoxylation reagent^26,27^. The catalytic C(sp^2^)-H trifluoromethoxylation of arenes with the new *N*-OCF_3_ reagents under photocatalytic conditions had been reported by Ngai and Togni, respectively^28–30^. One of the simplest methods for the formation of the C(sp^3^)-OCF_3_ group is the nucleophilic substitution because of widely available leaving groups and inexpensive starting materials^31–37^. However, due to the poor nucleophilicity and instability of trifluoromethoxide anion, most of the known nucleophilic trifluoromethoxylation methods require to use activated electrophiles such as allylic halides, benzylic halides or α-halo carbonyl compounds with few exceptions, and available trifluoromethoxide anion (^–^OCF_3_) sources are scarce and usually suffere from disadvantages (Fig. 1a)^38^. For example, direct nucleophilic trifluoromethoxylation of alkyl iodides or bromides with trifluoromethyl triflate (TFMT)^39,40^, 2,4-dinitro(trifluoromethoxy)benzene (DNTFB)^33^ or trifluoromethyl benzoate (TFBz)^37^ were reported. However, less than 10% yield of desired products were obtained with unactivated alkyl iodide such as citronellyl iodide in the absence of silver, and trifluoromethoxide anion (CF_3_O^−^) was generated from these reagents under the activation of a fluoride salt, which might form the fluorinated byproduct. Furthermore, only one example of trifluoromethoxylation of benzyl chloride with AgOCF_3_ was reported to generate the desired product in 29% yield^32^, and no method is reported to achieve the trifluoromethoxylation of alkyl chloride in the absence of silver. Therefore, the development of an efficient method for nucleophilic trifluoromethoxylation of unactivated alkyl halides with a trifluoromethoxylation reagent in the absence of silver is highly desirable.

We envisioned developing a trifluoromethoxylation reagent which is active enough to readily release CF_3_O^−^ without the activation of nucleophiles such as fluoride salts. Inspired by alkoxy anion generated from the base-promoted elimination reactions of (*E*)-*O*-alkyl-benzaldoximes^41^, we were wondering whether (*E*)-*O*-trifluoromethyl-benzaldoximes (TFBO) can be prepared and used as a trifluoromethoxylation reagent if a suitable base is found to activate the reagent to generate trifluoromethoxide anion in situ, which would react with unactivated alkyl halides. (Fig. 1b) Herein, we report the development of (*E*)-*O*-trifluoromethyl-benzaldoximes (TFBO) as a trifluoromethoxylation reagent, which can be easily prepared from benzyl aldehydes and *N*-trifluoromethoxy phthalimide^42^ in modest yields. TFBO is shelf-stable and can be easily activated by the base to release CF_3_O^−^ species (Fig. 1b). With (*E*)-*O*-trifluoromethyl-benzaldoximes (TFBO) as a trifluoromethoxylation reagent, an efficient nucleophilic trifluoromethoxylation of unactivated alkyl halides in the absence of silver is reported. This reaction is operationally simple, scalable, and which shows potential value in the field of drug synthesis (Fig. 1c).

## Results

### Investigations of reaction conditions and scope

**Fig. 2:**
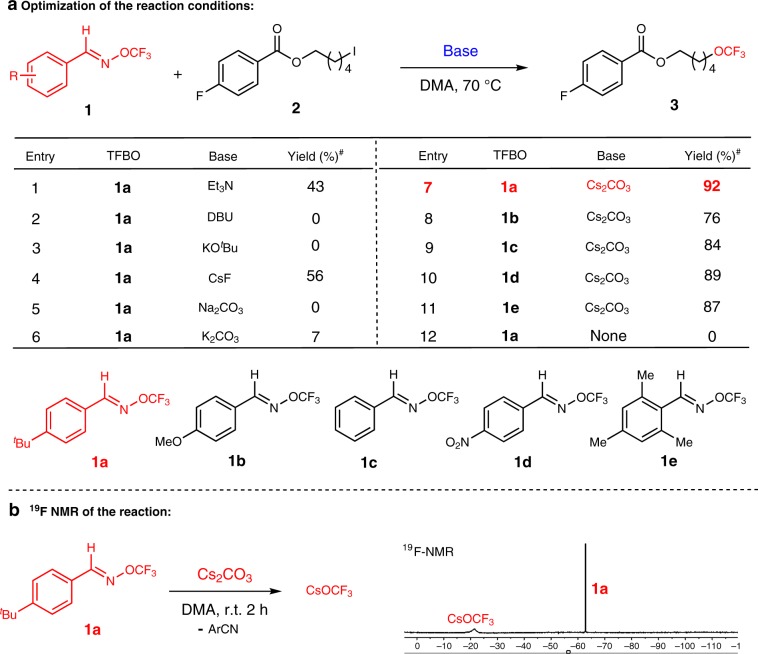
Reaction development. **a** Optimization of the reaction conditions. Standard reaction conditions: alkyl halide (1.0 equiv.), base (3.5 equiv.), TFBO (5.0 equiv.), DMA, 70 °C, N_2_. ^#^Yields were determined by ^19^F NMR with benzotrifluoride as a standard. **b** Monitoring of the reaction between TFBO (**1a**) and Cs_2_CO_3_ by ^19^F NMR spectroscopy.

The initial efforts were focused on the reaction of 5-iodopentyl 4-fluorobenzoate **2** with various (*E*)-*O*-trifluoromethyl-benzaldoximes (TFBO) in the presence of a base. As briefly illustrated in Fig. 2a, the use of a base was crucial for achieving an efficient transformation in the presence of TFBO, and Cs_2_CO_3_ was found to give the highest yield. Changing the base to other organic bases Et_3_N, DBU or inorganic bases KO^*t*^Bu, CsF, Na_2_CO_3_, K_2_CO_3_ resulted in lower yields. Next, a thorough evaluation of different TFBOs revealed that substituents on the aromatic rings influenced the reaction yields, and the (*E*)-*O*-trifluoromethyl-4-*tert*-butyl-benzaldoximes **1a** was found to be particularly effective. The control experiments were performed and no desired product was observed in the absence of a base. Monitoring of the reaction between TFBO (**1a**) and Cs_2_CO_3_ by ^19^F NMR spectroscopy indicated that CsOCF_3_ (–20.9 ppm) and aryl nitrile were generated in the reaction (Fig. 2b)^31^. After thoroughly optimizing the reaction conditions, reactions with 3.5 equiv. of Cs_2_CO_3_, 5.0 equiv. of TFBO (**1a**) in DMA under N_2_ atmosphere were found to produce high yields of the desired product.

**Fig. 3:**
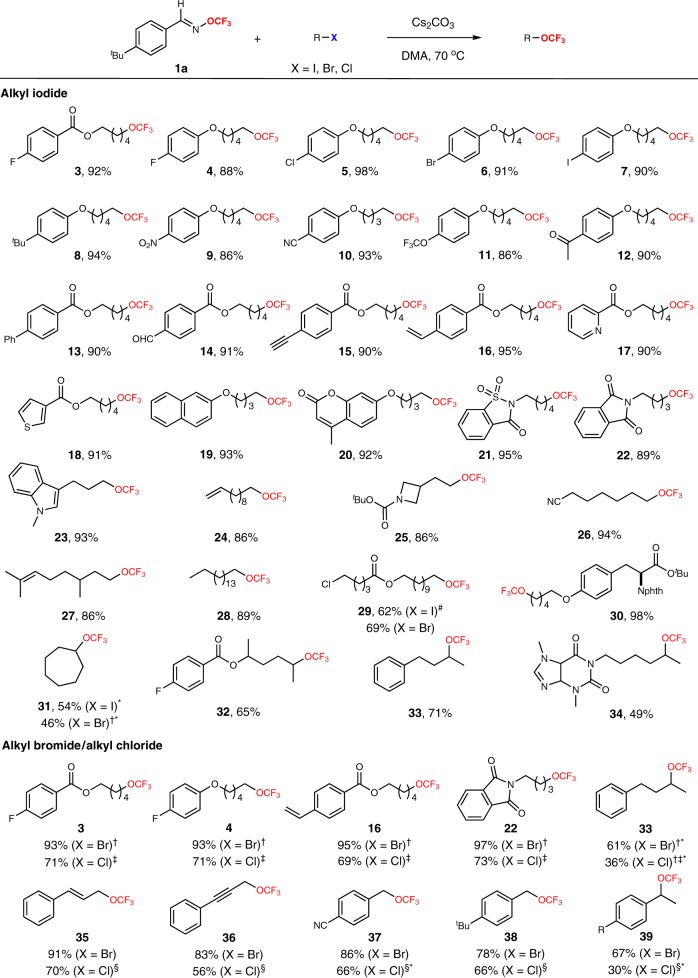
Substrates scope for trifluoromethoxylation of simple alkyl halides. Standard reaction conditions: alkyl halide (1.0 equiv.), Cs_2_CO_3_ (3.5 equiv.), TFBO (5.0 equiv.), DMA, 70 °C, N_2_. ^#^50 °C. ^†^90 °C. ^‡^Cs_2_CO_3_ (4.0 equiv.), TFBO (6.0 equiv.), TBAI (3.0 equiv.), HMPA. ^§^Cs_2_CO_3_ (4.0 equiv.), TFBO (6.0 equiv.), TBAI (0.2 equiv.), HMPA.^*^Yields were determined by ^19^F NMR with benzotrifluoride as a standard.

With the optimized conditions in hand, we explored the substrate scope of the transformation (Fig. 3). First, a wide range of unactivated alkyl iodides was successfully converted to the corresponding trifluoromethyl ethers with yields ranging from 49 to 98% (**3** to **34**). Substrates bearing electron-donating and electron-withdrawing substituents on aryl rings proceeded well. This transformation was also compatible with excellent functionalities, such as ester, ether, ketone, aldehyde, imide, amide, cyano, nitro, aryl chloride, bromide, and iodide groups. Notably, heteroaromatic substrates and amino acid derivative were also successfully employed to provide the corresponding products^17,18,30^. Generally, the reactions of a variety of primary alkyl iodides gave rise to the desired trifluoromethoxylated products in high yields (**3** to **30**), while secondary alkyl iodides gave slightly lower yields (**31** to **34**). Next, we turned our attention to expanding the substrate scope to alkyl bromides and alkyl chlorides. To our great delight, the trifluoromethoxylation of alkyl bromides and alkyl chlorides proceeded smoothly with yields ranging from 30 to 97%. Furthermore, the allyl, propargyl, and benzyl halides were successfully converted into the desired trifluoromethoxylated products (**35** to **39**). It is worth mentioning that chemoselectivity trifluoromethoxylation of alkyl iodides or alkyl bromides in the presence of alkyl chlorides were observed. For example, the alkyl iodide or alkyl bromide was selectively converted into a trifluoromethoxy group while the alkyl chloride remained intact^29^. The yields of fluorination byproducts were less than 10% in all cases. In addition, the product **33** with 8% ee was observed when the chiral substrate (85% ee) was used, which suggested that the S_N_1-type nucleophilic trifluoromethoxylation might be involved in the reaction. Furthermore, other elecrophiles such as alkyl OMs, alkyl OTs were also successfully converted into the desired trifluoromethoxylated products in good yields (see the Supplementary Information for details). The limitation of this method was that no desired trifluoromethoxylated products were observed with tertiary halides. In addition, to demonstrate both scalability and practicality of this method, we prepared **28** on a gram scale under the standard reaction conditions in 89% isolated yield.

**Fig. 4:**
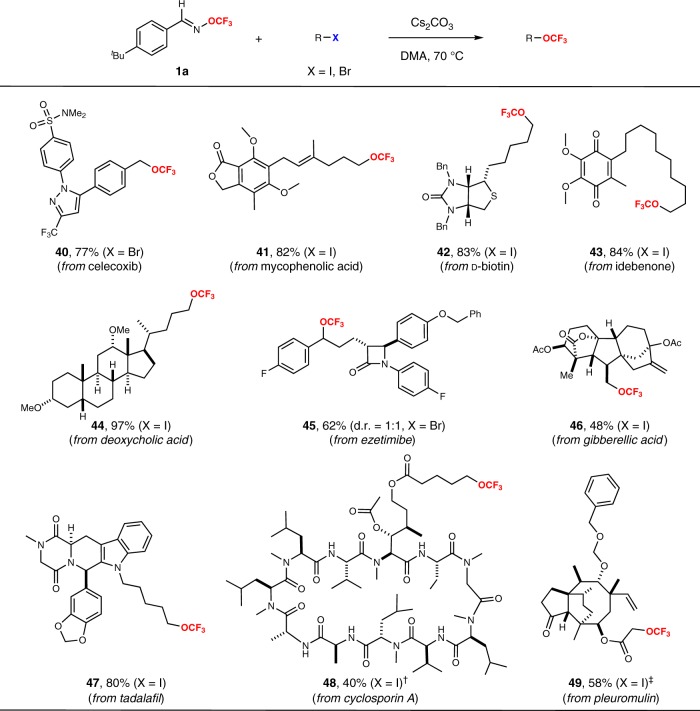
Substrates scope for trifluoromethoxylation of complex alkyl halides. Standard reaction conditions: alkyl halide (1.0 equiv.), Cs_2_CO_3_ (3.5 equiv.), TFBO (5.0 equiv.), DMA, 70 °C, N_2_. ^†^Cs_2_CO_3_ (4.0 equiv.), TFBO (6.0 equiv.), Dibenzo-18-crown-6 (2.0 equiv.). ^‡^50 °C.

Due to the ubiquity of the trifluoromethoxy group in small-molecule drugs and preclinical candidates, it would be more significant to achieve the late-stage trifluoromethoxylation of complex small molecules with our trifluoromethoxylation reagents. To confirm this strategy, we selected ten meaningful small molecules as the substrates of trifluoromethoxylation.(Fig. 4). Each of these architecturally complex molecules underwent trifluoromethoxylation of alkyl halides to achieve the corresponding trifluoromethoxylated products in moderate to excellent yields (**40** to **49**, 40–97% yield). For example, pentacyclic diterpene gibberellic acid is a plant hormone that promotes growth and influences developmental processes, including cell germination and elongation. Cyclosporin A is an immunosuppressant medication and natural product, which is a macrocyclic peptide of 11 amino acids. To our delight, the trifluoromethoxylation reaction with the gibberellic acid derivative and cyclosporin A derivative proceeded smoothly to provide the corresponding products (**46**, **48**) in good yields, which illustrates the ability to conduct the late-stage trifluoromethoxylation of complex structures.

## Discussion

In conclusion, we have developed (*E*)-*O*-trifluoromethyl-benzaldoximes (TFBO) as a trifluoromethoxylation reagent for nucleophilic trifluoromethoxylation of alkyl halides without silver. The method offers direct access to trifluoromethoxylated compounds from complex small molecules in the late stage. Compared to other trifluoromethoxylation reagents (see more detail in the Supplementary Table 12), TFBO can be easily activated by a base to release CF_3_O^−^ species. Additionally, the reaction tolerates a various range of functional groups and is amenable to gram-scale synthesis. We expect that the operational simplicity, efficacy and broad scope of this method will find widespread use in pharmaceutical and agrochemical research.

## Methods

### General procedure for the synthesis of trifluoromethoxylation reagents

In a round-bottom flask, PhthNOCF_3_ (**S1**) (2-(trifluoromethoxy)isoindoline-1,3-dione) (1.00 g, 4.33 mmol, 1.00 equiv.), water (4.00 mL), HCl (0.720 mL ca. 6.00 M aq., 4.33 mmol, 1.00 equiv.) and aldehyde (1.50 equiv.) were added. The mixture was stirred at 80 °C overnight. Afterwards, the reaction mixture was extracted with CH_2_Cl_2_ (20.0 mL × 2). The combined organic layer was dried over anhydrous MgSO_4_, filtered and concentrated. The residue was purified by silica gel chromatography, to afford trifluoromethoxylation reagents.

### General procedure for the synthesis of trifluoromethoxylated products

In a N_2_ glovebox, to alkyl halides (1.00 equiv.), (*E*)-*O*-trifluoromethyl-4-*tert*-butyl-benzaldoximes (**1a**) (307 mg, 1.25 mmol, 5.00 equiv.) in a 15.0 mL sealed vial were added DMA (2.00 mL). Cs_2_CO_3_ (285 mg, 0.875 mmol, 3.50 equiv.) was added to the reaction and the resulting mixture was stirred for overnight at 70 °C. After cooling to 50 °C, NMO (4-methylmorpholine *N*-oxide) (58.6 mg, 0.500 mmol, 2.00 equiv.) was added and the reaction mixture was stirred 2 h. Then the resulting mixture was filtered and concentrated *in vacuo*. The residue was purified by preparative TLC, to afford trifluoromethoxylated products.

## Supplementary information

Supplementary Information

## Data Availability

The authors declare that the data supporting this study are available within the article and supplementary information files. The X-ray crystallographic coordinates for compound (**1d**) reported in this study have been deposited at the Cambridge Crystallographic Data Centre (CCDC), under deposition numbers 1907514. These data can be obtained free of charge from The Cambridge Crystallographic Data Centre via www.ccdc.cam.ac.uk/data_request/cif.
