# Supplementary material for: Nucleophilic trifluoromethoxylation of alkyl halides without silver
Source: Nat Commun. 2020 Feb 6;11:755. doi: 10.1038/s41467-020-14598-1 (PMC7005179; doi:10.1038/s41467-020-14598-1)
Supplement: Supplementary file 1 — Supplementary Information [file 41467_2020_14598_MOESM1_ESM.pdf]

Supplementary Information

Nucleophilic Trifluoromethoxylation of Alkyl Halides with a Trifluoromethoxylation Reagent without Silver

Li *et al.*

Table of Contents

Supplementary Information 1

Supplementary Methods: 5

Effect of solvents on the reaction 6

Effect of "OCF_3" sources on the reaction 6

Effect of temperature on the reaction 8

Effect of concentration on the reaction 8

Effect of bases on the reaction 9

Effect of "OCF_3" amount on the reaction 10

Effect of reaction time on the reaction 11

Effect of H_2O amount on the reaction 12

Effect of the volume of sealed vial on the reaction 13

Effect of atmosphere on the reaction 13

2-(Trifluoromethoxy)isoindoline-1,3-dione (**S1**) 16

(*E*)-*O*-Trifluoromethyl-4-*tert*-butyl-benzaldoximes (**1a**) 16

(*E*)-*O*-Trifluoromethyl-4-methoxybenzaldoximes (**1b**) 17

(*E*)-*O*-Trifluoromethyl-benzaldoximes (**1c**) 17

(*E*)-*O*-Trifluoromethyl-4-nitrobenzaldoximes (**1d**) 18

(*E*)-*O*-Trifluoromethyl-2,4,6-trimethylbenzaldoximes (**1e**) 18

(*E*)-*O*-Trifluoromethyl-4-bromobenzaldoximes (**1f**) 19

(*E*)-*O*-Trifluoromethyl-4-iodobenzaldoximes (**1g**) 19

(*E*)-*O*-Trifluoromethyl-4-trifluoromethylbenzaldoximes (**1h**) 20

(*E*)-*O*-Trifluoromethyl-4-cyanobenzaldoximes (**1i**) 20

(*E*)-*O*-Trifluoromethyl-4-methoxycarbonylbenzaldoximes (**1j**) 21

(*E*)-*O*-Trifluoromethyl-2-nitrobenzaldoximes (**1k**) 21

(*E*)-*O*-Trifluoromethyl-4-fluorobenzaldoximes (**1l**) 22

(*E*)-*O*-Trifluoromethyl-4-phenylbenzaldoximes (**1m**) 22

(1*E*,2*E*)-*O*-Trifluoromethyl-cinnamaldoxime (**1n**) 23

(*E*)-*O*-Trifluoromethyl-4-chlorobenzaldoximes (**1o**) 23

(*E*)-*O*-Trifluoromethyl-2-naphthaldoxime (**1p**) 24

(*E*)-*O*-Trifluoromethyl-1-naphthaldoxime (**1q**) 24

5-Chloropentyl 4-fluorobenzoate (**S3-1**) 25

5-Bromopentyl 4-fluorobenzoate (**S3-2**) 25

5-Iodopentyl (1,1'-biphenyl)-4-carboxylate (**S13**) 26

5-Iodopentyl 4-formylbenzoate(**S14**) 26

5-Iodopentyl 4-ethynylbenzoate (**S15**) 27

5-Chloropentyl 4-vinylbenzoate (**S16-1**) 28

5-Bromopentyl 4-vinylbenzoate(**S16-2**) 29

5-Iodopentyl 4-vinylbenzoate (**S16**) 29

5-Iodopentyl picolinate (**S17**) 30

1-Methyl-3-(3-iodopropyl)-1H-indole (**S23**) 30

N-Boc-3-(2-iodoethyl)azetidine (**S25**) 31

11-Bromoundecyl 5-chloropentanoate (**S29-1**) 32

11-Iodoundecyl 5-chloropentanoate (**S29**) 32

1-(5-Iodohexyl)-3,7-dihydro-3,7-dimethyl-1H-purine-2,6-dione (**S34**) 33

N,*N*-Dimethyl-4-(5-(4-(bromomethyl)phenyl)-3-(trifluoromethyl)-1*H*-pyrazol-1-yl) benzenesulfonamide (**S40**) 33

Mycophenolic acid iodination derivative (**S41**) 34

(3aS,4S,6aR)-1,3-Dibenzyl-4-(5-iodopentyl)tetrahydro-1H-thieno[3,4-d]imidazol-2 (3H)-one (**S42**) 36

Ezetimibe derivative (**S45**) 36

Gibberellic acid derivative (**S46**) 38

7-(5-Iodopentyl)tadalafil (**S47**) 39

5-Iodovaleric acid acetylcyclosporin A ester (**S48**) 40

11-Benzyloxymethylenoxy 22-iodopleuromutilin (**S49**) 42

5-Trifluoromethoxypentyl 4-fluorobenzoate (**3**) 43

1-((5-Trifluoromethoxypentyl)oxy)-4-fluorobenzene (**4**) 45

1-((5-Trifluormethoxypentyl)oxy)-4-chlorobenzene (**5**) 46

1-((5-Trifluoromethoxypentyl)oxy)-4-bromobenzene (**6**) 47

1-((5-Trifluoromethoxypentyl)oxy)-4-iodobenzene (**7**) 47

1-(*tert*-Butyl)-4-((5-trifluoromethoxypentyl)oxy)benzene (**8**) 48

1-((5-Trifluoromethoxypentyl)oxy)-4-nitrobenzene (**9**) 48

4-(4-Trifluoromethoxybutoxy)benzonitrile (**10**) 49

1-(4-Trifluoromethoxybutoxy)-4-(trifluoromethoxy)benzene (**11**) 49

1-(4-((5-Trifluoromethoxypentyl)oxy)phenyl)ethanone (**12**) 50

5-Trifluoromethoxypentyl (1,1'-biphenyl)-4-carboxylate (**13**) 51

5-Trifluoromethoxypentyl 4-formylbenzoate (**14**) 51

5-Trifluoromethoxypentyl 4-ethynylbenzoate (**15**) 52

5-Trifluoromethoxypentyl 4-vinylbenzoate (**16**) 52

5-Trifluoromethoxypentyl nicotinate (**17**) 54

5-Trifluoromethoxypentyl thiophene-3-carboxylate (**18**) 54

2-(4-Trifluoromethoxybutoxy)naphthalene (**19**) 55

7-(4-Trifluoromethoxybutoxy)-4-methyl-2H-chromen-2-one (**20**) 55
N-(5-Trifluoromethoxypentyl)saccharine (**21**) 56
2-(4-Trifluoromethoxybutyl)isoindoline-1,3-dione (**22**) 56
1-Methyl-3-(3-(trifluoromethoxy)propyl)-1H-indole (**23**) 58
11-(Trifluoromethoxy)undec-1-ene (**24**) 58
N-Boc-3-(2-trifluoromethoxyethyl)azetidine (**25**) 59
7-(Trifluoromethoxy)heptanenitrile (**26**) 60
2,6-Dimethyl-8-(trifluoromethoxy)oct-2-ene (**27**) 60
1-(Trifluoromethoxy)hexadecane (**28**) 61
11-Trifluoromethoxyundecyl 5-chloropentanoate (**29**) 61
(S)-*tert*-Butyl 2-(1,3-dioxoisoindolin-2-yl)-3-(4-((5-trifluoromethoxypentyl) oxy)phenyl)-propanoate (**30**) 62
(Trifluoromethoxy)cycloheptane (**31**) 63
5-Trifluoromethoxyhexan-2-yl 4-fluorobenzoate (**32**) 64
3-Trifluoromethoxybutylbenzene (**33**) 64
1-(5-Trifluoromethoxyhexyl)-3,7-dihydro-3,7-dimethyl-1H-purine-2,6-dione (34)66
(*E*)-(3-(Trifluoromethoxy)prop-1-en-1-yl)benzene (**35**) 66
(3-(Trifluoromethoxy)prop-1-yn-1-yl)benzene (**36**) 67
4-((Trifluoromethoxy)methyl)benzonitrile (**37**) 68
1-(*tert*-Butyl)-4-((trifluoromethoxy)methyl)benzene (**38**) 69
1-Bromo-4-(1-(trifluoromethoxy)ethyl)benzene (**39**) 70
N,*N*-Dimethyl-4-(5-(4-((trifluoromethoxy)methyl)phenyl)-3-(trifluoromethyl)-1*H*-pyrazol-1-yl)benzenesulfonamide (**40**) 71
Mycophenolic acid trifluoromethoxy derivative (**41**) 71
(3aS,4S,6aR)-1,3-Dibenzyl-4-(5-trifluoromethoxypentyl)tetrahydro-1H-thieno[3,4-d]imidazol-2(3H)-one (**42**) 72
2,3-Dimethoxy-5-methyl-6-(10-(trifluoromethoxy)decyl)cyclohexa-2,5-diene-1,4-dione (**43**) 73
3,12-Dimethoxy cholan-24-trifluoromethoxy (**44**) 73
Ezetimibe derivative (**45**) 74
Gibberellic acid derivative (**46**) 75
7-(5-Trifluoromethoxypentyl)Tadalafil (**47**) 75
5-Trifluoromethoxyvaleric acid acetylcyclosporin A ester (**48**) 76
11-Benzyloxymethylenoxy 22-trifluoromethoxypleuromutilin (**49**) 77
4-(Trifluoromethoxy)butyl)benzene (**50**) 78
Gram-scale synthesis of 1-(Trifluoromethoxy)hexadecane (**28**) 78
Comparision of TFBO with TFMT 79

Mechanism study 80

X-ray Crystal Structure Data for (*E*)-4-Nitrobenzaldehyde *O*-trifluoromethyl oxime (**1d**) (CCDC: 1907514).. 81

Supplementary Figure 87

Supplementary References 333

Supplementary Methods:

DMA, HMPA, MeCN and DCM etc. used in reactions were dried according to the purification handbook *Purification of Laboratory Chemicals* before using. H_2O was distilled. Cs_2CO_3 was purchased from J&K. CF_3SO_2Na and $PhI(OAc)_2$ were purchased from Bidepharm. TLC was performed on silica gel Huanghai HSGF254 plates and visualized by quenching of UV fluorescence (λ_{max} = 254 nm). 200-300 mesh silica gel was purchased from Qingdao Haiyang Chemical Co., China. Unless otherwise noted, all other reagents and starting materials were purchased from commercial sources and used without further purification. The data for NMR spectra (^1H NMR, ^{13}C NMR and ^{19}F NMR) were recorded at 293 K on a Bruker AVANCE AV 400 (400MHz, 101MHz and 376MHz) and chemical shifts were recorded relative to the solvent resonance. Signal positions were recorded in ppm and the following abbreviations were used singularly or in combination to indicate the multiplicity of signals: s singlet, d doublet, t triplet, q quartet, m multiplet, Hz Hertz. For ^1H NMR: $CDCl_3$ = δ 7.26 ppm, DMSO = δ 2.50 ppm. For ^{13}C NMR: $CDCl_3$ = δ 77.16 ppm, DMSO = δ 39.52 ppm. Mass spectra were obtained on Agilent 6520 Q-TOF LC/MS and Aligent 7890/5975C-GS/MSD. HRMS were obtained on Varian 7.0T FTMS.

Supplementary Note:

The Trifluoromethoxylating reaction is sensitive to water, but is not sensitive to oxygen, which can be operated outside the glove box. And the yield was influenced by the sizes of sealed vial tube.

Please be careful of the fluorophosgene which is formed through the decomposition of trifluoromethoxy anio

Effect of solvents on the reaction

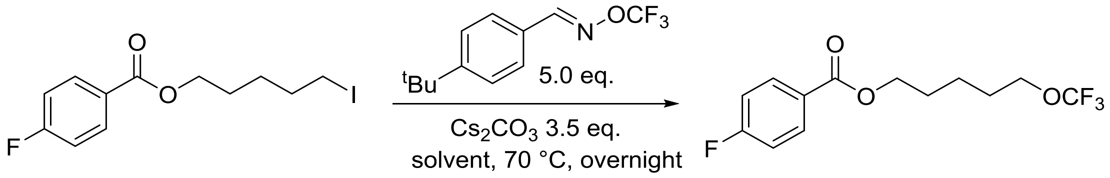

S3 **3**

In a N_2 glovebox, to 5-iodopentyl 4-fluorobenzoate (16.8 mg, 0.0500 mmol, 1.00 equiv.), (*E*)-*O*-trifluoromethyl-4-*tert*-butyl-benzaldoximes (**1a**) (61.3 mg, 0.250 mmol, 5.00 equiv.) in a 2.00 mL sealed vial were added solvent (0.400 mL). Cs_2CO_3 (57.0 mg, 0.175 mmol, 3.50 equiv.) was added to the reaction and the resulting mixture was stirred for overnight at 70 °C. After then, benzotrifluoride (6.0 μL, 0.0490 mmol) was added to the reaction mixture. The yield of 5-trifluoromethoxypentyl 4-fluorobenzoate was determined by comparing the integration of the ^{19}F NMR resonance of 5-trifluoromethoxypentyl 4-fluorobenzoate (-60.4 ppm) with that of benzotrifluoride (-62.8 ppm). Yields are reported in Supplementary Table 1.

Supplementary Table 1: Effect of solvents on the reaction

Solvents	Yield [%] (^{19}F NMR)	Solvents	Yield [%] (^{19}F NMR)
DMA	92	EA	0
DMF	27	MeCN	8
DMSO	1	Toluene	0
HMPA	80	DCM	0
CH_3NO_2	0	1.4-dioxane	0
THF	0	acetone	0

Effect of "OCF_3" sources on the reaction

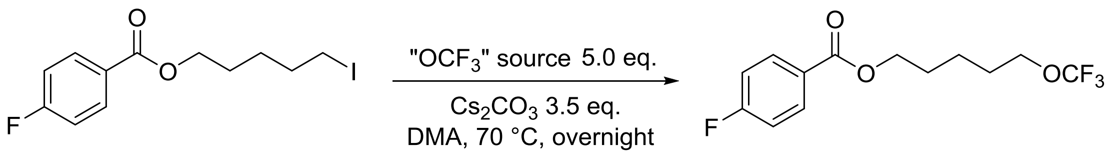

S3 **3**

In a N_2 glovebox, to 5-iodopentyl 4-fluorobenzoate (16.8 mg, 0.0500 mmol, 1.00 equiv.),

“OCF_3” source (0.250 mmol, 5.00 equiv.) in a 2.00 mL sealed vial were added DMA (0.400 mL). Cs_2CO_3 (57.0 mg, 0.175 mmol, 3.50 equiv.) was added to the reaction and the resulting mixture was stirred for overnight at 70 °C. After then, benzotrifluoride (6.0 µL, 0.0490 mmol) was added to the reaction mixture. The yield of 5-trifluoromethoxypentyl 4-fluorobenzoate was determined by comparing the integration of the ^{19}F NMR resonance of 5-trifluoromethoxypentyl 4-fluorobenzoate (-60.4 ppm) with that of benzotrifluoride (-62.8 ppm). Yields are reported in Supplementary Table 2.

Supplementary Table 2: Effect of “OCF_3” sources on the reaction

“OCF_3” sources	Yield [%] (^{19}F NMR)	“OCF_3” sources	Yield [%] (^{19}F NMR)
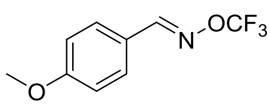	76	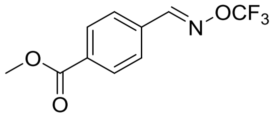	90
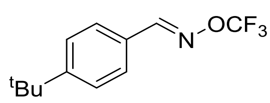	92	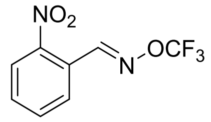	86
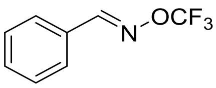	84	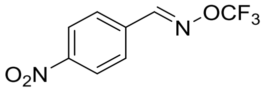	89
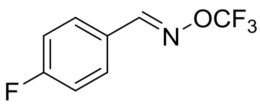	87	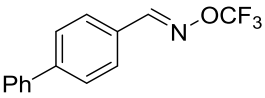	83
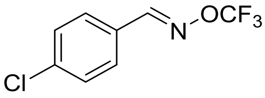	85	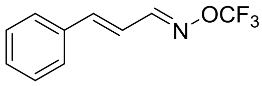	87
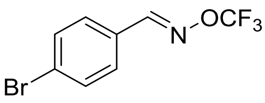	77	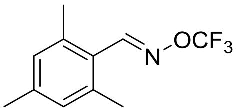	87
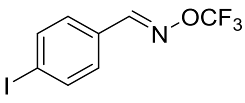	90	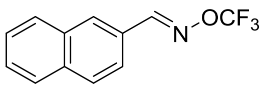	88
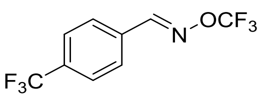	75	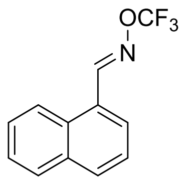	84
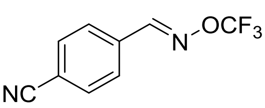	71		

Effect of temperature on the reaction

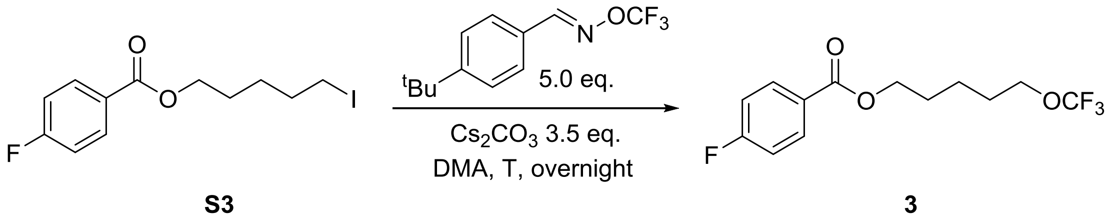

In a N_2 glovebox, to 5-iodopentyl 4-fluorobenzoate (16.8 mg, 0.0500 mmol, 1.00 equiv.), (*E*)-*O*-trifluoromethyl-4-*tert*-butyl-benzaldoximes (**1a**) (61.3 mg, 0.250 mmol, 5.00 equiv.) in a 2.00 mL sealed vial were added DMA (0.400 mL). Cs_2CO_3 (57.0 mg, 0.175 mmol, 3.50 equiv.) was added to the reaction and the resulting mixture was stirred for overnight at x °C. After then, benzotrifluoride (6.0 µL, 0.0490 mmol) was added to the reaction mixture. The yield of 5-trifluoromethoxypentyl 4-fluorobenzoate was determined by comparing the integration of the ^{19}F NMR resonance of 5-trifluoromethoxypentyl 4-fluorobenzoate (-60.4 ppm) with that of benzotrifluoride (-62.8 ppm). Yields are reported in Supplementary Table 3.

Supplementary Table 3: Effect of temperature on the reaction

Temperature	Yield [%] (^{19}F NMR)
30 °C	8
50 °C	76
70 °C	92
90 °C	44

Effect of concentration on the reaction

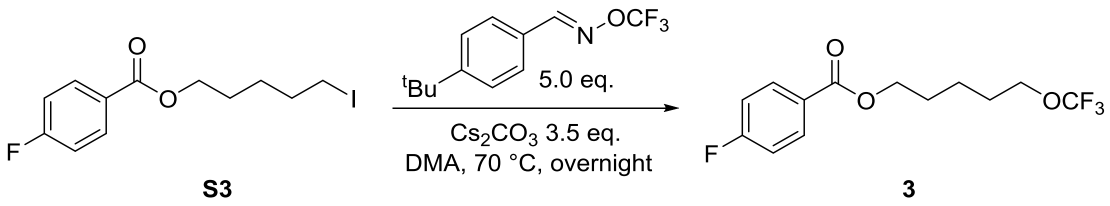

In a N_2 glovebox, to 5-iodopentyl 4-fluorobenzoate (16.8 mg, 0.0500 mmol, 1.00 equiv.), (*E*)-*O*-trifluoromethyl-4-*tert*-butyl-benzaldoximes (**1a**) (61.3 mg, 0.250 mmol, 5.00 equiv.) in a 2.00 mL sealed vial were added DMA. Cs_2CO_3 (57.0 mg, 0.175 mmol, 3.50 equiv.) was

added to the reaction and the resulting mixture was stirred for overnight at 70 °C. After then, benzotrifluoride (6.0 μL, 0.0490 mmol) was added to the reaction mixture. The yield of 5-trifluoromethoxypentyl 4-fluorobenzoate was determined by comparing the integration of the ^{19}F NMR resonance of 5-trifluoromethoxypentyl 4-fluorobenzoate (-60.4 ppm) with that of benzotrifluoride (-62.8 ppm). Yields are reported in Supplementary Table 4.

Supplementary Table 4: Effect of concentration on the reaction

Concentration	Yield [%] (^{19}F NMR)
0.250 M	86
0.125 M	92
0.0830 M	91
0.0630 M	88

Effect of bases on the reaction

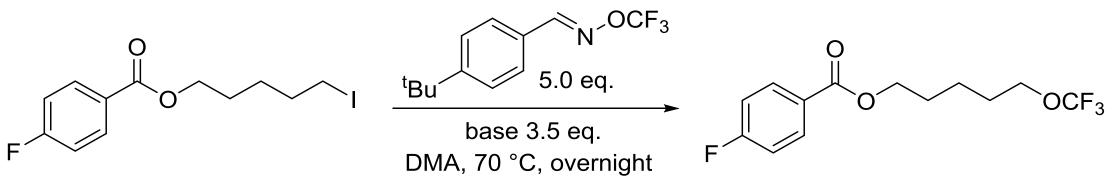

In a N_2 glovebox, to 5-iodopentyl 4-fluorobenzoate (16.8 mg, 0.0500 mmol, 1.00 equiv.), (*E*)-*O*-trifluoromethyl-4-*tert*-butyl-benzaldoximes (**1a**) (61.3 mg, 0.250 mmol, 5.00 equiv.) in a 2.00 mL sealed vial were added DMA (0.400 mL). Base (0.175 mmol, 3.50 equiv.) was added to the reaction and the resulting mixture was stirred for overnight at 70 °C. After then, benzotrifluoride (6.0 μL, 0.0490 mmol) was added to the reaction mixture. The yield of 5-trifluoromethoxypentyl 4-fluorobenzoate was determined by comparing the integration of the ^{19}F NMR resonance of 5-trifluoromethoxypentyl 4-fluorobenzoate (-60.4 ppm) with that of benzotrifluoride (-62.8 ppm). Yields are reported in Supplementary Table 5.

Supplementary Table 5: Effect of bases on the reaction

Bases	Yield [%] (^{19}F NMR)	Bases	Yield [%] (^{19}F NMR)
LiOH	0	Cs_2CO_3	92

$LiOCH_3$	0	$CsOCOCH_3$	37
KO^tBu	0	$CsOCOCF_3$	10
Na_2CO_3	0	$CsOCO^tBu$	18
K_2CO_3	7	K_3PO_4	0
Ag_2CO_3	10	Et_3N	43
Pyridine	0	DBU	0
CsF	56		

Effect of "OCF$_3$" amount on the reaction

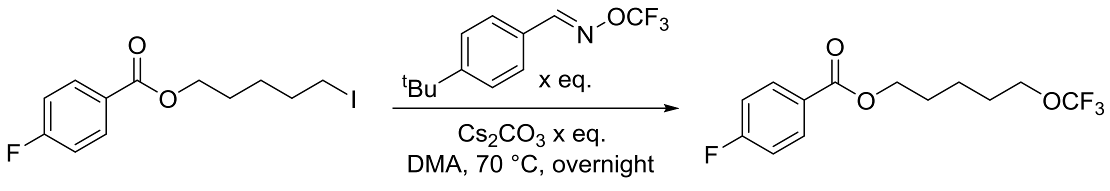

In a N_2 glovebox, to 5-iodopentyl 4-fluorobenzoate (16.8 mg, 0.0500 mmol, 1.00 equiv.), (*E*)-*O*-trifluoromethyl-4-*tert*-butyl-benzaldoximes (**1a**) in a 2.00 mL sealed vial were added DMA (0.400 mL). Cs_2CO_3 was added to the reaction and the resulting mixture was stirred for overnight at 70 °C. After then, benzotrifluoride (6.0 µL, 0.0490 mmol) was added to the reaction mixture. The yield of 5-trifluoromethoxypentyl 4-fluorobenzoate was determined by comparing the integration of the ^{19}F NMR resonance of 5-trifluoromethoxypentyl 4-fluorobenzoate (-60.4 ppm) with that of benzotrifluoride (-62.8 ppm). Yields are reported in Supplementary Table 6.

Supplementary Table 6: Effect of the amount of "OCF$_3$" on the reaction

Amount of "OCF$_3$" sources (equiv.)	Cs_2CO_3 (equiv.)	Yield [%] (^{19}F NMR)
2.0	1.0	48
3.0	1.8	55

3.0	2.0	53
4.0	2.5	82
4.0	3.0	85
5.0	3.0	83
5.0	3.5	92
6.0	4.0	83
6.0	4.5	89

Effect of reaction time on the reaction

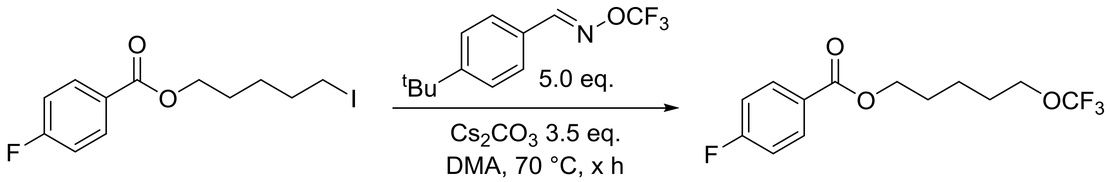

S3 **3**

In a N_2 glovebox, to 5-iodopentyl 4-fluorobenzoate (16.8 mg, 0.0500 mmol, 1.00 equiv.), (*E*)-*O*-trifluoromethyl-4-*tert*-butyl-benzaldoximes (**1a**) (61.3 mg, 0.250 mmol, 5.00 equiv.) in a 2.00 mL sealed vial were added DMA (0.400 mL). Cs_2CO_3 (57.0 mg, 0.175 mmol, 3.50 equiv.) was added to the reaction and the resulting mixture was stirred for x h at 70 °C. After then, benzotrifluoride (6.0 μL, 0.0490 mmol) was added to the reaction mixture. The yield of 5-trifluoromethoxypentyl 4-fluorobenzoate was determined by comparing the integration of the ^{19}F NMR resonance of 5-trifluoromethoxypentyl 4-fluorobenzoate (-60.4 ppm) with that of benzotrifluoride (-62.8 ppm). Yields are reported in Supplementary Table 7.

Supplementary Table 7: Effect of reaction time on the reaction

Reaction time	Yield [%] (^{19}F NMR)
6 h	85

8 h	89
10 h	92
12 h	92
14 h	92
16 h	90
18 h	91

Effect of H_2O amount on the reaction

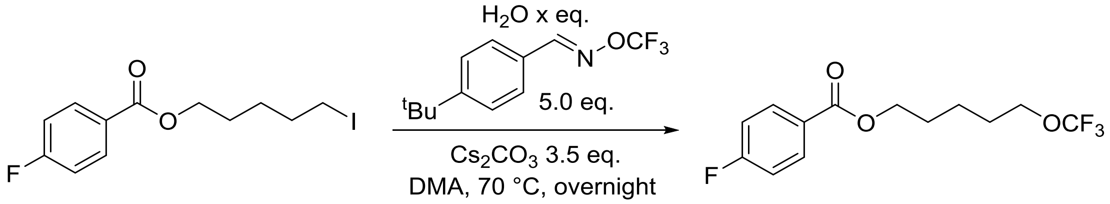

In a N_2 glovebox, to 5-iodopentyl 4-fluorobenzoate (16.8 mg, 0.0500 mmol, 1.00 equiv.), (*E*)-*O*-trifluoromethyl-4-*tert*-butyl-benzaldoximes (**1a**) (61.3 mg, 0.250 mmol, 5.00 equiv.) in a 2.00 mL sealed vial were added DMA (0.400 mL). Cs_2CO_3 (57.0 mg, 0.175 mmol, 3.50 equiv.), H_2O (x eq.) was added to the reaction and the resulting mixture was stirred for overnight at 70 °C. After then, benzotrifluoride (6.0 µL, 0.0490 mmol) was added to the reaction mixture. The yield of 5-trifluoromethoxypentyl 4-fluorobenzoate was determined by comparing the integration of the ^{19}F NMR resonance of 5-trifluoromethoxypentyl 4-fluorobenzoate (-60.4 ppm) with that of benzotrifluoride (-62.8 ppm). Yields are reported in Supplementary Table 8.

Supplementary Table 8: Effect of H_2O amount on the reaction

H_2O (equiv.)	Yield [%] (^{19}F NMR)
0	92
1.0	41
2.0	0

Effect of the volume of sealed vial on the reaction

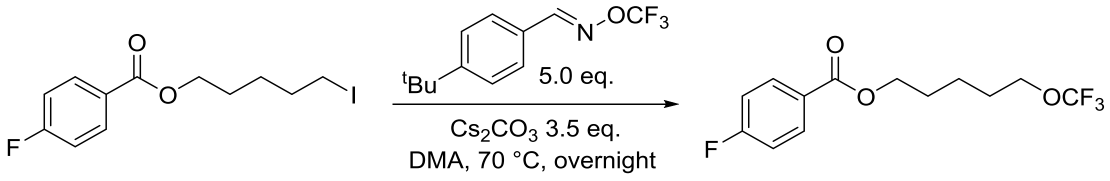

S3 3

In a N_2 glovebox, to 5-iodopentyl 4-fluorobenzoate (16.8 mg, 0.0500 mmol, 1.00 equiv.), (*E*)-*O*-trifluoromethyl-4-*tert*-butyl-benzaldoximes (**1a**) (61.3 mg, 0.250 mmol, 5.00 equiv.) in a x mL sealed vial were added DMA (0.400 mL). Cs_2CO_3 (57.0 mg, 0.175 mmol, 3.50 equiv.) was added to the reaction and the resulting mixture was stirred for overnight at 70 °C. After then, benzotrifluoride (6.0 μL, 0.0490 mmol) was added to the reaction mixture. The yield of 5-trifluoromethoxypentyl 4-fluorobenzoate was determined by comparing the integration of the ^{19}F NMR resonance of 5-trifluoromethoxypentyl 4-fluorobenzoate (-60.4 ppm) with that of benzotrifluoride (-62.8 ppm). Yields are reported in Supplementary Table 9.

Supplementary Table 9: Effect of the volume of sealed vial on the reaction

Volume of sealed vial	Yield [%] (^{19}F NMR)
2.0 mL	92
5.0 mL	72
15 mL	63

Effect of atmosphere on the reaction

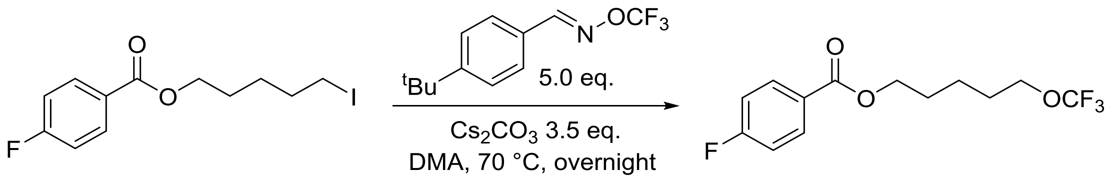

S3 3

To 5-iodopentyl 4-fluorobenzoate (16.8 mg, 0.0500 mmol, 1.00 equiv.), (*E*)-*O*-trifluoromethyl-4-*tert*-butyl-benzaldoximes (**1a**) (61.3 mg, 0.250 mmol, 5.00 equiv.) in a 2.0 mL sealed vial were added DMA (0.400 mL). Cs_2CO_3 (57.0 mg, 0.175 mmol, 3.50 equiv.) was added to the reaction and the resulting mixture was stirred for overnight at 70 °C. After then, benzotrifluoride (6.0 μL, 0.0490 mmol) was added to the reaction mixture. The yield of 5-trifluoromethoxypentyl 4-fluorobenzoate was determined by comparing the integration of the ^{19}F NMR resonance of 5-trifluoromethoxypentyl 4-fluorobenzoate (-60.4

ppm) with that of benzotrifluoride (-62.8 ppm). Yields are reported in Supplementary Table 10

Supplementary Table 10: Effect of atmosphere on the reaction

Atmosphere	Yield [%] (^{19}F NMR)
N_2	92
air	90
O_2	90

Supplementary Table 11: Scope of trifluoromethoxylating reagents

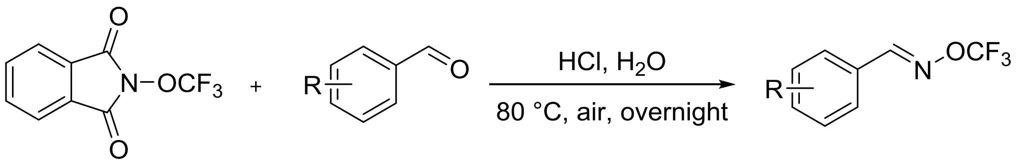

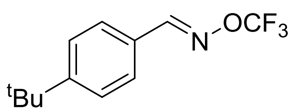

1a 59%[a](63%[b])

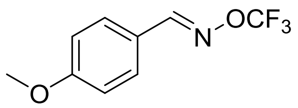

1b 54%[a]

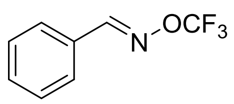

1c 11%[a](68%[b])

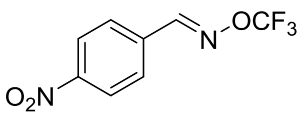

1d 51%[a]

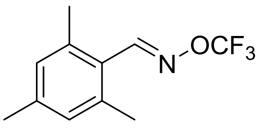

1e 53%[a]

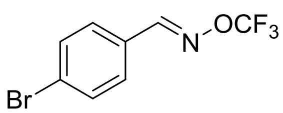

1f 54%[a]

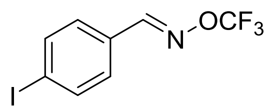

1g 41%[a]

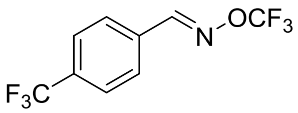

1h 32%[a](67%[b])

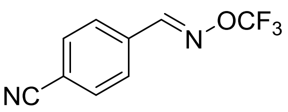

1i 54%[a]

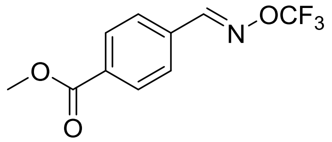

1j 36%[a](55%[b])

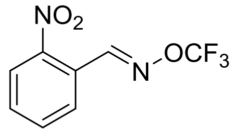

1k 45%[a](62%[b])

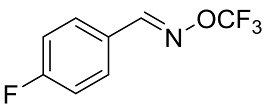

1l 22%[a](60%[b])

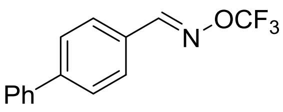

1m 51%[a]

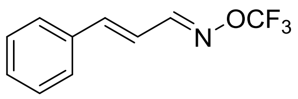

1n 15%[a]

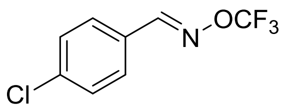

1o 58%[a]

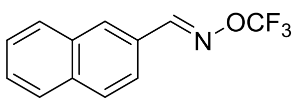

1p 33%[a](59%[b])

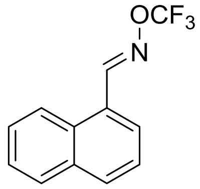

1q 57%[a]

a) Yields refer to isolated product. b) Yields were determined by ^{19}F NMR with benzotrifluoride (-62.8 ppm) as a standard.

2-(Trifluoromethoxy)isoindoline-1,3-dione (S1)

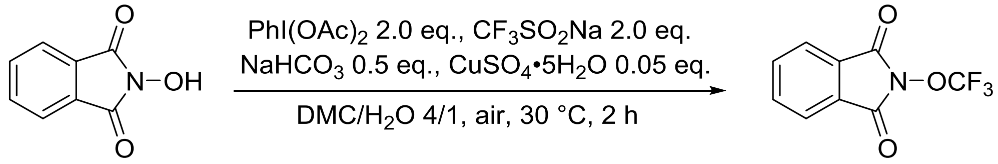

S1, 51%

In the air, to $NaHCO_3$ (2.57 g, 30.7 mmol, 0.500 equiv.), N-Hydroxyphthalimide (10.0 g, 61.3 mmol, 1.00 equiv.), CF_3SO_2Na (19.1 g, 123 mmol, 2.00 equiv.), $PhI(OAc)_2$ (39.5 g, 123 mmol, 2.00 equuiv.), $CuSO_4$•$5H_2O$ (737 mg, 3.47 mmol, 0.0500 equiv.) in a round-bottom flask, were added H_2O (48.0 mL) and DMC (Dimethyl carbonate) (196.0 mL) at 0 °C. The reaction mixture was stirred at 0 °C for 5 mins. Then the reaction was stirred at room temperature for 2 h. The aqueous layer was separated and extracted 2 times with EtOAc (100 mL). The combined organic layer was washed $NaHCO_3$ (aq.), dried over anhydrous $MgSO_4$, filtered and concentrated in *vacuo*. The residue was purified by preparative silica gel chromatography, eluting with *n*-hexane/acetone 15:1 (v/v) to afford 7.20 g product 2-(Trifluoromethoxy)isoindoline-1,3-dione (**S1)** as a solid (51 % yield).

R_f = 0.3 (*n*-hexane/acetone 10/1 (v/v)). NMR Spectroscopy: ^{1}H NMR (400 MHz, $CDCl_3$) δ 7.95 – 7.91 (m, 2H), 7.88 – 7.83 (m, 2H). ^{13}C NMR (101 MHz, $CDCl_3$) δ 161.7, 135.6, 128.6, 124.6, 122.2 (d, J = 268.3 Hz). ^{19}F NMR (400 MHz, $CDCl_3$) δ -65.0 (s, 3F). **S1** is a known compound and spectral data match the reported literature values.[1]

(*E*)-*O*-Trifluoromethyl-4-*tert*-butyl-benzaldoximes (1a)

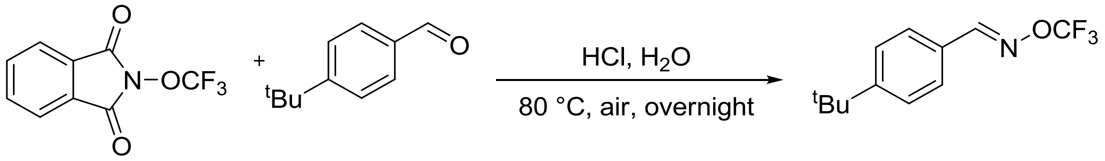

S1 **1a,** 59%

In a round-bottom flask, $PhthNOCF_3$ (**S1**) (2-(trifluoromethoxy)isoindoline-1,3-dione) (1.00 g, 4.33 mmol, 1.00 equiv.), water (4.00 mL), HCl (0.720 mL ca. 6.00 M aq., 4.33 mmol, 1.00 equiv.) and 4-(*tert*-butyl)benzaldehyde (1.09 mL, 6.50 mmol, 1.50 equiv.) were added. The mixture was stirred at 80 °C overnight. Afterwards, the reaction mixture was extracted with CH_2Cl_2 (20.0 mL×2). The combined organic layer was dried over anhydrous $MgSO_4$, filtered and concentrated. The residue was purified by silica gel chromatography, eluting with *n*-hexane/EtOAc 200:1 (v/v) to afford 626 mg (*E*)-*O*-trifluoromethyl-4-*tert*-butyl-benzaldoximes (**1a**) as a colorless liquid (59% yield).

R_f = 0.7 (*n*-hexane). NMR Spectroscopy: ^{1}H NMR (400 MHz, $CDCl_3$) δ 8.23 (s, 1H), 7.63 – 7.57 (m, 2H), 7.48 – 7.42 (m, 2H), 1.34 (s, 9H). ^{13}C NMR (101 MHz, $CDCl_3$) δ 155.7, 128.1, 126.7, 126.1, 122.5 (q, J = 258.9 Hz), 100.1, 35.2, 31.2. ^{19}F NMR (400 MHz, $CDCl_3$) δ -63.7 (s, 3F). Mass Spectrometry: HRMS-EI (m/z): Calcd for $C_{12}H_{14}F_3NO$ [M], 245.1027. Found, 260.1021.

(*E*)-*O*-Trifluoromethyl-4-methoxybenzaldoximes (1b)

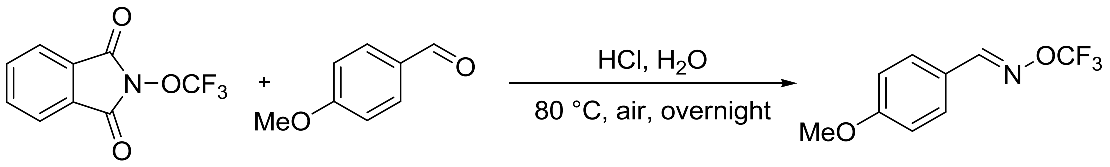

S1 **1b,** 54%

In a round-bottom flask, PhthNOCF$_3$ (**S1**) (2-(trifluoromethoxy)isoindoline-1,3-dione) (1.00 g, 4.33 mmol, 1.00 equiv.), water (4.00 mL), HCl (0.720 mL ca. 6.00 M aq., 4.33 mmol, 1.00 equiv.) and 4-methoxybenzaldehyde (0.788 mL, 6.49 mmol, 1.50 equiv.) were added. The mixture was stirred at 80 °C overnight. Afterwards, the reaction mixture was extracted with CH$_2$Cl$_2$ (20.0 mL×2). The combined organic layer was dried over anhydrous MgSO$_4$, filtered and concentrated. The residue was purified by silica gel chromatography, eluting with *n*-hexane/CH$_2$Cl$_2$ 10:1 (v/v) to afford 512 mg (*E*)-*O*-Trifluoromethyl-4-methoxybenzaldoximes (**1b**) as a colorless liquid (54% yield).

R$_f$ = 0.8 (*n*-hexane/CH$_2$Cl$_2$ 1:1 (v/v)). NMR Spectroscopy: ^{1}H NMR (400 MHz, CDCl$_3$) δ 8.19 (s, 1H), 7.61 (d, *J* = 8.7 Hz, 2H), 6.94 (d, *J* = 8.7 Hz, 2H), 3.86 (s, 3H). ^{13}C NMR (101 MHz, CDCl$_3$) δ 162.7, 155.4, 130.0, 122.5 (q, *J* = 258.4 Hz), 122.0, 114.6, 55.5. ^{19}F NMR (376 MHz, CDCl$_3$) δ -63.2 (s, 3F). Mass Spectrometry: HRMS-EI (m/z): Calcd for C$_9$H$_8$F$_3$NO$_2$ [M], 219.0507. Found, 219.0505.

(*E*)-*O*-Trifluoromethyl-benzaldoximes (1c)

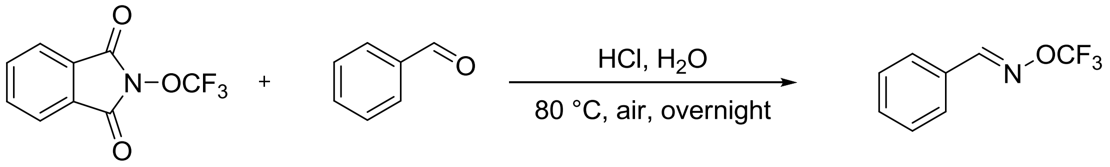

S1 **1c,** 11%

In a round-bottom flask, PhthNOCF$_3$ (**S1**) (2-(trifluoromethoxy)isoindoline-1,3-dione) (1.00 g, 4.33 mmol, 1.00 equiv.), water (3.60 mL), HCl (0.720 mL ca. 6.00 M aq., 4.33 mmol, 1.00 equiv.) and benzaldehyde (0.660 mL, 6.50 mmol, 1.50 equiv.) were added. The mixture was stirred at 80 °C overnight. Afterwards, the reaction mixture was extracted with CH$_2$Cl$_2$ (20.0 mL×2). The combined organic layer was dried over anhydrous MgSO$_4$, filtered and concentrated. The residue was purified by silica gel chromatography, eluting with *n*-hexane to afford 86.8 mg (*E*)-*O*-Trifluoromethyl-benzaldoximes (**1c**) as a colorless liquid (11% yield).

R$_f$ = 0.5 (*n*-hexane). NMR Spectroscopy: ^{1}H NMR (400 MHz, CDCl$_3$) δ 8.05 (s, 1H), 7.51 – 7.44 (m, 2H), 7.33 – 7.20 (m, 3H). ^{13}C NMR (101 MHz, CDCl$_3$) δ 155.6, 131.9, 129.4, 129.0, 128.2, 124.7 (q, J = 238.4 Hz). ^{19}F NMR (376 MHz, CDCl$_3$) δ -63.5 (s, 3F). Mass Spectrometry: HRMS-EI (m/z): Calcd for C$_8$H$_6$F$_3$NO [M], 189.0401. Found, 189.0394.

(*E*)-*O*-Trifluoromethyl-4-nitrobenzaldoximes (1d)

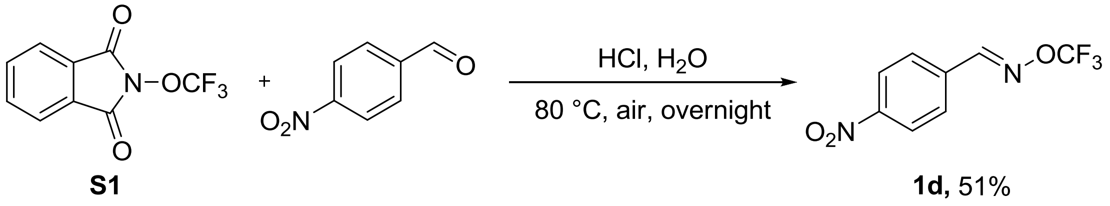

In a round-bottom flask, $PhthNOCF_3$ (**S1**) (2-(trifluoromethoxy)isoindoline-1,3-dione) (1.00 g, 4.33 mmol, 1.00 equiv.), water (3.60 mL), HCl (0.720 mL ca. 6.00 M aq., 4.33 mmol, 1.00 equiv.) and 4-nitrobenzaldehyde (980 mg, 6.50 mmol, 1.50 equiv.) were added. The mixture was stirred at 80 °C overnight. Afterwards, the reaction mixture was extracted with CH_2Cl_2 (20.0 mL×2). The combined organic layer was dried over anhydrous $MgSO_4$, filtered and concentrated. The residue was purified by silica gel chromatography, eluting with *n*-hexane/EtOAc 10:1 (v/v) to afford 520 mg (*E*)-*O*-Trifluoromethyl-4-nitrobenzaldoximes (**1d**) as a white solid (51% yield).

R_f = 0.5 (*n*-hexane/EtOAc 5:1 (v/v)). NMR Spectroscopy: ^1H NMR (400 MHz, $CDCl_3$) δ 8.34 (s, 1H), 8.31 (d, *J* = 8.8 Hz, 2H), 7.88 (d, *J* = 8.8 Hz, 2H). ^{13}C NMR (101 MHz, $CDCl_3$) δ 153.6, 149.8, 135.4, 132.2, 129.1, 124.3, 122.4(q, *J* = 260.5 Hz). ^{19}F NMR (376 MHz, $CDCl_3$) δ -64.0 (s, 3F). Mass Spectrometry: HRMS-EI (m/z): Calcd for $C_8H_5F_3N_2O_3$ [M], 234.0252. Found, 234.0245.

(*E*)-*O*-Trifluoromethyl-2,4,6-trimethylbenzaldoximes (1e)

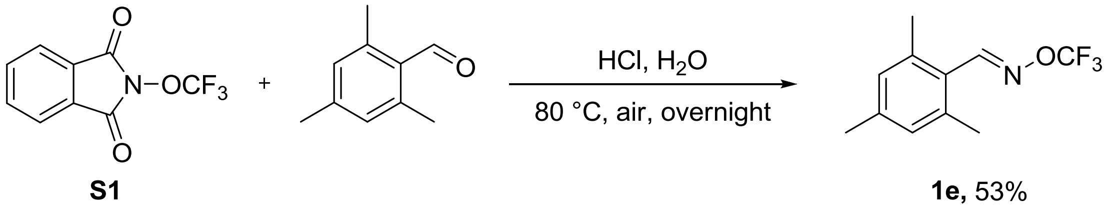

In a round-bottom flask, $PhthNOCF_3$ (**S1**) (2-(trifluoromethoxy)isoindoline-1,3-dione) (1.00 g, 4.33 mmol, 1.00 equiv.), water (3.60 mL), HCl (0.720 mL ca. 6.00 M aq., 4.33 mmol, 1.00 equiv.) and 2,4,6-trimethylbenzaldehyde (0.960 mL, 6.50 mmol, 1.50 equiv.) were added. The mixture was stirred at 80 °C overnight. Afterwards, the reaction mixture was extracted with CH_2Cl_2 (20.0 mL×2). The combined organic layer was dried over anhydrous $MgSO_4$, filtered and concentrated. The residue was purified by silica gel chromatography, eluting with *n*-hexane to afford 0.530 g (*E*)-*O*-Trifluoromethyl-2,4,6-trimethylbenzaldoximes (**1e**) as a white solid (53% yield).

R_f = 0.6 (*n*-hexane). NMR Spectroscopy: ^1H NMR (400 MHz, $CDCl_3$) δ 8.57 (s, 1H), 6.92 (s, 2H), 2.41 (s, 6H), 2.30 (s, 3H). ^{13}C NMR (101 MHz, $CDCl_3$) δ 155.2, 141.0, 138.8, 129.9, 124.7 (q, *J* = 229.6 Hz), 124.0, 31.2, 21.4. ^{19}F NMR (376 MHz, $CDCl_3$) δ -63.6 (s, 3F). Mass Spectrometry: HRMS-EI (m/z): Calcd for $C_{11}H_{12}F_3NO$ [M], 231.0871. Found, 231.0865.

(*E*)-*O*-Trifluoromethyl-4-bromobenzaldoximes (1f)

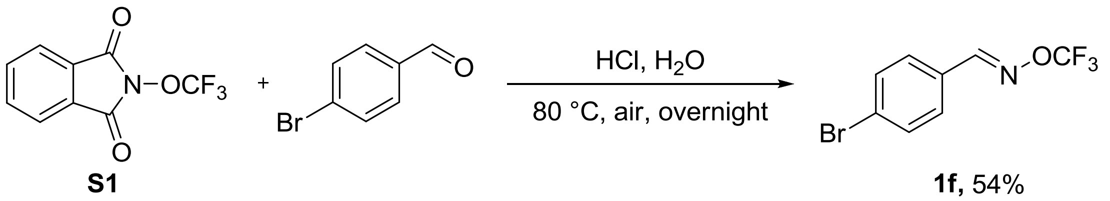

In a round-bottom flask, PhthNOCF$_3$ (**S1**) (2-(trifluoromethoxy)isoindoline-1,3-dione) (1.00 g, 4.33 mmol, 1.00 equiv.), water (3.60 mL), HCl (0.720 mL ca. 6.00 M aq., 4.33 mmol, 1.00 equiv.) and 4-bromorobenzaldehyde (1.20 g, 6.50 mmol, 1.50 equiv.) were added. The mixture was stirred at 80 °C overnight. Afterwards, the reaction mixture was extracted with CH_2Cl_2 (20.0 mL×2). The combined organic layer was dried over anhydrous $MgSO_4$, filtered and concentrated. The residue was purified by silica gel chromatography, eluting with *n*-hexane to afford 625 mg (*E*)-*O*-Trifluoromethyl-4-bromobenzaldoximes (**1f**) as a colorless liquid (54% yield).

R_f = 0.5 (*n*-hexane). NMR Spectroscopy: ^{1}H NMR (400 MHz, CDCl$_3$) δ 8.19 (s, 1H), 7.62 – 7.49 (m, 4H). ^{13}C NMR (101 MHz, CDCl$_3$) δ 154.7, 132.5, 129.6, 128.5, 126.6, 122.4 (q, J = 259.5 Hz). ^{19}F NMR (376 MHz, DMSO) δ -63.7 (s, 3F). Mass Spectrometry: HRMS-EI (m/z): Calcd for $C_8H_5BrF_3NO$ [M], 266.9507. Found, 266.9502.

(*E*)-*O*-Trifluoromethyl-4-iodobenzaldoximes (1g)

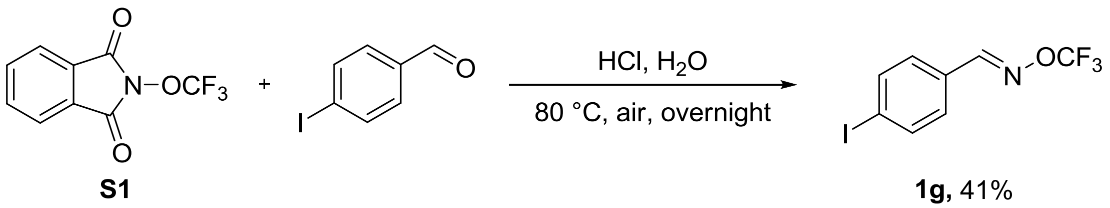

In a round-bottom flask, PhthNOCF$_3$ (**S1**) (2-(trifluoromethoxy)isoindoline-1,3-dione) (1.00 g, 0.580 mmol, 1.00 equiv.), water (3.60 mL), HCl (0.720 mL ca. 6.00 M aq., 4.33 mmol, 1.00 equiv.) and 4-iodobenzaldehyde (1.51 g, 6.50 mmol, 1.50 equiv.) were added. The mixture was stirred at 80 °C overnight. Afterwards, the reaction mixture was extracted with CH_2Cl_2 (5.0 mL×2). The combined organic layer was dried over anhydrous $MgSO_4$, filtered and concentrated. The residue was purified by silica gel chromatography, eluting with *n*-hexane to afford 553 mg (*E*)-*O*-Trifluoromethyl-4-iodobenzaldoximes (**1g**) as a colorless liquid (41% yield).

R_f = 0.5 (*n*-hexane). NMR Spectroscopy: ^{1}H NMR (400 MHz, CDCl$_3$) δ 8.18 (s, 1H), 7.84 – 7.70 (m, 2H), 7.44 – 7.34 (m, 2H). ^{13}C NMR (101 MHz, CDCl$_3$) δ 154.8, 138.3, 129.4, 128.9, 122.3 (d, J = 259.4 Hz), 98.7. ^{19}F NMR (376 MHz, CDCl$_3$) δ -63.6 (s, 3F). Mass Spectrometry: HRMS-EI (m/z): Calcd for $C_8H_5F_3INO$ [M], 314.9368. Found, 314.9356.

(*E*)-*O*-Trifluoromethyl-4-trifluoromethylbenzaldoximes (1h)

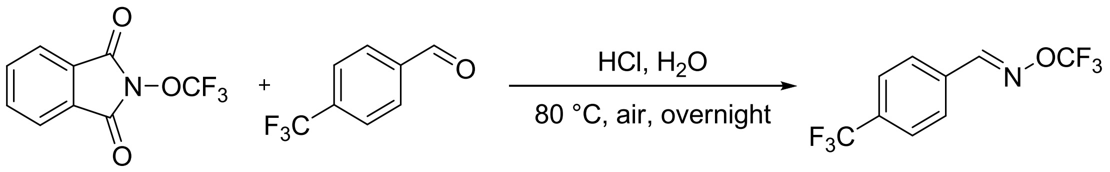

In a round-bottom flask, PhthNOCF$_3$ (**S1**) (2-(trifluoromethoxy)isoindoline-1,3-dione) (1.00 g, 4.33 mmol, 1.00 equiv.), water (3.60 mL), HCl (0.720 mL ca. 6.00 M aq., 4.33 mmol, 1.00 equiv.) and 4-trifluoromethylbenzaldehyde (0.880 mL, 6.50 mmol, 1.50 equiv.) were added. The mixture was stirred at 80 °C overnight. Afterwards, the reaction mixture was extracted with CH_2Cl_2 (20.0 mL×2). The combined organic layer was dried over anhydrous $MgSO_4$, filtered and concentrated. The residue was purified by silica gel chromatography, eluting with *n*-hexane to afford 352 mg (*E*)-*O*-Trifluoromethyl-4-(trifluoromethyl)benzaldoximes (**1h**) as a colorless liquid (32% yield).

R_f = 0.5 (*n*-hexane). NMR Spectroscopy: ^{1}H NMR (400 MHz, $CDCl_3$) δ 8.30 (s, 1H), 7.83 – 7.77 (m, 2H), 7.73 – 7.68 (m, 2H). ^{13}C NMR (101 MHz, $CDCl_3$) δ 154.2, 133.5 (q, *J* = 32.8 Hz), 132.8, 128.4, 126.0 (q, *J* = 3.8 Hz), 123.5 (q, *J* = 272.5 Hz), 122.3 (q, *J* = 260.0 Hz). ^{19}F NMR (376 MHz, $CDCl_3$) δ -63.4 (s, 3F), -63.9 (s, 3F). Mass Spectrometry: HRMS-EI (m/z): Calcd for $C_9H_5F_6NO$ [M], 257.0275. Found, 257.0265.

(*E*)-*O*-Trifluoromethyl-4-cyanobenzaldoximes (1i)

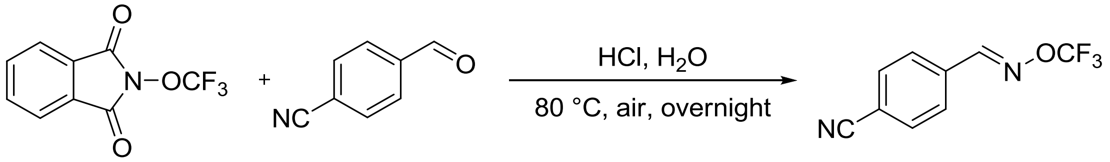

In a round-bottom flask, PhthNOCF$_3$ (**S1**) (2-(trifluoromethoxy)isoindoline-1,3-dione) (1.00 g, 4.33 mmol, 1.00 equiv.), water (3.60 mL), HCl (0.720 mL ca. 6.00 M aq., 4.33 mmol, 1.00 equiv.) and 4-formylbenzonitrile (851 mg, 6.50 mmol, 1.50 equiv.) were added. The mixture was stirred at 80 °C overnight. Afterwards, the reaction mixture was extracted with CH_2Cl_2 (20.0 mL×2). The combined organic layer was dried over anhydrous $MgSO_4$, filtered and concentrated. The residue was purified by silica gel chromatography, eluting with *n*-hexane/EtOAc 50: 1 (v/v) to afford 497 mg (*E*)-*O*-Trifluoromethyl-4-cyanobenzaldoximes (**1i**) as a white solid (54% yield).

R_f = 0.15 (*n*-hexane/EtOAc 1:50 (v/v)). NMR Spectroscopy: ^{1}H NMR (400 MHz, $CDCl_3$) δ 8.29 (s, 1H), 7.80 (m, 2H), 7.74 (m, 2H). ^{13}C NMR (101 MHz, $CDCl_3$) δ 153.8, 133.6, 132.7, 128.6, 122.2 (q, *J* = 260.5 Hz), 117.9, 115.3. ^{19}F NMR (376 MHz, $CDCl_3$) δ -64.0 (s, 3F). Mass Spectrometry: HRMS-EI (m/z): Calcd for $C_9H_5F_3N_2O$ [M], 214.0354. Found, 214.0346.

(*E*)-*O*-Trifluoromethyl-4-methoxycarbonylbenzaldoximes (1j)

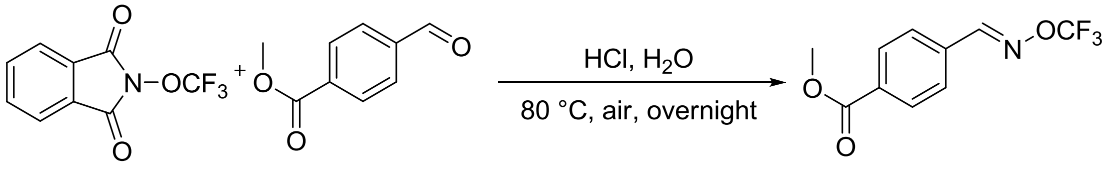

In a round-bottom flask, PhthNOCF$_3$ (**S1**) (2-(trifluoromethoxy)isoindoline-1,3-dione) (1.00 g, 4.33 mmol, 1.00 equiv.), water (3.60 mL), HCl (0.720 mL ca. 6.00 M aq., 4.33 mmol, 1.00 equiv.) and methyl 4-formylbenzoate (1.07 g, 6.50 mmol, 1.50 equiv.) were added. The mixture was stirred at 80 °C overnight. Afterwards, the reaction mixture was extracted with CH$_2$Cl$_2$ (20.0 mL×2). The combined organic layer was dried over anhydrous MgSO$_4$, filtered and concentrated. The residue was purified by silica gel chromatography, eluting with *n*-hexane/EtOAc 1:50 (v/v) to afford 382 mg (*E*)-*O*-Trifluoromethyl-4-methoxycarbonylbenzaldoximes (**1j**) as a white solid (36% yield).

R$_f$ = 0.3 (*n*-hexane/EtOAc 1:50 (v/v)). NMR Spectroscopy: ^{1}H NMR (400 MHz, CDCl$_3$) δ 8.29 (s, 1H), 8.10 (d, *J* = 8.4 Hz, 2H), 7.74 (d, *J* = 8.4 Hz, 2H), 3.94 (s, 3H). ^{13}C NMR (101 MHz, CDCl$_3$) δ 166.3, 154.8, 133.6, 133.1, 130.3, 128.2, 122.4(q, *J* = 259.7 Hz), 52.6. ^{19}F NMR (376 MHz, DMSO) δ -63.6 (s, 3F). Mass Spectrometry: HRMS-EI (m/z): Calcd for C$_{10}$H$_8$F$_3$NO$_3$ [M], 247.0456. Found, 247.0448.

(*E*)-*O*-Trifluoromethyl-2-nitrobenzaldoximes (1k)

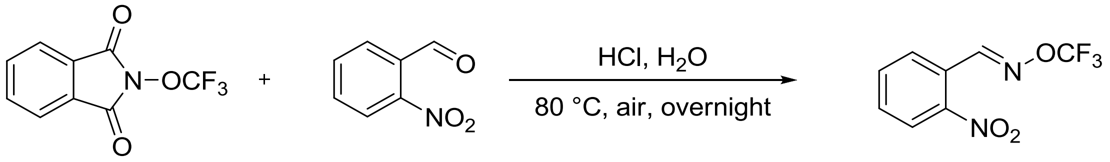

In a round-bottom flask, PhthNOCF$_3$ (**S1**) (2-(trifluoromethoxy)isoindoline-1,3-dione) (1.00 g, 4.33 mmol, 1.00 equiv.), water (3.60 mL), HCl (0.720 mL ca. 6.00 M aq., 4.33 mmol, 1.00 equiv.) and 2-nitrobenzaldehyde (0.980 g, 6.50 mmol, 1.50 equiv.) were added. The mixture was stirred at 80 °C overnight. Afterwards, the reaction mixture was extracted with CH$_2$Cl$_2$ (20.0 mL×2). The combined organic layer was dried over anhydrous MgSO$_4$, filtered and concentrated. The residue was purified by silica gel chromatography, eluting with *n*-hexane/EtOAc 10:1 (v/v) to afford 496 mg (*E*)-*O*-Trifluoromethyl-2-nitrobenzaldoximes (**1k**) as a colorless liquid (45% yield).

R$_f$ = 0.2 (*n*-hexane/EtOAc 10:1 (v/v)). ^{1}H NMR (400 MHz, CDCl$_3$) δ 8.91 (s, 1H), 8.27 – 8.13 (m, 1H), 8.02 – 7.89 (m, 1H), 7.81 – 7.59 (m, 2H). ^{13}C NMR (101 MHz, CDCl$_3$) δ 153.1, 134.3, 132.3, 129.9, 127.6, 125.4, 125.0, 122.4 (q, *J* = 260.4 Hz). ^{19}F NMR (376 MHz, DMSO) δ -64.1 (s, 3F). Mass Spectrometry: HRMS-EI (m/z): Calcd for C$_8$H$_5$F$_3$N$_2$O$_3$ [M], 234.0252. Found, 234.0248.

(*E*)-*O*-Trifluoromethyl-4-fluorobenzaldoximes (1l)

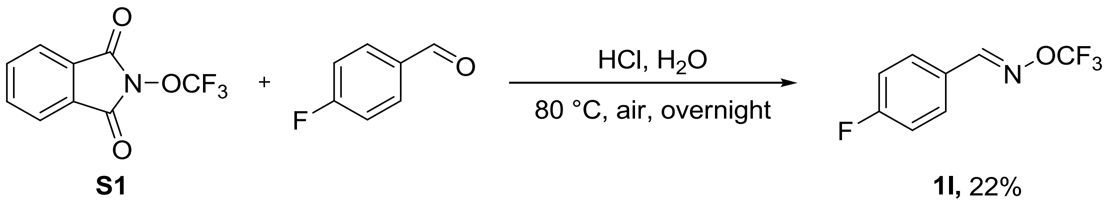

In a round-bottom flask, PhthNOCF$_3$ (**S1**) (2-(trifluoromethoxy)isoindoline-1,3-dione) (1.00 g, 4.33 mmol, 1.00 equiv.), water (3.60 mL), HCl (0.720 mL ca. 6.00 M aq., 4.33 mmol, 1.00 equiv.) and 4-fluorobenzaldehyde (0.690 mL, 6.50 mmol, 1.50 equiv.) were added. The mixture was stirred at 80 °C overnight. Afterwards, the reaction mixture was extracted with CH_2Cl_2 (20.0 mL×2). The combined organic layer was dried over anhydrous $MgSO_4$, filtered and concentrated. The residue was purified by silica gel chromatography, eluting with *n*-hexane to afford 194 mg (*E*)-*O*-Trifluoromethyl-4-fluorobenzaldoximes (**1l**) as a colorless liquid (22% yield).

R_f = 0.5 (*n*-hexane). NMR Spectroscopy: ^{1}H NMR (400 MHz, $CDCl_3$) δ 8.23 (s, 1H), 7.83 – 7.58 (m, 2H), 7.18 – 7.09 (m, 2H). ^{13}C NMR (101 MHz, $CDCl_3$) δ 164.9 (d, J = 253.2 Hz), 154.4, 130.2 (d, J = 8.8 Hz), 125.7, 122.3 (q, J = 259.3 Hz), 116.3 (d, J = 22.1 Hz). ^{19}F NMR (376 MHz, $CDCl_3$) δ -63.8 (s, 3F), -107.0 (s, 1F). Mass Spectrometry: HRMS-EI (m/z): Calcd for $C_8H_5F_4NO$ [M], 207.0307. Found,207.0299.

(*E*)-*O*-Trifluoromethyl-4-phenylbenzaldoximes (1m)

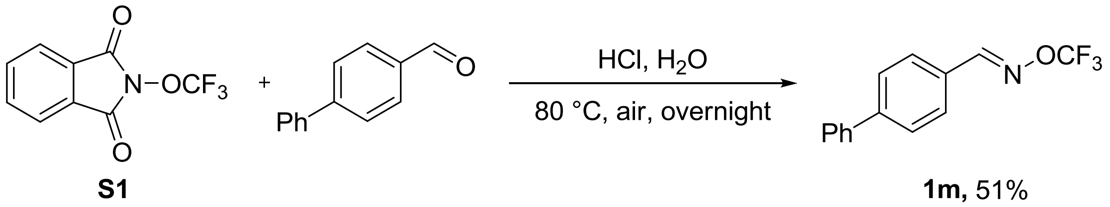

In a round-bottom flask, PhthNOCF$_3$ (**S1**) (2-(trifluoromethoxy)isoindoline-1,3-dione) (1.00 g, 4.33 mmol, 1.00 equiv.), water (3.60 mL), HCl (0.720 mL ca. 6.00 M aq., 4.33 mmol, 1.00 equiv.) and [1,1'-biphenyl]-4-carbaldehyde (1.18 g, 6.50 mmol, 1.50 equiv.) were added. The mixture was stirred at 80 °C overnight. Afterwards, the reaction mixture was extracted with CH_2Cl_2 (20.0 mL×2). The combined organic layer was dried over anhydrous $MgSO_4$, filtered and concentrated. The residue was purified by silica gel chromatography, eluting with *n*-hexane to afford 580 mg (*E*)-*O*-Trifluoromethyl-4-phenylbenzaldoximes (**1m**) as a white solid (51% yield).

R_f = 0.2 (*n*-hexane). NMR Spectroscopy: ^{1}H NMR (400 MHz, $CDCl_3$) δ 8.29 (s, 1H), 7.75 (m, 2H), 7.68 (m, 2H), 7.62 (m, 2H), 7.48 (m, 2H), 7.40 (m, 1H). ^{13}C NMR (101 MHz, $CDCl_3$) δ 155.5, 144.8, 140.0, 129.1, 128.8, 128.4, 128.3, 127.8, 127.3, 122.5 (q, J = 259.1 Hz). ^{19}F NMR (376 MHz, $CDCl_3$) δ -63.6 (s, 3F). Mass Spectrometry: HRMS-EI (m/z): Calcd for $C_{14}H_{10}F_3NO$ [M], 265.0714. Found, 265.0707.

(1*E*,2*E*)-*O*-Trifluoromethyl-cinnamaldoxime (1n)

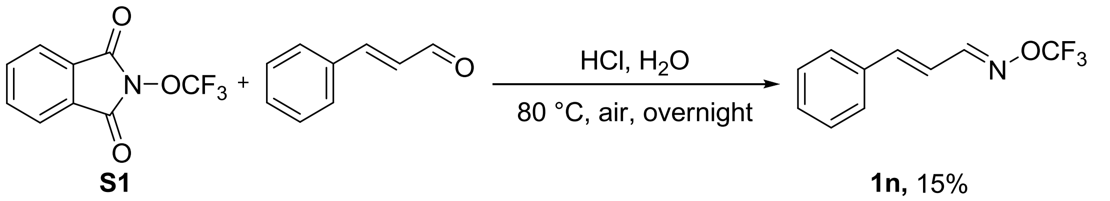

In a round-bottom flask, PhthNOCF$_3$ **S1**) (2-(trifluoromethoxy)isoindoline-1,3-dione) (1.00 g, 4.33 mmol, 1.00 equiv.), water (3.60 mL), HCl (0.720 mL ca. 6.00 M aq., 4.33 mmol, 1.00 equiv.) and cinnamaldehyde (0.820 mL, 6.50 mmol, 1.50 equiv.) was added. The mixture was stirred at 80 °C overnight. Afterwards, the reaction mixture was extracted with CH_2Cl_2 (20.0 mL×2). The combined organic layer was dried over anhydrous $MgSO_4$, filtered and concentrated. The residue was purified by silica gel chromatography, eluting with *n*-hexane to afford 138 mg (1*E*,2*E*)-*O*-trifluoromethyl-cinnamaldoxime (**1n**) as a colorless liquid (15% yield).

R_f = 0.2 (*n*-hexane). NMR Spectroscopy: ^{1}H NMR (400 MHz, CDCl$_3$) δ 8.04 (dd, *J* = 9.9, 1.4 Hz, 1H), 7.52 – 7.46 (m, 2H), 7.44 – 7.37 (m, 3H), 7.02 (d, *J* = 16.1 Hz, 1H), 6.86 (dd, *J* = 16.0, 9.9 Hz, 1H). ^{13}C NMR (101 MHz, CDCl$_3$) δ 157.2, 144.2, 135.0, 130.2, 129.1, 127.6, 122.4 (q, *J* = 258.9 Hz), 119.2. ^{19}F NMR (376 MHz, CDCl$_3$) δ -63.7 (s, 3F). Mass Spectrometry: HRMS-EI (m/z): Calcd for $C_{10}H_8F_3NO$ [M], 215.0558. Found, 215.0554.

(*E*)-*O*-Trifluoromethyl-4-chlorobenzaldoximes (1o)

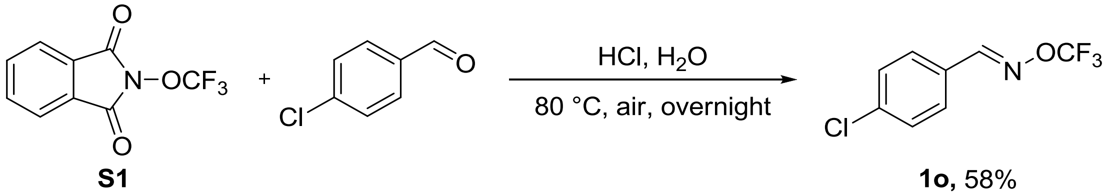

In a round-bottom flask, PhthNOCF$_3$ (**S1**) (2-(trifluoromethoxy)isoindoline-1,3-dione) (1.00 g, 4.33 mmol, 1.00 equiv.), water (3.60 mL), HCl (0.720 mL ca. 6.00 M aq., 4.33 mmol, 1.00 equiv.) and 4-chlororobenzaldehyde (910 mg, 6.50 mmol, 1.50 equiv.) were added. The mixture was stirred at 80 °C overnight. Afterwards, the reaction mixture was extracted with CH_2Cl_2 (20.0 mL×2). The combined organic layer was dried over anhydrous $MgSO_4$, filtered and concentrated. The residue was purified by silica gel chromatography, eluting with *n*-hexane to afford 565 mg (*E*)-*O*-Trifluoromethyl-4-chlorobenzaldoximes (**1o**) as a colorless liquid (58% yield).

R_f = 0.5 (*n*-hexane). NMR Spectroscopy: ^{1}H NMR (400 MHz, CDCl$_3$) δ 8.20 (s, 1H), 7.60 (d, *J* = 8.4 Hz, 2H), 7.41 (d, *J* = 8.4 Hz, 2H). ^{13}C NMR (101 MHz, CDCl$_3$) δ 154.6, 138.2, 129.5, 129.5, 128.1, 122.5 (q, *J* = 259.6 Hz). ^{19}F NMR (376 MHz, DMSO) δ -64.0 (s, 3F). Mass Spectrometry: HRMS-EI (m/z): Calcd for $C_8H_5ClF_3NO$ [M], 223.0012. Found, 223.0004.

(*E*)-*O*-Trifluoromethyl-2-naphthaldoxime (1p)

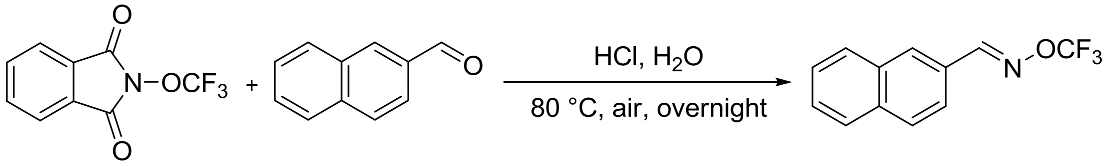

1p, 33%

In a round-bottom flask, PhthNOCF$_3$ (**S1**) (2-(trifluoromethoxy)isoindoline-1,3-dione) (1.00 g, 4.33 mmol, 1.00 equiv.), water (3.60 mL), HCl (0.720 mL ca. 6.00 M aq., 4.33 mmol, 1.00 equiv.) and 2-naphthaldehyde (1.01 g, 6.50 mmol, 1.50 equiv.) were added. The mixture was stirred at 80 °C overnight. Afterwards, the reaction mixture was extracted with CH$_2$Cl$_2$ (20.0 mL×2). The combined organic layer was dried over anhydrous MgSO$_4$, filtered and concentrated. The residue was purified by silica gel chromatography, eluting with *n*-hexane to afford 345 mg (*E*)-*O*-trifluoromethyl-2-naphthaldoxime (**1p**) as a white solid (33% yield).

R$_f$ = 0.4 (*n*-hexane). NMR Spectroscopy: ^{1}H NMR (400 MHz, CDCl$_3$) δ 8.39 (s, 1H), 7.98 (s, 1H), 7.95 – 7.83 (m, 4H), 7.64 – 7.51 (m, 2H). ^{13}C NMR (101 MHz, CDCl$_3$) δ 155.8, 134.9, 132.9, 130.8, 129.0, 128.6, 128.0, 128.0, 127.0, 127.0, 122.8, 122.4 (q, J = 259.1 Hz). ^{19}F NMR (376 MHz, DMSO) δ -63.8 (s, 3F). Mass Spectrometry: HRMS-EI (m/z): Calcd for C$_{12}$H$_8$F$_3$NO [M], 239.0558. Found, 239.0553.

(*E*)-*O*-Trifluoromethyl-1-naphthaldoxime (1q)

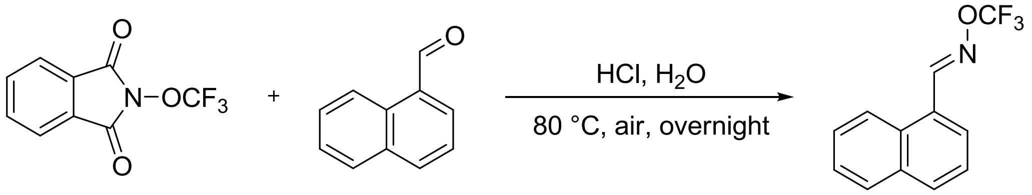

1q, 57%

In a round-bottom flask, PhthNOCF$_3$ (**S1**) (2-(trifluoromethoxy)isoindoline-1,3-dione) (1.00 g, 4.33 mmol, 1.00 equiv.), water (3.60 mL), HCl (0.720 mL ca. 6.00 M aq., 4.33 mmol, 1.00 equiv.) and 1-naphthaldehyde (0.880 mL, 6.50 mmol, 1.50 equiv.) were added. The mixture was stirred at 80 °C overnight. Afterwards, the reaction mixture was extracted with CH$_2$Cl$_2$ (20.0 mL×2). The combined organic layer was dried over anhydrous MgSO$_4$, filtered and concentrated. The residue was purified by silica gel chromatography, eluting with *n*-hexane to afford 592 mg (*E*)-*O*-trifluoromethyl-1-naphthaldoxime (**1q**) as a colorless liquid (57% yield).

R$_f$ = 0.4 (*n*-hexane). NMR Spectroscopy: ^{1}H NMR (400 MHz, CDCl$_3$) δ 8.70 (s, 1H), 8.44 (d, J = 8.4 Hz, 1H), 7.88 (d, J = 8.2 Hz, 1H), 7.82 (d, J = 8.0 Hz, 1H), 7.68 (d, J = 7.1 Hz, 1H), 7.55 (t, J = 7.2 Hz, 1H), 7.49 (t, J = 7.1 Hz, 1H), 7.41 (t, J = 7.7 Hz, 1H). ^{13}C NMR (101 MHz, CDCl$_3$) δ 156.0, 133.8, 132.7, 130.6, 130.0, 129.0, 128.0, 126.7, 125.4, 125.1, 124.5, 122.6 (q, J = 259.1 Hz). ^{19}F NMR (376 MHz, DMSO) δ -63.4 (s, 3F). Mass Spectrometry: HRMS-EI (m/z): Calcd for C$_{12}$H$_8$F$_3$NO [M], 239.0558. Found, 239.0553.

5-Chloropentyl 4-fluorobenzoate (S3-1)

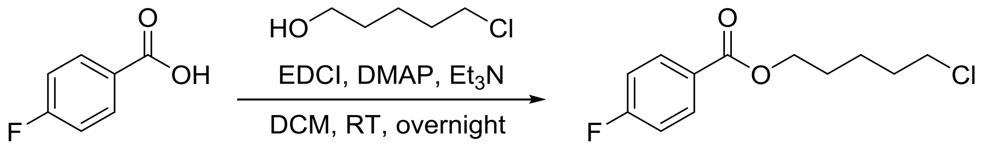

S3-1, 86%

To a solution of 4-fluorobenzoic acid (2.80 g, 20.0 mmol, 1.00 equiv.), DMAP (4-dimethylaminepyridine) (1.22 g, 10.0 mmol, 0.500 equiv.) and EDCI (*N*-(3-dimethylaminopropyl)-*N'*-ethylcarbodiimide hydrochloride) (5.76 g, 30.0 mmol, 1.50 equiv.) in dry CH_2Cl_2 (30.0 mL) at room temperature under N_2 were added distilled Et_3N (8.34 mL, 60.0 mmol, 3.00 equiv.) and 5-chloropentan-1-ol (4.62 mL, 60.0 mmol, 3.00 equiv.). The reaction was stirred at room temperature for 15 h before diluted with CH_2Cl_2 (20.0 mL) and quenched with H_2O (20.0 mL). The aqueous layer was separated and extracted 3 times with CH_2Cl_2 (50.0 mL). The combined organic layer was dried over anhydrous $MgSO_4$, filtered and concentrated in *vacuo*. The oily residue was subjected to silica gel chromatography using *n*-hexane/EtOAc 5:1 (v/v) as an eluent to afford 4.20 g 5-chloropentyl 4-fluorobenzoate(**S3-1**) as a light yellowish oil (86% yield).

R_f = 0.4 (*n*-hexane/EtOAc 9:1 (v/v)). NMR Spectroscopy: ^{1}H NMR (400 MHz, $CDCl_3$) δ 8.14 – 7.97 (m, 2H), 7.17 – 7.04 (m, 2H), 4.32 (t, *J* = 6.5 Hz, 2H), 3.57 (t, *J* = 6.5 Hz, 2H), 1.94 – 1.72 (m, 4H), 1.69 – 1.47 (m, 2H). ^{13}C NMR (101 MHz, $CDCl_3$) δ 165.9 (d, *J* = 253.7 Hz), 165.8, 132.2 (d, *J* = 9.3 Hz), 126.7, 115.6 (d, *J* = 22.0 Hz), 64.9, 44.9, 32.3, 28.2, 23.6. Mass Spectrometry: HRMS-EI (*m*/*z*): Calcd for $C_{14}H_{17}IO_2$ [M], 344.0273. Found, 344.0268.

5-Bromopentyl 4-fluorobenzoate (S3-2)

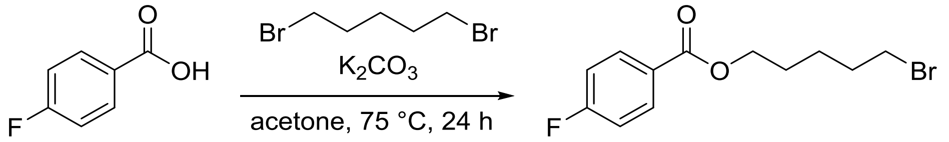

S3-2, 30%

To a solution of 4-fluorobenzoic acid (500 mg, 3.57 mmol, 1.00 equiv.) in acetone (10.0 mL) was added anhydrous K_2CO_3 (658 mg, 4.76 mmol, 1.33 equiv.) and 1,5-dibromopentane (0.640 mL, 4.76 mmol, 1.33 equiv.). The suspension was refluxed at 75 °C overnight. Afterwards the reaction was cooled to room temperature and concentrated to dryness. The solid was dissolved in EtOAc and washed with water and a saturated solution of sodium chloride. The organic phase was dried over anhydrous $MgSO_4$, filtered and concentrated in *vacuo*. The oily residue was subjected to silica gel chromatography, washed with *n*-hexane/CH_2Cl_2 9:1 (v/v) to remove excess 1,5-dibromopentane, then eluted with *n*-hexane /EtOAc 7:1 (v/v) to give 310 mg 5-bromopentyl 4-fluorobenzoate (**S3-2**) as a colorless oil (30% yield).

R_f = 0.7 (*n*-hexane/EtOAc 5:1 (v/v)). NMR Spectroscopy: ^{1}H NMR (400 MHz, $CDCl_3$) δ 8.19 – 7.91 (m, 2H), 7.19 – 7.01 (m, 2H), 4.33 (t, *J* = 6.5 Hz, 2H), 3.44 (t, *J* = 6.7 Hz, 2H), 2.04 –

1.88 (m, 2H), 1.88 – 1.74 (m, 2H), 1.71 – 1.57 (m, 2H). ^{13}C NMR (101 MHz, $CDCl_3$) δ 165.7 (d, J = 253.7 Hz), 165.6, 132.1 (d, J = 9.3 Hz), 126.6, 115.5 (d, J = 22.0 Hz), 64.8, 33.4, 32.3, 27.9, 24.7. Mass Spectrometry: HRMS-EI (m/z): Calcd for $C_{12}H_{14}FO_2$ $[M-Br]^+$, 209.0972. Found, 209.0973.

5-Iodopentyl (1,1'-biphenyl)-4-carboxylate (S13)

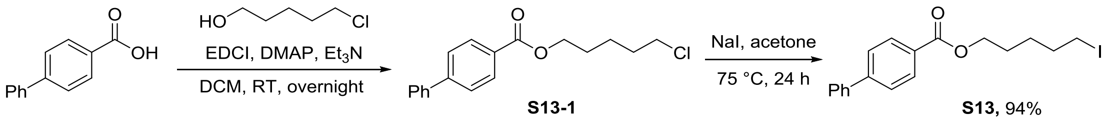

To a solution of (1.1'-biphenyl)-4-carboxylic acid (595 mg, 3.00 mmol, 1.00 equiv.), DMAP (4-dimethylaminepyridine) (183 mg, 1.50 mmol, 0.50 equiv.) and EDCI (N-(3-dimethylaminopropyl)-N'-ethylcarbodiimide hydrochloride) (864 mg, 4.50 mmol, 1.50 equiv.) in dry CH_2Cl_2 (5.0 mL) at room temperature under N_2 were added distilled Et_3N (1.25 mL, 9.00 mmol, 3.00 equiv.) and 5-chloropentan-1-ol (0.690 mL, 9.00 mmol, 3.00 equiv.). The reaction was stirred at room temperature for 22 h before diluted with CH_2Cl_2 (5.0 mL) and quenched with H_2O (5.0 mL). The aqueous layer was separated and extracted 2 times with CH_2Cl_2 (10.0 mL). The combined organic layer was dried over anhydrous $MgSO_4$, filtered and concentrated in *vacuo*. The residue was dissolved with CH_2Cl_2 (2.0 mL), passed through a pad of silica gel, washed with n-hexane/EtOAc 9:1 (v/v), the filtrate was concentrated in *vacuo* and the crude product 5-chloropentyl (1.1'-biphenyl)-4-carboxylate was used for next step directly.

The crude 5-chloropentyl (1.1'-biphenyl)-4-carboxylate (756 mg, 2.50 mmol, 1.00 equiv.) was dissolved with acetone (4.0 mL), sodium iodide (1.12 g, 7.49 mmol, 3.00 equiv.) was added at room temperature. Subsequently, the reaction flask was wrapped in aluminum foil and refluxed at 75 °C for 24 h. Afterwards the solvent was removed in reduced pressure. The solid was dissolved in EtOAc and washed successively with water 2 times. The organic phase was dried over anhydrous $MgSO_4$, filtered and concentrated in *vacuo*. The residue was purified by chromatography on silica gel, eluting with n-hexane /EtOAc 19:1 (v/v) to afford 928 mg 5-iodopentyl (1.1'-biphenyl)-4-carboxylate (**S13**) as a light yellowish oil (94% yield).

R_f = 0.6 (n-hexane/EtOAc 6:1 (v/v)). NMR Spectroscopy: ^{1}H NMR (400 MHz, $CDCl_3$) δ 8.17 – 8.07 (m, 2H), 7.72 – 7.60 (m, 4H), 7.52 – 7.44 (m, 2H), 7.44 – 7.36 (m, 1H), 4.36 (t, J = 6.5 Hz, 2H), 3.23 (t, J = 6.9 Hz, 2H), 1.98 – 1.87 (m, 2H), 1.87 – 1.77 (m, 2H), 1.66 – 1.54 (m, 2H). ^{13}C NMR (101 MHz, $CDCl_3$) δ 166.5, 145.6, 140.0, 130.1, 129.1, 129.0, 128.2, 127.3, 127.1, 64.7, 33.0, 27.7, 27.1, 6.6. Mass Spectrometry: HRMS-ESI (m/z): Calcd for $C_{18}H_{19}INaO_2$ $[M+Na]^+$, 417.0322. Found, 417.0325.

5-Iodopentyl 4-formylbenzoate(S14)

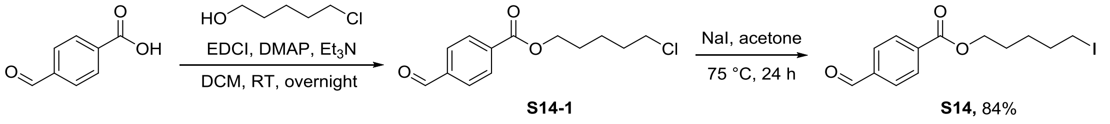

To a solution of 4-formylbenzoic acid (1.13 g, 7.50 mmol, 1.00 equiv.), DMAP

(4-dimethylaminepyridine) (458 mg, 3.75 mmol, 0.500 equiv.) and EDCI (*N*-(3-dimethylaminopropyl)-*N'*-ethylcarbodiimide hydrochloride) (2.16 g, 11.3 mmol,1.50 equiv.) in dry CH_2Cl_2 (12.0 mL) at room temperature under N_2 were added distilled Et_3N (3.12 mL, 22.5 mmol, 3.00 equiv.) and 5-chloropentan-1-ol (1.73 mL, 22.5 mmol, 3.00 equiv.). The reaction was stirred at room temperature for 24 h before diluted with CH_2Cl_2 (10.0 mL) and quenched with H_2O (10.0 mL). The aqueous layer was separated and extracted 3 times with CH_2Cl_2 (20.0 mL). The combined organic layer was dried over anhydrous $MgSO_4$, filtered and concentrated in *vacuo*. The residue was dissolved with CH_2Cl_2 (2.0 mL), passed through a pad of silica gel, washed with *n*-hexane /EtOAc 8:1 (v/v), the filtrate was concentrated in *vacuo* and the crude product 5-chloropentyl 4-formylbenzoate was used for next step directly.

The crude 5-chloropentyl 4-formylbenzoate (1.24 g, 4.87 mmol, 1.00 equiv.) was dissolved with acetone (8.0 mL), sodium iodide (2.19 g, 14.6 mmol, 3.00 equiv.) was added at room temperature. Subsequently, the reaction flask was wrapped in aluminum foil and refluxed at 75 °C for 24 h. Afterwards the solvent was removed in reduced pressure. The solid was dissolved in EtOAc and washed successively with water 2 times and a saturated solution of sodium chloride. The organic phase was dried over anhydrous $MgSO_4$, filtered and concentrated in *vacuo*. The residue was purified by chromatography on silica gel, eluting with *n*-hexane/EtOAc 12:1 (v/v) to afford 1.42 g 5-iodopentyl 4-formylbenzoate (**S14**) as a yellowish oil (84% yield).

R_f = 0.4 (*n*-hexane /EtOAc 9:1 (v/v)). NMR Spectroscopy: ^1H NMR (400 MHz, $CDCl_3$) δ 10.06 (s, 1H), 8.15 (d, *J* = 8.1 Hz, 2H), 7.91 (d, *J* = 8.2 Hz, 2H), 4.32 (t, *J* = 6.5 Hz, 2H), 3.18 (t, *J* = 6.9 Hz, 2H), 1.92 – 1.72 (m, 4H), 1.60 – 1.48 (m, 2H). ^{13}C NMR (101 MHz, $CDCl_3$) δ 191.6, 165.5, 139.1, 135.2, 130.1, 129.5, 65.2, 32.9, 27.6, 27.0, 6.6. Mass Spectrometry: HRMS-EI (*m*/*z*): Calcd for $C_{13}H_{14}IO_3$ $[M-H]^-$, 344.9993. Found, 344.9986

5-Iodopentyl 4-ethynylbenzoate (S15)

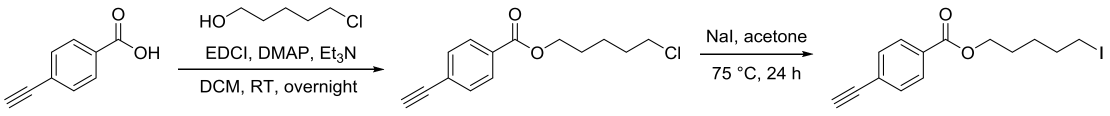

S15-1 S15, 71%

To a solution of 4-ethynylbenzoic acid (438 mg, 3.00 mmol, 1.00 equiv.), DMAP (4-dimethylaminepyridine) (183 mg, 1.50 mmol, 0.500 equiv.) and EDCI (*N*-(3-dimethylaminopropyl)-*N'*-ethylcarbodiimide hydrochloride) (864 mg, 4.50 mmol,1.50 equiv.) in dry CH_2Cl_2 (5.0 mL) at room temperature under N_2 were added distilled Et_3N (1.25 mL, 9.00 mmol, 3.00 equiv.) and 5-chloropentan-1-ol (0.69 mL, 9.00 mmol, 3.00 equiv.). The reaction was stirred at room temperature for 24 h before diluted with CH_2Cl_2 (5.0 mL) and quenched with H_2O (5.0 mL). The aqueous layer was separated and extracted 3 times with CH_2Cl_2 (10.0 mL). The combined organic layer was dried over anhydrous $MgSO_4$, filtered and concentrated in *vacuo*. The residue was dissolved with CH_2Cl_2 (2.0 mL), passed through a pad of silica gel, washed with *n*-hexane/EtOAc 9:1 (v/v), the filtrate was concentrated in *vacuo* and the crude product 5-chloropentyl 4-ethynylbenzoate was used for next step directly.

The crude 5-chloropentyl 4-ethynylbenzoate (307 mg, 1.22 mmol, 1.00 equiv.) was dissolved

with acetone (2.5 mL), sodium iodide (551 mg, 3.67 mmol, 3.00 equiv.) was added at room temperature. Subsequently, the reaction flask was wrapped in aluminum foil and refluxed at 75 °C for 24 h. Afterwards the solvent was removed in reduced pressure. The solid was dissolved in EtOAc and washed successively with water 2 times and a saturated solution of sodium chloride. The organic phase was dried over anhydrous $MgSO_4$, filtered and concentrated in *vacuo*. The residue was purified by chromatography on silica gel, eluting with *n*-hexane/EtOAc 15:1 (v/v) to afford 296 mg 5-iodopentyl 4-ethynylbenzoate (**S15**) as a white solid (71% yield).

R_f = 0.7 (*n*-hexane /EtOAc 4:1 (v/v)). NMR Spectroscopy: ^{1}H NMR (400 MHz, $CDCl_3$) δ 8.0 – 7.9 (m, 2H), 7.6 – 7.5 (m, 2H), 4.3 (t, J = 6.5 Hz, 2H), 3.3 – 3.2 (m, 3H), 1.9 – 1.9 (m, 2H), 1.8 – 1.7 (m, 2H), 1.6 – 1.5 (m, 2H). ^{13}C NMR (101 MHz, $CDCl_3$) δ 166.0, 132.2, 130.4, 129.5, 126.8, 82.9, 80.2, 65.0, 33.1, 27.7, 27.1, 6.7. Mass Spectrometry: HRMS-EI (*m*/*z*): Calcd for $C_{14}H_{15}O_2$ $[M-I]^+$, 215.1067. Found, 215.1065.

5-Chloropentyl 4-vinylbenzoate (S16-1)

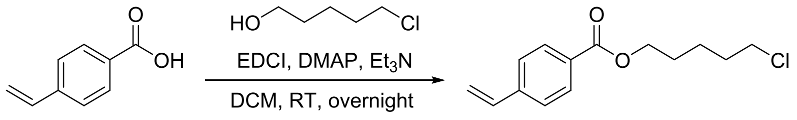

S16-1, 29%

To a solution of 4-vinylbenzoate acid (600 mg, 4.05 mmol, 1.00 equiv.), DMAP (4-dimethylaminepyridine) (247 mg, 2.02 mmol, 0.500 equiv.) and EDCI (*N*-(3-dimethylaminopropyl)-*N*'-ethylcarbodiimide hydrochloride) (1.16 g, 6.07 mmol,1.50 equiv.) in dry CH_2Cl_2 (7.5 mL) at room temperature under N_2 were added distilled Et_3N (1.82 mL, 12.1 mmol, 3.00 equiv.) and 5-chloropentan-1-ol (1.40 mL, 12.1 mmol, 3.00 equiv.). The reaction was stirred at room temperature for 16 h before diluted with CH_2Cl_2 (5.0 mL) and quenched with H_2O (5.0 mL). The aqueous layer was separated and extracted 2 times with CH_2Cl_2 (10.0 mL). The combined organic layer was dried over anhydrous $MgSO_4$, filtered and concentrated in *vacuo*. The residue was subjected to silica gel chromatography using *n*-hexane/EtOAc 30:1 (v/v) as an eluent to afford 300 mg pure 5-chloropentyl 4-vinylbenzoate (**S16-1**) as a light yellowish oil (29% yield).

R_f = 0.8 (*n*-hexane/EtOAc 9:1 (v/v)). NMR Spectroscopy: ^{1}H NMR (400 MHz, $CDCl_3$) δ 7.99 (d, J = 8.2 Hz, 2H), 7.46 (d, J = 8.1 Hz, 2H), 6.75 (dd, J = 17.6, 10.9 Hz, 1H), 5.86 (d, J = 17.6 Hz, 1H), 5.38 (d, J = 10.9 Hz, 1H), 4.33 (t, J = 6.5 Hz, 2H), 3.57 (t, J = 6.6 Hz, 2H), 1.94 – 1.74 (m, 4H), 1.68 – 1.58 (m, 2H). ^{13}C NMR (101 MHz, $CDCl_3$) δ 166.4, 142.0, 136.1, 129.9, 129.5, 126.2, 116.6, 64.7, 44.9, 32.3, 28.1, 23.6. Mass Spectrometry: HRMS-EI (*m*/*z*): Calcd for $C_{14}H_{17}ClO_2$ [M], 252.0917. Found, 252.0913.

5-Bromopentyl 4-vinylbenzoate(S16-2)

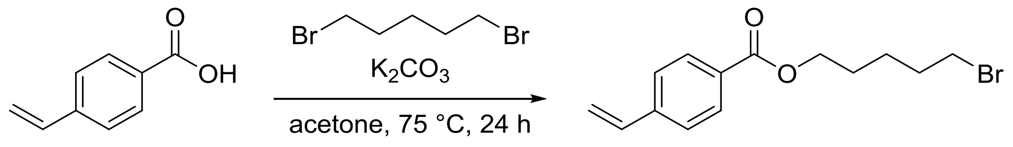

S16-2, 33%

To a solution of 4-vinylbenzoic acid (400 mg, 2.70 mmol, 1.00 equiv.) in acetone (8.0 mL) was added anhydrous K_2CO_3 (498 mg, 3.60 mmol, 1.33 equiv.) and 1,5-dibromopentane (0.49 0mL, 3.60 mmol, 1.33 equiv.). The suspension was refluxed at 75 °C overnight. Afterwards the reaction was cooled to room temperature and concentrated to dryness. The solid was dissolved in EtOAc and washed with water 2 times and a saturated solution of sodium chloride. The organic phase was dried over anhydrous $MgSO_4$, filtered and concentrated in *vacuo*. The oily residue was subjected to silica gel chromatography using *n*-hexane /EtOAc 10:1 (v/v) as an eluent to give 268 mg 5-bromopentyl 4-vinylbenzoate (**S16-2**) as a colorless oil (33% yield).

R_f = 0.4 (*n*-hexane /EtOAc 9:1 (v/v)). NMR Spectroscopy: ^{1}H NMR (400 MHz, $CDCl_3$) δ 8.00 (d, *J* = 8.0 Hz, 2H), 7.47 (d, *J* = 8.0 Hz, 2H), 6.76 (dd, *J* = 17.5, 10.9 Hz, 1H), 5.87 (d, *J* = 17.6 Hz, 1H), 5.39 (d, *J* = 10.9 Hz, 1H), 4.33 (t, *J* = 6.5 Hz, 2H), 3.44 (t, *J* = 6.7 Hz, 2H), 2.02 – 1.87 (m, 2H), 1.87 – 1.73 (m, 2H), 1.62 (p, *J* = 7.6, 7.1 Hz, 2H). ^{13}C NMR (101 MHz, $CDCl_3$) δ 166.5, 142.0, 136.1, 130.0, 129.5, 126.2, 116.6, 64.7, 33.6, 32.4, 28.0, 24.8. Mass Spectrometry: HRMS-EI (*m*/*z*): Calcd for $C_{14}H_{17}BrO_2$ [M], 296.0412. Found, 296.0409.

5-Iodopentyl 4-vinylbenzoate (S16)

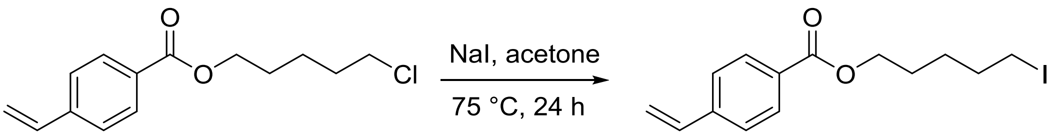

S16-1 **S16,** 69%

5-chloropentyl 4-vinylbenzoate (**S16-1)** (150 mg, 0.595 mmol, 1.00 equiv.) was dissolved with acetone (2.0 mL), sodium iodide (268 mg, 1.78 mmol, 3.00 equiv.) was added at room temperature. Subsequently, the reaction flask was wrapped in aluminum foil and refluxed at 75 °C for 24 h. Afterwards the solvent was removed in reduced pressure. The solid was dissolved in EtOAc and washed successively with water 2 times. The organic phase was dried over anhydrous $MgSO_4$, filtered and concentrated in *vacuo*. The residue was purified by preparative TLC with *n*-hexane/EtOAc 25:1 (v/v) to afford 142 mg 5-iodopentyl 4-vinylbenzoate (**S16)** as a light yellowish oil (69% yield).

R_f = 0.5 (*n*-hexane/EtOAc 4:1 (v/v)). NMR Spectroscopy: ^{1}H NMR (400 MHz, $CDCl_3$) δ 8.05 – 7.95 (m, 2H), 7.51 – 7.43 (m, 2H), 6.76 (dd, *J* = 17.6, 10.9 Hz, 1H), 5.87 (d, *J* = 17.6 Hz, 1H), 5.39 (d, *J* = 10.9 Hz, 1H), 4.33 (t, *J* = 6.5 Hz, 2H), 3.22 (t, *J* = 6.9 Hz, 2H), 1.96 – 1.86 (m, 2H), 1.86 – 1.76 (m, 2H), 1.64 – 1.57 (m, 2H). ^{13}C NMR (101 MHz, $CDCl_3$) δ 166.4, 141.9, 136.0, 129.9, 129.4, 126.1, 116.5, 64.6, 33.0, 27.7, 27.1, 6.5. Mass Spectrometry:

HRMS-EI (*m/z*): Calcd for $C_{14}H_{17}IO_2$ [M], 344.0273. Found, 344.0268.

5-Iodopentyl picolinate (S17)

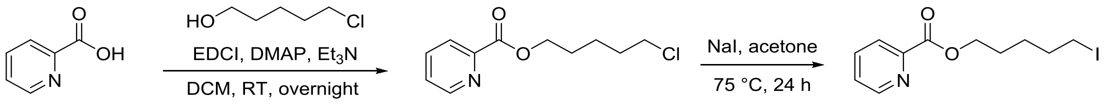

S17-1 **S17**, 46%

To a solution of picolinic acid (932 mg, 7.50 mmol, 1.00 equiv.), DMAP (4-dimethylaminepyridine) (458 mg, 3.75 mmol, 0.500 equiv.) and EDCI (*N*-(3-dimethylaminopropyl)-*N'*-ethylcarbodiimide hydrochloride) (2.16 g, 11.3 mmol, 1.50 equiv.) in dry CH_2Cl_2 (15.0 mL) at room temperature under N_2 were added distilled Et_3N (3.12 mL, 22.5 mmol, 3.00 equiv.) and 5-chloropentan-1-ol (1.73 mL, 22.5 mmol, 3.00 equiv.). The reaction was stirred at room temperature for 22 h before diluted with CH_2Cl_2 (7.5 mL) and quenched with H_2O (7.5 mL). The aqueous layer was separated and extracted 3 times with CH_2Cl_2 (20.0 mL). The residue was dissolved with CH_2Cl_2 (2.0 mL), passed through a pad of silica gel, washed with *n*-hexane/EtOAc 6:1 (v/v), the filtrate was concentrated in *vacuo* and the crude product 5-chloropentyl picolinate was used for next step directly.

The crude 5-chloropentyl picolinate (1.45 g, 6.35 mmol, 1.00 equiv.) was dissolved with acetone (10.0 mL), sodium iodide (2.86 g, 19.0 mmol, 3.00 equiv.) was added at room temperature. Subsequently, the reaction flask was wrapped in aluminum foil and refluxed at 75 °C for 24 h. Afterwards the solvent was removed in reduced pressure. The solid was dissolved in EtOAc (50.0 mL) and washed successively with water (30.0 mL) 2 times and a saturated solution of sodium chloride (30.0 mL). The organic phase was dried over sodium sulfate, filtered and concentrated in *vacuo*. The residue was purified by chromatography on silica gel, eluting with *n*-hexane/EtOAc 4:1 (v/v) to afford 732 mg 5-iodopentyl picolinate (**S17**) as a yellowish oil (46% yield).

R_f = 0.5 (*n*-hexane/EtOAc 1:1 (v/v)). NMR Spectroscopy: ^{1}H NMR (400 MHz, $CDCl_3$) δ 8.84 – 8.67 (m, 1H), 8.21 – 8.05 (m, 1H), 7.91 – 7.75 (m, 1H), 7.53 – 7.36 (m, 1H), 4.42 (t, *J* = 6.7 Hz, 2H), 3.20 (t, *J* = 6.9 Hz, 2H), 1.96 – 1.79 (m, 4H), 1.63 – 1.48 (m, 2H). ^{13}C NMR (101 MHz, $CDCl_3$) δ 165.4, 150.0, 148.3, 137.2, 127.0, 125.3, 65.7, 33.1, 27.8, 27.0, 6.5. Mass Spectrometry: HRMS-ESI (m/z): Calcd for $C_{11}H_{14}INNaO_2$ $[M+Na]^+$, 341.9961. Found, 341.9966.

1-Methyl-3-(3-iodopropyl)-1H-indole (S23)

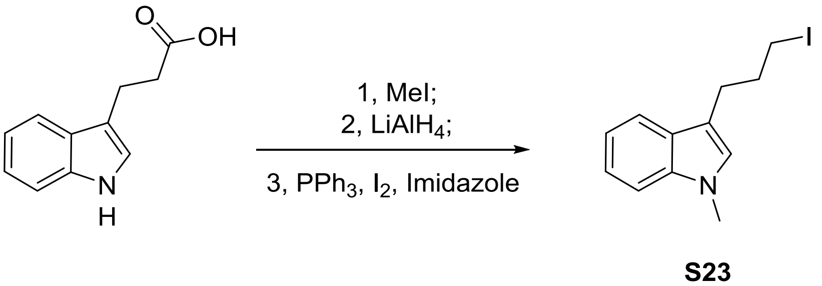

S23

To a solution of 3-indolepropionic acid (0.500 g, 2.64 mmol, 1.00 equiv.) in anhydrous DMF (5.00 mL) at 0 °C was added NaH (0.360 g, 7.93 mmol, 3.00 equiv.). After the addition, the mixture was allowed to stir for 10 mins and MeI (1.21 g, 7.93 mmol, 3.00 equiv.) was added. The reaction mixture was allowed to stir at room temperature for 1 h before the addition of 10.0 mL of ice water and 10.0 ml of EtOAc. After extraction with EtOAc (10.0 mL×2), the organic phase was dried over $MgSO_4$, filtered and concentrated under reduced pressure to give a yellow oil (0.610 g). The crude product was used in the next step without further purification.

To a flask containing a stirring solution of the crude product (0.610 mg, 2.90 mmol, 1.00 equiv.) in dry THF (5.00 mL) was added $LiAlH_4$ (0.380 g, 10.0 mmol, 3.50 equiv.) at 0 °C. After 1 h, the reaction was quenched with 10.0 ml ice water, the reaction mixture was extracted with EtOAc (10.0 mL×2), the organic phase was dried over $MgSO_4$, filtered and concentrated under reduced pressure to give a brown oil (0.410 g), The crude product was used in the next step without further purification.

In a round bottom flask, the brown crude product (0.210 g, 0.980 mmol, 1.00 equiv.), Ph_3P (0.310 g, 1.18 mmol, 1.20 equiv.) and imidazole (86.0 mg, 1.18 mmol, 1.20 equiv.) were dissolved in THF (5.00 mL), and the reaction was stirred for 10 mins at room temperature before the addition of iodine (0.310 g, 1.18 mmol, 1.20 equiv.). The reaction was stirred at room temperature for 1 h, then filtered and concentrated under reduced pressure. The residue was purified by preparative TLC, eluting with *n*-hexane/EtOAc 20:1 (v/v) to afford 120 mg 1-Methyl-3-(3-iodopropyl)-1H-indole (**S23**) as a clear oil (42% yield).

R_f = 0.2 (*n*-hexane/DCM 100:1 (v/v)). NMR Spectroscopy: ^{1}H NMR (400 MHz, $CDCl_3$) δ 7.52 – 7.46 (m, 1H), 7.21 – 7.08 (m, 2H), 7.05 – 6.97 (m, 1H), 6.75 (s, 1H), 3.60 (s, 3H), 3.09 (t, J = 6.8 Hz, 2H), 2.76 (t, J = 7.0 Hz, 2H), 2.13 – 1.99 (m, 2H). ^{13}C NMR (101 MHz, $CDCl_3$) δ 137.1, 127.7, 126.7, 121.6, 119.0, 118.8, 112.9, 109.3, 33.8, 32.7, 25.6, 7.7. Mass Spectrometry: Mass Spectrometry: HRMS-ESI (*m/z*): Calcd for $C_{12}H_{15}IN$ $[M+H]^+$, 300.0244. Found, 300.0240.

N-Boc-3-(2-iodoethyl)azetidine (S25)

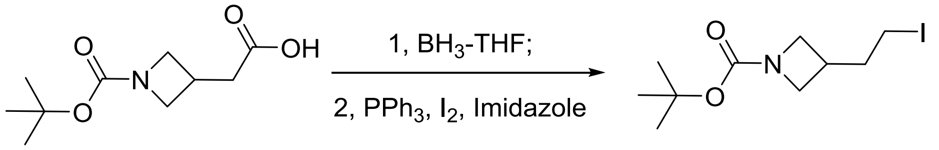

S25

A solution of 2-(1-(tert-butoxycarbonyl)azetidin-3-yl)acetic acid (300 mg, 1.39 mmol, 1.00 equiv.) in THF (5.00 mL) was cooled to 0 °C and treated with a borane 1M in THF (2.78 mL, 2.78 mmol, 2.00 equiv.), then slowly warmed to RT. After stirring for 18 h, the reaction mixture was quenched by drop-wise addition of 2 N NaOH (4.0 mL) then extracted with DCM (30.0 mL×4). The organic layer was combined, dried ($MgSO_4$), and concentrated. The residue was dissolved with dry THF (5.00 mL), to which was added sequentially PPh_3 (474 mg, 1.81 mmol, 1.30 equiv.), imidazole (132 mg, 1.95 mmol, 1.40 equiv.) and iodine (459 mg, 1.81 mmol, 1.30 equiv.) in three portions. The reaction was stirred 1 h at room temperature.

The reaction was quenched by the addition of a saturated solution of sodium thiosulfate and extracted three times with EtOAc (20.0 mL). The combined organic layer was washed with brine, dried over anhydrous $MgSO_4$, filtered and concentrated *in vacuo*. The residue was purified by chromatography on silica gel, eluting with *n*-hexane/EtOAc 20:1 (v/v) to afford *N*-Boc-3-(2-iodoethyl)azetidine (**S25**) as a colorless liquid (68% yield).

R_f = 0.1 (*n*-hexane/EtOAc 20:1 (v/v)). NMR Spectroscopy: ^{1}H NMR (400 MHz, $CDCl_3$) δ 3.97 (t, *J* = 8.4 Hz, 2H), 3.51 (dd, *J* = 8.6, 5.5 Hz, 2H), 3.05 (t, *J* = 7.0 Hz, 2H), 2.66 – 2.47 (m, 1H), 2.08 (q, *J* = 7.1 Hz, 2H), 1.37 (s, 9H). ^{13}C NMR (101 MHz, $CDCl_3$) δ 156.2, 79.3, 37.8, 29.8, 28.4, 2.4. Mass Spectrometry: HRMS-EI (*m/z*): Calcd for $C_{10}H_{18}INO_2$ [M], 311.0382. Found, 311.0379.

11-Bromoundecyl 5-chloropentanoate (S29-1)

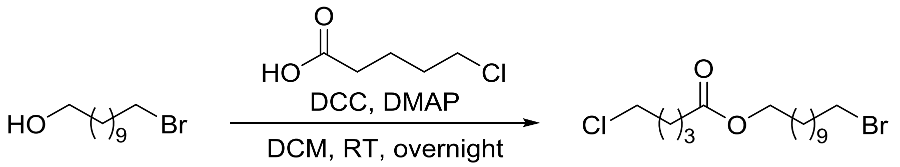

S29-1, 78%

To a solution of 1-Bromo-1-undecanol (168 mg, 0.669 mmol, 1.00 equiv.), DMAP (4-dimethylaminepyridine) (24.5 mg, 0.201 mmol, 0.300 equiv.) and DCC (dicyclohexylcarbodiimide) (332 mg, 1.61 mmol,2.40 equiv.) in dry CH_2Cl_2 (2.00 mL) at room temperature under N_2 were added 5-Chlorovaleric acid (110 mg, 0.803 mmol, 1.20 equiv.). The reaction was stirred at room temperature for overnight before diluted with CH_2Cl_2 (5.0 mL) and quenched with H_2O (5.0 mL). The aqueous layer was separated and extracted 2 times with CH_2Cl_2 (10.0 mL). The combined organic layer was dried over anhydrous $MgSO_4$, filtered and concentrated in *vacuo*. The residue was subjected to silica gel chromatography using *n*-hexane/EtOAc 30:1 (v/v) as an eluent to afford 194 mg 11-bromoundecyl 5-chloropentanoate (**S29-1**) as a white solid (78% yield).

R_f = 0.2 (*n*-hexane/EtOAc 30:1 (v/v)). NMR Spectroscopy: ^{1}H NMR (400 MHz, $CDCl_3$) δ 4.06 (t, *J* = 6.6 Hz, 2H), 3.55 (t, *J* = 5.7 Hz, 2H), 3.41 (t, *J* = 6.7 Hz, 2H), 2.34 (t, *J* = 6.5 Hz, 2H), 1.84 (dd, *J* = 16.6, 9.3 Hz, 6H), 1.67 – 1.57 (m, 2H), 1.28 (s, 14H). ^{13}C NMR (101 MHz, $CDCl_3$) δ 173.4, 64.8, 44.6, 34.2, 33.6, 33.0, 32.0, 29.6, 29.6, 29.5, 29.4, 28.9, 28.8, 28.3, 26.1, 22.4. Mass Spectrometry: HRMS-EI (*m/z*): Calcd for $C_{16}H_{31}BrClO_2$ $[M+H]^+$, 369.1190. Found, 369.1189.

11-Iodoundecyl 5-chloropentanoate (S29)

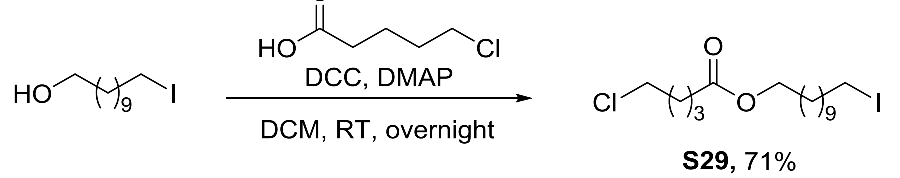

S29, 71%

To a solution of 1-iodoundecyl-1-undecanol (200 mg, 0.670 mmol, 1.00 equiv.), DMAP (4-dimethylaminepyridine) (24.6 mg, 0.201 mmol, 0.300 equiv.) and DCC

(dicyclohexylcarbodiimide) (332 mg, 1.61 mmol,2.40 equiv.) in dry CH_2Cl_2 (2.00 mL) at room temperature under N_2 were added 5-Chlorovaleric acid (110 mg, 0.805 mmol, 1.20 equiv.). The reaction was stirred at room temperature for overnight before diluted with CH_2Cl_2 (5.0 mL) and quenched with H_2O (5.0 mL). The aqueous layer was separated and extracted 2 times with CH_2Cl_2 (10.0 mL). The combined organic layer was dried over anhydrous $MgSO_4$, filtered and concentrated in *vacuo*. The residue was subjected to silica gel chromatography using *n*-hexane /EtOAc 30:1 (v/v) as an eluent to afford 198 mg 11-iodoundecyl 5-chloropentanoate (**S29**) as a white solid (71% yield).

R_f = 0.2 (*n*-hexane /EtOAc 30:1 (v/v)). NMR Spectroscopy: ^{1}H NMR (400 MHz, $CDCl_3$) δ 4.06 (t, *J* = 6.5 Hz, 2H), 3.55 (t, *J* = 5.6 Hz, 2H), 3.19 (t, *J* = 7.0 Hz, 2H), 2.34 (t, *J* = 6.4 Hz, 2H), 1.88 – 1.72 (m, 6H), 1.68 – 1.56 (m, 2H), 1.28 (m, 14H). ^{13}C NMR (101 MHz, $CDCl_3$) δ 173.4, 64.8, 44.6, 33.7, 33.6, 32.0, 30.6, 29.6, 29.6, 29.5, 29.4, 28.8, 28.7, 26.1, 22.4, 7.5. Mass Spectrometry: HRMS-EI (*m*/*z*): Calcd for $C_{16}H_{31}ClIO_2$ $[M+H]^+$, 417.1052. Found, 417.1050.

1-(5-Iodohexyl)-3,7-dihydro-3,7-dimethyl-1H-purine-2,6-dione (S34)

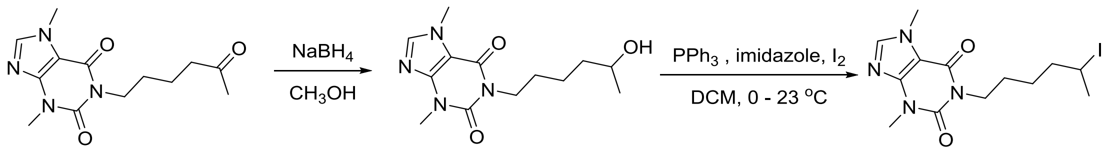

S34, 75%

To a solution of pentoxifylline (520 mg, 1.80 mmol, 1.00 equiv.) in dry CH_3OH (15.0 mL) at 0 °C under N_2 were added $NaBH_4$ (204 mg, 5.39 mmol, 3.00 equiv.). The reaction mixture was warmed to 23 °C and stirred overnight. When completed, the reaction was diluted with CH_2Cl_2 (10.0 mL) and quenched with H_2O (10.0 mL). The mixture was stirred for 15 min and extracted three times with CH_2Cl_2 (25.0 mL). The combined organic layer was dried over anhydrous $MgSO_4$, filtered and concentrated *in vacuo*. The residue was dissolved with dry CH_2Cl_2 (10.0 mL), to which was added sequentially PPh_3 (519 mg, 1.98 mmol, 1.10 equiv.), imidazole (147 mg, 2.16 mmol, 1.20 equiv.) and iodine (548 mg, 2.16 mmol, 1.20 equiv.) in three portions. The reaction was stirred overnight in the dark at room temperature. The reaction was quenched by the addition of a saturated solution of sodium thiosulfate and extracted three times with CH_2Cl_2 (20.0 mL). The combined organic layer was washed with brine, dried over anhydrous $MgSO_4$, filtered and concentrated *in vacuo*. The residue was purified by chromatography on silica gel, eluting with CH_2Cl_2/MeOH 10:1 (v/v) to afford 524 mg 1-(5-Iodohexyl)-3,7-dihydro-3,7-dimethyl-1H-purine-2,6-dione (**S34**) as a white solid (75% yield).

R_f = 0.2 (CH_2Cl_2/MeOH 10:1 (v/v)). NMR Spectroscopy: ^{1}H NMR (400 MHz, $CDCl_3$) δ 7.49 (s, 1H), 4.29 – 3.88 (m, 6H), 3.57 (s, 3H), 1.90 (dd, *J* = 12.2, 5.7 Hz, 4H), 1.78 – 1.36 (m, 5H). ^{13}C NMR (101 MHz, $CDCl_3$) δ 155.4, 151.6, 148.9, 141.5, 107.8, 42.5, 41.2, 33.7, 30.2, 29.8, 29.1, 27.2, 27.2. Mass Spectrometry: HRMS-ESI (*m*/*z*): Calcd for $C_{13}H_{20}IN_4O_2$ $[M+H]^+$, 391.0625. Found, 391.0628.

***N*,*N*-Dimethyl-4-(5-(4-(bromomethyl)phenyl)-3-(trifluoromethyl)-1*H*-pyrazol-1-yl)benze**

nesulfonamide (S40)

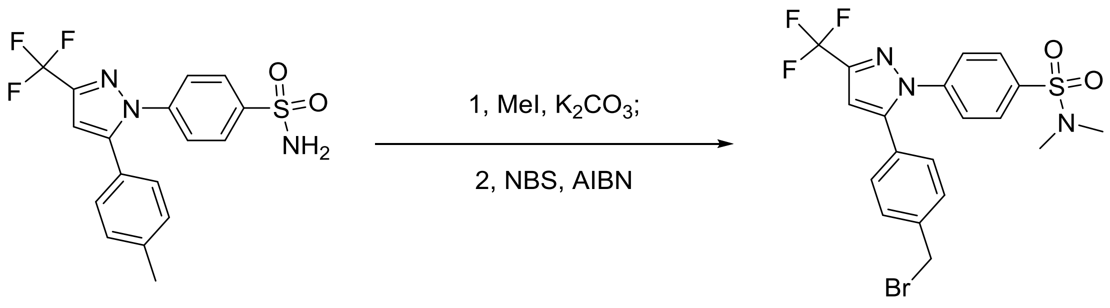

S40, 42%

To a solution of celecoxib (0.510 g, 1.31 mmol, 1.00 equiv.) and K_2CO_3 (0.970 g, 6.60 mmol, 5.00 equiv.) in anhydrous acetone (10.0 mL) at 0 °C was added MeI (0.960 g, 6.60 mmol, 5.00 equiv.). After the addition, the mixture was allowed to stir overnight at room temperature. The reaction mixture was concentrated *in vacuo* before the addition of 10.0 mL of water. After extraction with EtOAc (10.0 mL×2), the organic phase was dried over $MgSO_4$, filtered and concentrated under reduced pressure to give a yellow solid (0.540 g). The crude product of celecoxib derivative was used in the next step without further purification.

In a round bottom flask, the crude product of the last step (0.400 g, 0.980 mmol 1.00 equiv.), NBS (0.170 g, 0.980 mmol, 1.00 equiv.) and AIBN (34.0 mg, 0.200 mmol, 0.200 equiv.) were dissolved in CCl_4 (8.00 mL), and the reaction was stirred overnight at 60 °C. the reaction was cooled to room temperature before purified by preparative TLC, eluting with *n*-hexane/EtOAc 10:1 (v/v) to afford 210 mg celecoxib derivative (**S40**) as a white solid (42% yield).

R_f = 0.4 (*n*-hexane /EtOAc 4:1 (v/v)). NMR Spectroscopy: ^{1}H NMR (400 MHz, $CDCl_3$) 7.84 – 7.72 (m, 2H), 7.55 – 7.45 (m, 2H), 7.45 – 7.34 (m, 2H), 7.25 – 7.13 (m, 2H), 6.80 (s, 1H), 4.48 (s, 2H), 2.71 (s, 6H). ^{13}C NMR (101 MHz, $CDCl_3$) δ 144.5, 144.3 (q, J = 39.1 Hz), 142.4, 139.5, 135.5, 129.8, 129.3, 128.9, 128.6, 125.7, 121.0 (q, J = 269.3 Hz), 106.8, 37.9, 32.3. ^{19}F NMR (376 MHz, $CDCl_3$) -62.5 (s, 3F).Mass Spectrometry: HRMS-ESI (*m/z*): Calcd for $C_{19}H_{18}BrF_3N_3O_2S$ $[M+H]^+$, 488.0250. Found, 488.0255.

Mycophenolic acid iodination derivative (S41)

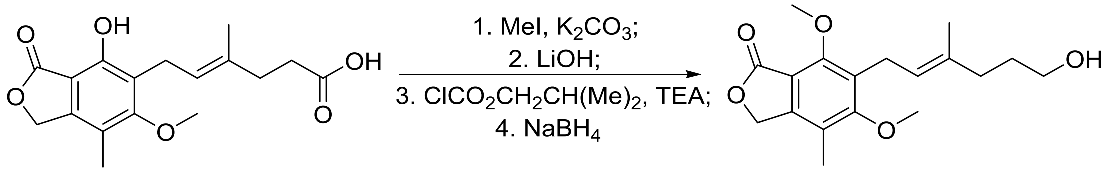

S41-1, 58%

To a solution of mycophenolic acid (500 mg, 1.56 mmol, 1.00 equiv.) and MeI (0.583 mL, 9.37 mmol, 6.00 equiv.) in acetone (10.0 mL) was added K_2CO_3 (1.51 g, 1.09 mmol, 7.00 equiv.). Subsequently, the reaction mixture was warmed to reflux for 5 h. Afterwards the reaction mixture was concentrated *in vacuo* and the residue was dissolved in EtOAc. The organic phase was washed with water, dried over sodium sulphate, filtered and concentrated

in *vacuo*. The residue and LiOH (224 mg, 9.36 mmol, 6.00 equiv.) were suspended in a mixture of H_2O and THF (1:4, 20.0 mL) and stirred at 30 °C for 12 h. The solution was acidified with aqueous HCl (1 M). The mixture was extracted with EtOAc (80.0 mL×3), the combined organic layer was washed with brine and dried ($MgSO_4$), and the solvents were evaporated.

The crude product of the last step, $ClCO_2CH_2CH(Me)_2$ (634 mg, 4.68 mmol, 3.00 equiv.), Et_3N (474 mg, 4.68 mmol, 3.00 equiv.) were dissolved in THF (10.0 mL), and the reaction was stirred at 0 °C. After 2 hours, the reaction was concentrated *in vacuo.* The intermediate was loaded into a round bottom flask, and dissolved in EtOH (10.0 mL). Sodium borohydride (354 mg, 9.36 mmol, 6.00 equiv.) was added and the reaction was stirred at 0 °C for 2 h. The reaction was quenched with water and saturated aqueous ammonium chloride, and the combined aqueous layers were extracted three times with EtOAc. The combined organic layer was washed with bring, dried over anhydrous $MgSO_4$, filtered, and concentrated *in vacuo*. The residue was purified by chromatography on silica gel, eluting with *n*-hexane/EtOAc 2:1 (v/v) to afford 289 mg Mycophenolic acid deoxidized derivative (**S41-1**) as a colorless liquid (58% yield).

R_f = 0.1 (*n*-hexane/EtOAc 2:1 (v/v)). NMR Spectroscopy: ^{1}H NMR (400 MHz, $CDCl_3$) δ 5.23 – 5.08 (m, 3H), 4.05 (s, 3H), 3.78 (s, 3H), 3.60 (t, *J* = 6.4 Hz, 2H), 3.41 (d, *J* = 6.8 Hz, 2H), 2.18 (s, 3H), 2.05 (t, *J* = 7.6 Hz, 2H), 1.81 (d, *J* = 1.3 Hz, 3H), 1.72 – 1.59 (m, 2H). ^{13}C NMR (101 MHz, $CDCl_3$) δ 169.1, 162.9, 156.9, 146.8, 135.4, 129.3, 123.3, 120.2, 112.7, 68.5, 63.0, 62.8, 61.1, 36.2, 30.9, 23.6, 16.3, 11.7. Mass Spectrometry: HRMS-ESI (*m*/*z*): Calcd for $C_{18}H_{25}O_5$ $[M+H]^+$, 321.1697. Found, 321.1694.

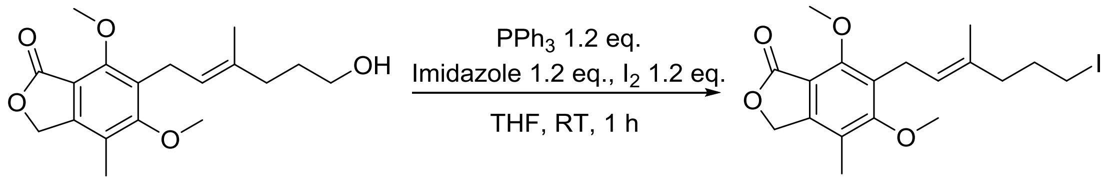

S41-1 **S41**, 82%

Mycophenolic acid deoxidized derivative (**S41-1**) (289 mg, 0.902 mmol, 1.00 equiv.) was dissolved with dry THF (5.00 mL), to which was added sequentially PPh_3 (284 mg, 1.08 mmol, 1.20 equiv.), imidazole (79.8 mg, 1.17 mmol, 1.30 equiv.) and iodine (275 mg, 1.08 mmol, 1.20 equiv.) in three portions. The reaction was stirred 1 h at room temperature. The reaction was quenched by the addition of a saturated solution of sodium thiosulfate and extracted three times with EtOAc (20.0 mL). The combined organic layer was washed with brine, dried over anhydrous $MgSO_4$, filtered and concentrated *in vacuo*. The residue was purified by chromatography on silica gel, eluting with *n*-hexane/EtOAc 10:1 (v/v) to afford 320 mg Mycophenolic acid iodination derivative (**S41**) as a colorless liquid (82% yield).

R_f = 0.2 (*n*-hexane/EtOAc 8:1 (v/v)). NMR Spectroscopy: ^{1}H NMR (400 MHz, $CDCl_3$) δ 5.23 – 5.01 (m, 3H), 4.02 (s, 3H), 3.76 (s, 3H), 3.38 (d, *J* = 6.9 Hz, 2H), 3.09 (t, *J* = 6.9 Hz, 2H), 2.16 (s, 3H), 2.04 (t, *J* = 7.3 Hz, 2H), 1.93 – 1.81 (m, 2H), 1.76 (d, *J* = 1.3 Hz, 3H). ^{13}C NMR (101 MHz, $CDCl_3$) δ 169.0, 162.8, 156.7, 146.8, 133.6, 129.0, 124.1, 120.1, 112.5, 68.4, 62.7, 61.1, 40.1, 31.6, 23.4, 16.1, 11.6, 6.7. Mass Spectrometry: HRMS-ESI (*m*/*z*): Calcd for

$C_{18}H_{24}IO_4$ $[M+H]^+$, 431.0714. Found, 431.0716.

(3aS,4S,6aR)-1,3-Dibenzyl-4-(5-iodopentyl)tetrahydro-1H-thieno[3,4-d]imidazol-2(3H)-one (S42)

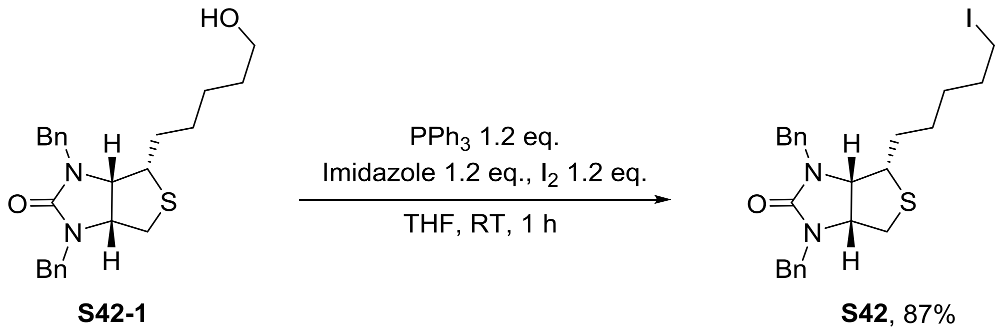

(3aS,4S,6aR)-1,3-dibenzyl-4-(5-hydroxypentyl)tetrahydro-1H-thieno[3,4-d]imidazol-2(3H)-one[2] (**S42-1**) (615 mg, 1.50 mmol, 1.00 equiv.) was dissolved with dry THF (10.0 mL), to which was added sequentially PPh_3 (471 mg, 1.80 mmol, 1.20 equiv.), imidazole (122 mg, 1.80 mmol, 1.20 equiv.) and iodine (456 mg, 1.80 mmol, 1.20 equiv.) in three portions. The reaction was stirred 1 h at room temperature. The reaction was quenched by the addition of a saturated solution of sodium thiosulfate and extracted three times with EtOAc (20.0 mL). The combined organic layer was washed with brine, dried over anhydrous $MgSO_4$, filtered and concentrated *in vacuo*. The residue was purified by chromatography on silica gel, eluting with *n*-hexane/EtOAc 5:1 (v/v) to afford 680 mg (3aS,4S,6aR)-1,3-dibenzyl-4-(5-iodopentyl) tetrahydro-1H-thieno[3,4-d]imidazol-2(3H)-one (**S42**) as a colorless liquid (87% yield).

R_f = 0.2 (*n*-hexane/EtOAc 5:1 (v/v)). NMR Spectroscopy: ^1H NMR (400 MHz, $CDCl_3$) δ 7.37 – 7.13 (m, 10H), 5.02 (d, *J* = 15.2 Hz, 1H), 4.70 (d, *J* = 15.2 Hz, 1H), 4.11 (d, *J* = 15.2 Hz, 1H), 3.99 – 3.87 (m, 2H), 3.82 (m, 1H), 3.14 (t, *J* = 6.9 Hz, 2H), 3.08 – 2.98 (m, 1H), 2.67 (d, *J* = 47.1 Hz, 2H), 1.85 – 1.67 (m, 2H), 1.66 – 1.14 (m, 6H). ^{13}C NMR (101 MHz, $CDCl_3$) δ 161.1, 137.1, 137.0, 128.8, 128.8, 128.4, 127.8, 127.7, 62.8, 61.3, 54.5, 48.1, 46.7, 34.8, 33.4, 30.3, 28.7, 28.1, 7.1. Mass Spectrometry: HRMS-ESI (*m*/*z*): Calcd for $C_{24}H_{29}IN_2NaOS$ $[M+Na]^+$, 543.0937. Found, 543.0942.

Ezetimibe derivative (S45)

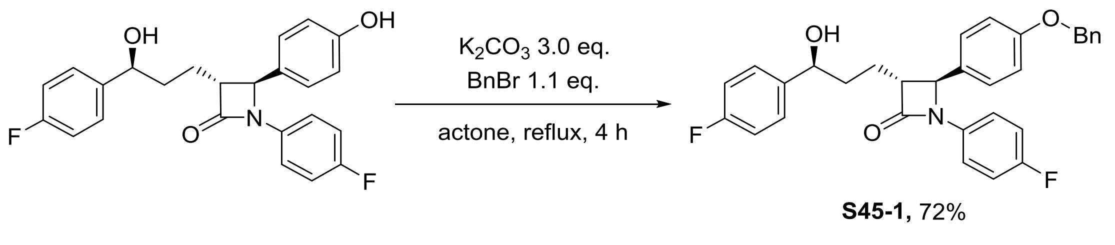

Potassium carbonate (1.01 g, 7.33 mmol, 3.00 equiv.) was suspended in dry acetone (20.0 mL), ezetimibe (1.00 g, 2.44 mmol, 1.00 equiv.) and BnBr (0.319 mL, 2.69 mmol, 1.10 equiv.) were added. Subsequently, the reaction mixture was refluxed for 4 h. Afterwards the cooled mixture was concentrated *in vacuo*. The residue was purified by chromatography on

silica gel, eluting with *n*-hexane/EtOAc 4:1 (v/v) to afford 879 mg ezetimibe derivative (**S45-1**) as a white solid (72% yield).

R_f = 0.3 (*n*-hexane/EtOAc 3:1 (v/v)). NMR Spectroscopy: ^{1}H NMR (400 MHz, $CDCl_3$) δ 7.74 – 7.46 (m, 11H), 7.33 – 7.15 (m, 6H), 5.33 (s, 2H), 5.02 – 4.93 (m, 1H), 4.85 (d, *J* = 2.2 Hz, 1H), 3.34 (dt, *J* = 7.6, 3.4 Hz, 1H), 2.78 (s, 1H), 2.32 – 2.07 (m, 4H). ^{13}C NMR (101 MHz, $CDCl_3$) δ 167.8, 162.3 (d, *J* = 245.7 Hz), 159.2, 159.1 (d, *J* = 243.3 Hz), 140.2 (d, *J* = 3.0 Hz), 136.8, 134.0, 129.7, 128.8, 128.2, 127.6, 127.5 (d, *J* = 7.9 Hz), 127.3, 118.5 (d, *J* = 7.7 Hz), 115.8 (d, *J* = 49.2 Hz), 115.7, 115.6 (d, *J* = 47.8 Hz), 73.2, 70.2, 61.2, 60.4, 36.7, 25.1. Mass Spectrometry: HRMS-ESI (*m/z*): Calcd for $C_{31}H_{27}F_2NNaO_3$ [M+Na]$^+$, 522.1851. Found, 522. 1855.

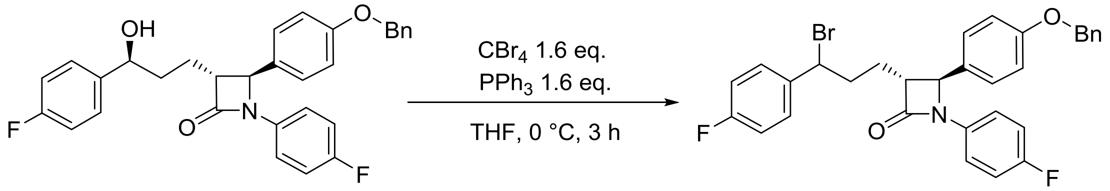

S45-1 **S45**, 71%

CBr_4 (149 mg, 0.448 mmol, 1.60 equiv.) was added to a 0 °C solution of the ezetimibe derivative (**S45-1**) (140 mg, 0.280 mmol, 1.00 equiv.) and PPh_3 (118 mg, 0.448 mmol, 1.60 equiv.) in THF (2.00 mL), then maintained the mixture at the same temperature for 1 h. When the reaction is complete (monitoring by TLC hexane/EtOAc 5:1), water was added, followed by an extraction with diethyl ether. The collected organic phase was dried over sodium sulphate, filtered and concentrated in *vacuo*. The residue was purified by preparative TLC, eluting with *n*-hexane /EtOAc 5:1 (v/v) to afford 111 mg ezetimibe derivative (**S45**) as a white solid (71% yield).

R_f = 0.3 (*n*-hexane/EtOAc 5:1 (v/v)). NMR Spectroscopy: ^{1}H NMR (400 MHz, $CDCl_3$) δ 7.47 – 7.19 (m, 11H), 7.06 – 6.89 (m, 6H), 5.07 (s, 2H), 4.96 – 4.88 (m, 1H), 4.63 – 4.55 (m, 1H), 3.14 – 3.03 (m, 1H), 2.60 – 1.78 (m, 4H). a ^{13}C NMR (101 MHz, $CDCl_3$) δ 166.9, 162.56 (d, *J* = 248.1 Hz), 159.25, 159.11 (d, *J* = 243.6 Hz), 137.6 (d, *J* = 5.5 Hz), 136.7, 133.9, 129.6, 129.1 (d, *J* = 8.2 Hz), 128.8, 128.3, 127.6, 127.3, 118.5 (d, *J* = 7.8 Hz), 116.0 (d, *J* = 22.7 Hz), 115.9 (d, *J* = 21.7 Hz), 115.7, 70.2, 61.1, 59.9, 53.8, 37.67, 27.4. b ^{13}C NMR (101 MHz, $CDCl_3$) δ 167.0, 162.6 (d, *J* = 248.1 Hz), 159.3, 159.1 (d, *J* = 243.6 Hz), 137.7 (d, *J* = 5.4 Hz), 136.7, 133.9, 129.5, 129.1 (d, *J* = 8.2 Hz), 128.8, 128.3, 127.6, 127.3, 118.5 (d, *J* = 7.8 Hz), 116.0 (d, *J* = 22.7 Hz), 115.9 (d, *J* = 21.8 Hz), 115.7, 70.2, 61.1, 59.9, 53.8, 37.7, 27.9. Mass Spectrometry: HRMS-ESI (*m/z*): Calcd for $C_{31}H_{26}F_2NO_2$ [M-Br]$^+$, 482.1926. Found, 482.1930.

Gibberellic acid derivative (S46)

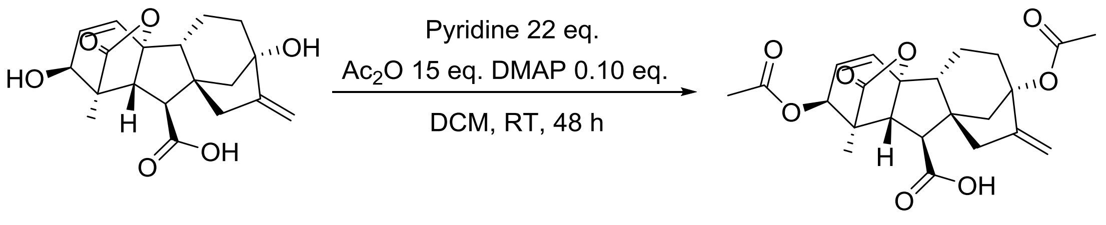

S46-1

To a solution of gibberellic acid (3.42 mg, 9.87 mmol, 1.0 equiv.), acetic anhydride (15.1.00 g, 148 mmol, 15.0 eq.) and DMAP (121 mg, 0.987 mmol, 0.1 equiv.) in anhydrous CH_2Cl_2 (100 mL) at room temperature was added pyridine (17.5 mL, 217 mmol, 22.0 equiv.). After the addition, the mixture was allowed to stir for 48 h at room temperature before the addition of 20 mL of water. After extraction with CH_2Cl_2 (100 mL×2), the organic phase was washed with 1 M HCl and dried over $MgSO_4$, filtered and concentrated under reduced pressure to give a white solid (3.70 g). The crude product of gibberellic acid derivative **S46-1** was used in the next step without further purification.[3]

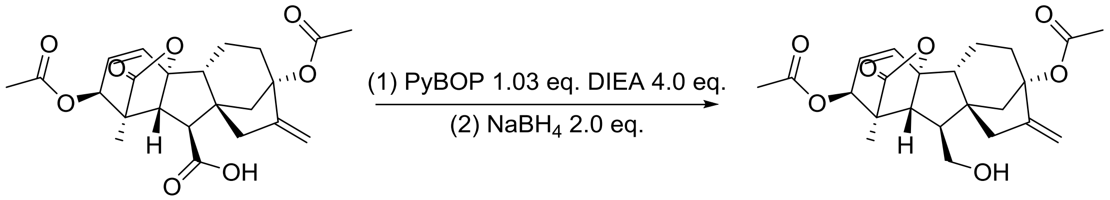

S46-1 **S46-2**, 36%

In a round bottom flask, gibberellic acid derivative **S46-1** (3.70 g, 8.60 mmol 1.00 equiv.), PyBOP (benzotriazol-1-yloxytripyrrolidinophosphonium hexafluorophosphate) (4.61 g, 8.85 mmol, 1.03 equiv.), and DIEA (*N*,*N*-diisopropylethylamine) (5.68 mL,34.4 mmol, 4.00 equiv.) were dissolved in CH_2Cl_2 (100 mL), and the reaction was stirred at room temperature. After one hour, the reaction was concentrated *in vacuo*. The intermediate (1.65 g, 3.56 mmol, 1.00 equiv.) was transferred into a round bottom flask under nitrogen, and dissolved in tetrahydrofuran (20.0 mL). Sodium borohydride (270 mg, 6.95 mmol, 2.00 equiv.) was added and the reaction was stirred at room temperature for 3.5 hours. The reaction was quenched with water and saturated aqueous sodium bicarbonate, and the combined aqueous layer was extracted three times with EtOAc. The combined organic layer was washed with brine, dried over anhydrous $MgSO_4$, filtered, and concentrated *in vacuo*. The residue was purified by chromatography on silica gel, eluting with CH_2Cl_2/CH_3OH 40:1 (v/v) to afford 650 mg gibberellic acid derivative **S46-2** as a white solid (36% yield).[4]

R_f = 0.3 (*n*-hexane /EtOAc 5:1 (v/v)). NMR Spectroscopy: ^1H NMR (400 MHz, $CDCl_3$) δ 6.36 (d, *J* = 9.3 Hz, 1H), 5.80 (dd, *J* = 9.3, 3.7 Hz, 1H), 5.28 (d, *J* = 3.7 Hz, 1H), 5.17 – 5.04 (m, 1H), 4.97 (s, 1H), 3.90 – 3.59 (m, 2H), 2.83 (d, *J* = 15.2 Hz, 1H), 2.64 (d, *J* = 10.5 Hz, 1H), 2.36 – 2.26 (m, 1H), 2.22 – 2.11 (m, 3H), 2.11 – 1.93 (m, 9H), 1.87 – 1.63 (m, 3H), 1.24 (s, 3H). ^{13}C NMR (101 MHz, $CDCl_3$) δ 177.7, 170.1, 153.9, 134.9, 128.8, 107.1, 90.6, 84.4, 71.1, 62.2, 52.5, 52.1, 51.0, 50.5, 46.5, 41.4, 41.3, 36.5, 22.2, 20.9, 16.9, 15.1. Mass Spectrometry: HRMS-ESI (*m*/*z*): Calcd for $C_{23}H_{28}NaO_7$ $[M+Na]^+$, 439.1727. Found,

439.1733.

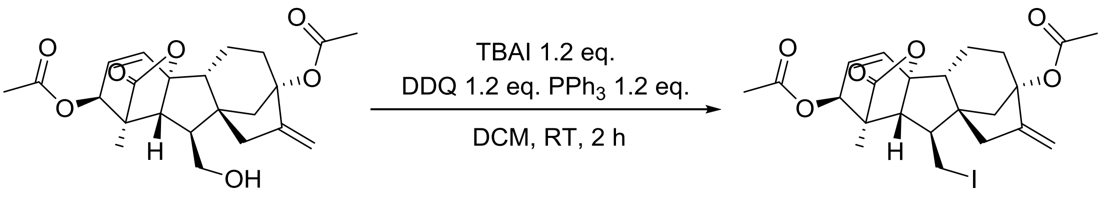

S46-2 **S46**, 60%

To a flask containing a stirring mixture of DDQ (167 mg, 0.738 mmol, 1.20 equiv.) and PPh_3 (193 mg, 0.738 mmol, 1.20 equiv.) in dry CH_2Cl_2 (5.00 mL) was added (n-butyl)$_4$NI (272 mg, 0.738 mmol, 1.20 equiv.) at room temperature. Gibberellic acid derivative **S46-2** (256 mg, 0.615 mmol, 1.00 equiv.) was then added to this mixture. After two hours, the reaction was concentrated *in vacuo*. The residue was purified by preparative TLC, eluting with *n*-hexane/EtOAc 2:1 (v/v) to afford 194 mg gibberellic acid derivative **S46** as a white solid (60% yield).

R_f = 0.8 (*n*-hexane /EtOAc 1:1 (v/v)). NMR Spectroscopy: ^{1}H NMR (400 MHz, $CDCl_3$) δ 6.37 (d, *J* = 9.3 Hz, 1H), 5.85 (dd, *J* = 9.3, 3.8 Hz, 1H), 5.32 (d, *J* = 3.8 Hz, 1H), 5.16 (d, *J* = 2.0 Hz, 1H), 5.04 (s, 1H), 3.32 (dd, *J* = 10.8, 2.7 Hz, 1H), 3.19 (dd, *J* = 10.7, 9.0 Hz, 1H), 2.83 (d, *J* = 14.7 Hz, 1H), 2.62 (d, *J* = 10.4 Hz, 1H), 2.45 (d, *J* = 10.6 Hz, 1H), 2.35 (ddd, *J* = 11.3, 8.4, 2.0 Hz, 1H), 2.27 – 2.10 (m, 6H), 2.06 – 1.94 (m, 4H), 1.90 – 1.65 (m, 3H), 1.31 (s, 3H). ^{13}C NMR (101 MHz, $CDCl_3$) δ 177.1, 170.0, 153.2, 134.7, 129.0, 107.8, 89.7, 84.0, 71.1, 58.4, 52.5, 51.7, 51.1, 45.3, 40.8, 40.6, 36.5, 22.2, 21.0, 16.9, 15.0, 4.8. Mass Spectrometry: HRMS-ESI (*m*/*z*): Calcd for $C_{23}H_{27}INaO_6$ [M+Na]$^+$, 549.0745. Found, 549.0750.

7-(5-Iodopentyl)tadalafil (S47)

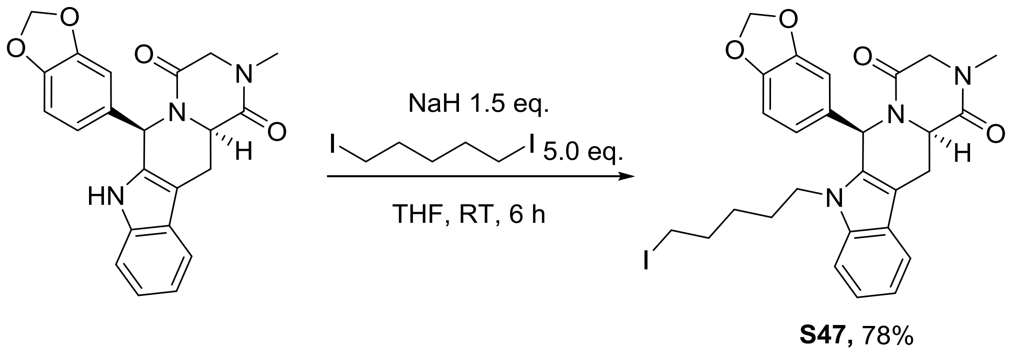

S47, 78%

To a solution of tadalafil (300 mg, 0.770 mmol, 1.00 equiv.) and 1,5-diiodopentane (0.573 mL, 3.85 mmol, 5.00 equiv.) in dry THF (5.00 mL) at 0 °C was added NaH (27.7 mg, 1.16 mmol, 1.50 equiv.). Subsequently, the reaction mixture was warmed to 25 °C for 6 h. Afterwards the cooled mixture was concentrated *in vacuo*. The residue was purified by chromatography on silica gel, eluting with CH_2Cl_2/CH_3OH 60:1 (v/v) to afford 350 mg 7-(5-iodopentyl)tadalafil (**S47**) as a white solid (78% yield).

R_f = 0.2 (CH_2Cl_2/CH_3OH 40:1 (v/v)). NMR Spectroscopy: ^{1}H NMR (400 MHz, $CDCl_3$) δ

7.54 (d, J = 7.8 Hz, 1H), 7.32 – 7.21 (m, 2H), 7.16 (t, J = 7.2 Hz, 1H), 7.01 (s, 1H), 6.81 (s, 1H), 6.76 – 6.64 (m, 2H), 5.96 (s, 2H), 4.36 (dd, J = 11.9, 4.1 Hz, 1H), 4.18 – 3.96 (m, 2H), 3.94 – 3.62 (m, 2H), 3.56 (dd, J = 15.5, 4.3 Hz, 1H), 3.13 – 2.90 (m, 6H), 1.69 (dt, J = 15.9, 8.2 Hz, 3H), 1.45 (dt, J = 13.5, 7.4 Hz, 1H), 1.29 (dd, J = 20.8, 6.9 Hz, 2H). ^{13}C NMR (101 MHz, $CDCl_3$) δ 165.7, 161.5, 148.4, 148.3, 136.8, 131.7, 130.9, 126.3, 122.6, 122.4, 119.8, 118.7, 109.6, 109.1, 108.5, 107.8, 101.6, 52.1, 51.6, 51.1, 43.7, 33.5, 33.0, 28.8, 28.0, 27.7, 6.1. Mass Spectrometry: HRMS-ESI (*m*/*z*): Calcd for $C_{27}H_{28}IN_3NaO_4$ $[M+Na]^+$, 608.1017. Found, 608.1020.

5-Iodovaleric acid acetylcyclosporin A ester (S48)

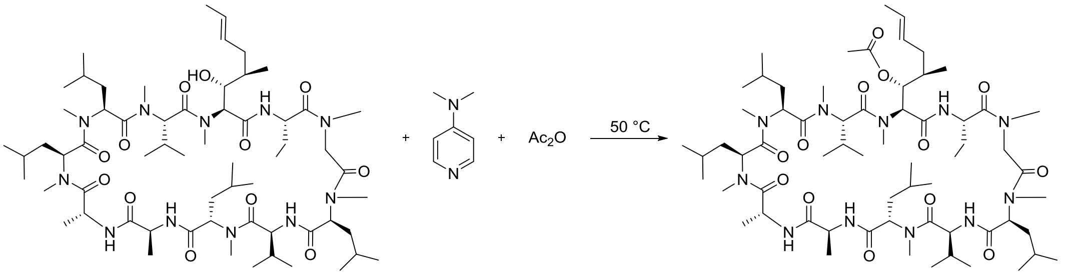

S48-1

A mixture of cyclosporin A (1.20 g, 0.998 mmol, 1.00 equiv.), DMAP (0.244 g, 2.00 mmol, 2.00 equiv.) and excess Ac_2O (10.0 mL) were stirred under N_2 at 50 °C for 6 hours. After this time, the mixture was poured slowly into sat. aq. $NaHCO_3$ (20.0 mL) and stirred for 1 h (Caution: vigorous emission of CO_2 when the reaction was quenched). The organic layer was separated and the aqueous layer was extracted with CH_2Cl_2 (30.0 mL×2). The combined organic layer was washed with aq 1 M HCl (500 mL×2), sat. aq. $NaHCO_3$ (20.0 mL), and brine (20.0 mL). The organic layer was then dried ($MgSO_4$), filtered, and concentrated under reduced pressure to give crude product acetylcyclosporin A (**S48-1**). The crude was used without further purification.

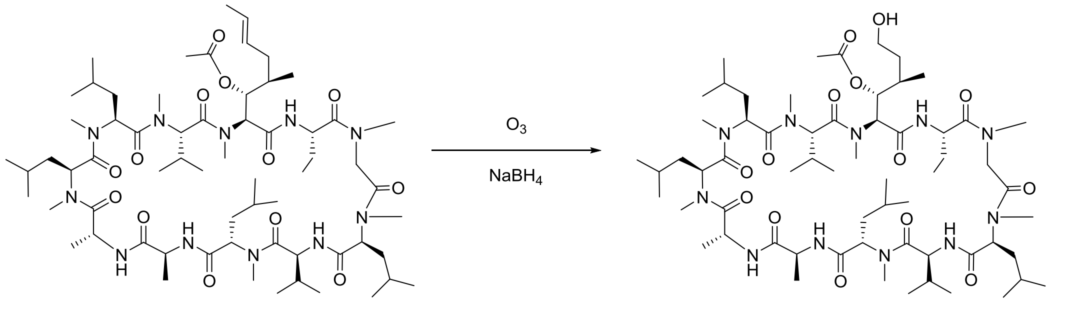

S48-1 **S48-2**

A stirred solution of acetylcyclosporin A (**S48-1**) in CH_2Cl_2 (120 mL) cooled to –78 °C was saturated with ozone. After 30 mins, MeOH (60.0 mL) and excess sodium borohydride (900 mg) were added, and the reaction was allowed to warm to room temperature. Additional sodium borohydride (900 mg, excess) was added at room temperature. After 2 hours, the reaction was quenched with sat. aq. NH_4Cl (20.0 mL), and water (100 mL) was added. Organic solvents were removed by rotary evaporation and the crude product was extracted with EtOAc (50.0 mL×5). The combined organic extracts were dried ($MgSO_4$), filtered

through a pad of silica gel and concentrated *in vacuo* to give 778 mg crude product (**S48-2**). The crude product was used without further purification.[5]

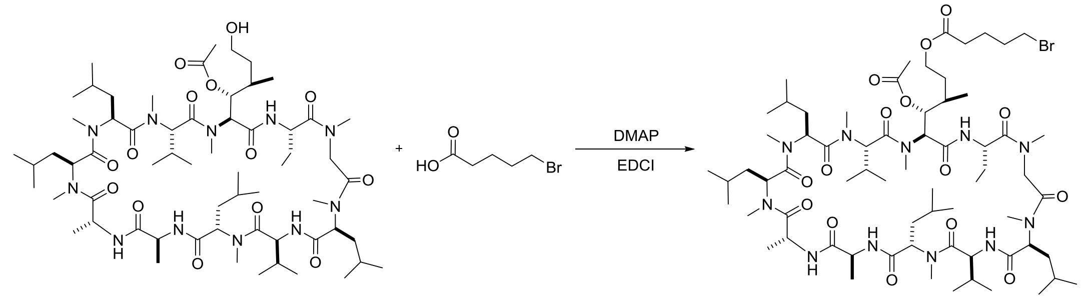

S48-2 **S48-3**

To a solution of the crude **S48-2** (778 mg, 630 mmol, 1.00 equiv.), DMAP (4-dimethylaminepyridine) (308 mg, 2.52 mmol, 4.00 equiv.) and EDCI (*N*-(3-dimethylaminopropyl)-*N*'-ethylcarbodiimide hydrochloride) (483 mg, 2.52 mmol, 4.00 equiv.) in dry CH_2Cl_2 (10.0 mL) at room temperature under N_2 were added 5-Bromovaleric acid (456 mg, 2.52 mmol, 4.00 equiv.). The reaction was stirred at room temperature for 12 h before diluted with CH_2Cl_2 (20.0 mL) and quenched with H_2O (20.0 mL). The aqueous layer was separated and extracted times with CH_2Cl_2 (50.0 mL × 3). The combined organic layer was dried over anhydrous $MgSO_4$, filtered through a pad of silica gel and concentrated *in vacuo* to give crude product (**S48-3**). The crude product was used for next step directly.

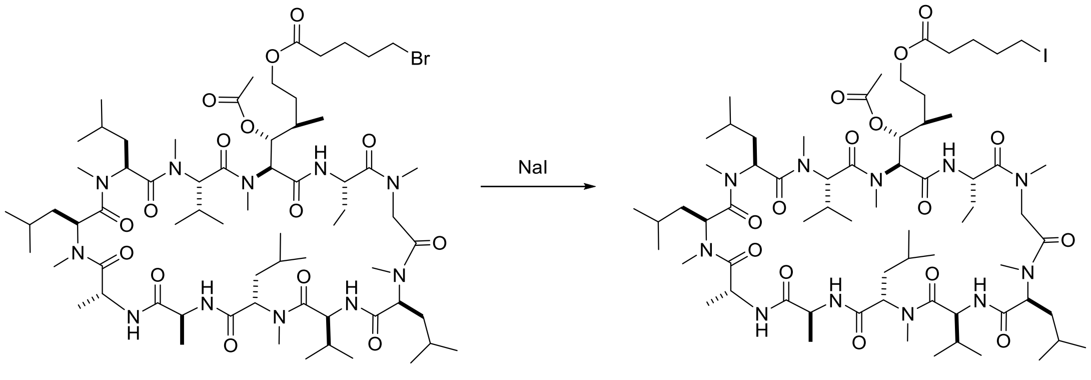

S48-3 **S48**, 30%

The crude **S48-3** was dissolved with acetone (2.0 mL), sodium iodide (250 mg) was added at room temperature. Subsequently, the reaction flask was wrapped in aluminum foil and refluxed at 75 °C for 4 h. Afterwards the solvent was removed in reduced pressure. The solid was dissolved in EtOAc and washed successively with water 2 times and a saturated solution of sodium chloride. The organic phase was dried over anhydrous $MgSO_4$, filtered and concentrated in *vacuo*. The residue was purified by chromatography on silica gel, eluting with CH_2Cl_2/CH_3OH 60:1 (v/v) to afford 440 mg 5-iodopentyl 4-ethynylbenzoate (**S48**) as a white solid (30% yield).

R_f = 0.1 (CH_2Cl_2/CH_3OH 60:1 (v/v)). ^{1}H NMR (400 MHz, $CDCl_3$) δ 8.58 (d, *J* = 9.8 Hz, 1H), 7.99 (d, *J* = 6.8 Hz, 1H), 7.57 (d, *J* = 7.7 Hz, 1H), 7.46 (d, *J* = 9.1 Hz, 1H), 5.65 (dd, *J* = 11.0, 3.9 Hz, 1H), 5.50 – 5.42 (m, 2H), 5.39 (dd, *J* = 12.0, 3.3 Hz, 1H), 5.25 (dd, *J* = 11.5, 3.8 Hz, 1H), 5.16 – 5.08 (m, 1H), 4.95 (d, *J* = 11.2 Hz, 2H), 4.87 – 4.77 (m, 1H), 4.71 (t, *J* = 9.5 Hz, 1H), 4.62 (d, *J* = 13.8 Hz, 1H), 4.43 – 4.32 (m, 1H), 4.31 – 4.19 (m, 1H), 3.90 – 3.73 (m, 1H), 3.40 (s, 3H), 3.27 (s, 3H), 3.26 (s, 3H), 3.22 – 3.17 (m, 5H), 3.15 (d, *J* = 13.7 Hz, 1H), 3.06 (s,

3H), 2.64 (s, 3H), 2.63 (s, 3H), 2.50 – 2.36 (m, 1H), 2.36 – 2.06 (m, 6H), 2.05 – 1.77 (m, 10H), 1.77 – 1.60 (m, 5H), 1.47 – 1.10 (m, 12H), 1.08 – 0.71 (m, 42H). ^{13}C NMR (101 MHz, $CDCl_3$) δ 173.8, 173.6, 173.1, 172.9, 172.7, 171.6, 171.3, 171.1, 170.9, 170.5, 170.5, 170.2, 168.0, 73.7, 63.5, 58.6, 57.3, 56.2, 55.4, 54.8, 54.0, 50.0, 48.8, 48.5, 48.0, 44.7, 41.0, 39.4, 39.2, 37.2, 35.9, 33.0, 32.9, 32.3, 31.7, 31.6, 31.4, 30.4, 30.0, 29.8, 29.6, 28.8, 25.8, 25.3, 25.2, 25.0, 24.9, 24.8, 24.6, 24.3, 23.9, 23.9, 23.6, 23.6, 22.0, 21.5, 21.3, 20.8, 20.6, 19.8, 18.7, 18.5, 18.2, 17.7, 15.1, 10.0, 6.2. Mass Spectrometry: HRMS-ESI (*m*/*z*): Calcd for $C_{67}H_{118}IN_{11}NaO_{15}$ $[M+Na]^+$, 1466.7746. Found, 1466.7748.

11-Benzyloxymethylenoxy 22-iodopleuromutilin (S49)

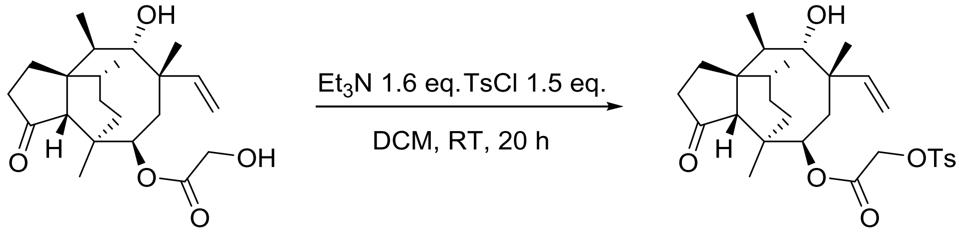

S49-1, 64%

To a solution of pleuromutilin (5.00 g, 13.2 mmol, 1.00 equiv.) and Et_3N (2.75 mL, 19.8 mmol, 1.50 equiv.) in dry CH_2Cl_2 (50.0 mL) at 0 °C was added TsCl (3.53 g, 18.5 mmol, 1.40 equiv.). Subsequently, the reaction mixture was warmed to RT for 20 h. Afterwards the cooled mixture was concentrated *in vacuo*. The residue was purified by chromatography on silica gel, eluting with *n*-hexane/EtOAc 4:1 (v/v) to afford 4.50 g 22-O-tosylpleuromutilin (**S49-1**) as a white solid (64% yield).

R_f = 0.2 (*n*-hexane/EtOAc 2:1 (v/v)). NMR Spectroscopy: ^{1}H NMR (400 MHz, $CDCl_3$) δ 7.81 (d, *J* = 8.2 Hz, 2H), 7.34 (d, *J* = 8.1 Hz, 2H), 6.41 (dd, *J* = 17.4, 11.0 Hz, 1H), 5.76 (d, *J* = 8.5 Hz, 1H), 5.32 (d, *J* = 11.0 Hz, 1H), 5.18 (d, *J* = 17.4 Hz, 1H), 4.47 (s, 2H), 3.34 (s, 1H), 2.45 (s, 3H), 2.33 – 1.97 (m, 5H), 1.82 – 1.04 (m, 15H), 0.87 (d, *J* = 7.0 Hz, 3H), 0.62 (d, *J* = 7.0 Hz, 3H). ^{13}C NMR (101 MHz, $CDCl_3$) δ 216.9, 165.0, 145.4, 138.8, 132.8, 130.1, 128.2, 117.5, 74.7, 70.4, 65.2, 58.2, 45.5, 44.7, 44.1, 42.0, 36.7, 36.2, 34.5, 30.5, 26.9, 26.5, 25.0, 21.8, 16.7, 14.9, 11.6. Mass Spectrometry: HRMS-ESI (*m*/*z*): Calcd for $C_{29}H_{40}NaO_7S$ $[M+Na]^+$, 555.2387. Found, 555.2390.

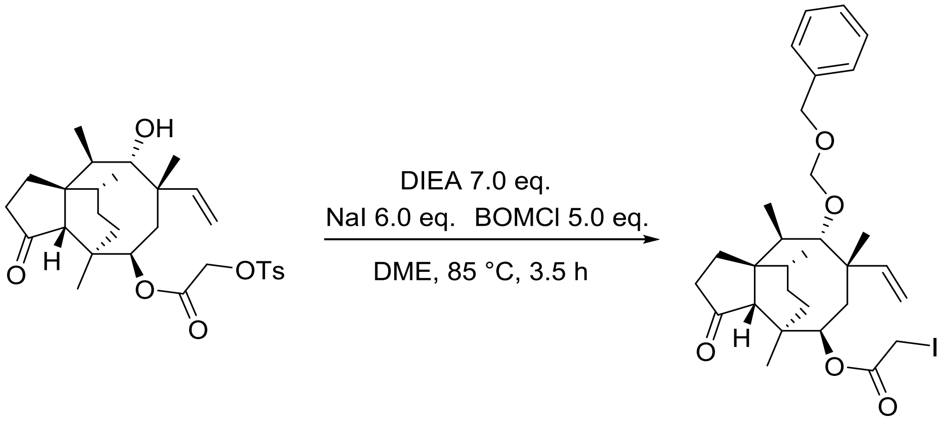

S49-1 **S49,** 55%

To a solution of 22-O-tosylpleuromutilin (**S49-1**) (0.600 g, 1.59 mmol, 1.00 equiv.) and DIEA (*N*,*N*-diisopropylethylamine) (1.83 mL, 11.1 mmol, 7.00 equiv.) in dry DME (1,2-dimethoxyethane) (20.0 mL) at 24 °C were added sodium iodide (1.43 g, 9.51 mmol, 6.00 equiv.) and BOMCl (benzyl chloromethyl ether) (1.32 mL, 9.51 mmol, 6.00 equiv.). The reaction vessel was sealed and placed in an 85 °C oil bath. The reaction mixture was stirred and heated for 3.5 h at 85 °C. The product mixture was allowed to cool over 30 mins to 0 °C with an ice bath. A saturated aqueous sodium bicarbonate solution (20.0 mL) was added dropwise via syringe to the product mixture. The resulting mixture was stirred for 10 mins at 0 °C. The resulting mixture was transferred to a separatory funnel charged with CH_2Cl_2 (50.0 mL), extracted, then the aqueous layer was further extracted with CH_2Cl_2 (20.0 mL × 3). The organic layer was combined, and the combined organic layer was dried over sodium sulfate. The dried solution was filtered, and the filtrate was concentrated *in vacuo*. The residue was purified by chromatography on silica gel, eluting with *n*-hexane/EtOAc 8:1 (v/v) to afford 543 mg 11-benzyloxymethylenoxy 22-iodopleuromutilin (**S49)** as a white solid (55% yield).[6]

R_f = 0.3 (*n*-hexane /EtOAc 5:1 (v/v)). NMR Spectroscopy: ^{1}H NMR (400 MHz, $CDCl_3$) δ 7.39 – 7.27 (m, 5H), 6.25 (dd, *J* = 17.6, 11.2 Hz, 1H), 5.65 (d, *J* = 8.4 Hz, 1H), 5.32 – 5.17 (m, 2H), 4.84 – 4.75 (m, 2H), 4.67 (s, 2H), 3.70 – 3.51 (m, 2H), 3.37 (d, *J* = 6.0 Hz, 1H), 2.47 – 2.33 (m, 1H), 2.30 – 1.97 (m, 4H), 1.83 – 1.07 (m, 14H), 0.98 (d, *J* = 7.1 Hz, 3H), 0.71 (d, *J* = 6.6 Hz, 3H). ^{13}C NMR (101 MHz, $CDCl_3$) δ 217.3, 166.7, 140.1, 138.0, 128.6, 127.9, 127.8, 116.3, 97.0, 83.7, 71.0, 71.0, 58.7, 45.6, 44.8, 44.6, 42.2, 37.2, 36.9, 34.8, 30.6, 29.1, 26.9, 25.3, 16.7, 15.0, 12.2, -3.7. Mass Spectrometry: HRMS-ESI (*m*/*z*): Calcd for $C_{30}H_{41}INaO_5$ $[M+Na]^+$, 631.1891. Found, 631.1894.

5-Trifluoromethoxypentyl 4-fluorobenzoate (3)

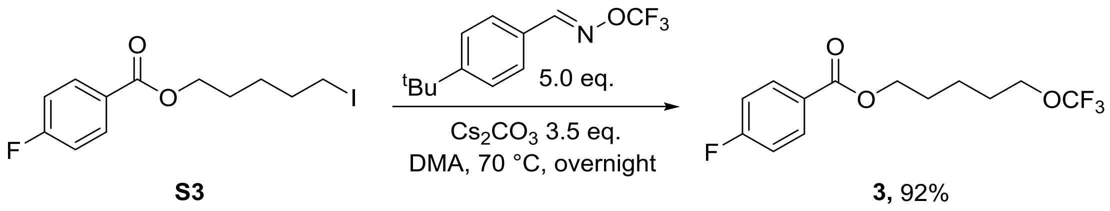

In a N_2 glovebox, to 5-iodopentyl 4-fluorobenzoate (**S3**) (84.0 mg, 0.250 mmol, 1.00 equiv.), (*E*)-*O*-trifluoromethyl-4-*tert*-butyl-benzaldoximes (**1a**) (307 mg, 1.25 mmol, 5.00 equiv.) in a 15.0 mL sealed vial were added DMA (2.00 mL). Cs_2CO_3 (285 mg, 0.875 mmol, 3.50 equiv.) was added to the reaction and the resulting mixture was stirred for overnight at 70 °C. After cooling to 50 °C, NMO (4-methylmorpholine *N*-oxide) (58.6 mg, 0.500 mmol, 2.00 equiv.) was added and the reaction mixture was stirred 2 h. Then the resulting mixture was filtered and concentrated *in vacuo*. The residue was purified by preparative TLC, eluting with *n*-hexane/EtOAc 50:1 (v/v) to afford 67.4 mg 5-trifluoromethoxypentyl 4-fluorobenzoate (**3**) as a colorless liquid (92% yield).

R_f= 0.20 (*n*-hexane/EtOAc 30:1 (v/v)). NMR Spectroscopy: ^{1}H NMR (400 MHz, $CDCl_3$) δ 8.10-7.99 (m, 2H), 7.16-7.04 (m, 2H), 4.33 (t, *J* = 6.4 Hz, 2H), 3.99 (t, *J* = 6.4 Hz, 2H), 1.88 – 1.71 (m, 4H), 1.58-1.54 (m, 2H).^{13}C NMR (101 MHz, $CDCl_3$) δ 165.9 (d, *J* = 253.7 Hz),

165.7, 132.2 (d, J = 9.4 Hz), 126.7 (d, J = 3.0 Hz), 121.8 (q, J = 253.8 Hz), 115.6 (d, J = 22.0 Hz), 67.2 (q, J = 3.1 Hz), 64.8, 28.4, 28.3, 22.3. ^{19}F NMR (376 MHz, $CDCl_3$) δ -60.4 (s, 3F), -105.5 – -105.6 (m, 1F). Mass Spectrometry: HRMS-EI (*m/z*): Calcd for $C_{13}H_{14}F_4O_3$ [M], 294.0879. Found, 294.0876.

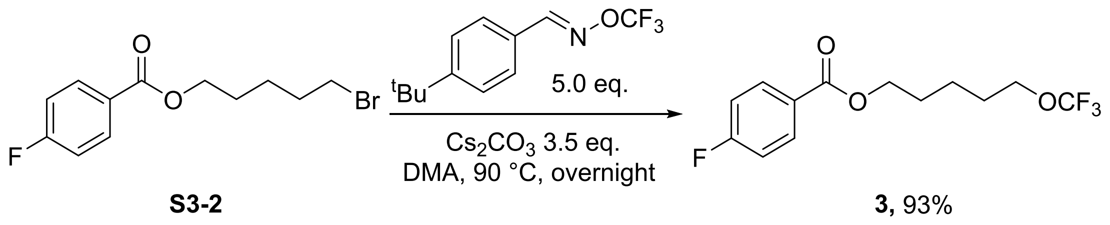

In a N_2 glovebox, to 5-bromopentyl 4-fluorobenzoate (**S3-2**) (63.1 mg, 0.250 mmol, 1.00 equiv.), (*E*)-*O*-trifluoromethyl-4-*tert*-butyl-benzaldoximes (**1a**) (307 mg, 1.25 mmol, 5.00 equiv.) in a 15.0 mL sealed vial were added DMA (2.00 mL). Cs_2CO_3 (285 mg, 0.875 mmol, 3.50 equiv.) was added to the reaction and the resulting mixture was stirred for overnight at 90 °C. After cooling to 70 °C, NMO (4-methylmorpholine *N*-oxide) (58.6 mg, 0.500 mmol, 2.00 equiv.) was added and the reaction mixture was stirred 2 h. Then the resulting mixture was filtered and concentrated *in vacuo*. The residue was purified by preparative TLC, eluting with *n*-hexane/EtOAc 50:1 (v/v) to afford 60.0 mg 5-trifluoromethoxypentyl 4-fluorobenzoate (**3**) as a colorless liquid (93% yield).

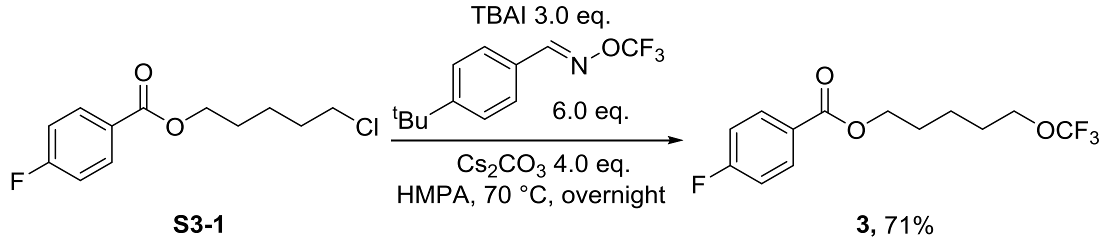

In a N_2 glovebox, to 5-chloropentyl 4-fluorobenzoate (**S3-1**) (61.2 mg, 0.250 mmol, 1.00 equiv.), TBAI (tetrabutylammonium iodide) (277 mg, 0.750 mmol, 3.00 equiv.), (*E*)-*O*-trifluoromethyl-4-*tert*-butyl-benzaldoximes (**1a**) (368 mg, 1.50 mmol, 6.00 equiv.) in a 2.0 mL sealed vial were added HMPA (0.400 mL). Cs_2CO_3 (326 mg, 1.00 mmol, 4.00 equiv.) was added to the reaction and the resulting mixture was stirred for overnight at 70 °C. NaI (74.9 mg, 0.500 mmol, 2.00 equiv.) was added and the reaction mixture was stirred 3 h. After cooling to room temperature, the resulting mixture was filtered through a pad of silica gel and concentrated *in vacuo*. NMO (4-methylmorpholine *N*-oxide) (58.6 mg, 0.500 mmol, 2.00 equiv.) was added and the reaction mixture was stirred 2 h at 50 °C. Then the resulting mixture was filtered and concentrated *in vacuo*. The residue was purified by preparative TLC, eluting with *n*-hexane/EtOAc 50:1 (v/v) to afford 52.0 mg 5-trifluoromethoxypentyl 4-fluorobenzoate (**3**) as a colorless liquid (71% yield).

1-((5-Trifluoromethoxypentyl)oxy)-4-fluorobenzene (4)

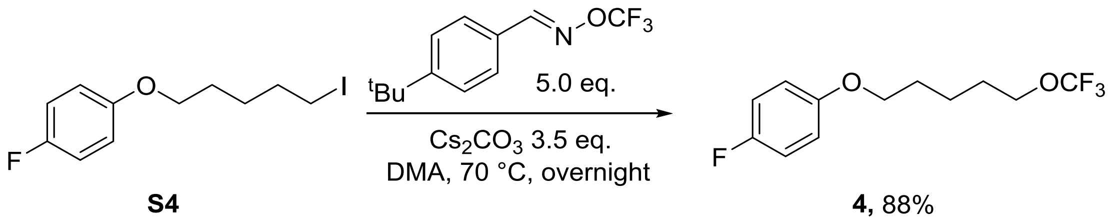

In a N_2 glovebox, to 1-((5-iodopentyl)oxy)-4-fluorobenzene (**S4**) (77.0 mg, 0.250 mmol, 1.00 equiv.), (*E*)-*O*-trifluoromethyl-4-*tert*-butyl-benzaldoximes (**1a**) (307 mg, 1.25 mmol, 5.00 equiv.) in a 15.0 mL sealed vial were added DMA (2.00 mL). Cs_2CO_3 (285 mg, 0.875 mmol, 3.50 equiv.) was added to the reaction and the resulting mixture was stirred for overnight at 70 °C. After cooling to 50 °C, NMO (4-methylmorpholine *N*-oxide) (58.6 mg, 0.500 mmol, 2.00 equiv.) was added and the reaction mixture was stirred 2 h. Then the resulting mixture was filtered and concentrated *in vacuo*. The residue was purified by preparative TLC, eluting with *n*-hexane/EtOAc 60:1 (v/v) to afford 58.4 mg 1-((5-trifluoromethoxypentyl)oxy)-4-fluorobenzene (**4**) as a colorless liquid (88% yield).

R_f = 0.3 (*n*-hexane/EtOAc 30:1 (v/v)). NMR Spectroscopy: ^{1}H NMR (400 MHz, $CDCl_3$) δ 7.01 – 6.92 (m, 2H), 6.86 – 6.78 (m, 2H), 3.99 (t, *J* = 6.4 Hz, 2H), 3.93 (t, *J* = 6.3 Hz, 2H), 1.87 – 1.71 (m, 4H), 1.64 – 1.54 (m, 2H).^{13}C NMR (101 MHz, $CDCl_3$) δ 157.3 (d, *J* = 237.9 Hz), 155.2 (d, *J* = 2.1 Hz), 121.8 (q, *J* = 253.7 Hz), 115.9 (d, *J* = 23.0 Hz), 115.5 (d, *J* = 8.0 Hz), 68.3, 67.4 (q, *J* = 3.1 Hz), 28.9, 28.6 22.4. ^{19}F NMR (376 MHz, $CDCl_3$) δ -60.7 (s, 3F), -124.1 – -124.4 (m, 1F). Mass Spectrometry: HRMS-EI (*m/z*): Calcd for $C_{12}H_{14}F_4O_2$ [M], 266.0930. Found, 266.0922.

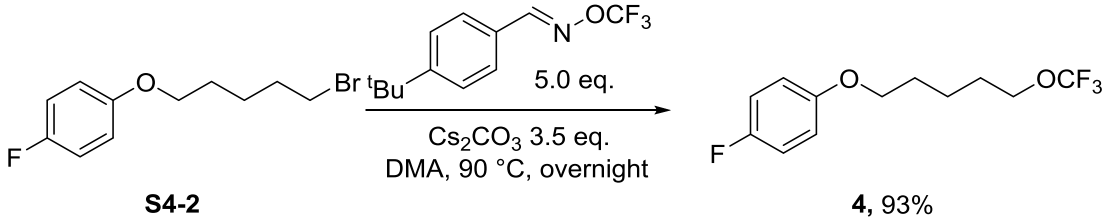

In a N_2 glovebox, to 1-((5-bromopentyl)oxy)-4-fluorobenzene (**S4-2**) (68.9 mg, 0.264 mmol, 1.00 equiv.), (*E*)-*O*-trifluoromethyl-4-*tert*-butyl-benzaldoximes (**1a**) (324 mg, 1.32 mmol, 5.00 equiv.) in a 15.0 mL sealed vial were added DMA (2.10 mL). Cs_2CO_3 (301 mg, 0.924 mmol, 3.50 equiv.) was added to the reaction and the resulting mixture was stirred for overnight at 90 °C. After cooling to 70 °C, NMO (4-methylmorpholine *N*-oxide) (61.3 mg, 0.528 mmol, 2.00 equiv.) was added and the reaction mixture was stirred 2 h. Then the resulting mixture was filtered and concentrated *in vacuo*. The residue was purified by preparative TLC, eluting with *n*-hexane/EtOAc 60:1 (v/v) to afford 65.3 mg 1-((5-trifluoromethoxypentyl)oxy)-4-fluorobenzene (**4**) as a colorless liquid (93% yield).

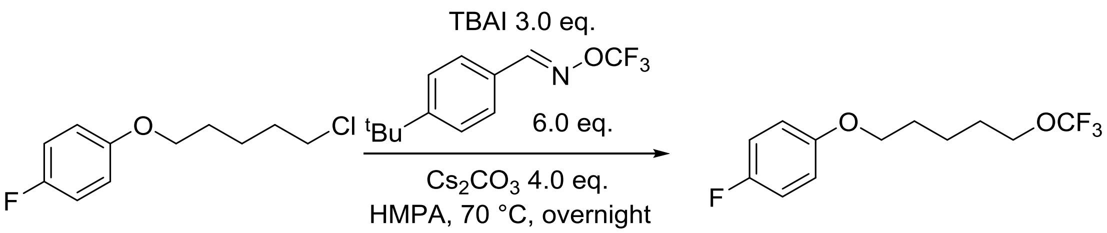

S4-1 **4,** 71%

In a N_2 glovebox, to 1-((5-chloropentyl)oxy)-4-fluorobenzene (**S4-1**) (54.2 mg, 0.250 mmol, 1.00 equiv.), TBAI (tetrabutylammonium iodide) (277 mg, 0.750 mmol, 3.00 equiv.), (*E*)-*O*-trifluoromethyl-4-*tert*-butyl-benzaldoximes (**1a**) (368 mg, 1.50 mmol, 6.00 equiv.) in a 2.0 mL sealed vial were added HMPA (0.400 mL). Cs_2CO_3 (326 mg, 1.00 mmol, 4.00 equiv.) was added to the reaction and the resulting mixture was stirred for overnight at 70 °C. NaI (74.9 mg, 0.500 mmol, 2.00 equiv.) was added and the reaction mixture was stirred 3 h. After cooling to room temperature, the resulting mixture was filtered through a pad of silica gel and concentrated *in vacuo*. NMO (4-methylmorpholine *N*-oxide) (58.6 mg, 0.500 mmol, 2.00 equiv.) was added and the reaction mixture was stirred 2 h at 50 °C. Then the resulting mixture was filtered and concentrated *in vacuo*. The residue was purified by preparative TLC, eluting with *n*-hexane/EtOAc 60:1 (v/v) to afford 38.5 mg 1-((5-trifluoromethoxypentyl)oxy)-4-fluorobenzene (**4**) as a colorless liquid (71% yield).

1-((5-Trifluormethoxypentyl)oxy)-4-chlorobenzene (5)

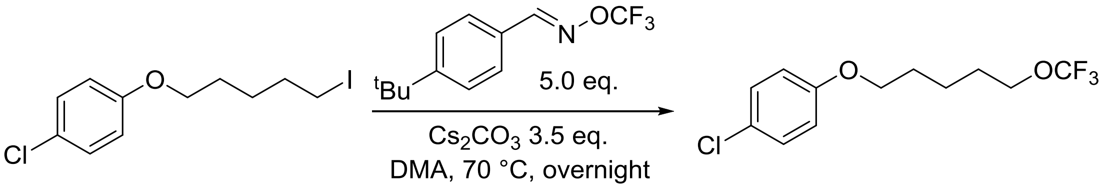

S5 **5,** 98%

In a N_2 glovebox, to 1-((5-iodopentyl)oxy)-4-chlorobenzene (**S5**) (81.2 mg, 0.250 mmol, 1.00 equiv.), (*E*)-*O*-trifluoromethyl-4-*tert*-butyl-benzaldoximes (**1a**) (307 mg, 1.25 mmol, 5.00 equiv.) in a 15.0 mL sealed vial were added DMA (2.00 mL). Cs_2CO_3 (285 mg, 0.875 mmol, 3.50 equiv.) was added to the reaction and the resulting mixture was stirred for overnight at 70 °C. After cooling to 50 °C, NMO (4-methylmorpholine *N*-oxide) (58.6 mg, 0.500 mmol, 2.00 equiv.) was added and the reaction mixture was stirred 2 h. Then the resulting mixture was filtered and concentrated *in vacuo*. The residue was purified by preparative TLC, eluting with *n*-hexane/EtOAc 80:1 (v/v) to afford 69.3 mg 1-((5-trifluormethoxypentyl)oxy)-4-chlorobenzene (**5**) as a colorless liquid (98% yield).

R_f = 0.4 (*n*-hexane/EtOAc 30:1 (v/v)). NMR Spectroscopy: ^1H NMR (400 MHz, $CDCl_3$) δ 7.17 – 7.10 (m, 2H), 6.76 – 6.69 (m, 2H), 3.90 (t, *J* = 6.4 Hz, 2H), 3.84 (t, *J* = 6.3 Hz, 2H), 1.78 – 1.61 (m, 4H), 1.56 – 1.41 (m, 2H). ^{13}C NMR (101 MHz, $CDCl_3$) δ 157.7, 129.4, 121.8 (q, *J* = 253.7 Hz), 115.8, 67.9, 67.4 (q, *J* = 3.0 Hz), 28.8, 28.6, 22.3. ^{19}F NMR (376 MHz, $CDCl_3$) δ -60.6 (s, 3F). Mass Spectrometry: HRMS-EI (*m/z*): Calcd for $C_{12}H_{14}ClF_3O_2$ [M],

282.0634. Found, 282.0630.

1-((5-Trifluoromethoxypentyl)oxy)-4-bromobenzene (6)

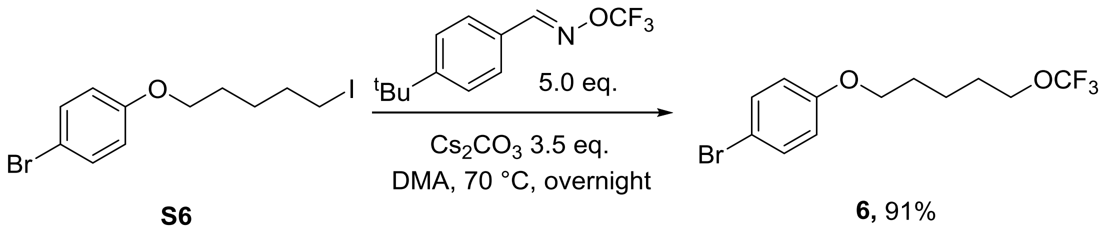

In a N_2 glovebox, to 1-((5-iodopentyl)oxy)-4-bromobenzene (**S6**) (92.3 mg, 0.250 mmol, 1.00 equiv.), (*E*)-*O*-trifluoromethyl-4-*tert*-butyl-benzaldoximes (**1a**) (307 mg, 1.25 mmol, 5.00 equiv.) in a 15.0 mL sealed vial were added DMA (2.00 mL). Cs_2CO_3 (285 mg, 0.875 mmol, 3.50 equiv.) was added to the reaction and the resulting mixture was stirred for overnight at 70 °C. After cooling to 50 °C, NMO (4-methylmorpholine *N*-oxide) (58.6 mg, 0.500 mmol, 2.00 equiv.) was added and the reaction mixture was stirred 2 h. Then the resulting mixture was filtered and concentrated *in vacuo*. The residue was purified by preparative TLC, eluting with *n*-hexane/EtOAc 60:1 (v/v) to afford 74.3 mg 1-((5-trifluoromethoxypentyl)oxy)-4-bromobenzene (**6**) as a colorless liquid (91% yield).

R_f = 0.3 (*n*-hexane/EtOAc 30:1 (v/v)). NMR Spectroscopy: ^{1}H NMR (400 MHz, $CDCl_3$) δ 7.37 (d, *J* = 8.9 Hz, 2H), 6.77 (d, *J* = 8.9 Hz, 2H), 3.96 (dt, *J* = 22.8, 6.3 Hz, 4H), 1.79 (dp, *J* = 20.6, 6.5 Hz, 4H), 1.65 – 1.52 (m, 2H). ^{13}C NMR (101 MHz, CDC13) δ 158.2, 132.4, 121.8 (q, *J* = 253.8 Hz), 116.4, 112.9, 67.9, 67.3 (q, *J* = 3.1 Hz), 28.8, 28.6, 22.3. ^{19}F NMR (376 MHz, DMSO) δ -60.9 (s, 3F). Mass Spectrometry: HRMS-EI (*m/z*): Calcd for $C_{12}H_{14}BrF_3O_2$ [M], 326.0129. Found, 326.0118.

1-((5-Trifluoromethoxypentyl)oxy)-4-iodobenzene (7)

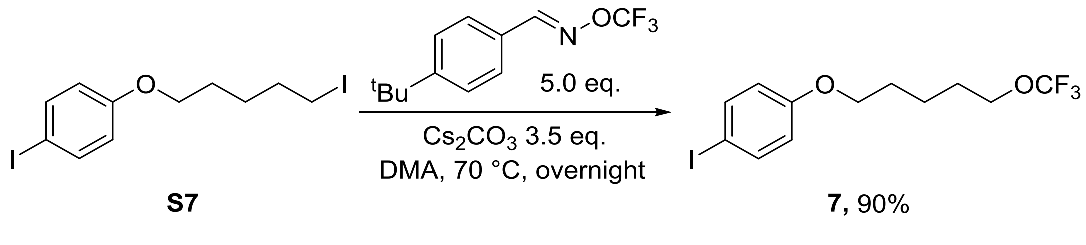

In a N_2 glovebox, to 1-((5-iodopentyl)oxy)-4-iodobenzene (**S7**) (104 mg, 0.250 mmol, 1.00 equiv.), (*E*)-*O*-trifluoromethyl-4-*tert*-butyl-benzaldoximes (**1a**) (307 mg, 1.25 mmol, 5.00 equiv.) in a 15.0 mL sealed vial were added DMA (2.00 mL). Cs_2CO_3 (285 mg, 0.875 mmol, 3.50 equiv.) was added to the reaction and the resulting mixture was stirred for overnight at 70 °C. After cooling to 50 °C, NMO (4-methylmorpholine *N*-oxide) (58.6 mg, 0.500 mmol, 2.00 equiv.) was added and the reaction mixture was stirred 2 h. Then the resulting mixture was filtered and concentrated *in vacuo*. The residue was purified by preparative TLC, eluting with *n*-hexane/EtOAc 60:1 (v/v) to afford 84.0 mg 1-((5-trifluoromethoxypentyl)oxy)-4-iodobenzene (**7**) as a colorless liquid (90% yield).

R_f = 0.3 (*n*-hexane/EtOAc 30:1 (v/v)). NMR Spectroscopy: ^{1}H NMR (400 MHz, $CDCl_3$) δ 7.57 – 7.52 (m, 2H), 6.70 – 6.63 (m, 2H), 3.99 (t, *J* = 6.4 Hz, 2H), 3.93 (t, *J* = 6.3 Hz, 2H), 1.87 – 1.71 (m, 4H), 1.63 – 1.54 (m, 2H). ^{13}C NMR (101 MHz, $CDCl_3$) δ 150.0, 138.3, 121.8 (q, *J* = 254.0 Hz), 117.0, 82.8, 67.8, 67.4 (q, *J* = 3.1 Hz), 28.7, 28.6, 22.3. ^{19}F NMR (376 MHz, $CDCl_3$) δ -60.4 (s, 3F). Mass Spectrometry: HRMS-EI (*m/z*): Calcd for $C_{12}H_{14}F_3IO_2$ [M], 373.9991. Found, 373.9989.

1-(*tert*-Butyl)-4-((5-trifluoromethoxypentyl)oxy)benzene (8)

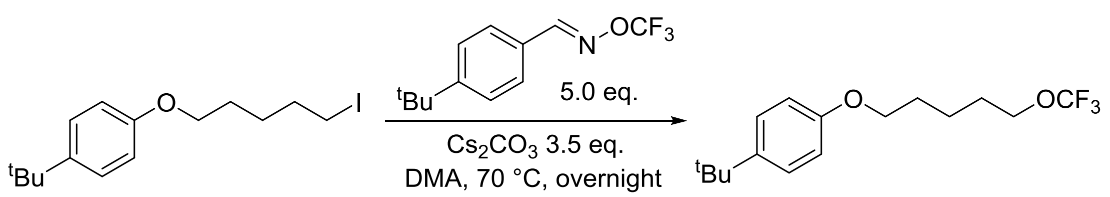

S8 **8**, 94%

In a N_2 glovebox, to 1-(*tert*-butyl)-4-((5-iodopentyl)oxy)benzene (**S8**) (94.0 mg, 0.250 mmol, 1.00 equiv.), (*E*)-*O*-trifluoromethyl-4-*tert*-butyl-benzaldoximes (**1a**) (307 mg, 1.25 mmol, 5.00 equiv.) in a 15.0 mL sealed vial were added DMA (2.00 mL). Cs_2CO_3 (285 mg, 0.875 mmol, 3.50 equiv.) was added to the reaction and the resulting mixture was stirred for overnight at 70 °C. After cooling to 50 °C, NMO (4-methylmorpholine *N*-oxide) (58.6 mg, 0.500 mmol, 2.00 equiv.) was added and the reaction mixture was stirred 2 h. Then the resulting mixture was filtered and concentrated *in vacuo*. The residue was purified by preparative TLC, eluting with *n*-hexane/EtOAc 80:1 (v/v) to afford 77.4 mg 1-(*tert*-butyl)-4-((5-trifluoromethoxypentyl)oxy)benzene (**8**) as a colorless liquid (94% yield).

R_f = 0.4 (*n*-hexane/EtOAc 30:1 (v/v)). NMR Spectroscopy: ^{1}H NMR (400 MHz, $CDCl_3$) δ 7.38 – 7.28 (m,2H), 6.93 – 6.77 (m, 2H), 4.06 – 3.93 (m, 4H), 1.91 – 1.72 (m, 4H), 1.69 – 1.54 (m, 2H), 1.34 (s, 9H). ^{13}C NMR (101 MHz, $CDCl_3$) δ 156.8, 143.5, 126.4, 121.8 (q, *J* = 253.7 Hz), 114.0, 67.6, 67.4 (q, *J* = 2.7 Hz), 34.2, 31.7, 28.9, 28.6, 22.4. ^{19}F NMR (376 MHz, $CDCl_3$) δ -60.8 (s, 3F). Mass Spectrometry: HRMS-EI (*m/z*): Calcd for $C_{16}H_{23}F_3O_2$ [M], 304.1650. Found, 304.1639.

1-((5-Trifluoromethoxypentyl)oxy)-4-nitrobenzene (9)

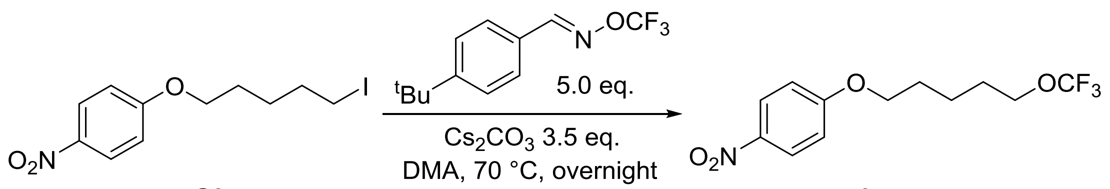

S9 **9**, 86%

In a N_2 glovebox, to 1-((5-iodopentyl)oxy)-4-nitrobenzene (**S9**) (83.8 mg, 0.250 mmol, 1.00 equiv.), (*E*)-*O*-trifluoromethyl-4-*tert*-butyl-benzaldoximes (**1a**) (307 mg, 1.25 mmol, 5.00 equiv.) in a 15.0 mL sealed vial were added DMA (2.00 mL). Cs_2CO_3 (285 mg, 0.875 mmol, 3.50 equiv.) was added to the reaction and the resulting mixture was stirred for overnight at 70 °C. After cooling to 50 °C, NMO (4-methylmorpholine *N*-oxide) (58.6 mg, 0.500 mmol,

2.00 equiv.) was added and the reaction mixture was stirred 2 h. Then the resulting mixture was filtered and concentrated *in vacuo*. The residue was purified by preparative TLC, eluting with *n*-hexane/EtOAc 40:1 (v/v) to afford 63.0 mg 1-((5-trifluoromethoxypentyl)oxy)-4-nitrobenzene (**9**) as a colorless liquid (86% yield).

R_f = 0.2 (*n*-hexane/EtOAc 20:1 (v/v)). NMR Spectroscopy: ^{1}H NMR (400 MHz, $CDCl_3$) δ 8.21 – 8.13 (m, 2H), 6.97 – 6.88 (m, 2H), 4.06 (t, *J* = 6.3 Hz, 2H), 4.00 (t, *J* = 6.3 Hz, 2H), 1.92 – 1.82 (m, 2H), 1.76 (q, *J* = 7.3, 6.3 Hz, 2H), 1.65 – 1.54 (m, 2H). ^{13}C NMR (101 MHz, $CDCl_3$) δ 164.2, 141.5, 126.0, 121.6 (q, *J* = 253.7 Hz), 114.5, 68.5, 67.3 (q, *J* = 3.0 Hz), 28.6, 28.5, 22.2.^{19}F NMR (376 MHz, $CDCl_3$) δ -60.8 (s, 3F). Mass Spectrometry: HRMS-EI (*m/z*): Calcd for $C_{12}H_{14}F_3NO_4$ [M], 293.0875. Found, 293.0869.

4-(4-Trifluoromethoxybutoxy)benzonitrile (10)

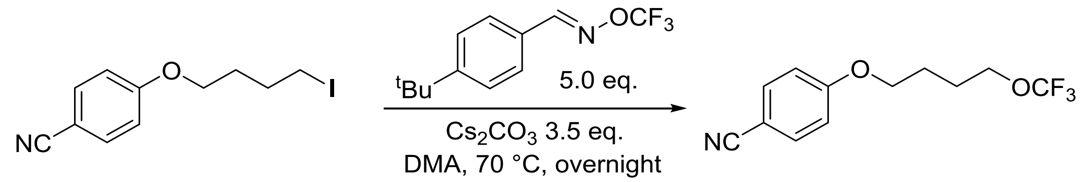

S10 **10,** 93%

In a N_2 glovebox, to 4-(4-iodobutoxy)benzonitrile (**S10**) (75.3 mg, 0.250 mmol, 1.00 equiv.), (*E*)-*O*-trifluoromethyl-4-*tert*-butyl-benzaldoximes (**1a**) (307 mg, 1.25 mmol, 5.00 equiv.) in a 15.0 mL sealed vial were added DMA (2.00 mL). Cs_2CO_3 (285 mg, 0.875 mmol, 3.50 equiv.) was added to the reaction and the resulting mixture was stirred for overnight at 70 °C. After cooling to 50 °C, NMO (4-methylmorpholine *N*-oxide) (58.6 mg, 0.500 mmol, 2.00 equiv.) was added and the reaction mixture was stirred 2 h. Then the resulting mixture was filtered and concentrated *in vacuo*. The residue was purified by preparative TLC, eluting with *n*-hexane/EtOAc 40:1 (v/v) to afford 60.0 mg 4-(4-trifluoromethoxybutoxy)benzonitrile (**10**) as a colorless liquid (93% yield).

R_f = 0.3 (*n*-hexane/EtOAc 20:1 (v/v)). NMR Spectroscopy: ^{1}H NMR (400 MHz, $CDCl_3$) δ 7.62 – 7.55 (m, 2H), 6.96 – 6.90 (m, 2H), 4.05 (t, *J* = 5.8 Hz, 2H), 2.00 – 1.84 (m, 2H). ^{13}C NMR (101 MHz, $CDCl_3$) δ 162.2, 134.1, 121.6 (q, *J* = 253.9 Hz), 119.3, 115.2, 103.9, 67.5, 66.9 (q, *J* = 3.2 Hz), 25.5, 25.3. ^{19}F NMR (376 MHz, $CDCl_3$) δ -60.4 (s, 3F). Mass Spectrometry: HRMS-EI (*m/z*): Calcd for $C_{12}H_{12}F_3NO_2$ [M], 259.0820. Found, 259.0814.

1-(4-Trifluoromethoxybutoxy)-4-(trifluoromethoxy)benzene (11)

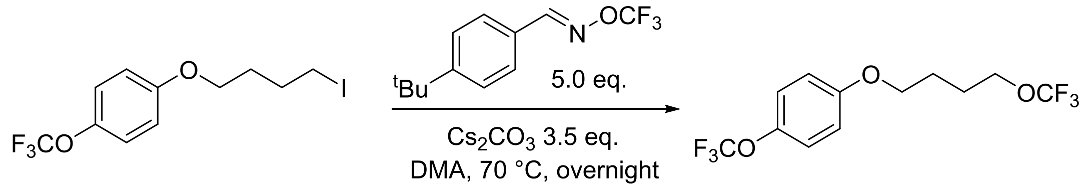

S11 **11,** 86%

In a N_2 glovebox, to 1-(4-iodobutoxy)-4-(trifluoromethoxy)benzene (**S11**) (93.5 mg, 0.250 mmol, 1.00 equiv.), (*E*)-*O*-trifluoromethyl-4-*tert*-butyl-benzaldoximes (**1a**) (307 mg, 1.25

mmol, 5.00 equiv.) in a 15.0 mL sealed vial were added DMA (2.00 mL). Cs_2CO_3 (285 mg, 0.875 mmol, 3.50 equiv.) was added to the reaction and the resulting mixture was stirred for overnight at 70 °C. After cooling to 50 °C, NMO (4-methylmorpholine *N*-oxide) (58.6 mg, 0.500 mmol, 2.00 equiv.) was added and the reaction mixture was stirred 2 h. Then the resulting mixture was filtered and concentrated *in vacuo*. The residue was purified by preparative TLC, eluting with *n*-hexane/EtOAc 80:1 (v/v) to afford 71.2 mg 1-(4-trifluoromethoxybutoxy)-4-(trifluoromethoxy)benzene (**11**) as a colorless liquid (86% yield).

R_f = 0.4 (*n*-hexane/EtOAc 30:1 (v/v)). NMR Spectroscopy: ^{1}H NMR (400 MHz, $CDCl_3$) δ 7.17 – 7.10 (m, 2H), 6.90 – 6.83 (m, 2H), 4.05 (t, *J* = 5.9 Hz, 2H), 3.98 (t, *J* = 5.7 Hz, 2H), 1.94 – 1.86 (m, 4H). ^{13}C NMR (101 MHz, $CDCl_3$) δ 157.6, 142.9 (q, *J* = 2.0 Hz), 122.6, 121.9 (q, *J* = 253.9 Hz), 120.8 (q, *J* = 255.8 Hz), 115.3, 67.6, 67.2 (q, *J* = 3.2 Hz), 25.7, 25.5. ^{19}F NMR (376 MHz, $CDCl_3$) δ -60.4 (s, 3F), -62.7 (s, 3F). Mass Spectrometry: HRMS-EI (*m/z*): Calcd for $C_{12}H_{12}F_6O_3$ [M], 318.0691. Found, 318.0682.

1-(4-((5-Trifluoromethoxypentyl)oxy)phenyl)ethanone (12)

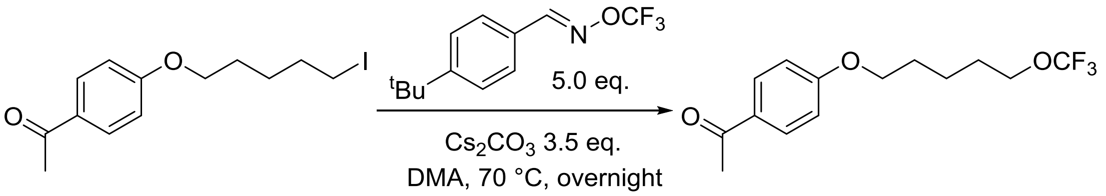

In a N_2 glovebox, to 1-(4-((5-iodopentyl)oxy)phenyl)ethanone (**S12**) (83.1 mg, 0.250 mmol, 1.00 equiv.), (*E*)-*O*-trifluoromethyl-4-*tert*-butyl-benzaldoximes (**1a**) (307 mg, 1.25 mmol, 5.00 equiv.) in a 15.0 mL sealed vial were added DMA (2.00 mL). Cs_2CO_3 (285 mg, 0.875 mmol, 3.50 equiv.) was added to the reaction and the resulting mixture was stirred for overnight at 70 °C. After cooling to 50 °C, NMO (4-methylmorpholine *N*-oxide) (58.6 mg, 0.500 mmol, 2.00 equiv.) was added and the reaction mixture was stirred 2 h. Then the resulting mixture was filtered and concentrated *in vacuo*. The residue was purified by preparative TLC, eluting with *n*-hexane/EtOAc 10:1 (v/v) to afford 65.6 mg 1-(4-((5-trifluoromethoxypentyl)oxy)phenyl)ethanone (**12**) as a colorless liquid (90% yield).

R_f = 0.3 (*n*-hexane/EtOAc 4:1 (v/v)). NMR Spectroscopy: ^{1}H NMR (400 MHz, $CDCl_3$) δ 7.91 (d, *J* = 7.9 Hz, 2H), 6.90 (d, *J* = 8.0 Hz, 2H), 4.07 – 3.91 (m, 4H), 2.53 (s, 3H), 1.90 – 1.69 (m, 4H), 1.65 – 1.48 (m, 2H). ^{13}C NMR (101 MHz, $CDCl_3$) δ 196.8, 163.0, 130.7, 130.3, 121.8 (q, *J* = 253.6 Hz), 114.2, 67.8, 67.3 (d, *J* = 3.0 Hz), 28.6, 28.5, 26.4, 22.3 . ^{19}F NMR (376 MHz, $CDCl_3$) δ -60.6 (s, 3F). Mass Spectrometry: HRMS-EI (*m/z*): Calcd for $C_{14}H_{17}F_3O_3$ [M], 290.1130. Found, 352.1120.

5-Trifluoromethoxypentyl (1,1'-biphenyl)-4-carboxylate (13)

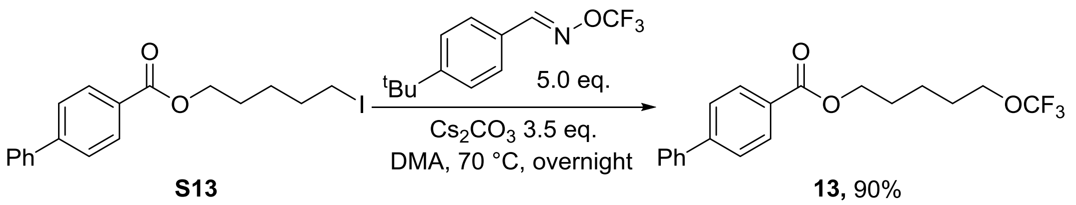

In a N_2 glovebox, to 5-iodopentyl (1,1'-biphenyl)-4-carboxylate (**S13**) (72.5 mg, 0.184 mmol, 1.00 equiv.), (*E*)-*O*-trifluoromethyl-4-*tert*-butyl-benzaldoximes (**1a**) (226 mg, 0.920 mmol, 5.00 equiv.) in a 15.0 mL sealed vial were added DMA (2.00 mL). Cs_2CO_3 (210 mg, 0.644 mmol, 3.50 equiv.) was added to the reaction and the resulting mixture was stirred for overnight at 70 °C. After cooling to 50 °C, NMO (4-methylmorpholine *N*-oxide) (58.6 mg, 0.500 mmol, 2.00 equiv.) was added and the reaction mixture was stirred 2 h. Then the resulting mixture was filtered and concentrated *in vacuo*. The residue was purified by preparative TLC, eluting with *n*-hexane/EtOAc 60:1 (v/v) to afford 58.1 mg 5-trifluoromethoxypentyl (1,1'-biphenyl)-4-carboxylate (**13**) as a white solid (90% yield).

R_f = 0.2 (*n*-hexane/EtOAc 20:1 (v/v)). NMR Spectroscopy: ^{1}H NMR (400 MHz, $CDCl_3$) δ 8.07 – 7.98 (m, 2H), 7.62 – 7.49 (m, 4H), 7.42 – 7.34 (m, 2H), 7.34 – 7.26 (m, 1H), 4.27 (t, *J* = 6.5 Hz, 2H), 3.91 (t, *J* = 6.4 Hz, 2H), 1.81 – 1.64 (m, 4H), 1.55 – 1.43 (m, 2H). ^{13}C NMR (101 MHz, $CDCl_3$) δ 166.6, 145.8, 140.1, 130.2, 129.2, 129.1, 128.3, 127.4, 127.2, 121.8 (q, *J* = 253.7 Hz), 67.3 (q, *J* = 2.9 Hz), 64.7, 28.5, 28.4, 22.3. ^{19}F NMR (376 MHz, $CDCl_3$) δ -60.7 (s, 3F). Mass Spectrometry: HRMS-EI (*m/z*): Calcd for $C_{19}H_{19}F_3O_3$ [M], 352.1286. Found, 352.1279.

5-Trifluoromethoxypentyl 4-formylbenzoate (14)

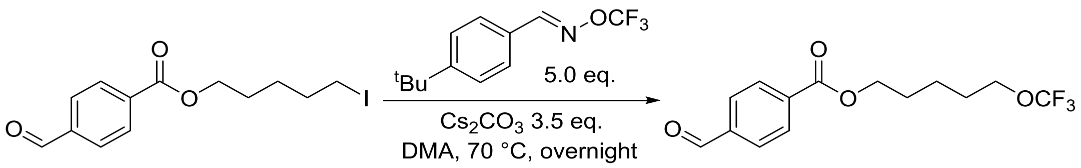

In a N_2 glovebox, to 5-iodopentyl 4-formylbenzoate (**S14**) (86.5 mg, 0.250 mmol, 1.00 equiv.), (*E*)-*O*-trifluoromethyl-4-*tert*-butyl-benzaldoximes (**1a**) (307 mg, 1.25 mmol, 5.00 equiv.) in a 15.0 mL sealed vial were added DMA (2.00 mL). Cs_2CO_3 (285 mg, 0.875 mmol, 3.50 equiv.) was added to the reaction and the resulting mixture was stirred for overnight at 70 °C. After cooling to 50 °C, NMO (4-methylmorpholine *N*-oxide) (58.6 mg, 0.500 mmol, 2.00 equiv.) was added and the reaction mixture was stirred 2 h. Then the resulting mixture was filtered and concentrated *in vacuo*. The residue was purified by preparative TLC, eluting with *n*-hexane/EtOAc 50:1 (v/v) to afford 69.5 mg 5-trifluoromethoxypentyl 4-formylbenzoate (**14**) as a white solid (91% yield).

R_f = 0.1 (*n*-hexane/EtOAc 30:1 (v/v)). NMR Spectroscopy: ^{1}H NMR (400 MHz, $CDCl_3$) δ 10.08 (s, 1H), 8.17 (d, *J* = 8.2 Hz, 2H), 7.94 (d, *J* = 8.3 Hz, 2H), 4.36 (t, *J* = 6.5 Hz, 2H), 3.98 (t, *J* = 6.3 Hz, 2H), 1.87 – 1.72 (m, 4H), 1.62 – 1.51 (m, 2H). ^{13}C NMR (101 MHz, $CDCl_3$) δ

191. 8, 165.7, 139.2, 135.35, 130.2, 129.6, 121.8 (q, J = 253.8 Hz), 67.2 (q, J = 3.1 Hz), 65.3, 28.4, 28.2, 22.2. ^{19}F NMR (376 MHz, $CDCl_3$) δ -60.7 (s, 3F) Mass Spectrometry: HRMS-EI (*m/z*): Calcd for $C_{14}H_{14}F_3O_4$ $[M-H]^+$, 303.0839. Found, 303.0837.

5-Trifluoromethoxypentyl 4-ethynylbenzoate (15)

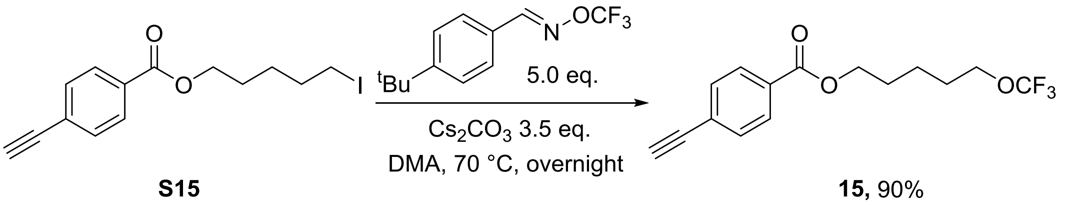

S15 **15**, 90%

In a N_2 glovebox, to 5-iodopentyl 4-ethynylbenzoate (**S15**) (85.5 mg, 0.250 mmol, 1.00 equiv.), (*E*)-*O*-trifluoromethyl-4-*tert*-butyl-benzaldoximes (**1a**) (307 mg, 1.25 mmol, 5.00 equiv.) in a 15.0 mL sealed vial were added DMA (2.00 mL). Cs_2CO_3 (285 mg, 0.875 mmol, 3.50 equiv.) was added to the reaction and the resulting mixture was stirred for overnight at 70 °C. After cooling to 50 °C, NMO (4-methylmorpholine *N*-oxide) (58.6 mg, 0.500 mmol, 2.00 equiv.) was added and the reaction mixture was stirred 2 h. Then the resulting mixture was filtered and concentrated *in vacuo*. The residue was purified by preparative TLC, eluting with *n*-hexane/EtOAc 20:1 (v/v) to afford 67.2 mg 5-trifluoromethoxypentyl 4-ethynylbenzoate (**15**) as a white solid (90% yield).

R_f = 0.2 (*n*-hexane/EtOAc 10:1 (v/v)). NMR Spectroscopy: 1H NMR (400 MHz, CDCl3) δ 7.99 (d, J = 8.5 Hz, 2H), 7.55 (d, J = 8.3 Hz, 2H), 4.33 (t, J = 6.5 Hz, 2H), 3.98 (t, J = 6.3 Hz, 2H), 3.23 (s, 1H), 1.90 – 1.68 (m, 4H), 1.67 – 1.46 (m, 2H). ^{13}C NMR (101 MHz, $CDCl_3$) δ 166.0, 132.2, 130.4, 129.6, 126.9, 121.8 (q, J = 254.3 Hz), 82.9, 80.2, 67.2 (q, J = 3.0 Hz), 65.0, 28.5, 28.3, 22.3. ^{19}F NMR (376 MHz, $CDCl_3$) δ -60.7 (s, 3F). Mass Spectrometry: HRMS-EI (*m/z*): Calcd for $C_{15}H_{15}F_3O_3$ [M], 300.0973. Found, 300.0969.

5-Trifluoromethoxypentyl 4-vinylbenzoate (16)

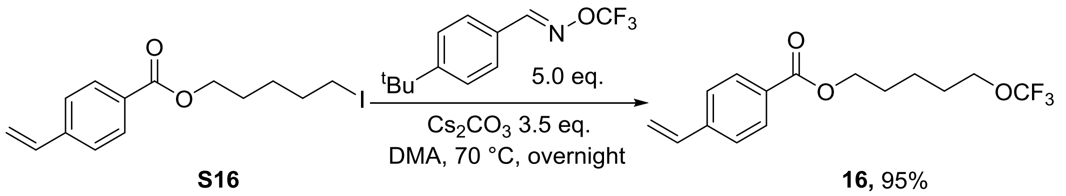

S16 **16**, 95%

In a N_2 glovebox, to 5-iodopentyl 4-vinylbenzoate (**S16**) (68.0 mg, 0.198 mmol, 1.00 equiv.), (*E*)-*O*-trifluoromethyl-4-*tert*-butyl-benzaldoximes (**1a**) (242 mg, 0.988 mmol, 5.00 equiv.) in a 15.0 mL sealed vial were added DMA (2.00 mL). Cs_2CO_3 (225 mg, 0.691 mmol, 3.50 equiv.) was added to the reaction and the resulting mixture was stirred for overnight at 70 °C. After cooling to 50 °C, NMO (4-methylmorpholine *N*-oxide) (46.4 mg, 0.396 mmol, 2.00 equiv.) was added and the reaction mixture was stirred 2 h. Then the resulting mixture was filtered and concentrated *in vacuo*. The residue was purified by preparative TLC, eluting with *n*-hexane/EtOAc 90:1 (v/v) to afford 56.9 mg 5-trifluoromethoxypentyl 4-vinylbenzoate (**16**) as a colorless liquid (95% yield).

R_f = 0.3 (*n*-hexane/EtOAc 40:1 (v/v)). NMR Spectroscopy: ^{1}H NMR (400 MHz, $CDCl_3$) δ 7.99 (d, *J* = 8.3 Hz, 2H), 7.46 (d, *J* = 8.3 Hz, 2H), 6.75 (dd, *J* = 17.6, 10.9 Hz, 1H), 5.86 (d, *J* = 17.6 Hz, 1H), 5.38 (d, *J* = 10.9 Hz, 1H), 4.33 (t, *J* = 6.5 Hz, 2H), 3.98 (t, *J* = 6.4 Hz, 2H), 1.87 – 1.70 (m, 4H), 1.63 – 1.49 (m, 2H). ^{13}C NMR (101 MHz, $CDCl_3$) δ 166.4, 142.0, 136.1, 129.9, 129.5, 126.2, 121.8 (q, *J* = 253.8 Hz), 116.6, 67.3 (q, *J* = 3.0 Hz), 64.6, 28.5, 28.3, 22.3. ^{19}F NMR (376 MHz, DMSO) δ -61.2 (s, 3F). Mass Spectrometry: HRMS-EI (*m/z*): Calcd for $C_{15}H_{17}F_3O_3$ [M], 302.1130. Found, 302.1125.

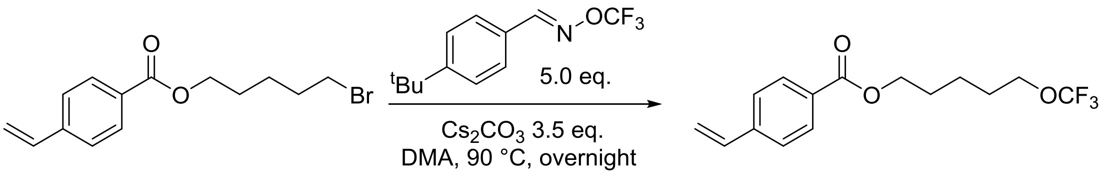

S16-2 **16,** 95%

In a N_2 glovebox, to 5-bromopentyl 4-vinylbenzoate (**S16-2**) (74.3 mg, 0.250 mmol, 1.00 equiv.), (*E*)-*O*-trifluoromethyl-4-*tert*-butyl-benzaldoximes (**1a**) (307 mg, 1.25 mmol, 5.00 equiv.) in a 15.0 mL sealed vial were added DMA (2.00 mL). Cs_2CO_3 (285 mg, 0.875 mmol, 3.50 equiv.) was added to the reaction and the resulting mixture was stirred for overnight at 90 °C. After cooling to 70 °C, NMO (4-methylmorpholine *N*-oxide) (58.6 mg, 0.500 mmol, 2.00 equiv.) was added and the reaction mixture was stirred 2 h. Then the resulting mixture was filtered and concentrated *in vacuo*. The residue was purified by preparative TLC, eluting with *n*-hexane/EtOAc 90:1 (v/v) to afford 71.8 mg 5-trifluoromethoxypentyl 4-vinylbenzoate (**16**) as a colorless liquid (95% yield).

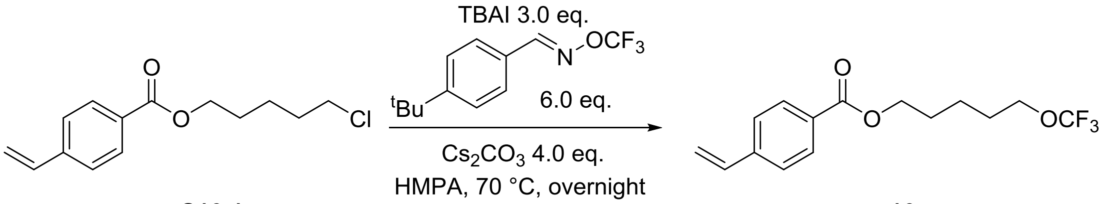

S16-1 **16,** 69%

In a N_2 glovebox, to 5-chloropentyl 4-vinylbenzoate (**S16-1**) (63.2 mg, 0.250 mmol, 1.00 equiv.), TBAI (tetrabutylammonium iodide) (277 mg, 0.750 mmol, 3.00 equiv.), (*E*)-*O*-trifluoromethyl-4-*tert*-butyl-benzaldoximes (**1a**) (368 mg, 1.50 mmol, 6.00 equiv.) in a 2.0 mL sealed vial were added HMPA (0.400 mL). Cs_2CO_3 (326 mg, 1.00 mmol, 4.00 equiv.) was added to the reaction and the resulting mixture was stirred for overnight at 70 °C. NaI (74.9 mg, 0.500 mmol, 2.00 equiv.) was added and the reaction mixture was stirred 3 h. After cooling to room temperature, the resulting mixture was filtered through a pad of silica gel and concentrated *in vacuo*. NMO (4-methylmorpholine *N*-oxide) (58.6 mg, 0.500 mmol, 2.00 equiv.) was added and the reaction mixture was stirred 2 h at 50 °C. Then the resulting mixture was filtered and concentrated *in vacuo*. The residue was purified by preparative TLC, eluting with *n*-hexane/EtOAc 90:1 (v/v) to afford 52.0 mg 5-trifluoromethoxypentyl 4-vinylbenzoate (**16**) as a colorless liquid (69% yield).

5-Trifluoromethoxypentyl nicotinate (17)

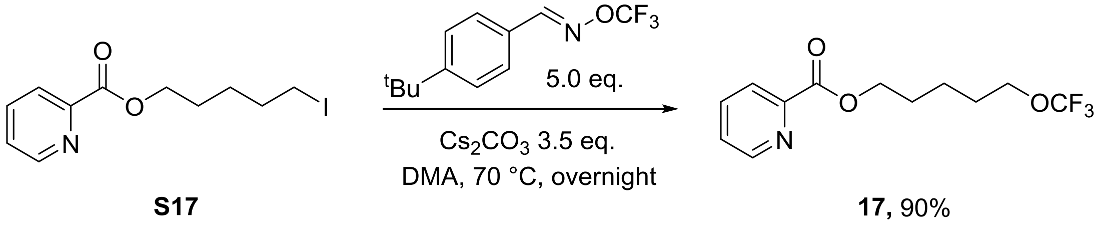

In a N_2 glovebox, to 5-iodopentyl nicotinate (**S17**) (79.8 mg, 0.250 mmol, 1.00 equiv.), (*E*)-*O*-trifluoromethyl-4-*tert*-butyl-benzaldoximes (**1a**) (307 mg, 1.25 mmol, 5.00 equiv.) in a 15.0 mL sealed vial were added DMA (2.00 mL). Cs_2CO_3 (285 mg, 0.875 mmol, 3.50 equiv.) was added to the reaction and the resulting mixture was stirred for overnight at 70 °C. After cooling to 50 °C, NMO (4-methylmorpholine *N*-oxide) (58.6 mg, 0.500 mmol, 2.00 equiv.) was added and the reaction mixture was stirred 2 h. Then the resulting mixture was filtered and concentrated *in vacuo*. The residue was purified by preparative TLC, eluting with *n*-hexane/EtOAc 20:1 (v/v) to afford 62.5 mg 5-trifluoromethoxypentyl nicotinate (**17**) as a white solid (90% yield).

R_f = 0.1 (*n*-hexane/EtOAc 10:1 (v/v)). NMR Spectroscopy: ^{1}H NMR (400 MHz, $CDCl_3$) δ 8.84 – 8.70 (m, 1H), 8.18 – 8.06 (m, 1H), 7.85 (t, *J* = 7.7 Hz, 1H), 7.54 – 7.43 (m, 1H), 4.44 (t, *J* = 6.7 Hz, 2H), 3.98 (t, *J* = 6.4 Hz, 2H), 1.93 – 1.82 (m, 2H), 1.82 – 1.70 (m, 2H), 1.63 – 1.49 (m, 2H). ^{13}C NMR (101 MHz, $CDCl_3$) δ 165.4, 150.1, 148.3, 137.2, 127.1, 125.3, 121. 8 (q, *J* = 253.8 Hz), 67.2 (q, *J* = 3.1 Hz), 65.7, 28.5, 28.3, 22.2. ^{19}F NMR (376 MHz, $CDCl_3$) δ -60.7 (s, 3F). Mass Spectrometry: HRMS-ESI (*m/z*): Calcd for $C_{12}H_{14}F_3NNaO_3$ $[M+Na]^+$, 300.0818. Found, 300. 0819.

5-Trifluoromethoxypentyl thiophene-3-carboxylate (18)

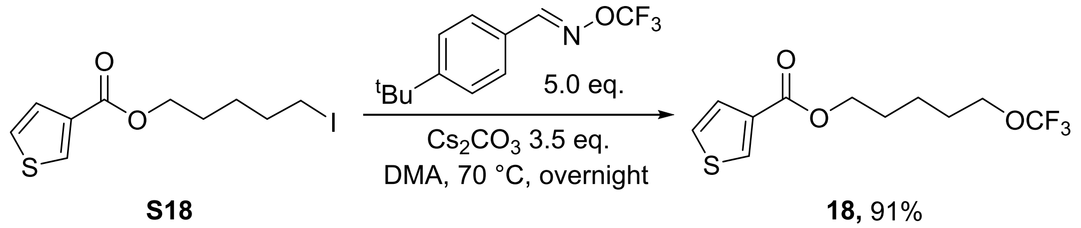

In a N_2 glovebox, to 5-iodopentyl thiophene-3-carboxylate (**S18**) (81.0 mg, 0.250 mmol, 1.00 equiv.), (*E*)-*O*-trifluoromethyl-4-*tert*-butyl-benzaldoximes (**1a**) (307 mg, 1.25 mmol, 5.00 equiv.) in a 15.0 mL sealed vial were added DMA (2.00 mL). Cs_2CO_3 (285 mg, 0.875 mmol, 3.50 equiv.) was added to the reaction and the resulting mixture was stirred for overnight at 70 °C. After cooling to 50 °C, NMO (4-methylmorpholine *N*-oxide) (58.6 mg, 0.500 mmol, 2.00 equiv.) was added and the reaction mixture was stirred 2 h. Then the resulting mixture was filtered and concentrated *in vacuo*. The residue was purified by preparative TLC, eluting with *n*-hexane/EtOAc 60:1 (v/v) to afford 64.3 mg 5-trifluoromethoxypentyl thiophene-3-carboxylate (**18**) as a colorless liquid (91% yield).

R_f = 0.4 (*n*-hexane/EtOAc 20:1 (v/v)). NMR Spectroscopy: ^{1}H NMR (400 MHz, $CDCl_3$) δ

8.14 – 8.06 (m, 1H), 7.56 – 7.48 (m, 1H), 7.36 – 7.28 (m, 1H), 4.29 (t, *J* = 6.5 Hz, 2H), 3.98 (t, *J* = 6.4 Hz, 2H), 1.87-1.71 (m, 4H), 1.59-1.47 (m, 2H).^{13}C NMR (101 MHz, $CDCl_3$) δ 162.9, 133.9, 132.7, 128.0, 126.1, 121.8 (q, *J* = 254.0 Hz), 67.3 (d, *J* = 2.9 Hz), 64.4, 28.5, 28.3, 22.2. ^{19}F NMR (376 MHz, $CDCl_3$) δ -60.4 (s, 3F). Mass Spectrometry: HRMS-ESI (*m/z*): Calcd for $C_{11}H_{13}F_3NaO_3S$ $[M+Na]^+$, 305.0430. Found, 305.0435。

2-(4-Trifluoromethoxybutoxy)naphthalene (19)

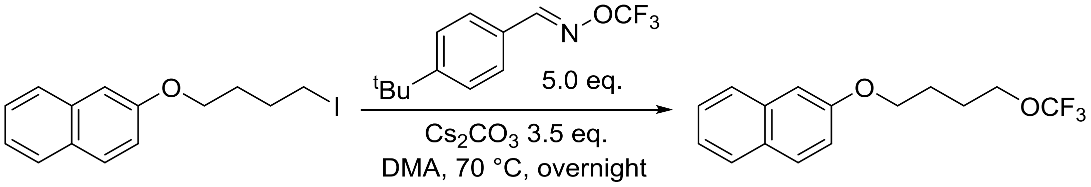

S19 **19,** 93%

In a N_2 glovebox, to 2-(4-iodobutoxy)-naphthalene (**S19**) (81.5 mg, 0.250 mmol, 1.00 equiv.), (*E*)-*O*-trifluoromethyl-4-*tert*-butyl-benzaldoximes (**1a**) (307 mg, 1.25 mmol, 5.00 equiv.) in a 15.0 mL sealed vial were added DMA (2.00 mL). Cs_2CO_3 (285 mg, 0.875 mmol, 3.50 equiv.) was added to the reaction and the resulting mixture was stirred for overnight at 70 °C. After cooling to 50 °C, NMO (4-methylmorpholine *N*-oxide) (58.6 mg, 0.500 mmol, 2.00 equiv.) was added and the reaction mixture was stirred 2 h. Then the resulting mixture was filtered and concentrated *in vacuo*. The residue was purified by preparative TLC, eluting with *n*-hexane/EtOAc 80:1 (v/v) to afford 66.0 mg 2-(4-trifluoromethoxybutoxy)-naphthalene (**19**) as a white solid (93% yield).

R_f = 0.4 (*n*-hexane/EtOAc 30:1 (v/v)). NMR Spectroscopy: ^{1}H NMR (400 MHz, $CDCl_3$) δ 7.86 – 7.72 (m, 3H), 7.54 – 7.44 (m, 1H), 7.43 – 7.34 (m, 1H), 7.22 – 7.12 (m, 2H), 4.16 – 4.06 (m, 4H), 2.05 – 1.91 (m, 4H).^{13}C NMR (101 MHz, $CDCl_3$) δ 156.9, 134.7, 129.6, 129.1, 127.8, 126.8, 126.5, 123.8, 121.9 (q, *J* = 254.9 Hz), 119.0, 106.6, 67.2 (q, *J* = 3.1 Hz), 67.1, 25.8, 25.5. ^{19}F NMR (376 MHz, $CDCl_3$) δ -60.8 (s, 3F). Mass Spectrometry: HRMS-EI (*m/z*): Calcd for $C_{15}H_{15}F_3O_2$ [M], 284.1024. Found, 284.1016.

7-(4-Trifluoromethoxybutoxy)-4-methyl-2H-chromen-2-one (20)

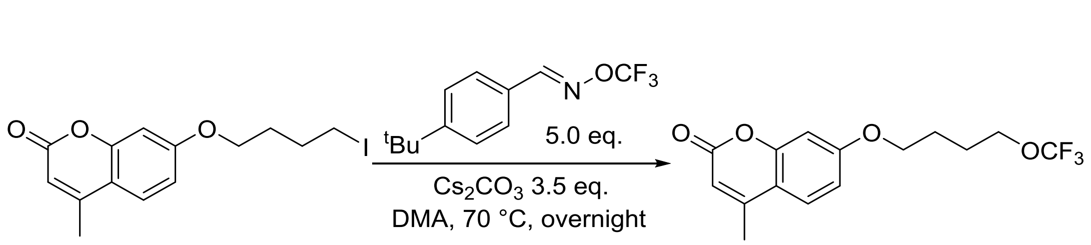

S20 **20,** 92%

In a N_2 glovebox, to 7-(4-iodobutoxy)-4-methyl-2H-chromen-2-one (**S20**) (89.54 mg, 0.250 mmol, 1.00 equiv.), (*E*)-*O*-trifluoromethyl-4-*tert*-butyl-benzaldoximes (**1a**) (307 mg, 1.25 mmol, 5.00 equiv.) in a 15.0 mL sealed vial were added DMA (2.00 mL). Cs_2CO_3 (285 mg, 0.875 mmol, 3.50 equiv.) was added to the reaction and the resulting mixture was stirred for overnight at 70 °C. After cooling to 50 °C, NMO (4-methylmorpholine *N*-oxide) (58.6 mg,

0.500 mmol, 2.00 equiv.) was added and the reaction mixture was stirred 2 h. Then the resulting mixture was filtered and concentrated *in vacuo*. The residue was purified by preparative TLC, eluting with *n*-hexane/EtOAc 6:1 (v/v) to afford 72.5 mg 7-(4-trifluormethoxybutoxy)-4-methyl-2H-chromen-2-one (**20**) as a white solid (92% yield).

R_f = 0.2 (*n*-hexane/EtOAc 4:1 (v/v)). NMR Spectroscopy: ^{1}H NMR (400 MHz, $CDCl_3$) δ 7.48 (d, *J* = 8.8 Hz, 1H), 6.83 (dd, *J* = 8.8, 2.4 Hz, 1H), 6.79 – 6.73 (m, 1H), 6.11 (s, 1H), 4.04 (t, *J* = 5.7 Hz, 4H), 2.38 (s, 1H), 2.01 – 1.82 (m, 4H).^{13}C NMR (101 MHz, $CDCl_3$) δ 161.9, 161.4, 155.3, 152.7, 125.7, 121.7 (q, *J* = 254.1 Hz), 113.7, 112.6, 112.0, 101.4, 67.7, 67.1 (q, *J* = 3.2 Hz), 25.6, 25.3, 18.8. ^{19}F NMR (376 MHz, $CDCl_3$) δ -60.7 (s, 3F). Mass Spectrometry: HRMS-ESI (*m/z*): Calcd for $C_{15}H_{15}F_3NaO_4$ $[M+Na]^+$, 339.0815. Found, 339. 0818.

_N_-(5-Trifluoromethoxypentyl)saccharine (21)

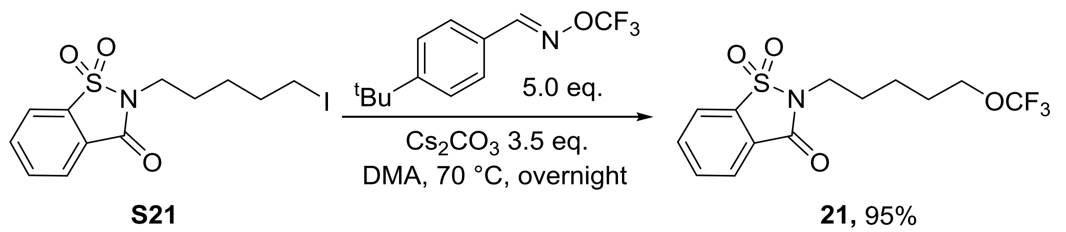

In a N_2 glovebox, to *N*-(5-iodopentyl)saccharine (**S21**) (94.8 mg, 0.250 mmol, 1.00 equiv.), (*E*)-*O*-trifluoromethyl-4-*tert*-butyl-benzaldoximes (**1a**) (307 mg, 1.25 mmol, 5.00 equiv.) in a 15.0 mL sealed vial were added DMA (2.00 mL). Cs_2CO_3 (285 mg, 0.875 mmol, 3.50 equiv.) was added to the reaction and the resulting mixture was stirred for overnight at 70 °C. After cooling to 50 °C, NMO (4-methylmorpholine *N*-oxide) (58.6 mg, 0.500 mmol, 2.00 equiv.) was added and the reaction mixture was stirred 2 h. Then the resulting mixture was filtered and concentrated *in vacuo*. The residue was purified by preparative TLC, eluting with *n*-hexane/EtOAc 6:1 (v/v) to afford 80.0 mg *N*-(5-trifluoromethoxypentyl)saccharine (**21**) as a white solid (95% yield).

R_f = 0.2 (*n*-hexane/EtOAc 4:1 (v/v)). NMR Spectroscopy: ^{1}H NMR (400 MHz, $CDCl_3$) δ 8.09 – 8.02 (m, 2H), 7.95 – 7.79 (m, 3H), 3.96 (t, *J* = 6.4 Hz, 2H), 3.79 (t, *J* = 7.4 Hz, 2H), 1.95 – 1.83 (m, 2H), 1.82 – 1.70 (m, 2H), 1.59 – 1.46 (m, 2H).^{13}C NMR (101 MHz, $CDCl_3$) δ 159.1, 137.7, 134.9, 134.4, 127.4, 125.2, 121.7 (q, *J* = 253.8 Hz), 121.0, 67.2 (q, *J* = 3.1 Hz), 39.1, 28.2, 27.9, 22.8. ^{19}F NMR (376 MHz, $CDCl_3$) δ -60.7 (s, 3F). Mass Spectrometry: HRMS-ESI (*m/z*): Calcd for $C_{13}H_{14}F_3NNaO_4S$ $[M+Na]^+$, 360.0488. Found, 360.0489.

2-(4-Trifluoromethoxybutyl)isoindoline-1,3-dione (22)

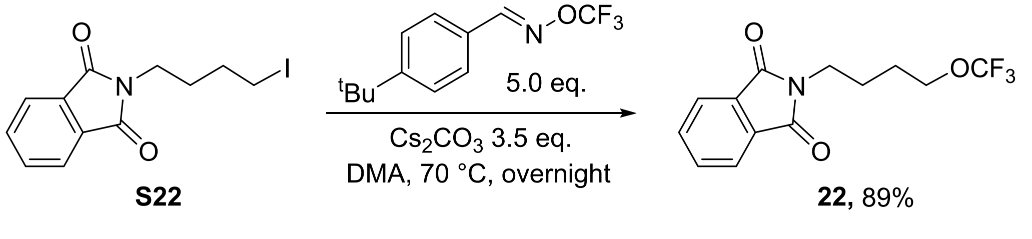

In a N_2 glovebox, to 2-(4-iodobutyl)isoindoline-1,3-dione (**S22**) (82.3 mg, 0.250 mmol, 1.00 equiv.), (*E*)-*O*-trifluoromethyl-4-*tert*-butyl-benzaldoximes (**1a**) (307 mg, 1.25 mmol, 5.00 equiv.) in a 15.0 mL sealed vial were added DMA (2.00 mL). Cs_2CO_3 (285 mg, 0.875 mmol, 3.50 equiv.) was added to the reaction and the resulting mixture was stirred for overnight at 70 °C. After cooling to 50 °C, NMO (4-methylmorpholine *N*-oxide) (58.6 mg, 0.500 mmol, 2.00 equiv.) was added and the reaction mixture was stirred 2 h. Then the resulting mixture was filtered and concentrated *in vacuo*. The residue was purified by preparative TLC, eluting with *n*-hexane/EtOAc 6:1 (v/v) to afford 64.1 mg 2-(4-trifluoromethoxybutyl)isoindoline-1,3-dione (**22**) as a white solid (89% yield).

R_f = 0.3 (*n*-hexane/EtOAc 4:1 (v/v)). NMR Spectroscopy: ^{1}H NMR (400 MHz, $CDCl_3$) δ 7.88 – 7.79 (m, 2H), 7.75 – 7.67 (m, 2H), 3.99 (t, *J* = 5.9 Hz, 2H), 3.73 (t, *J* = 6.6 Hz, 2H), 1.89 – 1.66 (m, 4H). ^{13}C NMR (101 MHz, $CDCl_3$) δ 168.5, 134.2, 132.2, 123.4, 121.7 (q, *J* = 254.1 Hz), 66.8 (q, *J* = 3.2 Hz), 37.3, 26.2, 24.9. ^{19}F NMR (376 MHz, $CDCl_3$) δ -60.7 (s, 3F). Mass Spectrometry: HRMS-ESI (*m/z*): Calcd for $C_{13}H_{12}F_3NNaO_3$ $[M+Na]^+$, 310.0661. Found, 310.0664

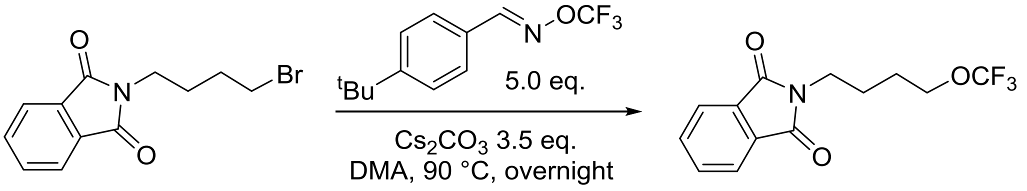

S22-2 **22,** 97%

In a N_2 glovebox, to 2-(4-bromobutyl)isoindoline-1,3-dione (**S22-2**) (70.5 mg, 0.250 mmol, 1.00 equiv.), (*E*)-*O*-trifluoromethyl-4-*tert*-butyl-benzaldoximes (**1a**) (307 mg, 1.25 mmol, 5.00 equiv.) in a 15.0 mL sealed vial were added DMA (2.00 mL). Cs_2CO_3 (285 mg, 0.875 mmol, 3.50 equiv.) was added to the reaction and the resulting mixture was stirred for overnight at 90 °C. After cooling to 70 °C, NMO (4-methylmorpholine *N*-oxide) (58.6 mg, 0.500 mmol, 2.00 equiv.) was added and the reaction mixture was stirred 2 h. Then the resulting mixture was filtered and concentrated *in vacuo*. The residue was purified by preparative TLC, eluting with *n*-hexane/EtOAc 6:1 (v/v) to afford 69.8 mg 2-(4-trifluoromethoxybutyl)isoindoline-1,3-dione (**22**) as a white solid (97% yield).

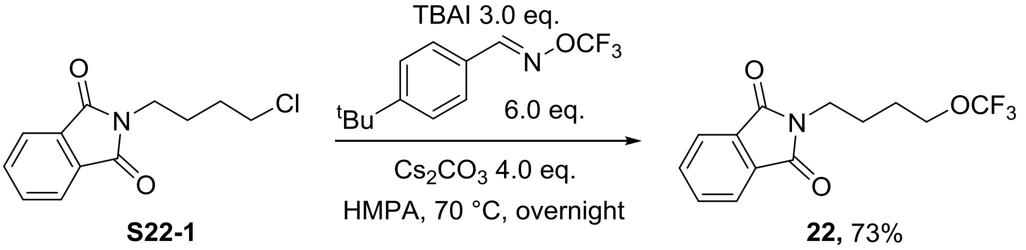

S22-1 **22,** 73%

In a N_2 glovebox, to 2-(4-chlorobutyl)isoindoline-1,3-dione (**S22-1**) (59.4 mg, 0.250 mmol, 1.00 equiv.), TBAI (tetrabutylammonium iodide) (277 mg, 0.750 mmol, 3.00 equiv.), (*E*)-*O*-trifluoromethyl-4-*tert*-butyl-benzaldoximes (**1a**) (368 mg, 1.50 mmol, 6.00 equiv.) in a 2.0 mL sealed vial were added HMPA (0.400 mL). Cs_2CO_3 (326 mg, 1.00 mmol, 4.00 equiv.) was added to the reaction and the resulting mixture was stirred for overnight at 70 °C. NaI

(74.9 mg, 0.500 mmol, 2.00 equiv.) was added and the reaction mixture was stirred 3 h. After cooling to room temperature, the resulting mixture was filtered through a pad of silica gel and concentrated *in vacuo*. NMO (4-methylmorpholine *N*-oxide) (58.6 mg, 0.500 mmol, 2.00 equiv.) was added and the reaction mixture was stirred 2 h at 50 °C. Then the resulting mixture was filtered and concentrated *in vacuo*. The residue was purified by preparative TLC, eluting with *n*-hexane/EtOAc 6:1 (v/v) to afford 52.5 mg 2-(4-trifluoromethoxybutyl)isoindoline-1,3-dione (**22**) as a white solid (73% yield).

1-Methyl-3-(3-(trifluoromethoxy)propyl)-1H-indole (23)

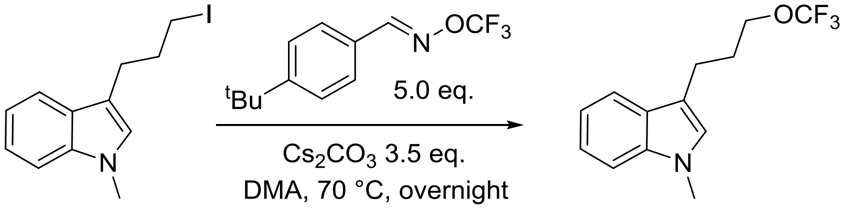

S23 **23**, 93%

In a N_2 glovebox, to 1-methyl-3-(3-iodopropyl)-1H-indole (**S23**) (74.8 mg, 0.250 mmol, 1.00 equiv.), (*E*)-*O*-trifluoromethyl-4-*tert*-butyl-benzaldoximes (**1a**) (307 mg, 1.25 mmol, 5.00 equiv.) in a 15.0 mL sealed vial were added DMA (2.00 mL). Cs_2CO_3 (285 mg, 0.875 mmol, 3.50 equiv.) was added to the reaction and the resulting mixture was stirred for overnight at 70 °C. After cooling to 50 °C, NMO (4-methylmorpholine *N*-oxide) (58.6 mg, 0.500 mmol, 2.00 equiv.) was added and the reaction mixture was stirred 2 h. The reaction mixture was filtered and concentrated *in vacuo*. The residue was purified by preparative TLC, eluting with *n*-hexane/DCM 100:1 (v/v) to afford 59.5 mg 1-methyl-3-(3-(trifluoromethoxy)propyl) -1H-indole (**23**) as a colorless liquid (93% yield).

R_f = 0.4 (*n*-hexane/EtOAc 20:1 (v/v)). NMR Spectroscopy: ^{1}H NMR (400 MHz, $CDCl_3$) δ 7.60 – 7.53 (m, 1H), 7.32 – 7.26 (m, 1H), 7.26 – 7.19 (m, 1H), 7.14 – 7.07 (m, 1H), 6.84 (s, 1H), 3.99 (t, J = 6.3 Hz, 2H), 3.74 (s, 3H), 2.87 (t, J = 7.3 Hz, 2H), 2.13 – 2.01 (m, 2H). ^{13}C NMR (101 MHz, $CDCl_3$) δ 137.3, 127.8, 126.6, 121.93 (q, J = 253.8 Hz), 121.8, 119.0, 118.9, 113.3, 109.4, 66.9 (q, J = 2.9 Hz), 32.7, 29.4, 21.0. ^{19}F NMR (376 MHz, $CDCl_3$) δ -60.5 (s, 3F). Mass Spectrometry: HRMS-ESI (*m/z*): Calcd for $C_{13}H_{15}F_3NO$ $[M+H]^+$, 258.1100. Found, 258.1101.

11-(Trifluoromethoxy)undec-1-ene (24)

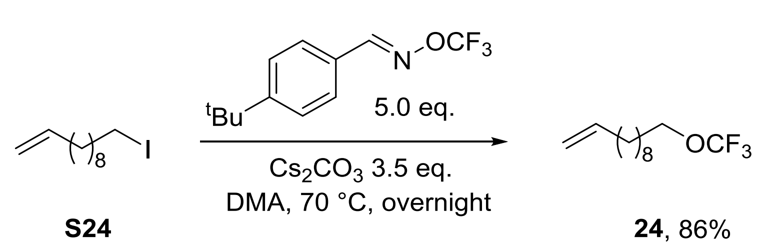

S24 **24**, 86%

In a N_2 glovebox, to 11-iodoundec-1-ene (**S24**) (70.1 mg, 0.250 mmol, 1.00 equiv.), (*E*)-*O*-trifluoromethyl-4-*tert*-butyl-benzaldoximes (**1a**) (307 mg, 1.25 mmol, 5.00 equiv.) in a

15.0 mL sealed vial were added DMA (2.00 mL). Cs_2CO_3 (285 mg, 0.875 mmol, 3.50 equiv.) was added to the reaction and the resulting mixture was stirred for overnight at 70 °C. After cooling to 50 °C, NMO (4-methylmorpholine *N*-oxide) (58.6 mg, 0.500 mmol, 2.00 equiv.) was added and the reaction mixture was stirred 2 h. Then the resulting mixture was filtered and concentrated *in vacuo*. The residue was purified by chromatography on silica gel, eluting with *n*-hexane to afford 51.4 mg 11-(trifluoromethoxy)undec-1-ene (**24**) as a colorless liquid (86% yield).

R_f = 0.8 (*n*-hexane). NMR Spectroscopy: ^{1}H NMR (400 MHz, $CDCl_3$) δ 5.89 – 5.73 (m, 1H), 5.05 – 4.88 (m, 2H), 3.94 (t, *J* = 6.6 Hz, 2H), 2.04 (m, 2H), 1.74 – 1.61 (m, 2H), 1.44 – 1.25 (m, 12H). ^{13}C NMR (101 MHz, $CDCl_3$) δ 139.3, 121.9 (q, *J* = 253.6 Hz), 114.3, 67.7 (q, *J* = 3.0 Hz), 33.9, 29.5, 29.5, 29.2, 29.2, 29.0, 28.8, 25.6. ^{19}F NMR (376 MHz, $CDCl_3$) δ -60.5 (s, 3F). **24** is a known compound and spectral data match the reported literature values.[7]

N-Boc-3-(2-trifluoromethoxyethyl)azetidine (25)

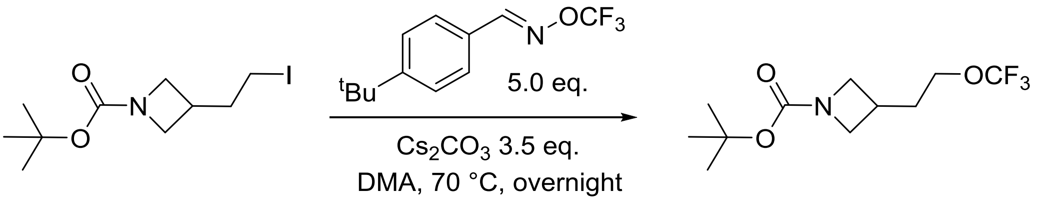

S25 **25**, 86%

In a N_2 glovebox, to N-Boc-3-(2-iodoethyl)azetidine (**S25**) (77.8 mg, 0.250 mmol, 1.00 equiv.), (*E*)-*O*-trifluoromethyl-4-*tert*-butyl-benzaldoximes (**1a**) (307 mg, 1.25 mmol, 5.00 equiv.) in a 15.0 mL sealed vial were added DMA (2.00 mL). Cs_2CO_3 (285 mg, 0.875 mmol, 3.50 equiv.) was added to the reaction and the resulting mixture was stirred for overnight at 70 °C. After cooling to 50 °C, NMO (4-methylmorpholine *N*-oxide) (58.6 mg, 0.500 mmol, 2.00 equiv.) was added and the reaction mixture was stirred 2 h. Then the resulting mixture was filtered and concentrated *in vacuo*. The residue was purified by preparative TLC, eluting with *n*-hexane/EtOAc 10:1 (v/v) to afford 58.1 mg N-Boc-3-(2-trifluoromethoxyethyl) azetidine (**25**) as a colorless liquid (86% yield).

R_f = 0.2 (*n*-hexane/EtOAc 10:1 (v/v)). NMR Spectroscopy: ^{1}H NMR (400 MHz, $CDCl_3$) δ 4.04 (t, *J* = 8.4 Hz, 2H), 3.94 (t, *J* = 6.2 Hz, 2H), 3.59 (dd, *J* = 8.7, 5.6 Hz, 2H), 2.75 – 2.54 (m, 1H), 1.98 (q, *J* = 6.6 Hz, 2H), 1.42 (s, 9H). ^{13}C NMR (101 MHz, $CDCl_3$) δ 156.4, 121.7 (q, *J* = 254.3 Hz), 79.5, 65.5 (q, *J* = 3.2 Hz), 33.3, 28.5, 26.0. ^{19}F NMR (376 MHz, $CDCl_3$) δ -60.8 (s, 3F). Mass Spectrometry: HRMS-EI (*m/z*): Calcd for $C_{11}H_{18}F_3NO_3$ [M], 269.1239. Found, 269.1231.

7-(Trifluoromethoxy)heptanenitrile (26)

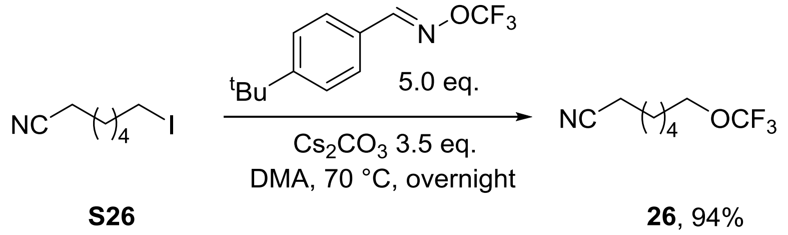

In a N_2 glovebox, to 7-iodoheptanenitrile (**S26**) (74.2 mg, 0.313 mmol, 1.00 equiv.), (*E*)-*O*-trifluoromethyl-4-*tert*-butyl-benzaldoximes (**1a**) (384 mg, 1.56 mmol, 5.00 equiv.) in a 15.0 mL sealed vial were added DMA (2.50 mL). Cs_2CO_3 (357 mg, 1.10 mmol, 3.50 equiv.) was added to the reaction and the resulting mixture was stirred for overnight at 70 °C. After cooling to 50 °C, NMO (4-methylmorpholine *N*-oxide) (72.2 mg, 0.626 mmol, 2.00 equiv.) was added and the reaction mixture was stirred 2 h. Then the resulting mixture was filtered and concentrated *in vacuo*. The residue was purified by chromatography on silica gel, eluting with *n*-hexane/EtOAc 20:1 (v/v) to afford 57.4 mg 7-(trifluoromethoxy)heptanenitrile (**26**) as a colorless liquid (94% yield).

R_f = 0.2 (*n*-hexane/EtOAc 20:1 (v/v)). NMR Spectroscopy: ^{1}H NMR (400 MHz, $CDCl_3$) δ 3.95 (t, *J* = 6.4 Hz, 2H), 2.34 (t, *J* = 7.1 Hz, 2H), 1.78 – 1.60 (m, 4H), 1.55 – 1.36 (m, 4H). ^{13}C NMR (101 MHz, $CDCl_3$) δ 121.7 (q, *J* = 253.8 Hz), 119.7 (s), 67.2 (q, *J* = 3.1 Hz), 28.5, 28.2 , 25.3, 24.9, 17.1. ^{19}F NMR (376 MHz, $CDCl_3$) δ -60.6 (s, 3F). Mass Spectrometry: HRMS-EI (*m/z*): Calcd for $C_8H_{11}F_3NO^+$ $[M-H]^+$, 194.0787. Found, 194.0788.

2,6-Dimethyl-8-(trifluoromethoxy)oct-2-ene (27)

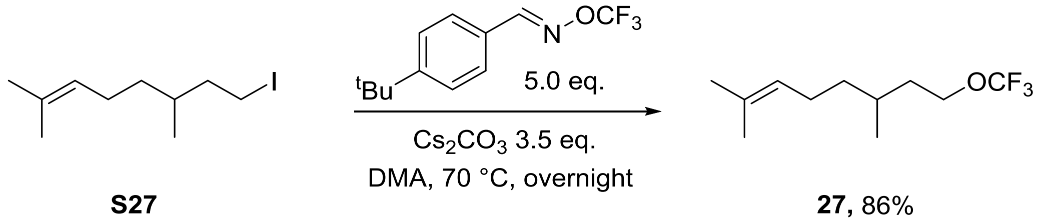

In a N_2 glovebox, to 8-iodo-2,6-dimethyloct-2-ene (**S27**) (66.5 mg, 0.250 mmol, 1.00 equiv.), (*E*)-*O*-trifluoromethyl-4-*tert*-butyl-benzaldoximes (**1a**) (307 mg, 1.25 mmol, 5.00 equiv.) in a 15.0 mL sealed vial were added DMA (2.00 mL). Cs_2CO_3 (285 mg, 0.875 mmol, 3.50 equiv.) was added to the reaction and the resulting mixture was stirred for overnight at 70 °C. After cooling to 50 °C, NMO (4-methylmorpholine *N*-oxide) (58.6 mg, 0.500 mmol, 2.00 equiv.) was added and the reaction mixture was stirred 2 h. Then the resulting mixture was filtered and concentrated *in vacuo*. The residue was purified by chromatography on silica gel, eluting with *n*-hexane to afford 48.0 mg 2,6-dimethyl-8-(trifluoromethoxy)oct-2-ene (**27**) as a colorless liquid (86% yield).

R_f = 0.7 (*n*-hexane). NMR Spectroscopy: ^{1}H NMR (400 MHz, $CDCl_3$) δ 5.15 – 5.03 (m, 1H), 4.07 – 3.93 (m, 2H), 2.09 – 1.88 (m, 2H), 1.79 – 1.67 (m, 4H), 1.67 – 1.54 (m, 4H), 1.54 – 1.43 (m, 1H), 1.40 – 1.30 (m, 1H), 1.25 – 1.13 (m, 1H), 0.92 (d, *J* = 6.6 Hz, 3H). ^{13}C NMR

(101 MHz, $CDCl_3$) δ 131.5, 124.3, 121.7 (d, *J* = 253.5 Hz), 65.8 (q, *J* = 2.9 Hz), 36.8, 35.5, 28.9, 25.7, 25.3, 19.1, 17.6. ^{19}F NMR (376 MHz, DMSO) δ -61.2 (s, 3F). **27** is a known compound and spectral data match the reported literature values.[8]

1-(Trifluoromethoxy)hexadecane (28)

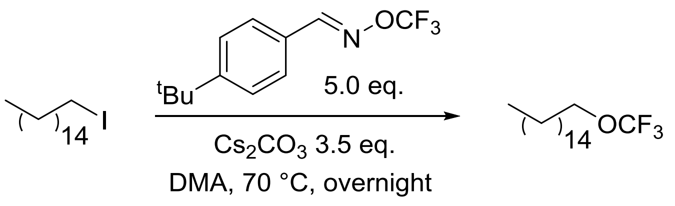

In a N_2 glovebox, to 1-iodohexadecane (**S28**) (88.1 mg, 0.250 mmol, 1.00 equiv.), (*E*)-*O*-trifluoromethyl-4-*tert*-butyl-benzaldoximes (**1a**) (307 mg, 1.25 mmol, 5.00 equiv.) in a 15.0 mL sealed vial were added DMA (2.00 mL). Cs_2CO_3 (285 mg, 0.875 mmol, 3.50 equiv.) was added to the reaction and the resulting mixture was stirred for overnight at 70 °C. After cooling to 50 °C, NMO (4-methylmorpholine *N*-oxide) (58.6 mg, 0.500 mmol, 2.00 equiv.) was added and the reaction mixture was stirred 2 h. Then the resulting mixture was filtered and concentrated *in vacuo*. The residue was purified by chromatography on silica gel, eluting with *n*-hexane to afford 78.0 mg 1-trifluoromethoxyhexadecane (**28**) as a colorless liquid (89% yield).

R_f = 0.9 (*n*-hexane). NMR Spectroscopy: ^{1}H NMR (400 MHz, $CDCl_3$) δ 3.97 (t, *J* = 6.5 Hz, 2H), 1.75 – 1.66 (m, 2H), 1.38 – 1.25 (m, 26H), 0.91 (t, *J* = 6.4 Hz, 3H). ^{13}C NMR (101 MHz, $CDCl_3$) δ 121.9 (q, *J* = 253.4 Hz), 67.7 (q, *J* = 2.9 Hz), 32.1, 29.9, 29.9, 29.8, 29.7, 29.7, 29.6, 29.3, 28.9, 25.6, 22.9, 14.3. ^{19}F NMR (376 MHz, $CDCl_3$) δ -60.7 (s, 3F). Mass Spectrometry: HRMS-EI (*m/z*): Calcd for $C_{17}H_{33}F_3O$ [M], 310.2484. Found, 310.2476.

11-Trifluoromethoxyundecyl 5-chloropentanoate (29)

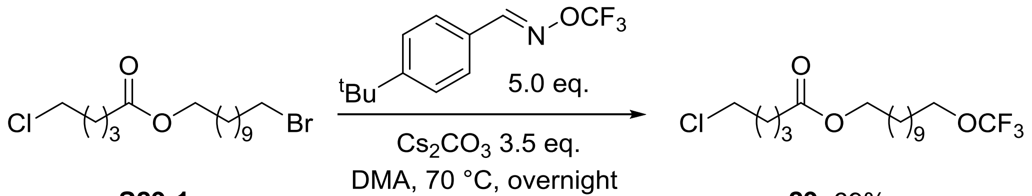

In a N_2 glovebox, to 11-bromoundecyl 5-chloropentanoate (**S29-1**) (92.4 mg, 0.250 mmol, 1.00 equiv.), (*E*)-*O*-trifluoromethyl-4-*tert*-butyl-benzaldoximes (**1a**) (307 mg, 1.25 mmol, 5.00 equiv.) in a 15.0 mL sealed vial were added DMA (2.00 mL). Cs_2CO_3 (285 mg, 0.875 mmol, 3.50 equiv.) was added to the reaction and the resulting mixture was stirred for overnight at 70 °C. NMO (4-methylmorpholine *N*-oxide) (58.6 mg, 0.500 mmol, 2.00 equiv.) was added and the reaction mixture was stirred 2 h. Then the resulting mixture was filtered and concentrated *in vacuo*. The residue was purified by chromatography on silica gel, eluting with *n*-hexane/EtOAc 40:1 (v/v) to afford 64.2 mg 11-trifluoromethoxyundecyl

5-chloropentanoate (**29**) as a colorless liquid (69% yield).

R_f = 0.2 (*n*-hexane/EtOAc 20:1). NMR Spectroscopy: ^{1}H NMR (400 MHz, $CDCl_3$) δ 4.08 (t, *J* = 6.7 Hz, 2H), 3.96 (t, *J* = 6.6 Hz, 2H), 3.56 (t, *J* = 6.2 Hz, 2H), 2.35 (t, *J* = 6.8 Hz, 2H), 1.89 – 1.57 (m, 8H), 1.32 (d, *J* = 18.6 Hz, 14H). ^{13}C NMR (101 MHz, $CDCl_3$) δ 173.2, 121.7 (d, *J* = 253.8 Hz), 67.5 (q, *J* = 2.7 Hz), 64.6, 44.4, 33.4, 31.9, 29.4, 29.4, 29.2, 29.0, 28.7, 28.6, 25.9, 25.4, 22.3. ^{19}F NMR (376 MHz, DMSO) δ -60.5 (s, 3F). Mass Spectrometry: HRMS-EI (*m/z*): Calcd for $C_{17}H_{30}ClF_3NaO_3$ $[M+Na]^+$, 397.1728. Found, 397.1733.

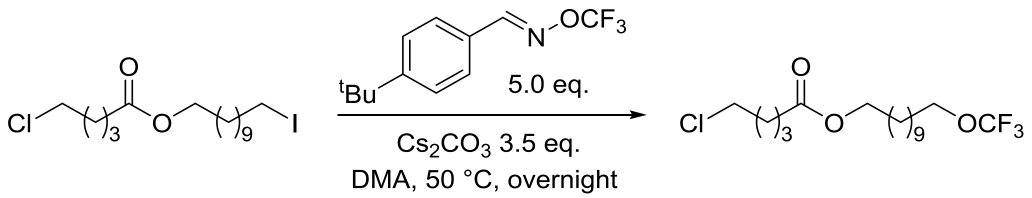

S29 **29**, 62%

In a N_2 glovebox, to 11-iodoundecyl 5-chloropentanoate (**S29**) (77.0 mg, 0.185 mmol, 1.00 equiv.), (*E*)-*O*-trifluoromethyl-4-*tert*-butyl-benzaldoximes (**1a**) (227 mg, 0.924 mmol, 5.00 equiv.) in a 15.0 mL sealed vial were added DMA (1.50 mL). Cs_2CO_3 (211 mg, 0.647 mmol, 3.50 equiv.) was added to the reaction and the resulting mixture was stirred for overnight at 50 °C. NMO (4-methylmorpholine *N*-oxide) (58.6 mg, 0.500 mmol, 2.00 equiv.) was added and the reaction mixture was stirred 2 h. Then the resulting mixture was filtered and concentrated *in vacuo*. The residue was purified by chromatography on silica gel, eluting with *n*-hexane/EtOAc 40:1 (v/v) to afford 42.6 mg 11-trifluoromethoxyundecyl 5-chloropentanoate (**29**) as a colorless liquid (62% yield).

(S)-*tert*-Butyl 2-(1,3-dioxoisoindolin-2-yl)-3-(4-((5-trifluoromethoxypentyl) oxy)phenyl)-propanoate (30)

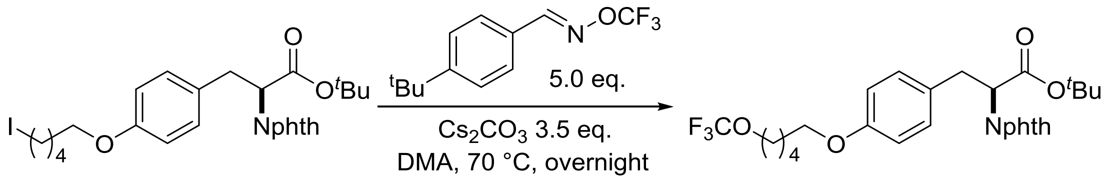

S30 **30**, 98%

In a N_2 glovebox, to (S)-*tert*-butyl 2-(1,3-dioxoisoindolin-2-yl)-3-(4-((5-iodopentyl)oxy) phenyl)propanoate (**S30**) (104 mg, 0.250 mmol, 1.00 equiv.), (*E*)-*O*-trifluoromethyl-4-*tert*-butyl-benzaldoximes (**1a**) (307 mg, 1.25 mmol, 5.00 equiv.) in a 15.0 mL sealed vial were added DMA (2.00 mL). Cs_2CO_3 (285 mg, 0.875 mmol, 3.50 equiv.) was added to the reaction and the resulting mixture was stirred for overnight at 70 °C. After cooling to 50 °C, NMO (4-methylmorpholine *N*-oxide) (58.6 mg, 0.500 mmol, 2.00 equiv.) was added and the reaction mixture was stirred 2 h. Then the resulting mixture was filtered and concentrated *in vacuo*. The residue was purified by preparative TLC, eluting with *n*-hexane/EtOAc 10:1 (v/v) to afford 92.0 mg 2-(4-trifluoromethoxybutyl)isoindoline-1,3-dione (**30**) as a white solid (98% yield).

R_f = 0.3 (*n*-hexane/EtOAc 8:1 (v/v)). NMR Spectroscopy: ^{1}H NMR (400 MHz, $CDCl_3$) δ 7.82

– 7.71 (m, 2H), 7.70 – 7.60 (m, 2H), 7.05 (d, J = 8.5 Hz, 2H), 6.69 (d, J = 8.5 Hz, 2H), 5.02 (dd, J = 9.8, 6.8 Hz, 1H), 3.94 (t, J = 6.4 Hz, 2H), 3.90 – 3.76 (m, 2H), 3.53 – 3.42 (m, 2H), 1.79 – 1.63 (m, 4H), 1.58 – 1.46 (m, 2H), 1.44 (s, 9H). ^{13}C NMR (101 MHz, $CDCl_3$) δ 167.9, 167.7, 157.7, 134.1, 131.7, 129.9, 129.1, 123.4, 121.7 (q, J = 253.7 Hz), 114.5, 82.7, 67.4, 67.3 (q, J = 3.2 Hz), 54.4, 33.9, 28.7, 28.5, 27.9, 22.2. ^{19}F NMR (376 MHz, DMSO) δ -60.9 (s, 3F). Mass Spectrometry: HRMS-ESI (*m/z*): Calcd for $C_{27}H_{31}F_3NO_6$ $[M+H]^+$, 522.2098. Found, 522. 2095.

(Trifluoromethoxy)cycloheptane[8] (31)

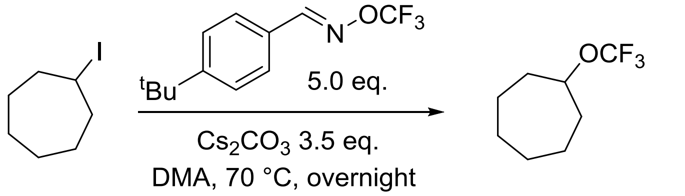

S31 **31,** 54%(^{19}F NMR)

In a N_2 glovebox, to cyclohetyl iodine (**S31**) (11.2 mg, 0.0500 mmol, 1.00 equiv.), (*E*)-*O*-trifluoromethyl-4-*tert*-butyl-benzaldoximes (**1a**) (61.3 mg, 0.250 mmol, 5.00 equiv.) in a 2.00 mL sealed vial were added DMA (0.400 mL). Cs_2CO_3 (57.0 mg, 0.175 mmol, 3.50 equiv.) was added to the reaction and the resulting mixture was stirred for overnight at 70 °C. The yield of (trifluoromethoxy)cycloheptane (**31**) was determined by comparing the integration of the ^{19}F NMR resonance of (trifluoromethoxy)cycloheptane (-60.7 ppm) with that of benzotrifluoride (-62.8 ppm). (^{19}F NMR yield 54% yield).

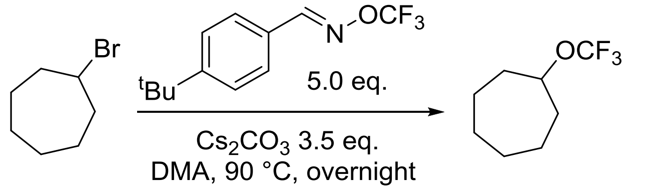

S31-1 **31,** 46%(^{19}F NMR)

In a N_2 glovebox, to cyclohetyl bromide (**S31-1**) (8.8 mg, 0.0500 mmol, 1.00 equiv.), (*E*)-*O*-trifluoromethyl-4-*tert*-butyl-benzaldoximes (**1a**) (61.3 mg, 0.250 mmol, 5.00 equiv.) in a 2.00 mL sealed vial were added DMA (0.400 mL). Cs_2CO_3 (57.0 mg, 0.175 mmol, 3.50 equiv.) was added to the reaction and the resulting mixture was stirred for overnight at 90 °C. After then, benzotrifluoride (6.0 μL, 0.0490 mmol) was added to the reaction mixture. The yield of (trifluoromethoxy)cycloheptane (**31**) was determined by comparing the integration of the ^{19}F NMR resonance of (trifluoromethoxy)cycloheptane (-60.7 ppm) with that of benzotrifluoride (-62.8 ppm). (^{19}F NMR yield 46% yield).

5-Trifluoromethoxyhexan-2-yl 4-fluorobenzoate (32)

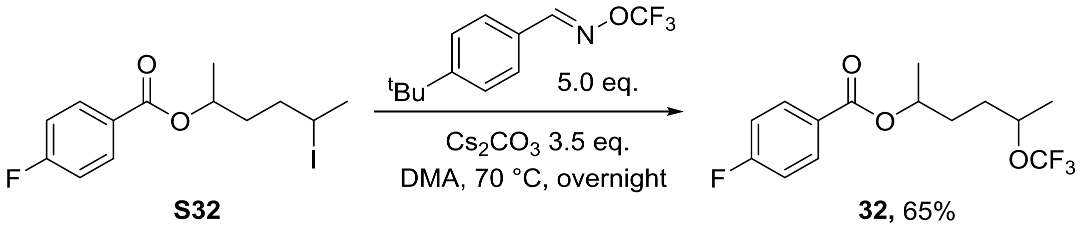

In a N_2 glovebox, to 5-iodohexan-2-yl 4-fluorobenzoate (**S32**) (75.0 mg, 0.214 mmol, 1.00 equiv.), (*E*)-*O*-trifluoromethyl-4-*tert*-butyl-benzaldoximes (**1a**) (263 mg, 1.07 mmol, 5.00 equiv.) in a 15.0 mL sealed vial were added DMA (1.70 mL). Cs_2CO_3 (244 mg, 0.750 mmol, 3.50 equiv.) was added to the reaction and the resulting mixture was stirred for overnight at 70 °C. Then, Cs_2CO_3 (138 mg, 0.424 mmol, 2.00 equiv.) was added to the reaction and the resulting mixture was stirred 2 h at 70 °C. After cooling to room temperature, the reaction mixture was filtered and concentrated *in vacuo*. The residue was purified by preparative TLC, eluting with *n*-hexane/EtOAc 100:1 (v/v) to afford 43.2 mg 5-trifluoromethoxyhexan-2-yl 4-fluorobenzoate (**32**) as a colorless liquid (65% yield).

R_f = 0.6 (*n*-hexane/EtOAc 20:1). NMR Spectroscopy: ^{1}H NMR (400 MHz, $CDCl_3$) δ 8.11 – 7.98 (m, 2H), 7.10 (t, *J* = 8.4 Hz, 2H), 5.14 (dd, *J* = 12.2, 6.1 Hz, 1H), 4.35 (dt, *J* = 11.9, 6.0 Hz, 1H), 1.96 – 1.57 (m, 4H), 1.47 – 1.23 (m, 6H). ^{13}C NMR (101 MHz, $CDCl_3$) δ 165.88 (d, *J* = 253.6 Hz), 165.31, 165.27, 132.18 (d, *J* = 9.3 Hz), 126.95, 121.87 (d, *J* = 253.5 Hz), 115.60 (d, *J* = 21.9 Hz), 76.23, 75.90, 71.53, 71.12, 32.62, 32.39, 31.70, 31.53, 20.99, 20.95, 20.23, 20.20. ^{19}F NMR (376 MHz, $CDCl_3$) δ -60.4 (s, 3F), -57.7 (s, 3F). Mass Spectrometry: HRMS-ESI (*m/z*): Calcd for $C_{14}H_{16}F_4NaO_3$ $[M+Na]^+$, 331.0928. Found, 331.0933.

3-Trifluoromethoxybutylbenzene (33)

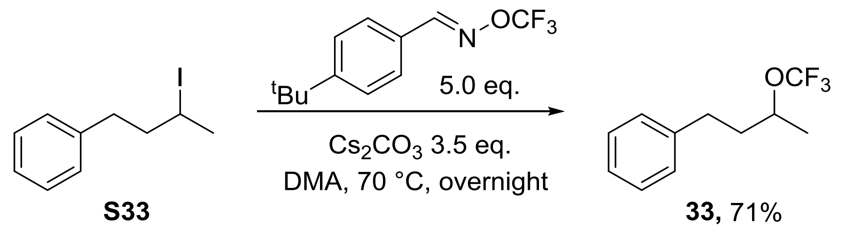

In a N_2 glovebox, to 3-iodobutylbenzene (**S33**) (65.0 mg, 0.250 mmol, 1.00 equiv.), (*E*)-*O*-trifluoromethyl-4-*tert*-butyl-benzaldoximes (**1a**) (307 mg, 1.25 mmol, 5.00 equiv.) in a 15.0 mL sealed vial were added DMA (2.00 mL). Cs_2CO_3 (285 mg, 0.875 mmol, 3.50 equiv.) was added to the reaction and the resulting mixture was stirred for overnight at 70 °C. Then, Cs_2CO_3 (138 mg, 0.424 mmol, 2.00 equiv.) was added to the reaction and the resulting mixture was stirred 2 h at 70 °C. After cooling to room temperature, the reaction mixture was filtered and concentrated *in vacuo*. The residue was purified by preparative TLC, eluting with *n*-hexane to afford 39.0 mg 3-trifluoromethoxybutylbenzene (**33**) as a colorless liquid (71% yield).

R_f = 0.6 (*n*-hexane). NMR Spectroscopy: ^{1}H NMR (400 MHz, $CDCl_3$) δ 7.34 – 7.27 (m, 2H), 7.21 (t, J = 8.6 Hz, 3H), 4.42 – 4.30 (m, 1H), 2.83 – 2.62 (m, 2H), 2.08 – 1.79 (m, 2H), 1.38 (d, J = 6.2 Hz, 3H). ^{13}C NMR (101 MHz, $CDCl_3$) δ 141.2, 128.7, 128.5, 126.2, 121.9 (q, J = 253.7 Hz), 75.8 (q, J = 1.8 Hz), 38.3, 31.4, 21.0 . ^{19}F NMR (376 MHz, $CDCl_3$) δ -57.6 (s, 3F). **33** is a known compound and spectral data match the reported literature values.[8]

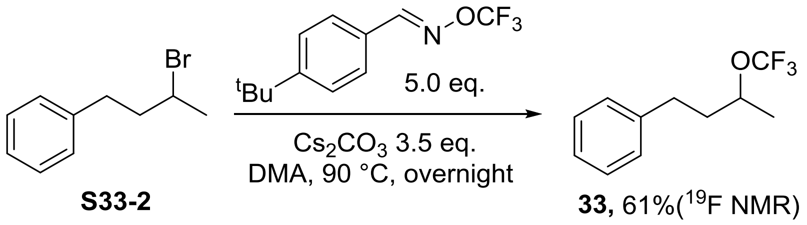

In a N_2 glovebox, to 3-iodobutylbenzene (**S33-2**) (53.3 mg, 0.250 mmol, 1.00 equiv.), (*E*)-*O*-trifluoromethyl-4-*tert*-butyl-benzaldoximes (**1a**) (307 mg, 1.25 mmol, 5.00 equiv.) in a 15.0 mL sealed vial were added DMA (2.00 mL). Cs_2CO_3 (285 mg, 0.875 mmol, 3.50 equiv.) was added to the reaction and the resulting mixture was stirred for overnight at 90 °C. The yield of 3-Trifluoromethoxybutylbenzene (**33**) was determined by comparing the integration of the^{19}F NMR resonance of (trifluoromethoxy)cycloheptane (-57.6 ppm) with that of benzotrifluoride (-62.8 ppm). (^{19}F NMR yield: 61%).

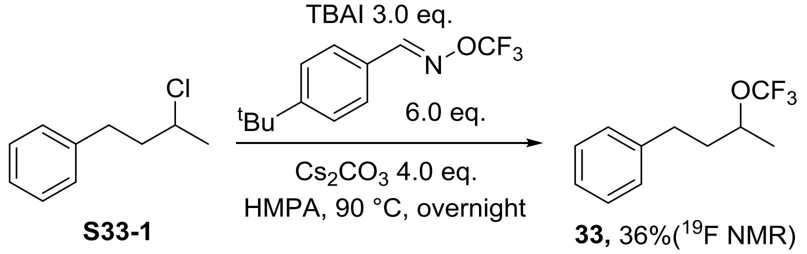

In a N_2 glovebox, to 3-chlorobutylbenzene (**S33-1**) (42.0 mg, 0.250 mmol, 1.00 equiv.), TBAI (tetrabutylammonium iodide) (277 mg, 0.750 mmol, 3.00 equiv.), (*E*)-*O*-trifluoromethyl-4-*tert*-butyl-benzaldoximes (**1a**) (368 mg, 1.50 mmol, 6.00 equiv.) in a 2.0 mL sealed vial were added HMPA (0.400 mL). Cs_2CO_3 (326 mg, 1.00 mmol, 4.00 equiv.) was added to the reaction and the resulting mixture was stirred for overnight at 90 °C. The yield of 3-trifluoromethoxybutylbenzene (**33**) was determined by comparing the integration of the ^{19}F NMR resonance of (trifluoromethoxy)cycloheptane (-57.6 ppm) with that of benzotrifluoride (-62.8 ppm). (^{19}F NMR yield: 36%).

1-(5-Trifluoromethoxyhexyl)-3,7-dihydro-3,7-dimethyl-1H-purine-2,6-dione (34)

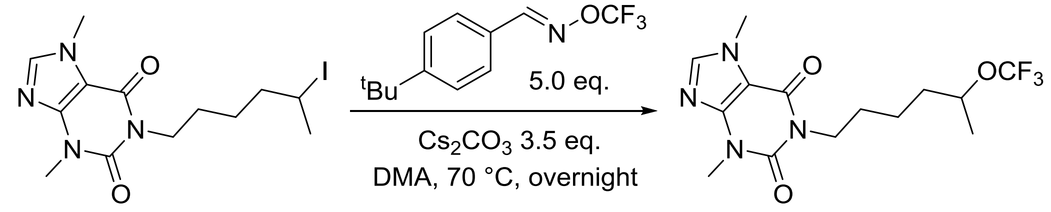

S34 **34,** 49%

In a N_2 glovebox, to 1-(5-iodohexyl)-3,7-dihydro-3,7-dimethyl-1H-purine-2,6-dione (**S34**) (83.6 mg, 0.214 mmol, 1.00 equiv.), (*E*)-*O*-trifluoromethyl-4-*tert*-butyl-benzaldoximes (**1a**) (263 mg, 1.07 mmol, 5.00 equiv.) in a 15.0 mL sealed vial were added DMA (1.70 mL). Cs_2CO_3 (244 mg, 0.750 mmol, 3.50 equiv.) was added to the reaction and the resulting mixture was stirred for overnight at 70 °C. Then, Cs_2CO_3 (138 mg, 0.424 mmol, 2.00 equiv.) was added to the reaction and the resulting mixture was stirred 2 h at 70 °C. After cooling to room temperature, the reaction mixture was filtered and concentrated *in vacuo*. The residue was purified by preparative TLC, eluting with CH_2Cl_2/MeOH 30:1 (v/v) to afford crude product. Then the crude product was purified by preparative HPLC (large column) to afford 36.8 mg 1-(5-trifluoromethoxyhexyl)-3,7-dihydro-3,7-dimethyl-1H-purine-2,6-dione (**34**) as a white solid (49% yield).

R_f = 0.2 (CH_2Cl_2/MeOH 10:1 (v/v)). NMR Spectroscopy: ^{1}H NMR (400 MHz, $CDCl_3$) δ 7.49 (s, 1H), 4.30 (q, J = 6.0 Hz, 1H), 3.98 (d, J = 8.6 Hz, 5H), 3.56 (s, 3H), 1.79 – 1.25 (m, 9H). ^{13}C NMR (101 MHz, $CDCl_3$) δ 155.4, 151.6, 148.9, 141.5, 121.9 (q, J = 253.3 Hz), 107.8, 76.3 (q, J = 2.0 Hz), 41.1, 36.2, 33.7, 29.8, 27.8, 22.5, 20.9. ^{19}F NMR (376 MHz, DMSO) δ -58.2 (s, 3F). Mass Spectrometry: HRMS-ESI (*m/z*): Calcd for $C_{14}H_{19}F_3N_4NaO_3$ $[M+Na]^+$, 371.1301. Found, 371.1304.

(*E*)-(3-(Trifluoromethoxy)prop-1-en-1-yl)benzene (35)

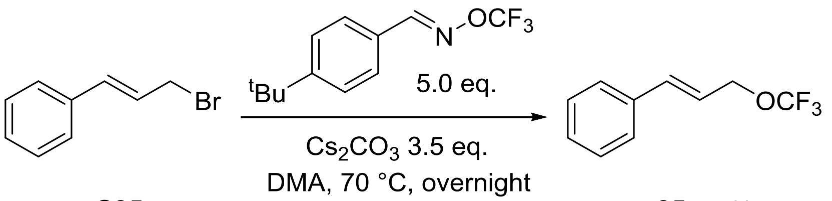

S35 **35,** 91%

In a N_2 glovebox, to cinnamyl bromide (**S35**) (49.3 mg, 0.250 mmol, 1.00 equiv.), (*E*)-*O*-trifluoromethyl-4-*tert*-butyl-benzaldoximes (**1a**) (307 mg, 1.25 mmol, 5.00 equiv.) in a 15.0 mL sealed vial were added DMA (2.00 mL). Cs_2CO_3 (285 mg, 0.875 mmol, 3.50 equiv.) was added to the reaction and the resulting mixture was stirred for overnight at 70 °C. After cooling to room temperature, the reaction mixture was filtered and concentrated *in vacuo*. The residue was purified by preparative TLC, eluting with *n*-hexane to afford 46.0 mg (*E*)-(3-(trifluoromethoxy)prop-1-en-1-yl)benzene (**35**) as a colorless liquid (91% yield).

R_f = 0.6 (*n*-hexane). NMR Spectroscopy: ^{1}H NMR (400 MHz, $CDCl_3$) δ 7.45 – 7.27 (m, 5H),

6.69 (d, J = 15.9 Hz, 1H), 6.25 (dt, J = 15.6, 6.4 Hz, 1H), 4.61 (d, J = 6.4 Hz, 2H). ^{13}C NMR (101 MHz, $CDCl_3$) δ 135.8, 135.3, 128.8, 128.6, 126.9, 121.9 (q, J = 255.3 Hz), 121.5, 68.1 (q, J = 3.5 Hz). ^{19}F NMR (376 MHz, $CDCl_3$) δ -60.0 (s, 3F). **35** is a known compound and spectral data match the reported literature values.[8]

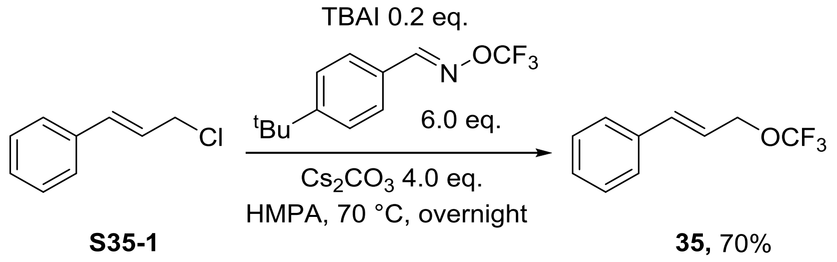

In a N_2 glovebox, to cinnamyl chloride (**S35-1**) (38.2 mg, 0.250 mmol, 1.00 equiv.), TBAI (tetrabutylammonium iodide) (18.5 mg, 0.050 mmol, 0.200 equiv.), (*E*)-*O*-trifluoromethyl-4-*tert*-butyl-benzaldoximes (**1a**) (368 mg, 1.50 mmol, 6.00 equiv.) in a 5.0 mL sealed vial were added HMPA (0.400 mL). Cs_2CO_3 (326 mg, 1.00 mmol, 4.00 equiv.) was added to the reaction and the resulting mixture was stirred for overnight at 70 °C. After cooling to room temperature, the reaction mixture was filtered and concentrated *in vacuo*. The residue was purified by preparative TLC, eluting with *n*-hexane to afford 35.5 mg (*E*)-(3-(trifluoromethoxy)prop-1-en-1-yl)benzene (**35**) as a colorless liquid (70% yield).

(3-(Trifluoromethoxy)prop-1-yn-1-yl)benzene (36)

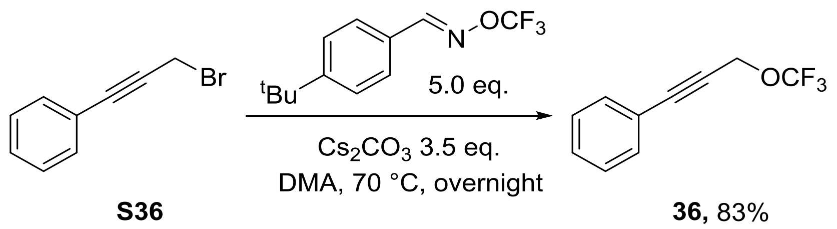

In a N_2 glovebox, to 3-bromo-1-phenylpropyne (**S36**) (48.8 mg, 0.250 mmol, 1.00 equiv.), (*E*)-*O*-trifluoromethyl-4-*tert*-butyl-benzaldoximes (**1a**) (307 mg, 1.25 mmol, 5.00 equiv.) in a 15.0 mL sealed vial were added DMA (2.00 mL). Cs_2CO_3 (285 mg, 0.875 mmol, 3.50 equiv.) was added to the reaction and the resulting mixture was stirred for overnight at 70 °C. After cooling to room temperature, the reaction mixture was filtered and concentrated *in vacuo*. The residue was purified by preparative TLC, eluting with *n*-hexane to afford 41.5 mg ((3-(trifluoromethoxy)prop-1-yn-1-yl)benzene (**36**) as a colorless liquid (83% yield).

R_f = 0.6 (*n*-hexane). NMR Spectroscopy: ^{1}H NMR (400 MHz, $CDCl_3$) δ 7.52 – 7.43 (m, 2H), 7.41 – 7.29 (m, 3H), 4.83 (s, 2H). ^{13}C NMR (101 MHz, $CDCl_3$) δ 132.0, 129.3, 128.5, 121.8 (q, J = 257.3 Hz), 121.7, 88.3, 80.9, 56.1 (q, J = 4.5 Hz).^{19}F NMR (376 MHz, $CDCl_3$) δ -61.2 (s, 3F). **36** is a known compound and spectral data match the reported literature values.[8]

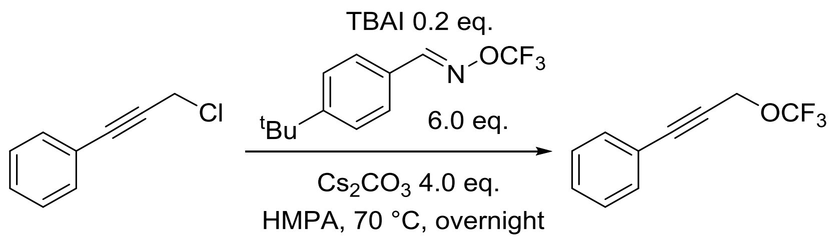

S36-1 **36**, 56%

In a N_2 glovebox, to 3-chloro-1-phenylpropyne (**S36-1**) (38.2 mg, 0.250 mmol, 1.00 equiv.), TBAI (tetrabutylammonium iodide) (18.5 mg, 0.050 mmol, 0.200 equiv.), (*E*)-*O*-trifluoromethyl-4-*tert*-butyl-benzaldoximes (**1a**) (368 mg, 1.50 mmol, 6.00 equiv.) in a 5.0 mL sealed vial were added HMPA (0.400 mL). Cs_2CO_3 (326 mg, 1.00 mmol, 4.00 equiv.) was added to the reaction and the resulting mixture was stirred for overnight at 70 °C. After cooling to room temperature, the reaction mixture was filtered and concentrated *in vacuo*. The residue was purified by preparative TLC, eluting with *n*-hexane to afford 28.2 mg ((3-(trifluoromethoxy)prop-1-yn-1-yl)benzene (**36**) as a colorless liquid (56% yield).

4-((Trifluoromethoxy)methyl)benzonitrile (37)

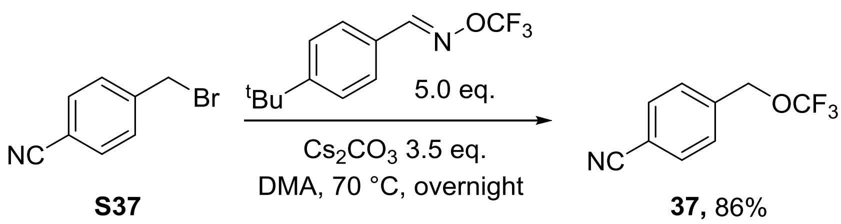

S37 **37**, 86%

In a N_2 glovebox, to 4-cyanobenzyl bromide (**S37**) (49.0 mg, 0.250 mmol, 1.00 equiv.), (*E*)-*O*-trifluoromethyl-4-*tert*-butyl-benzaldoximes (**1a**) (307 mg, 1.25 mmol, 5.00 equiv.) in a 15.0 mL sealed vial were added DMA (2.00 mL). Cs_2CO_3 (285 mg, 0.875 mmol, 3.50 equiv.) was added to the reaction and the resulting mixture was stirred for overnight at 70 °C. After cooling to room temperature, the reaction mixture was filtered and concentrated *in vacuo*. The residue was purified by preparative TLC, eluting with *n*-hexane/CH_2Cl_2 6:1 (v/v) to afford 43.0 mg 4-((trifluoromethoxy)methyl)benzonitrile (**37**) as a colorless liquid (86% yield).

R_f = 0.1 (*n*-hexane/EtOAc 60:1 (v/v)). NMR Spectroscopy: ^{1}H NMR (400 MHz, $CDCl_3$) δ 7.69 (d, *J* = 8.3 Hz, 2H), 7.48 (d, *J* = 8.2 Hz, 2H), 5.04 (s, 2H). ^{13}C NMR (101 MHz, $CDCl_3$) δ 139.1, 132.6, 128.1, 121.5 (q, *J* = 253.3 Hz), 118.4, 112.8, 67.8 (q, *J* = 3.5 Hz). ^{19}F NMR (376 MHz, $CDCl_3$) δ -60.9 (s, 3F). **37** is a known compound and spectral data match the reported literature values.[9]

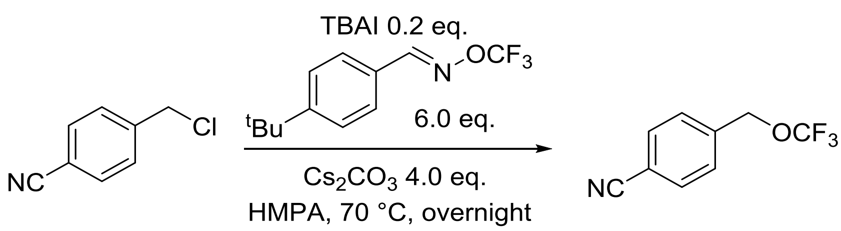

S37-1 **37**, 66%(^{19}F NMR)

In a N_2 glovebox, to 4-(Chloromethyl)tolunitrile (**S37-1**) (37.9 mg, 0.250 mmol, 1.00 equiv.), TBAI (tetrabutylammonium iodide) (18.5 mg, 0.050 mmol, 0.200 equiv.), (*E*)-*O*-trifluoromethyl-4-*tert*-butyl-benzaldoximes (**1a**) (368 mg, 1.50 mmol, 6.00 equiv.) in a 5.0 mL sealed vial were added HMPA (0.400 mL). Cs_2CO_3 (326 mg, 1.00 mmol, 4.00 equiv.) was added to the reaction and the resulting mixture was stirred for overnight at 70 °C. The yield of 4-((trifluoromethoxy)methyl)benzonitrile (**37**) was determined by comparing the integration of the ^{19}F NMR resonance of (trifluoromethoxy)cycloheptane (-60.9 ppm) with that of benzotrifluoride (-62.8 ppm). (^{19}F NMR yield: 66%).

1-(*tert*-Butyl)-4-((trifluoromethoxy)methyl)benzene (38)

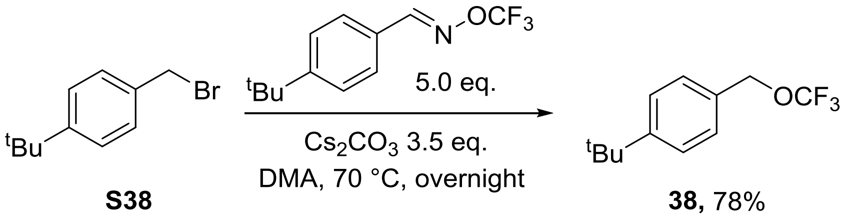

In a N_2 glovebox, to 4-*tert*-butylbenzyl bromide (**S38**) (56.8 mg, 0.250 mmol, 1.00 equiv.), (*E*)-*O*-trifluoromethyl-4-*tert*-butyl-benzaldoximes (**1a**) (307 mg, 1.25 mmol, 5.00 equiv.) in a 15.0 mL sealed vial were added DMA (2.00 mL). Cs_2CO_3 (285 mg, 0.875 mmol, 3.50 equiv.) was added to the reaction and the resulting mixture was stirred for overnight at 70 °C. After cooling to room temperature, the reaction mixture was filtered and concentrated *in vacuo*. The residue was purified by preparative TLC, eluting with *n*-hexane to afford 45.2 mg 1-(*tert*-Butyl)-4-((trifluoromethoxy)methyl)benzene (**38**) as a colorless liquid (78% yield).

R_f = 0.6 (*n*-hexane). NMR Spectroscopy: ^{1}H NMR (400 MHz, $CDCl_3$) δ 7.43 (d, *J* = 8.3 Hz, 2H), 7.32 (d, *J* = 8.2 Hz, 2H), 4.96 (s, 2H), 1.33 (s, 9H). ^{13}C NMR (101 MHz, $CDCl_3$) δ 152.3, 131.0, 128.2, 125.8, 121.8 (q, *J* = 255.1 Hz), 69.2, 34.8, 31.4. ^{19}F NMR (376 MHz, $CDCl_3$) δ -60.4 (s, 3F). **38** is a known compound and spectral data match the reported literature values.[9]

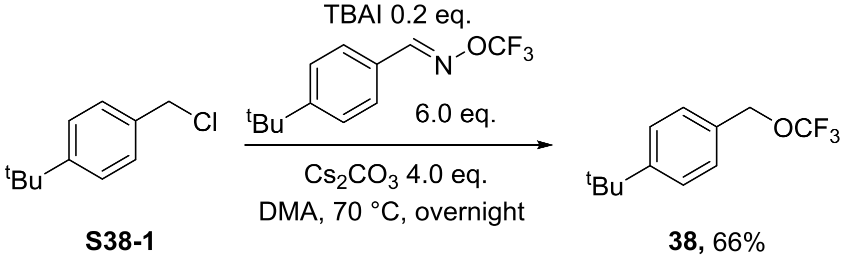

In a N_2 glovebox, to 4-*tert*-butylbenzyl chloride (**S38-1**) (45.7 mg, 0.250 mmol, 1.00 equiv.), TBAI (tetrabutylammonium iodide) (18.5 mg, 0.050 mmol, 0.200 equiv.), (*E*)-*O*-trifluoromethyl-4-*tert*-butyl-benzaldoximes (**1a**) (368 mg, 1.50 mmol, 6.00 equiv.) in a 5.0 mL sealed vial were added HMPA (0.400 mL). Cs_2CO_3 (326 mg, 1.00 mmol, 4.00 equiv.) was added to the reaction and the resulting mixture was stirred for overnight at 70 °C. After cooling to room temperature, the reaction mixture was filtered and concentrated *in vacuo*. The residue was purified by preparative TLC, eluting with *n*-hexane to afford 33.2 mg 1-(*tert*-Butyl)-4-((trifluoromethoxy)methyl)benzene (**38**) as a colorless liquid (66% yield).

1-Bromo-4-(1-(trifluoromethoxy)ethyl)benzene (39)

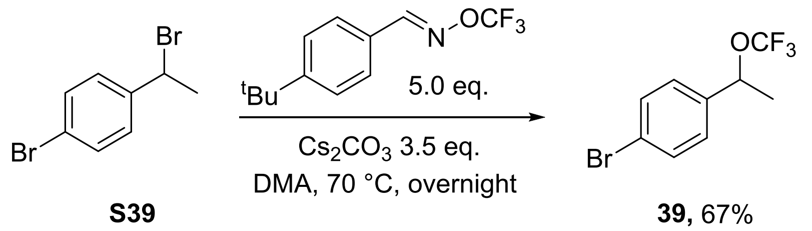

In a N_2 glovebox, to 1-bromo-4-(1-bromoethyl)benzene (**S39**) (66.0 mg, 0.250 mmol, 1.00 equiv.), (*E*)-*O*-trifluoromethyl-4-*tert*-butyl-benzaldoximes (**1a**) (307 mg, 1.25 mmol, 5.00 equiv.) in a 15.0 mL sealed vial were added DMA (2.00 mL). Cs_2CO_3 (285 mg, 0.875 mmol, 3.50 equiv.) was added to the reaction and the resulting mixture was stirred for overnight at 70 °C. After cooling to room temperature, the reaction mixture was filtered and concentrated *in vacuo*. The residue was purified by preparative TLC, eluting with *n*-hexane to afford 45.3 mg 1-bromo-4-(1-(trifluoromethoxy)ethyl)benzene (**39**) as a colorless liquid (67% yield).

R_f = 0.7 (*n*-hexane). NMR Spectroscopy: ^{1}H NMR (400 MHz, $CDCl_3$) δ 7.51 (d, *J* = 8.3 Hz, 2H), 7.23 (d, *J* = 8.3 Hz, 2H), 5.26 (q, *J* = 6.5 Hz, 1H), 1.61 (d, *J* = 6.5 Hz, 3H). ^{13}C NMR (101 MHz, $CDCl_3$) δ 139.6, 132.0, 127.6, 122.5, 121.7 (d, *J* = 255.6 Hz), 76.5 (d, *J* = 2.8 Hz), 23.5. ^{19}F NMR (376 MHz, DMSO) δ -58.6 (s, 3F). **39** is a known compound and spectral data match the reported literature values.[8]

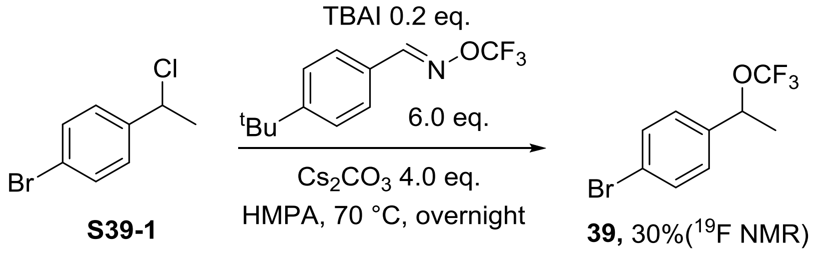

In a N_2 glovebox, to 1-bromo-4-(1-chloroethyl)benzene (**S39-1**) (54.8 mg, 0.250 mmol, 1.00 equiv.), TBAI (tetrabutylammonium iodide) (18.5 mg, 0.050 mmol, 0.200 equiv.), (*E*)-*O*-trifluoromethyl-4-*tert*-butyl-benzaldoximes (**1a**) (368 mg, 1.50 mmol, 6.00 equiv.) in a 5.0 mL sealed vial were added HMPA (0.400 mL). Cs_2CO_3 (326 mg, 1.00 mmol, 4.00 equiv.) was added to the reaction and the resulting mixture was stirred for overnight at 70 °C. The yield of 1-Bromo-4-(1-(trifluoromethoxy)ethyl)benzene (**39**) was determined by comparing the integration of the ^{19}F NMR resonance of (trifluoromethoxy)cycloheptane (-58.6 ppm) with that of benzotrifluoride (-62.8 ppm). (^{19}F NMR yield: 30%).

N,N-Dimethyl-4-(5-(4-((trifluoromethoxy)methyl)phenyl)-3-(trifluoromethyl)-1H-pyrazol-1-yl)benzenesulfonamide (40)

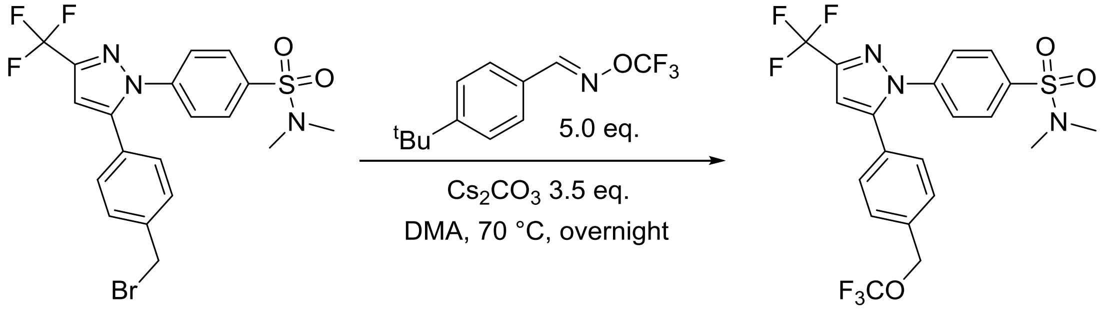

S40 **40**, 77%

In a N_2 glovebox, to N,N-Dimethyl-4-(5-(4-(bromomethyl)phenyl)-3-(trifluoromethyl)-1H-pyrazol-1-yl)benzenesulfonamide (**S40**) (122 mg, 0.250 mmol, 1.00 equiv.), (*E*)-*O*-trifluoromethyl-4-*tert*-butyl-benzaldoximes (**1a**) (307 mg, 1.25 mmol, 5.00 equiv.) in a 15.0 mL sealed vial were added DMA (2.00 mL). Cs_2CO_3 (285 mg, 0.875 mmol, 3.50 equiv.) was added to the reaction and the resulting mixture was stirred for overnight at 70 °C. After cooling to room temperature, the reaction mixture was filtered and concentrated *in vacuo*. The residue was purified by preparative TLC, eluting with *n*-hexane/EtOAc 10:1 (v/v) to afford 95.0 mg N,N-Dimethyl-4-(5-(4-(trifluoromethoxymethyl)phenyl)-3-(trifluoromethyl)-1H-pyrazol-1-yl)benzenesulfonamide (**40**) as a solid (77% yield).

R_f = 0.4 (*n*-hexane/EtOAc 4:1 (v/v)). NMR Spectroscopy: ^{1}H NMR (400 MHz, $CDCl_3$) δ 7.84 – 7.74 (m, 2H), 7.54 – 7.45 (m, 2H), 7.42 – 7.35 (m, 2H), 7.31 – 7.22 (m, 2H), 6.82 (s, 1H), 5.02 (s, 2H), 2.71 (s, 6H). ^{13}C NMR (101 MHz, $CDCl_3$) δ 144.5, 144.4 (q, J = 38.8 Hz) , 142.4, 135.7, 135.6, 129.3, 129.2, 128.9, 128.5, 125.8, 121.8 (q, J = 255.7 Hz), 121.1 (q, J = 269.2 Hz), 106.9, 68.3 (q, J = 3.5 Hz), 38.0.^{19}F NMR (376 MHz, $CDCl_3$) δ -60.5 (s, 3F), -62.4 (s, 3F). Mass Spectrometry: HRMS-ESI (*m/z*): Calcd for $C_{20}H_{18}F_6N_3O_3S$ $[M+H]^+$, 494.0968. Found, 494.0970.

Mycophenolic acid trifluoromethoxy derivative (41)

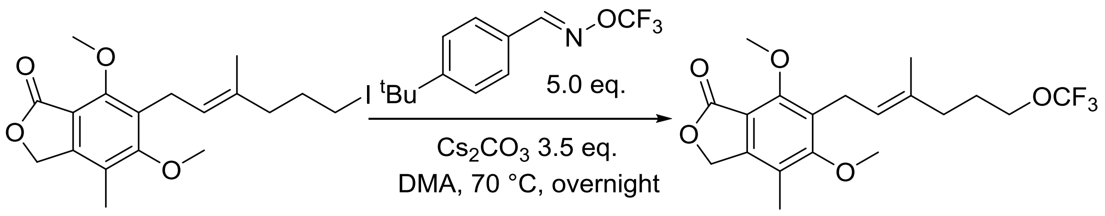

S41 **41**, 82%

In a N_2 glovebox, to Mycophenolic acid iodination derivative (**S41**) (108 mg, 0.250 mmol, 1.00 equiv.), (*E*)-*O*-trifluoromethyl-4-*tert*-butyl-benzaldoximes (**1a**) (307 mg, 1.25 mmol, 5.00 equiv.) in a 15.0 mL sealed vial were added DMA (2.00 mL). Cs_2CO_3 (285 mg, 0.875 mmol, 3.50 equiv.) was added to the reaction and the resulting mixture was stirred for overnight at 70 °C. After cooling to 50 °C, NMO (4-methylmorpholine *N*-oxide) (58.6 mg, 0.500 mmol, 2.00 equiv.) was added and the reaction mixture was stirred 2 h. Then the

resulting mixture was filtered and concentrated *in vacuo*. The residue was purified by preparative TLC, eluting with *n*-hexane/EtOAc 10:1 (v/v) to afford 80.0 mg Mycophenolic acid trifluoromethoxy derivative (**41**) as a colorless liquid (82% yield).

R_f = 0.2 (*n*-hexane/EtOAc 8:1 (v/v)). NMR Spectroscopy: ^{1}H NMR (400 MHz, $CDCl_3$) δ 5.23 – 5.01 (m, 3H), 4.02 (s, 3H), 3.88 (t, *J* = 6.5 Hz, 2H), 3.76 (s, 3H), 3.39 (d, *J* = 6.9 Hz, 2H), 2.16 (s, 3H), 2.04 (t, *J* = 7.6 Hz, 2H), 1.84 – 1.66 (m, 5H).^{13}C NMR (101 MHz, $CDCl_3$) δ 169.0, 162.8, 156.7, 146.8, 133.8, 129.0, 124.0, 121.7 (q, *J* = 253.9 Hz), 120.2, 112.6, 68.4, 67.0 (q, *J* = 3.0 Hz), 62.6, 61.0, 35.3, 26.9, 23.4, 16.1, 11.5. ^{19}F NMR (376 MHz, $CDCl_3$) δ -60.7 (s, 3F). Mass Spectrometry: HRMS-ESI (*m*/*z*): Calcd for $C_{19}H_{24}F_3O_5$ $[M+H]^+$, 389.1570. Found, 389.1568.

(3aS,4S,6aR)-1,3-Dibenzyl-4-(5-trifluoromethoxypentyl)tetrahydro-1H-thieno[3,4-d]imidazol-2(3H)-one (42)

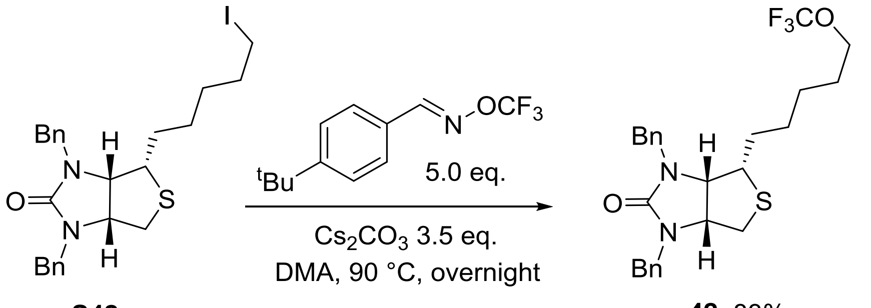

S42 **42**, 83%

In a N_2 glovebox, to (3aS,4S,6aR)-1,3-dibenzyl-4-(5-iodopentyl)tetrahydro-1H-thieno[3,4-d] imidazole-2(3H)-one (**S42**) (130 mg, 0.250 mmol, 1.00 equiv.), (*E*)-*O*-trifluoromethyl-4-*tert*-butyl-benzaldoximes (**1a**) (307 mg, 1.25 mmol, 5.00 equiv.) in a 15.0 mL sealed vial were added DMA (2.00 mL). Cs_2CO_3 (285 mg, 0.875 mmol, 3.50 equiv.) was added to the reaction and the resulting mixture was stirred for overnight at 70 °C. After cooling to 50 °C, NMO (4-methylmorpholine *N*-oxide) (58.6 mg, 0.500 mmol, 2.00 equiv..) was added and the reaction mixture was stirred 2 h. Then the resulting mixture was filtered and concentrated *in vacuo*. The residue was purified by preparative TLC, eluting with *n*-hexane/EtOAc 8:1 (v/v) to afford 99.4 mg (3aS,4S,6aR)-1,3-Dibenzyl-4-(5-trifluoromethoxypentyl)tetrahydro-1H-thieno[3,4-d]imidazol-2(3H)-one (**42**) as a colorless liquid (83% yield).

R_f = 0.2 (*n*-hexane/EtOAc 5:1 (v/v)). NMR Spectroscopy: ^{1}H NMR (400 MHz, $CDCl_3$) δ 7.47 – 7.09 (m, 10H), 5.05 (d, *J* = 15.2 Hz, 1H), 4.74 (d, *J* = 15.1 Hz, 1H), 4.15 (d, *J* = 15.2 Hz, 1H), 4.05 – 3.79 (m, 5H), 3.16 – 3.00 (m, 1H), 2.81 – 2.60 (m, 2H), 1.83 – 1.15 (m, 8H).^{13}C NMR (101 MHz, $CDCl_3$) δ 161.1, 137.1, 137.0, 128.8, 128.8, 128.4, 127.8, 127.7, 121.8 (q, *J* = 253.8 Hz), 67.4 (q, *J* = 3.1 Hz), 62.8, 61.3, 54.4, 48.1, 46.7, 34.8, 28.8, 28.7, 25.4. ^{19}F NMR (376 MHz, $CDCl_3$) δ -60.6 (s, 3F). Mass Spectrometry: HRMS-ESI (*m*/*z*): Calcd for $C_{25}H_{29}F_3N_2NaO_2S$ $[M+Na]^+$, 501.1794. Found, 501.1798.

2,3-Dimethoxy-5-methyl-6-(10-(trifluoromethoxy)decyl)cyclohexa-2,5-diene-1,4-dione (43)

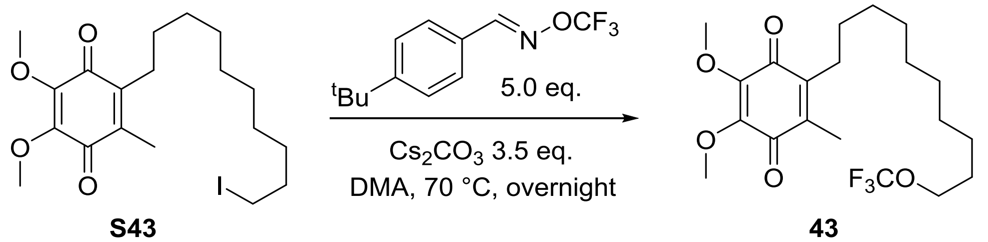

In a N_2 glovebox, to 2-(10-iododecyl)-5,6-dimethoxy-3-methylcyclohexa-2,5-diene-1,4-dione (**S43**) (112 mg, 0.250 mmol, 1.00 equiv.), (*E*)-*O*-trifluoromethyl-4-*tert*-butyl-benzaldoximes (**1a**) (307 mg, 1.25 mmol, 5.00 equiv.) in a 15.0 mL sealed vial were added DMA (2.00 mL). Cs_2CO_3 (285 mg, 0.875 mmol, 3.50 equiv.) was added to the reaction and the resulting mixture was stirred for overnight at 70 °C. After cooling to 50 °C, NMO (4-methylmorpholine *N*-oxide) (58.6 mg, 0.500 mmol, 2.00 equiv.) was added and the reaction mixture was stirred 2 h. Then the resulting mixture was filtered and concentrated *in vacuo*. The residue was purified by preparative TLC, eluting with *n*-hexane/EtOAc 6:1 (v/v) to afford 85.4 mg 2,3-dimethoxy-5-methyl-6-(10-(trifluoromethoxy)decyl)cyclohexa -2,5-diene-1,4-dione (**43**) as a red liquid (84% yield).

R_f = 0.2 (*n*-hexane/EtOAc 6:1 (v/v)). NMR Spectroscopy: ^{1}H NMR (400 MHz, $CDCl_3$) δ 4.02 – 3.85 (m, 8H), 2.50 – 2.33 (m, 2H), 1.98 (s, 3H), 1.70 – 1.58 (m, 2H), 1.44 – 1.17 (m, 14H). ^{13}C NMR (101 MHz, $CDCl_3$) δ 184.8, 184.23, 144.4, 144.4, 143.1, 138.8, 121.8 (d, *J* = 253.5 Hz), 67.6 (d, *J* = 3.1 Hz), 61.2, 29.9, 29.5, 29.4, 29.4, 29.1, 28.8, 28.8, 26.5, 25.5, 12.0. ^{19}F NMR (376 MHz, $CDCl_3$) δ -60.6 (s, 3F). **43** is a known compound and spectral data match the reported literature values.[10]

3,12-Dimethoxy cholan-24-trifluoromethoxy (44)

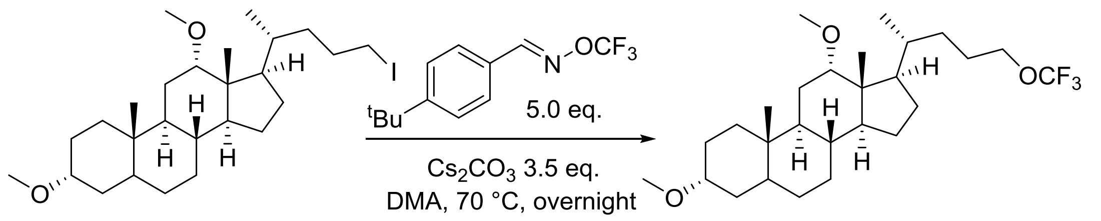

In a N_2 glovebox, to 3,12-dimethoxy cholan-24-iodo (**S44**) (129 mg, 0.250 mmol, 1.00 equiv.), (*E*)-*O*-trifluoromethyl-4-*tert*-butyl-benzaldoximes (**1a**) (307 mg, 1.25 mmol, 5.00 equiv.) in a 15.0 mL sealed vial were added DMA (2.00 mL). Cs_2CO_3 (285 mg, 0.875 mmol, 3.50 equiv.) was added to the reaction and the resulting mixture was stirred for overnight at 70 °C. After cooling to room temperature, the reaction mixture was filtered and concentrated *in vacuo*. The residue was purified by chromatography on silica gel, eluting with *n*-hexane/EtOAc 50:1 (v/v) to afford 115 mg 3,12-dimethoxy cholan-24-trifluoromethoxy (**44**) as a colorless liquid (97% yield).

R_f = 0.4 (*n*-hexane/EtOAc 20:1 (v/v)). NMR Spectroscopy: ^{1}H NMR (400 MHz, $CDCl_3$) δ

3.90 (t, J = 6.7 Hz, 2H), 3.37 (s, 1H), 3.32 (s, 3H), 3.23 (s, 3H), 3.18 – 3.07 (m, 1H), 1.90 – 1.63 (m, 9H), 1.53 (p, J = 7.7, 7.3 Hz, 4H), 1.46 – 1.05 (m, 13H), 0.92 – 0.87 (m, 6H), 0.65 (s, 3H). ^{13}C NMR (101 MHz, $CDCl_3$) δ 121.8 (q, J = 253.6 Hz), 82.4, 80.6, 68.2 (d, J = 2.9 Hz), 55.7, 55.6, 49.0, 46.6, 46.4, 42.2, 36.1, 35.4, 35.2, 34.6, 33.6, 32.7, 31.6, 27.6, 27.4, 26.9, 26.2, 25.5, 23.8, 23.4, 22.0, 17.7, 12.8. ^{19}F NMR (376 MHz, $CDCl_3$) δ -60.5 (s, 3F). Mass Spectrometry: HRMS-ESI (*m/z*): Calcd for $C_{27}H_{45}F_3NaO_3$ $[M+Na]^+$, 497.3213. Found, 497.3218.

Ezetimibe derivative (45)

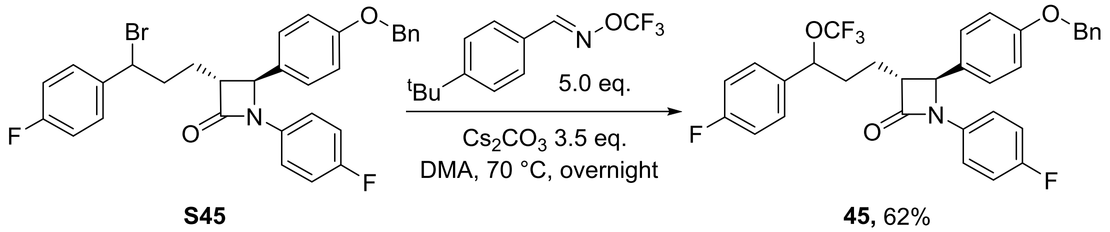

In a N_2 glovebox, to ezetimibe derivative (**S45**) (113 mg, 0.200 mmol, 1.00 equiv.), (*E*)-*O*-trifluoromethyl-4-*tert*-butyl-benzaldoximes (**1a**) (245 mg, 1.00 mmol, 5.00 equiv.) in a 15.0 mL sealed vial were added DMA (1.60 mL). Cs_2CO_3 (228 mg, 0.700 mmol, 3.50 equiv.) was added to the reaction and the resulting mixture was stirred for overnight at 70 °C. After cooling to room temperature, the reaction mixture was filtered and concentrated *in vacuo*. The residue was purified by preparative TLC, eluting with n-hexane/CH_2Cl_2 2:1 (v/v) to afford 70.2 mg ezetimibe derivative (**45**) as a white solid (62% yield).

R_f = 0.2 (*n*-hexane/EtOAc 5:1 (v/v)). NMR Spectroscopy: ^{1}H NMR (400 MHz, $CDCl_3$) δ 7.47 – 7.17 (m, 11H), 7.10 – 6.87 (m, 6H), 5.17 – 5.01 (m, 3H), 4.55 (dd, J = 9.6, 2.1 Hz, 1H), 3.12 – 2.98 (m, 1H), 2.26 – 1.76 (m, 3H).

a. ^{13}C NMR (101 MHz, $CDCl_3$) δ 166.9, 162.8 (d, J = 247.5 Hz), 159.2, 159.1 (d, J = 243.5 Hz), 136.7, 134.8 (d, J = 15.0 Hz), 133.9 (t, J = 2.6 Hz), 129.5, 128.8, 128.2, 128.0 (d, J = 8.4 Hz), 127.6, 127.3, 121.7 (q, J = 255.6 Hz), 118.5 (d, J = 7.7 Hz), 115.9 (d, J = 22.7 Hz), 115.8 (d, J = 21.8 Hz), 115.7, 79.7 (q, J = 2.2 Hz), 70.2, 60.9, 60.0, 34.7, 24.4.
b. ^{13}C NMR (101 MHz, $CDCl_3$) δ 167.0, 162.8 (d, J = 247.5 Hz), 159.2, 159.1 (d, J = 243.5 Hz), 136.7, 134.8 (d, J = 15.0 Hz), 133.9 (d, J = 2.6 Hz), 129.5, 128.8, 128.2, 127.9 (d, J = 8.3 Hz), 127.6, 127.3, 121.7 (d, J = 255.6 Hz), 118.5 (d, J = 7.7 Hz), 115.9 (d, J = 22.7 Hz), 115.8 (d, J = 21.9 Hz), 115.7, 80.0 (q, J = 2.2 Hz), 70.2, 61.0, 60.0, 35.0, 24.9.

^{19}F NMR (376 MHz, DMSO) δ -58.3 (s, 3F). Mass Spectrometry: HRMS-ESI (*m/z*): Calcd for $C_{31}H_{26}F_2NO_2$ $[M-OCF_3]^+$, 482.1926. Found, 482.1929.

Gibberellic acid derivative (46)

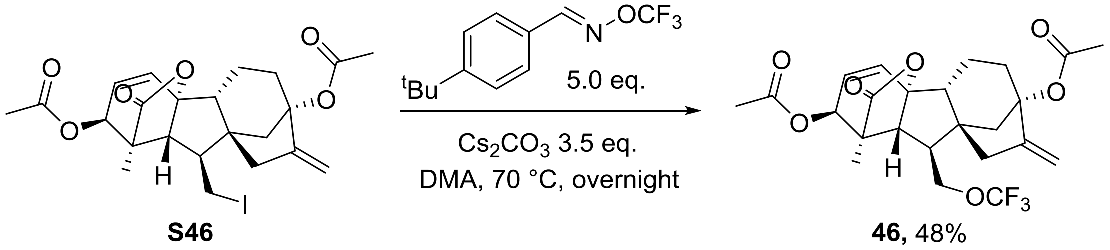

S46 **46,** 48%

In a N_2 glovebox, to gibberellic acid derivative (**S46**) (52.64 mg, 0.100 mmol, 1.00 equiv.), (*E*)-*O*-trifluoromethyl-4-*tert*-butyl-benzaldoximes (**1a**) (123 mg, 0.500 mmol, 5.00 equiv.) in a 5.0 mL sealed vial were added DMA (0.800 mL). Cs_2CO_3 (114 mg, 0.350 mmol, 3.50 equiv.) was added to the reaction and the resulting mixture was stirred for overnight at 70 °C. After cooling to room temperature, the reaction mixture was filtered and concentrated *in vacuo*. The residue was purified by preparative HPLC (large column) to afford 23.2 mg gibberellic acid derivative (**46**) as a white solid (48% yield).

R_f = 0.2 (*n*-hexane/EtOAc 5:1 (v/v)). NMR Spectroscopy: ^{1}H NMR (400 MHz, $CDCl_3$) δ 6.38 (d, *J* = 9.3 Hz, 1H), 5.86 (dd, *J* = 9.3, 3.8 Hz, 1H), 5.33 (d, *J* = 3.8 Hz, 1H), 5.17 – 5.12 (m, 1H), 5.06 – 5.01 (m, 1H), 4.17 – 4.00 (m, 2H), 2.75 (d, *J* = 10.5 Hz, 1H), 2.71 – 2.60 (m, 1H), 2.38 – 2.29 (m, 1H), 2.19 (d, *J* = 19.7 Hz, 4H), 2.10 (s, 3H), 2.06 – 1.95 (m, 4H), 1.92 – 1.85 (m, 1H), 1.84 – 1.68 (m, 2H), 1.27 (s, 3H). ^{13}C NMR (101 MHz, $CDCl_3$) δ 177.0, 170.0, 153.1, 134.7, 129.1, 121.6 (q, *J* = 255.4 Hz), 107.8, 90.1, 84.1, 71.1, 66.3 (q, *J* = 2.9 Hz), 66.3, 52.5, 52.3, 50.8, 50.3, 43.7, 41.5, 41.2, 36.5, 22.2, 20.8, 16.9, 15.0. ^{19}F NMR (376 MHz, $CDCl_3$) δ -60.8 (s, 3F). Mass Spectrometry: HRMS-ESI (*m/z*): Calcd for $C_{24}H_{27}F_3NaO_7$ $[M+Na]^+$, 507.1601. Found, 507.1603.

7-(5-Trifluoromethoxypentyl)Tadalafil (47)

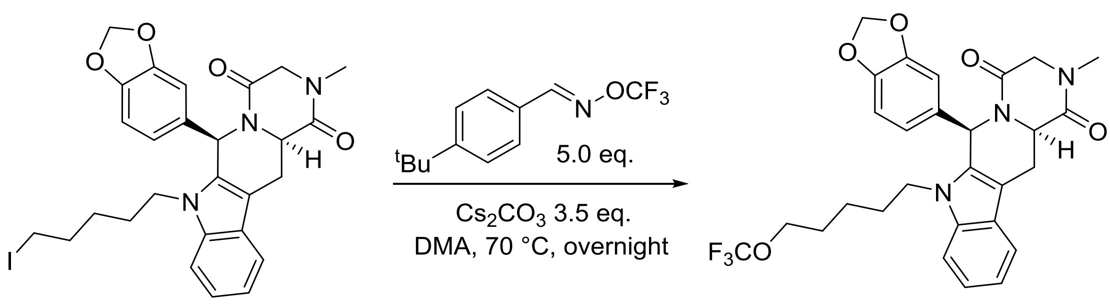

S47 **47,** 80%

In a N_2 glovebox, to 7-(5-iodopentyl)tadalafil (**S47**)(73.2 mg, 0.125 mmol, 1.00 equiv.), (*E*)-*O*-trifluoromethyl-4-*tert*-butyl-benzaldoximes (**1a**) (154 mg, 0.625 mmol, 5.00 equiv.) in a 15.0 mL sealed vial were added DMA (1.00 mL). Cs_2CO_3 (143 mg, 0.438 mmol, 3.50 equiv.) was added to the reaction and the resulting mixture was stirred for overnight at 70 °C.

After cooling to room temperature, the reaction mixture was filtered and concentrated *in vacuo*. The residue was purified by preparative TLC, eluting with CH_2Cl_2 to afford 54.5 mg 7-(5-trifluoromethoxypentyl)tadalafil (**47**) as a white solid (80% yield).

R_f = 0.2 (CH_2Cl_2/CH_3OH 80:1 (v/v)). NMR Spectroscopy: ^1H NMR (400 MHz, $CDCl_3$) δ 7.54 (d, J = 7.8 Hz, 1H), 7.32 – 7.21 (m, 2H), 7.15 (t, J = 7.2 Hz, 1H), 7.01 (s, 1H), 6.81 (s, 1H), 6.75 – 6.62 (m, 2H), 5.95 (s, 2H), 4.35 (dd, J = 12.0, 4.2 Hz, 1H), 4.19 – 3.95 (m, 2H), 3.95 – 3.62 (m, 4H), 3.56 (dd, J = 15.5, 4.4 Hz, 1H), 3.03 – 2.90 (m, 4H), 1.81 – 1.25 (m, 6H). ^{13}C NMR (101 MHz, $CDCl_3$) δ 165.7, 161.4, 148.4, 148.3, 131.7, 130.8, 126.2, 122.5, 122.4, 121.7 (q, J = 254.2 Hz). 119.8, 118.7, 109.6, 109.1, 108.5, 107.8, 101.5, 67.0 (q, J = 3.1 Hz), 52.0, 51.6, 51.0, 43.7, 33.5, 29.4, 28.4, 27.6, 23.0. ^{19}F NMR (376 MHz, $CDCl_3$) δ -60.4 (s, 3F). Mass Spectrometry: HRMS-ESI (*m/z*): Calcd for $C_{28}H_{28}F_3N_3NaO_5$ $[M+Na]^+$, 566.1873. Found, 566.1878.

5-Trifluoromethoxyvaleric acid acetylcyclosporin A ester (48)

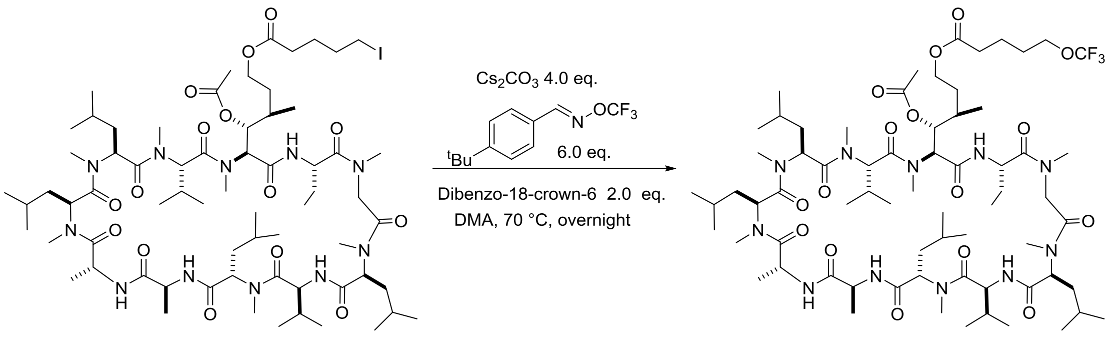

S48 **48**

In a N_2 glovebox, to 5-iodovaleric acid acetylcyclosporin A ester (**S48**) (85.7 mg, 0.0600 mmol, 1.00 equiv.), Dibenzo-18-crown-6(43.3 mg, 0.120 mmol, 2.00 equiv.), (*E*)-4-(*tert*-butyl) benzaldehyde *O*-trifluoromethyl oxime (88.3 mg, 0.360 mmol, 6.00 equiv.) in a 2.0 mL sealed vial were added DMA (0.480 mL). Cs_2CO_3 (78.2 mg, 0.240 mmol, 4.00 equiv.) was added to the reaction and the resulting mixture was stirred for overnight at 70 °C. After cooling to room temperature, the reaction mixture was filtered and concentrated *in vacuo*. The residue was purified by preparative TLC, eluting with CH_2Cl_2/CH_3OH 60:1 (v/v) to afford 33.4 mg 5-trifluoromethoxyvaleric acid acetylcyclosporin A ester (**48**) as a white solid (40% yield).

R_f = 0.1 (CH_2Cl_2/CH_3OH 60:1 (v/v)). NMR Spectroscopy: ^1H NMR (400 MHz, $CDCl_3$) δ 8.60 (d, J = 9.7 Hz, 1H), 8.01 (d, J = 6.8 Hz, 1H), 7.58 (d, J = 7.7 Hz, 1H), 7.47 (d, J = 9.0 Hz, 1H), 5.66 (dd, J = 10.9, 4.0 Hz, 1H), 5.52 – 5.44 (m, 2H), 5.40 (dd, J = 12.1, 3.4 Hz, 1H), 5.26 (dd, J = 11.6, 3.8 Hz, 1H), 5.17 – 5.08 (m, 1H), 5.02 – 4.92 (m, 2H), 4.89 – 4.79 (m, 1H), 4.72 (t, J = 9.5 Hz, 1H), 4.63 (d, J = 13.8 Hz, 1H), 4.44 – 4.34 (m, 1H), 4.32 – 4.21 (m, 1H), 3.97 (t, J = 5.9 Hz, 2H), 3.89 – 3.78 (m, 1H), 3.42 (s, 3H), 3.28 (s, 3H), 3.27 (s, 3H), 3.20 (s, 3H), 3.16 (d, J = 13.9 Hz, 1H), 3.07 (s, 3H), 2.66 (s, 3H), 2.64 (s, 3H), 2.49 – 2.39 (m, 1H), 2.35 – 2.28 (m, 1H), 2.27 – 2.05 (m, 5H), 1.99 (s, 3H), 1.96 – 1.80 (m, 3H), 1.80 – 1.62 (m, 9H), 1.47 – 1.12 (m, 12H), 1.07 – 0.72 (m, 42H)^{13}C NMR (101 MHz, $CDCl_3$) δ 173.9, 173.6,

173.2, 172.9, 172.7, 171.6, 171.3, 171.1, 170.9, 170.6, 170.5, 170.2, 168.1, 121.8 (q, *J* = 253.9 Hz), 73.7, 67.2 (q, *J* = 3.1 Hz), 63.5, 58.6, 57.3, 56.3, 55.4, 54.9, 54.0, 50.1, 48.8, 48.5, 48.0, 44.8, 41.1, 39.4, 39.2, 37.2, 36.0, 33.3, 32.3, 31.7, 31.6, 31.6, 31.5, 30.4, 30.0, 29.8, 29.6, 28.9, 28.3, 25.3, 25.0, 24.9, 24.8, 24.6, 24.2, 23.9, 23.9, 23.6, 22.0, 21.5, 21.2, 21.0, 20.8, 20.6, 19.9, 18.8, 18.5, 18.2, 17.8, 15.1, 10.0. ^{19}F NMR (376 MHz, DMSO) δ -61.1 (s, F). Mass Spectrometry: HRMS-MALDI (*m/z*): Calcd for $C_{68}H_{118}F_3N_{11}NaO_{16}$ $[M+Na]^+$, 1424.8602. Found, 1424.8605.

11-Benzyloxymethylenoxy 22-trifluoromethoxypleuromutilin (49)

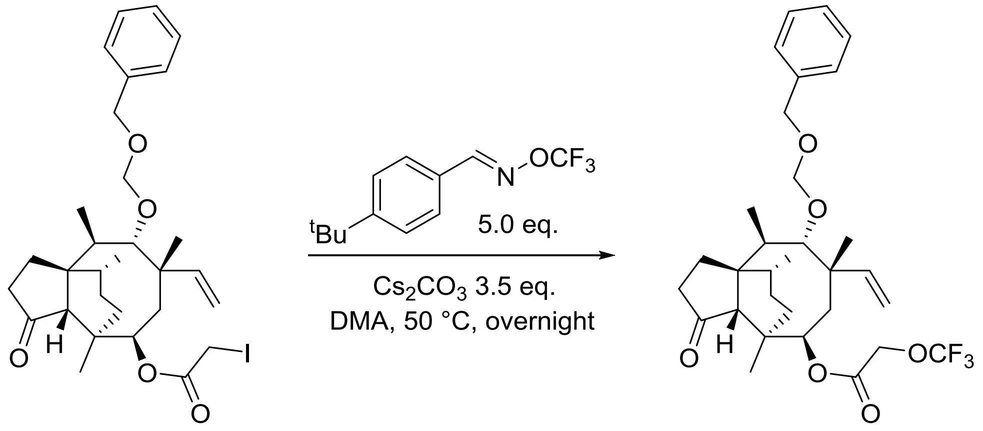

In a N_2 glovebox, to 11-benzyloxymethylenoxy 22-iodopleuromutilin (**S49**) (60.9 mg, 0.100 mmol, 1.00 equiv.), (*E*)-*O*-trifluoromethyl-4-*tert*-butyl-benzaldoximes (**1a**) (123 mg, 0.500 mmol, 5.00 equiv.) in a 5.0 mL sealed vial were added DMA (0.800 mL). Cs_2CO_3 (114 mg, 0.350 mmol, 3.50 equiv.) was added to the reaction and the resulting mixture was stirred for overnight at 50 °C. After cooling to room temperature, the reaction mixture was filtered and concentrated *in vacuo*. The residue was purified by preparative TLC, eluting with with *n*-hexane/EtOAc 5:1 (v/v) to afford crude product. Then the crude product was purified by preparative HPLC (large column) to afford 32.8 mg 11-benzyloxymethylenoxy 22-trifluoromethoxypleuromutilin (**49**) as a white solid (58% yield).

R_f = 0.2 (*n*-hexane/EtOAc 5:1 (v/v)). NMR Spectroscopy: ^{1}H NMR (400 MHz, $CDCl_3$) δ 7.42 – 7.26 (m, 5H), 6.30 (dd, *J* = 17.5, 11.2 Hz, 1H), 5.78 (d, *J* = 8.3 Hz, 1H), 5.24 (dd, *J* = 33.5, 14.3 Hz, 2H), 4.86 – 4.73 (m, 2H), 4.67 (s, 2H), 4.37 (s, 2H), 3.38 (d, *J* = 5.7 Hz, 1H), 2.49 – 2.34 (m, 1H), 2.32 – 1.99 (m, 4H), 1.85 – 1.30 (m, 10H), 1.29 – 1.06 (m, 4H), 0.98 (d, *J* = 6.9 Hz, 3H), 0.70 (d, *J* = 6.7 Hz, 3H). ^{13}C NMR (101 MHz, $CDCl_3$) δ 216.0, 163.6, 138.8, 136.7, 127.4, 126.7, 126.6, 120.4 (q, *J* = 256.7 Hz), 115.4, 95.9, 82.5, 69.8, 62.6 (q, *J* = 3.5 Hz), 57.4, 44.4, 43.9, 43.6, 41.0, 36.0, 35.5, 33.6, 29.3, 27.8, 25.7, 24.1, 15.1, 13.7, 11.1. ^{19}F NMR (376 MHz, DMSO) δ -60.9 (s, 3F). Mass Spectrometry: HRMS-ESI (*m/z*): Calcd for $C_{31}H_{41}F_3NaO_6$ $[M+Na]^+$, 589.2747. Found, 589.2750.

4-(Trifluoromethoxy)butyl)benzene[8] (50)

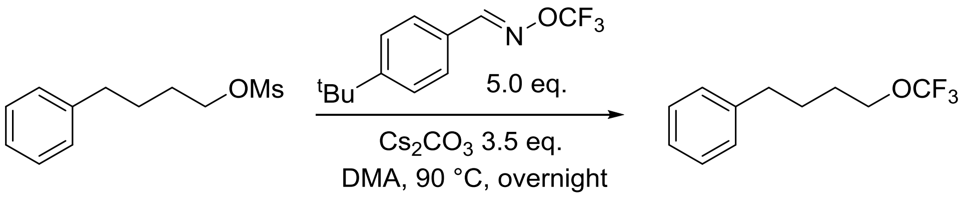

S50-1 **50**, 94%(^{19}F NMR)

In a N_2 glovebox, to 4-phenylbutyl methanesulfonate (**S50-1**) (11.4 mg, 0.0500 mmol, 1.00 equiv.), (*E*)-*O*-trifluoromethyl-4-*tert*-butyl-benzaldoximes (**1a**) (61.3 mg, 0.250 mmol, 5.00 equiv.) in a 2.00 mL sealed vial were added DMA (0.400 mL). Cs_2CO_3 (57.0 mg, 0.175 mmol, 3.50 equiv.) was added to the reaction and the resulting mixture was stirred for overnight at 90 °C. The yield of 4-(Trifluoromethoxy)butyl)benzene (**50**) was determined by comparing the integration of the ^{19}F NMR resonance of 4-(Trifluoromethoxy)butyl)benzene (-60.7 ppm) with that of benzotrifluoride (-62.8 ppm). (^{19}F NMR yield 94% yield).

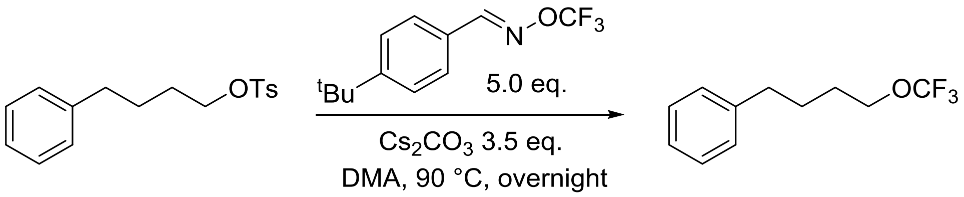

S50-2 **50**, 84%(^{19}F NMR)

In a N_2 glovebox, to 4-Phenylbutyl 4-methylbenzenesulfonate (**S50-2**) (15.2 mg, 0.0500 mmol, 1.00 equiv.), (*E*)-*O*-trifluoromethyl-4-*tert*-butyl-benzaldoximes (**1a**) (61.3 mg, 0.250 mmol, 5.00 equiv.) in a 2.00 mL sealed vial were added DMA (0.400 mL). Cs_2CO_3 (57.0 mg, 0.175 mmol, 3.50 equiv.) was added to the reaction and the resulting mixture was stirred for overnight at 90 °C. The yield of 4-(Trifluoromethoxy)butyl)benzene (**50**) was determined by comparing the integration of the ^{19}F NMR resonance of 4-(Trifluoromethoxy)butyl)benzene (-60.7 ppm) with that of benzotrifluoride (-62.8 ppm). (^{19}F NMR yield 84% yield).

Gram-scale synthesis of 1-(Trifluoromethoxy)hexadecane (28)

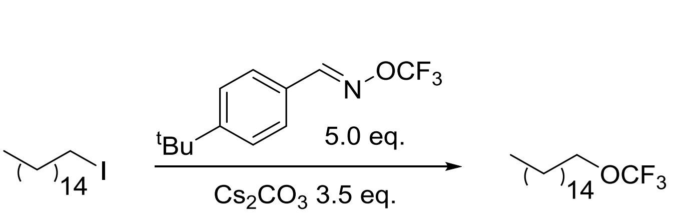

S28 **28**, 89%

In a N_2 glovebox, to 1-iodohexadecane (1.60 g, 4.54 mmol, 1.00 equiv.), (*E*)-*O*-trifluoromethyl-4-*tert*-butyl-benzaldoximes (**1a**) (5.57 g, 22.7 mmol, 5.00 equiv.) in a 150 mL sealed vial were added DMA (36.0 mL). Cs_2CO_3 (5.18 g, 15.9 mmol, 3.50 equiv.) was added to the reaction and the resulting mixture was stirred for overnight at 70 °C. After cooling to 50 °C, NMO (4-methylmorpholine *N*-oxide) (1.06 g, 9.08 mmol, 2.00 equiv.) was

added and the reaction mixture was stirred 2 h. Then the resulting mixture was filtered and concentrated *in vacuo*. The residue was purified by chromatography on silica gel, eluting with n-hexane to afford 1.25 g 1-trifluoromethoxyhexadecane (**28**) as a colorless liquid (89% yield).

Comparision of TFBO with TFMT

Supplementary Table 12: Comparision of TFBO with TFMT

Substrate	Yield [%]/(^{19}**F NMR**)		Yield [%]/(**isoliated**)
	**TFMT** “$AgOCF_3$”	**TFMT** “$n Bu_4NOCF_3$”	**TFBO**
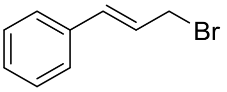	57[a]	53[a]	91
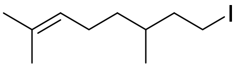	64[a]/62	4[a]/4	86
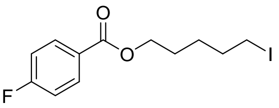	62	13	92
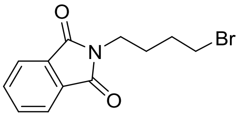	21	2	86
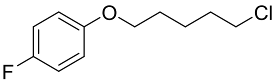	0	trace	71
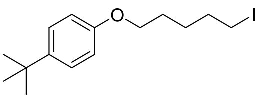	76	24	94
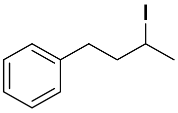	38	6	71
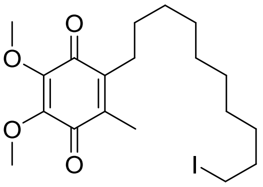	69	2	84

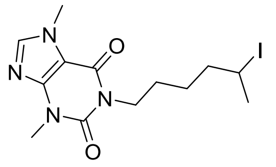	10	4	49

a, The yield was reported in the literature and the method was developed by Kolomeitsev[11] and Langlois[7]

The method of "$AgOCF_3$":

In a 2.00 mL sealed vial, equipped with a rubber septum and a magnetic stirrer, silver fluoride (42.3 mg, 0.333 mmol, 1.00 equiv.) was introduced. In a N_2 glovebox, anhydrous CH_3CN (0.670 mL) were added and the heterogenous mixture was cooled to -30 °C, TFMT (0.100 mL) was then added, the vessel was tightly closed (autogenous pressure of COF_2 is needed to allow the reaction to proceed) and the reaction mixture was stirred for 2 h at -30 °C. After addition of the substrate (neat when liquid or dissolved in the minimum of CH_3CN when solid) by the mean of a gas-tight syringe, stirring was continued at -30 °C for 30 mins then at RT for 24 h (in the dark). The yield was determined by comparing the integration of the ^{19}F NMR resonance of product with that of benzotrifluoride (-62.8 ppm).

The method of "*n*Bu_4NOCF_3":

In a 2.00 mL sealed vial, equipped with a rubber septum and a magnetic stirrer, silver fluoride (42.3 mg, 0.333 mmol, 1.00 equiv.) and TBAI (123 mg, 0.333 mmol, 1.00 equiv.) was introduced. In a N_2 glovebox, anhydrous CH_3CN (0.670 mL) were added and the heterogenous mixture was cooled to -30 °C, TFMT (0.100 mL) was then added, the vessel was tightly closed (autogenous pressure of COF_2 is needed to allow the reaction to proceed) and the reaction mixture was stirred for 2 h at -30 °C. After addition of the substrate (neat when liquid or dissolved in the minimum of CH_3CN when solid) by the mean of a gas-tight syringe, stirring was continued at -30 °C for 30 mins then at RT for 24 h (in the dark). The yield was determined by comparing the integration of the ^{19}F NMR resonance of product with that of benzotrifluoride (-62.8 ppm).

Mechanism study

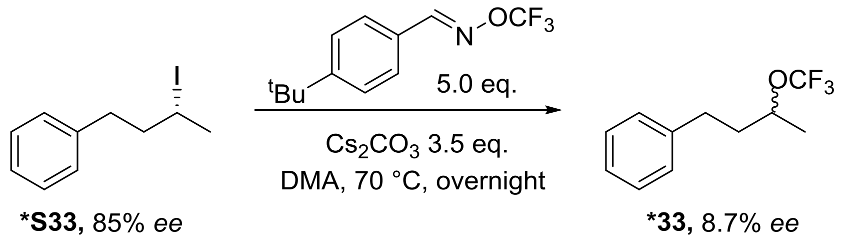

***S33,** 85% *ee* ***33,** 8.7% *ee*

In a N_2 glovebox, to 3-iodobutylbenzene (***S33**) (65.0 mg, 0.250 mmol, 1.00 equiv.), (*E*)-*O*-trifluoromethyl-4-*tert*-butyl-benzaldoximes (**1a**) (307 mg, 1.25 mmol, 5.00 equiv.) in a 15.0 mL sealed vial were added DMA (2.00 mL). Cs_2CO_3 (285 mg, 0.875 mmol, 3.50 equiv.) was added to the reaction and the resulting mixture was stirred for overnight at 70 °C. After cooling to room temperature, the reaction mixture was filtered and concentrated *in vacuo*. The residue was purified by preparative TLC, eluting with n-hexane to afford 3-trifluoromethoxybutylbenzene (***33**) as a colorless liquid.

8.7% *ee* HPLC (Daicel OD-H+OD-H chiralpak, *i*-PrOH/hexane = 2:98, 0.5 mL/min, 220 nm), t_1 = 18.178 min (major), t_2 = 19.751 min (minor).

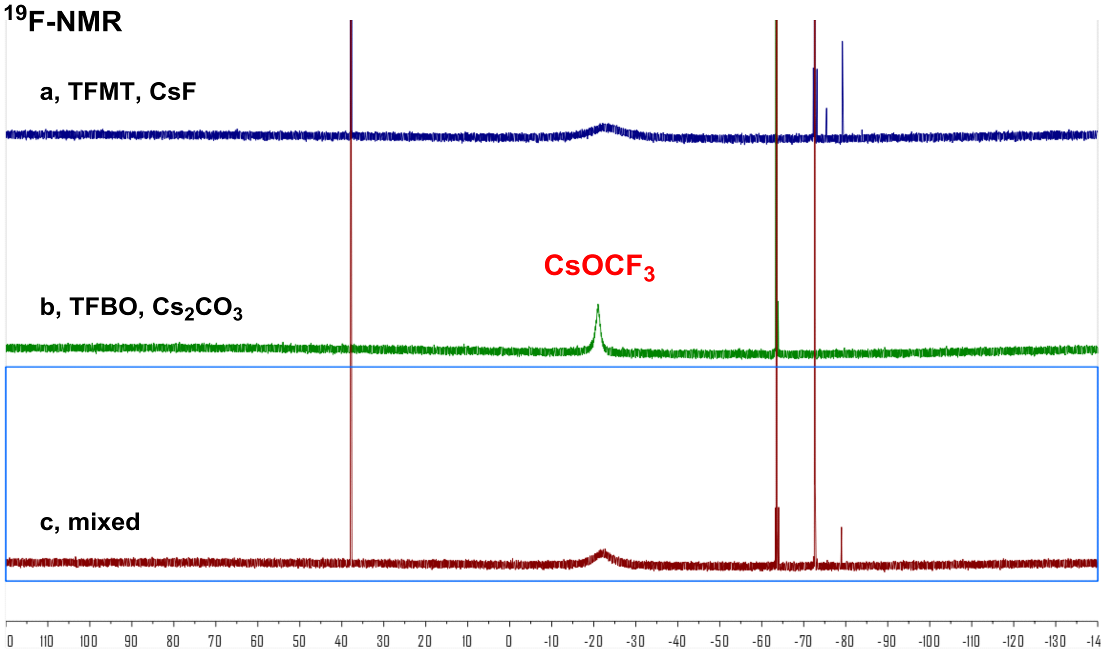

Supplementary Figure 1. ^{19}F NMR Spectra of Intermediates

a, In a 2.00 mL sealed vial, equipped with a rubber septum and a magnetic stirrer, Caesium fluoride (50.6 mg, 0.333 mmol, 1.00 equiv.) was introduced. In a N_2 glovebox, anhydrous CH_3CN (0.670 mL) were added and the heterogenous mixture was cooled to -30 °C, TFMT (0.100 mL) was then added and the reaction mixture was stirred for 2 h at -30 °C.

b, In a N_2 glovebox, to (*E*)-*O*-trifluoromethyl-4-*tert*-butyl-benzaldoximes (**1a**) (61.3 mg, 0.250 mmol) in a 2.00 mL sealed vial were added DMA (0.400 mL). Cs_2CO_3 (57.0 mg, 0.175 mmol) was added to the reaction and the resulting mixture was stirred for 0.5 h at 30 °C.

c, After the reaction, the reaction mixture of a and b was mixed.

X-ray Crystal Structure Data for (*E*)-4-Nitrobenzaldehyde *O*-trifluoromethyl oxime (1d) (CCDC: 1907514)

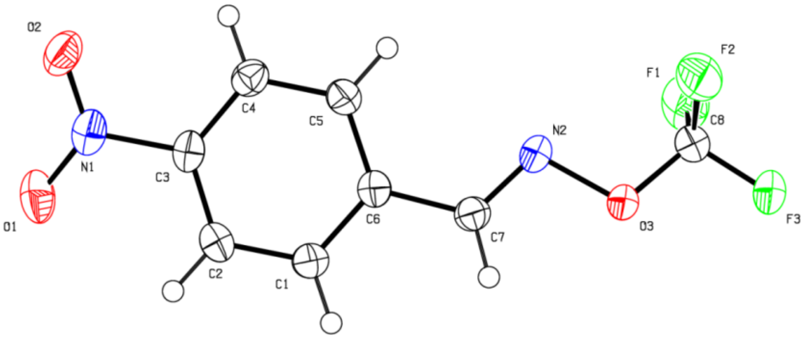

Supplementary Table 13: Crystal data and structure refinement for **1d**

Identification code	R20180423A1
Empirical formula	C8 H5 F3 N2 O3

Formula weight	234.14
Temperature	113(2) K
Wavelength	0.71073 A
Crystal system, space group	Monoclinic, P2(1)/c
Unit cell dimensions	a = 14.604(3) A alpha = 90 deg.
	b = 4.9833(10) A beta = 91.48(3) deg.
	c = 12.718(3) A gamma = 90 deg.
Volume	925.2(3) A^3
Z, Calculated density	4, 1.681 Mg/m^3
Absorption coefficient	0.167 mm^-1
F(000)	472
Crystal size	0.200 x 0.180 x 0.120 mm
Theta range for data collection	1.395 to 27.861 deg.
Limiting indices	-19<=h<=18, -6<=k<=6, -16<=l<=16
Reflections collected / unique	10389 / 2204 [R(int) = 0.0593]
Completeness to theta = 25.242	99.7 %
Absorption correction	Semi-empirical from equivalents
Max. and min. transmission	1.0000 and 0.8555
Refinement method	Full-matrix least-squares on F^2
Data / restraints / parameters	2204 / 0 / 145
Goodness-of-fit on F^2	1.058
Final R indices [I>2sigma(I)]	R1 = 0.0393, wR2 = 0.1068
R indices (all data)	R1 = 0.0568, wR2 = 0.1128
Extinction coefficient	n/a
Largest diff. peak and hole	0.212 and -0.234 e. A^-3

Supplementary Table 14. Atomic coordinates (x 10^4) and equivalent isotropic displacement parameters (A^2 x 10^3) for R20180423A1.
U(eq) is defined as one third of the trace of the orthogonalized
Uij tensor.

	x	y	z	U(eq)
F(1)	5480(1)	10480(3)	6762(1)	60(1)
F(2)	6426(1)	13698(3)	6781(1)	62(1)
F(3)	5077(1)	14277(3)	6121(1)	54(1)

O(1)	9313(1)	34(2)	2419(1)	43(1)
O(2)	10067(1)	593(3)	3894(1)	44(1)
O(3)	6011(1)	11910(2)	5244(1)	36(1)
N(1)	9437(1)	1132(3)	3276(1)	32(1)
N(2)	6730(1)	9920(3)	5296(1)	31(1)
C(1)	7573(1)	6168(3)	3094(1)	30(1)
C(2)	8194(1)	4219(3)	2814(1)	30(1)
C(3)	8788(1)	3234(3)	3577(1)	26(1)
C(4)	8795(1)	4132(3)	4609(1)	30(1)
C(5)	8171(1)	6090(3)	4882(1)	29(1)
C(6)	7554(1)	7115(3)	4124(1)	25(1)
C(7)	6869(1)	9158(3)	4364(1)	28(1)
C(8)	5761(1)	12558(4)	6218(1)	36(1)

Supplementary Table 15. Bond lengths [A] and angles [deg] for R20180423A1.

F(1)-C(8)	1.317(2)
F(2)-C(8)	1.321(2)
F(3)-C(8)	1.319(2)
O(1)-N(1)	1.2284(19)
O(2)-N(1)	1.2245(19)
O(3)-C(8)	1.3393(19)
O(3)-N(2)	1.4448(17)
N(1)-C(3)	1.471(2)
N(2)-C(7)	1.266(2)
C(1)-C(2)	1.381(2)
C(1)-C(6)	1.394(2)
C(1)-H(1)	0.9500
C(2)-C(3)	1.375(2)
C(2)-H(2)	0.9500
C(3)-C(4)	1.386(2)
C(4)-C(5)	1.385(2)
C(4)-H(4)	0.9500
C(5)-C(6)	1.400(2)
C(5)-H(5)	0.9500
C(6)-C(7)	1.465(2)

C(7)-H(7)	0.9500
C(8)-O(3)-N(2)	109.80(12)
O(2)-N(1)-O(1)	124.26(14)
O(2)-N(1)-C(3)	117.90(14)
O(1)-N(1)-C(3)	117.84(14)
C(7)-N(2)-O(3)	107.31(12)
C(2)-C(1)-C(6)	120.61(15)
C(2)-C(1)-H(1)	119.7
C(6)-C(1)-H(1)	119.7
C(3)-C(2)-C(1)	118.39(15)
C(3)-C(2)-H(2)	120.8
C(1)-C(2)-H(2)	120.8
C(2)-C(3)-C(4)	122.81(14)
C(2)-C(3)-N(1)	118.02(14)
C(4)-C(3)-N(1)	119.17(14)
C(5)-C(4)-C(3)	118.44(15)
C(5)-C(4)-H(4)	120.8
C(3)-C(4)-H(4)	120.8
C(4)-C(5)-C(6)	120.01(15)
C(4)-C(5)-H(5)	120.0
C(6)-C(5)-H(5)	120.0
C(1)-C(6)-C(5)	119.74(14)
C(1)-C(6)-C(7)	117.50(14)
C(5)-C(6)-C(7)	122.77(14)
N(2)-C(7)-C(6)	122.04(14)
N(2)-C(7)-H(7)	119.0
C(6)-C(7)-H(7)	119.0
F(1)-C(8)-F(3)	108.28(15)
F(1)-C(8)-F(2)	106.78(15)
F(3)-C(8)-F(2)	108.43(15)
F(1)-C(8)-O(3)	113.08(16)
F(3)-C(8)-O(3)	107.10(13)
F(2)-C(8)-O(3)	113.01(15)

Symmetry transformations used to generate equivalent atoms:

Supplementary Table 16. Anisotropic displacement parameters (A^2 x 10^3) for R20180423A1.

The anisotropic displacement factor exponent takes the form:

-2 pi^2 [h^2 a*^2 U11 + ... + 2 h k a* b* U12]

	U11	U22	U33	U23	U13	U12
F(1)	67(1)	64(1)	52(1)	15(1)	27(1)	10(1)
F(2)	61(1)	66(1)	58(1)	-30(1)	-18(1)	12(1)
F(3)	56(1)	68(1)	39(1)	-6(1)	4(1)	36(1)
O(1)	41(1)	34(1)	55(1)	-16(1)	9(1)	0(1)
O(2)	43(1)	45(1)	43(1)	14(1)	10(1)	19(1)
O(3)	38(1)	44(1)	26(1)	-1(1)	2(1)	19(1)
N(1)	30(1)	25(1)	42(1)	6(1)	12(1)	0(1)
N(2)	28(1)	32(1)	32(1)	-1(1)	1(1)	10(1)
C(1)	29(1)	33(1)	28(1)	1(1)	-1(1)	2(1)
C(2)	32(1)	31(1)	28(1)	-4(1)	6(1)	-1(1)
C(3)	23(1)	21(1)	35(1)	1(1)	8(1)	-1(1)
C(4)	26(1)	31(1)	32(1)	4(1)	2(1)	1(1)
C(5)	29(1)	32(1)	26(1)	-2(1)	2(1)	1(1)
C(6)	23(1)	24(1)	28(1)	-1(1)	3(1)	-2(1)
C(7)	26(1)	31(1)	26(1)	2(1)	0(1)	2(1)
C(8)	36(1)	42(1)	28(1)	-4(1)	1(1)	10(1)

Supplementary Table 17. Hydrogen coordinates (x 10^4) and isotropic displacement parameters (A^2 x 10^3) for R20180423A1.

	x	y	z	U(eq)
H(1)	7156	6870	2579	36
H(2)	8210	3574	2112	36
H(4)	9217	3421	5117	36
H(5)	8163	6738	5584	35
H(7)	6519	9936	3803	34

Supplementary Table 18. Torsion angles [deg] for R20180423A1.

z

C(8)-O(3)-N(2)-C(7)	172.79(15)
C(6)-C(1)-C(2)-C(3)	0.2(2)
C(1)-C(2)-C(3)-C(4)	-0.6(2)
C(1)-C(2)-C(3)-N(1)	179.16(14)
O(2)-N(1)-C(3)-C(2)	167.34(14)
O(1)-N(1)-C(3)-C(2)	-12.5(2)
O(2)-N(1)-C(3)-C(4)	-12.9(2)
O(1)-N(1)-C(3)-C(4)	167.25(14)
C(2)-C(3)-C(4)-C(5)	0.5(2)
N(1)-C(3)-C(4)-C(5)	-179.26(14)
C(3)-C(4)-C(5)-C(6)	0.0(2)
C(2)-C(1)-C(6)-C(5)	0.3(2)
C(2)-C(1)-C(6)-C(7)	-179.25(15)
C(4)-C(5)-C(6)-C(1)	-0.4(2)
C(4)-C(5)-C(6)-C(7)	179.11(14)
O(3)-N(2)-C(7)-C(6)	-179.43(13)
C(1)-C(6)-C(7)-N(2)	171.94(16)
C(5)-C(6)-C(7)-N(2)	-7.6(2)
N(2)-O(3)-C(8)-F(1)	-57.92(18)
N(2)-O(3)-C(8)-F(3)	-177.12(13)
N(2)-O(3)-C(8)-F(2)	63.54(19)

Symmetry transformations used to generate equivalent atoms:

Supplementary Figure

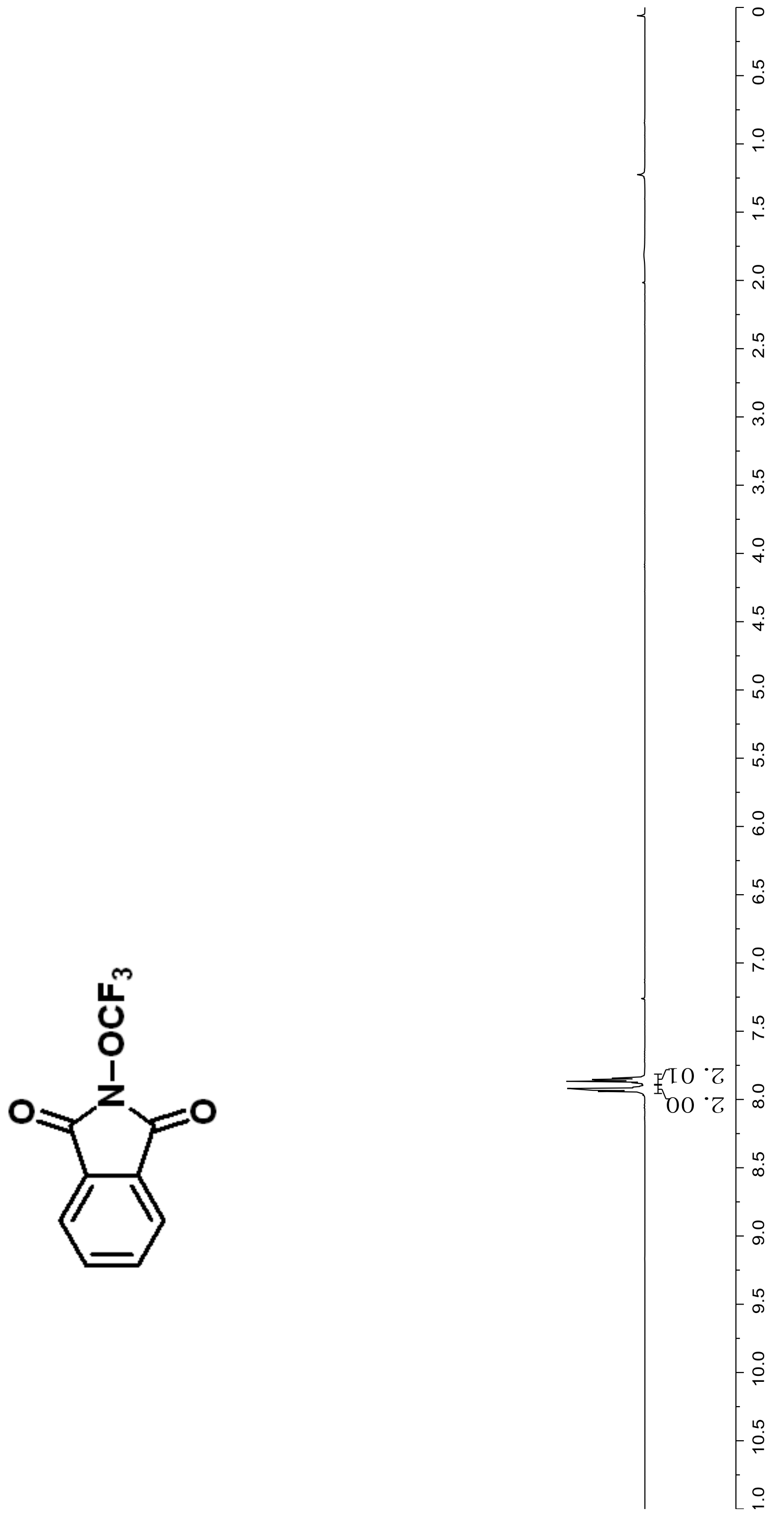

Supplementary Figure 2: ^{1}H NMR spectrum (400 MHz, $CDCl_3$, 23 °C) of **S1**

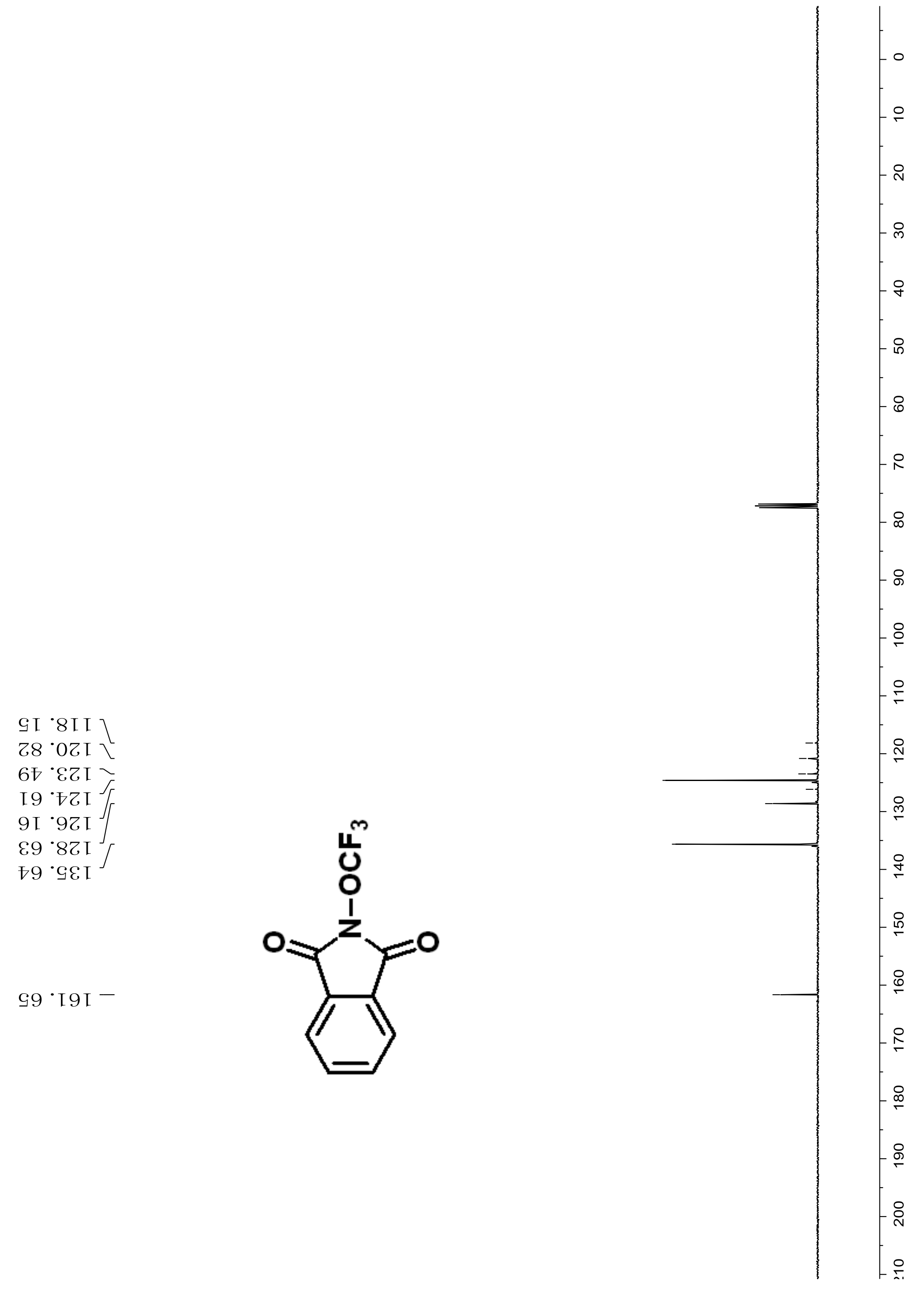

Supplementary Figure 3: ^{13}C NMR spectrum (101 MHz, $CDCl_3$, 23 °C) of **S1**

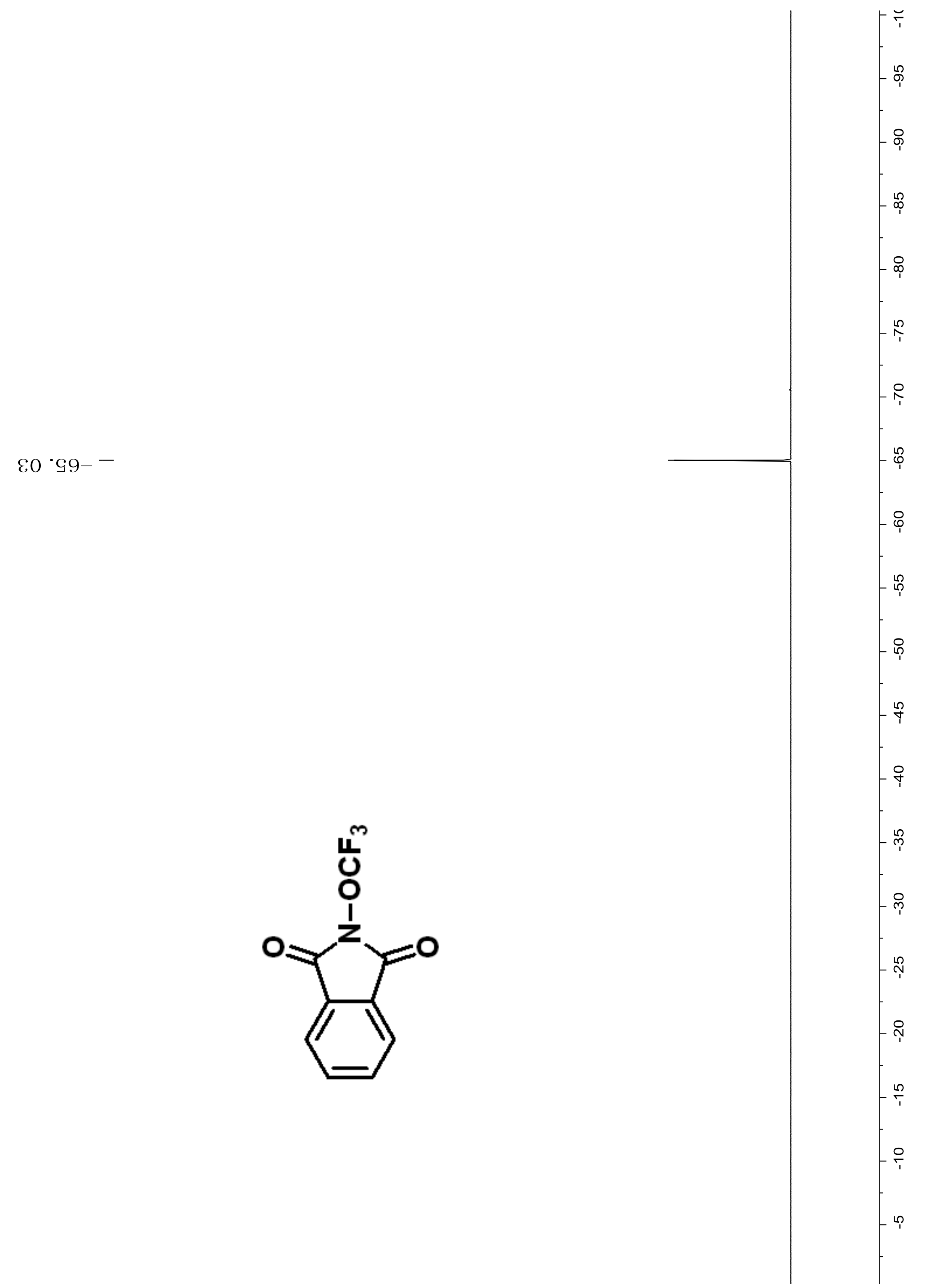

Supplementary Figure 4: ^{19}F NMR spectrum (376 MHz, $CDCl_3$, 23 °C) of **S1**

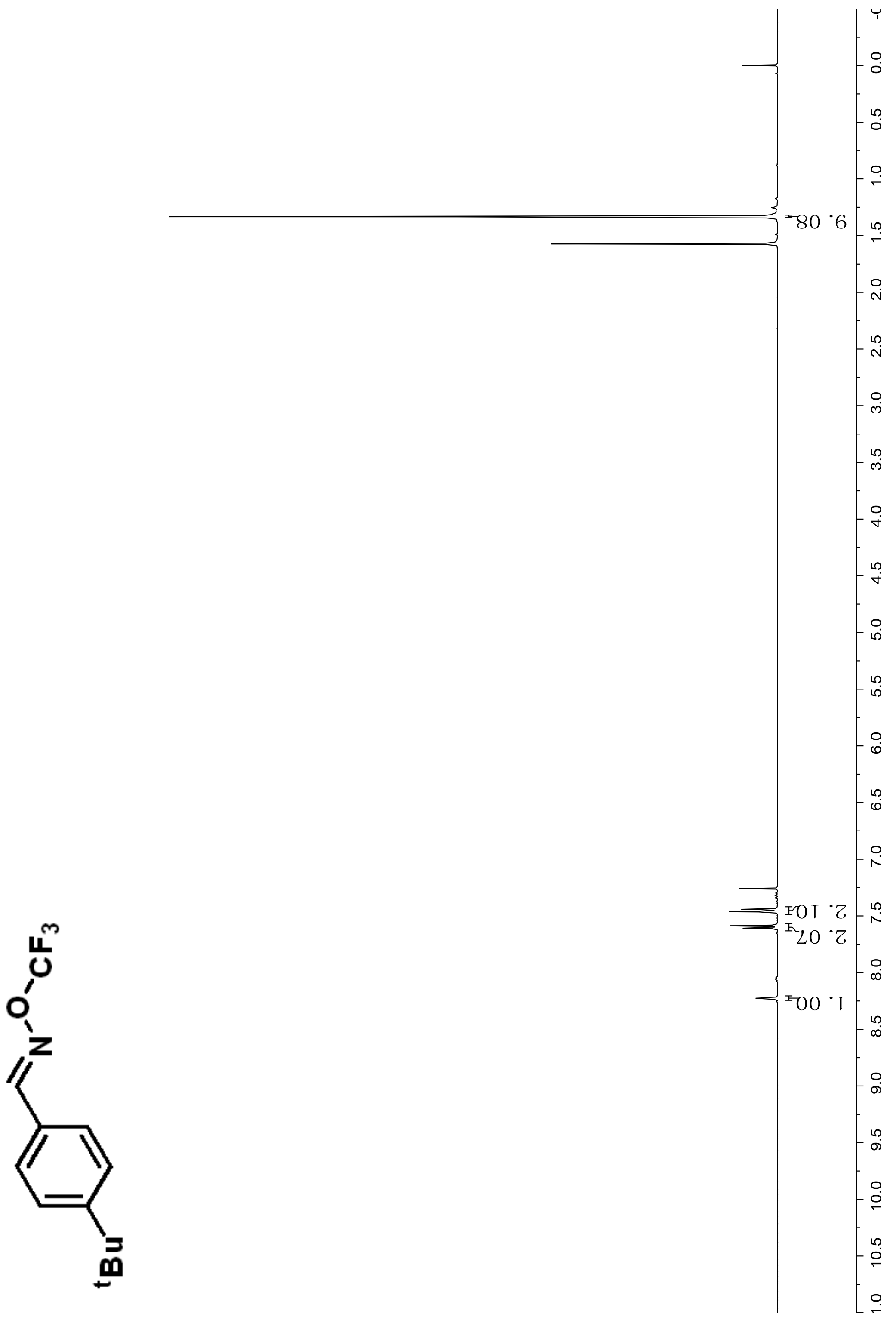

Supplementary Figure 5: ^{1}H NMR spectrum (400 MHz, $CDCl_3$, 23 °C) of **1a**

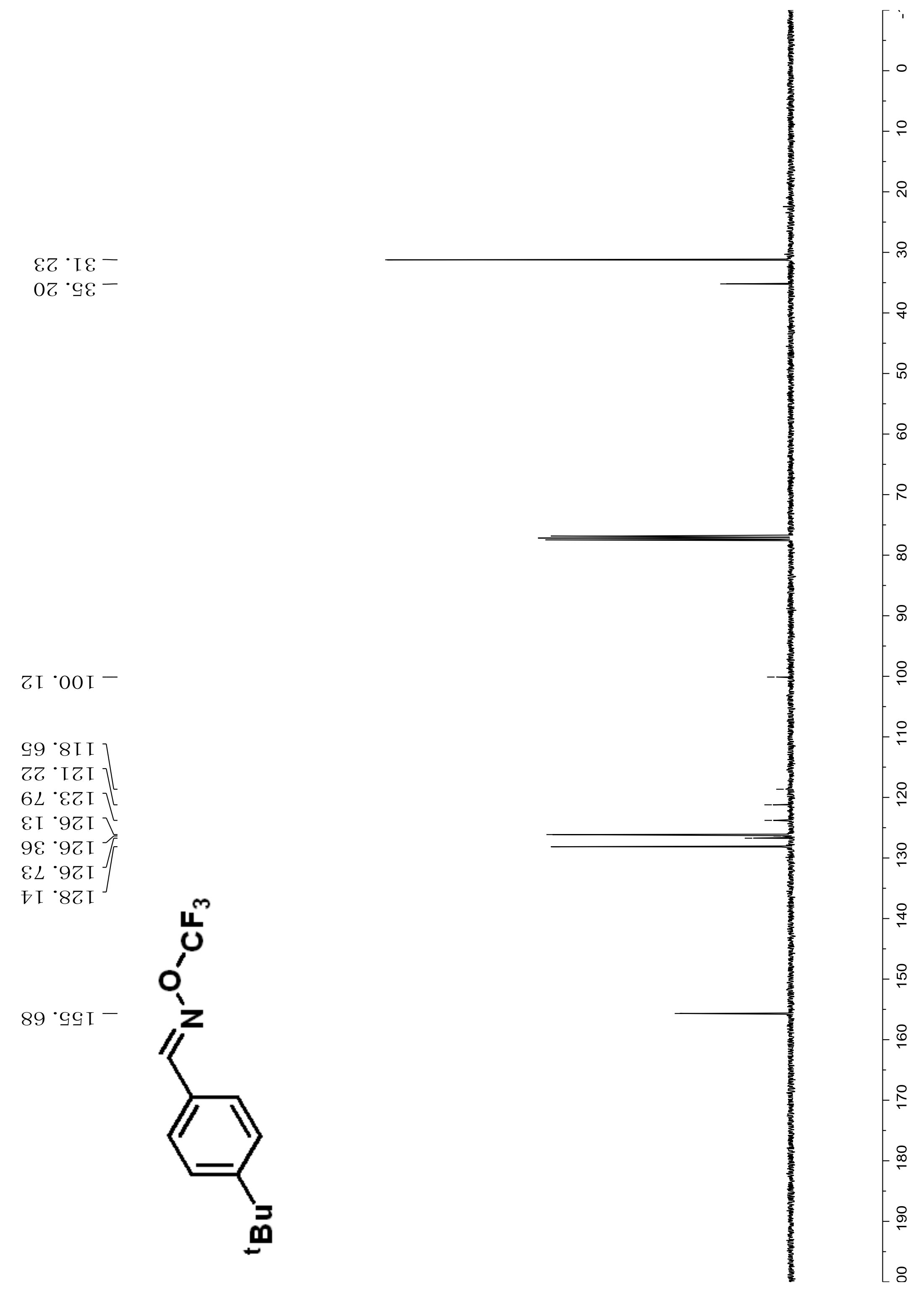

Supplementary Figure 6: ^{13}C NMR spectrum (101 MHz, $CDCl_3$, 23 °C) of **1a**

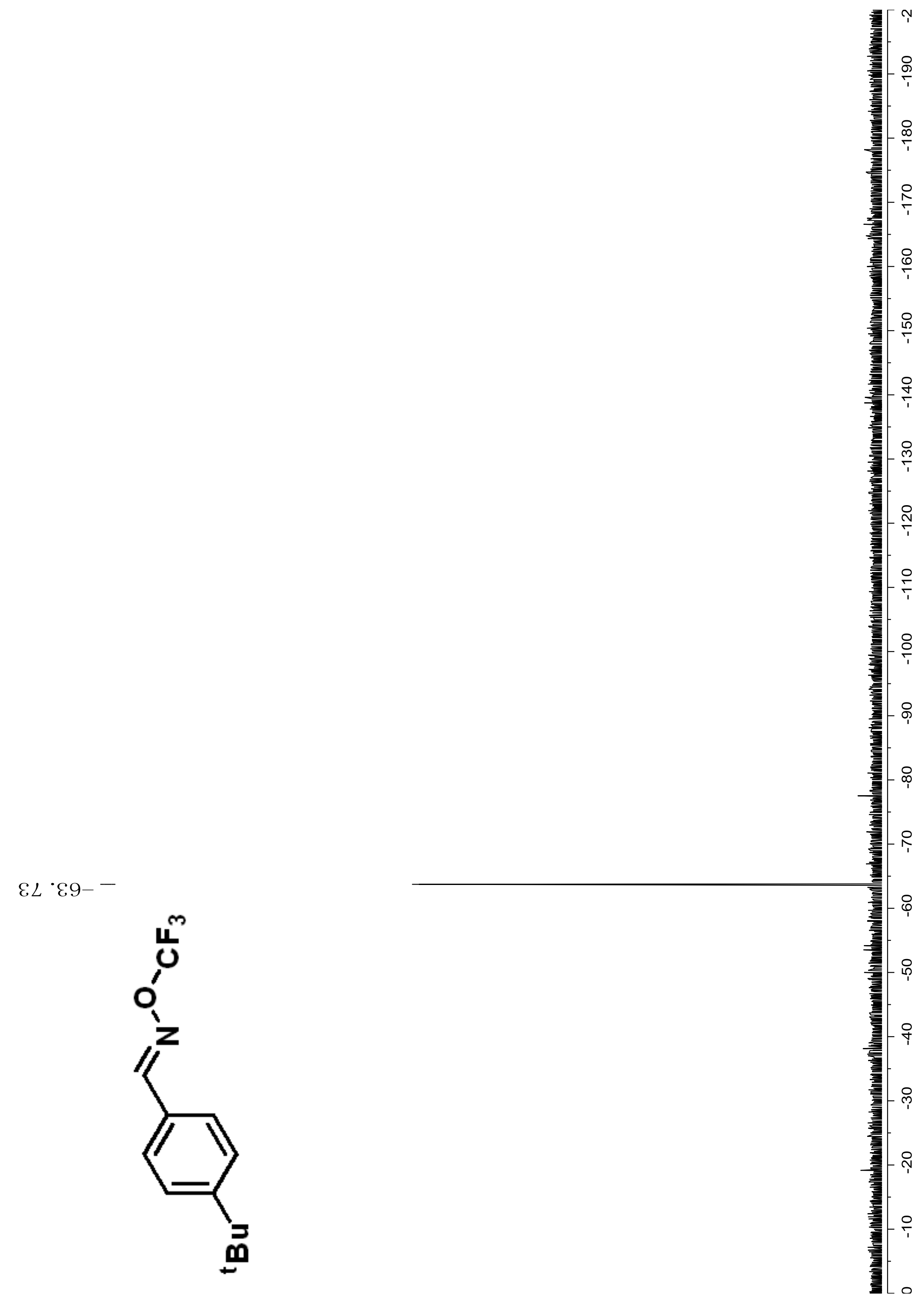

Supplementary Figure 7: ^{19}F NMR spectrum (376 MHz, $CDCl_3$, 23 °C) of **1a**

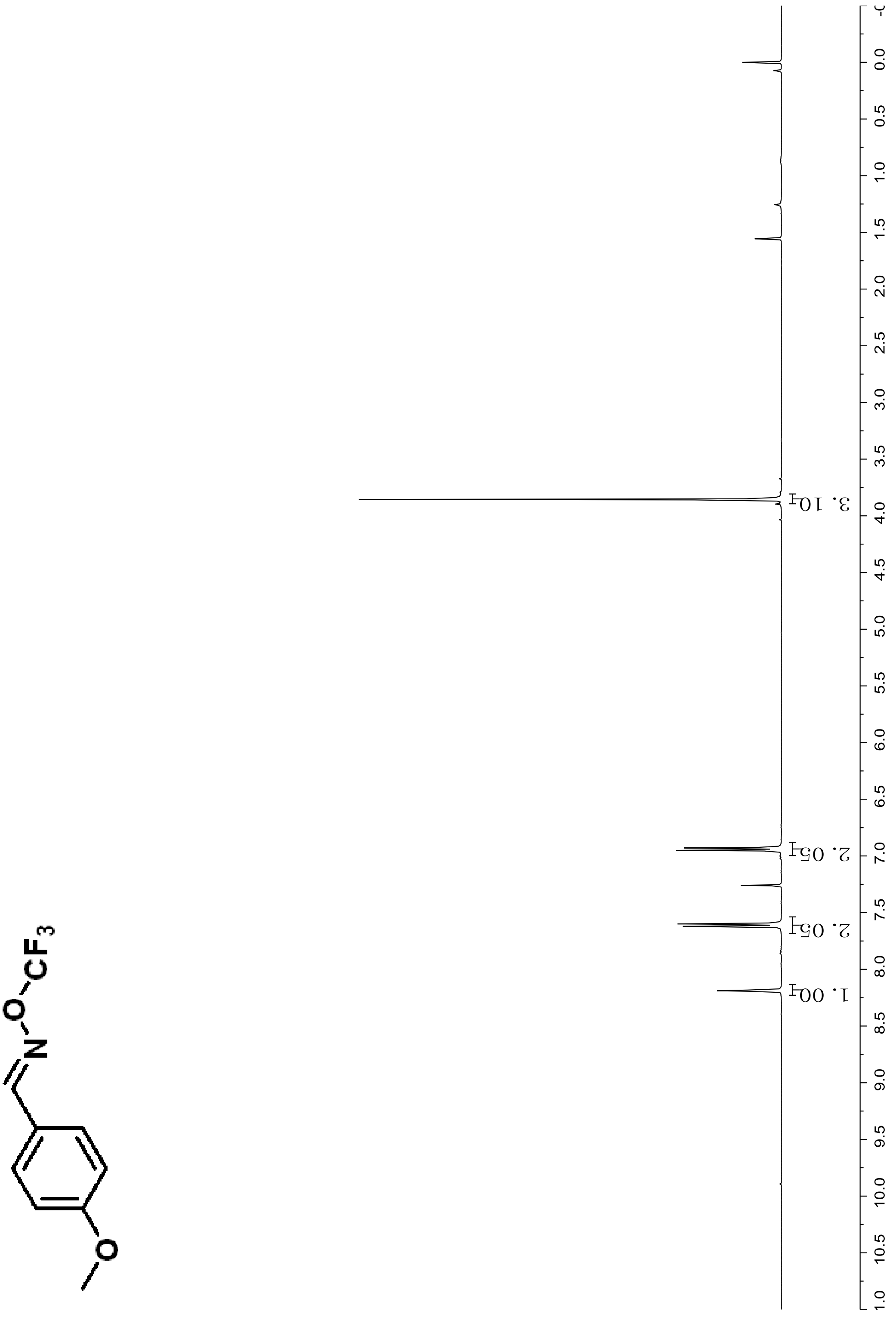

Supplementary Figure 8: ^{1}H NMR spectrum (400 MHz, $CDCl_3$, 23 °C) of **1b**

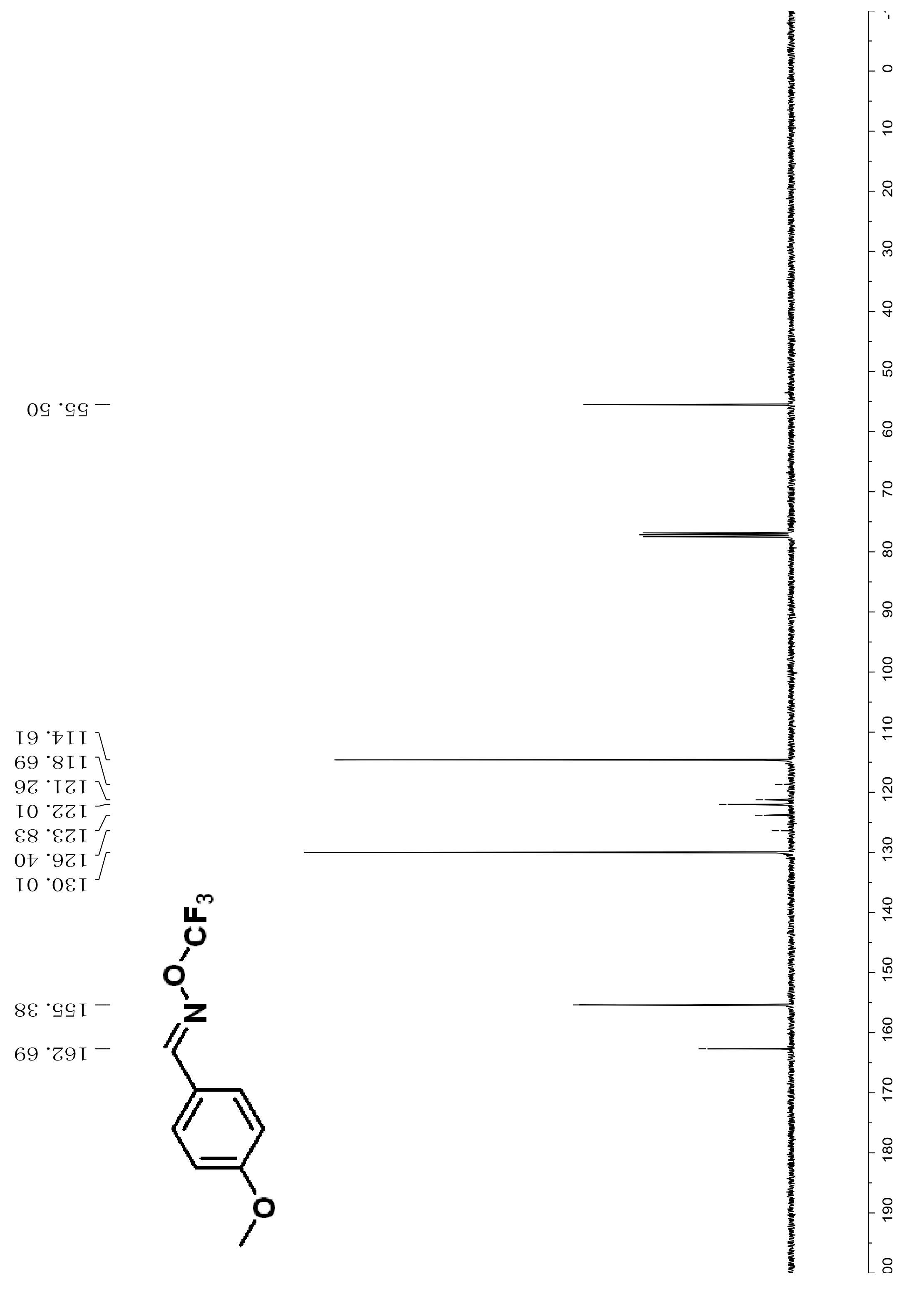

Supplementary Figure 9: ^{13}C NMR spectrum (101 MHz, $CDCl_3$, 23 °C) of **1b**

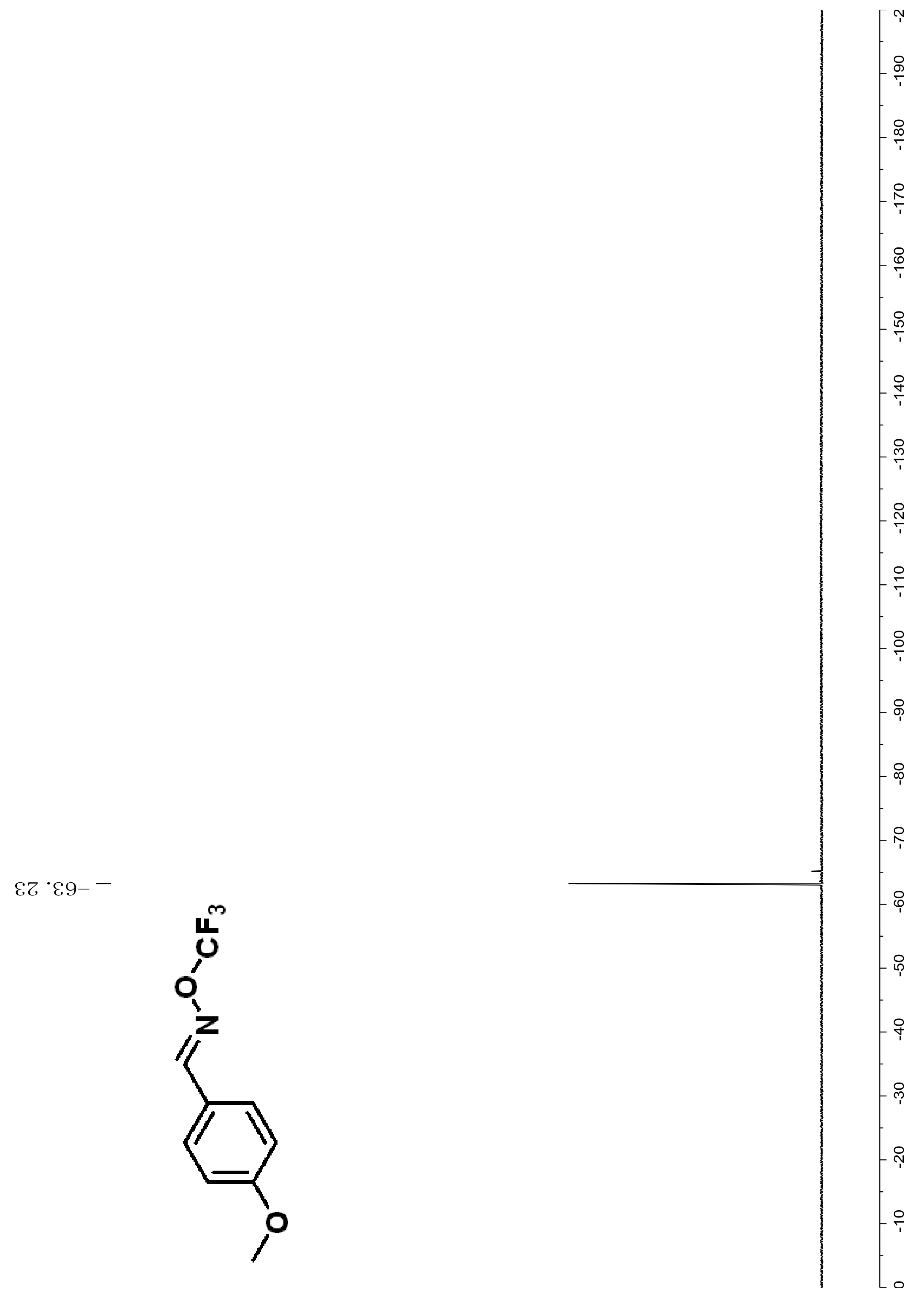

Supplementary Figure 10: ^{19}F NMR spectrum (376 MHz, $CDCl_3$, 23 °C) of **1b**

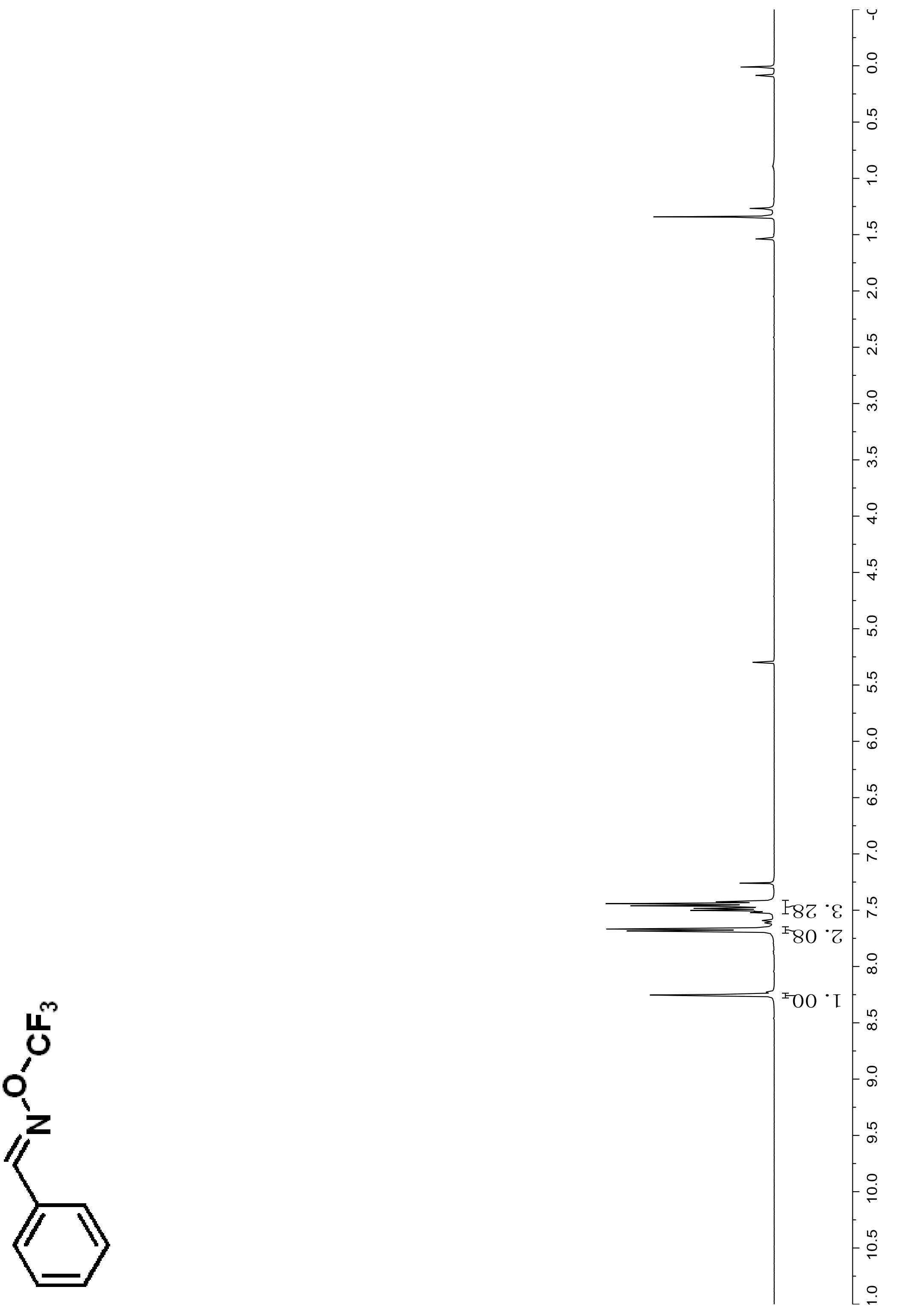

Supplementary Figure 11: ^{1}H NMR spectrum (400 MHz, $CDCl_3$, 23 °C) of **1c**

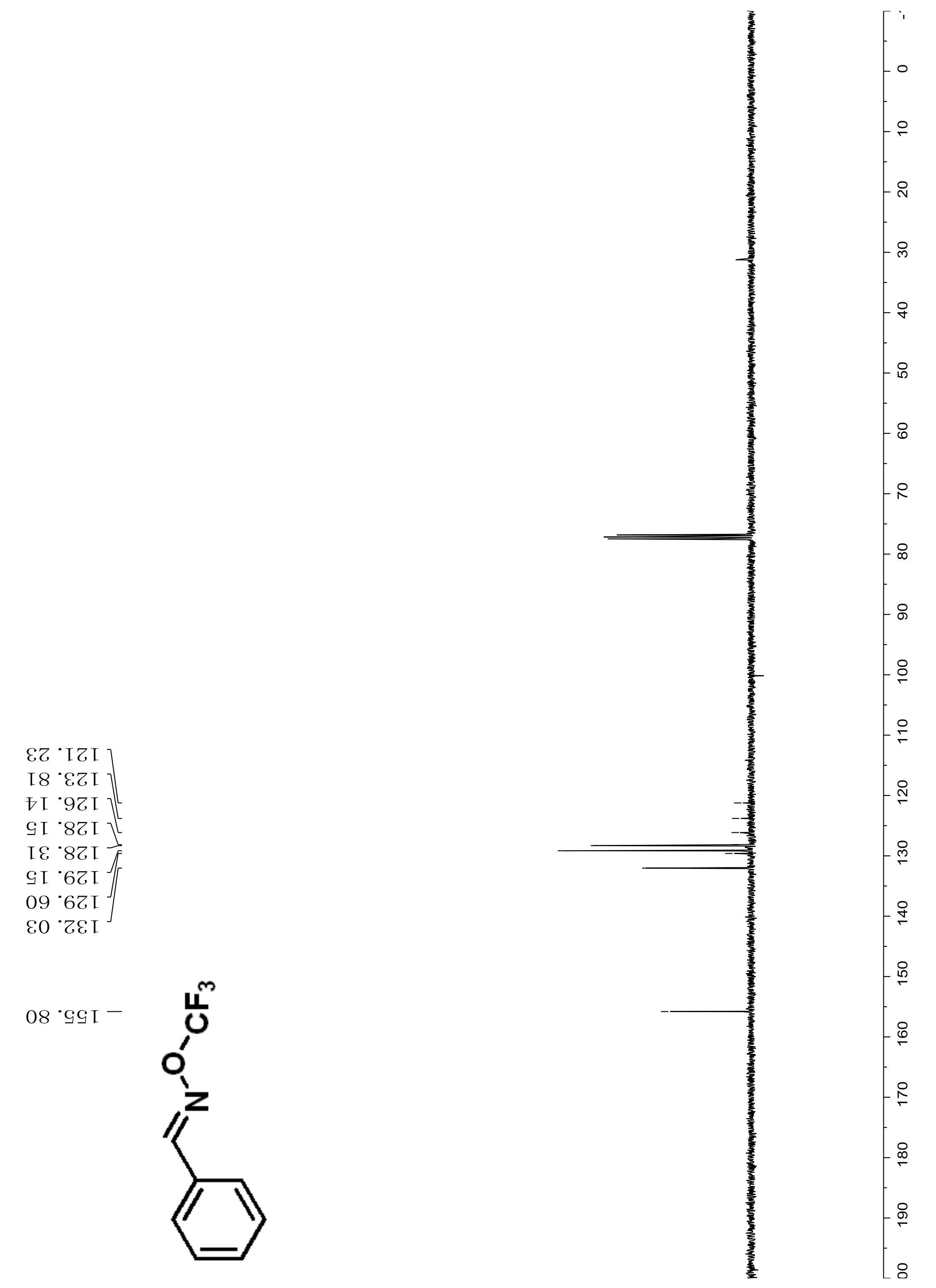

Supplementary Figure 12: ^{13}C NMR spectrum (101 MHz, $CDCl_3$, 23 °C) of **1c**

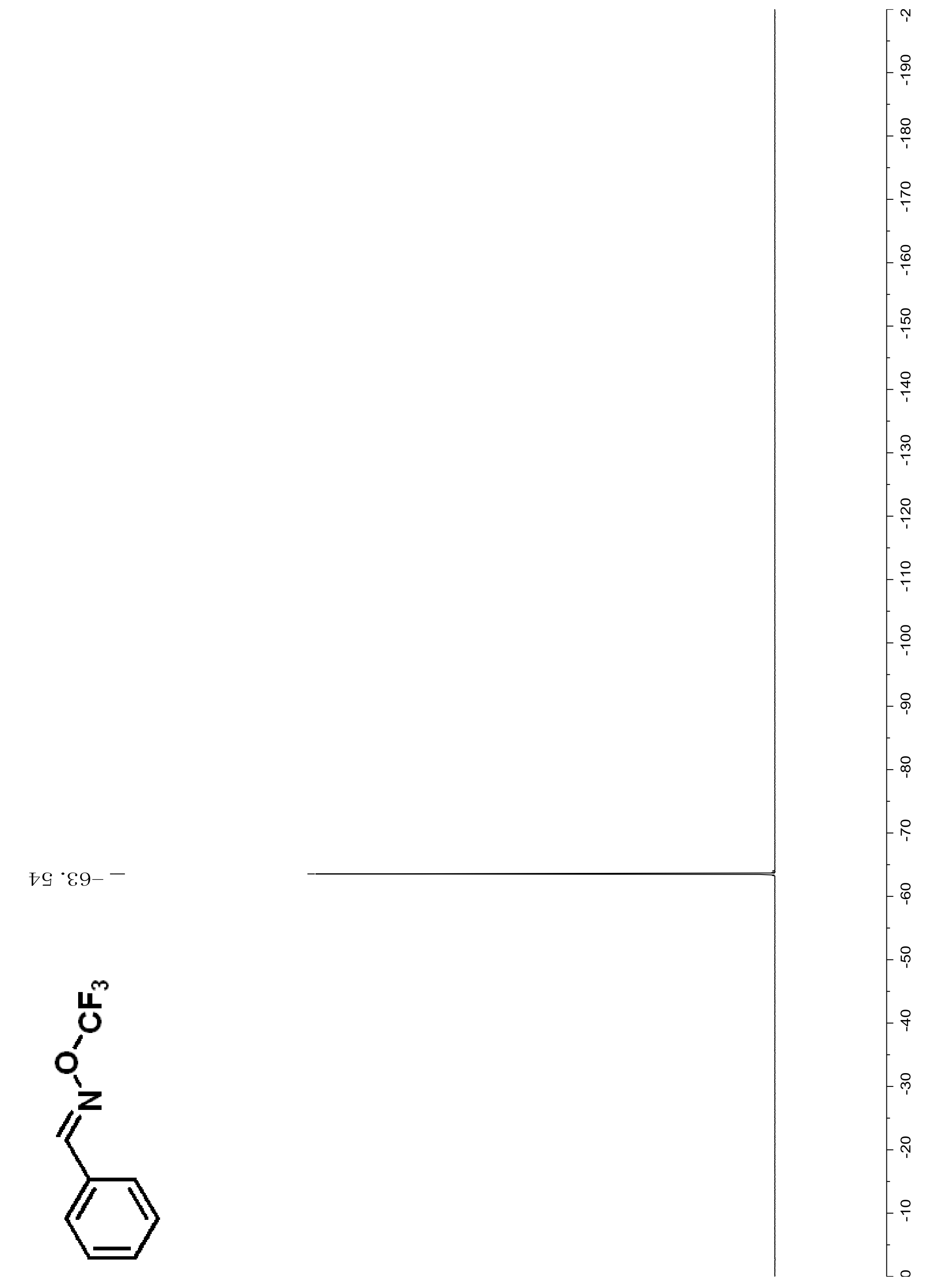

Supplementary Figure 13: ^{19}F NMR spectrum (376 MHz, $CDCl_3$, 23 °C) of **1c**

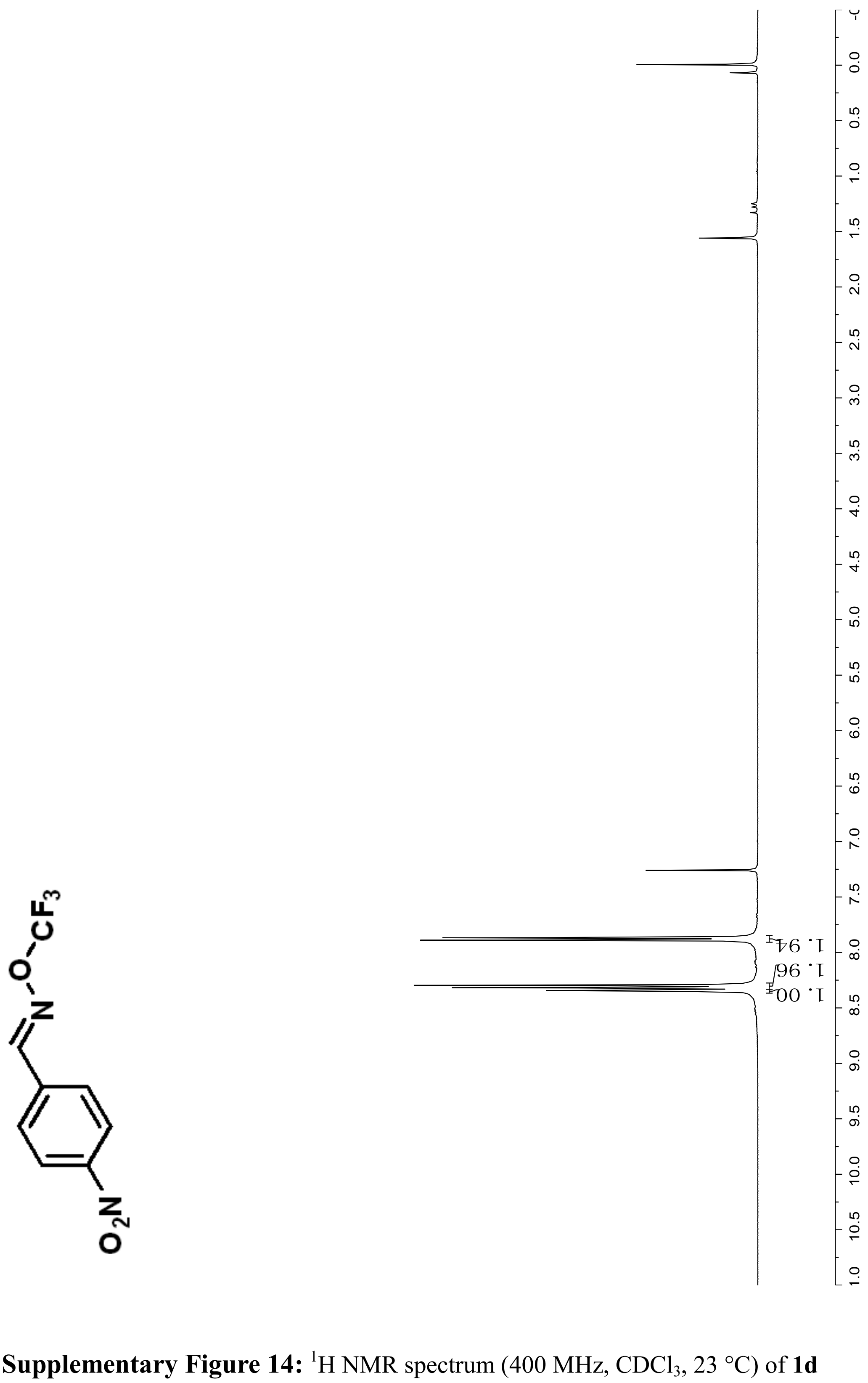

Supplementary Figure 14: ^{1}H NMR spectrum (400 MHz, $CDCl_3$, 23 °C) of **1d**

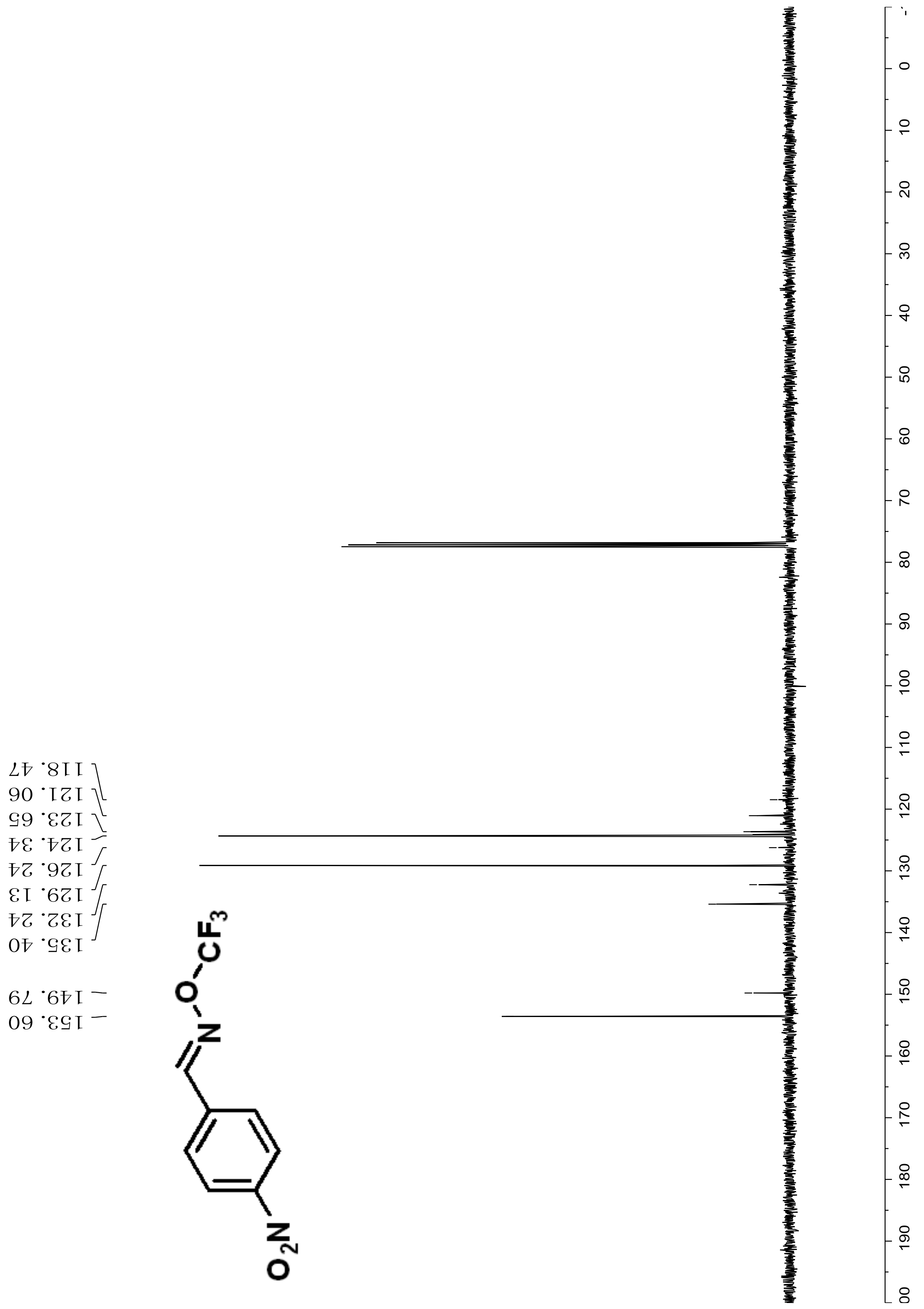

Supplementary Figure 15: ^{13}C NMR spectrum (101 MHz, $CDCl_3$, 23 °C) of **1d**

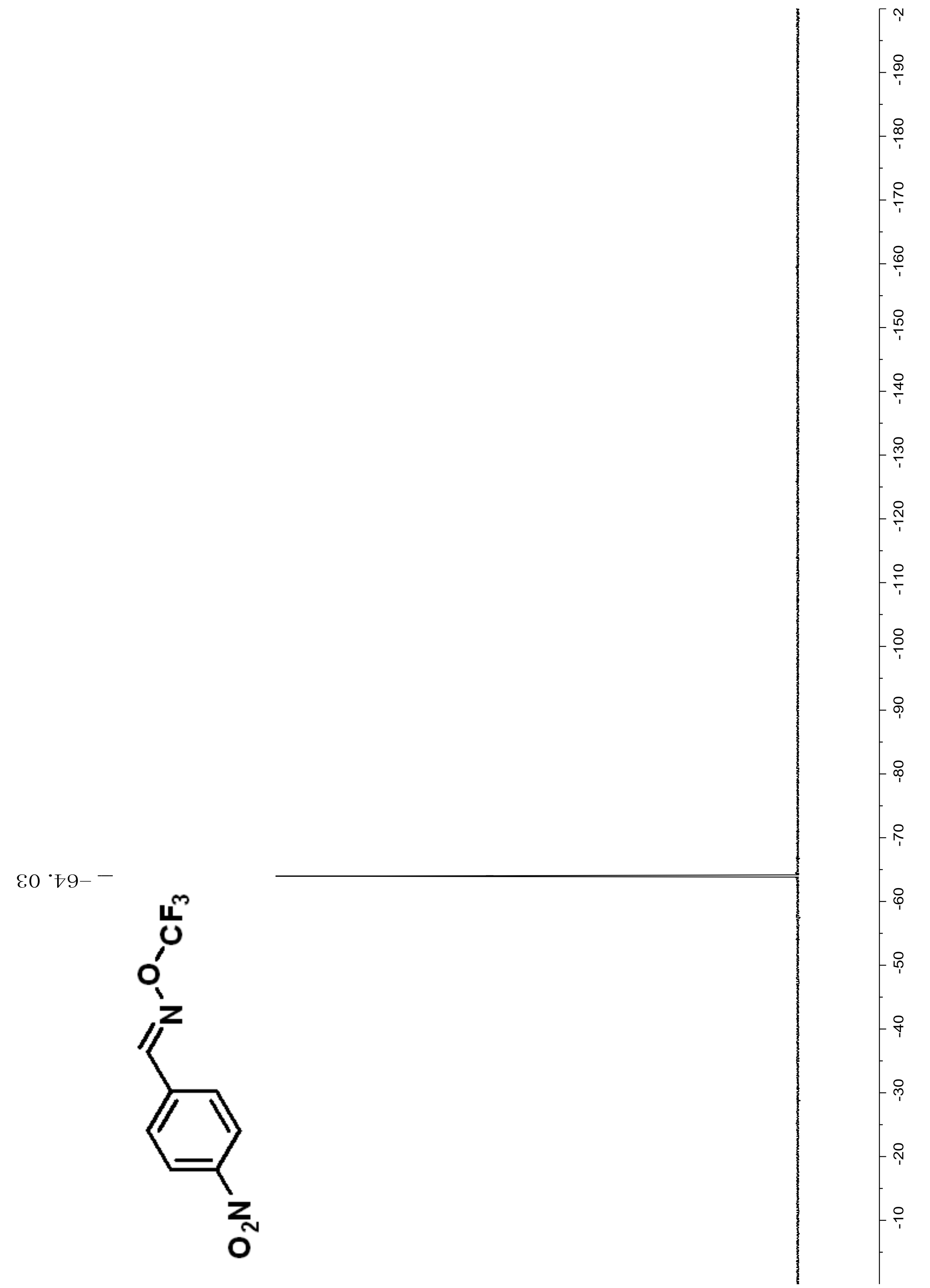

Supplementary Figure 16: ^{19}F NMR spectrum (376 MHz, $CDCl_3$, 23 °C) of **1d**

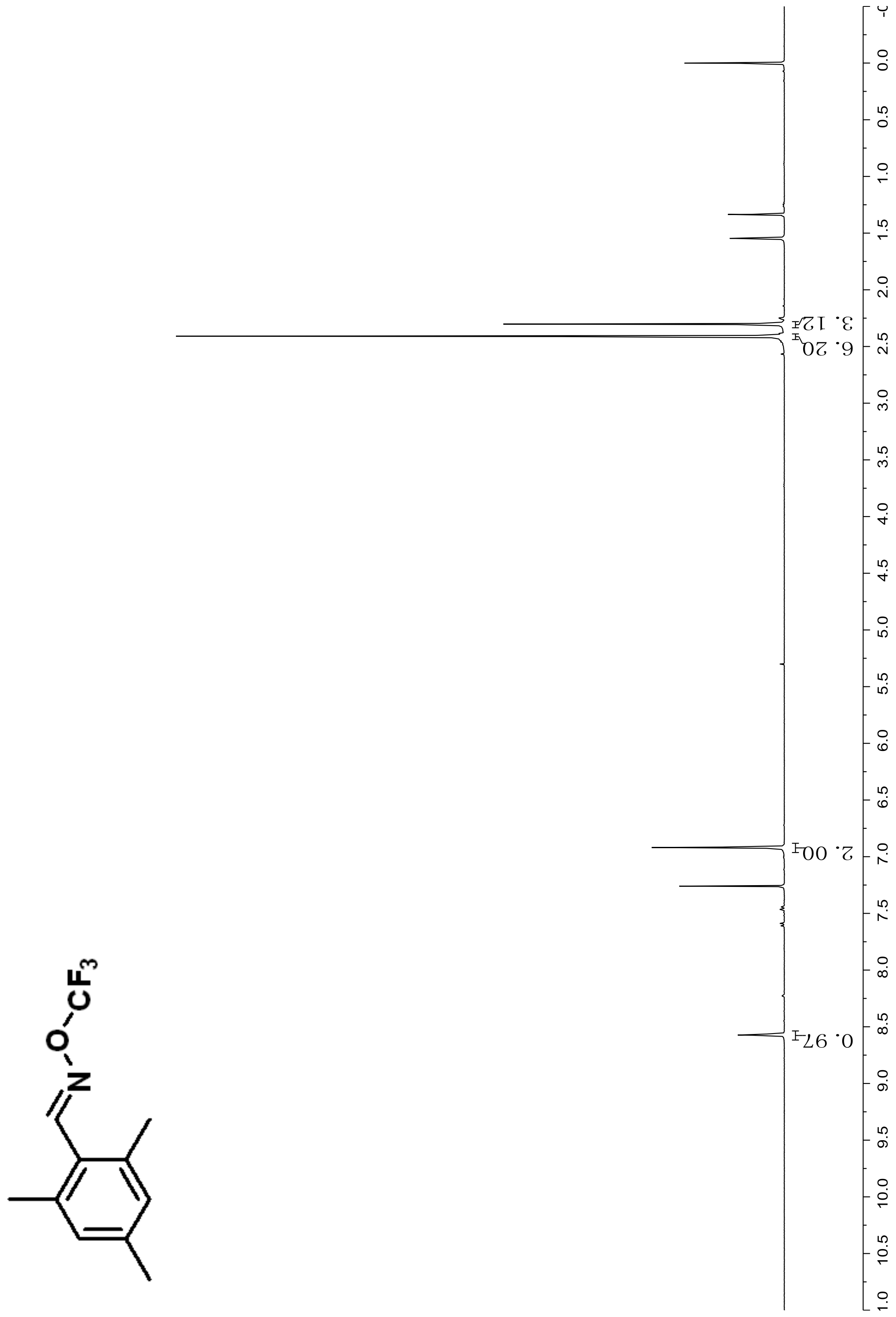

Supplementary Figure 17: ^{1}H NMR spectrum (400 MHz, $CDCl_3$, 23 °C) of **1e**

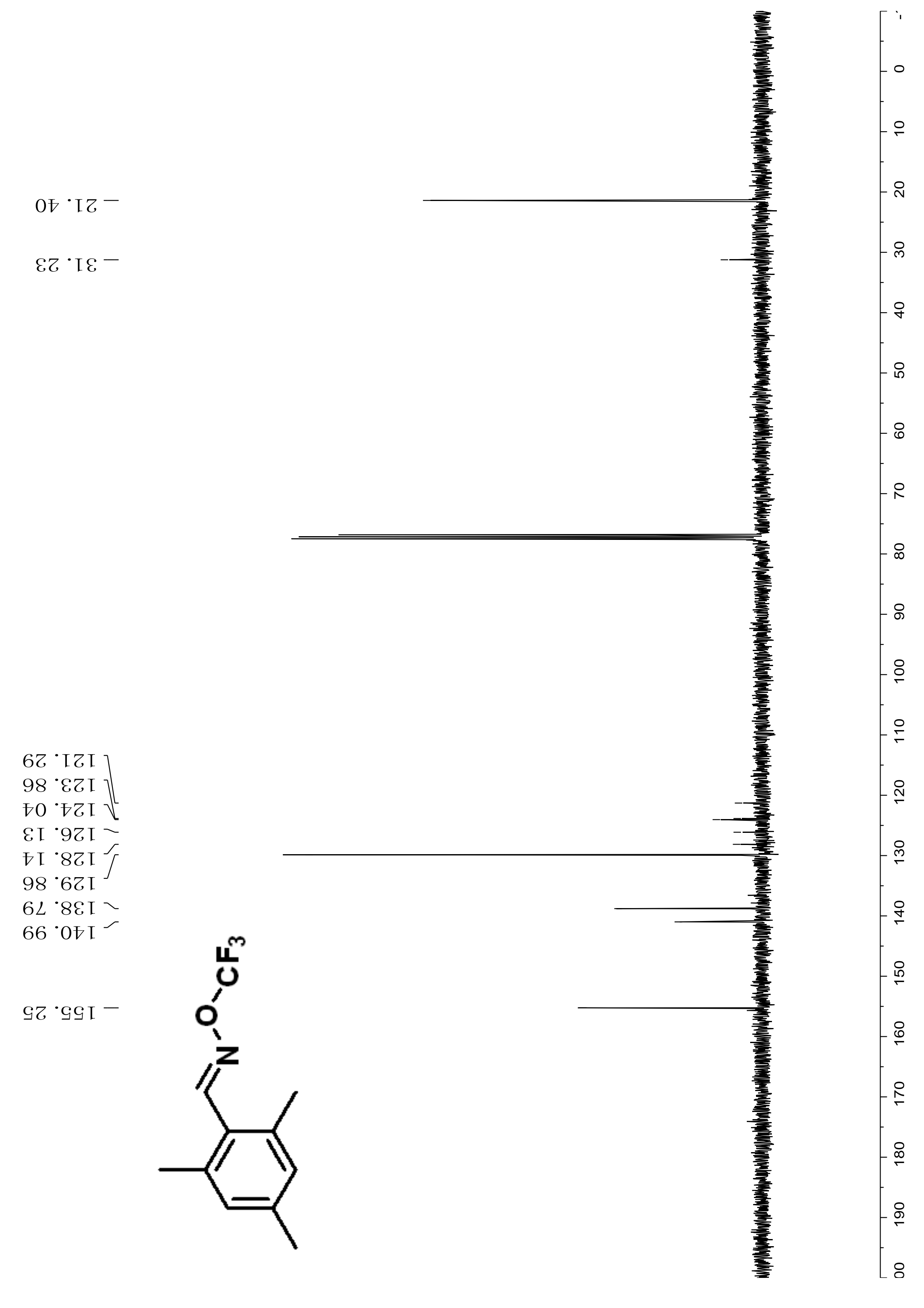

Supplementary Figure 18: ^{13}C NMR spectrum (101 MHz, $CDCl_3$, 23 °C) of **1e**

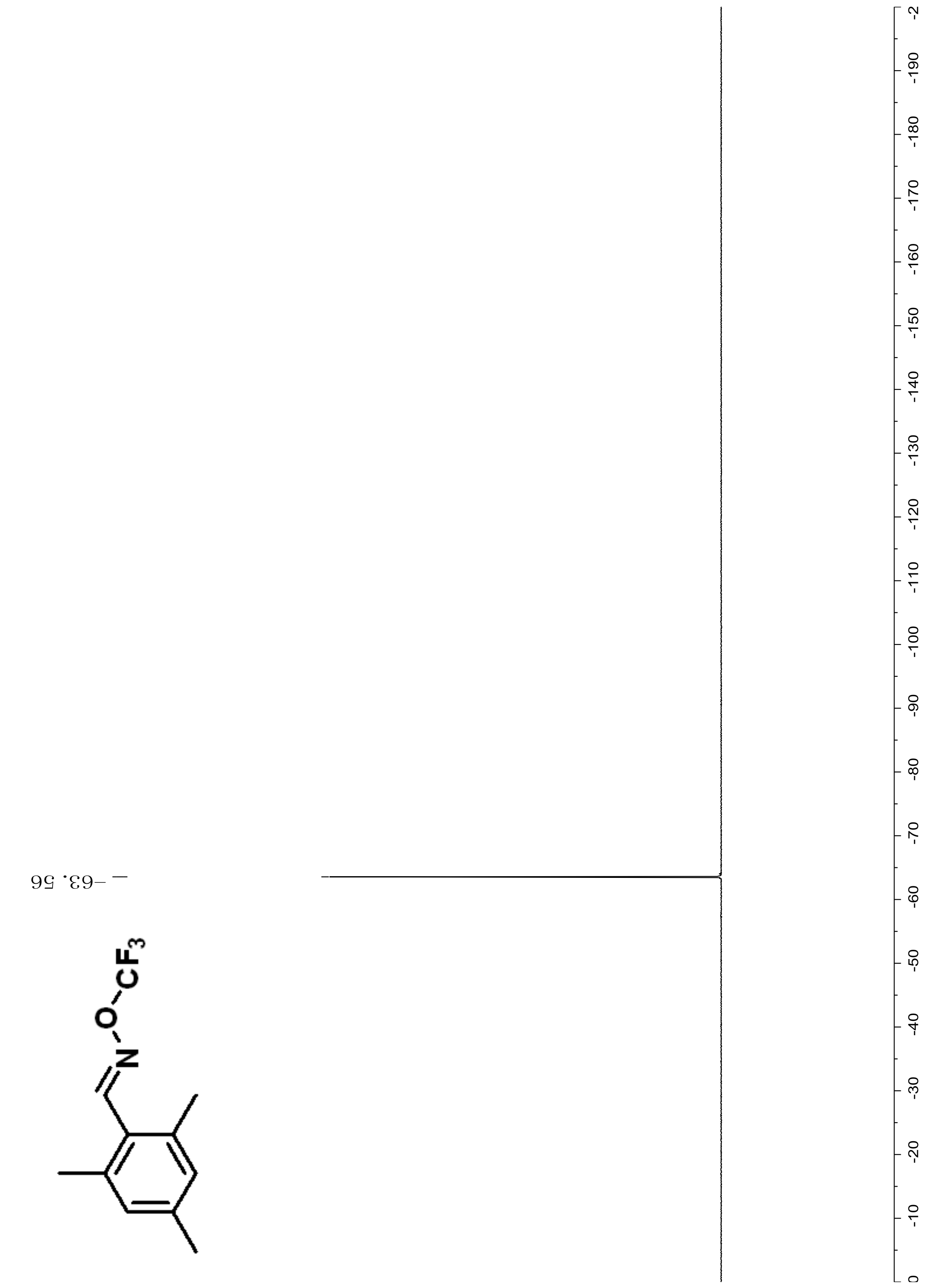

Supplementary Figure 19: ^{19}F NMR spectrum (376 MHz, $CDCl_3$, 23 °C) of **1e**

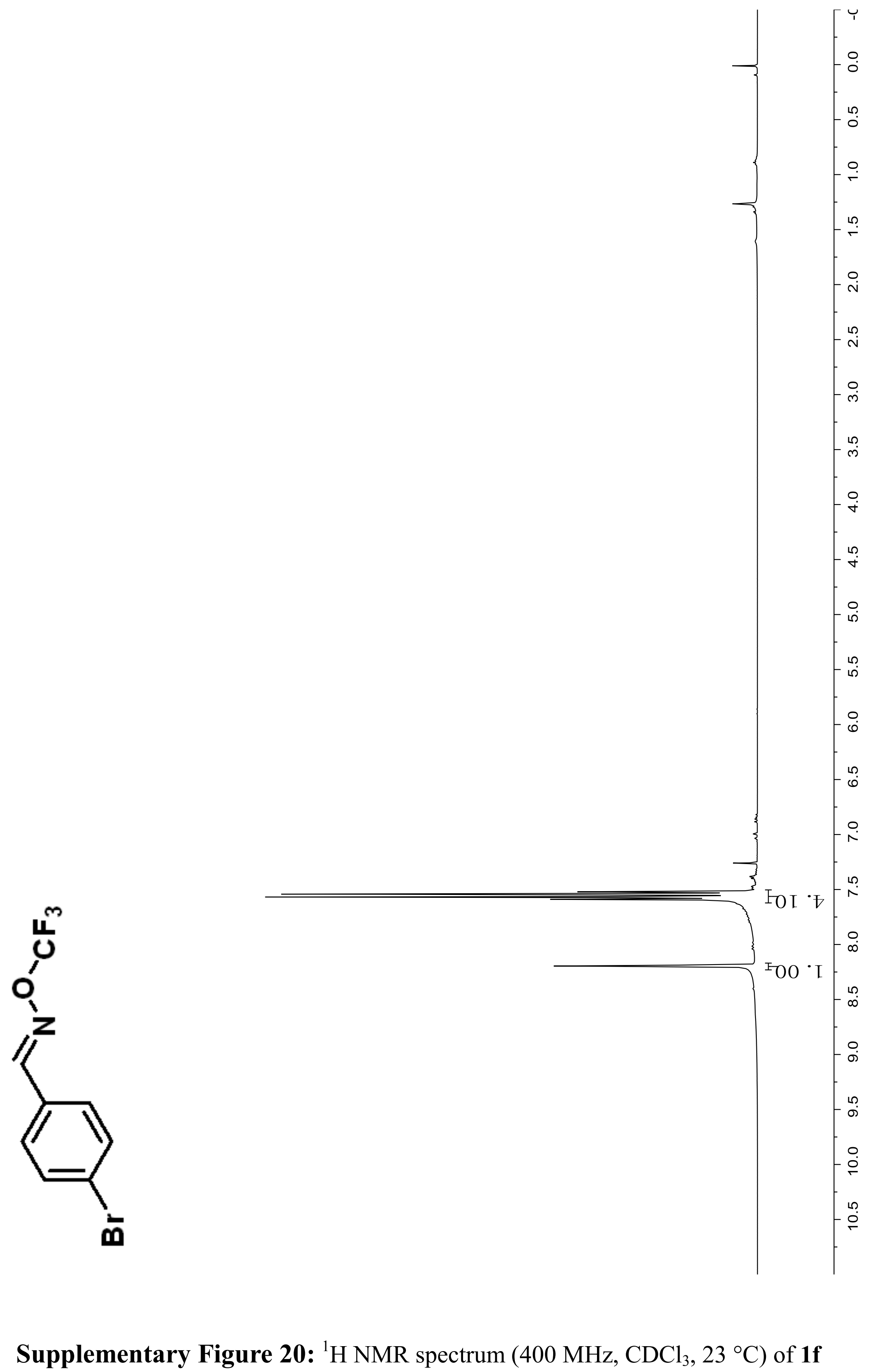

Supplementary Figure 20: ^{1}H NMR spectrum (400 MHz, $CDCl_3$, 23 °C) of **1f**

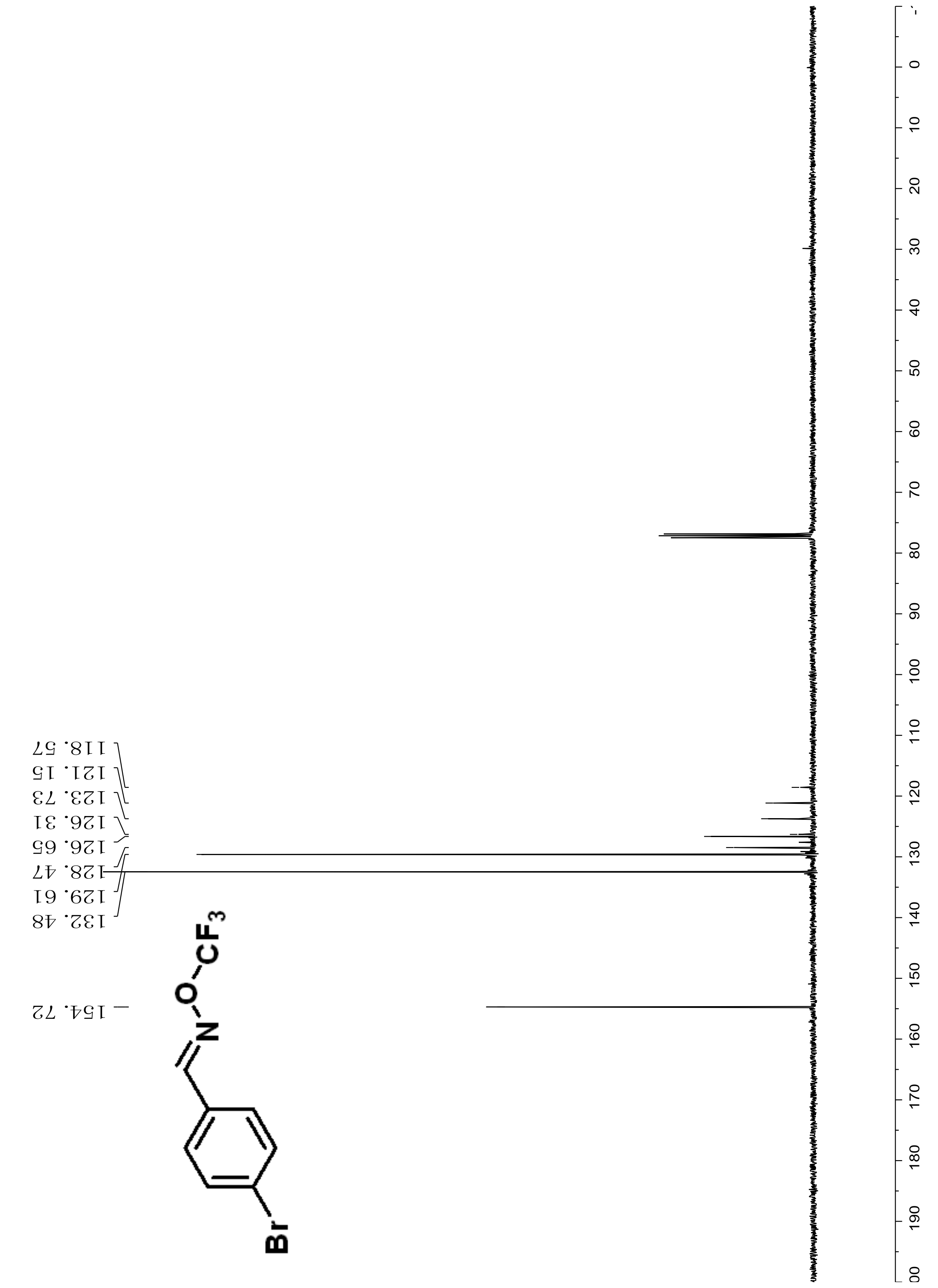

Supplementary Figure 21: ^{13}C NMR spectrum (101 MHz, $CDCl_3$, 23 °C) of **1f**

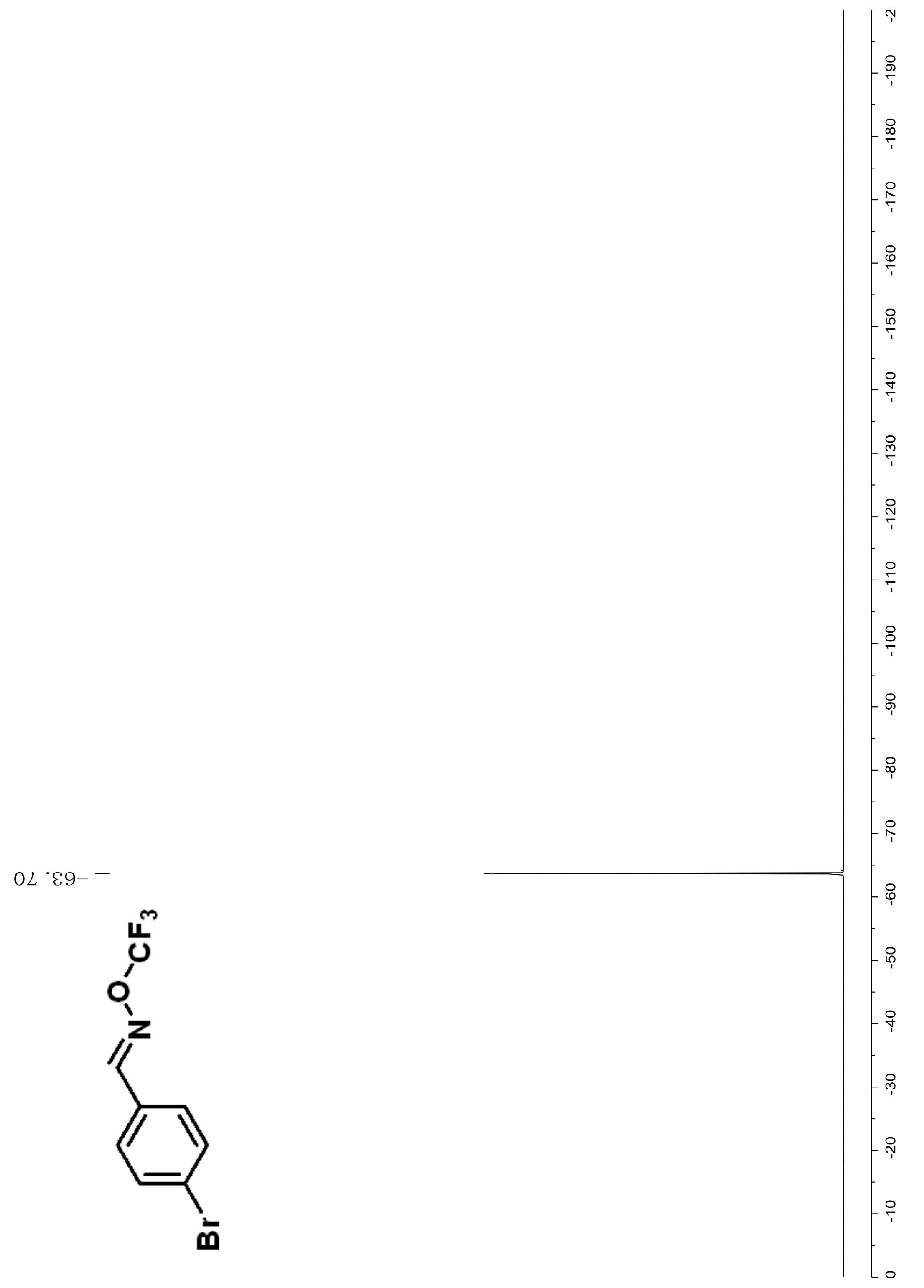

Supplementary Figure 22: ^{19}F NMR spectrum (376 MHz, DMSO, 23 °C) of **1f**

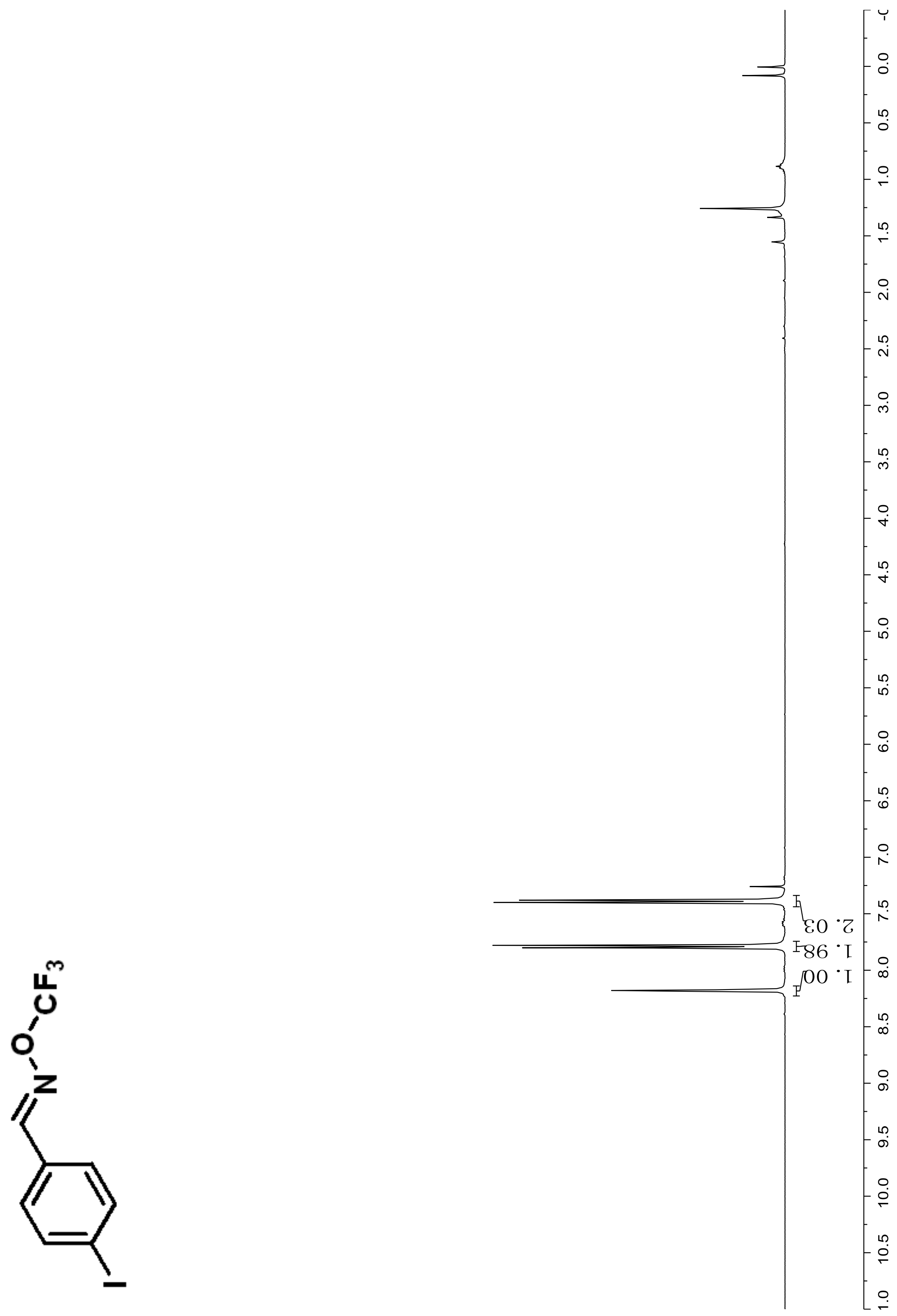

Supplementary Figure 23: ^{1}H NMR spectrum (400 MHz, $CDCl_3$, 23 °C) of **1g**

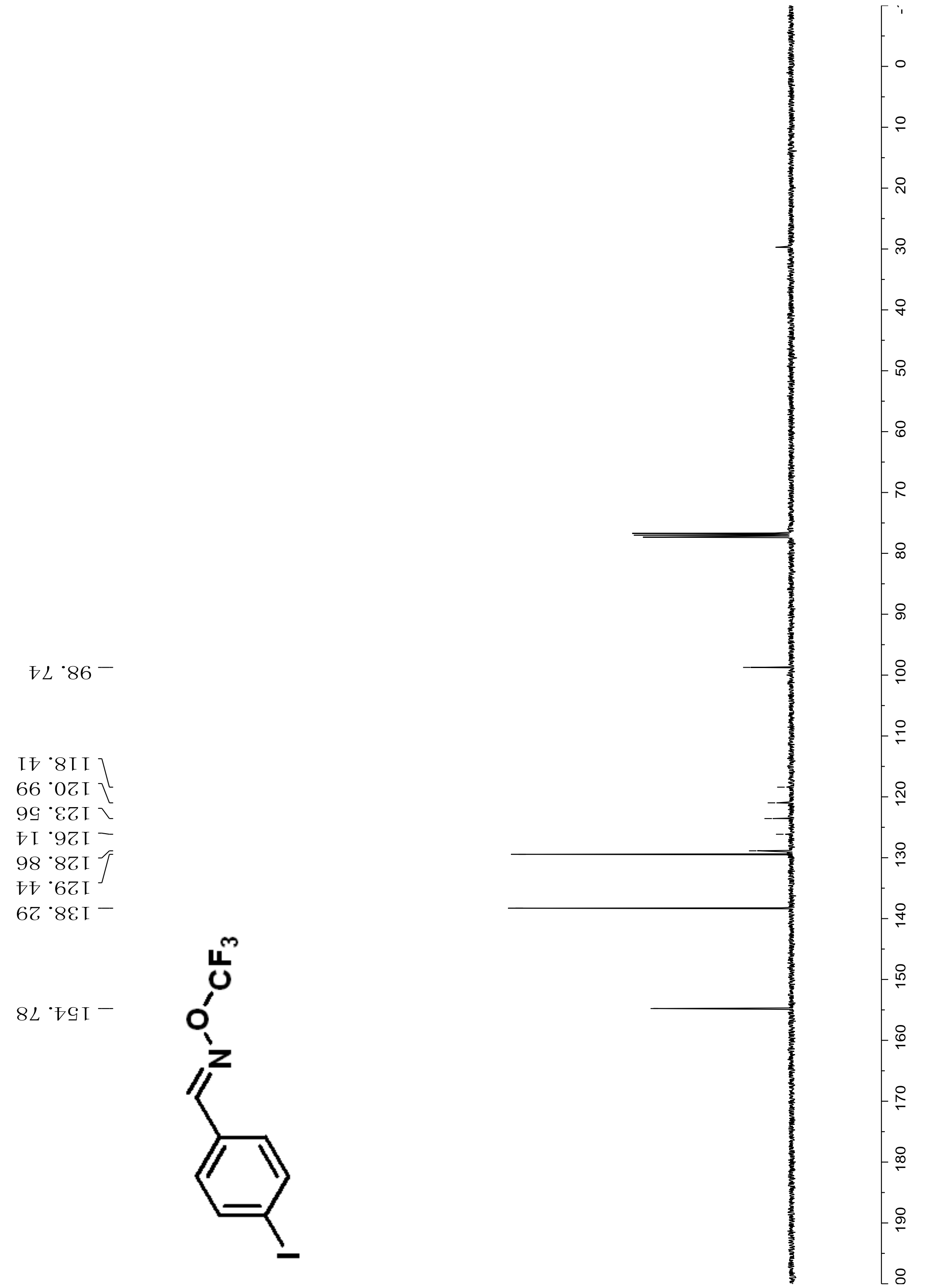

Supplementary Figure 24: ^{13}C NMR spectrum (101 MHz, $CDCl_3$, 23 °C) of **1g**

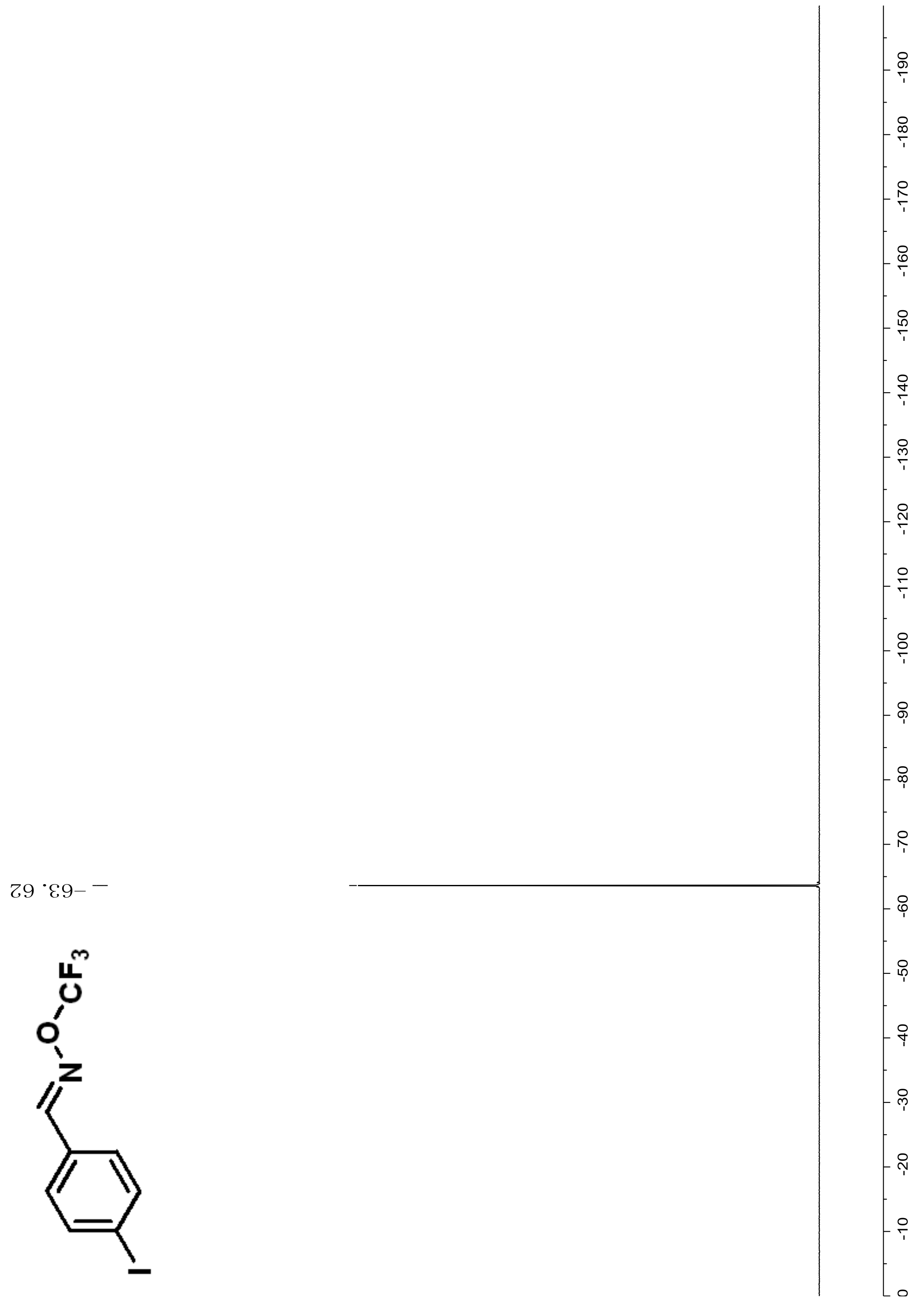

Supplementary Figure 25: ^{19}F NMR spectrum (376 MHz, $CDCl_3$, 23 °C) of **1g**

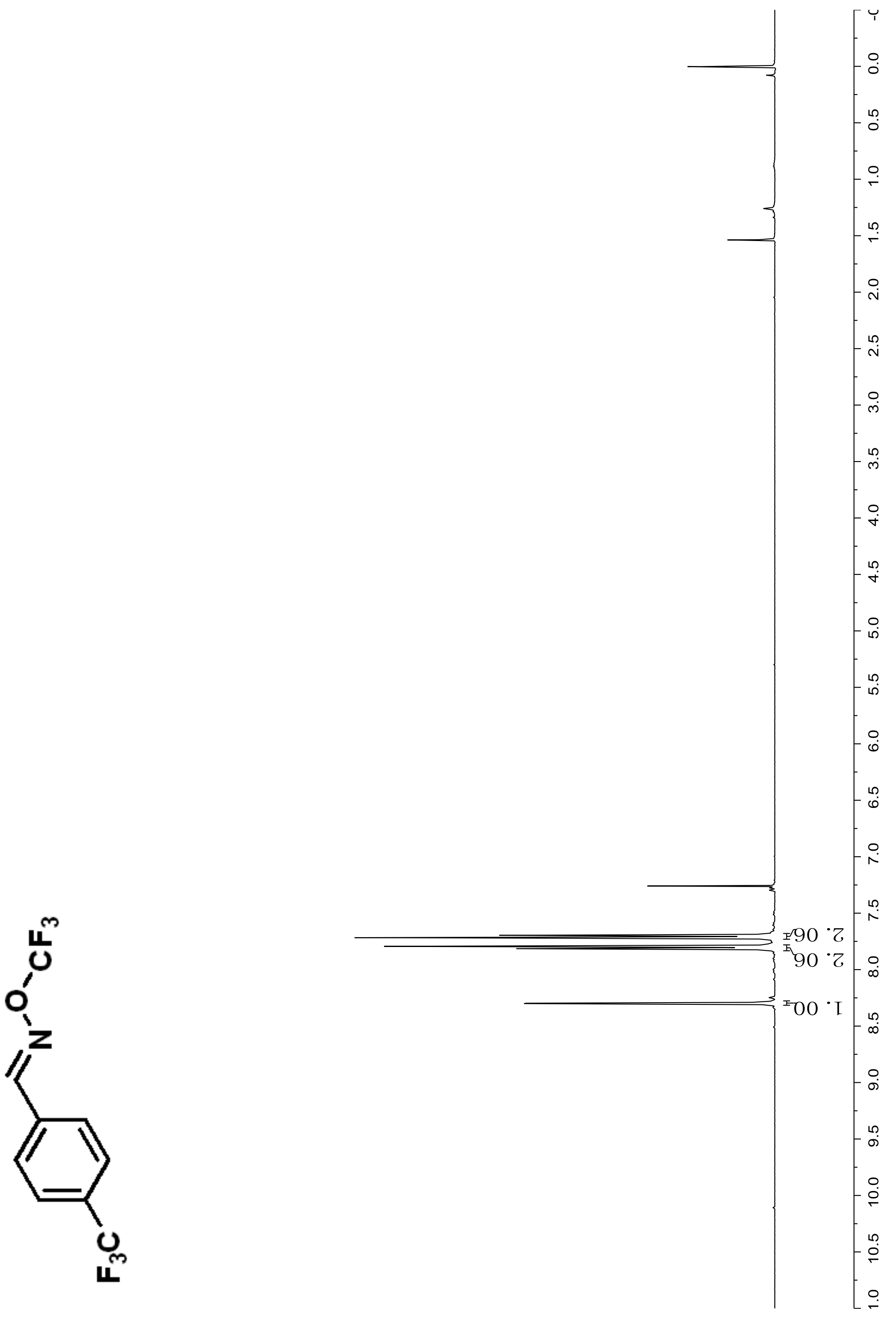

Supplementary Figure 26: ^{1}H NMR spectrum (400 MHz, $CDCl_3$, 23 °C) of **1h**

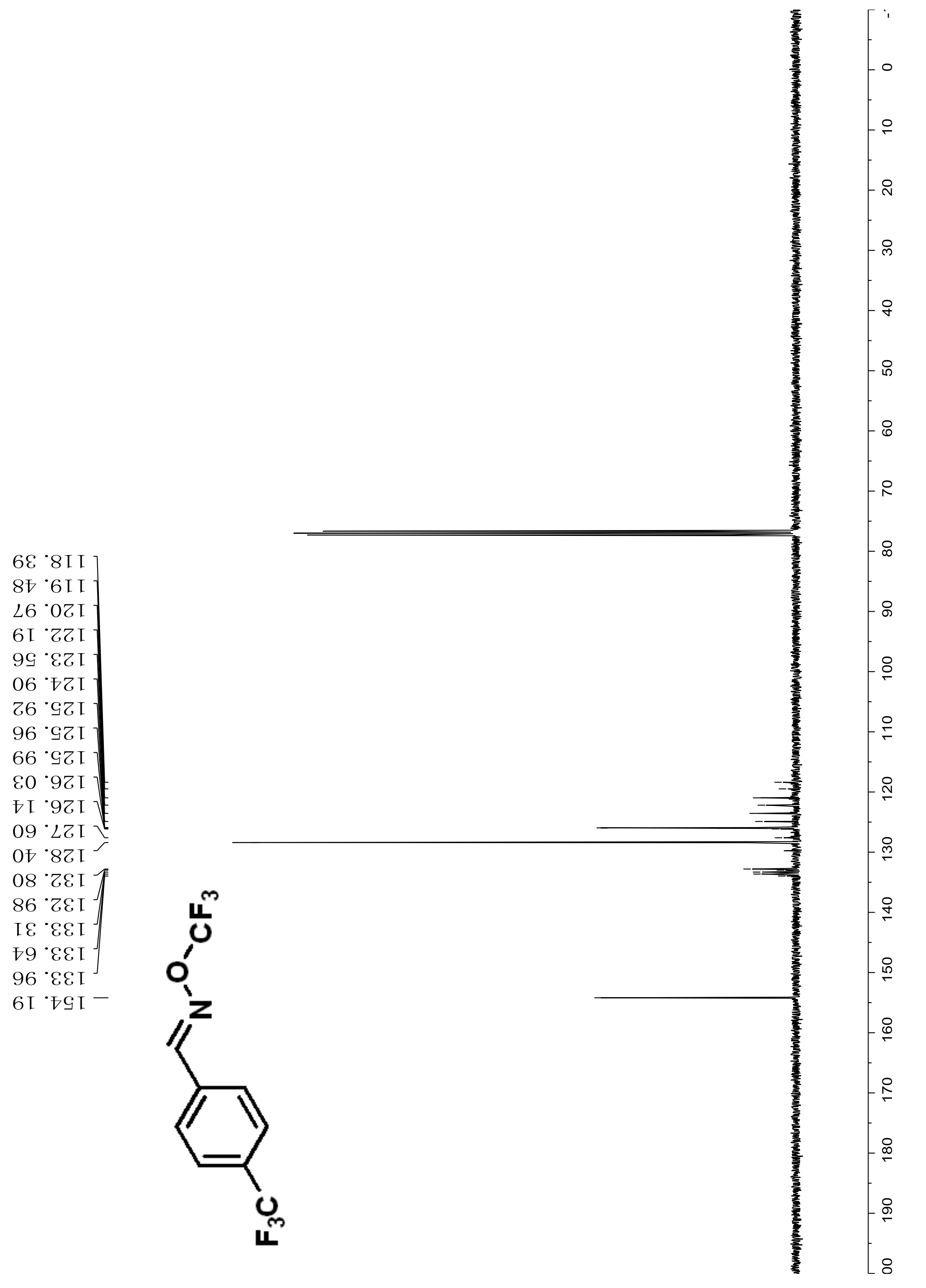

Supplementary Figure 27: ^{13}C NMR spectrum (101 MHz, $CDCl_3$, 23 °C) of **1h**

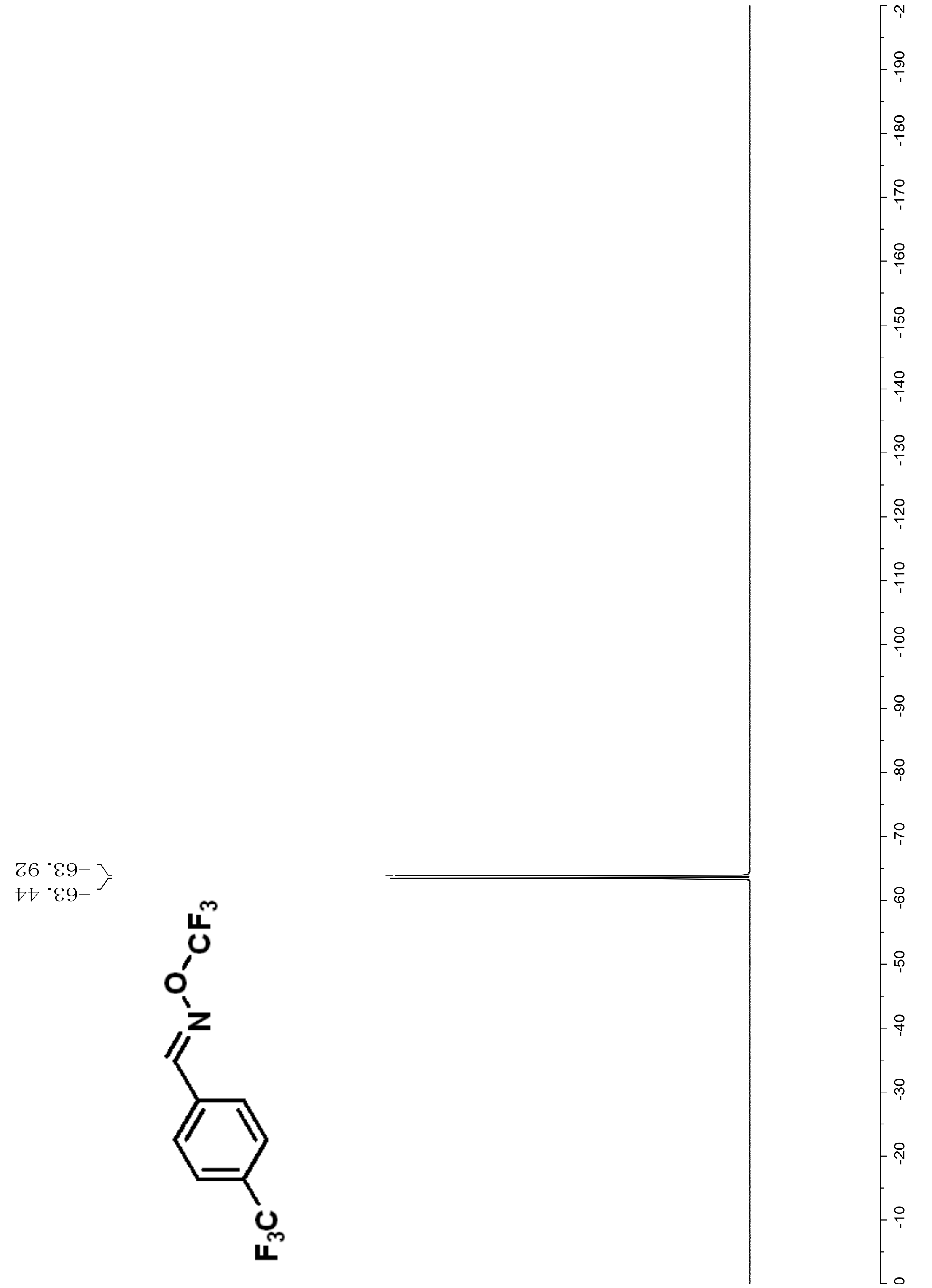

Supplementary Figure 28: ^{19}F NMR spectrum (376 MHz, $CDCl_3$, 23 °C) of **1h**

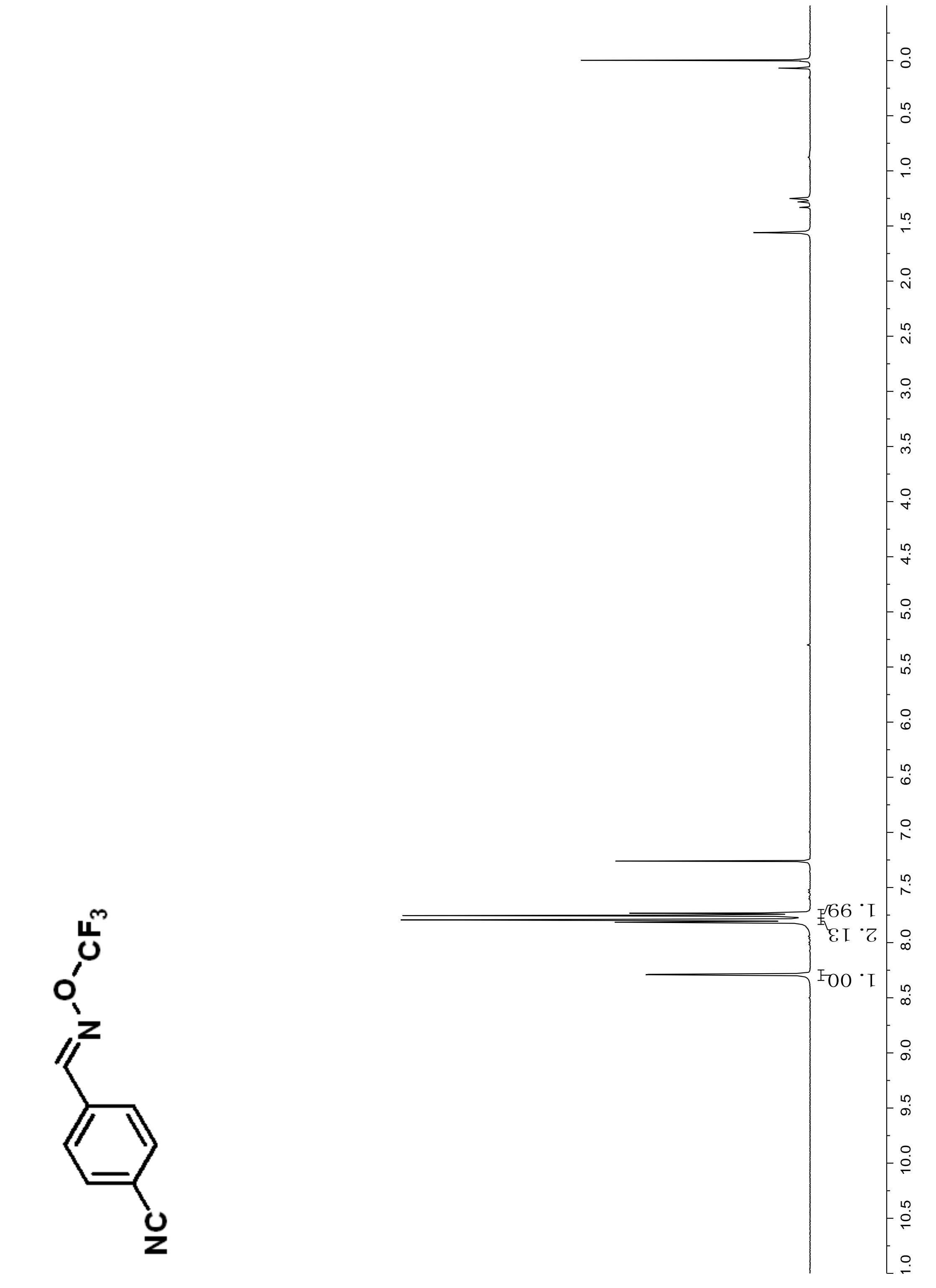

Supplementary Figure 29: ^{1}H NMR spectrum (400 MHz, $CDCl_3$, 23 °C) of **1i**

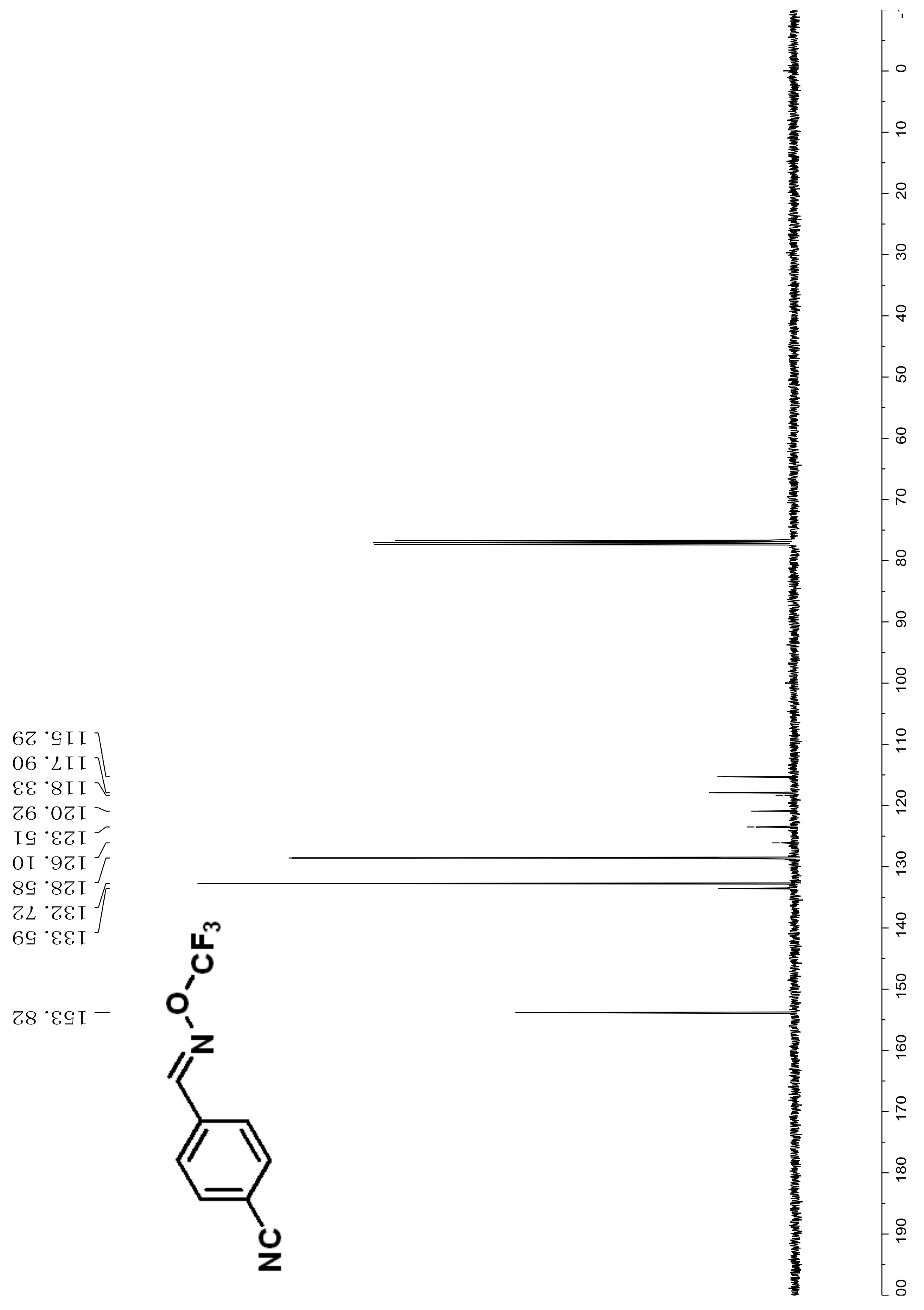

Supplementary Figure 30: ^{13}C NMR spectrum (101 MHz, $CDCl_3$, 23 °C) of **1i**

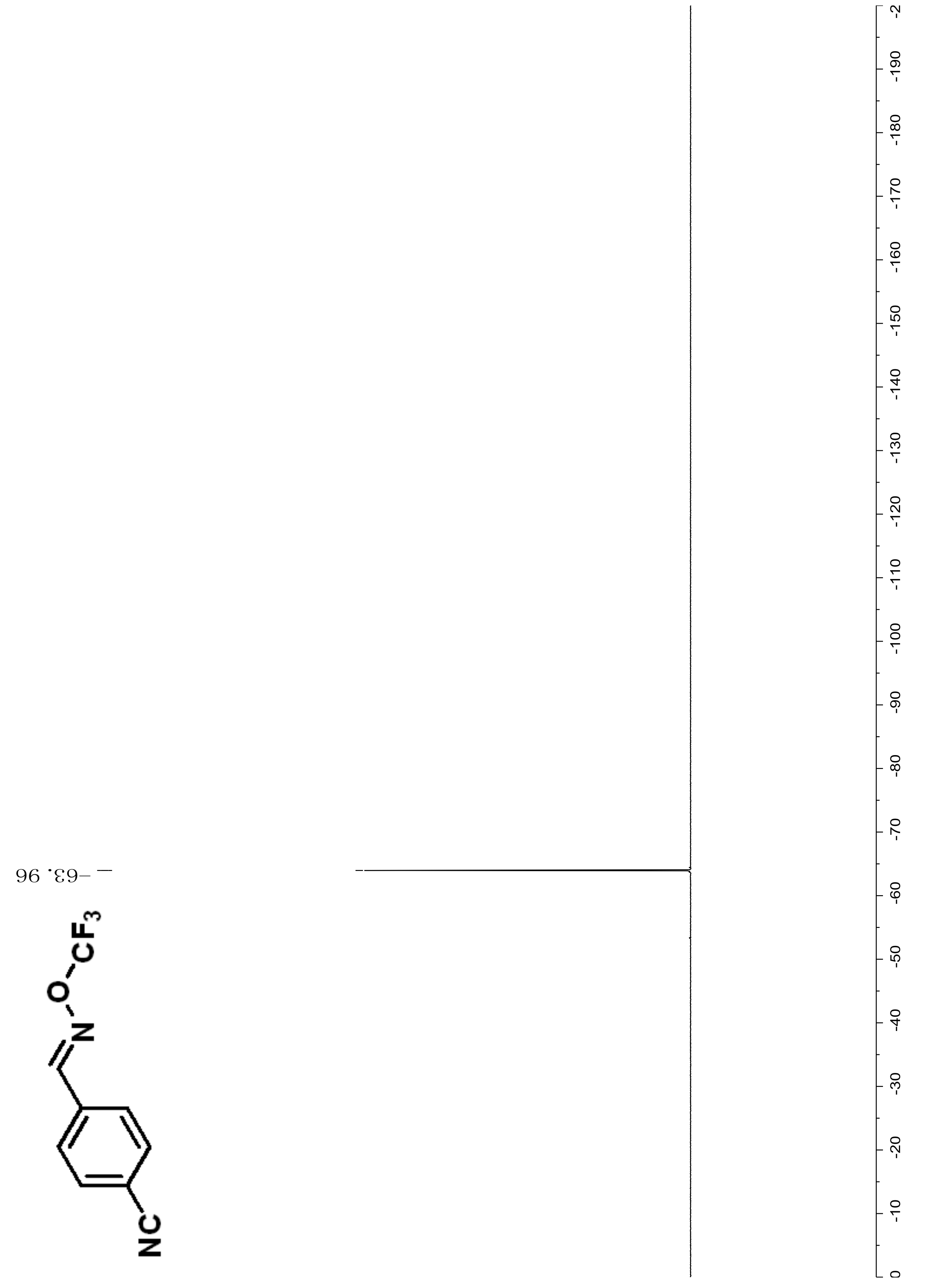

Supplementary Figure 31: ^{19}F NMR spectrum (376 MHz, $CDCl_3$, 23 °C) of **1i**

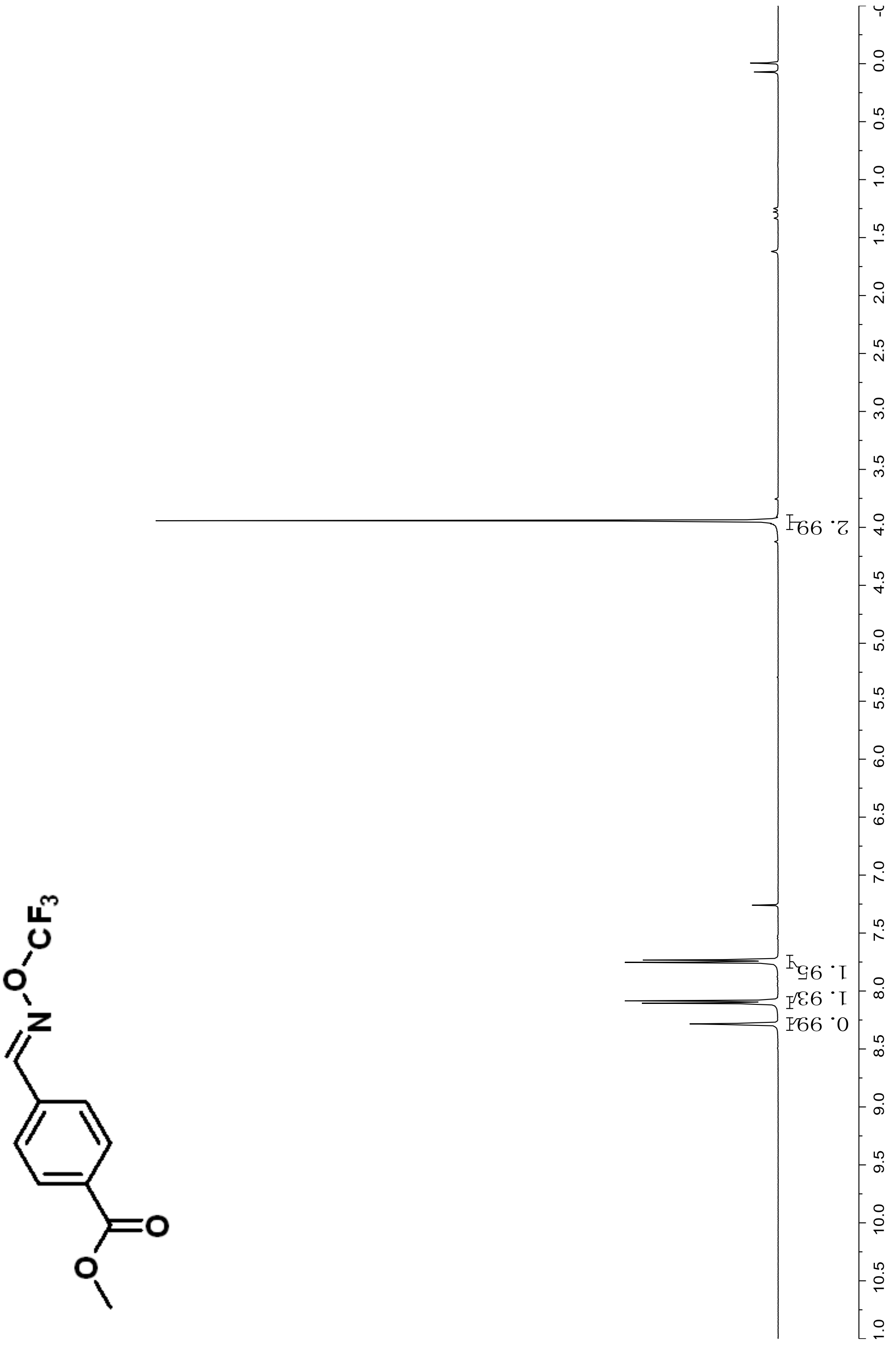

Supplementary Figure 31: ^{1}H NMR spectrum (400 MHz, $CDCl_3$, 23 °C) of **1j**

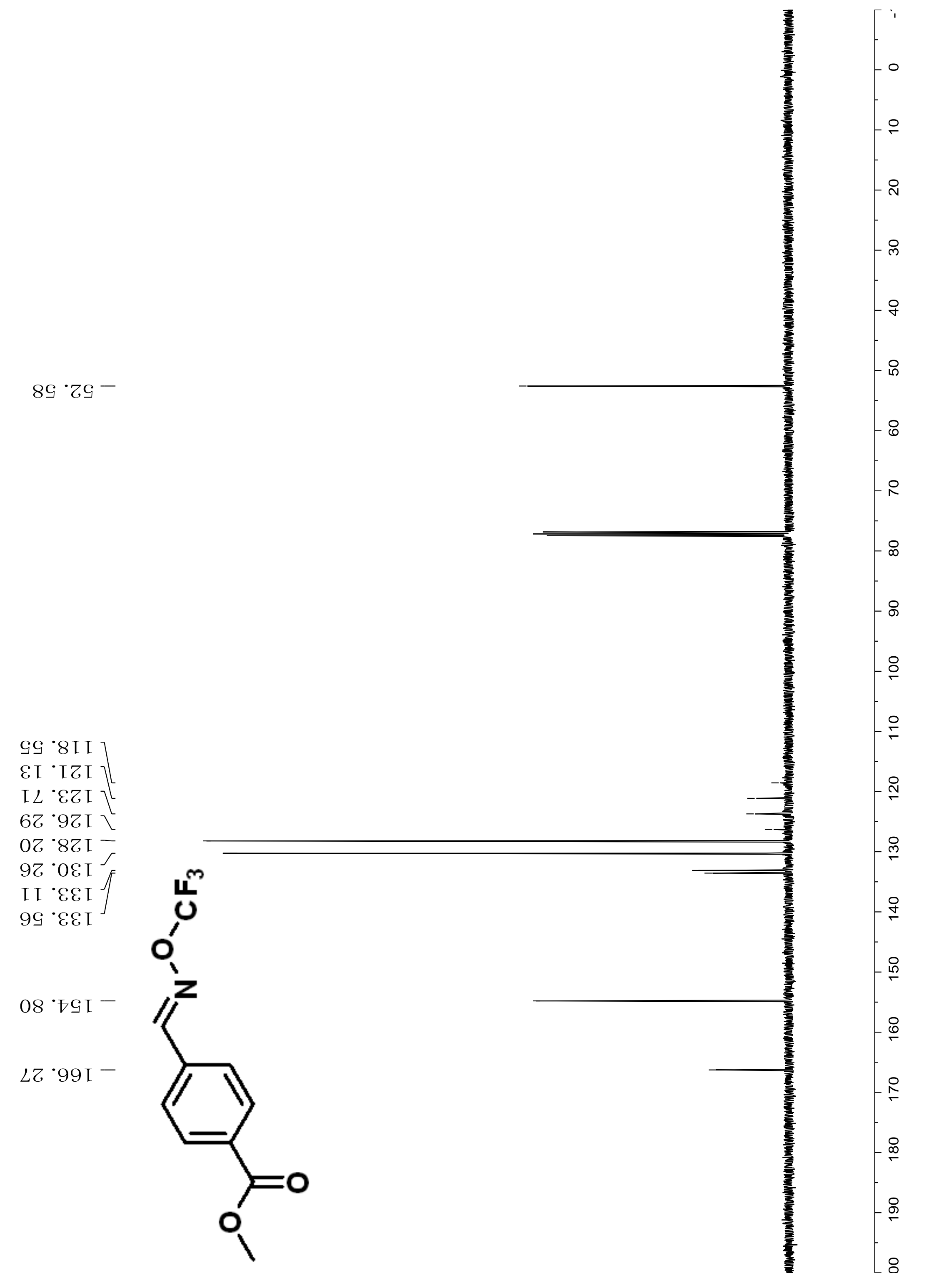

Supplementary Figure 33: ^{13}C NMR spectrum (101 MHz, $CDCl_3$, 23 °C) of **1j**

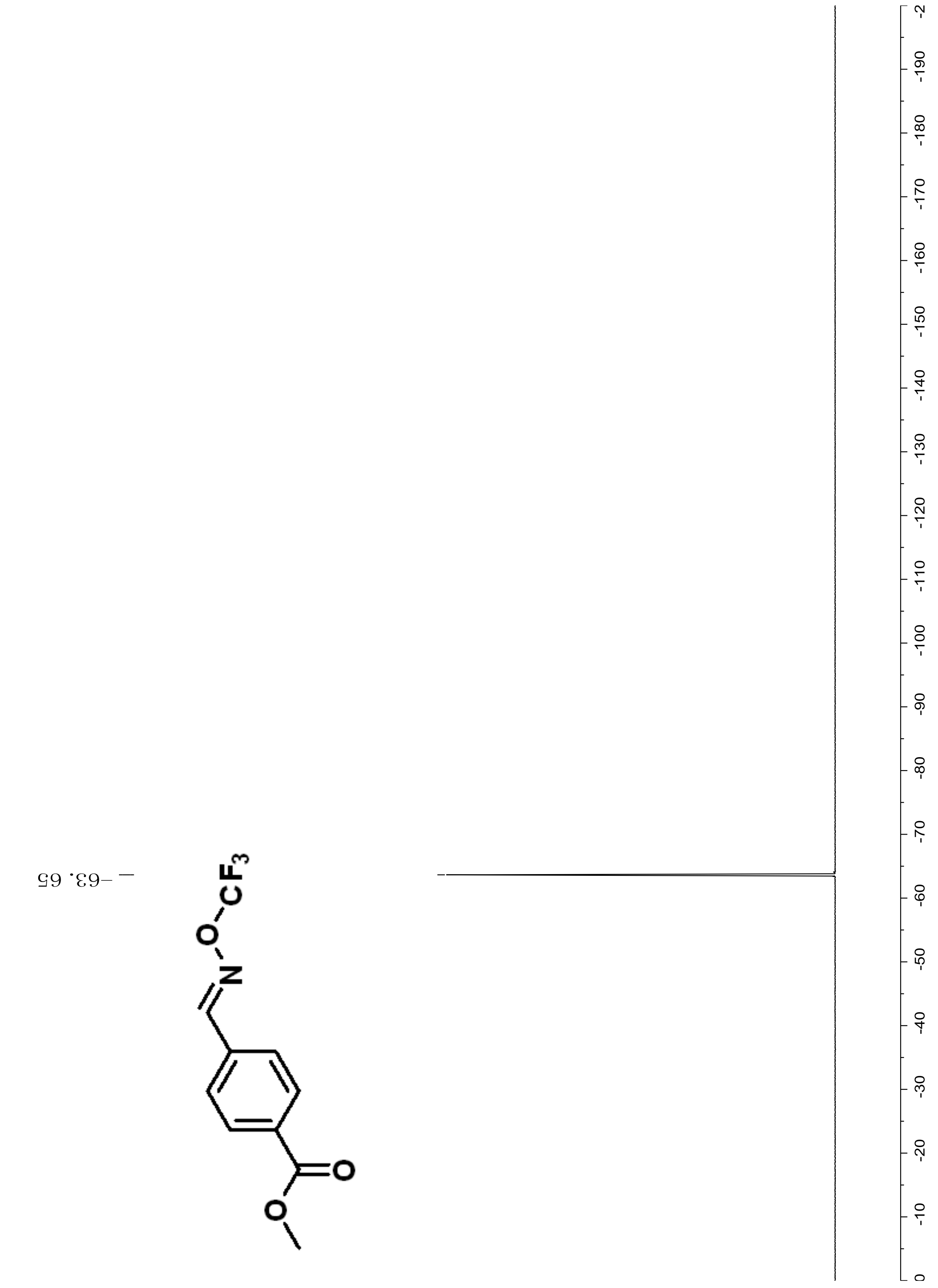

Supplementary Figure 34: ^{19}F NMR spectrum (376 MHz, DMSO, 23 °C) of **1j**

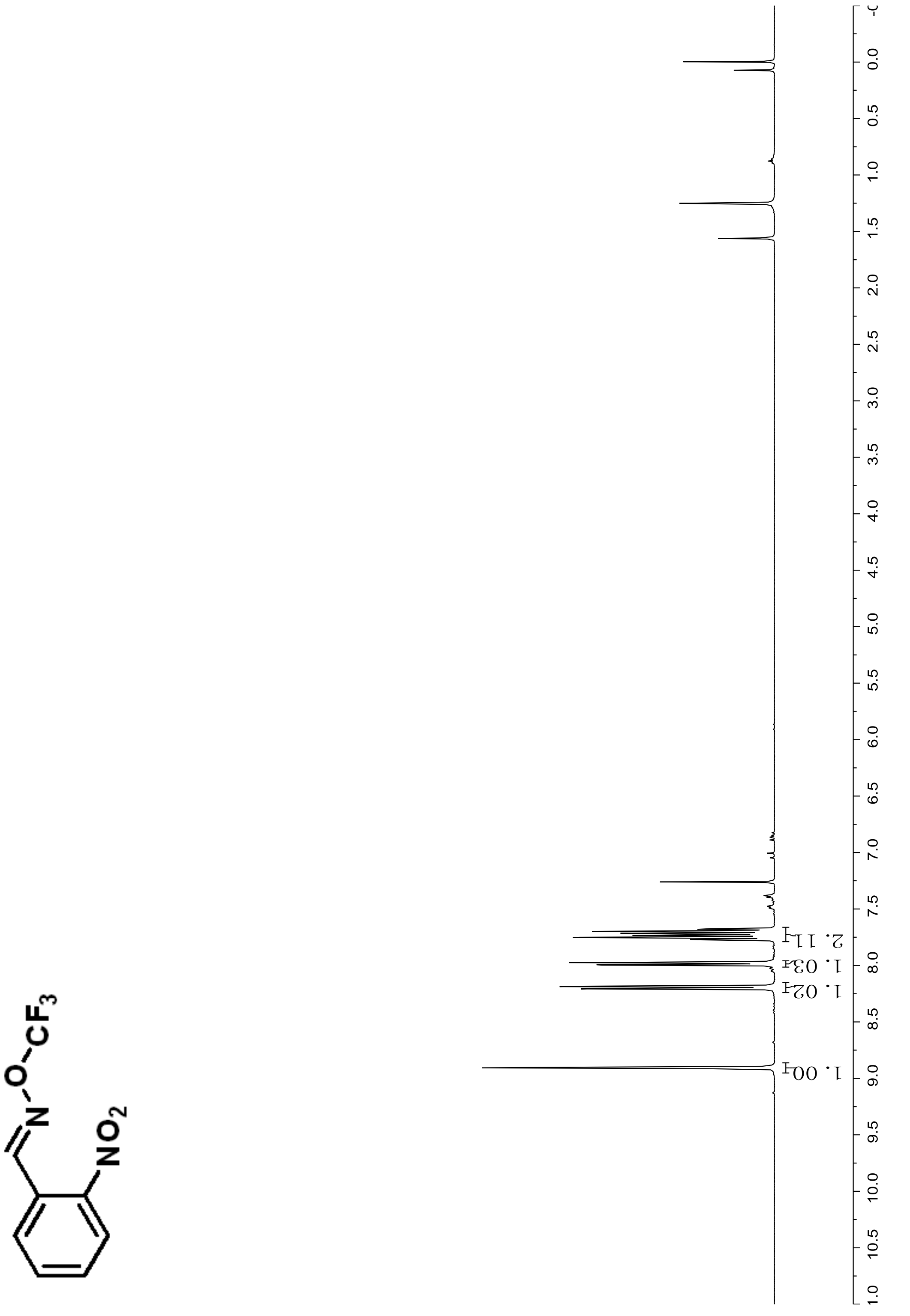

Supplementary Figure 35: ^{1}H NMR spectrum (400 MHz, $CDCl_3$, 23 °C) of **1k**

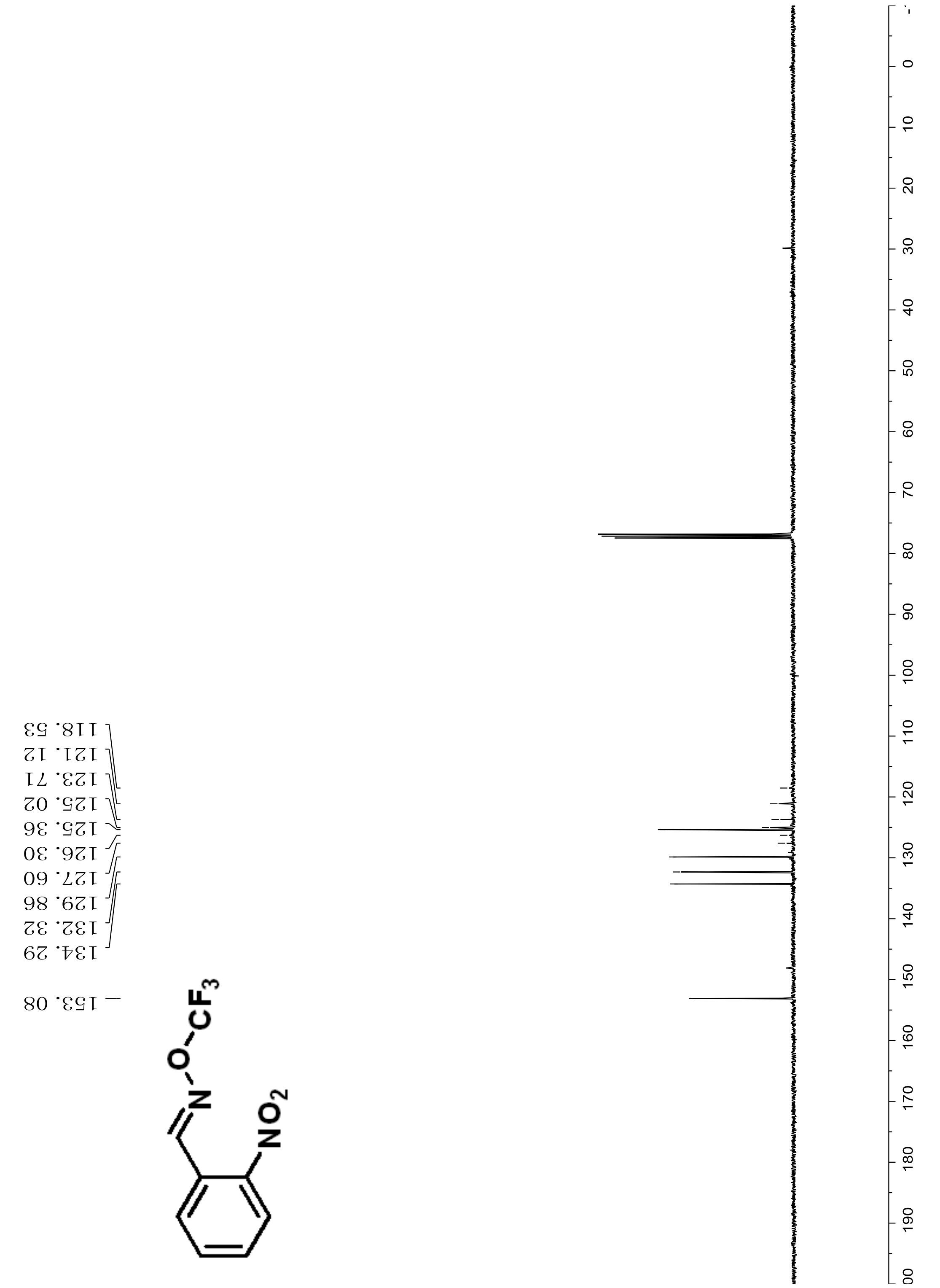

Supplementary Figure 36:^{13}C NMR spectrum (101 MHz, $CDCl_3$, 23 °C) of **1k**

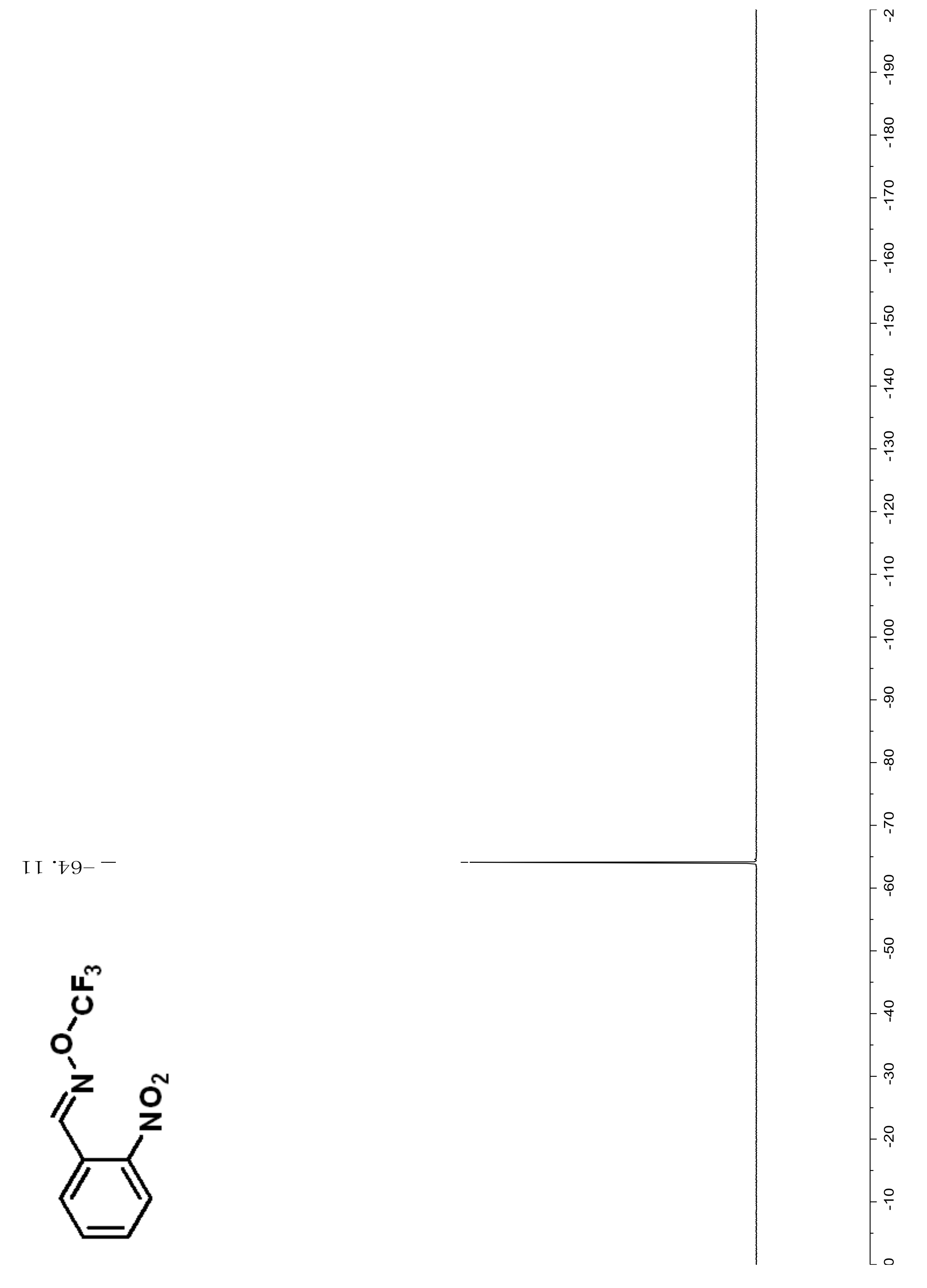

Supplementary Figure 37: ^{19}F NMR spectrum (376 MHz, DMSO, 23 °C) of **1k**

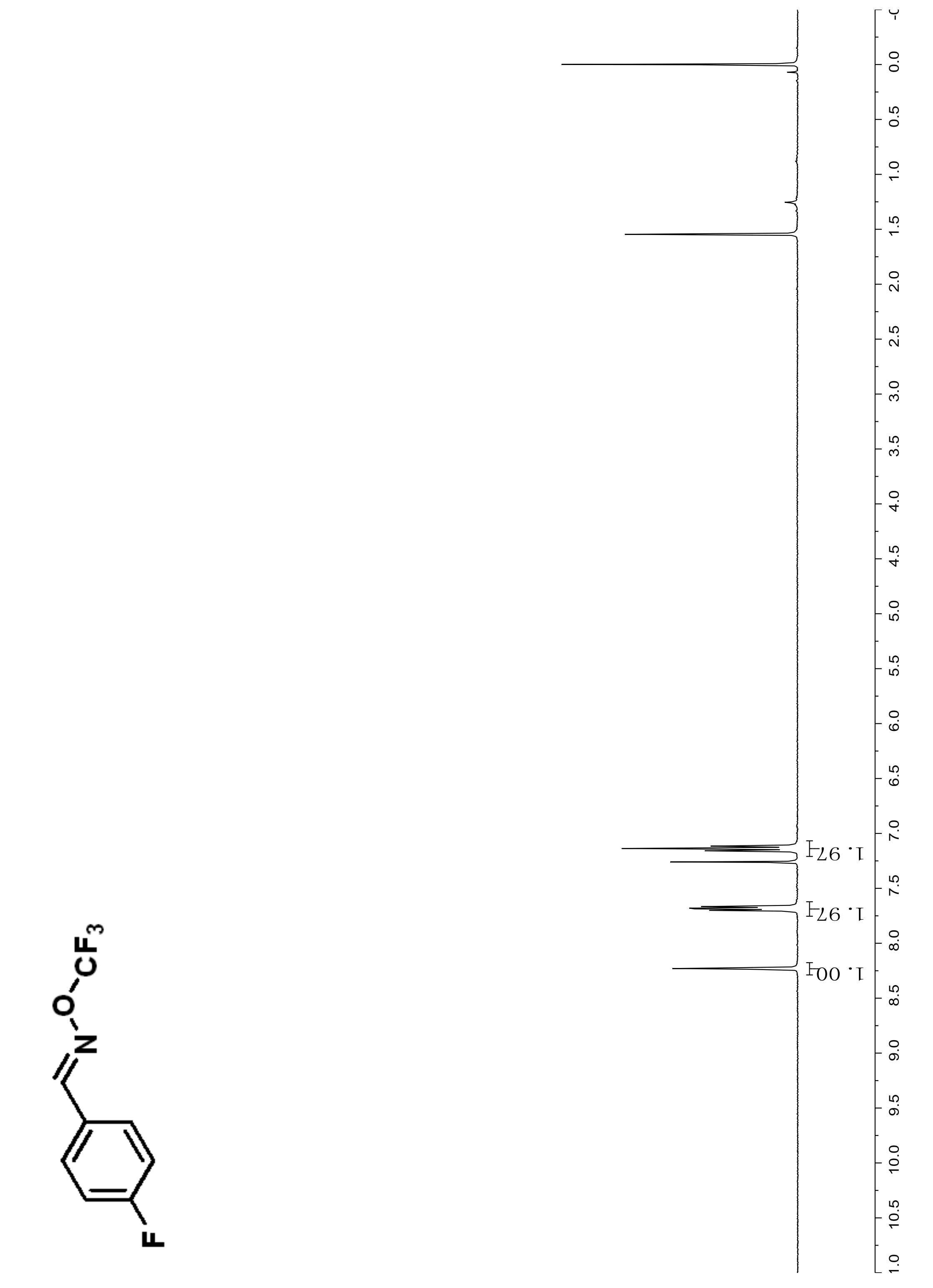

Supplementary Figure 38: ^{1}H NMR spectrum (400 MHz, $CDCl_3$, 23 °C) of **1l**

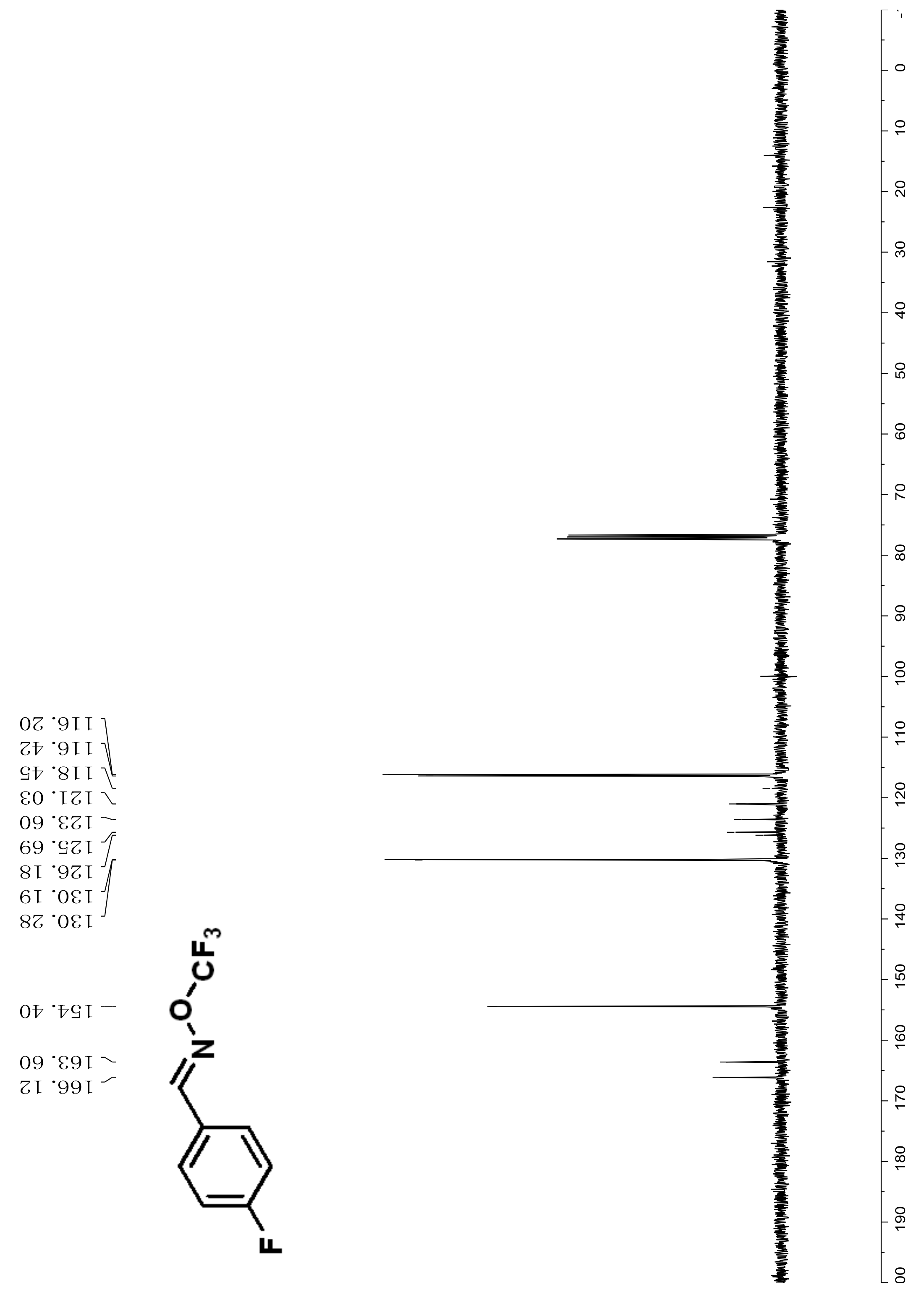

Supplementary Figure 39: ^{13}C NMR spectrum (101 MHz, $CDCl_3$, 23 °C) of **1l**

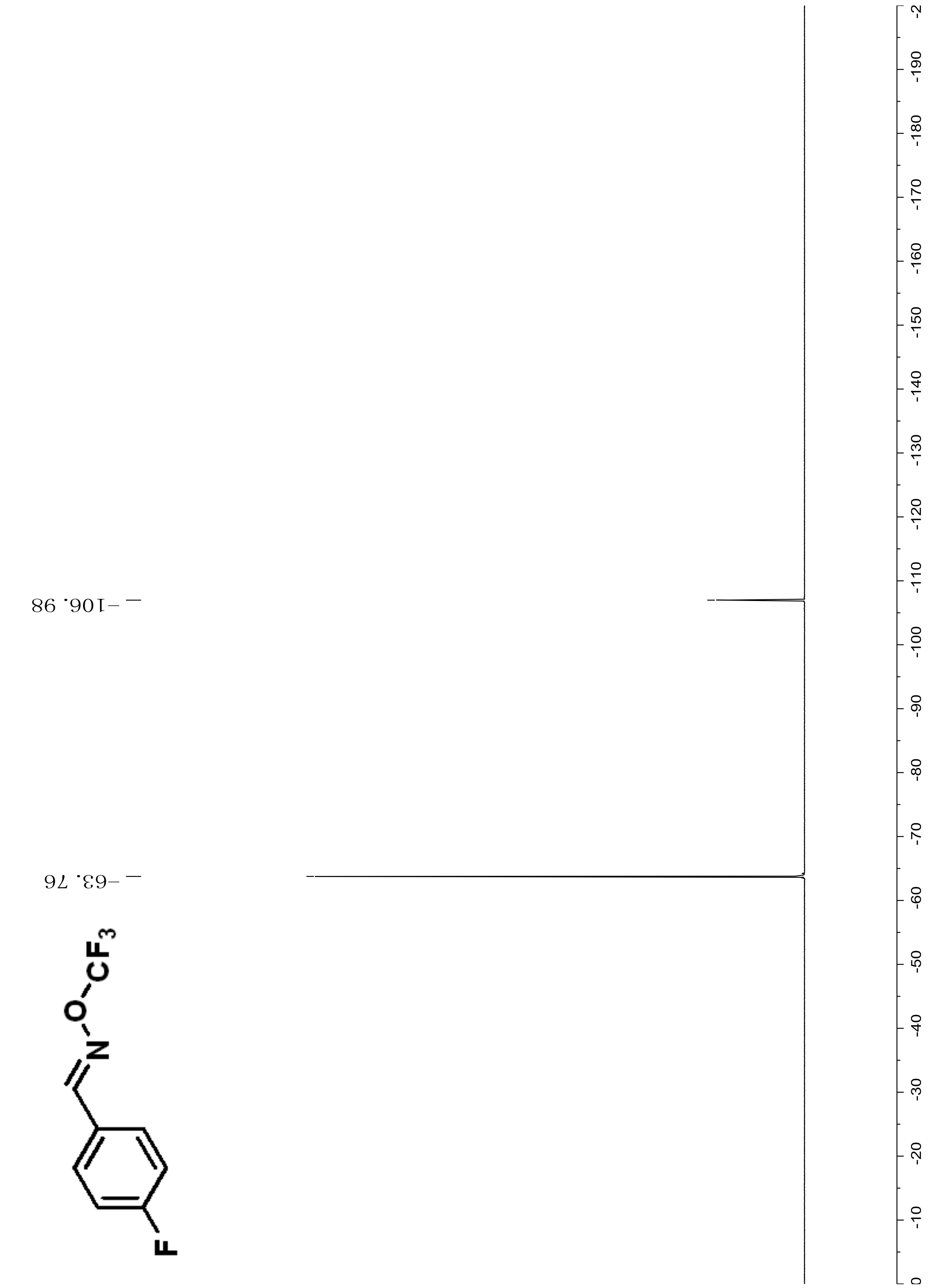

Supplementary Figure 40: ^{19}F NMR spectrum (376 MHz, $CDCl_3$, 23 °C) of **1l**

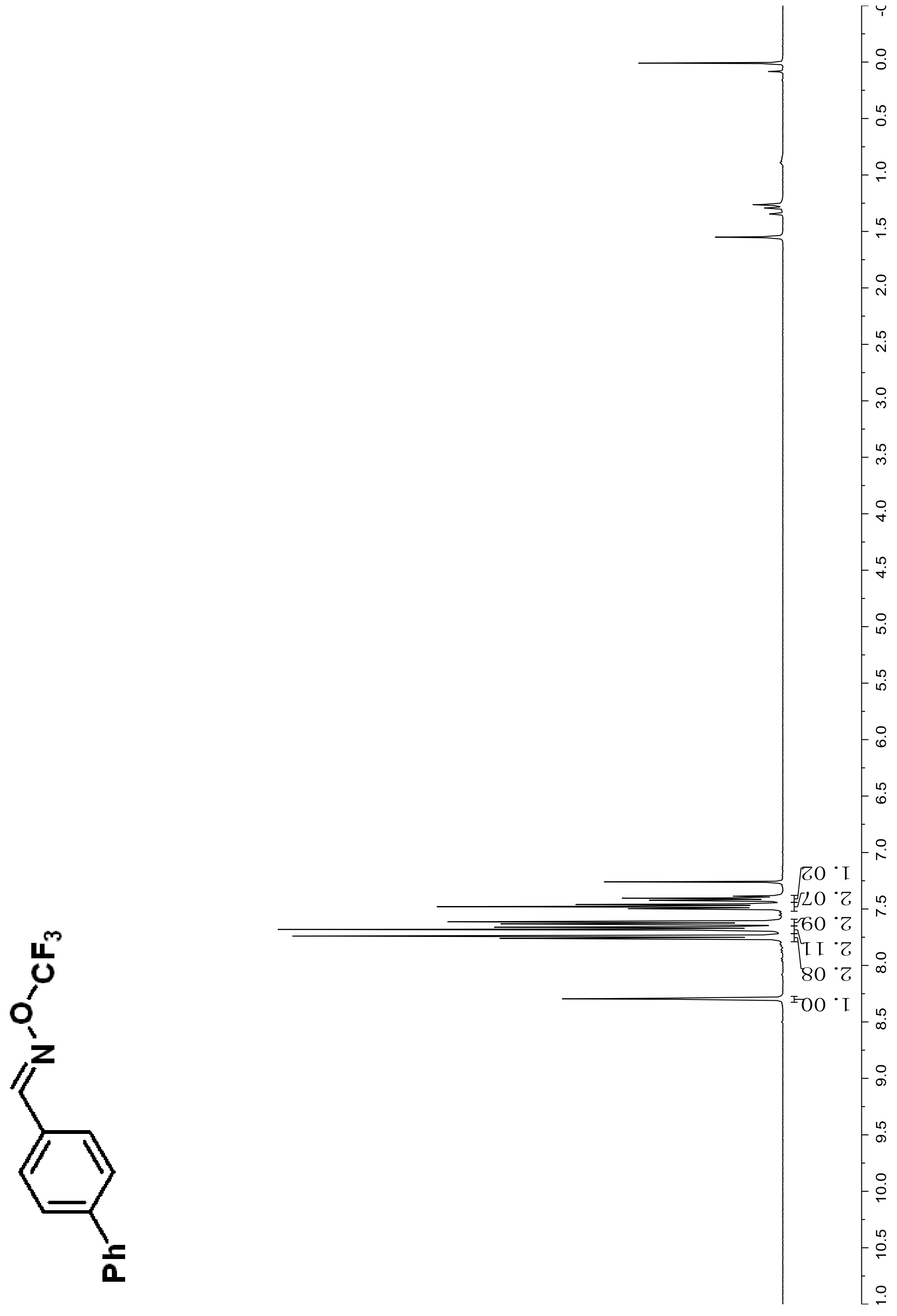

Supplementary Figure 41: ^{1}H NMR spectrum (400 MHz, $CDCl_3$, 23 °C) of **1m**

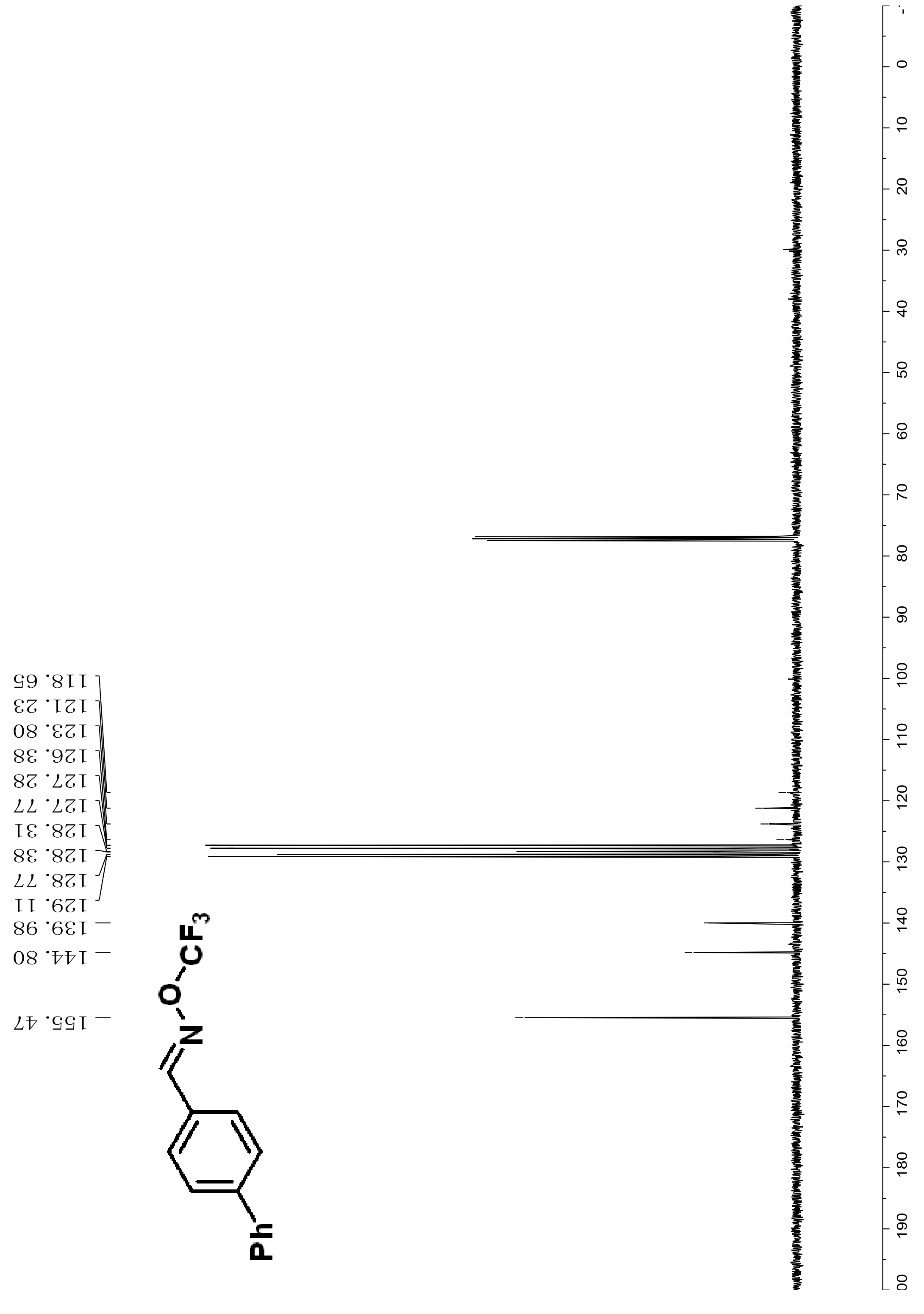

Supplementary Figure 42: ^{13}C NMR spectrum (101 MHz, $CDCl_3$, 23 °C) of **1m**

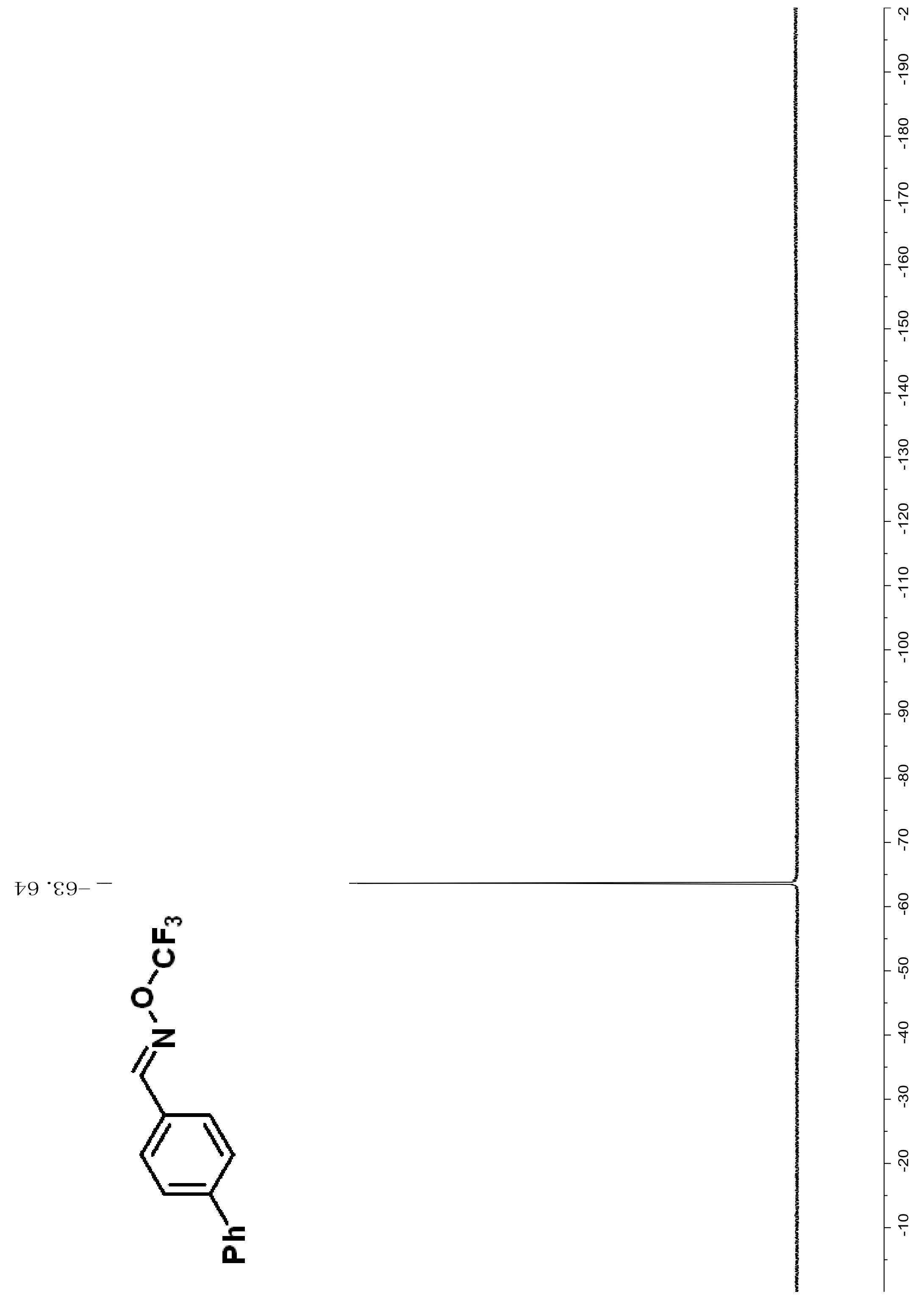

Supplementary Figure 43: ^{19}F NMR spectrum (376 MHz, $CDCl_3$, 23 °C) of **1m**

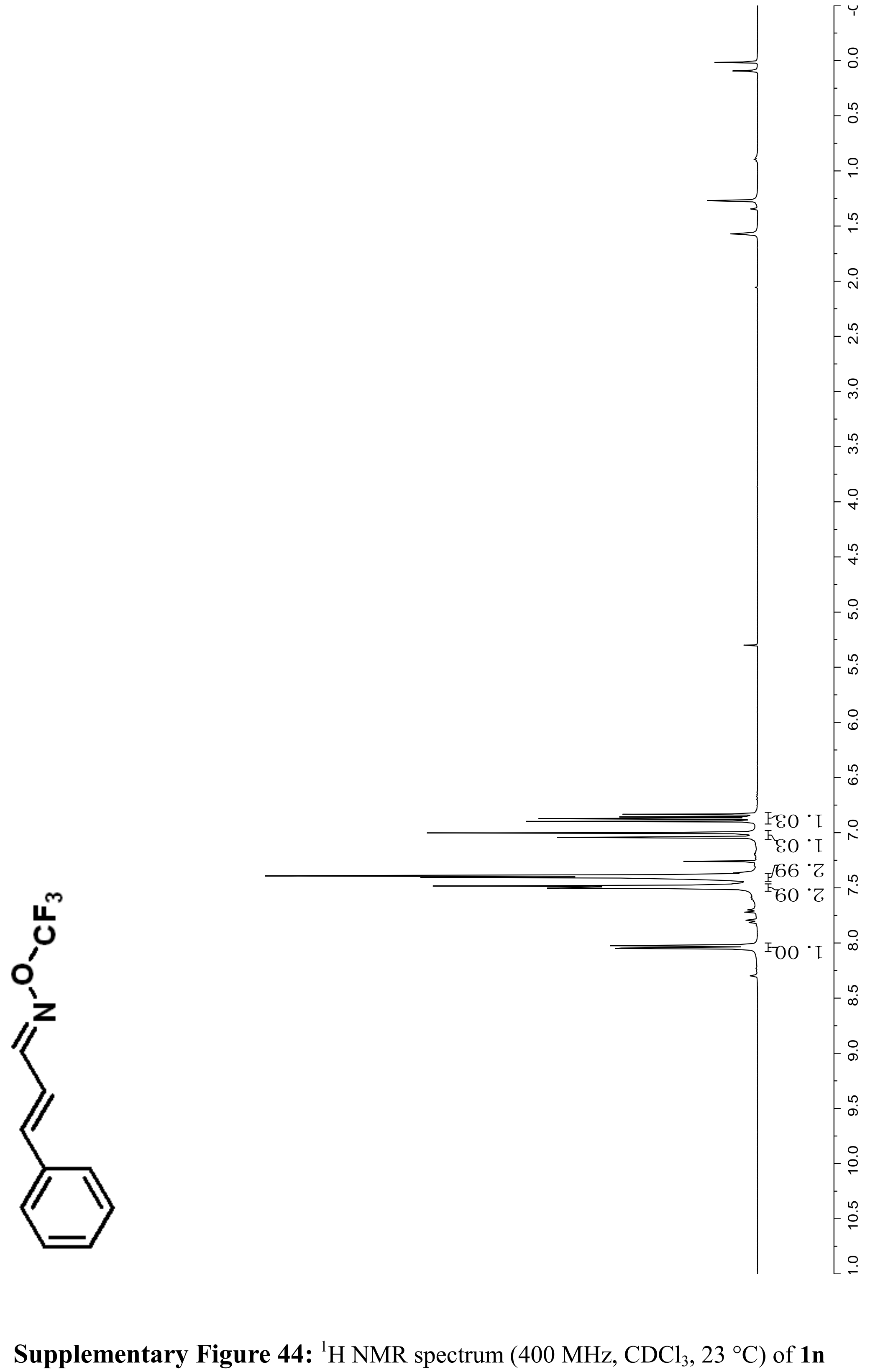

Supplementary Figure 44: ^{1}H NMR spectrum (400 MHz, $CDCl_3$, 23 °C) of **1n**

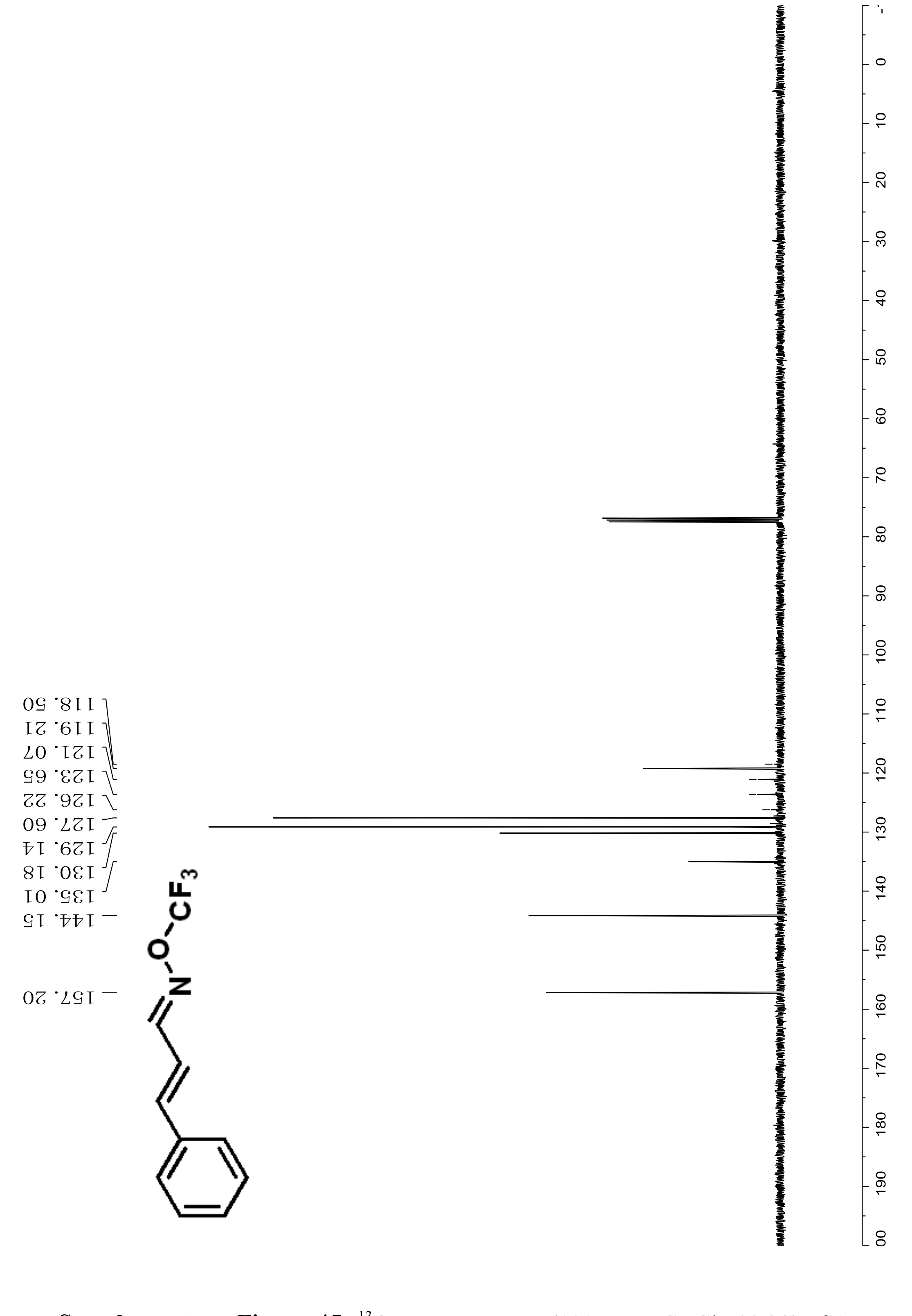

Supplementary Figure 45: ^{13}C NMR spectrum (101 MHz, $CDCl_3$, 23 °C) of **1n**

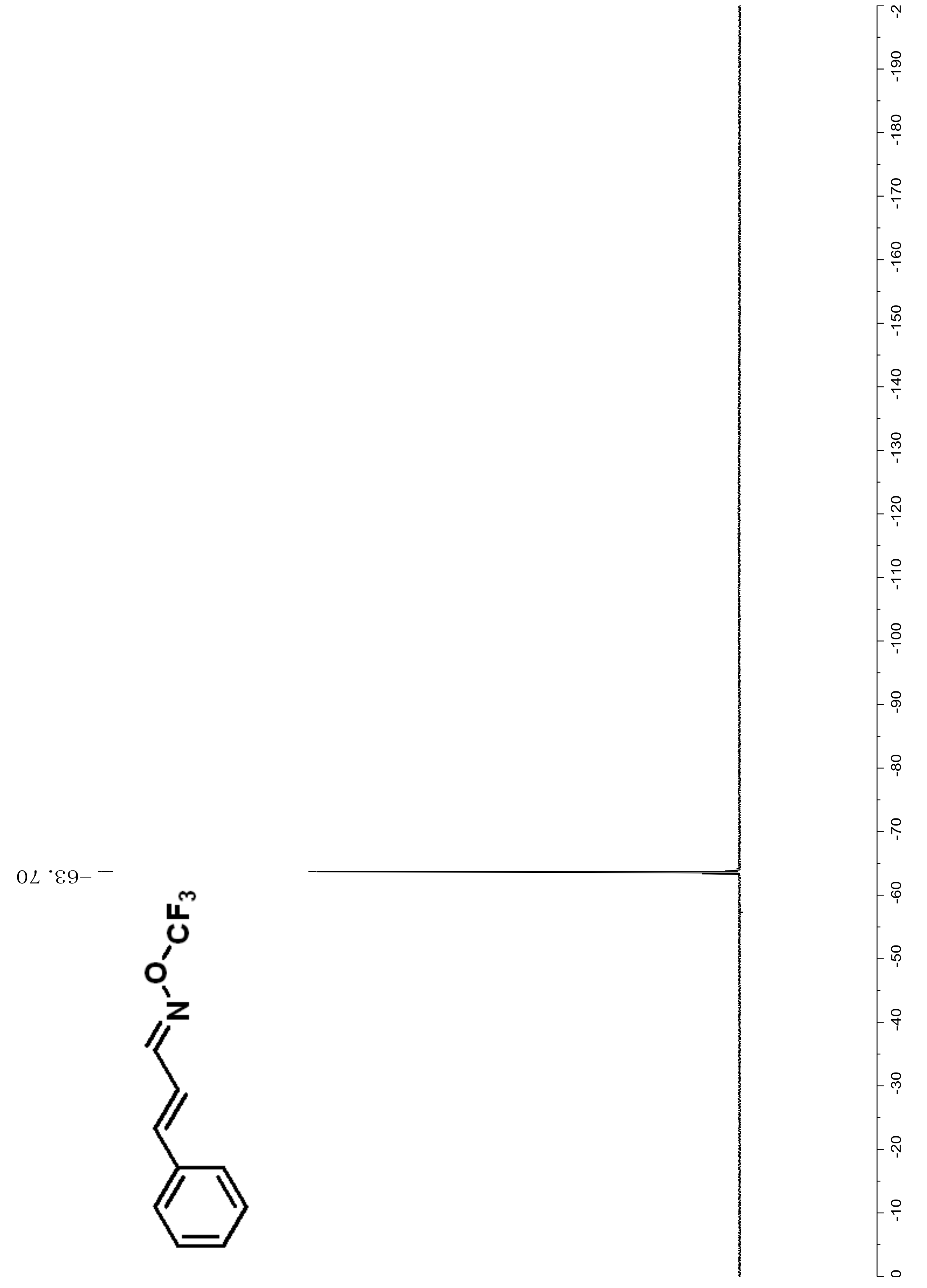

Supplementary Figure 46: ^{19}F NMR spectrum (376 MHz, $CDCl_3$, 23 °C) of **1n**

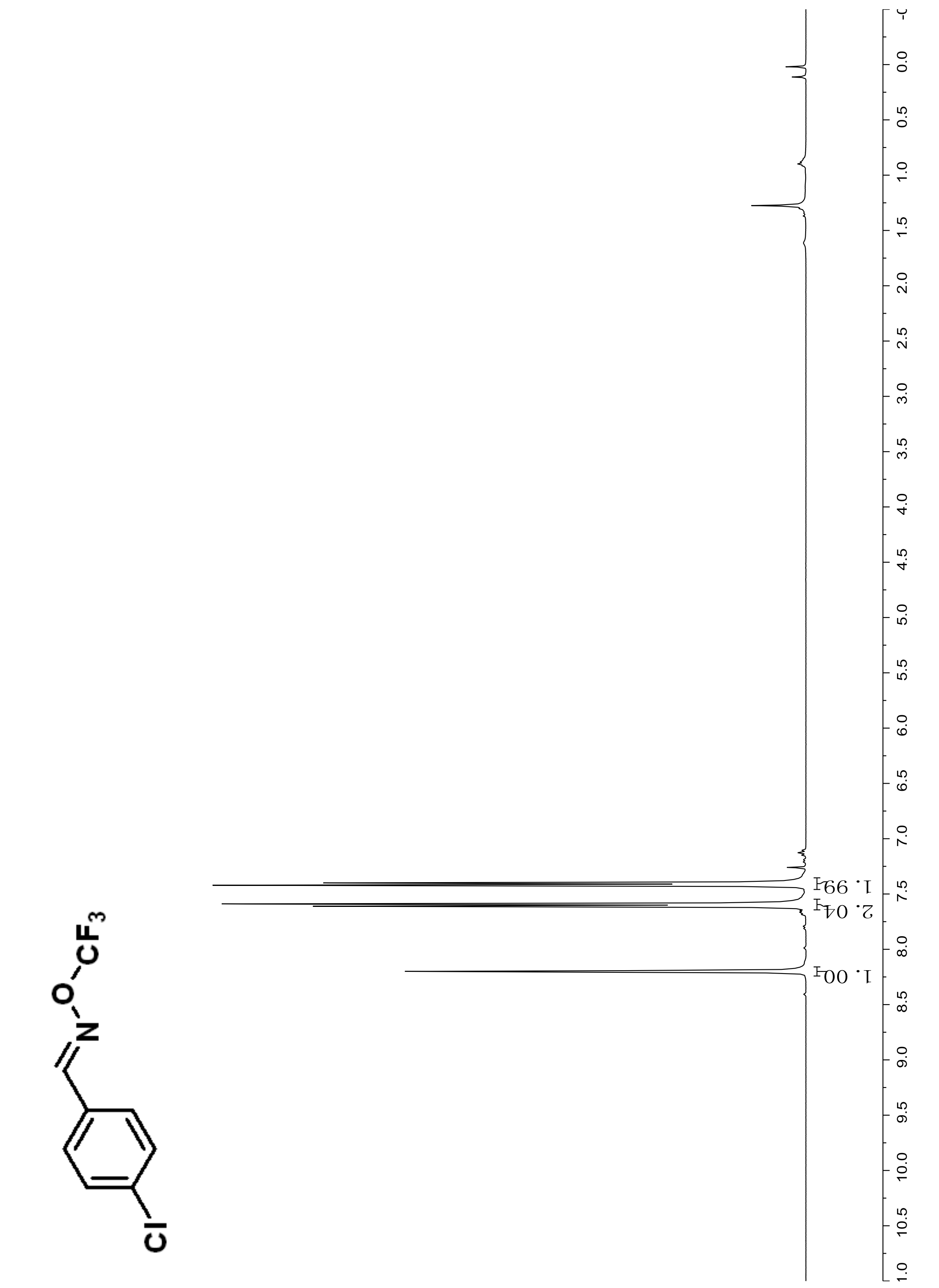

Supplementary Figure 47: ^{1}H NMR spectrum (400 MHz, $CDCl_3$, 23 °C) of **1o**

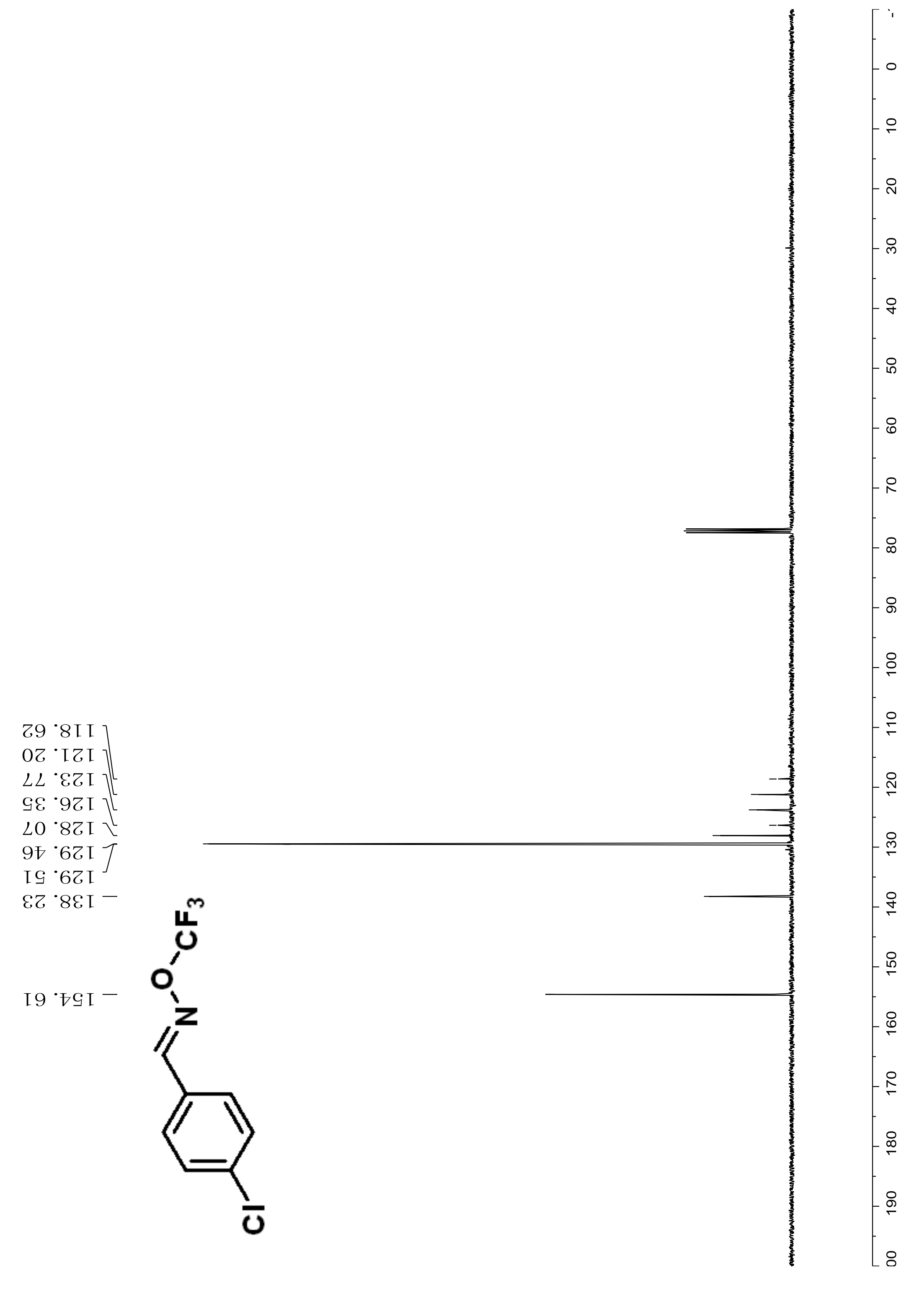

Supplementary Figure 48: ^{13}C NMR spectrum (101 MHz, $CDCl_3$, 23 °C) of **1o**

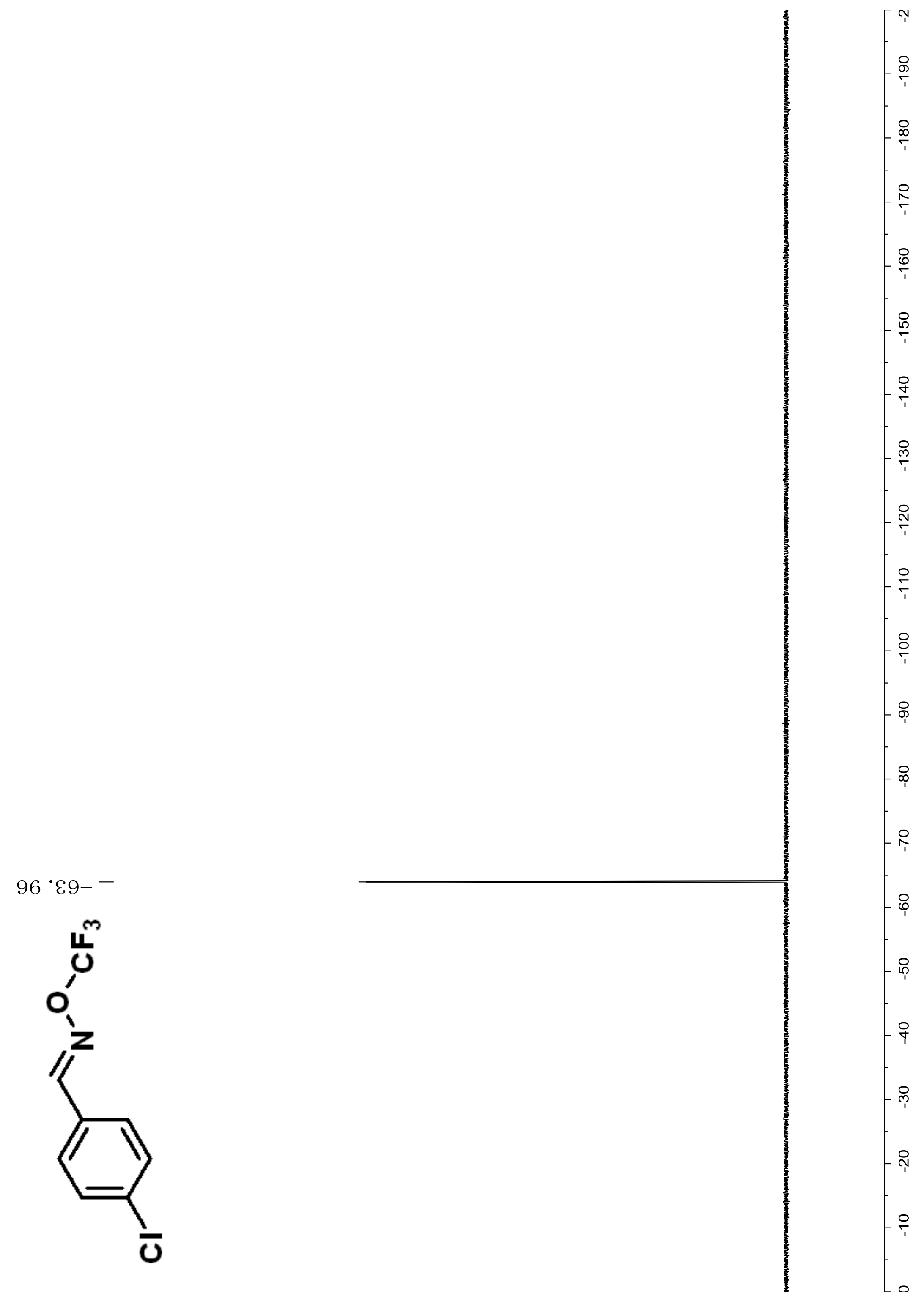

Supplementary Figure 49: ^{19}F NMR spectrum (376 MHz, DMSO, 23 °C) of **1o**

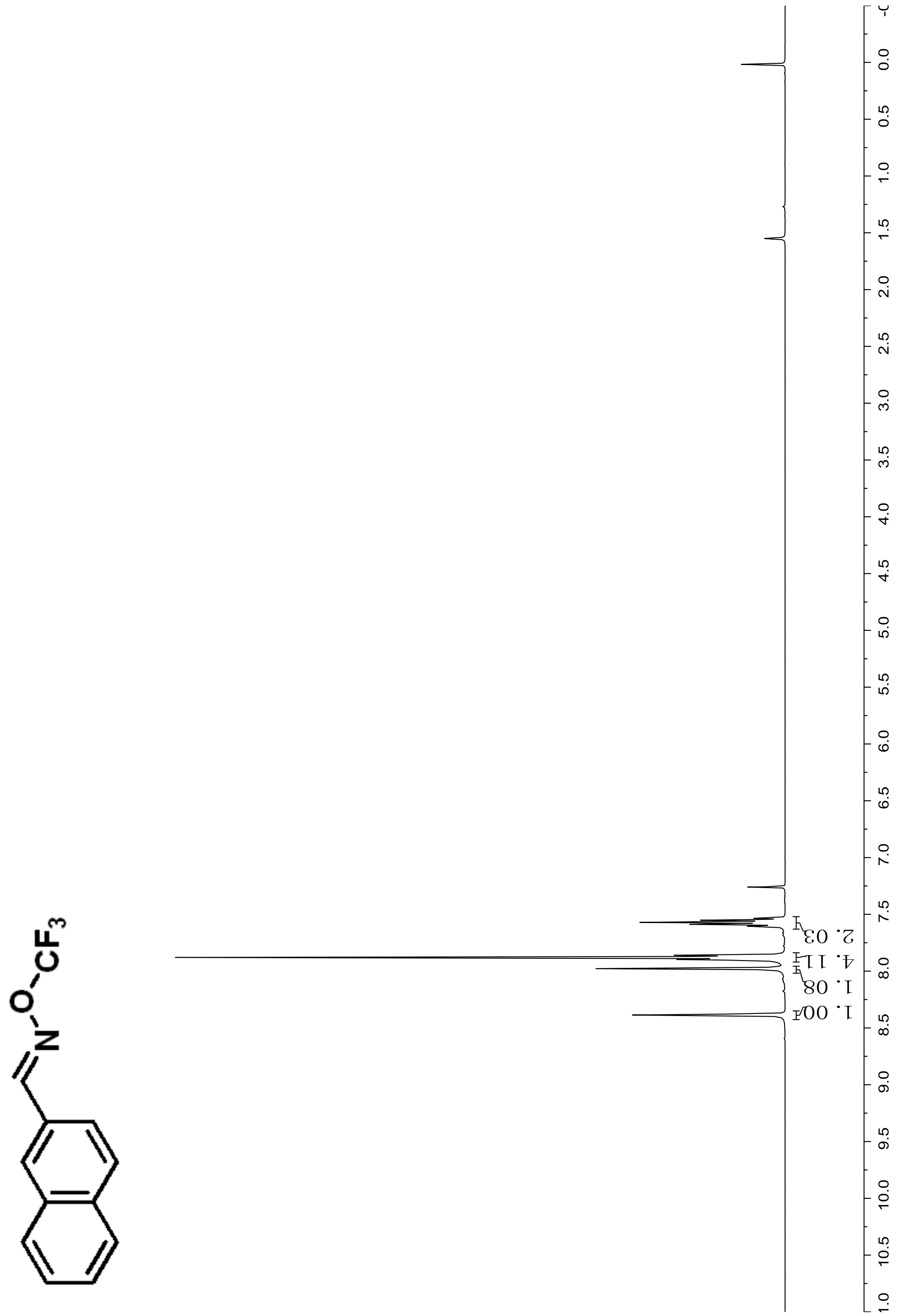

Supplementary Figure 50: ^{1}H NMR spectrum (400 MHz, $CDCl_3$, 23 °C) of **1p**

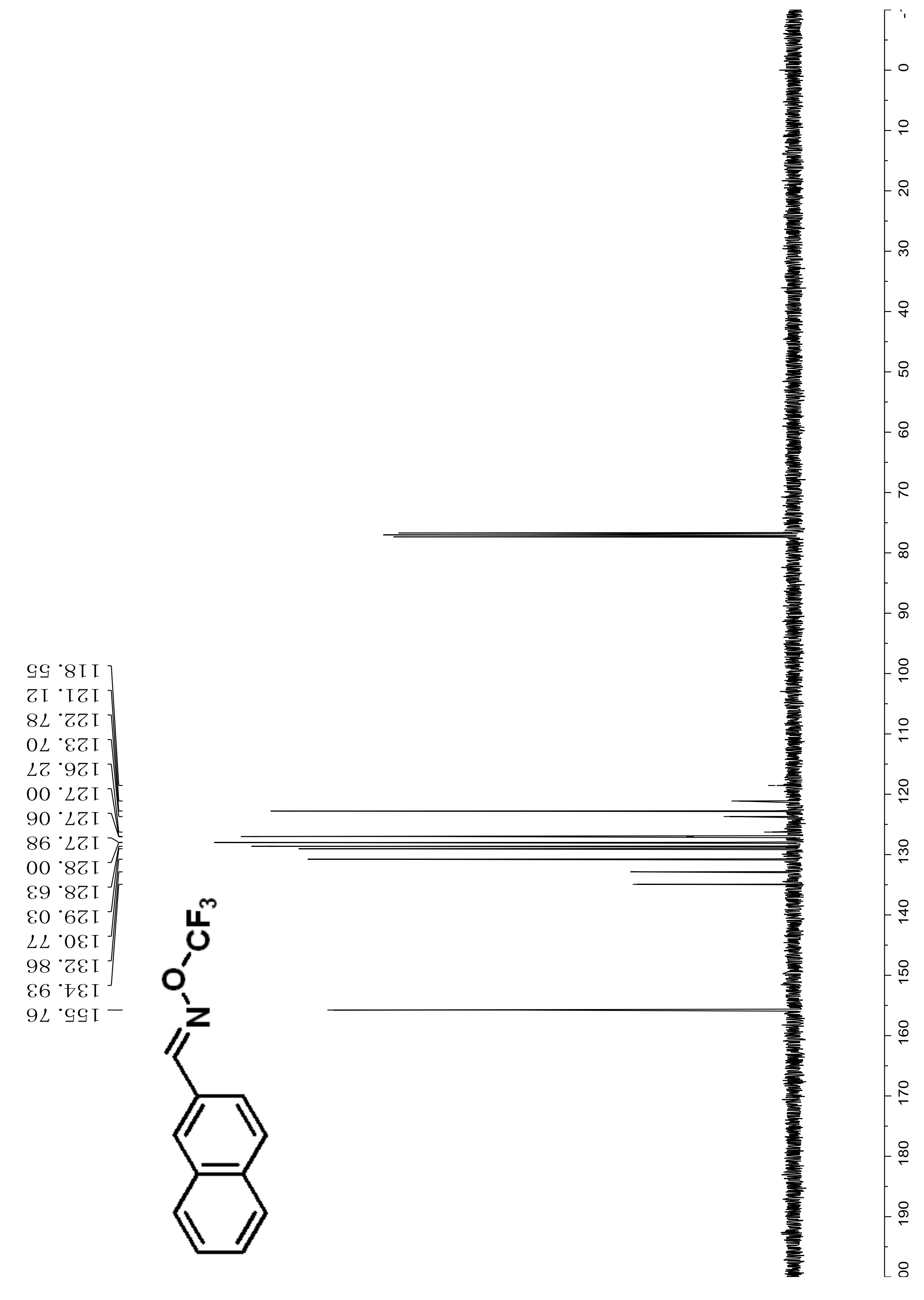

Supplementary Figure 51: ^{13}C NMR spectrum (101 MHz, $CDCl_3$, 23 °C) of **1p**

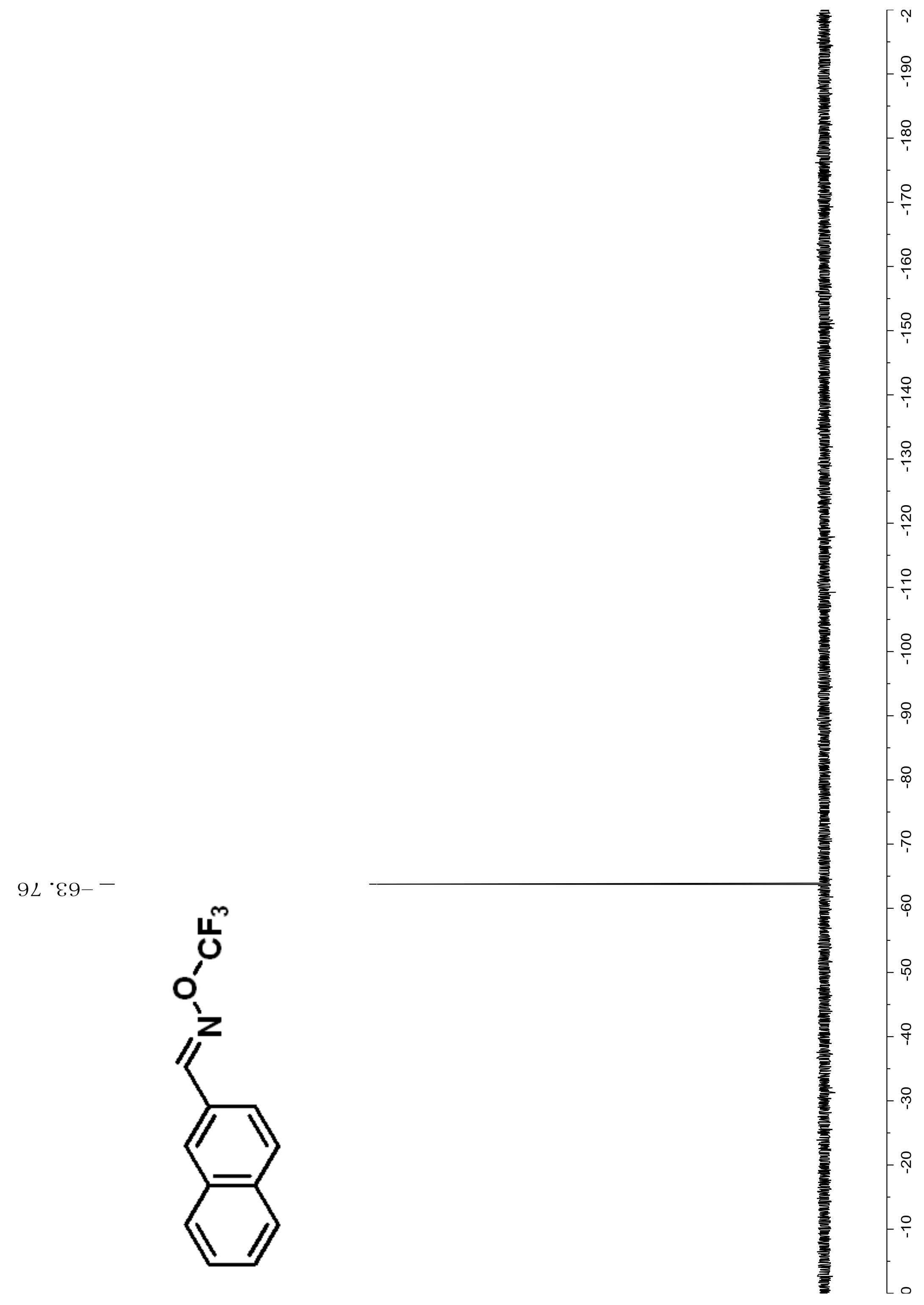

Supplementary Figure 52: ^{19}F NMR spectrum (376 MHz, DMSO, 23 °C) of **1p**

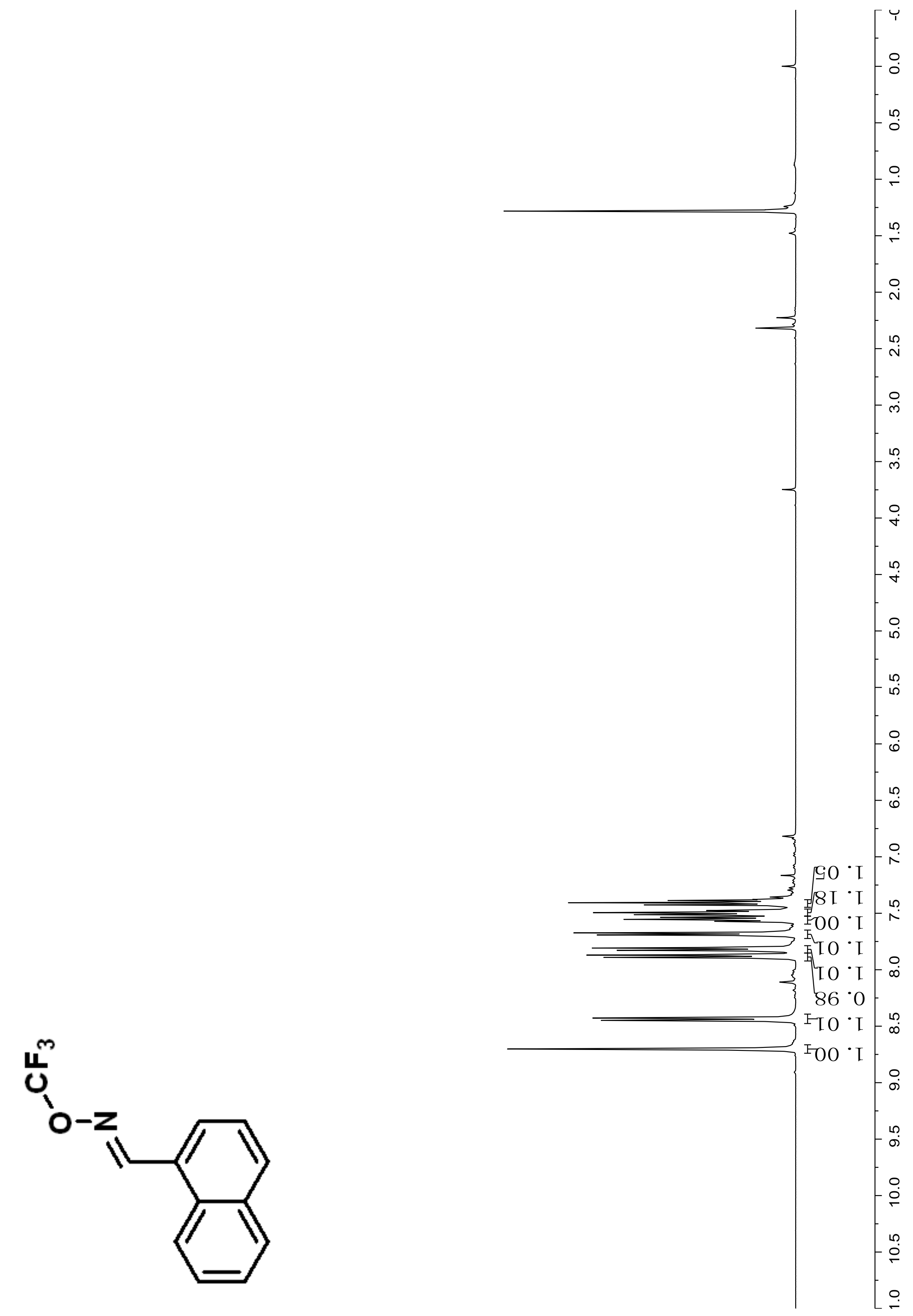

Supplementary Figure 53: ^{1}H NMR spectrum (400 MHz, $CDCl_3$, 23 °C) of **1q**

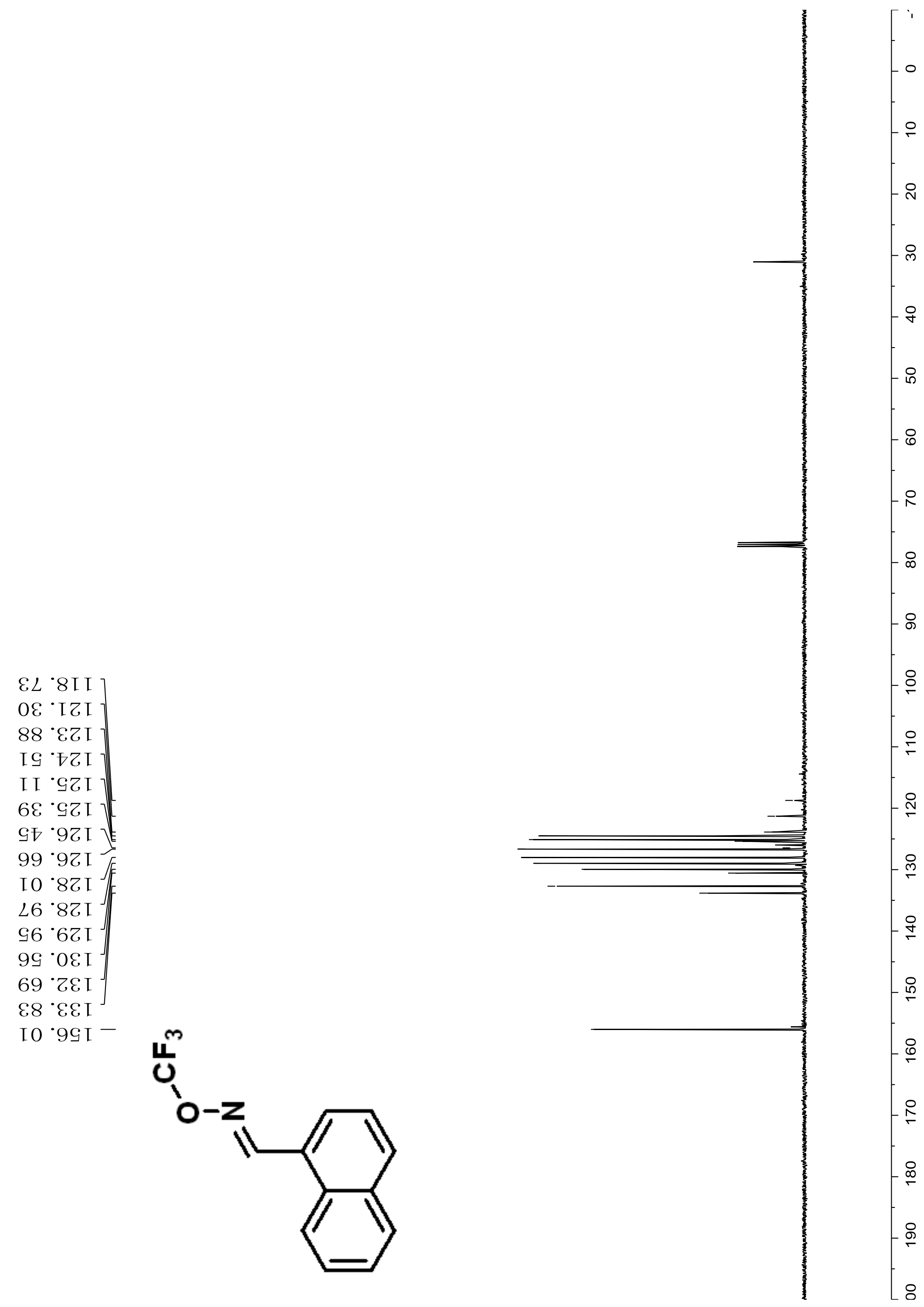

Supplementary Figure 54: ^{13}C NMR spectrum (101 MHz, $CDCl_3$, 23 °C) of **1q**

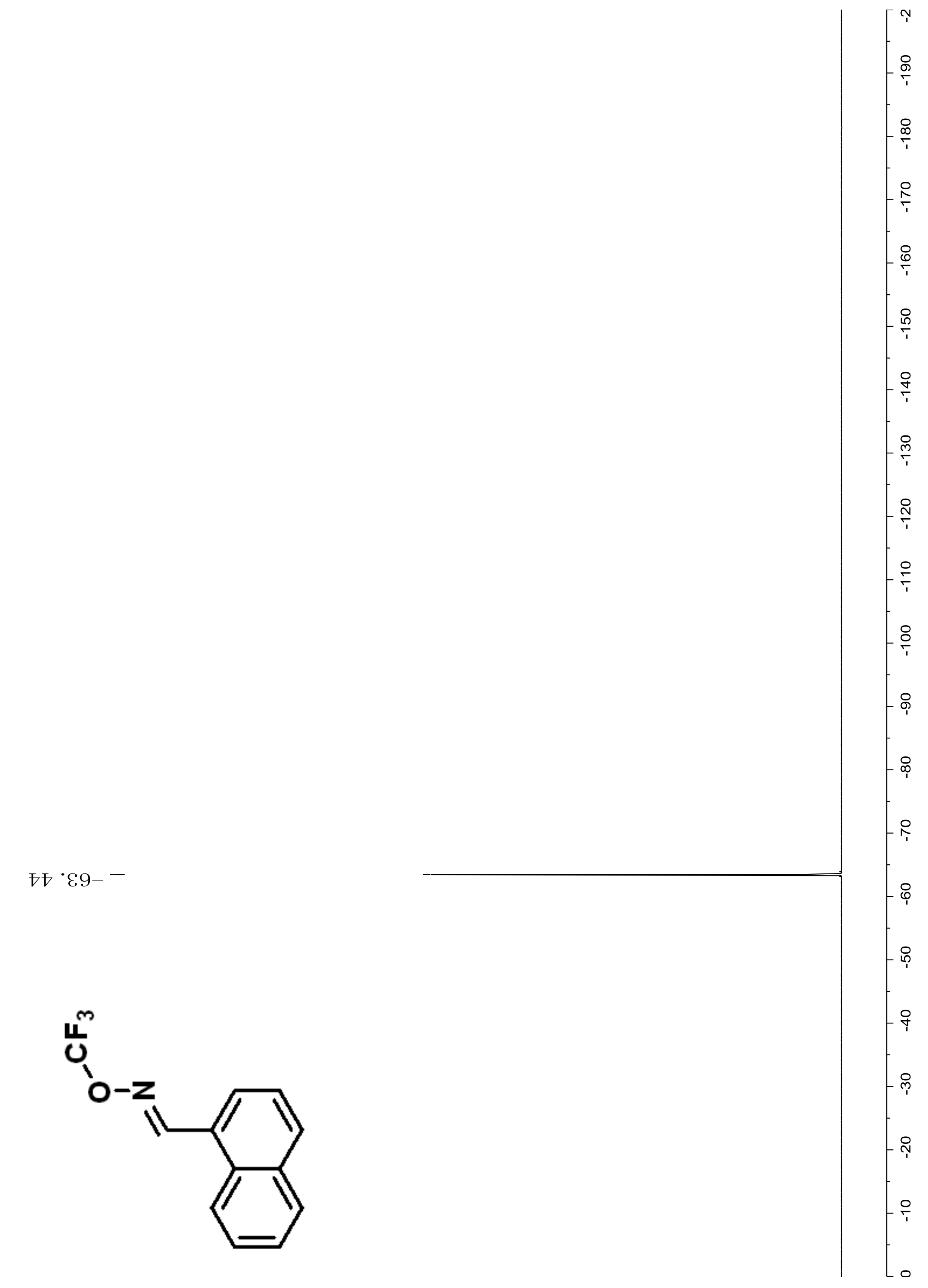

Supplementary Figure 55: ^{19}F NMR spectrum (376 MHz, DMSO, 23 °C) of **1q**

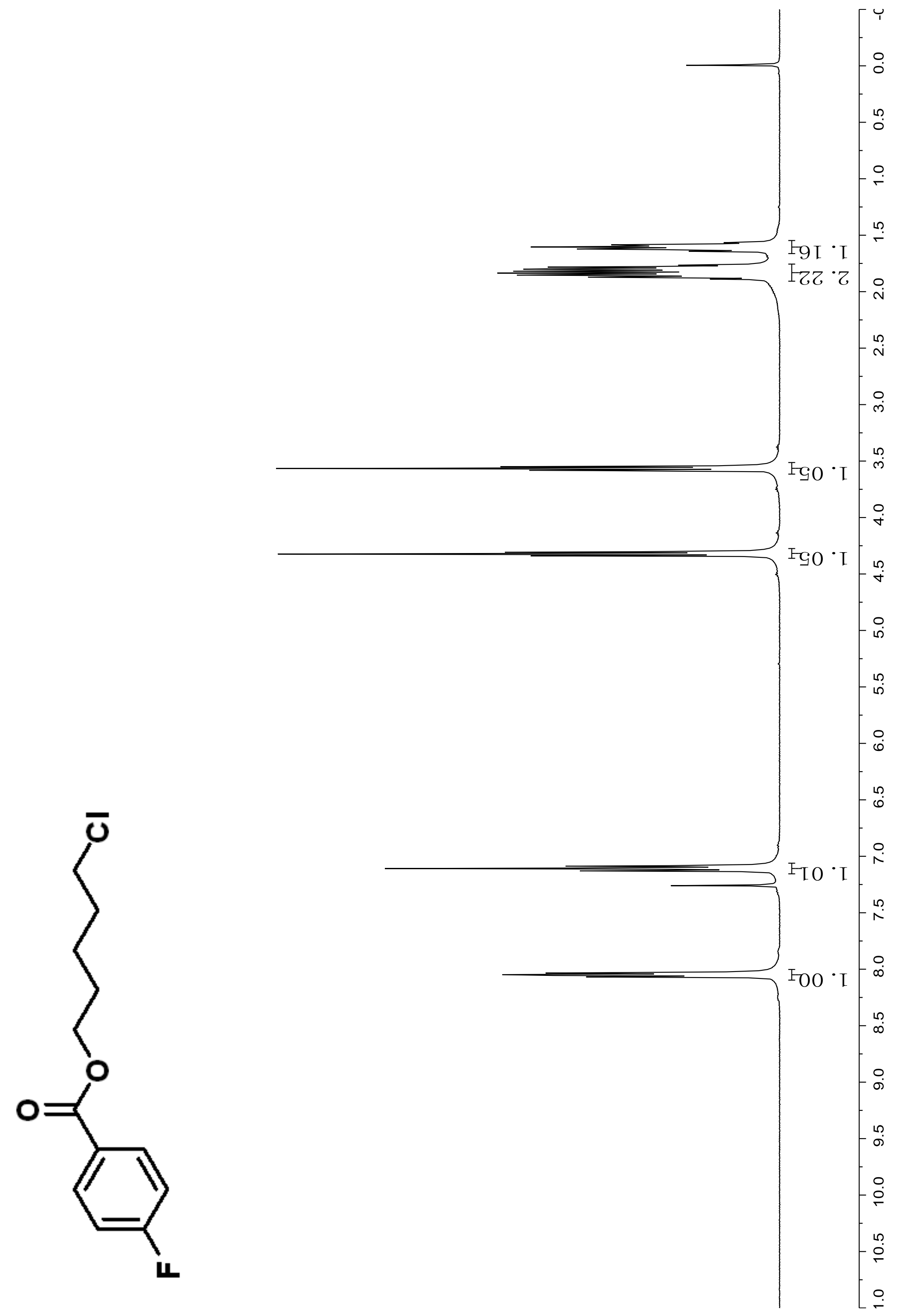

Supplementary Figure 56: ^{1}H NMR spectrum (400 MHz, $CDCl_3$, 23 °C) of **S3-1**

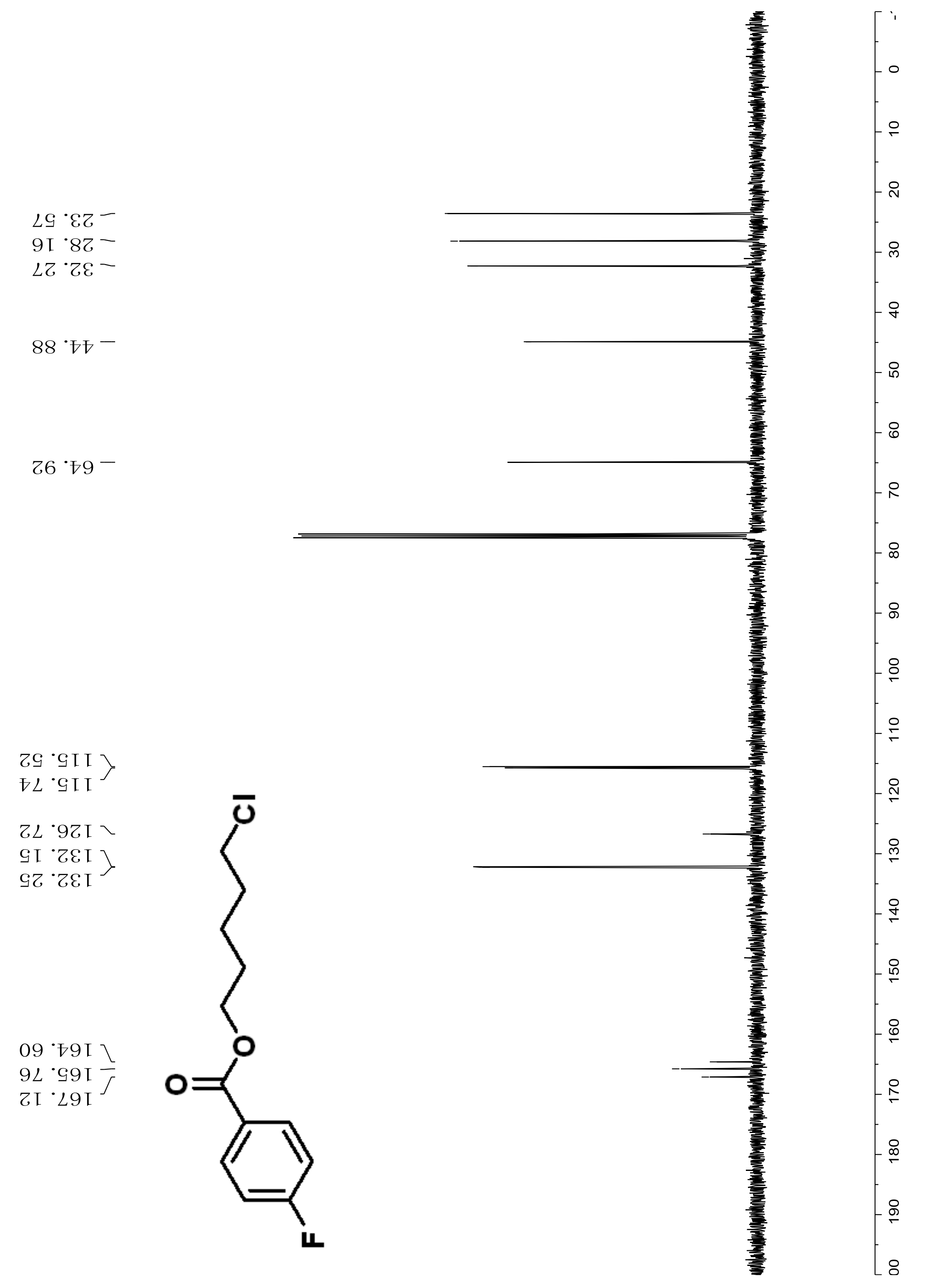

Supplementary Figure 57: ^{13}C NMR spectrum (101 MHz, $CDCl_3$, 23 °C) of **S3-1**

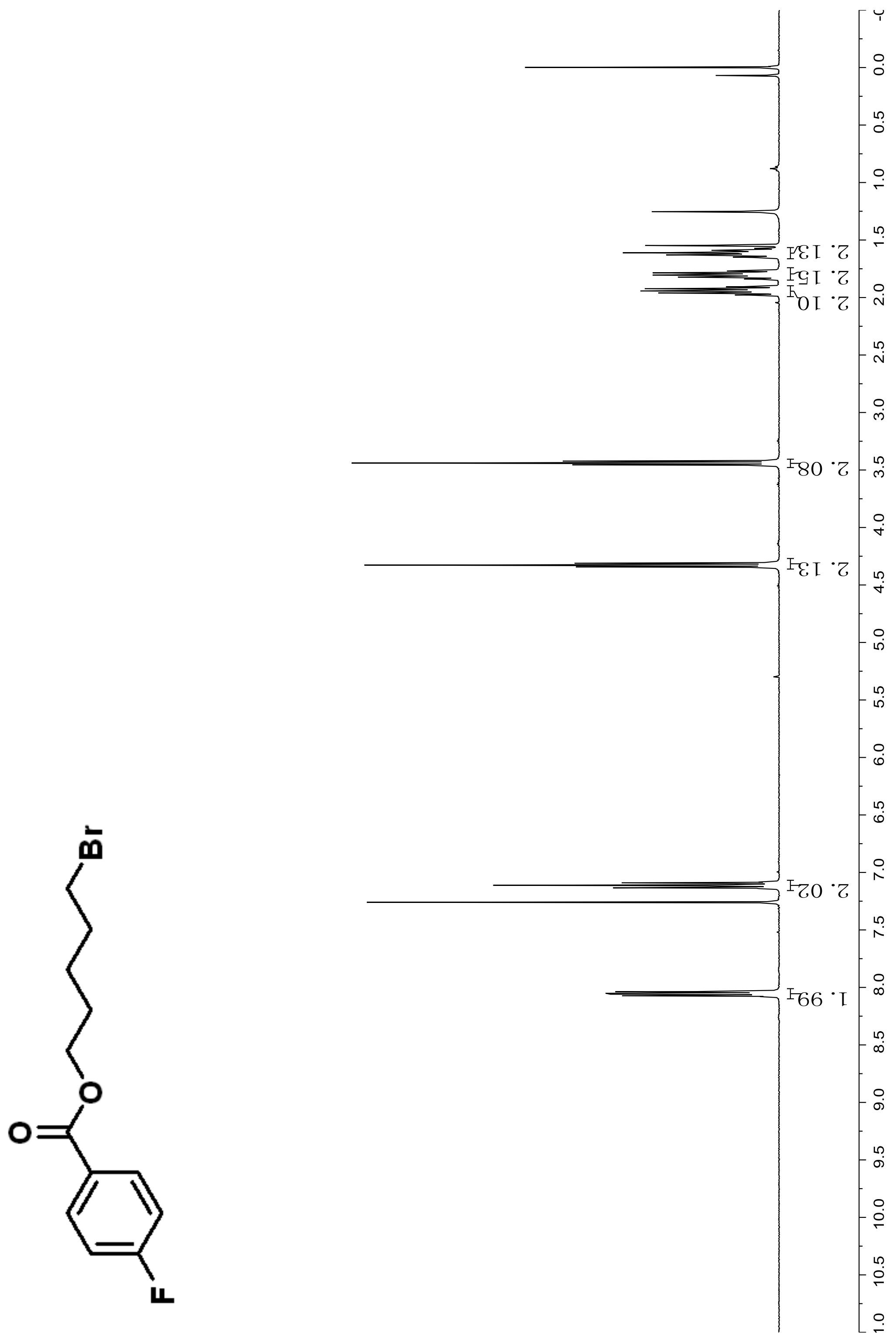

Supplementary Figure 58: ^{1}H NMR spectrum (400 MHz, $CDCl_3$, 23 °C) of **S3-2**

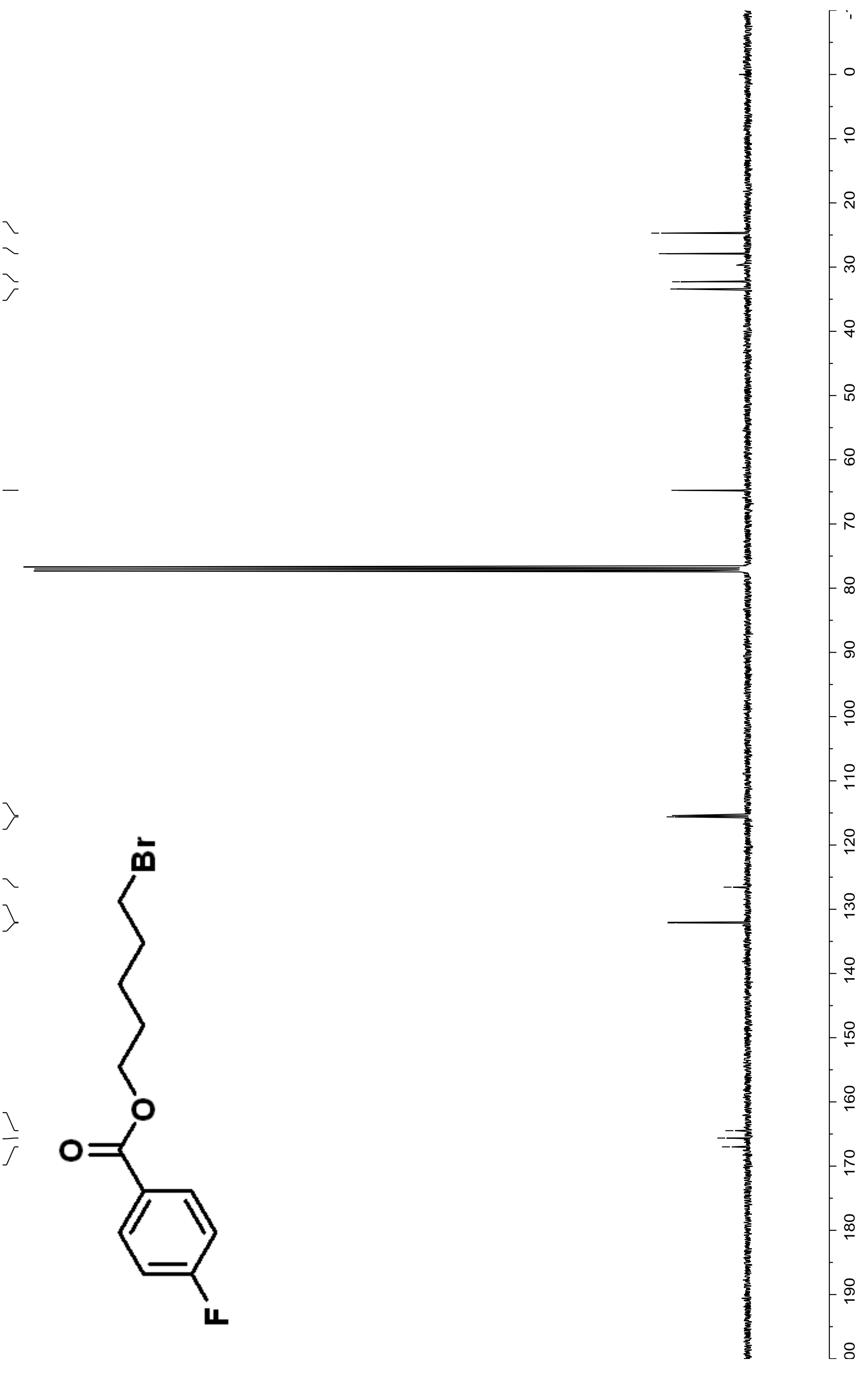

Supplementary Figure 59: ^{13}C NMR spectrum (101 MHz, $CDCl_3$, 23 °C) of **S3-2**

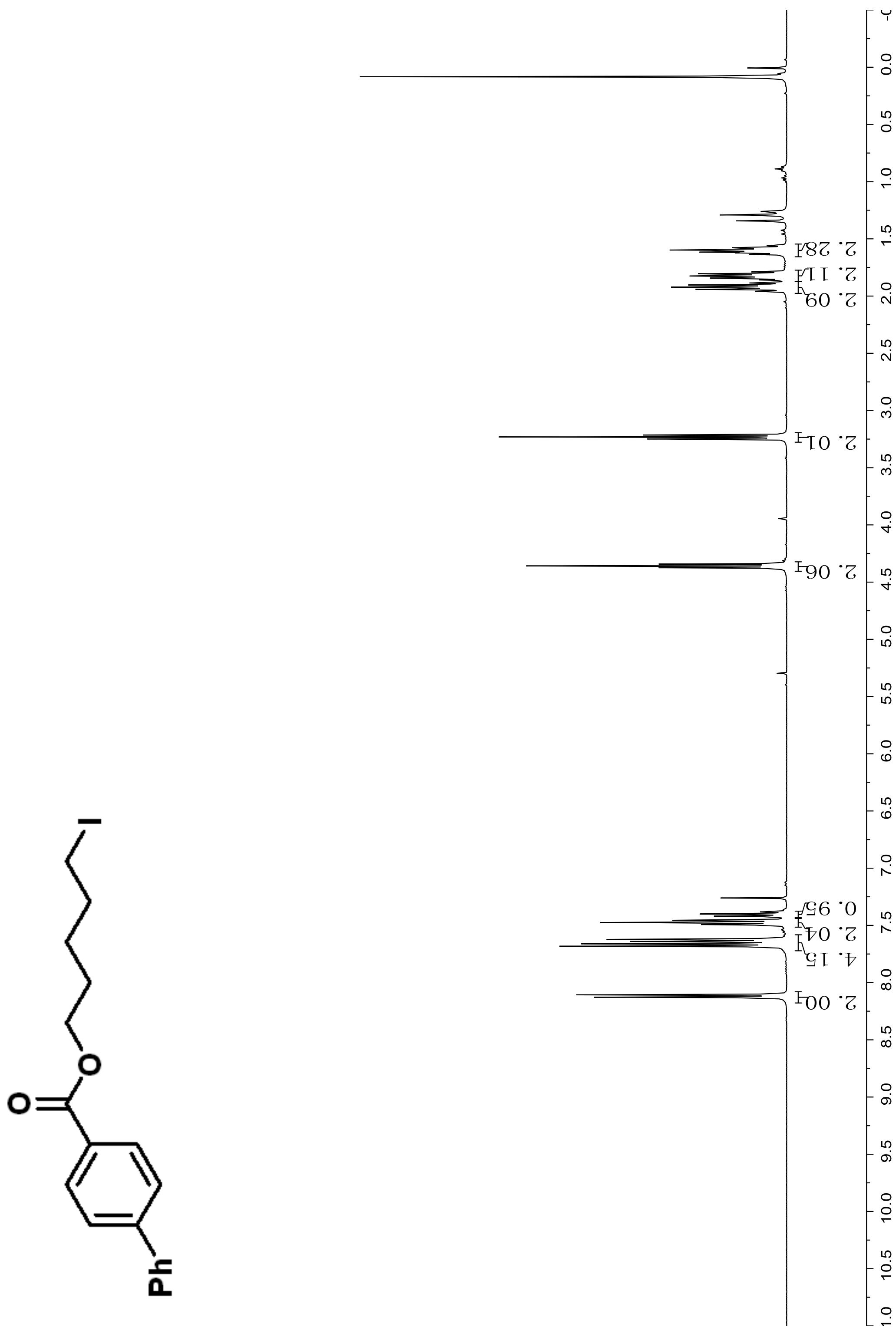

Supplementary Figure 60: ^{1}H NMR spectrum (400 MHz, $CDCl_3$, 23 °C) of **S13**

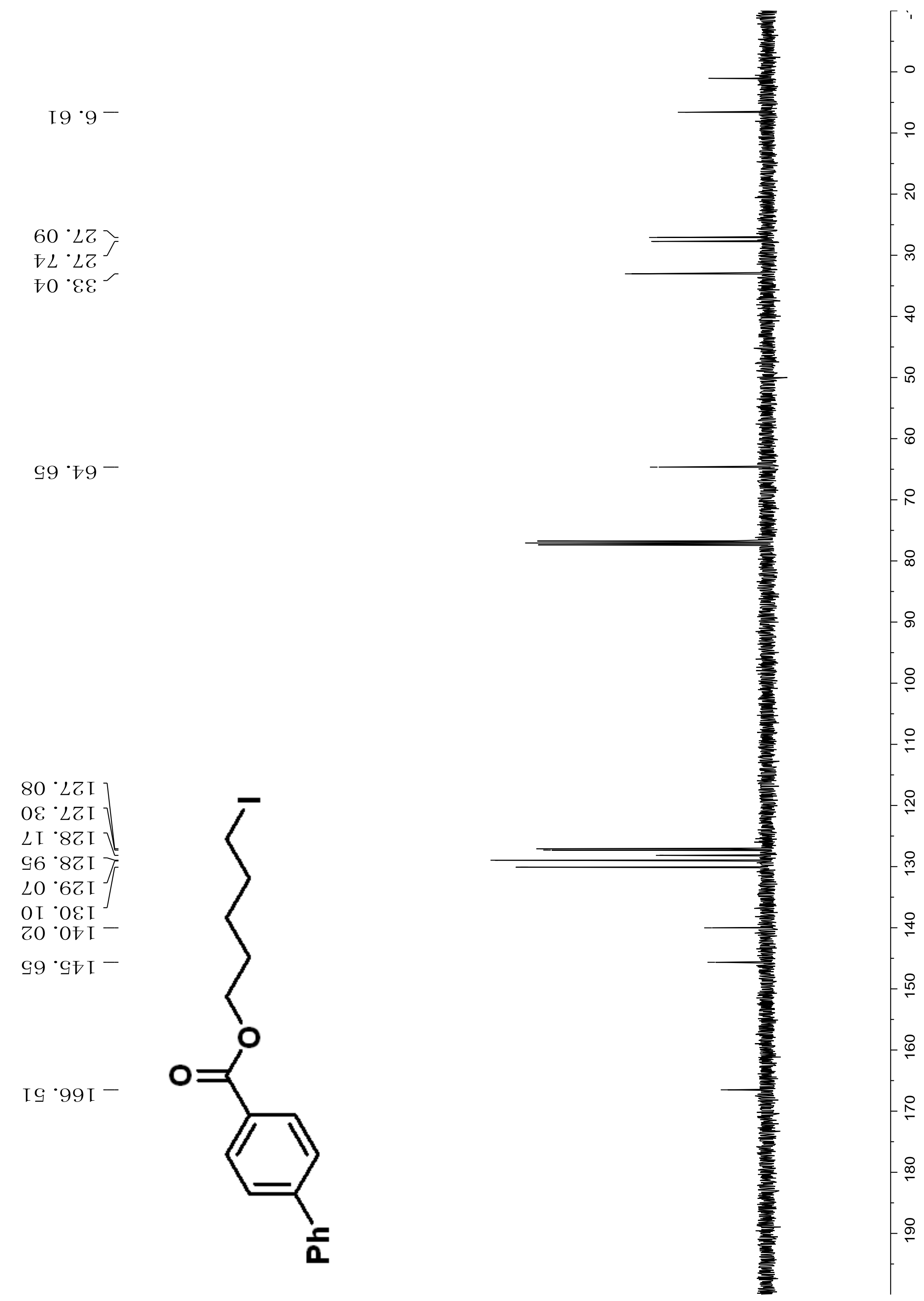

Supplementary Figure 61: ^{13}C NMR spectrum (101 MHz, $CDCl_3$, 23 °C) of **S13**

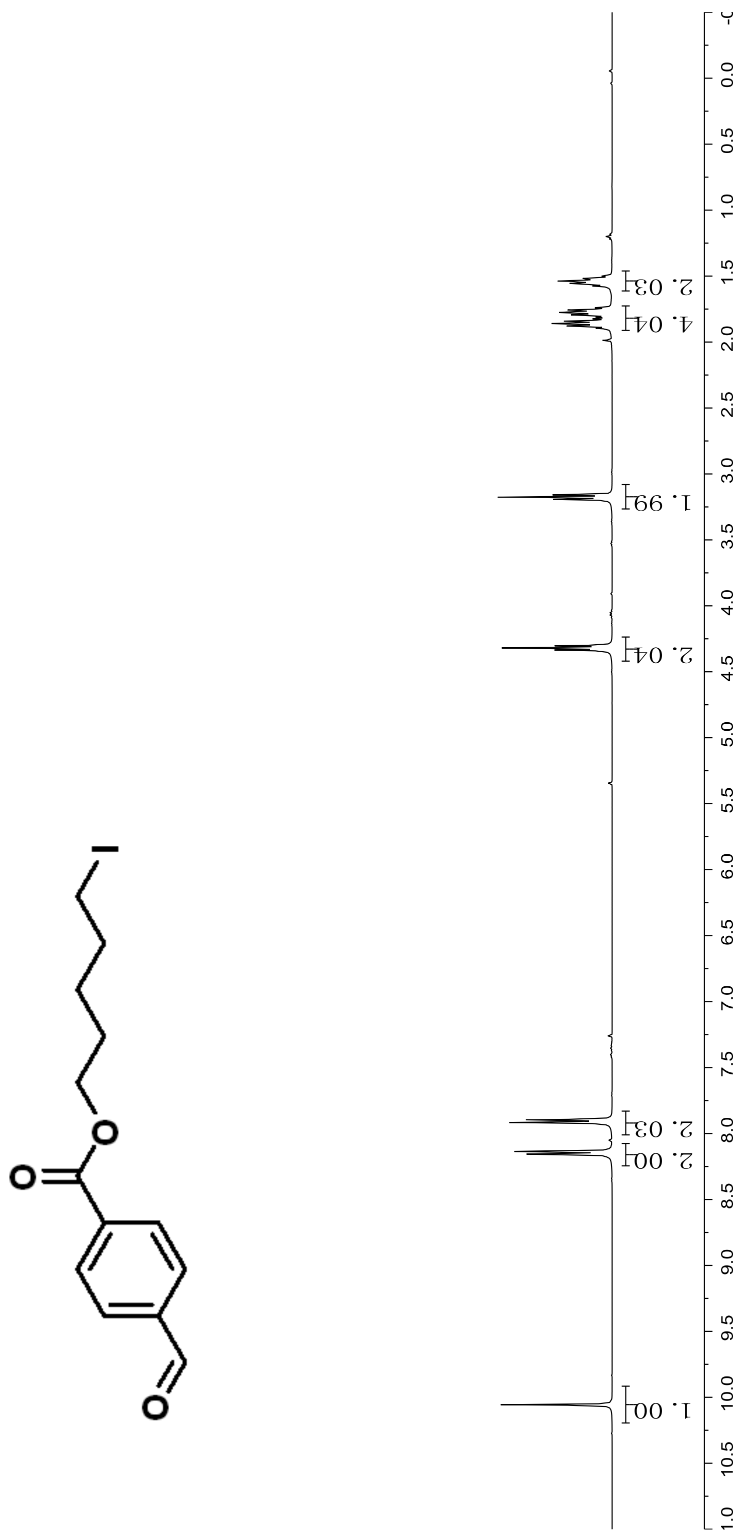

Supplementary Figure 62: ^{1}H NMR spectrum (400 MHz, $CDCl_3$, 23 °C) of **S14**

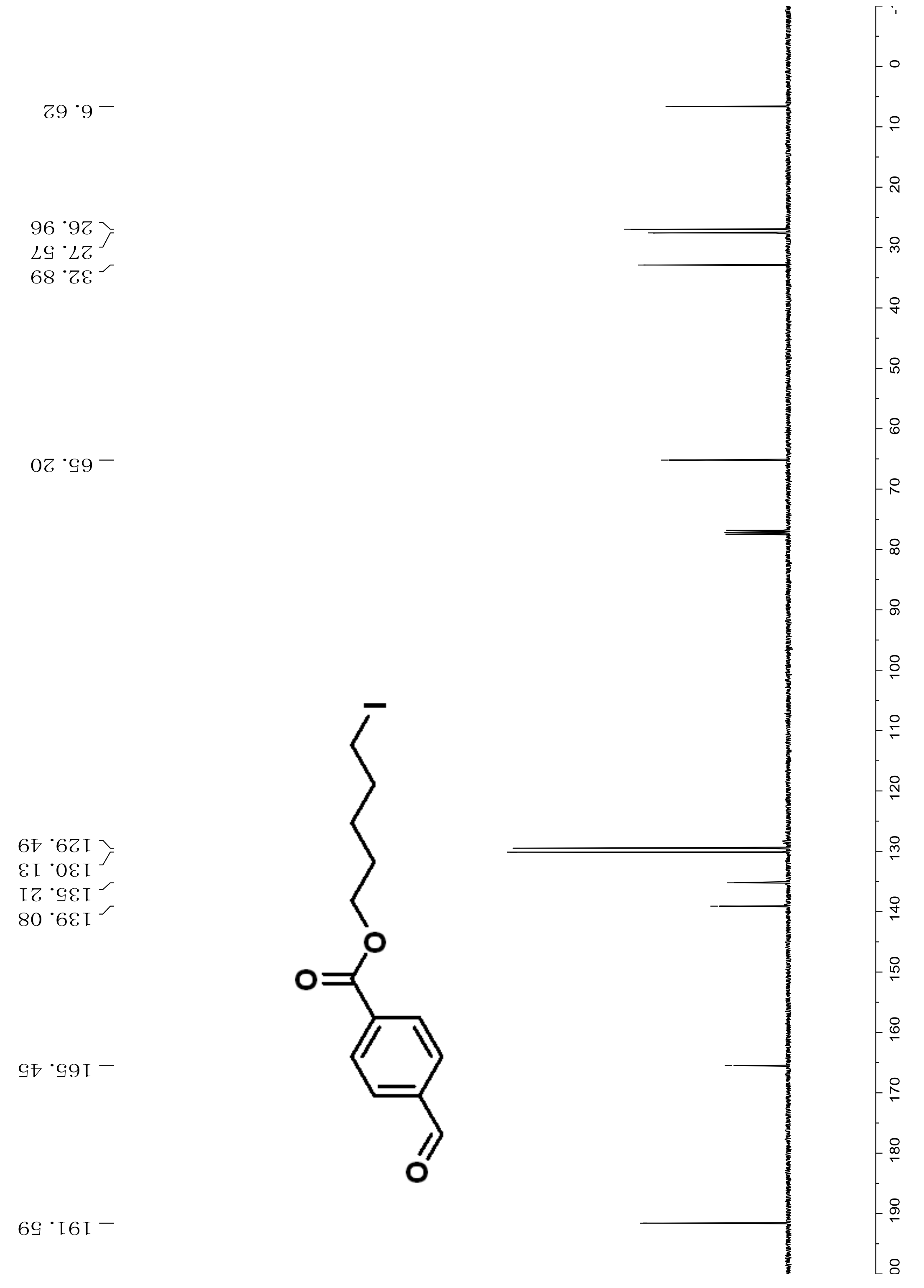

Supplementary Figure 63: ^{13}C NMR spectrum (101 MHz, $CDCl_3$, 23 °C) of **S14**

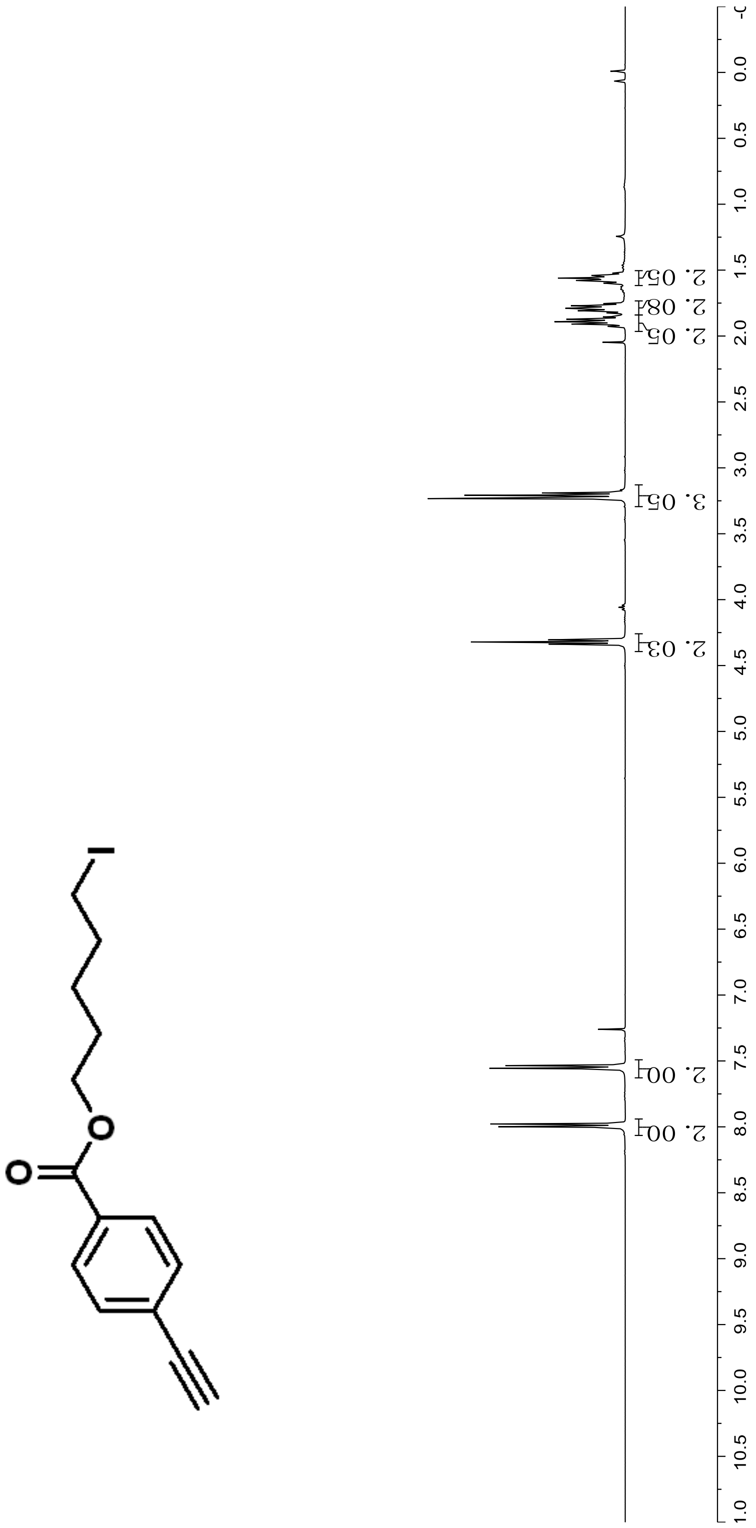

Supplementary Figure 64: ^{1}H NMR spectrum (400 MHz, $CDCl_3$, 23 °C) of **S15**

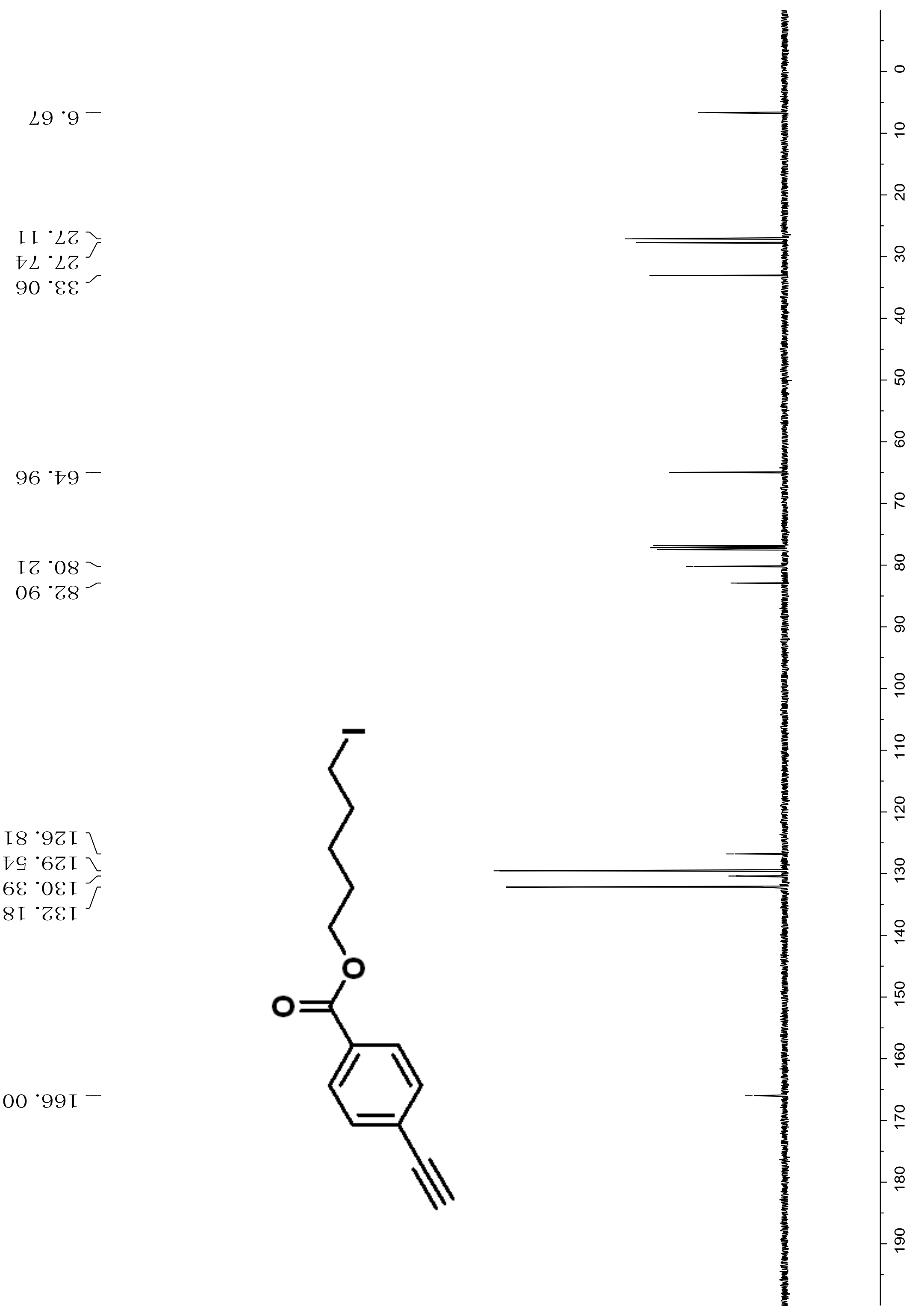

Supplementary Figure 65: ^{13}C NMR spectrum (101 MHz, $CDCl_3$, 23 °C) of **S15**

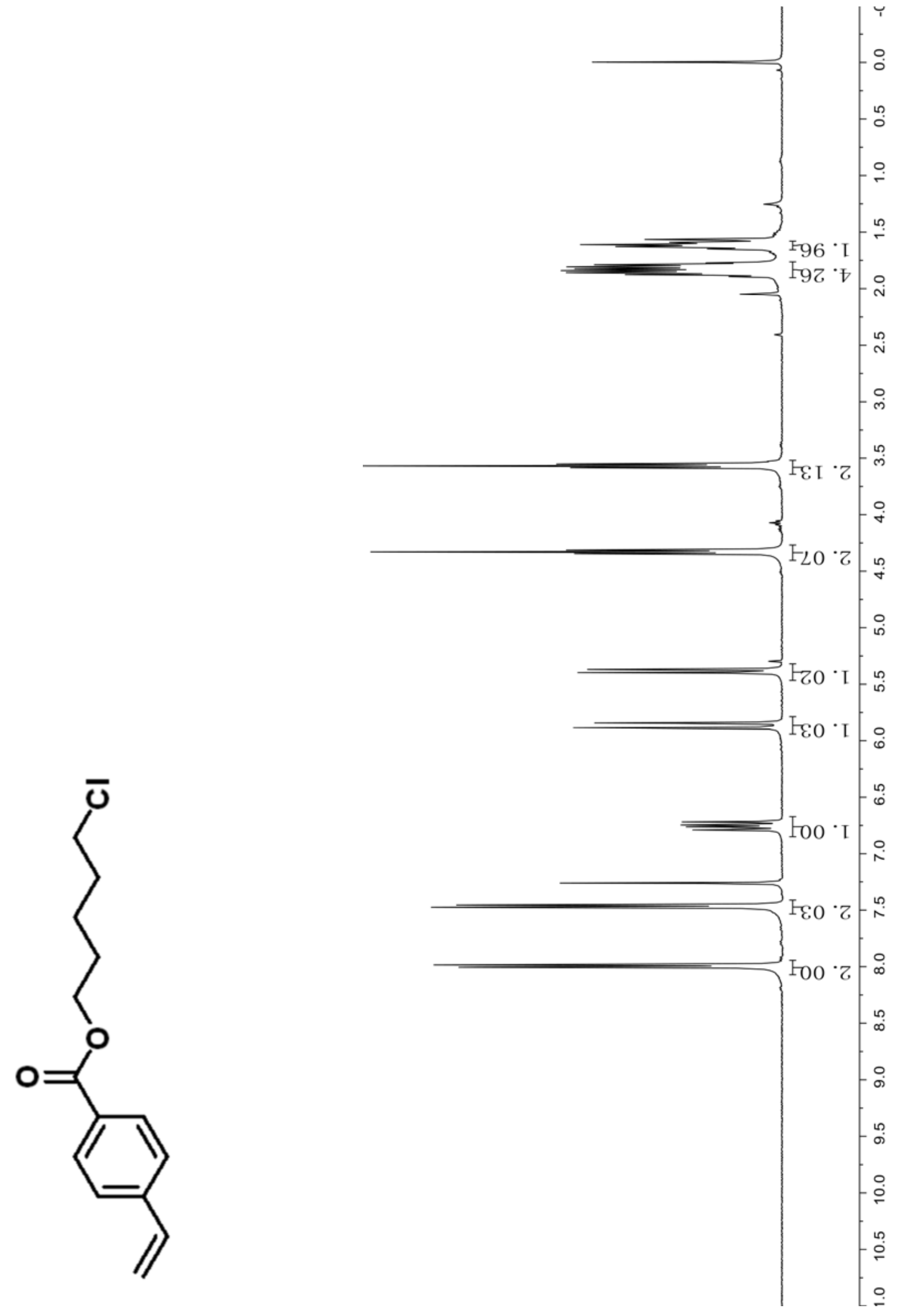

Supplementary Figure 66: ^{1}H NMR spectrum (400 MHz, $CDCl_3$, 23 °C) of **S16-1**

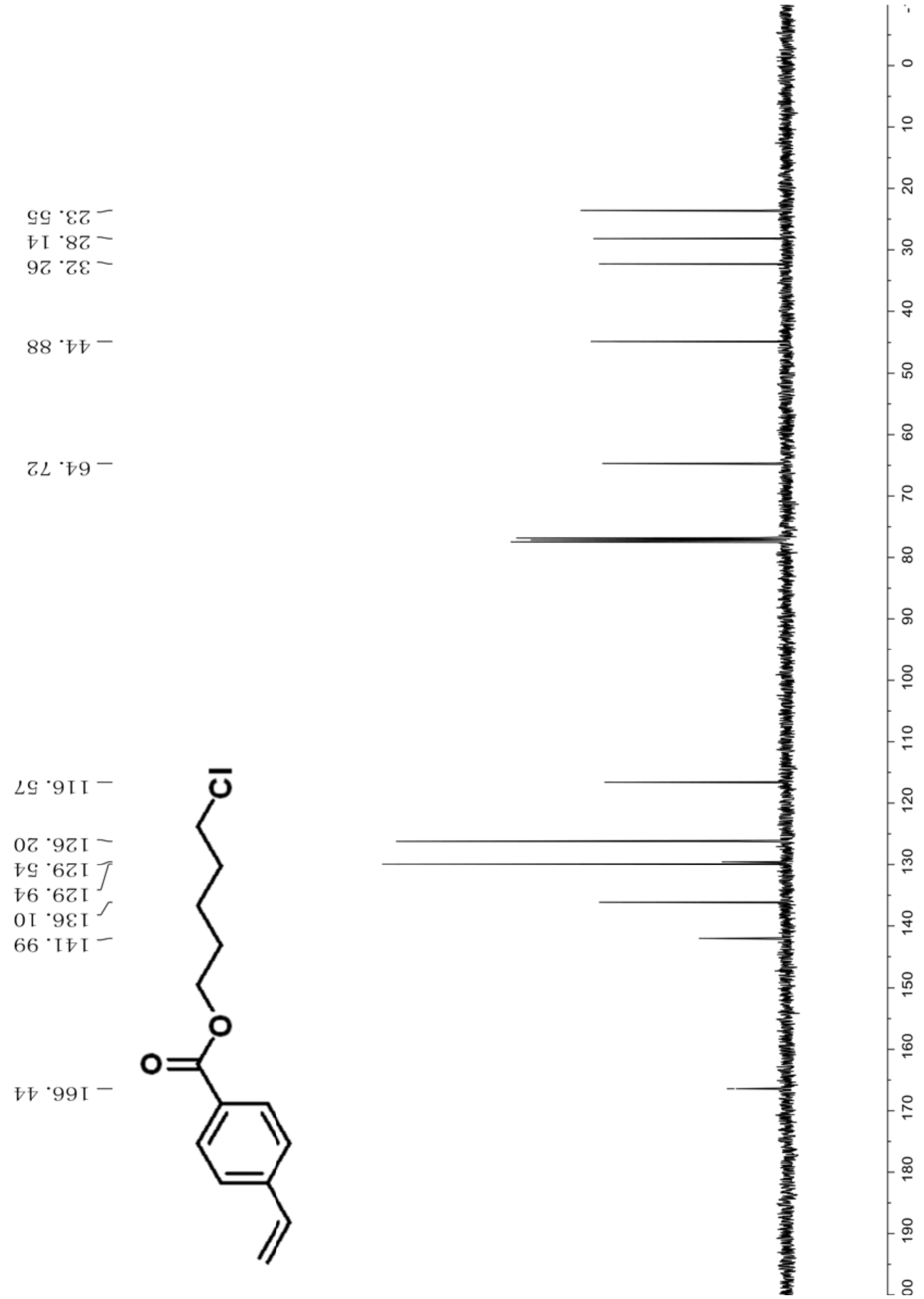

Supplementary Figure 67: ^{13}C NMR spectrum (101 MHz, $CDCl_3$, 23 °C) of **S16-1**

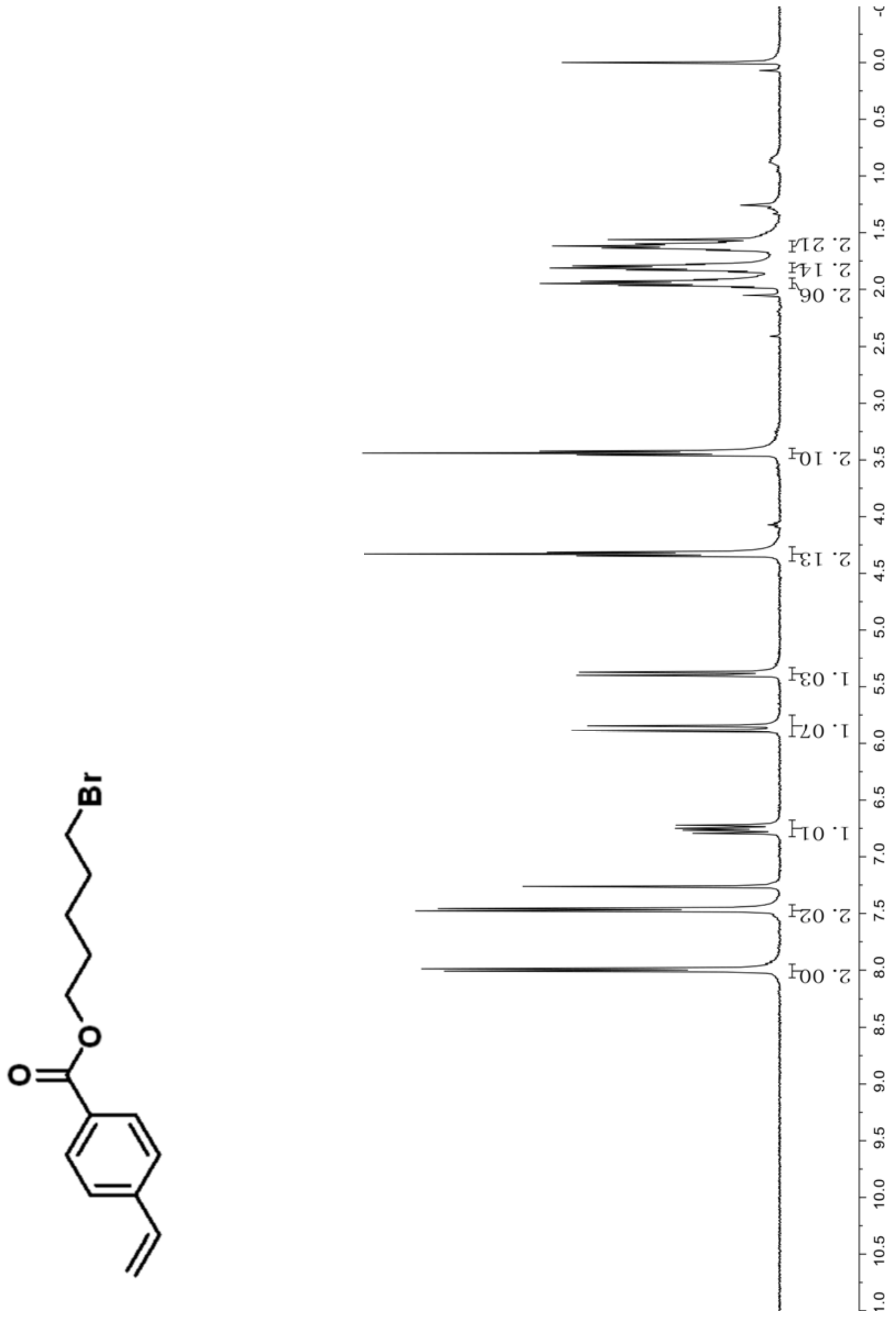

Supplementary Figure 68: ^{1}H NMR spectrum (400 MHz, $CDCl_3$, 23 °C) of **S16-2**

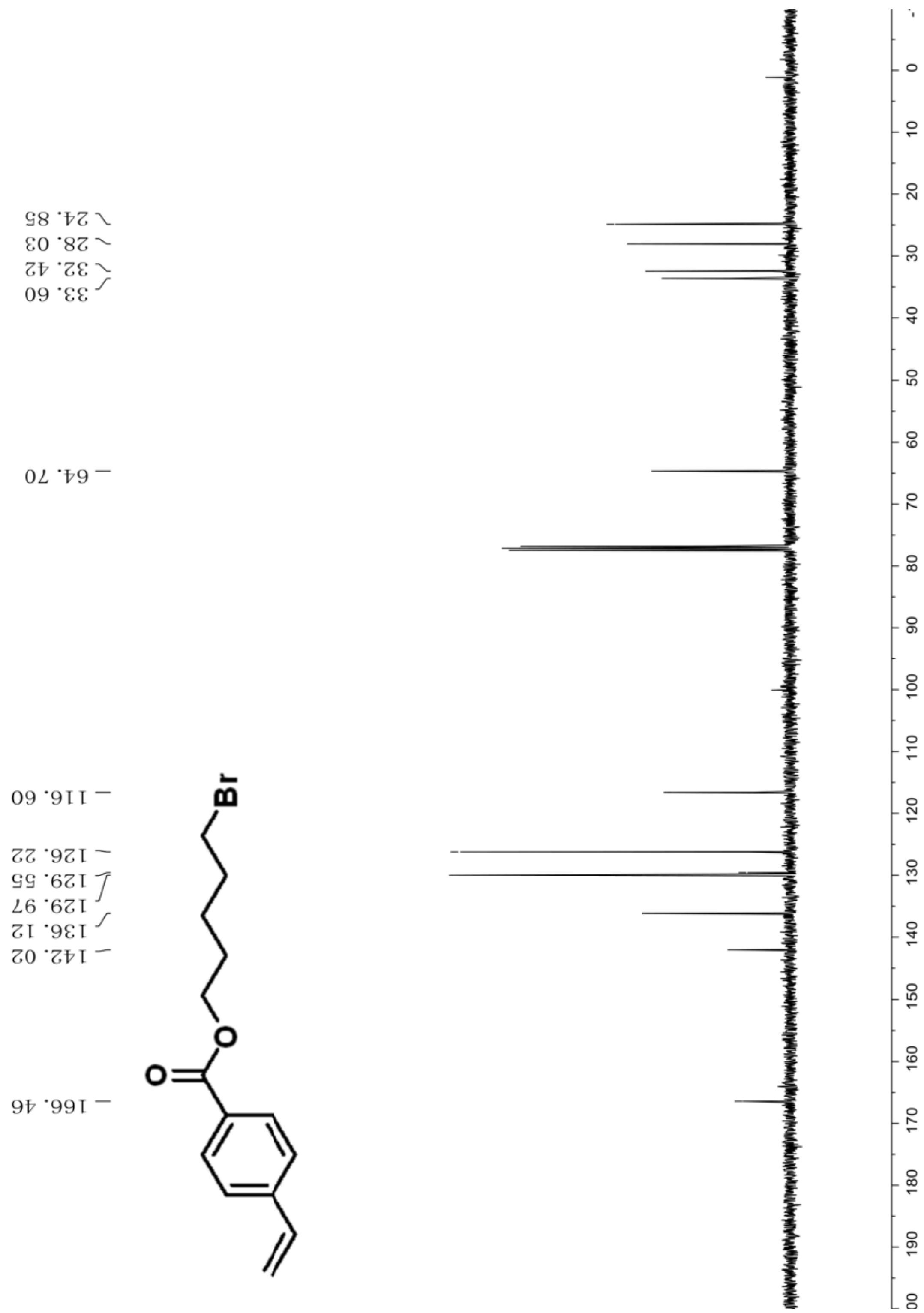

Supplementary Figure 69: ^{13}C NMR spectrum (101 MHz, $CDCl_3$, 23 °C) of **S16-2**

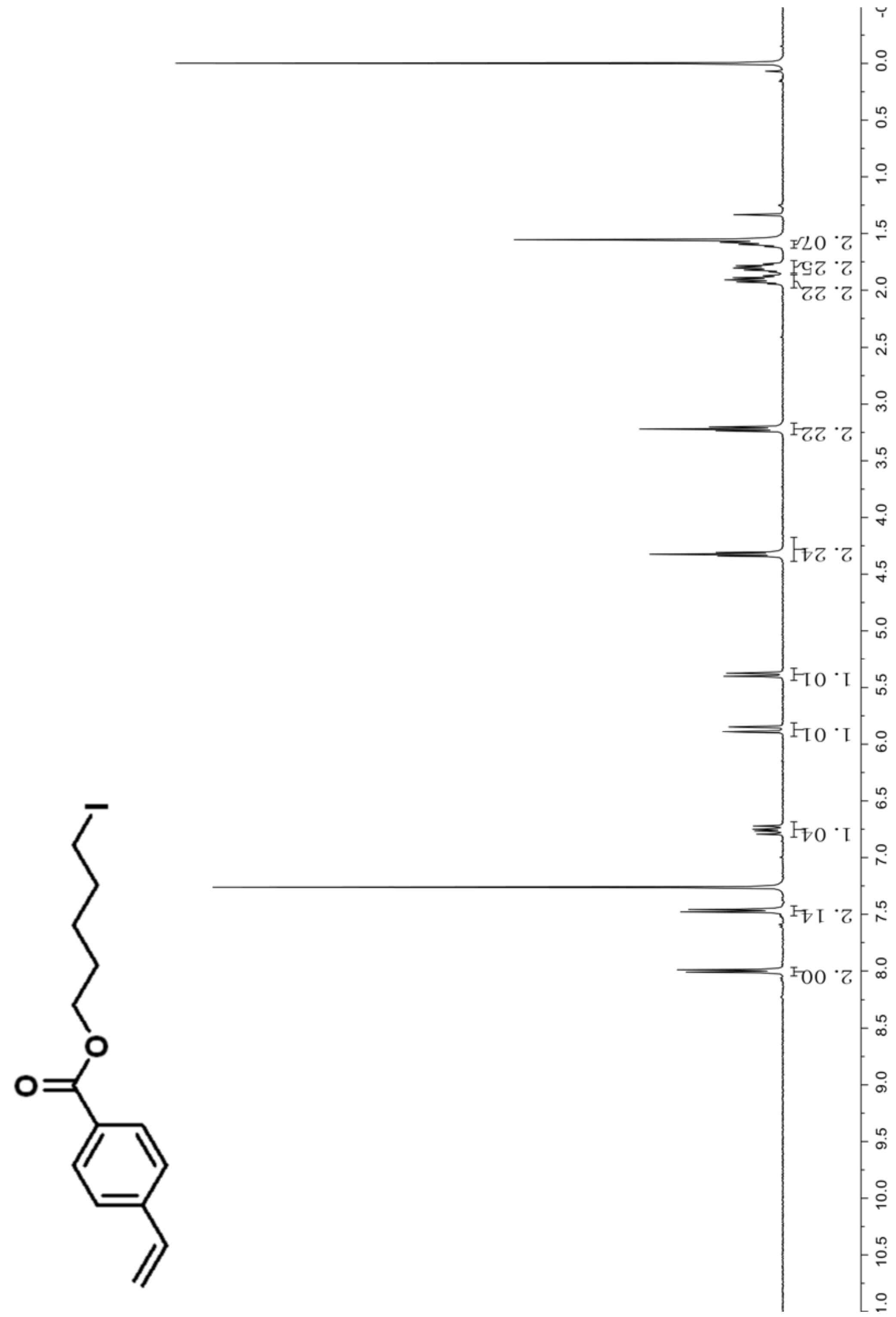

Supplementary Figure 70: ^{1}H NMR spectrum (400 MHz, $CDCl_3$, 23 °C) of **S16**

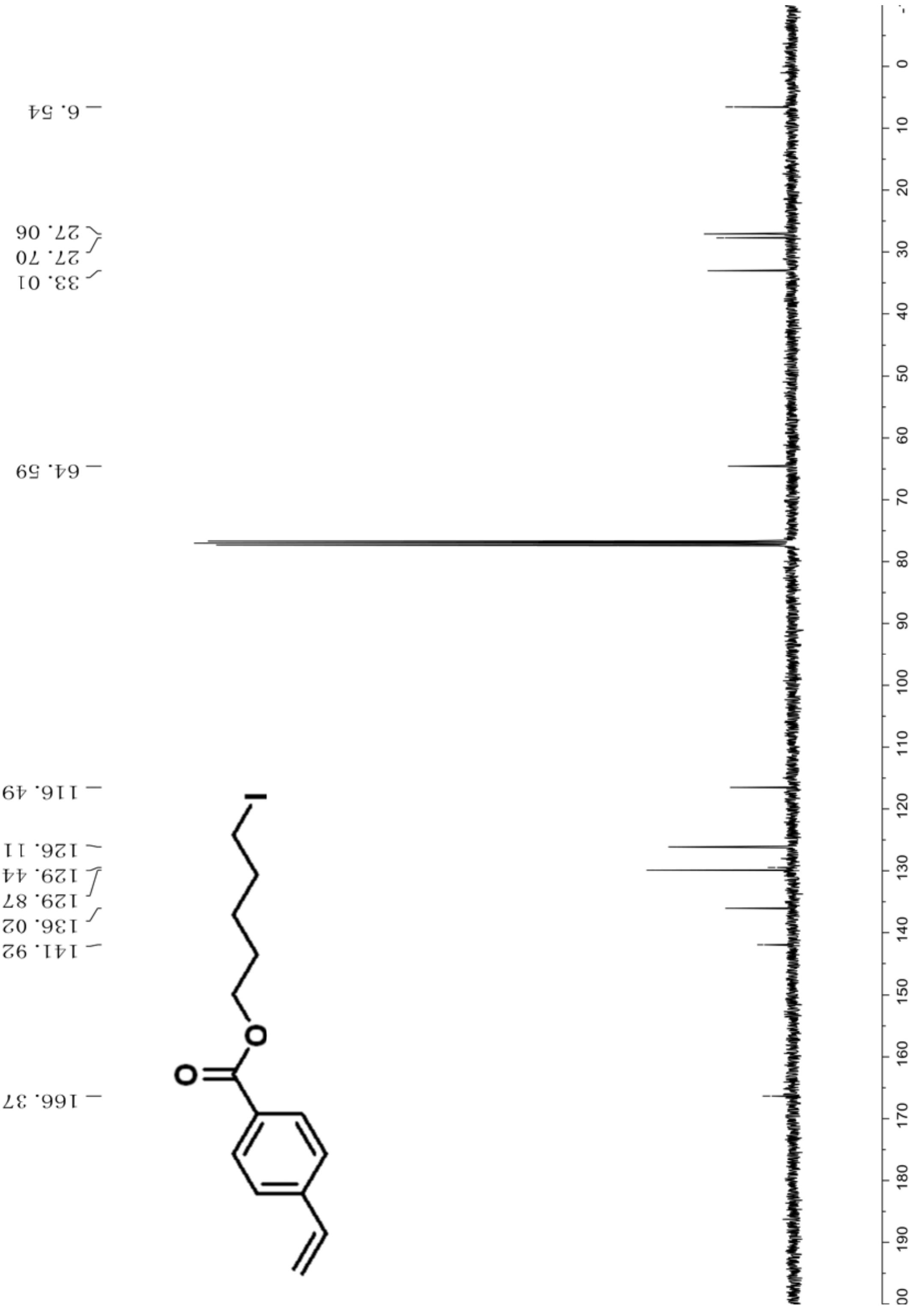

Supplementary Figure 71: ^{13}C NMR spectrum (101 MHz, $CDCl_3$, 23 °C) of **S16**

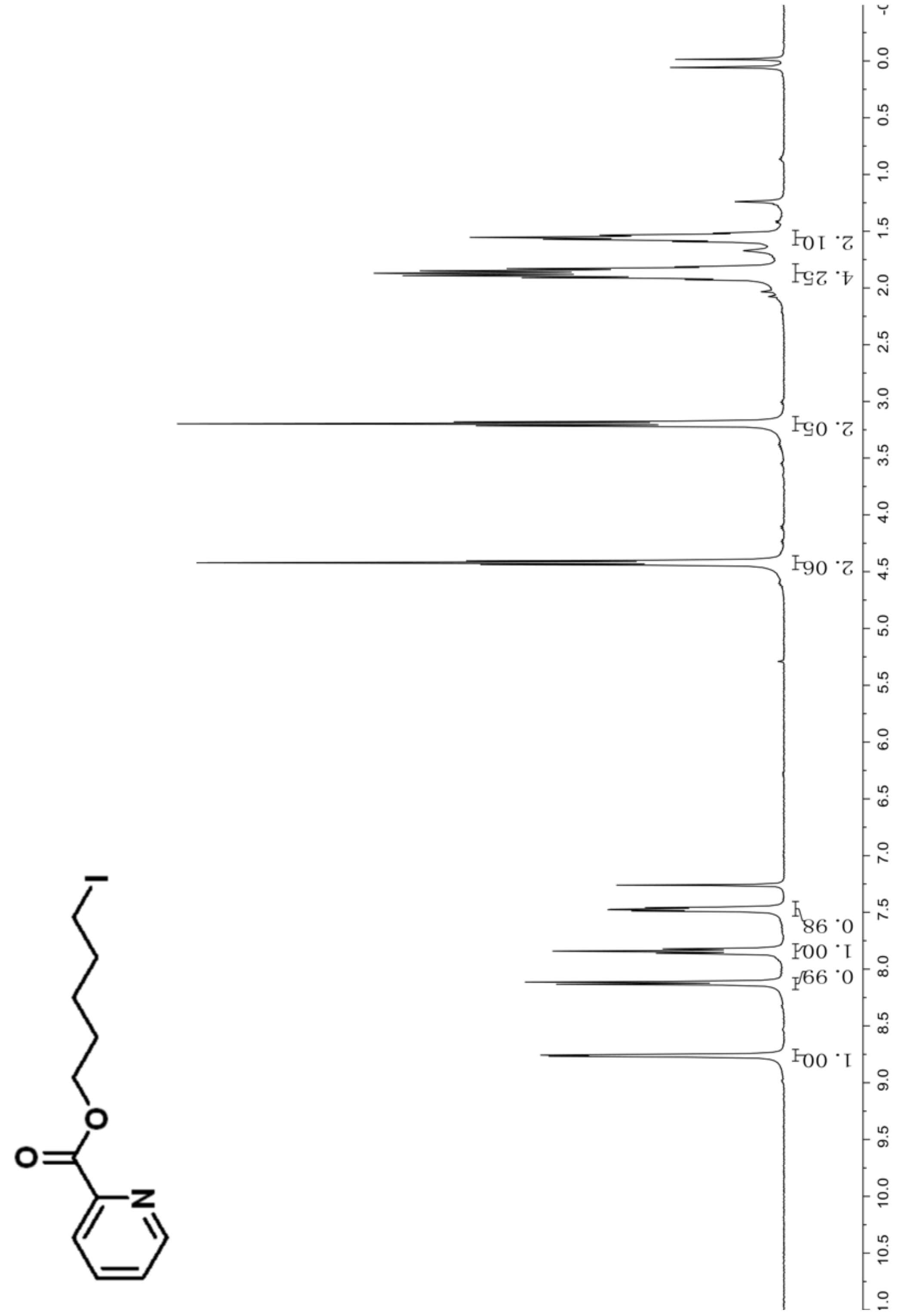

Supplementary Figure 72: ^{1}H NMR spectrum (400 MHz, $CDCl_3$, 23 °C) of **S17**

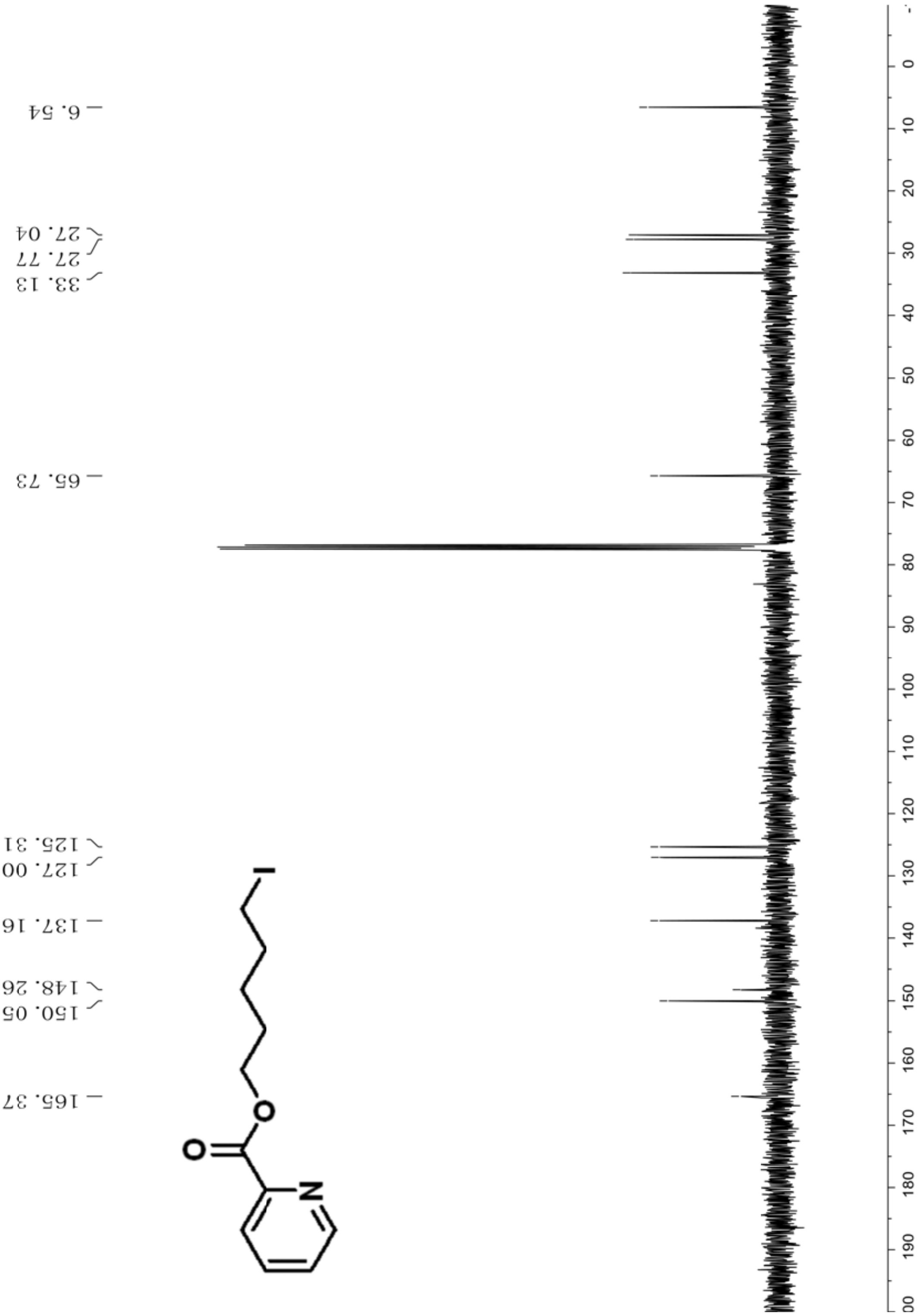

Supplementary Figure 73: ^{13}C NMR spectrum (101 MHz, $CDCl_3$, 23 °C) of **S17**

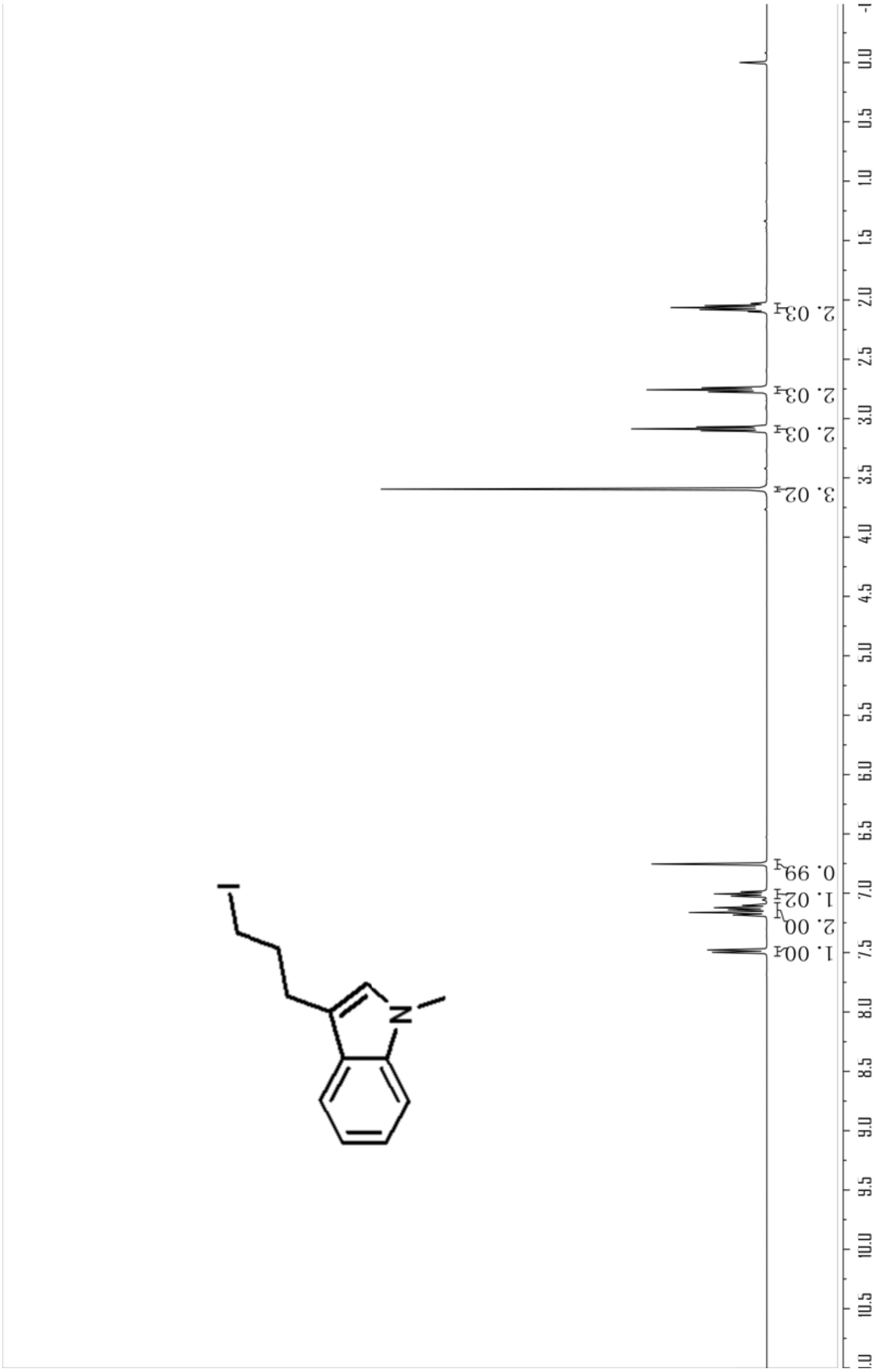

Supplementary Figure 74: ^{1}H NMR spectrum (400 MHz, $CDCl_3$, 23 °C) of **S23**

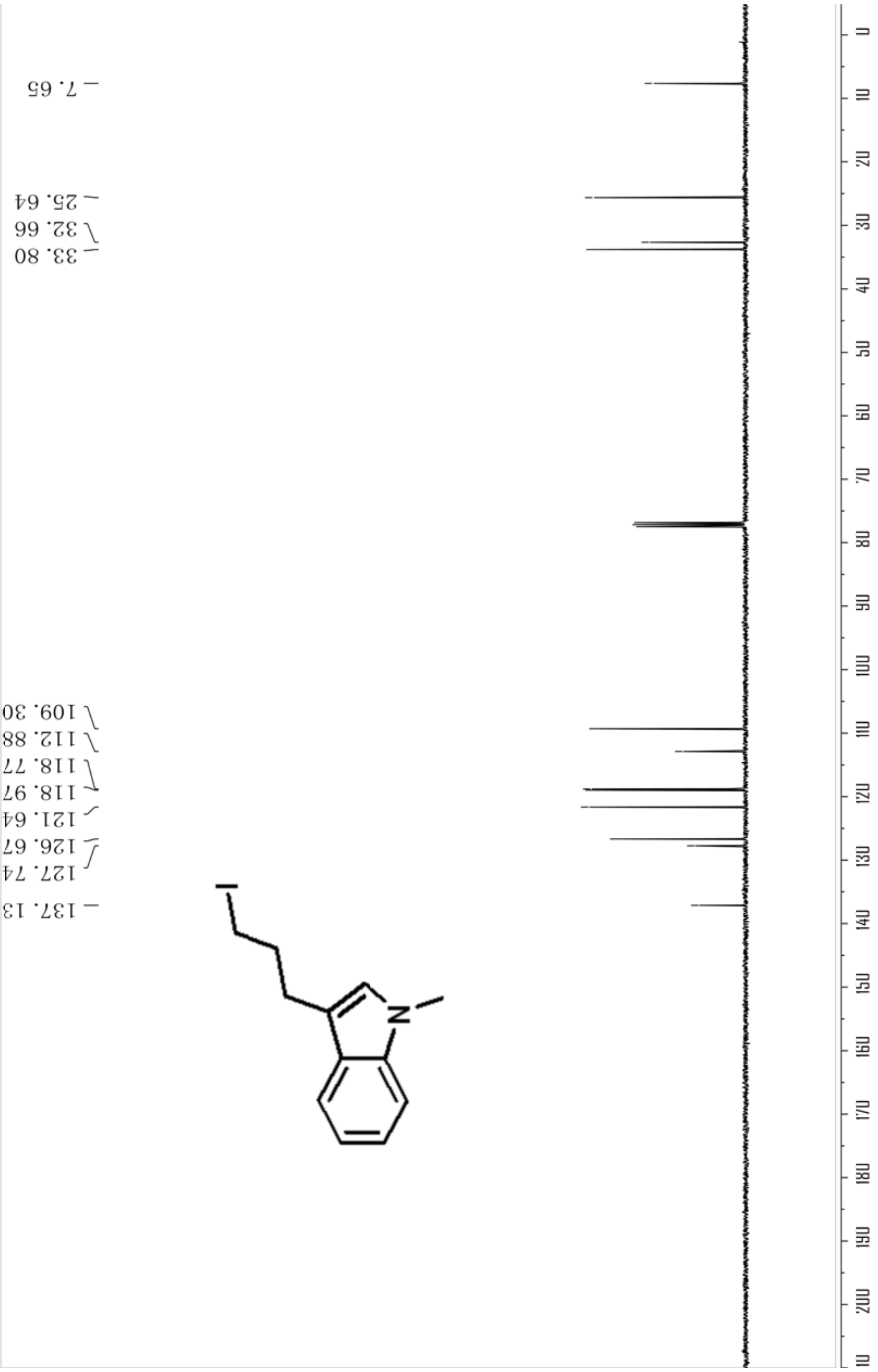

Supplementary Figure 75: ^{13}C NMR spectrum (101 MHz, $CDCl_3$, 23 °C) of **S23**

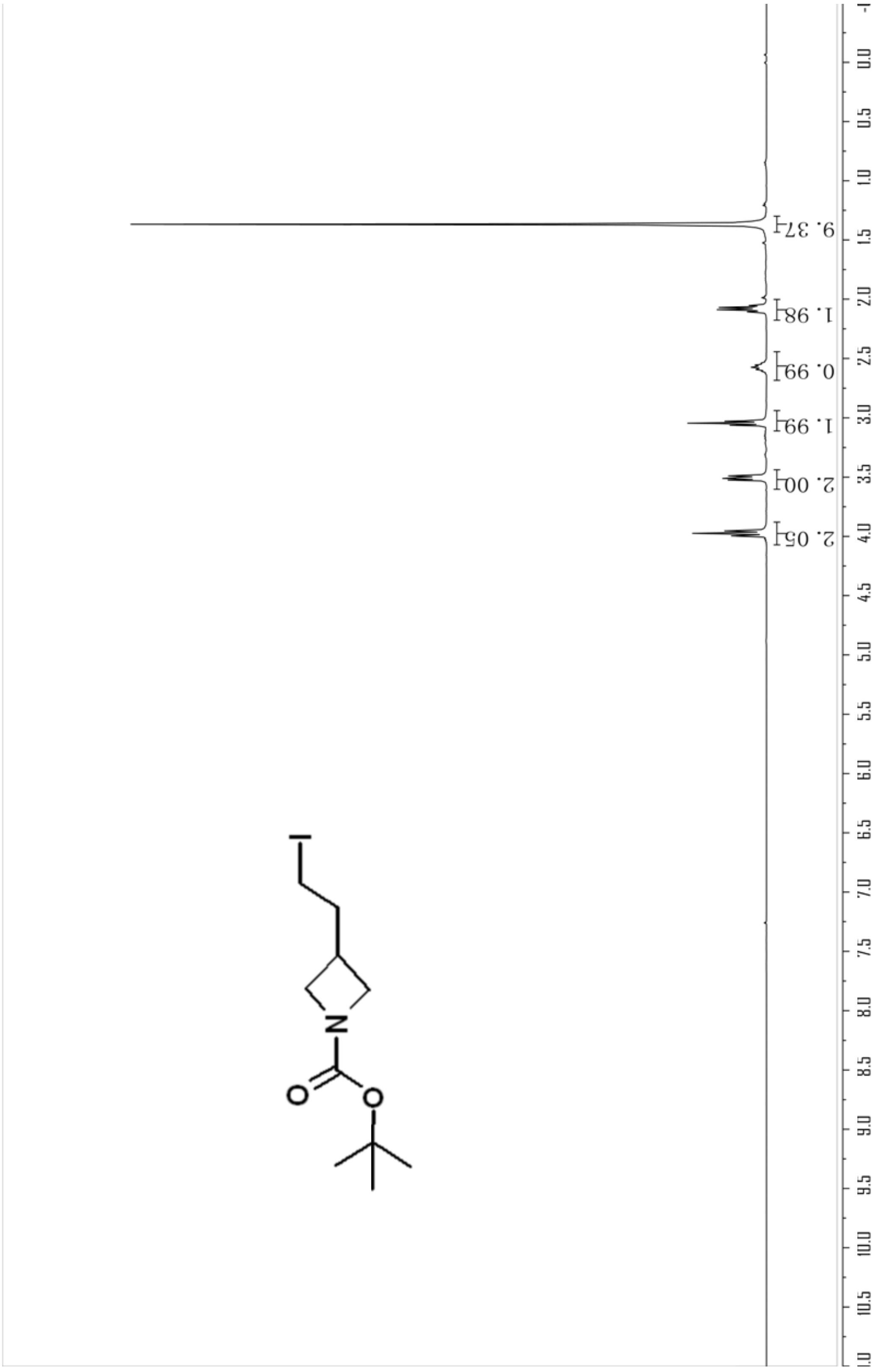

Supplementary Figure 76: ^{1}H NMR spectrum (400 MHz, $CDCl_3$, 23 °C) of **S25**

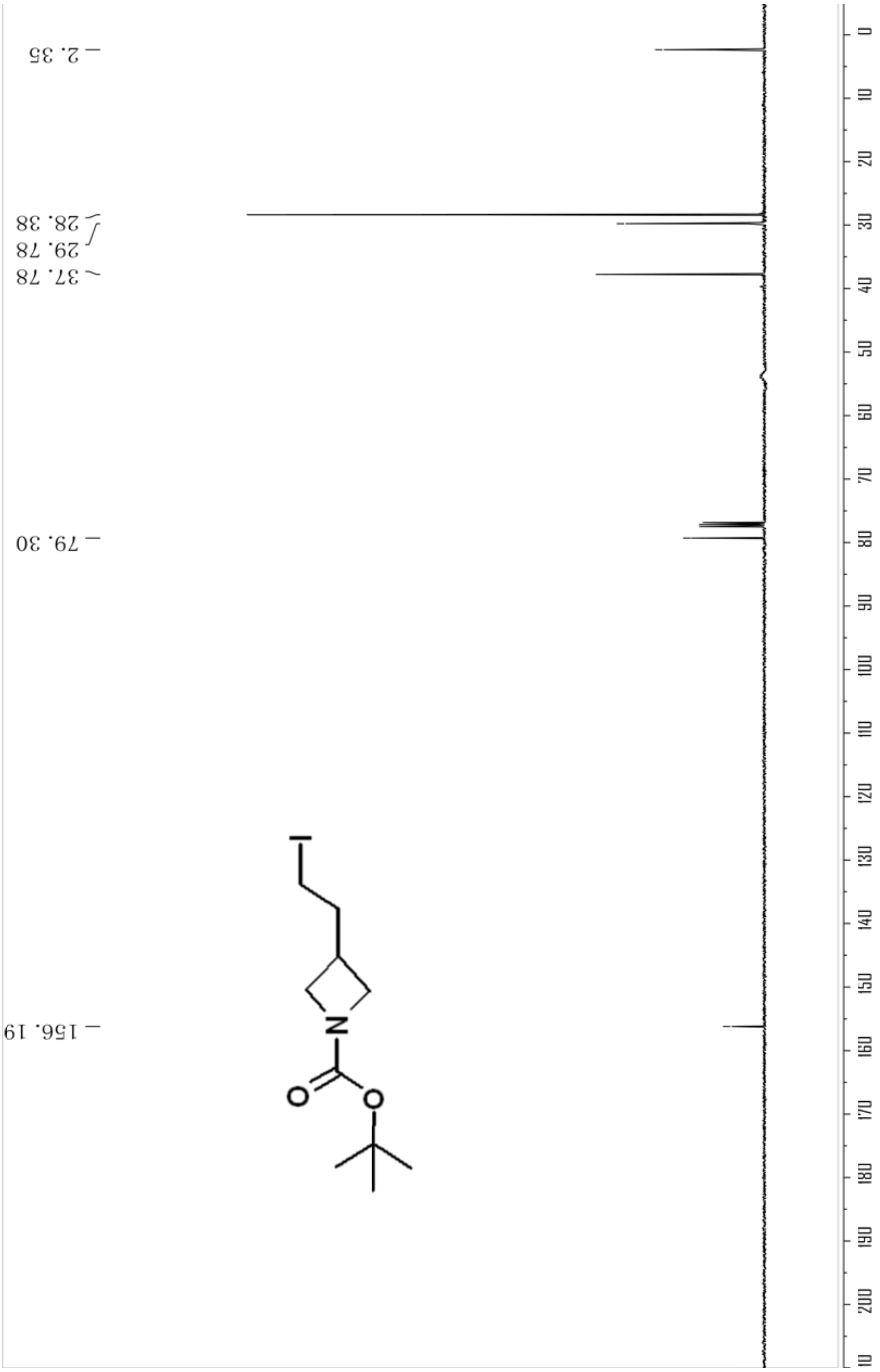

Supplementary Figure 77: ^{13}C NMR spectrum (101 MHz, $CDCl_3$, 23 °C) of **S25**

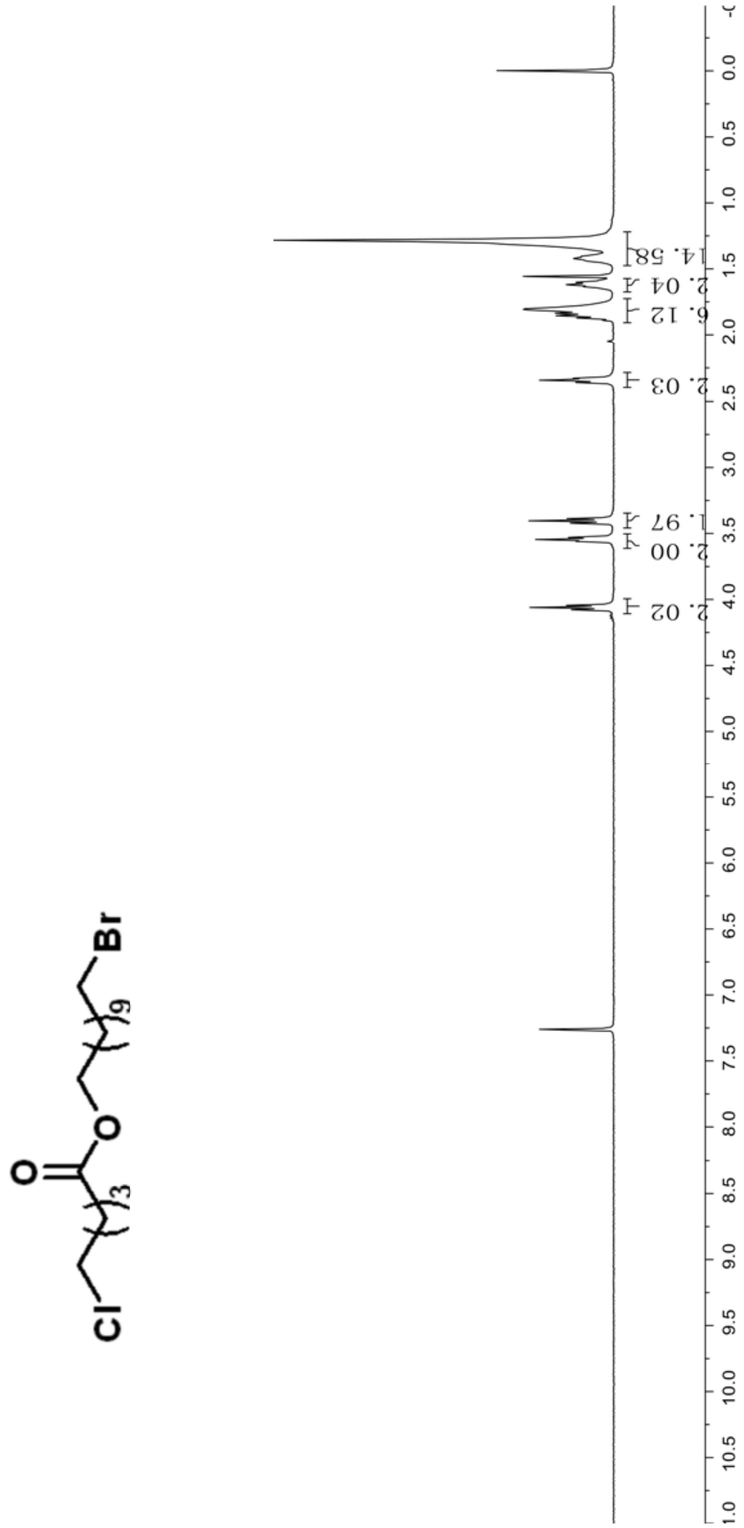

Supplementary Figure 78: ^{1}H NMR spectrum (400 MHz, $CDCl_3$, 23 °C) of **S29-1**

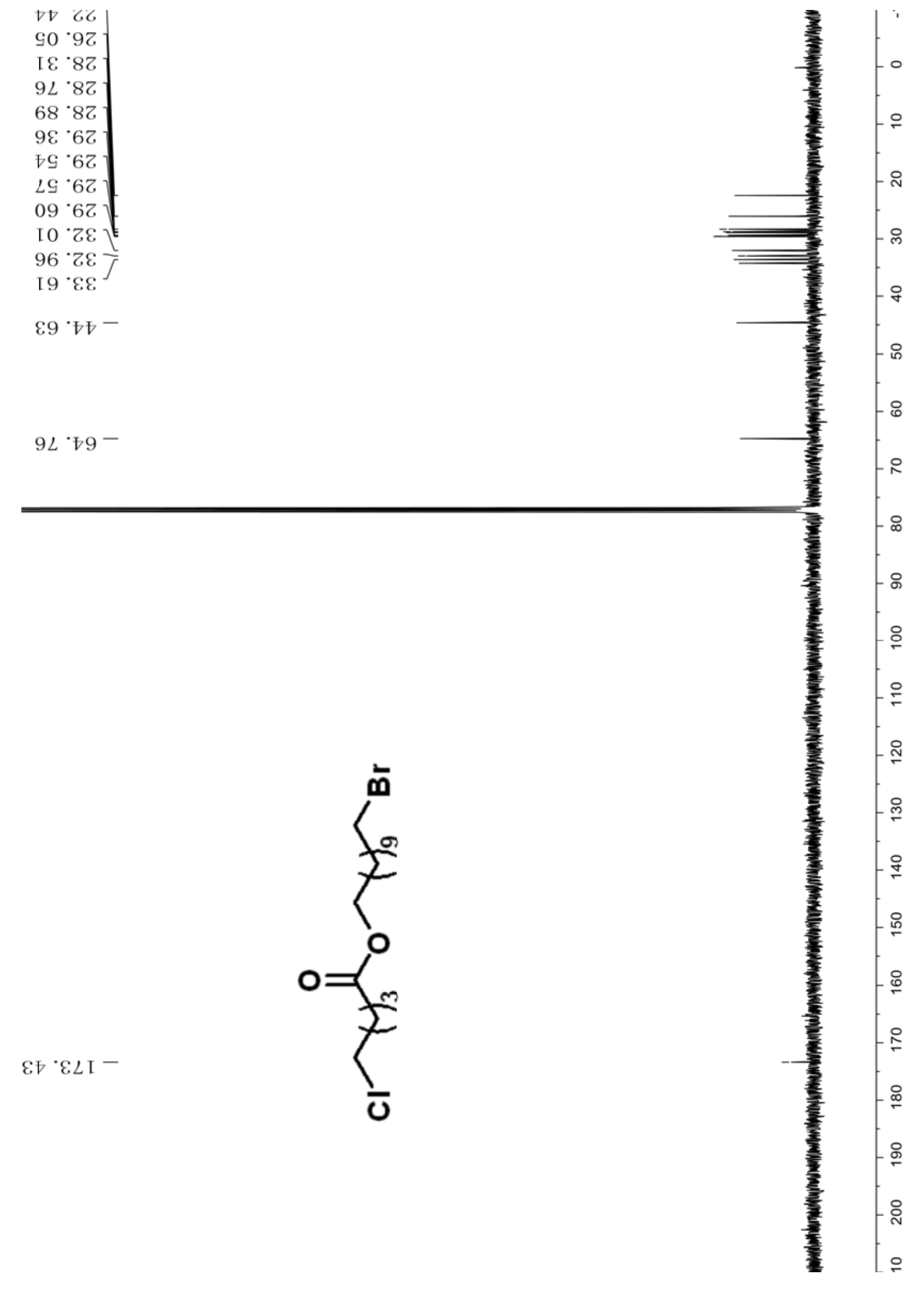

Supplementary Figure 79: ^{13}C NMR spectrum (101 MHz, $CDCl_3$, 23 °C) of **S29-1**

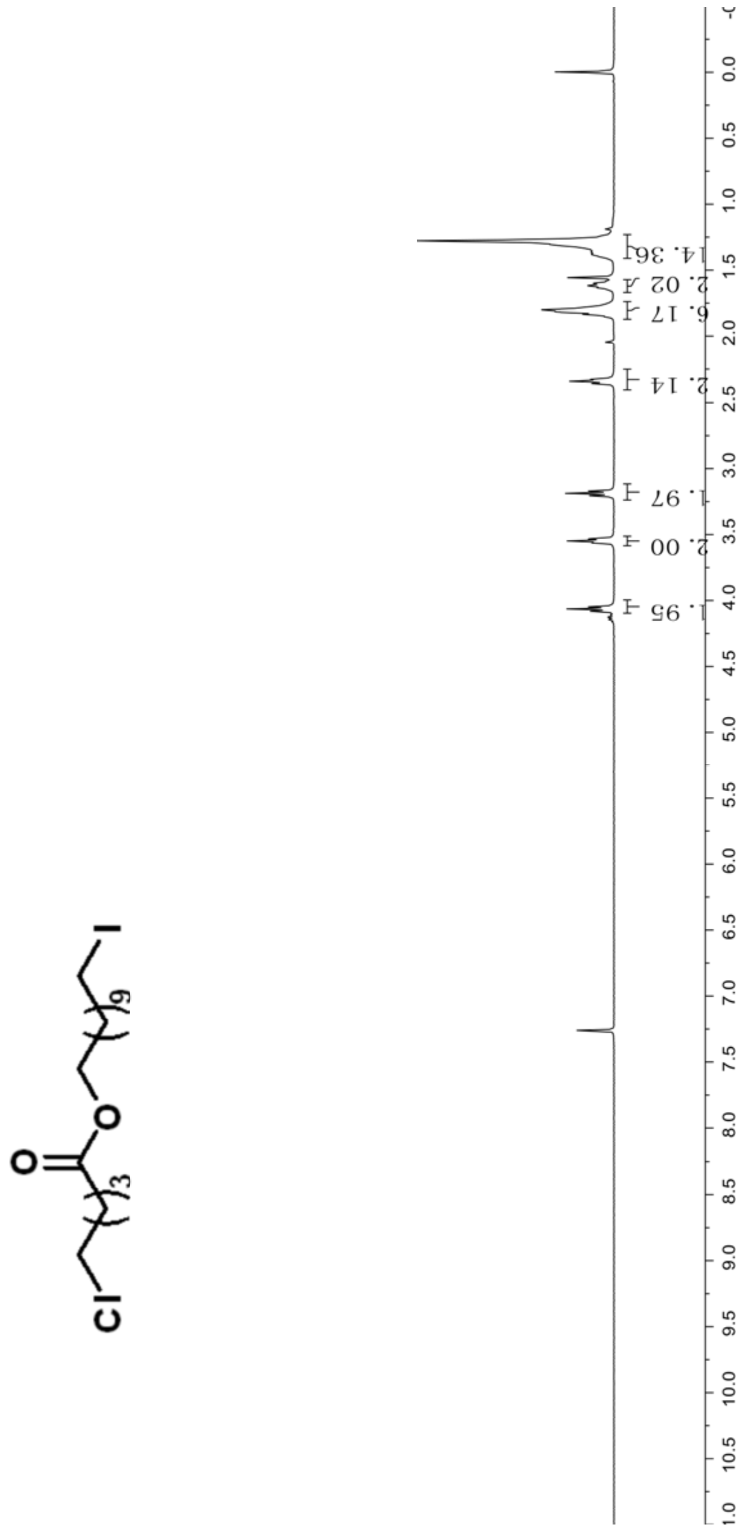

Supplementary Figure 80: ^{1}H NMR spectrum (400 MHz, $CDCl_3$, 23 °C) of **S29**

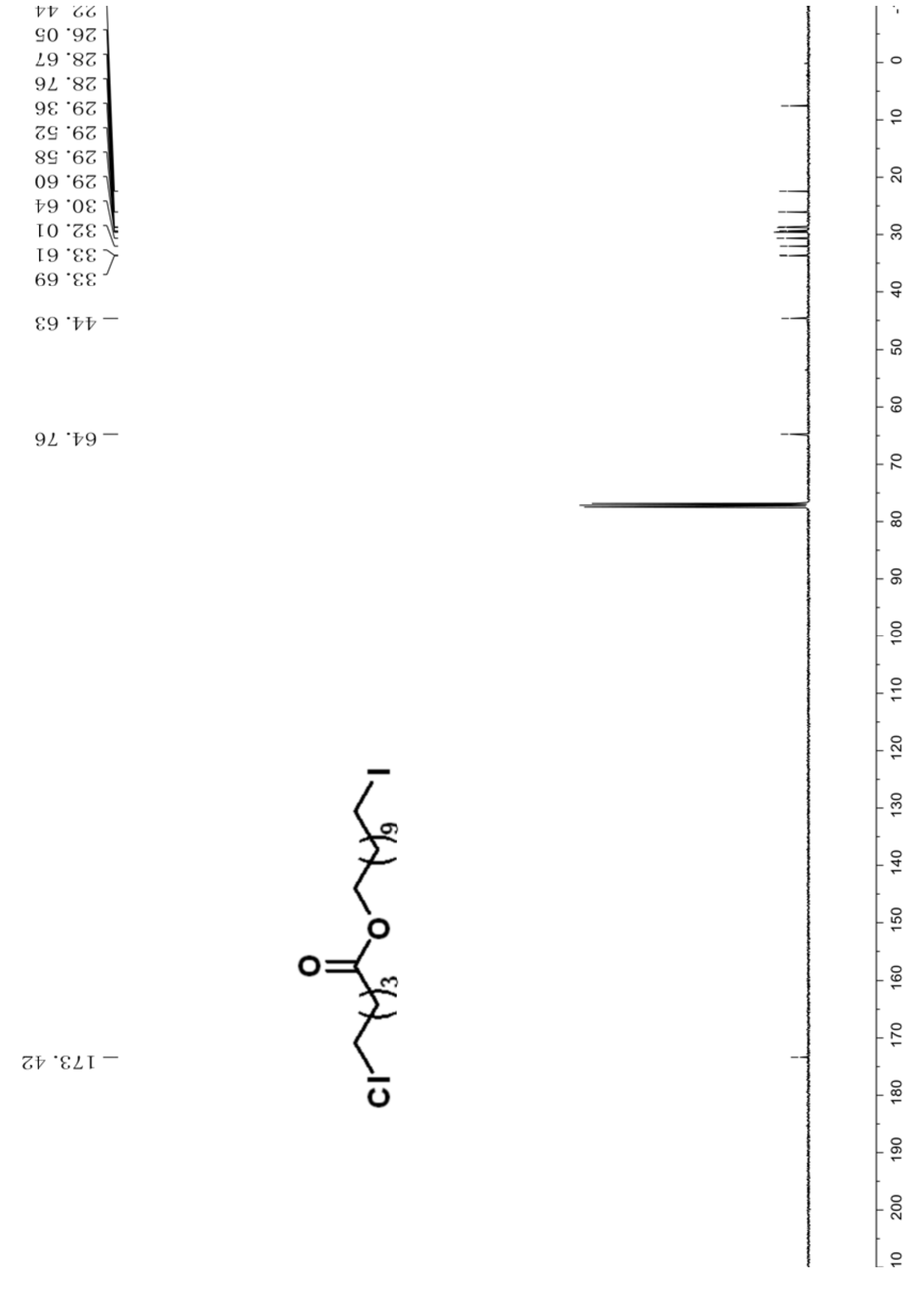

Supplementary Figure 81: ^{13}C NMR spectrum (101 MHz, $CDCl_3$, 23 °C) of **S29**

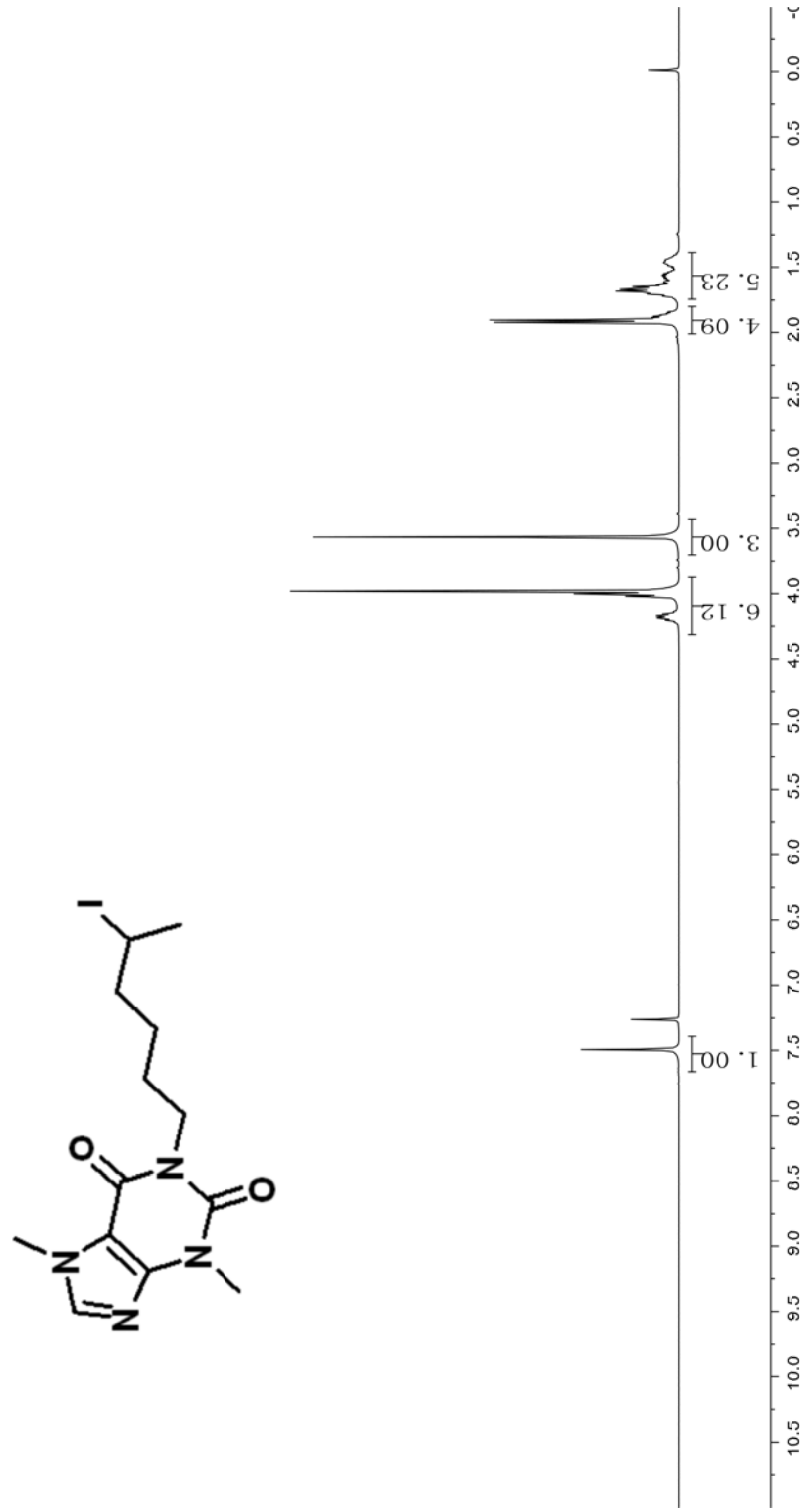

Supplementary Figure 82: ^{1}H NMR spectrum (400 MHz, $CDCl_3$, 23 °C) of **S34**

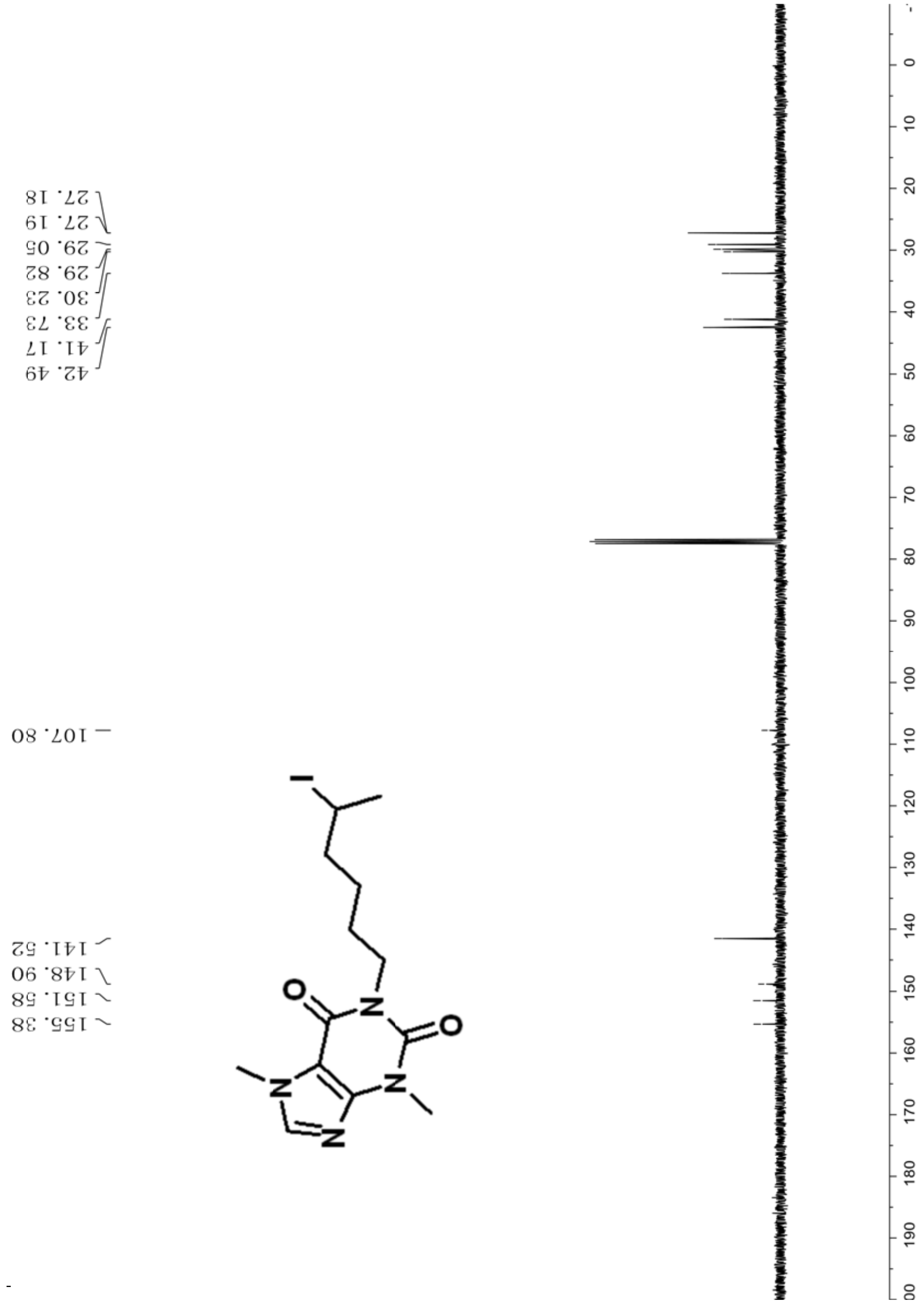

Supplementary Figure 83: ^{13}C NMR spectrum (101 MHz, $CDCl_3$, 23 °C) of **S34**

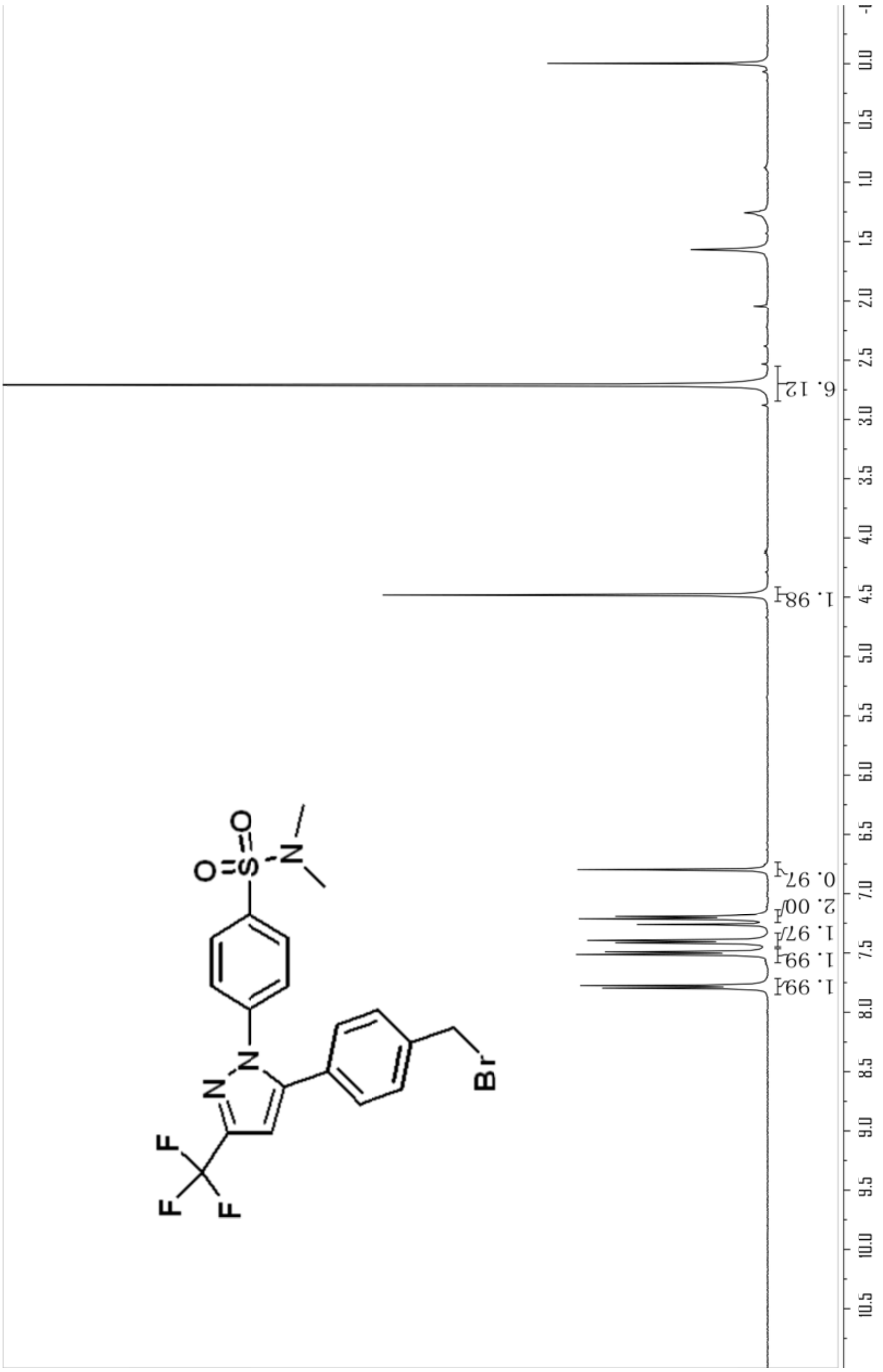

Supplementary Figure 84: ^{1}H NMR spectrum (400 MHz, $CDCl_3$, 23 °C) of **S40**

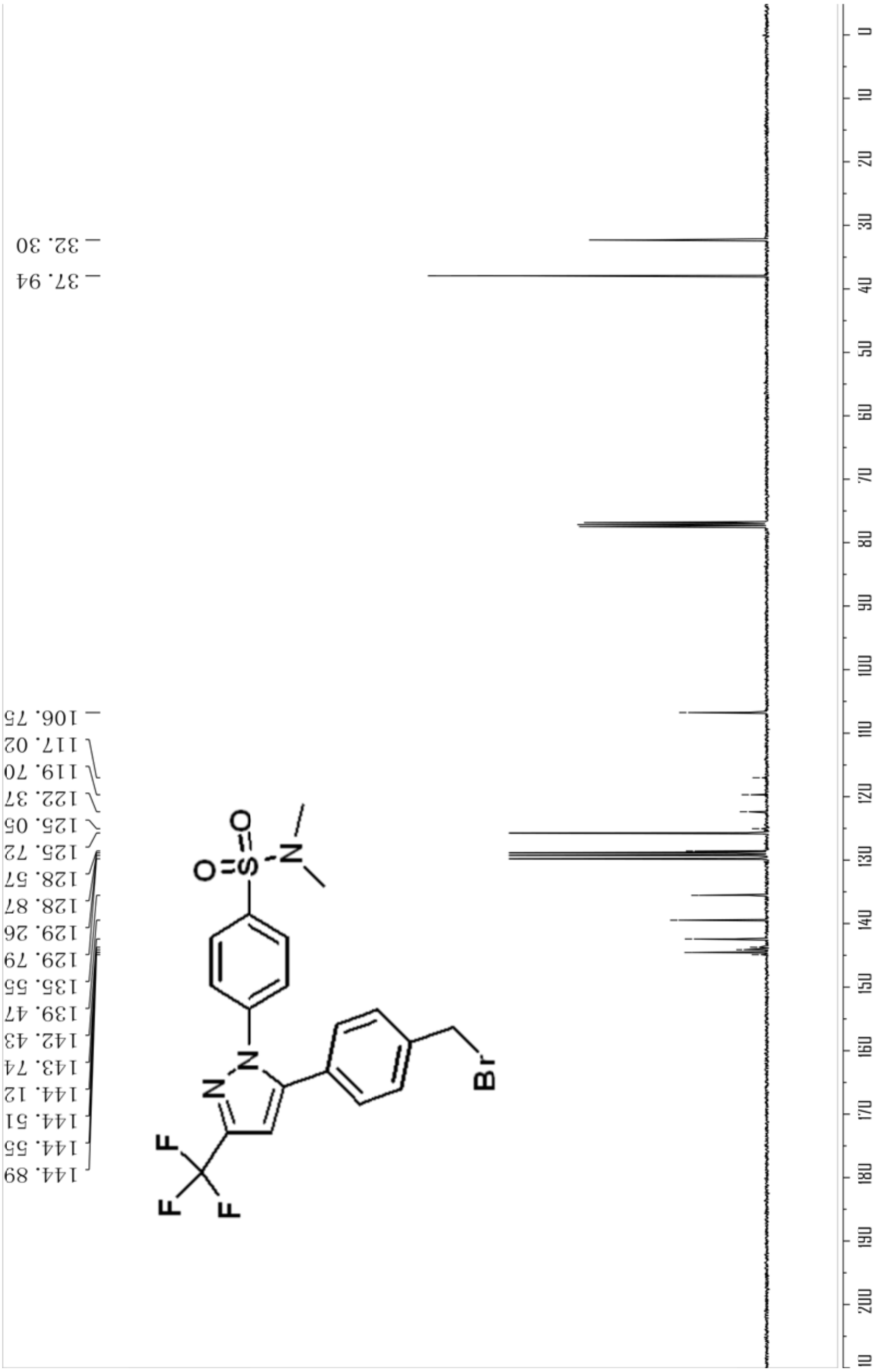

Supplementary Figure 85: ^{13}C NMR spectrum (101 MHz, $CDCl_3$, 23 °C) of **S40**

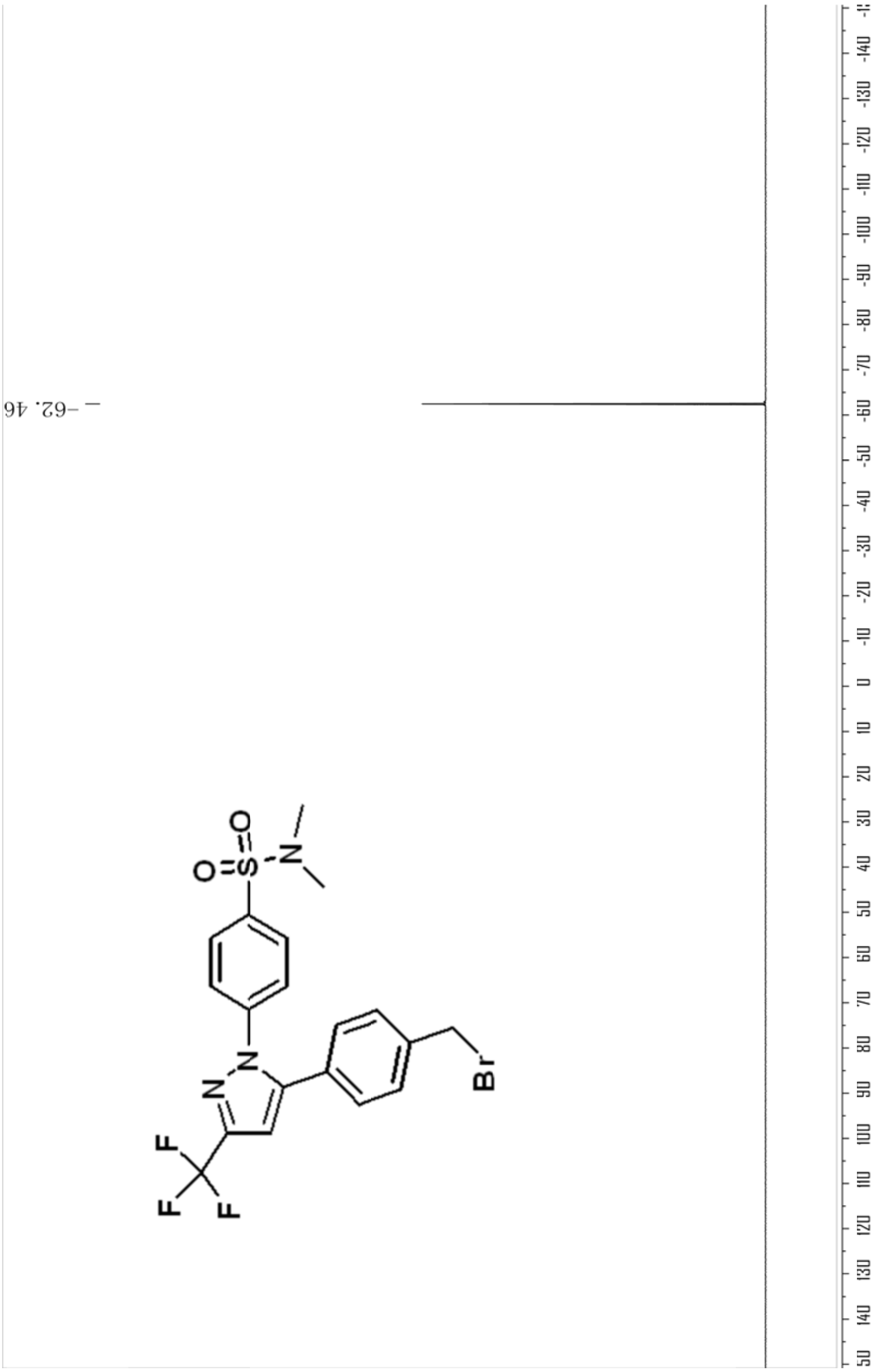

Supplementary Figure 86: ^{19}F NMR spectrum (376 MHz, $CDCl_3$, 23 °C) of **S40**

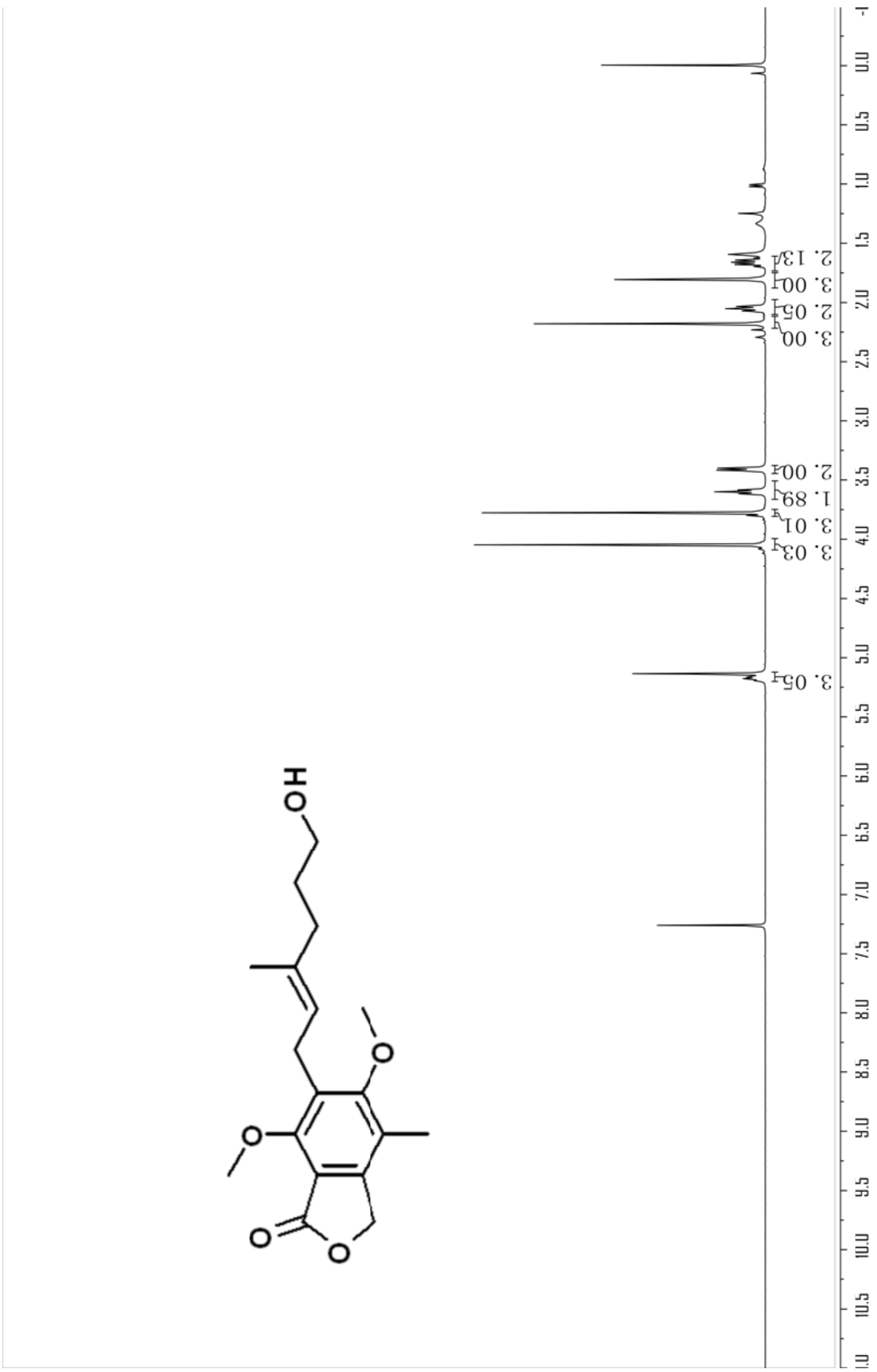

Supplementary Figure 87: ^{1}H NMR spectrum (400 MHz, $CDCl_3$, 23 °C) of **S41-1**

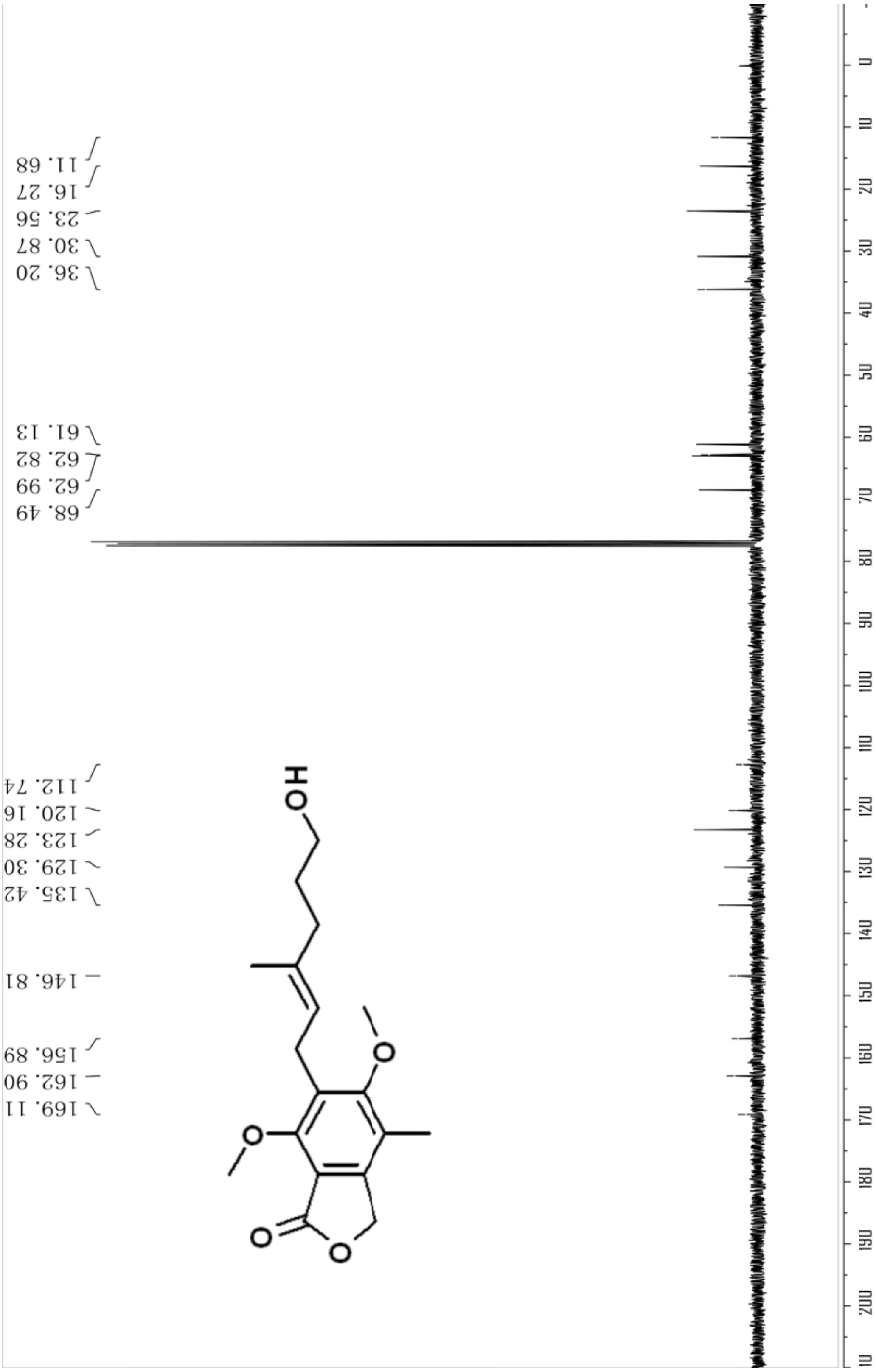

Supplementary Figure 88: ^{13}C NMR spectrum (101 MHz, $CDCl_3$, 23 °C) of **S41-1**

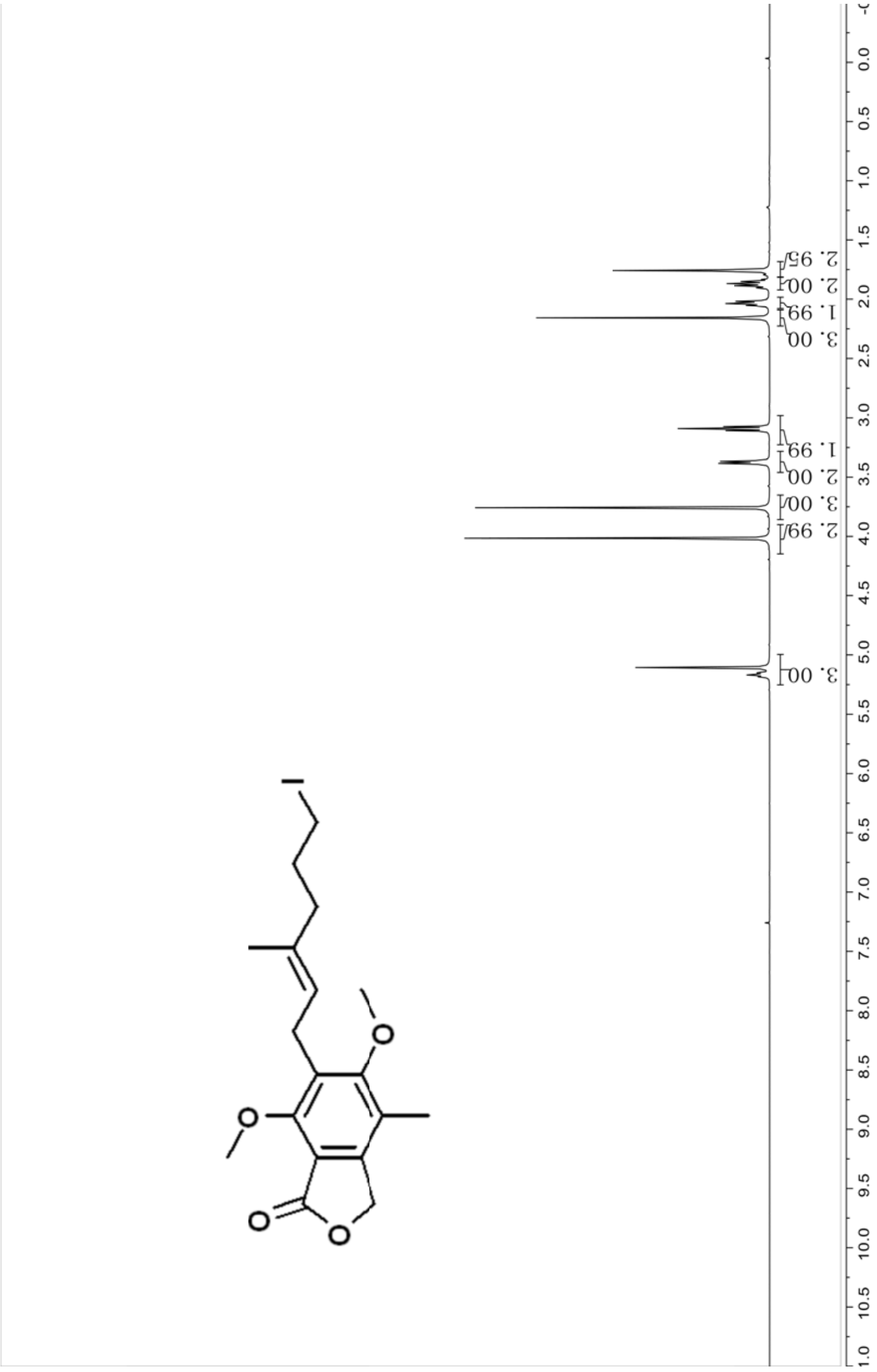

Supplementary Figure 89: ^{1}H NMR spectrum (400 MHz, $CDCl_3$, 23 °C) of **S41**

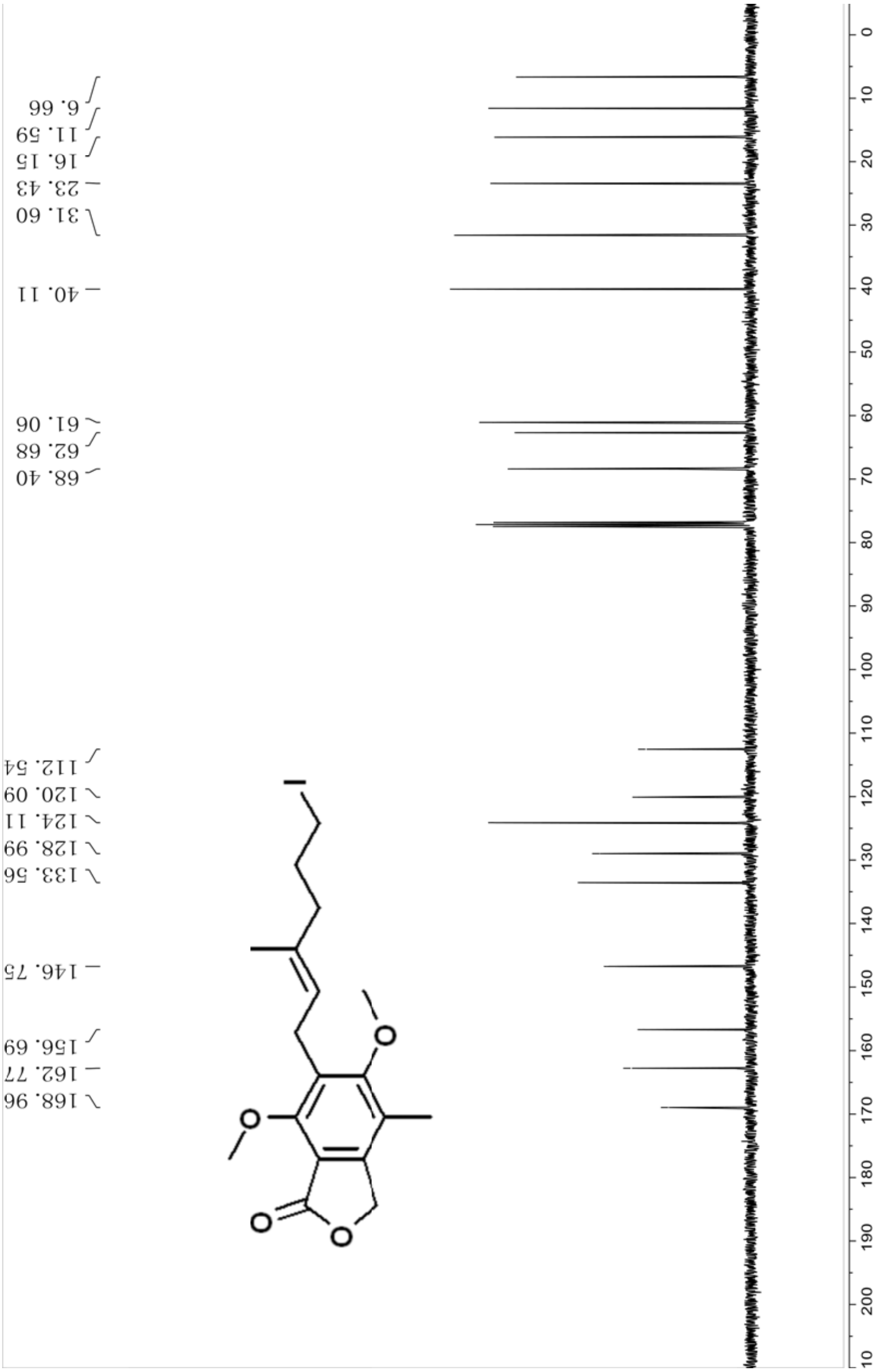

Supplementary Figure 90: ^{13}C NMR spectrum (101 MHz, $CDCl_3$, 23 °C) of **S41**

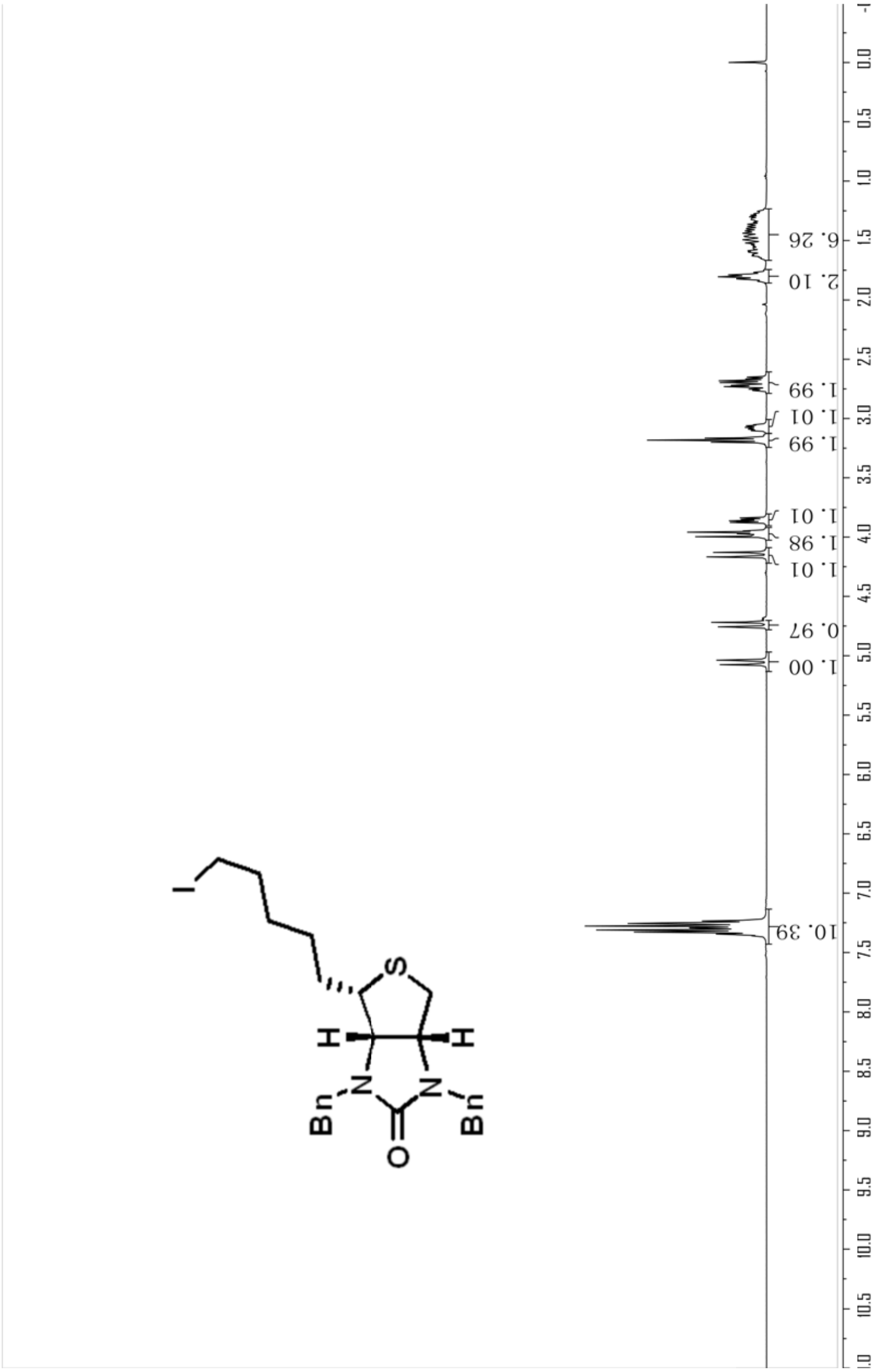

Supplementary Figure 91: ^{1}H NMR spectrum (400 MHz, $CDCl_3$, 23 °C) of **S42**

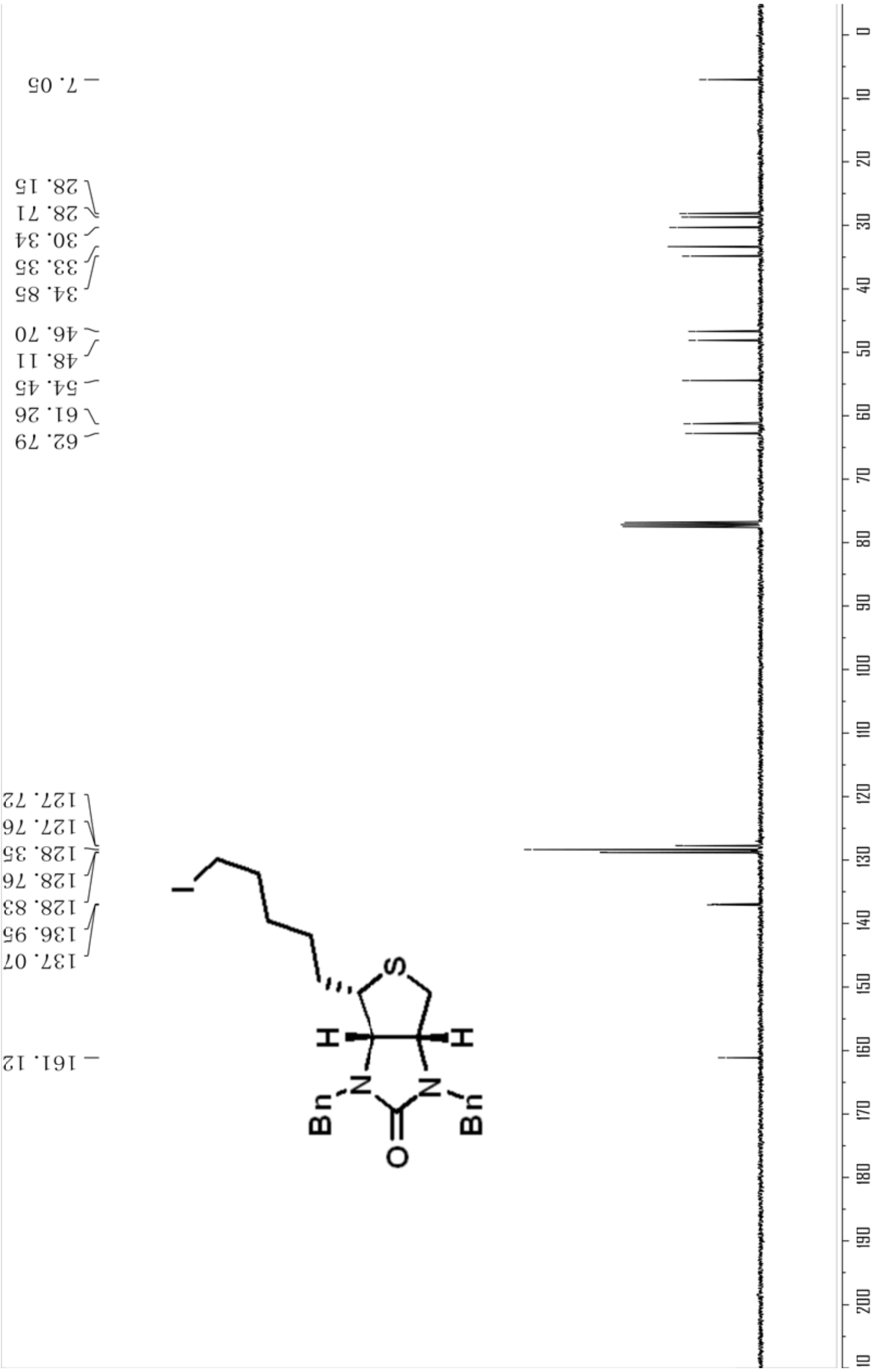

Supplementary Figure 92: ^{13}C NMR spectrum (101 MHz, $CDCl_3$, 23 °C) of **S42**

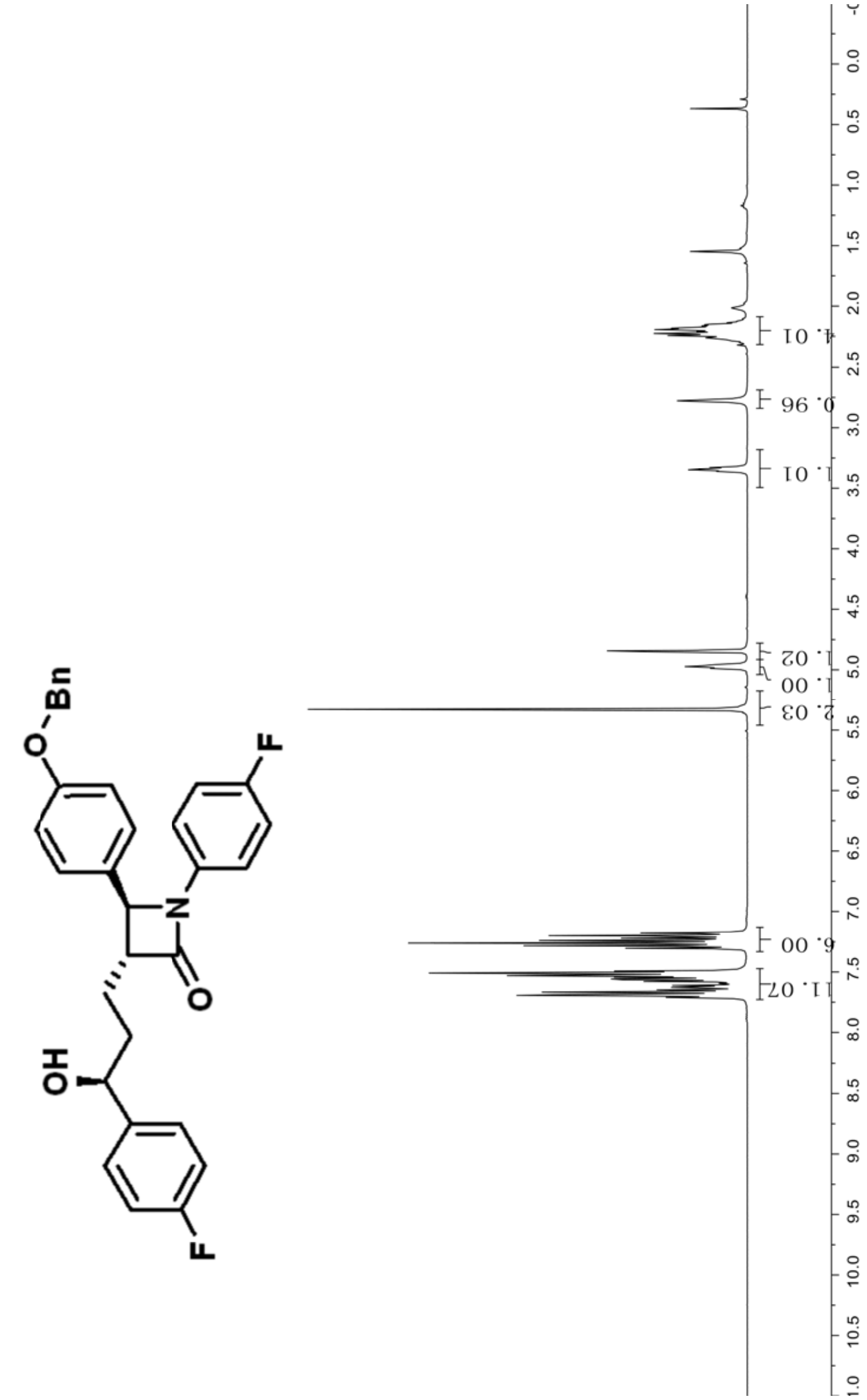

Supplementary Figure 93: ^{1}H NMR spectrum (400 MHz, $CDCl_3$, 23 °C) of **S45-1**

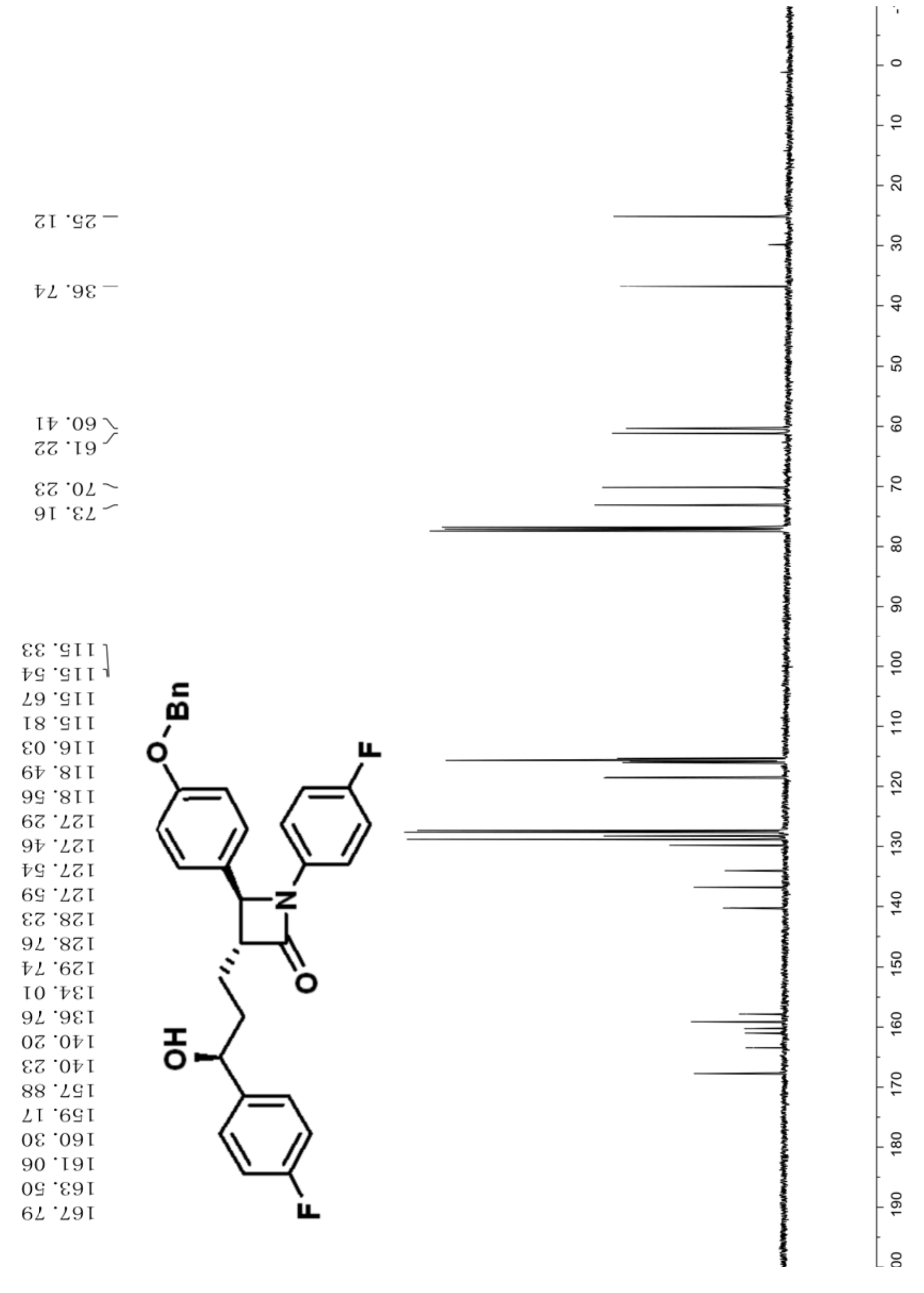

Supplementary Figure 94: ^{13}C NMR spectrum (101 MHz, $CDCl_3$, 23 °C) of **S45-1**

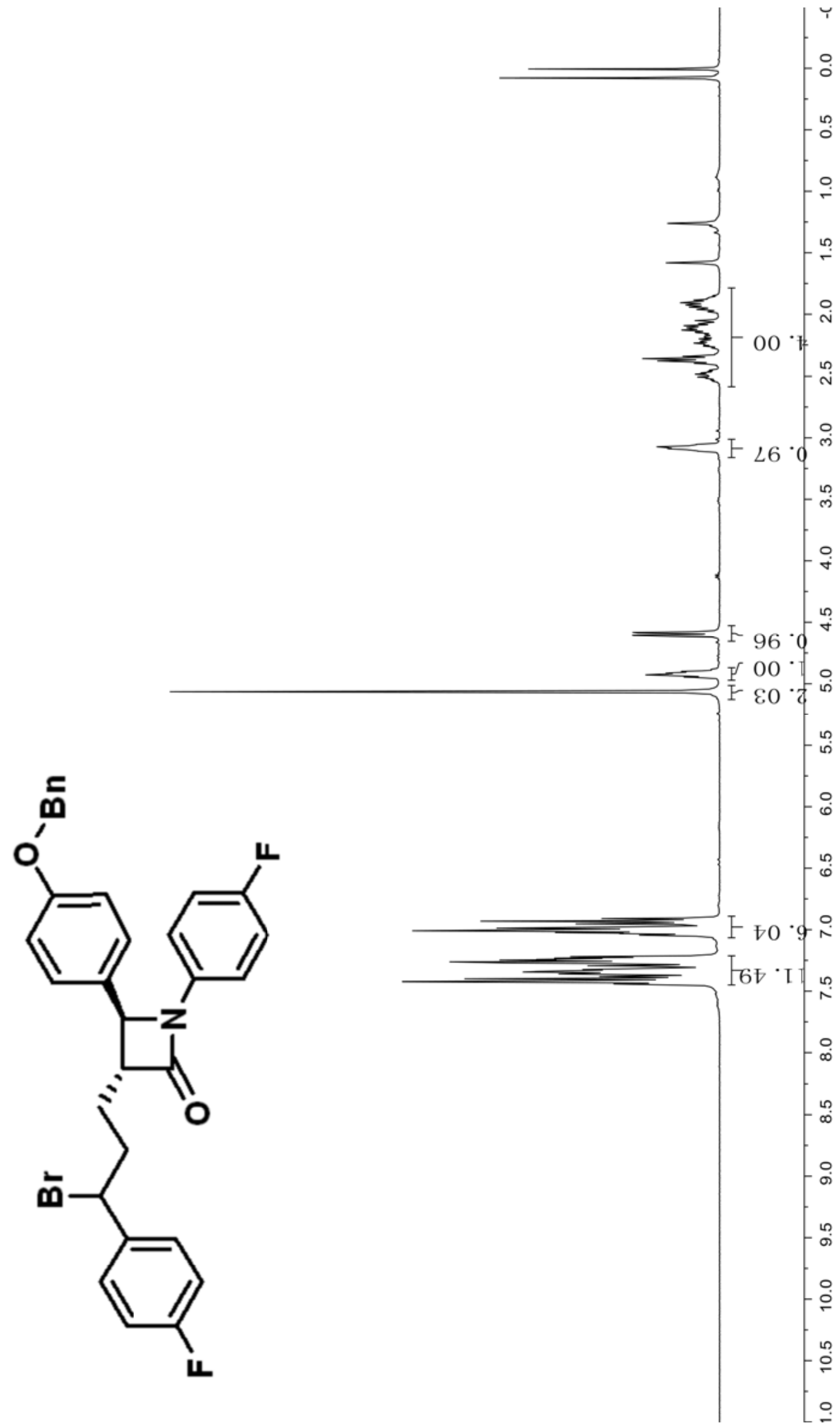

Supplementary Figure 94: ^{1}H NMR spectrum (400 MHz, $CDCl_3$, 23 °C) of **S45**

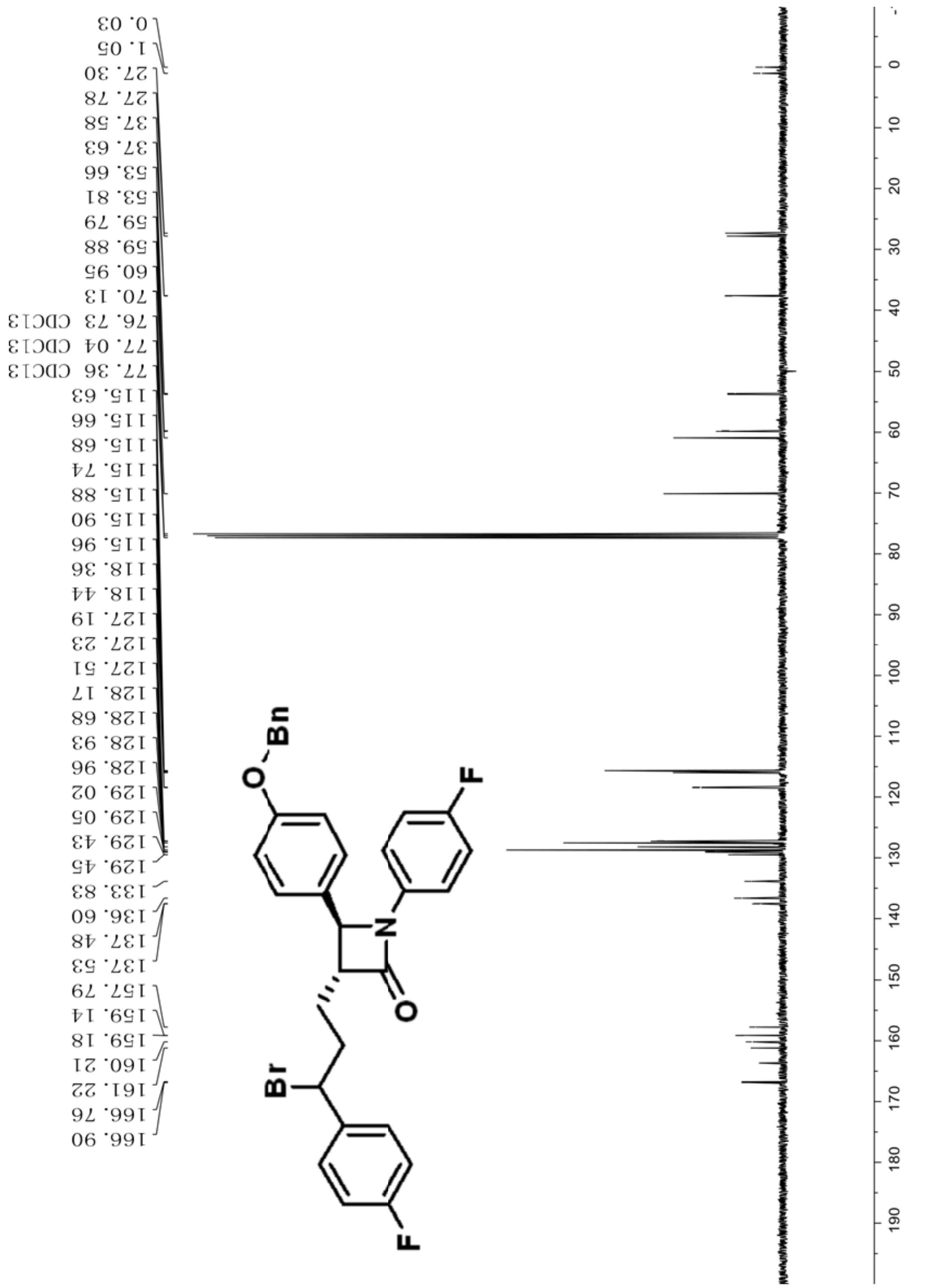

Supplementary Figure 95: ^{13}C NMR spectrum (101 MHz, $CDCl_3$, 23 °C) of **S45**

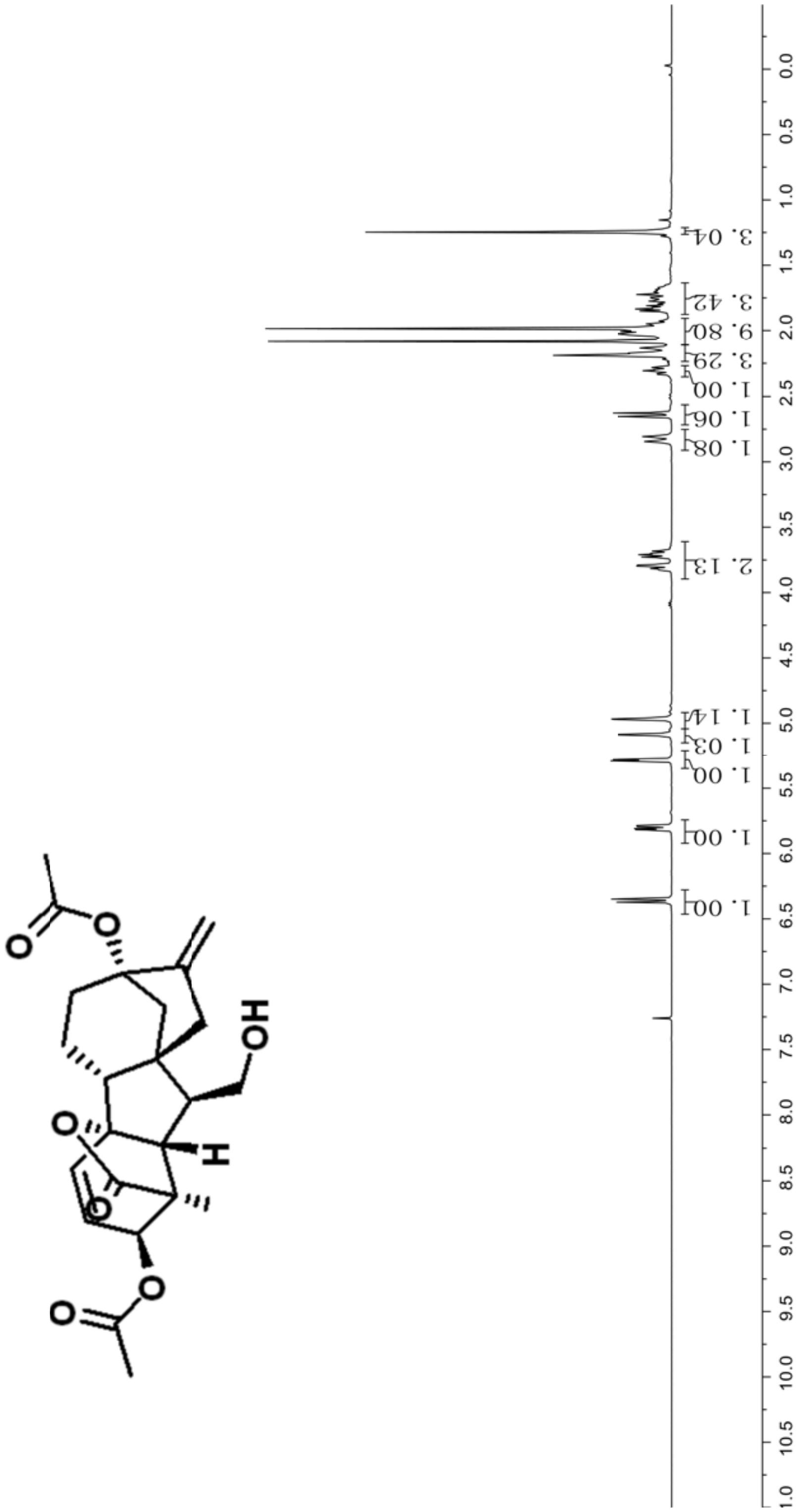

Supplementary Figure 96: ^{1}H NMR spectrum (400 MHz, $CDCl_3$, 23 °C) of **S46-1**

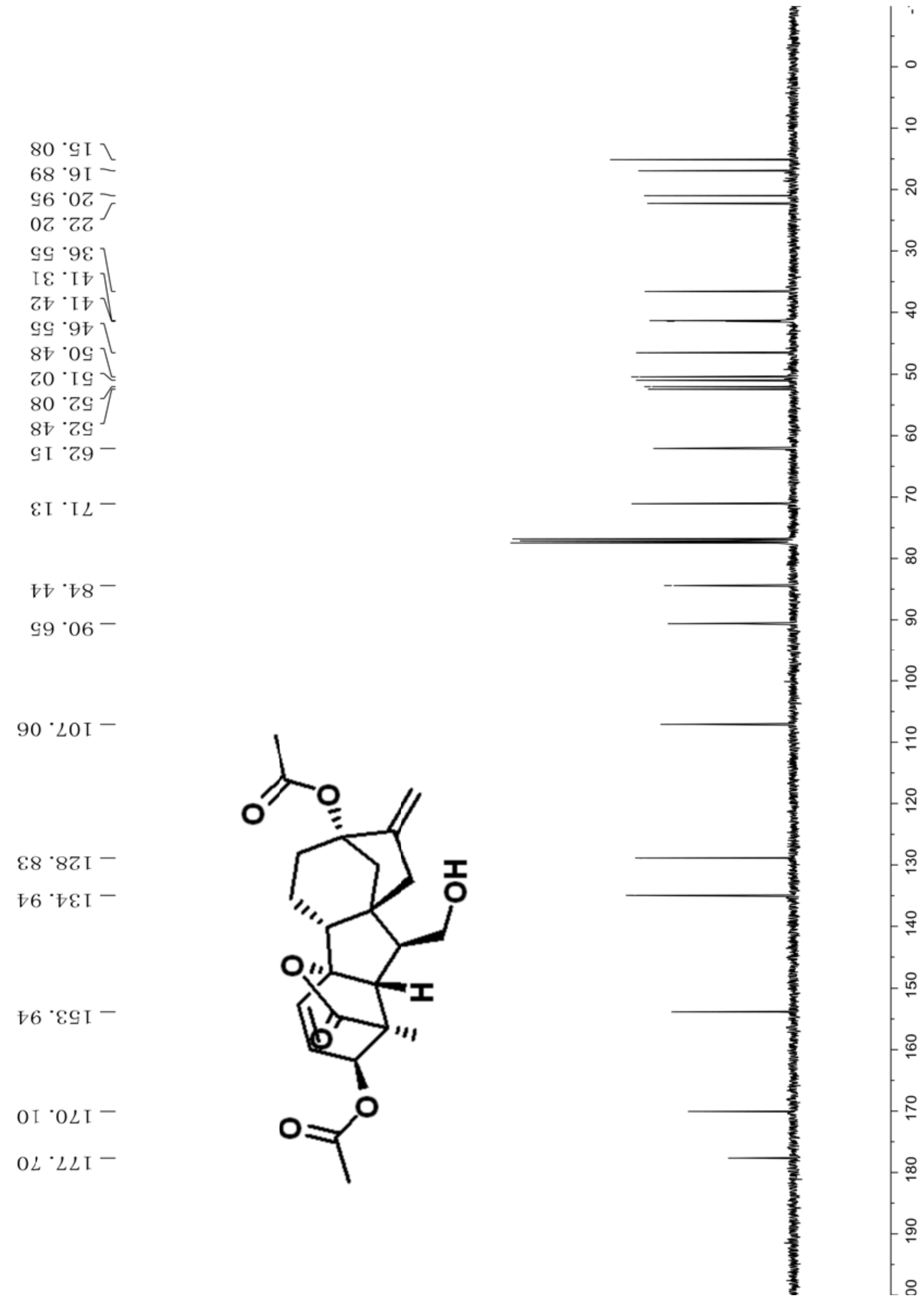

Supplementary Figure 97: ^{13}C NMR spectrum (101 MHz, $CDCl_3$, 23 °C) of **S46-1**

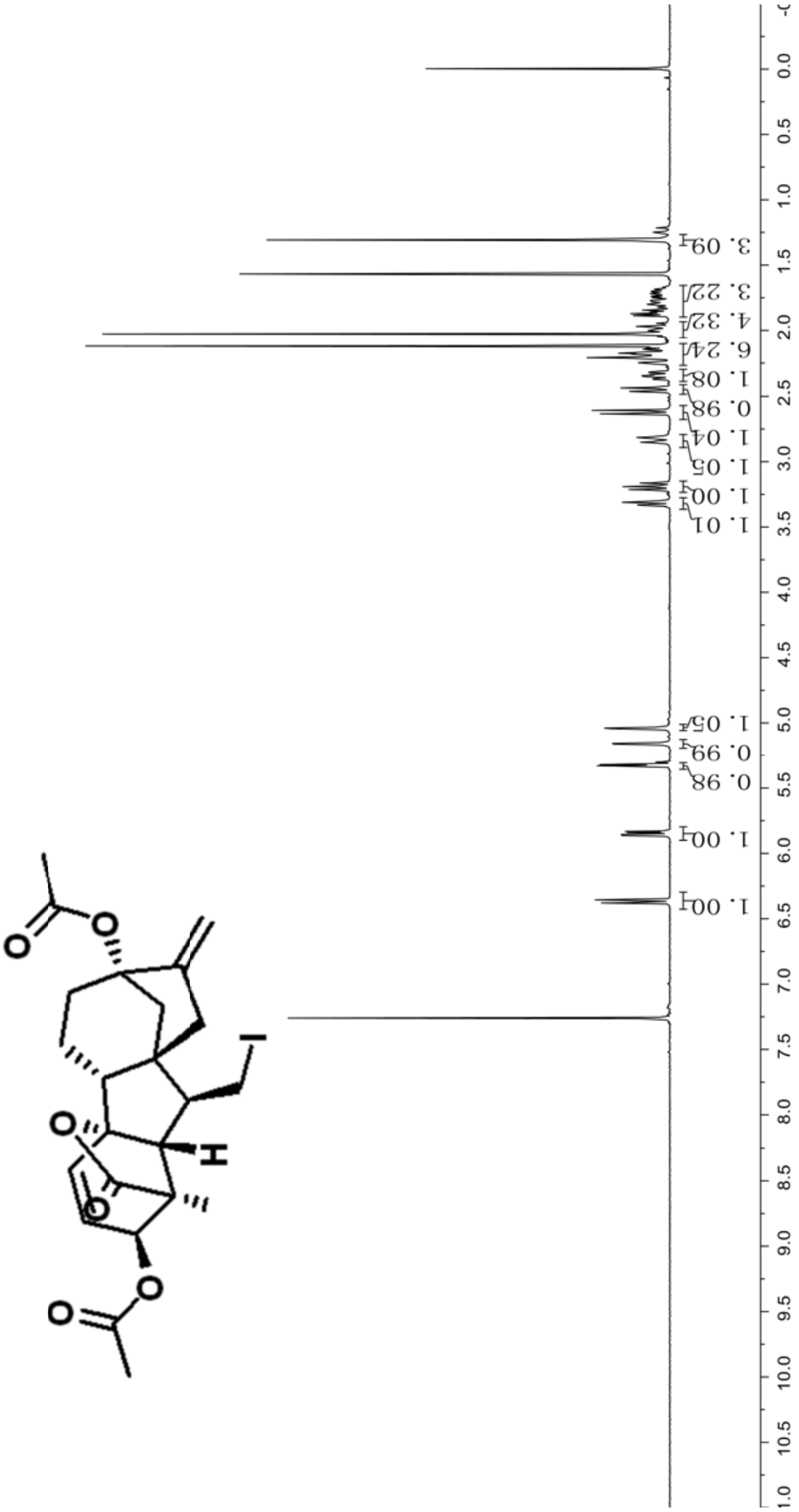

Supplementary Figure 98: ^{1}H NMR spectrum (400 MHz, $CDCl_3$, 23 °C) of **S46**

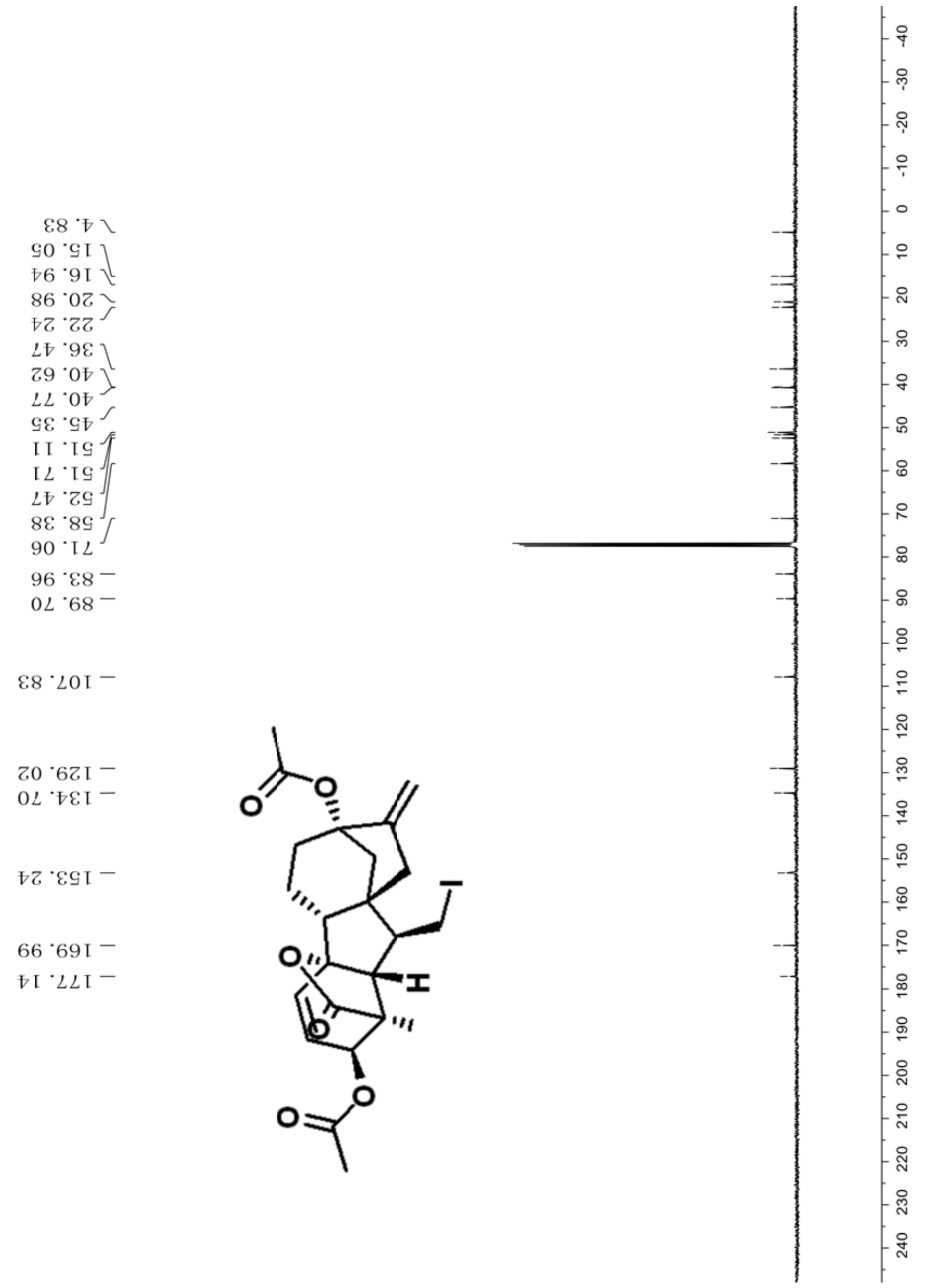

Supplementary Figure 99: ^{13}C NMR spectrum (101 MHz, CDCl3, 23 °C) of **S46**

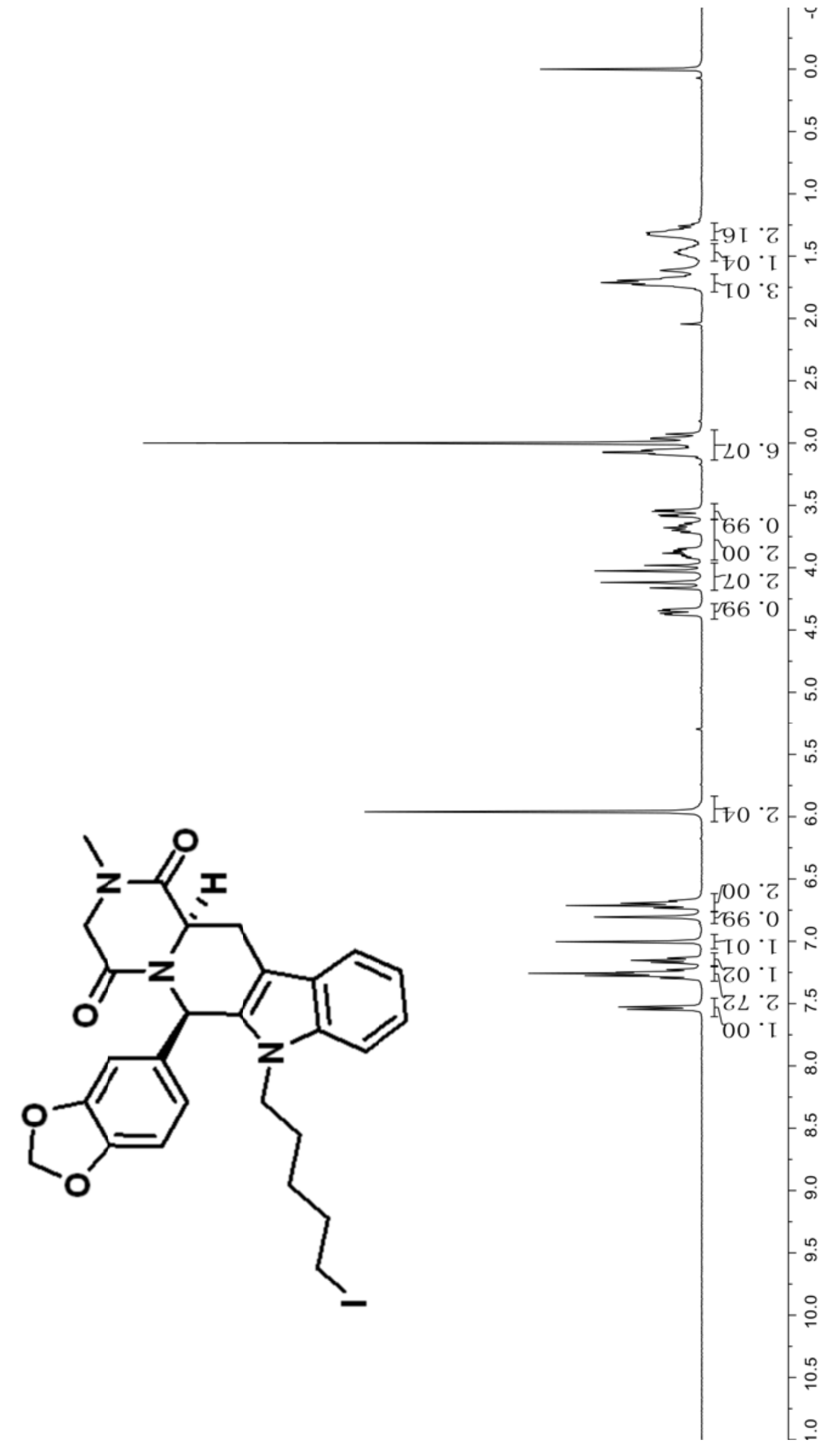

Supplementary Figure 100: ^{1}H NMR spectrum (400 MHz, $CDCl_3$, 23 °C) of **S47**

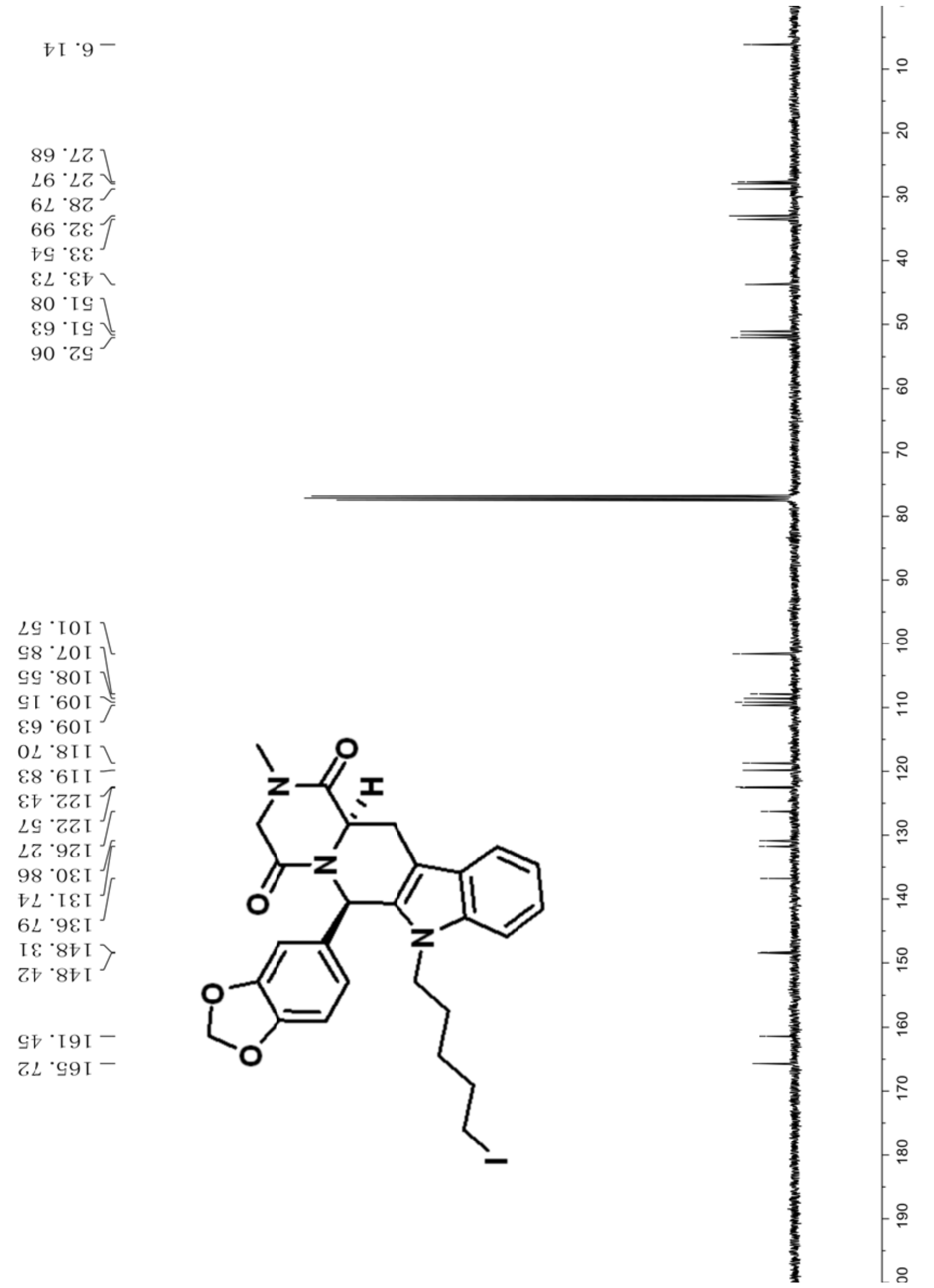

Supplementary Figure 101: ^{13}C NMR spectrum (101 MHz, $CDCl_3$, 23 °C) of **S47**

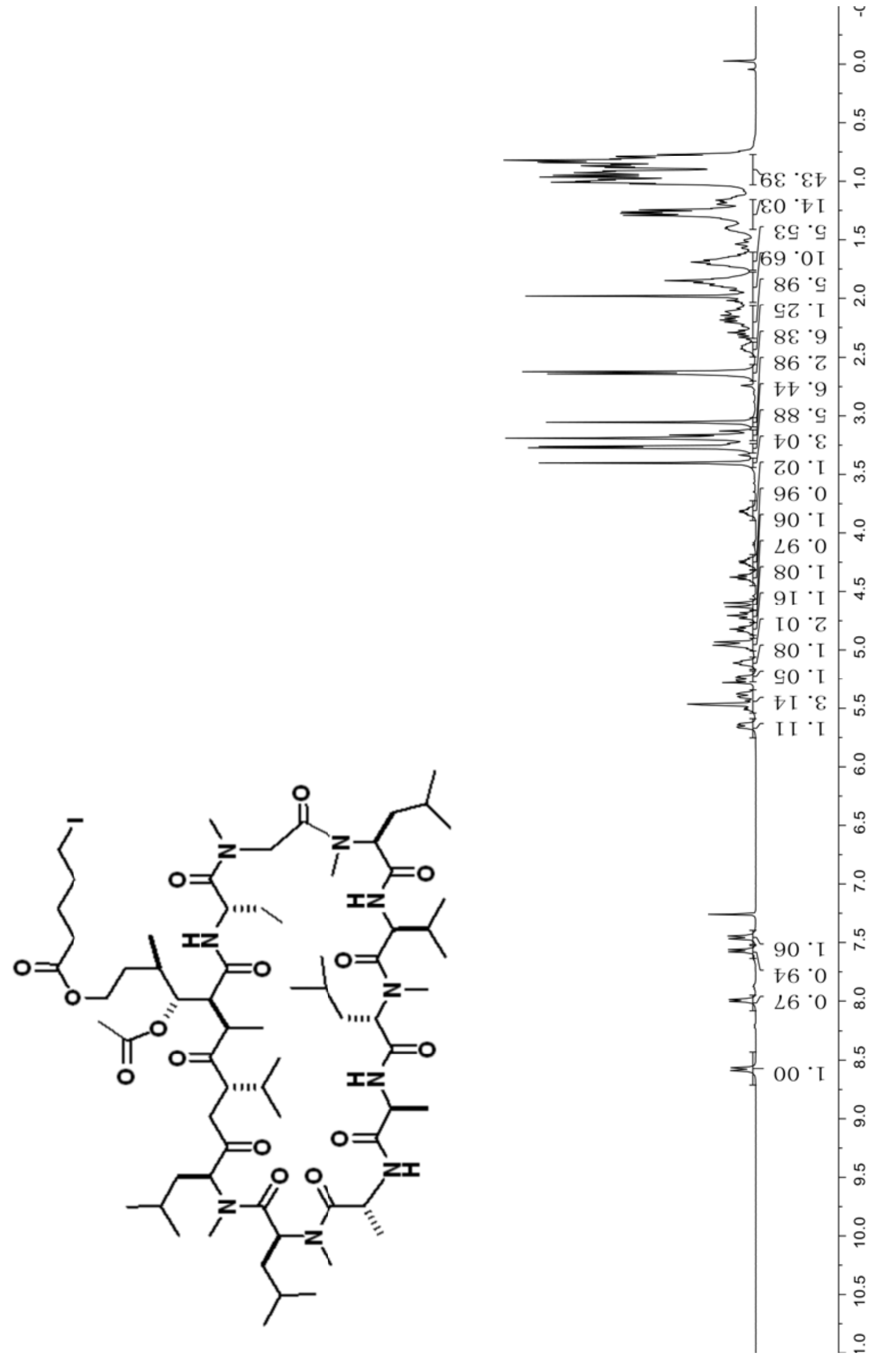

Supplementary Figure 102: ^{1}H NMR spectrum (400 MHz, $CDCl_3$, 23 °C) of **S48**

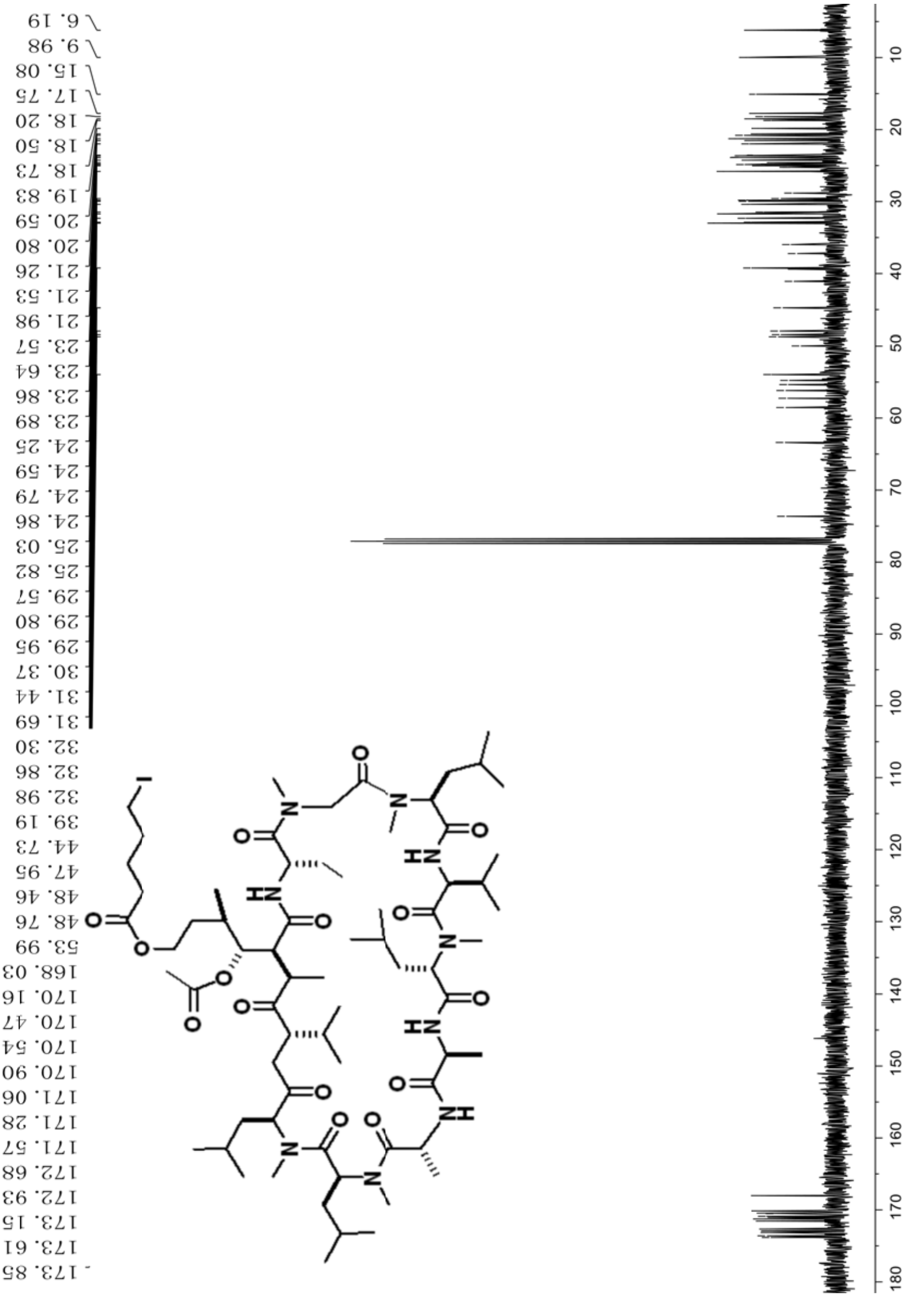

Supplementary Figure 103: ^{13}C NMR spectrum (101 MHz, $CDCl_3$, 23 °C) of **S48**

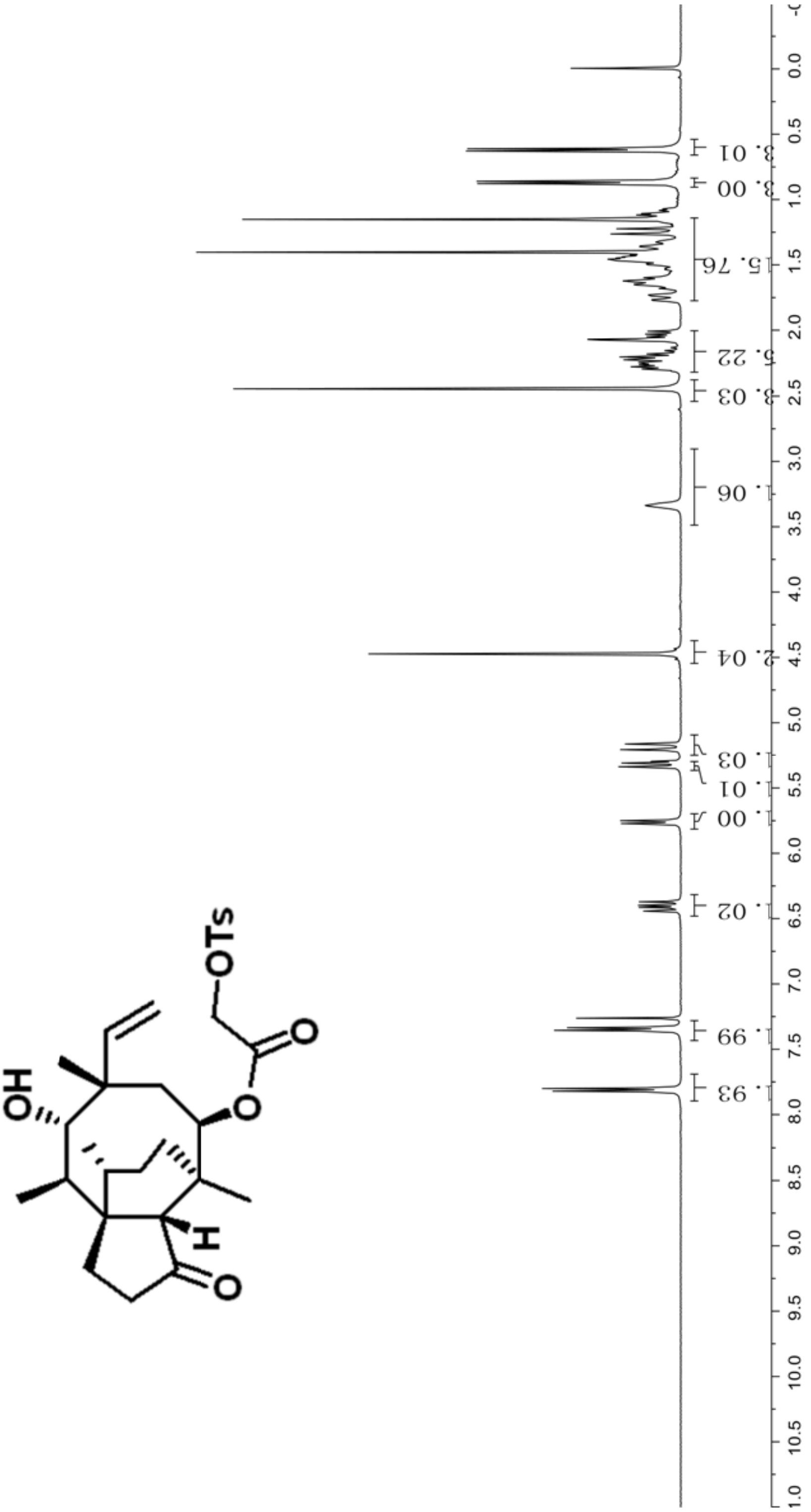

Supplementary Figure 104 ^{1}H NMR spectrum (400 MHz, $CDCl_3$, 23 °C) of **S49-1**

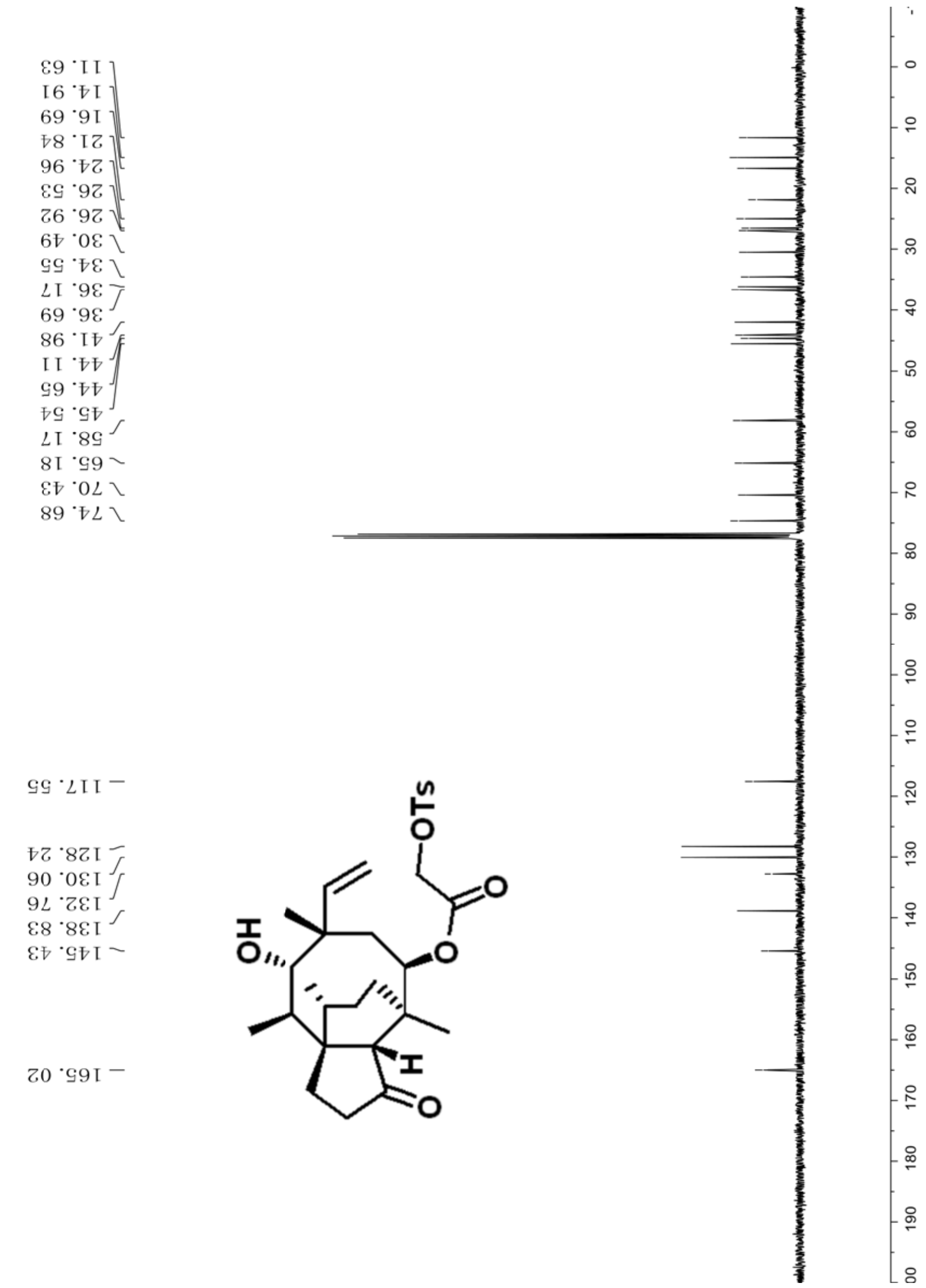

Supplementary Figure 105: ^{13}C NMR spectrum (101 MHz, $CDCl_3$, 23 °C) of **S49-1**

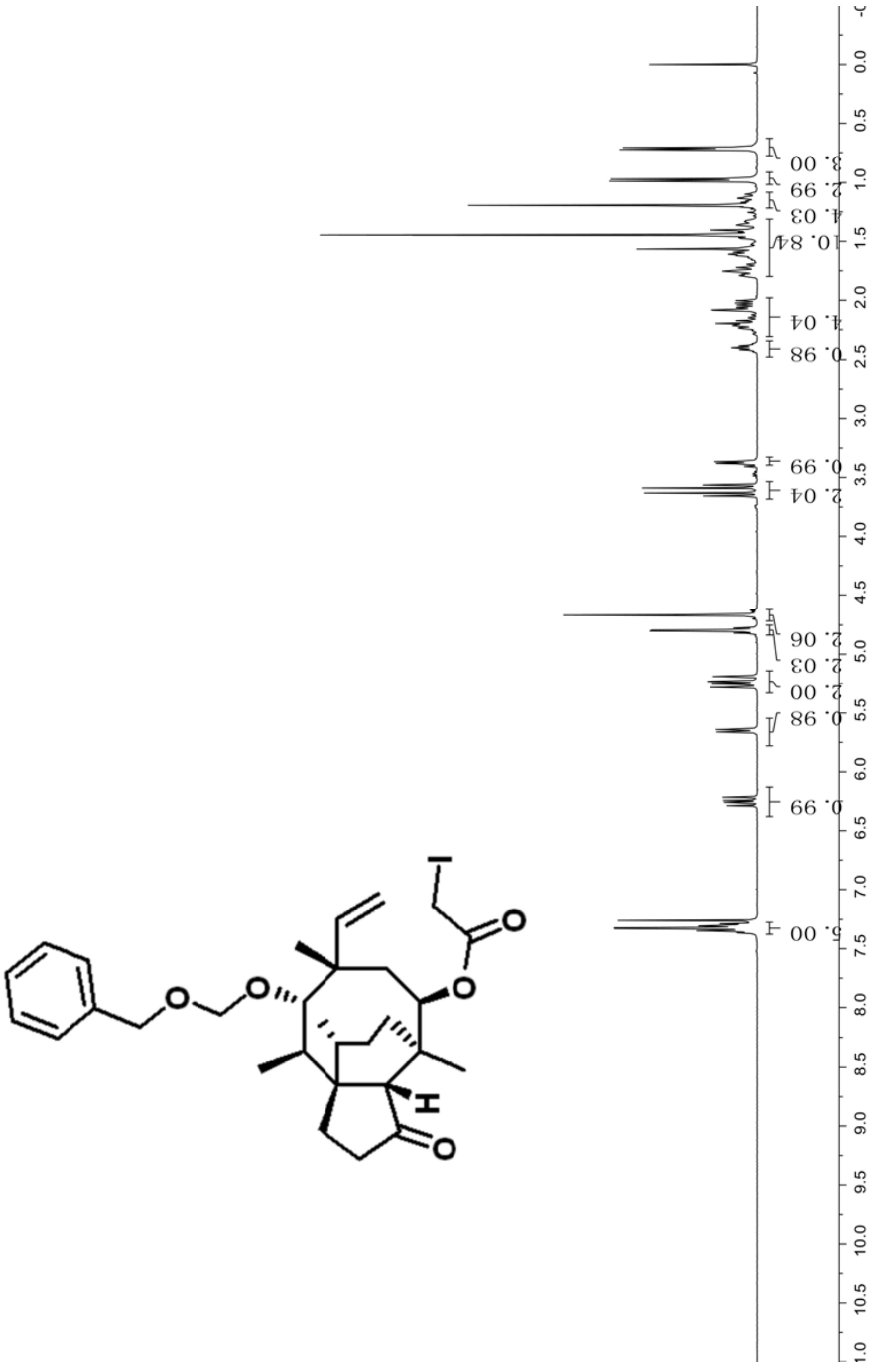

Supplementary Figure 106: ^{1}H NMR spectrum (400 MHz, $CDCl_3$, 23 °C) of **S49**

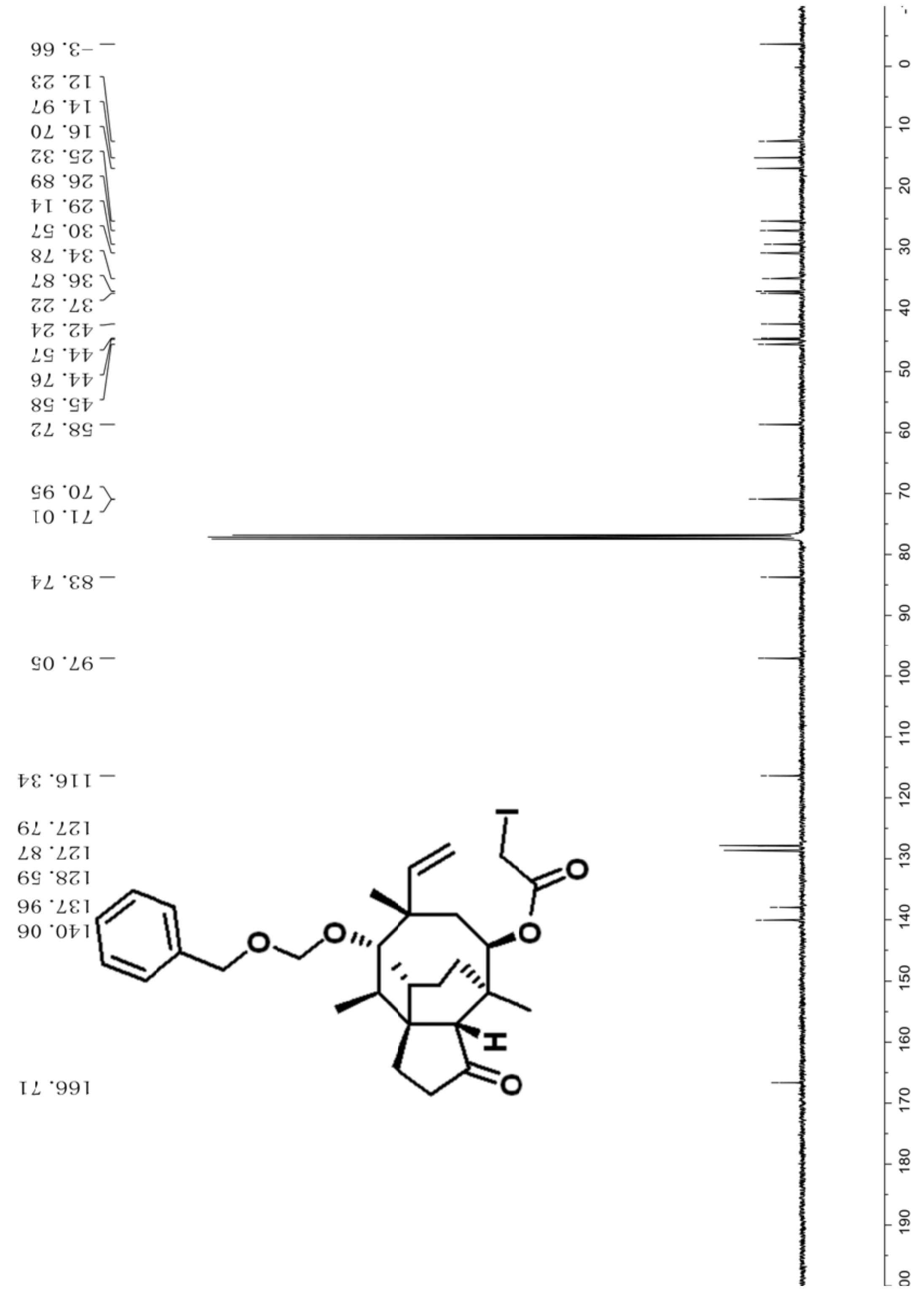

Supplementary Figure 107: ^{13}C NMR spectrum (101 MHz, $CDCl_3$, 23 °C) of **S49**

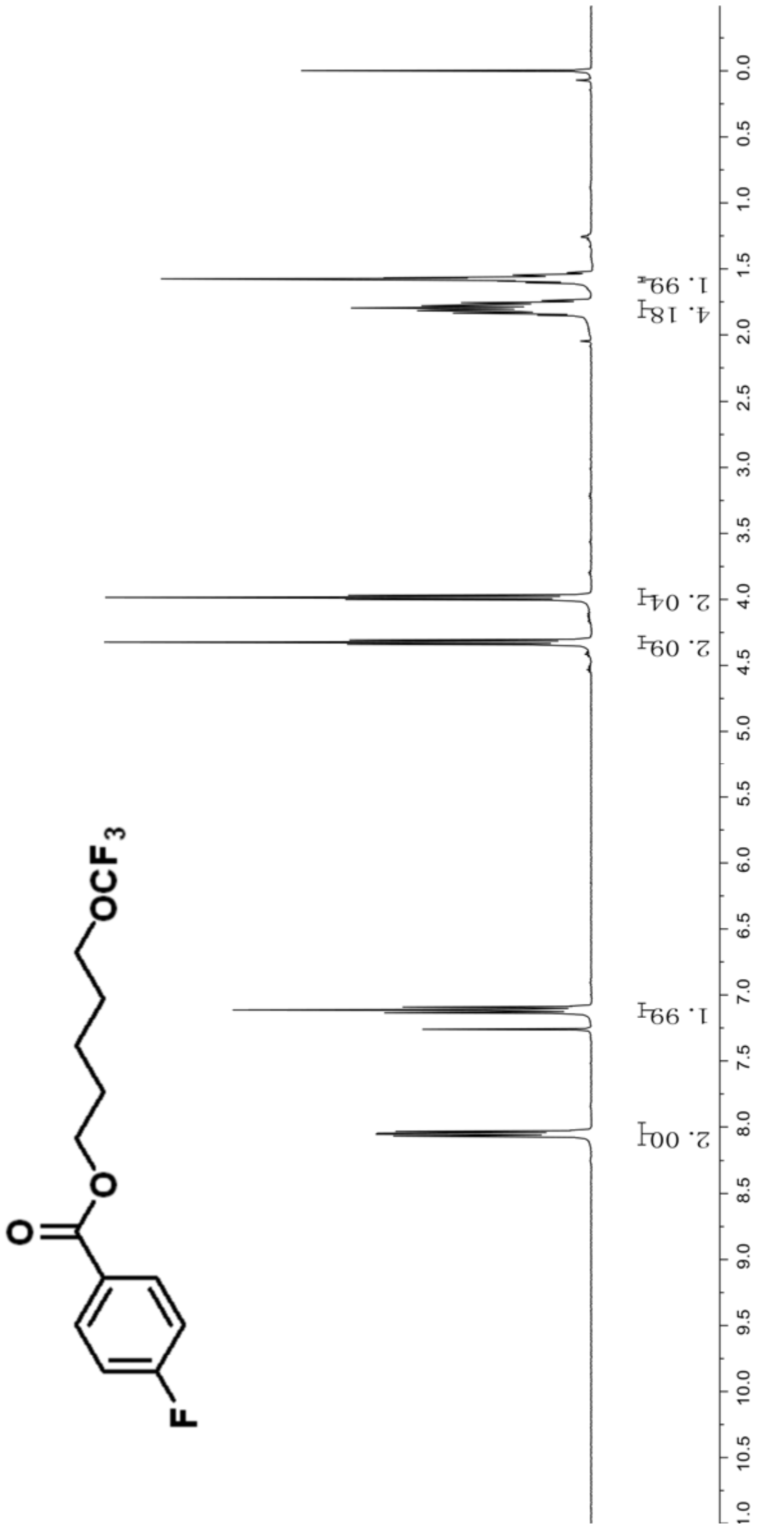

Supplementary Figure 108: ^{1}H NMR spectrum (400 MHz, $CDCl_3$, 23 °C) of **3**

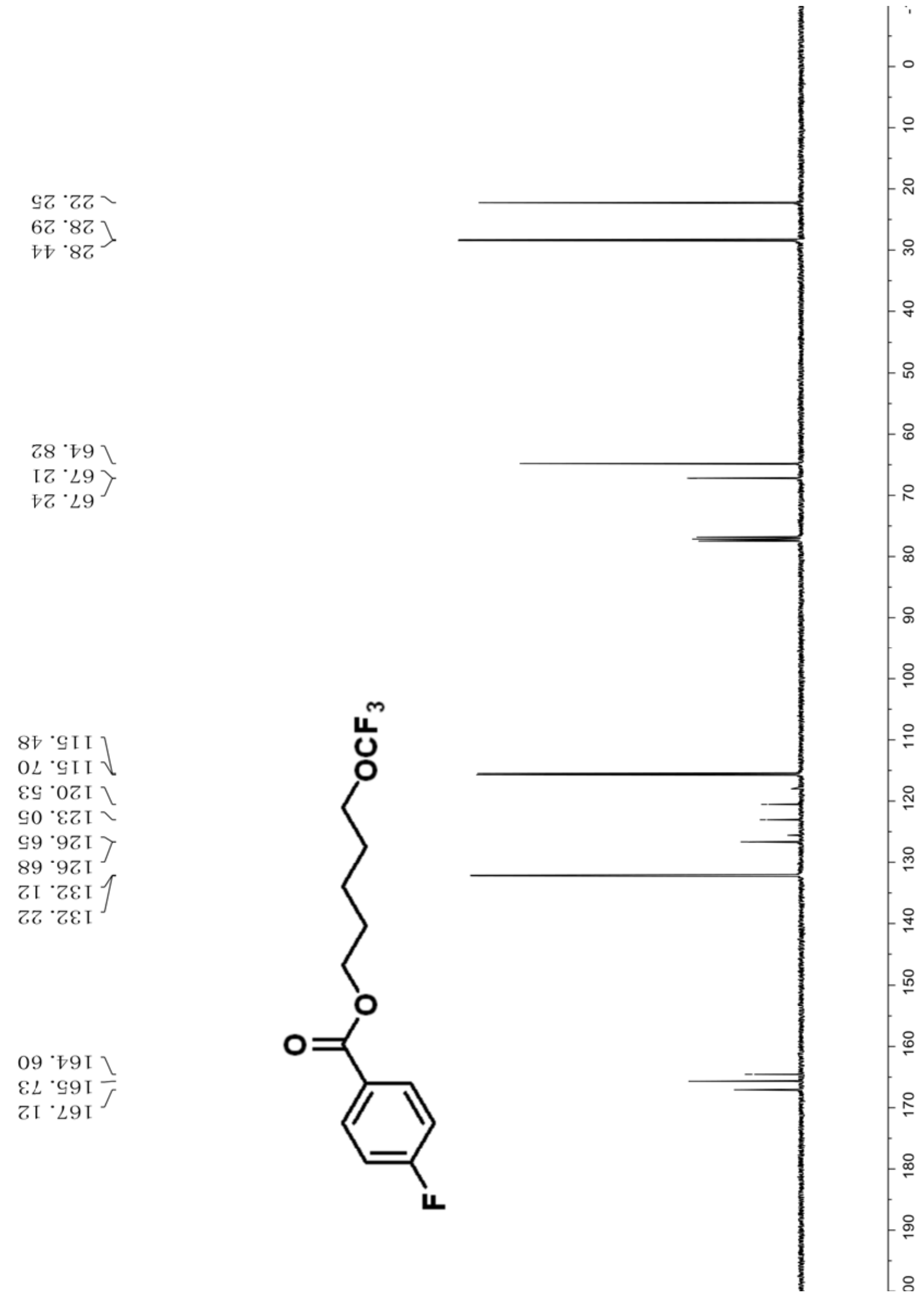

Supplementary Figure 109: ^{13}C NMR spectrum (101 MHz, $CDCl_3$, 23 °C) of **3**

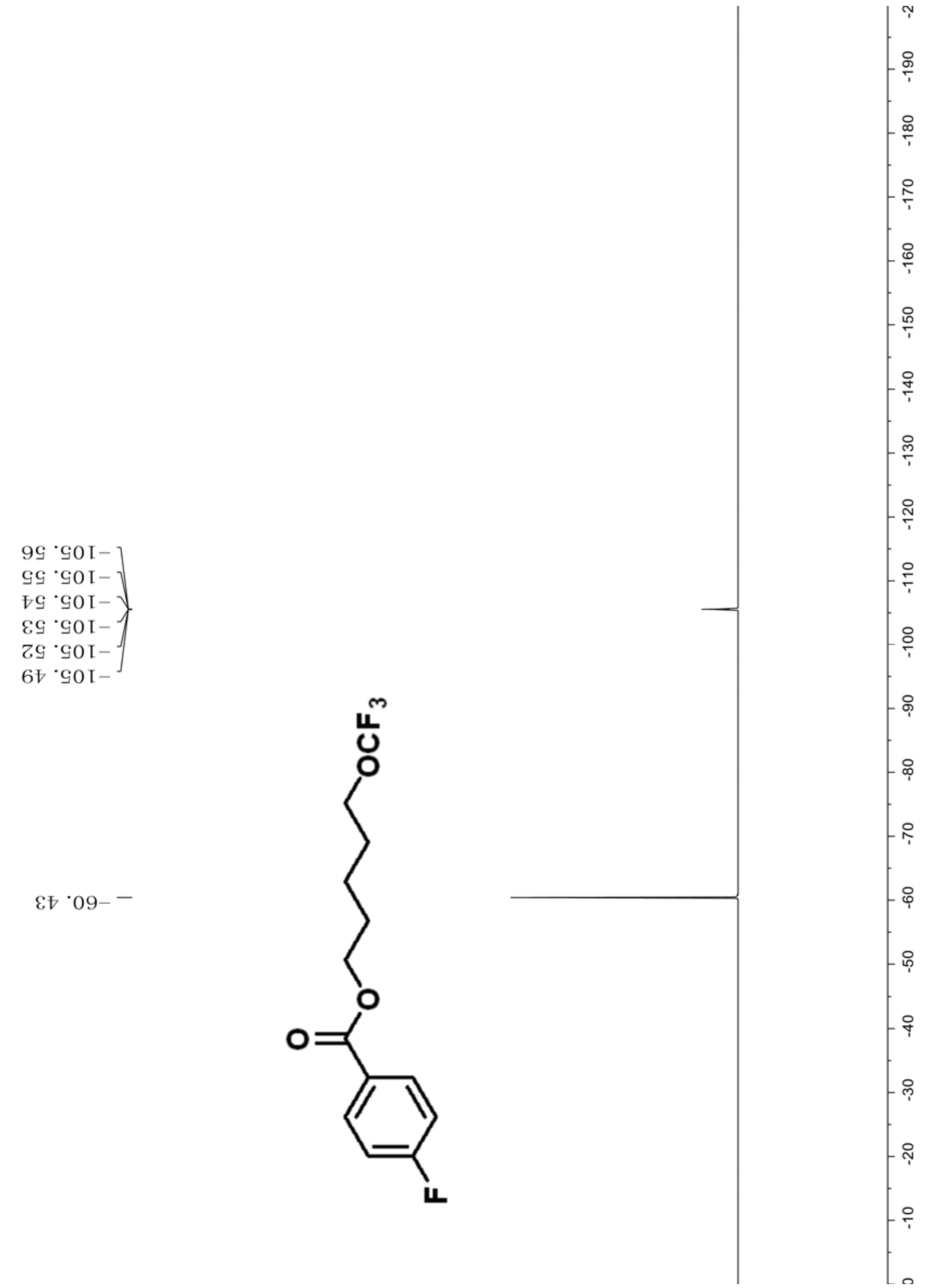

Supplementary Figure 110: ^{19}F NMR spectrum (376 MHz, $CDCl_3$, 23 °C) of **3**

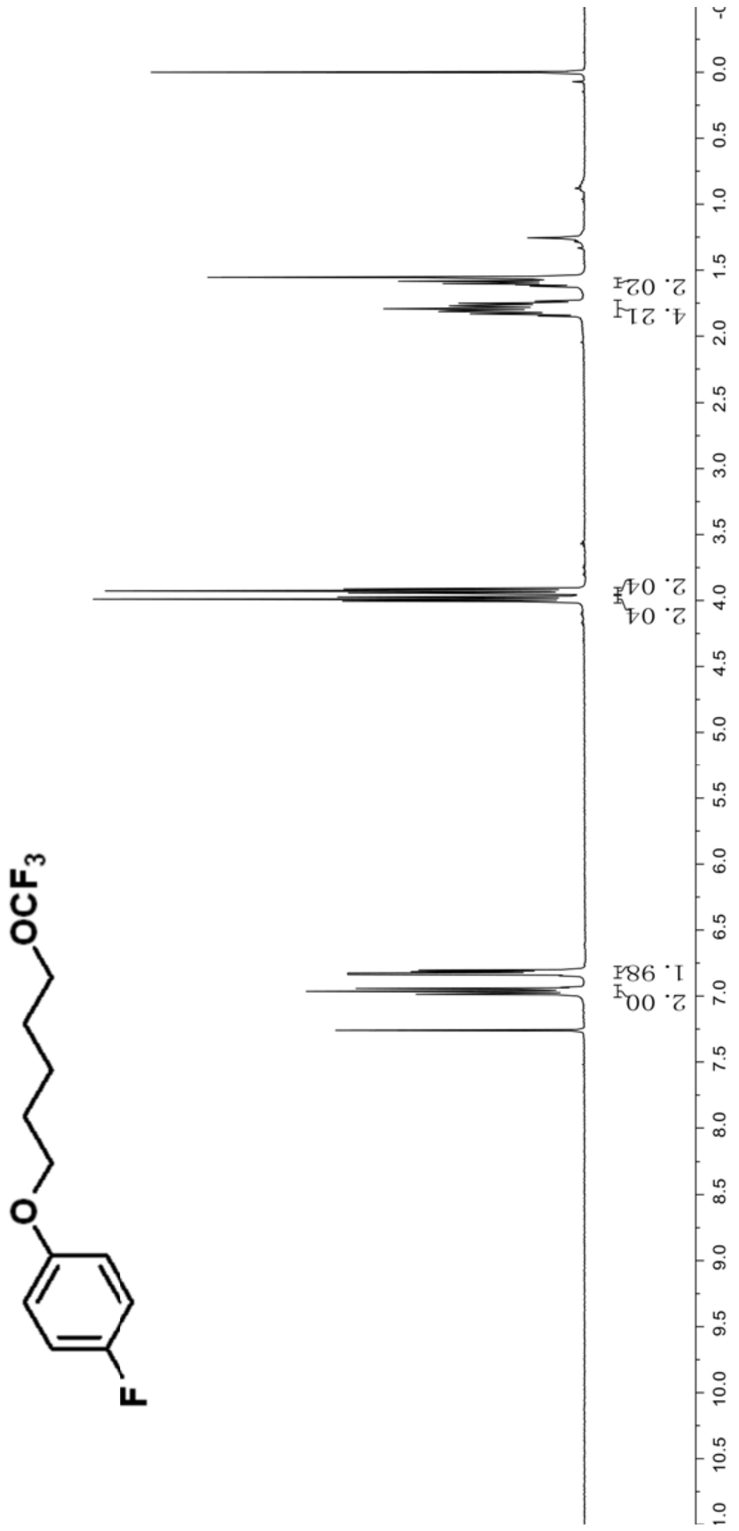

Supplementary Figure 111: ^{1}H NMR spectrum (400 MHz, $CDCl_3$, 23 °C) of **4**

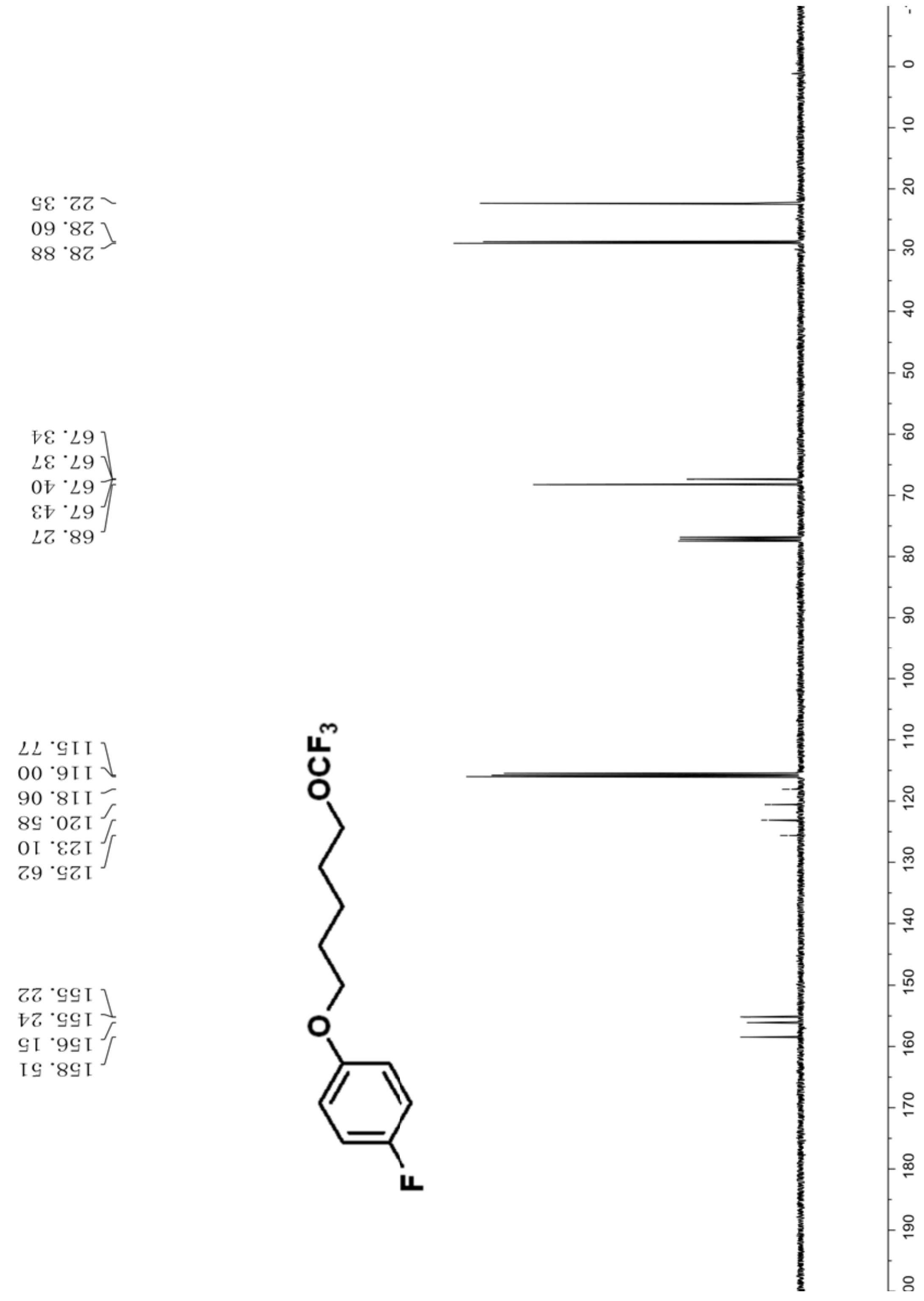

Supplementary Figure 112: ^{13}C NMR spectrum (101 MHz, $CDCl_3$, 23 °C) of **4**

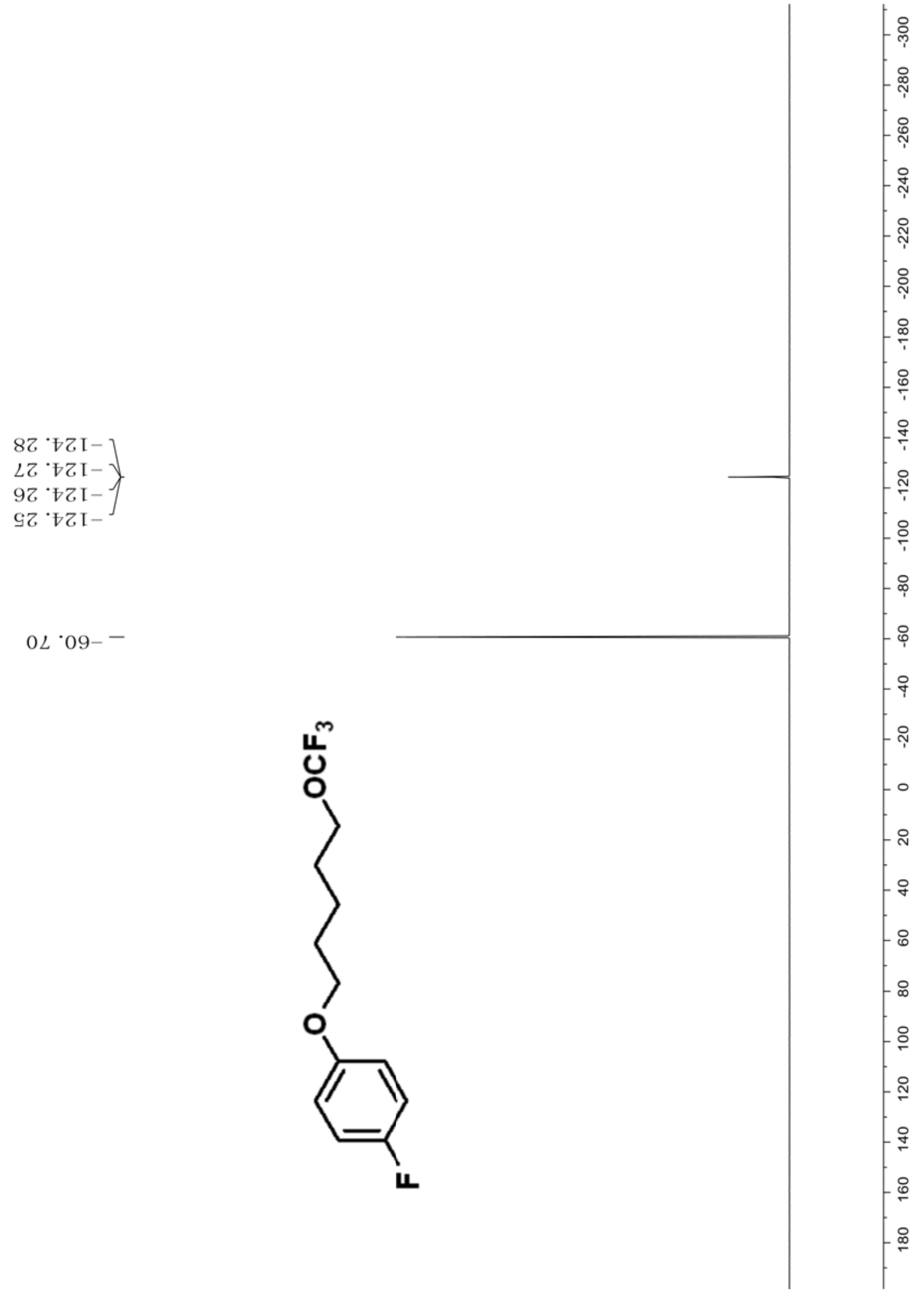

Supplementary Figure 113: ^{19}F NMR spectrum (376 MHz, $CDCl_3$, 23 °C) of **4**

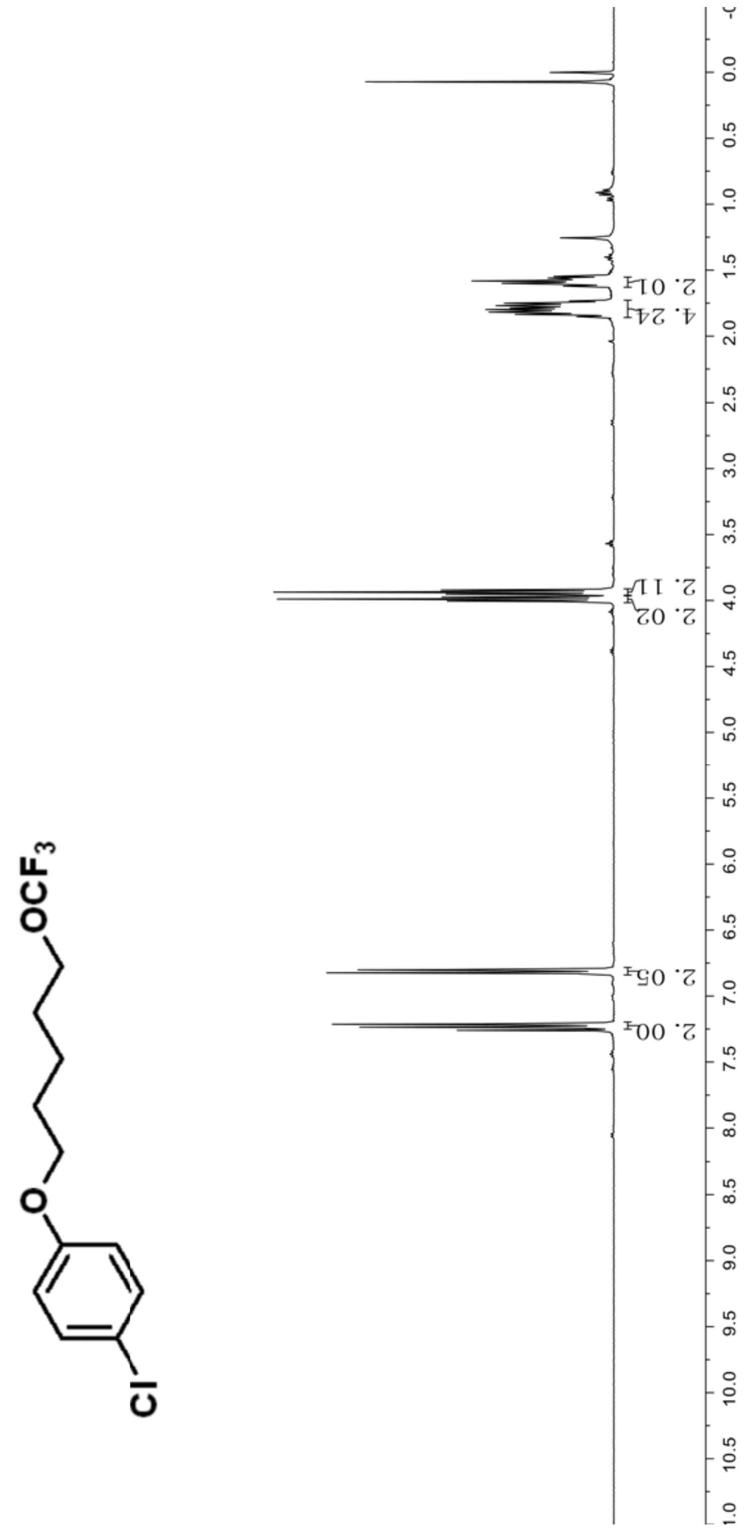

Supplementary Figure 114: ^{1}H NMR spectrum (400 MHz, $CDCl_3$, 23 °C) of **5**

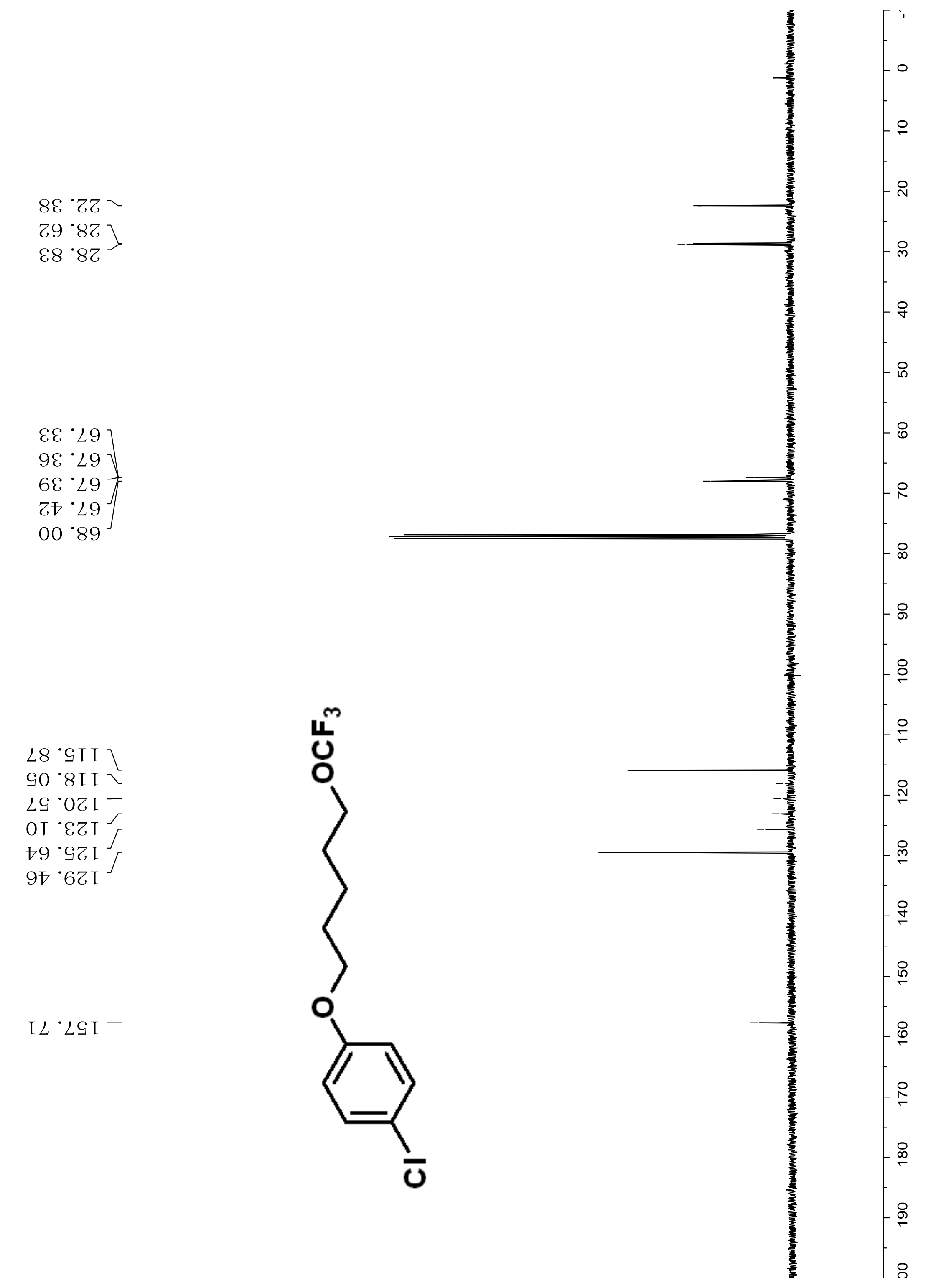

Supplementary Figure 115: ^{13}C NMR spectrum (101 MHz, $CDCl_3$, 23 °C) of **5**

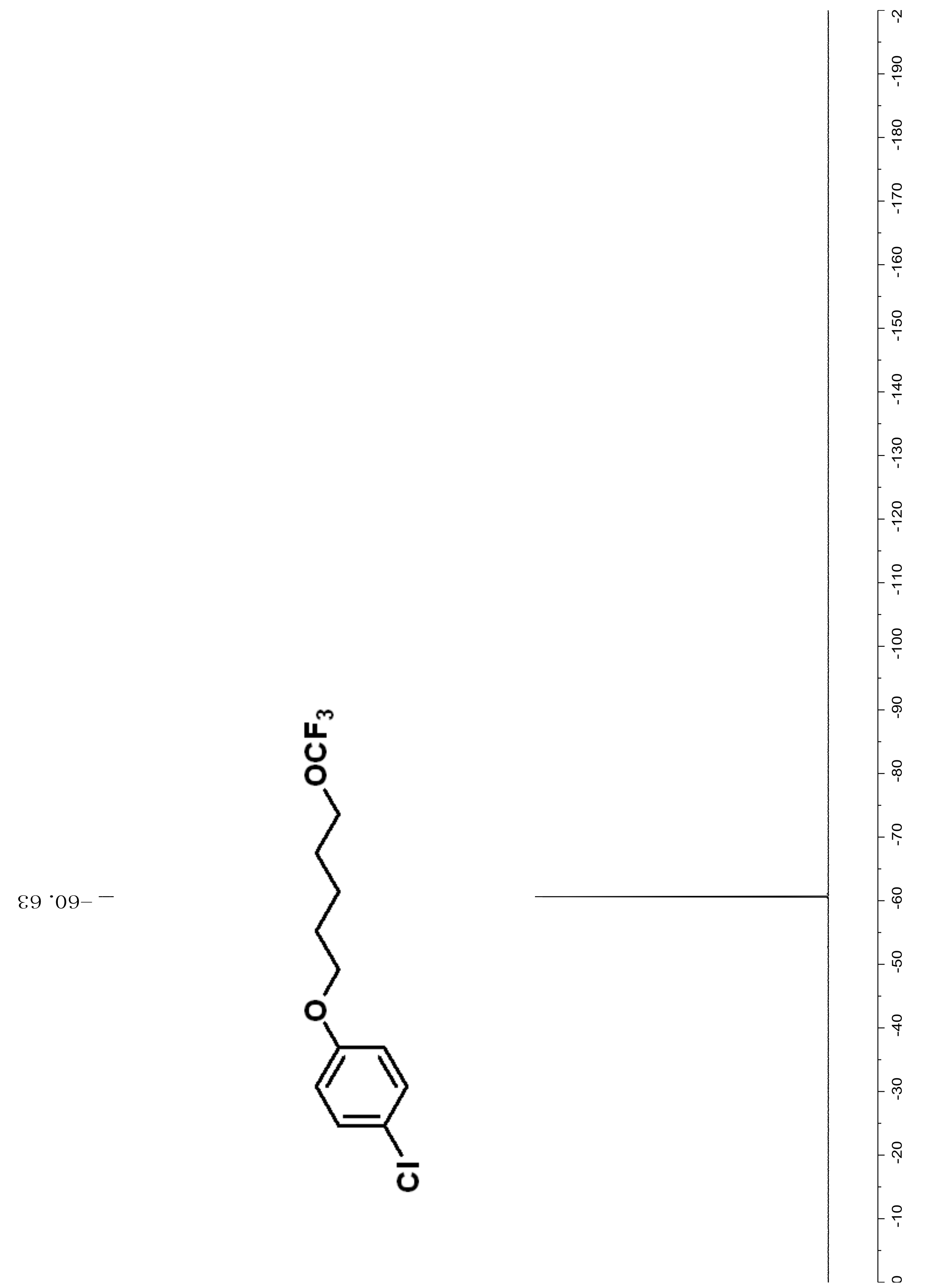

Supplementary Figure 116: ^{19}F NMR spectrum (376 MHz, $CDCl_3$, 23 °C) of **5**

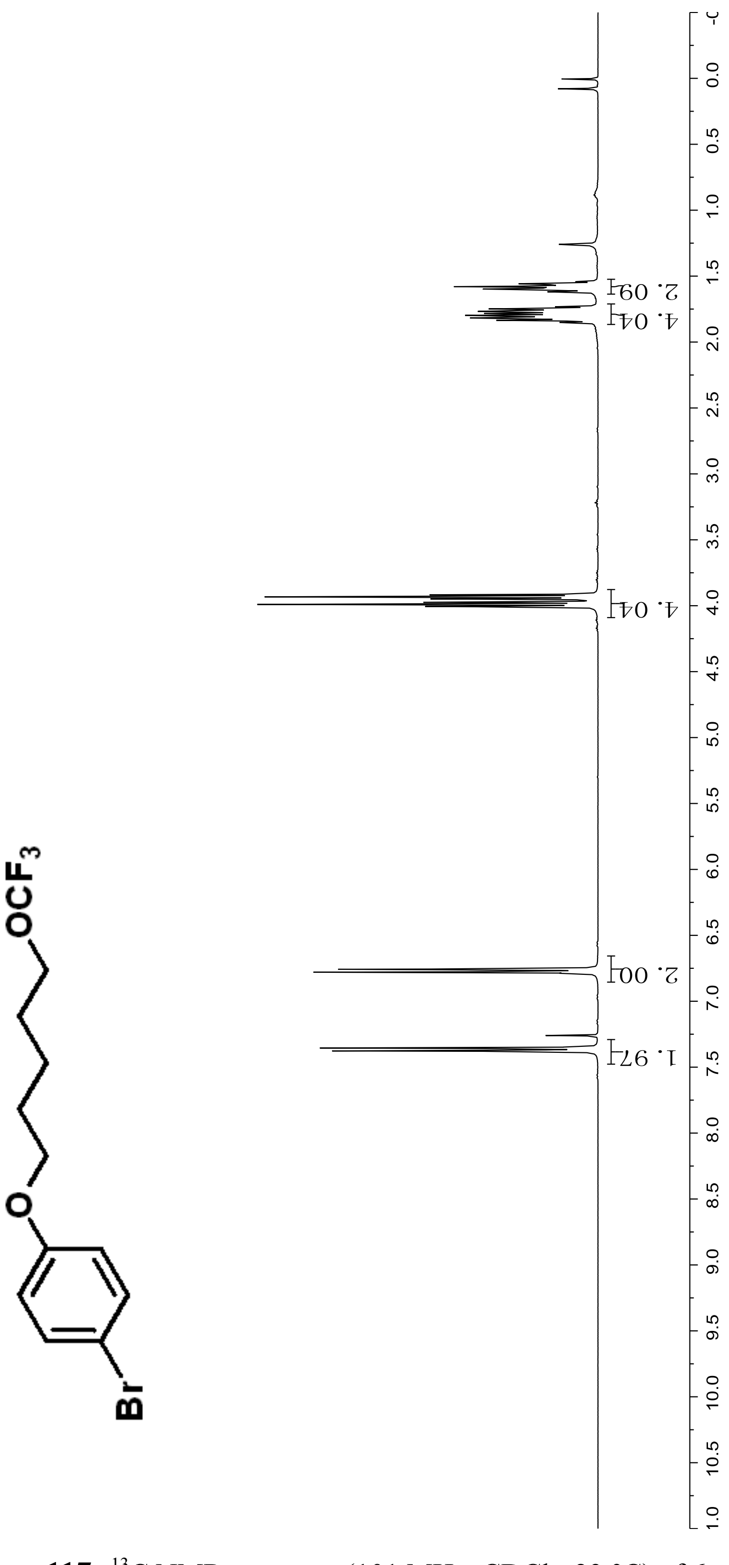

Supplementary Figure 117: ^{13}C NMR spectrum (101 MHz, $CDCl_3$, 23 °C) of **6**

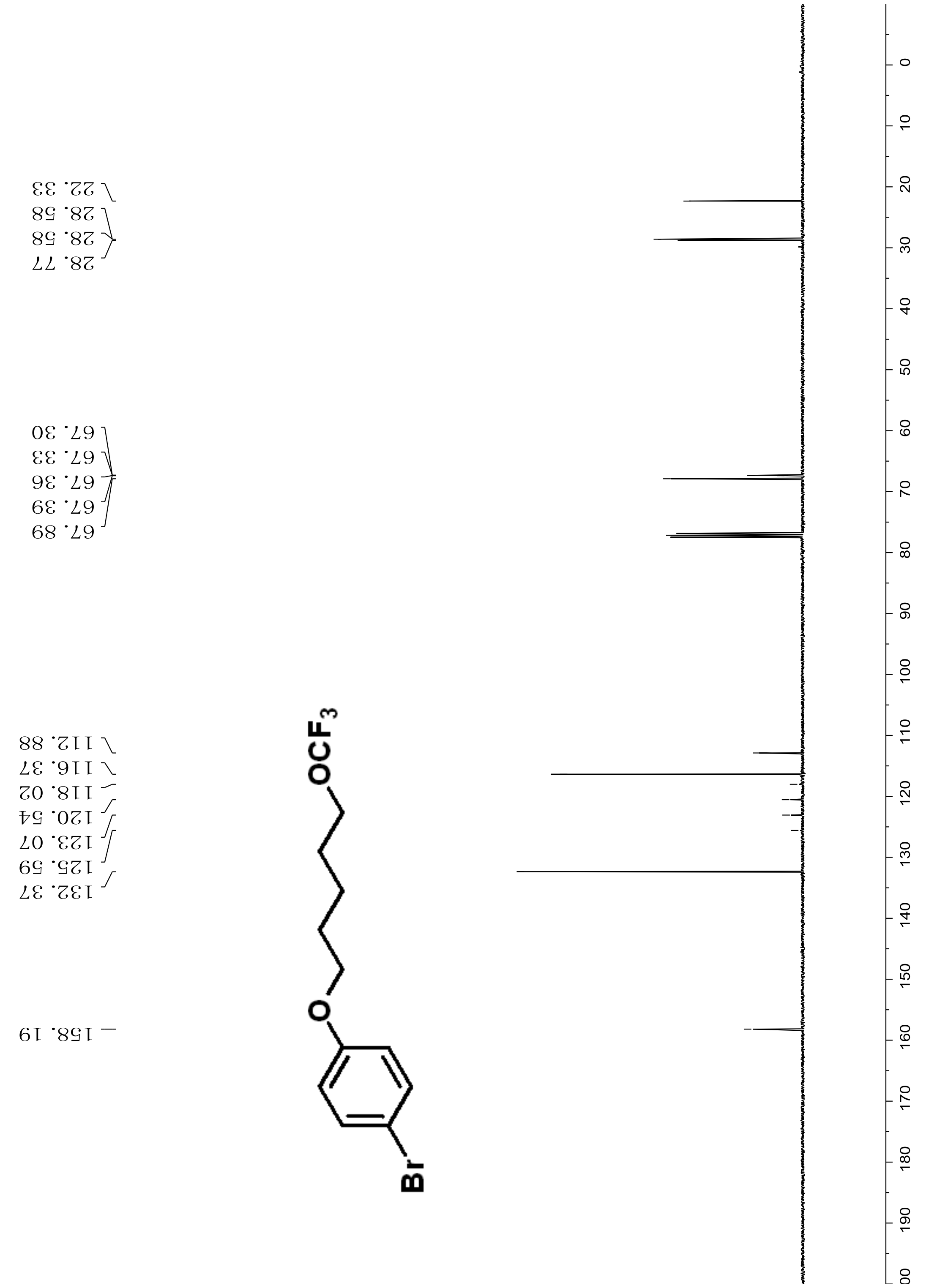

Supplementary Figure 118: ^{13}C NMR spectrum (101 MHz, $CDCl_3$, 23 °C) of **6**

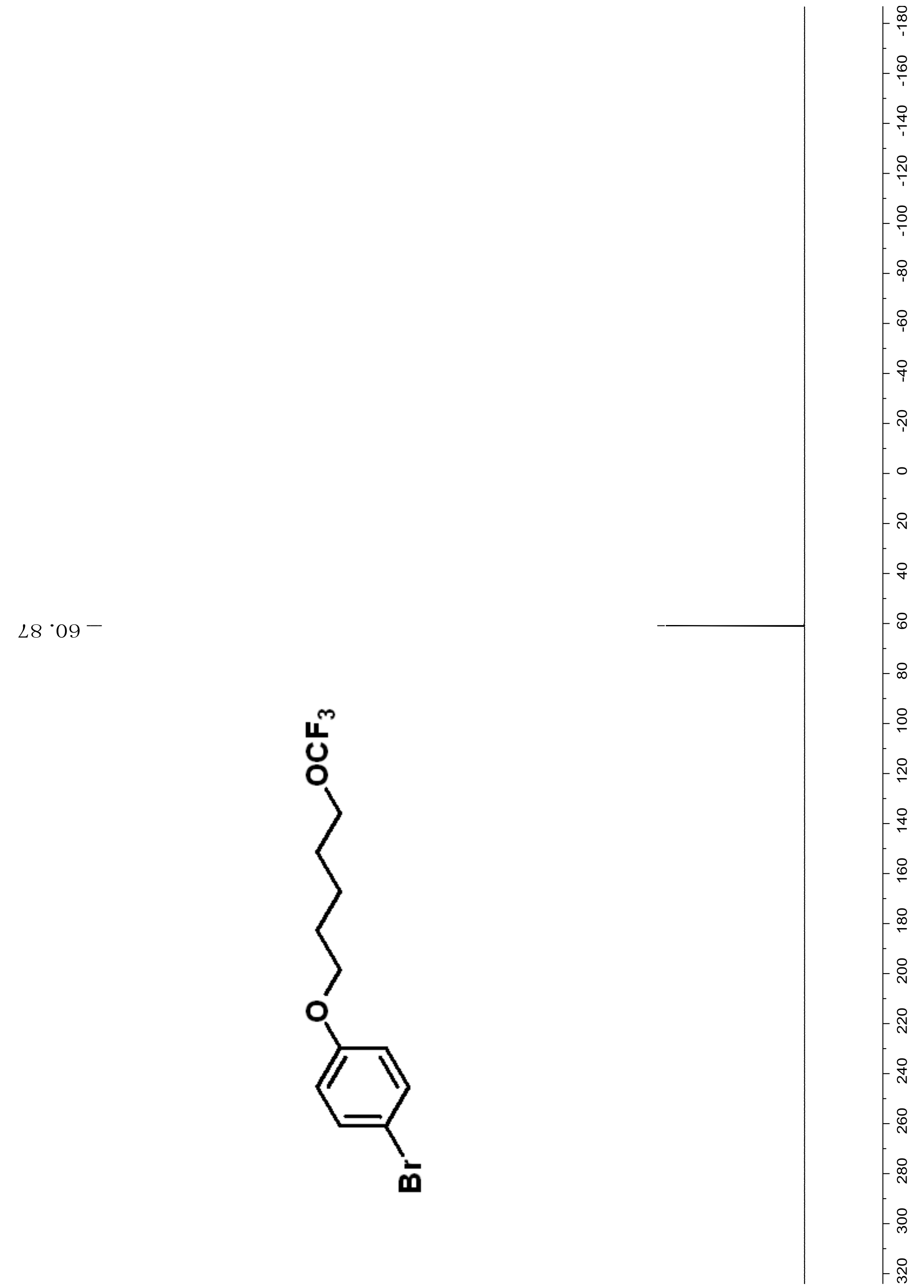

Supplementary Figure 119: ^{19}F NMR spectrum (376 MHz, DMSO, 23 °C) of **6**

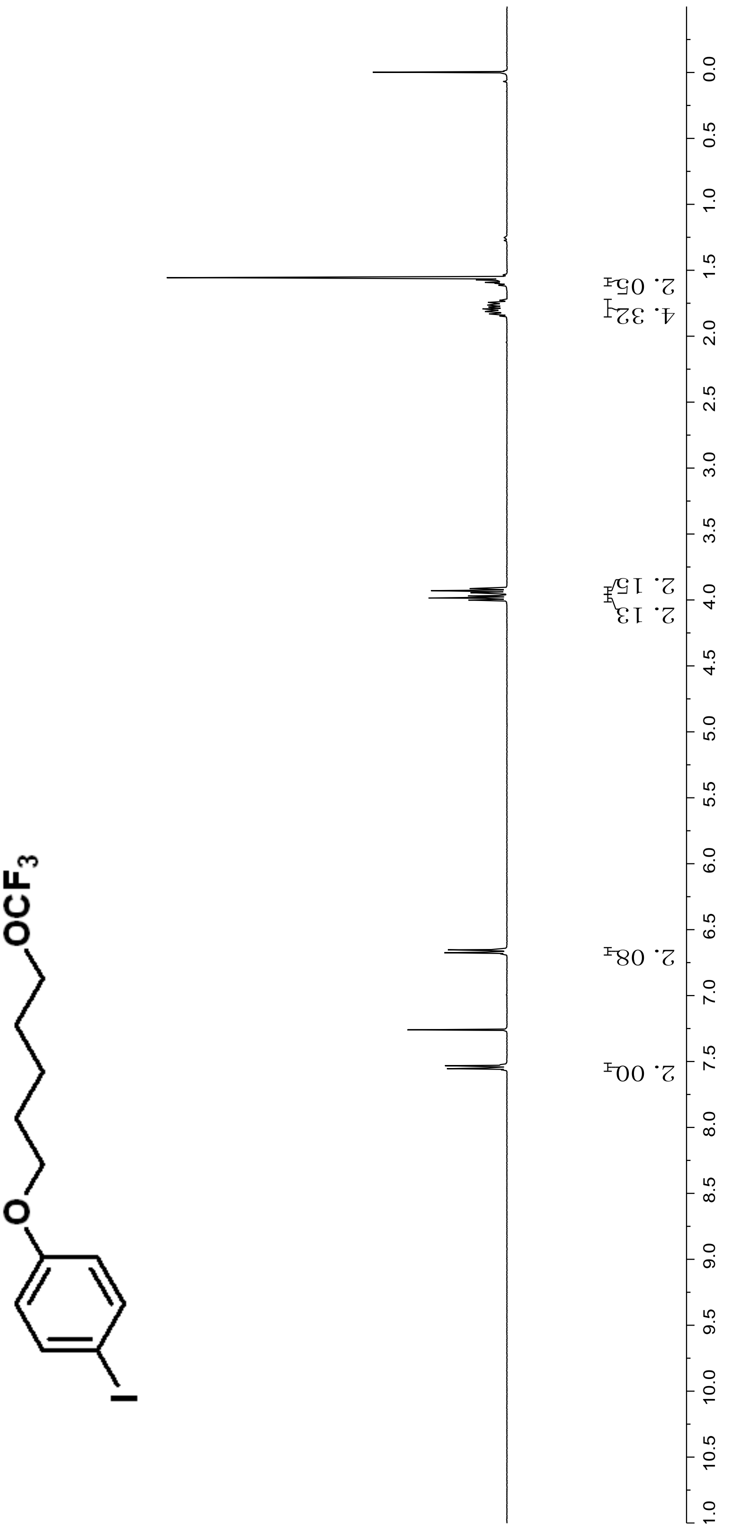

Supplementary Figure 120: ^{1}H NMR spectrum (400 MHz, $CDCl_3$, 23 °C) of **7**

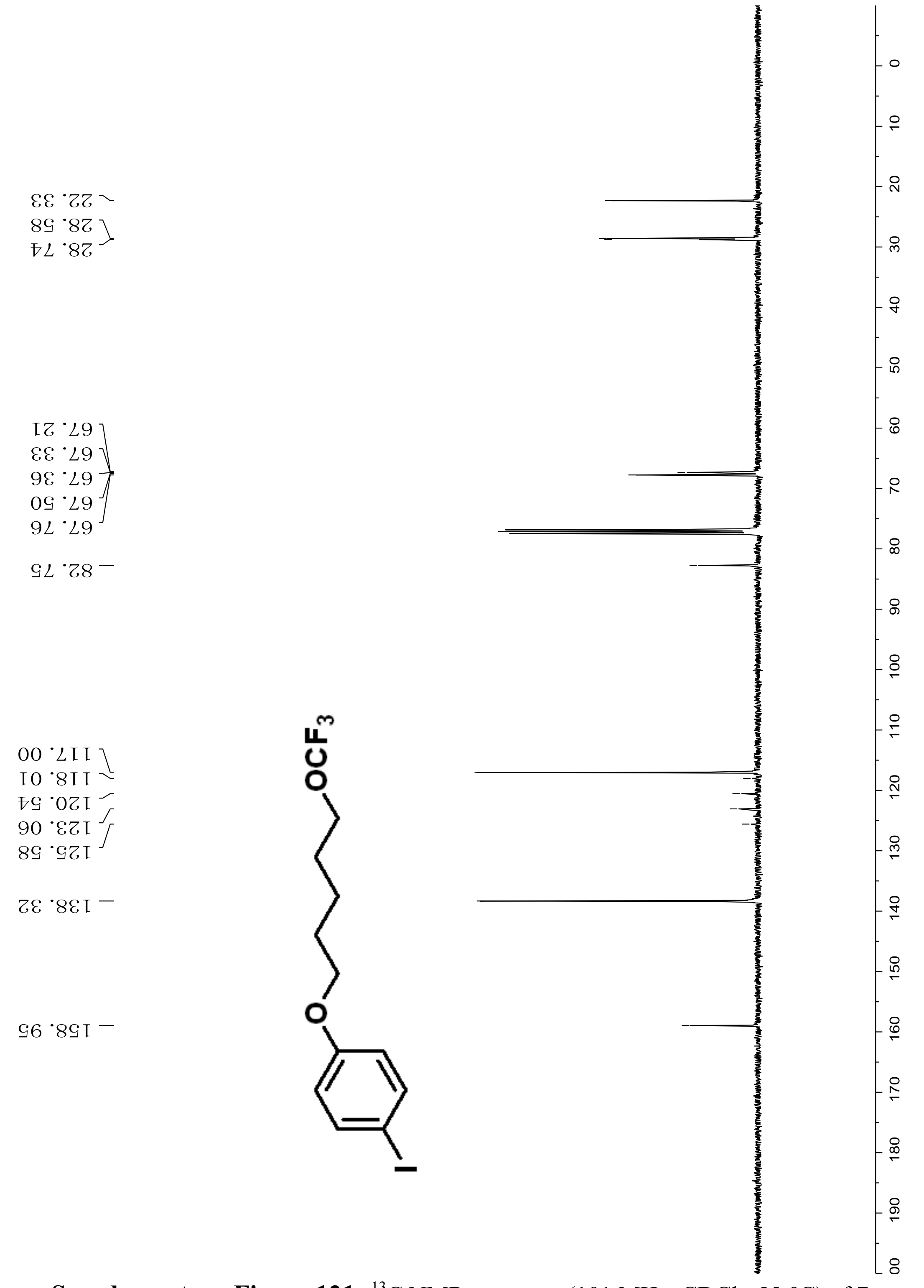

Supplementary Figure 121: ^{13}C NMR spectrum (101 MHz, $CDCl_3$, 23 °C) of **7**

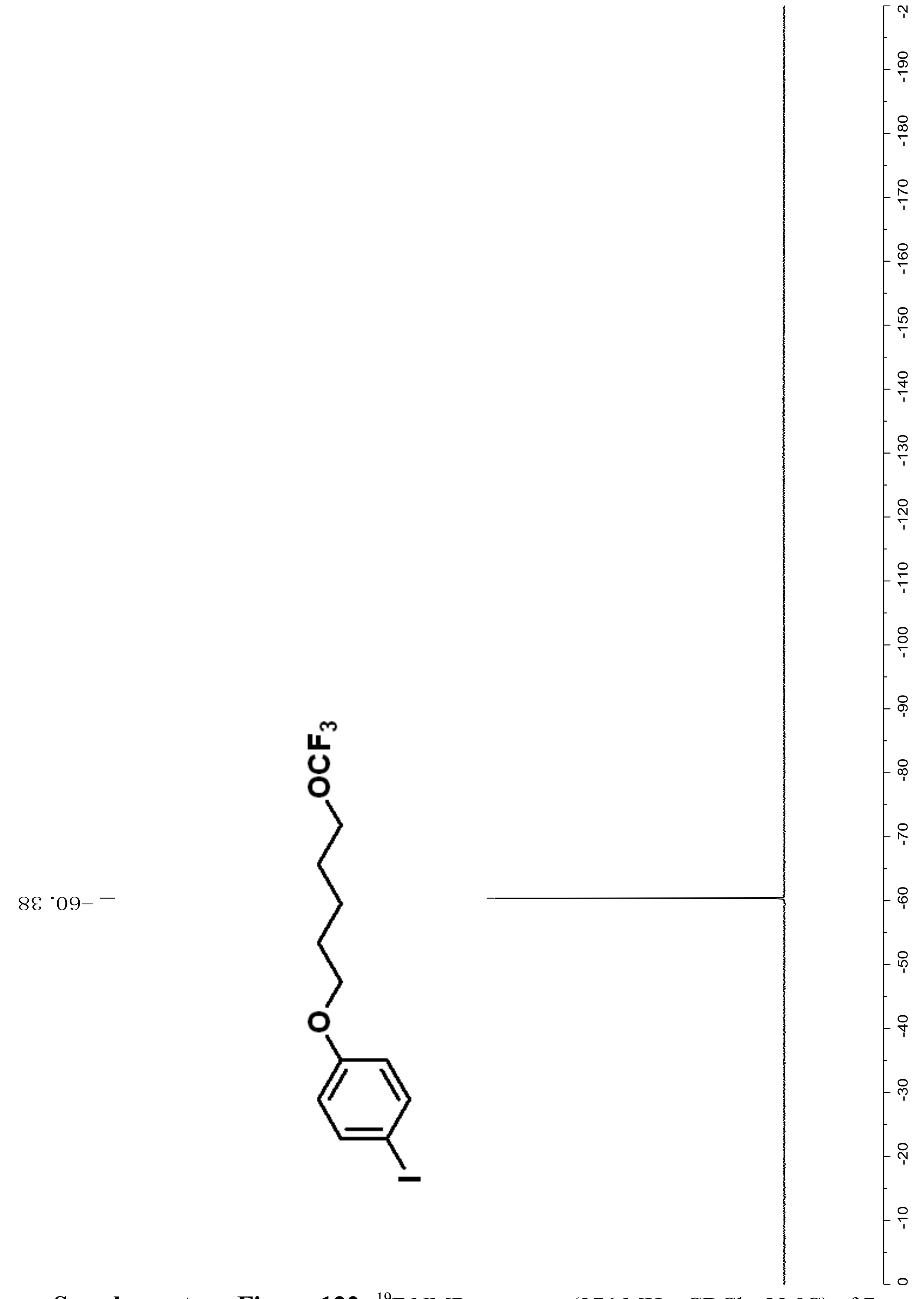

Supplementary Figure 122: ^{19}F NMR spectrum (376 MHz, $CDCl_3$, 23 °C) of **7**

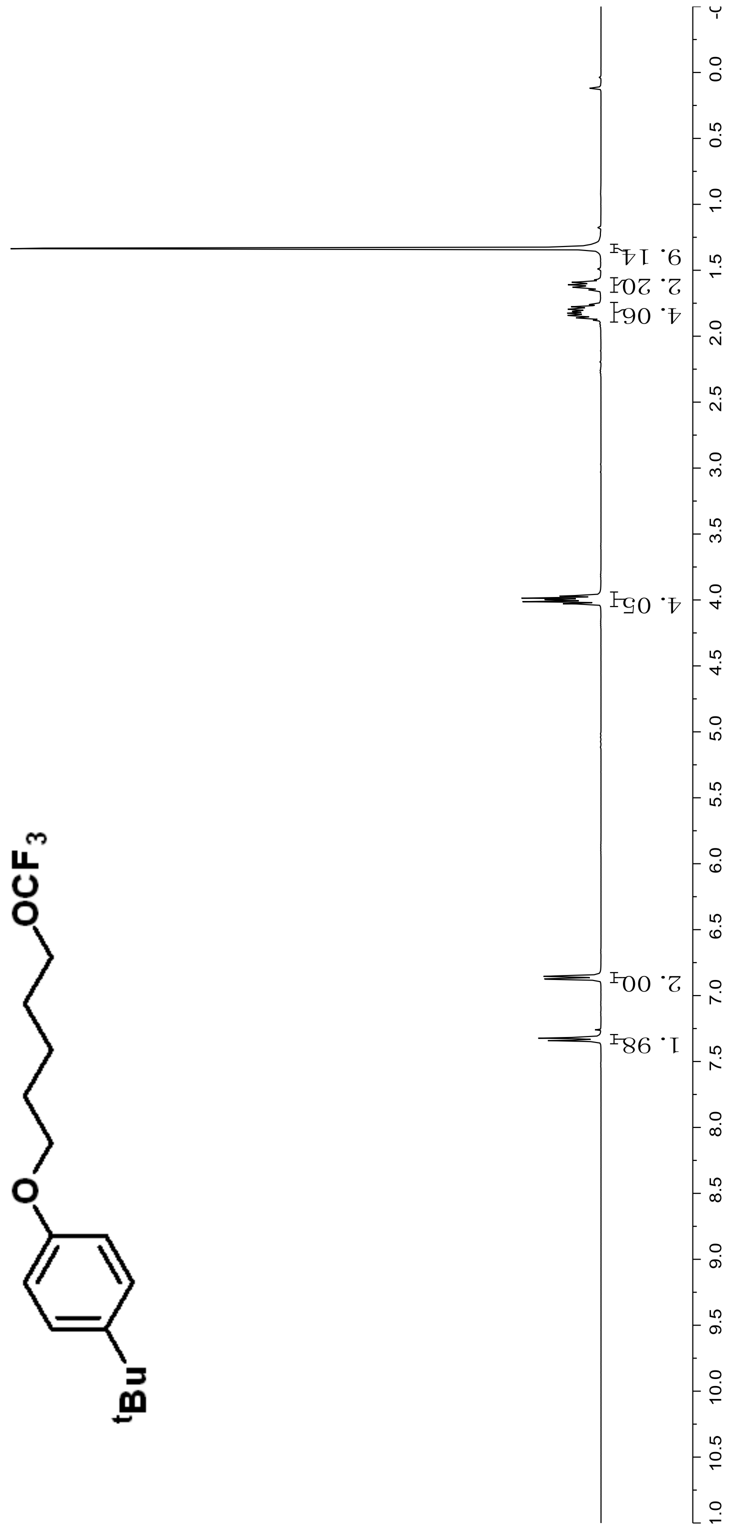

Supplementary Figure 123: ^{1}H NMR spectrum (400 MHz, $CDCl_3$, 23 °C) of **8**

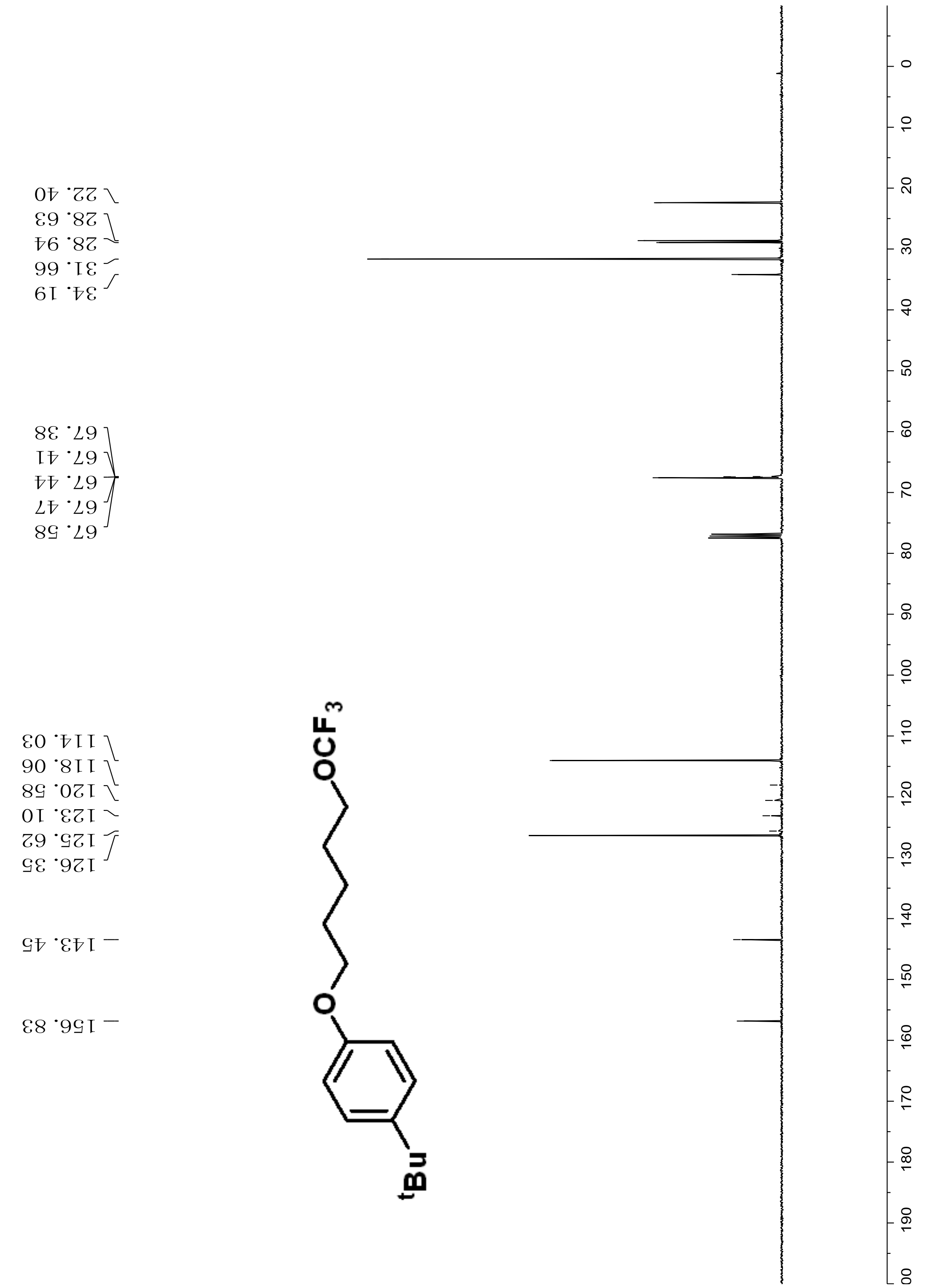

Supplementary Figure 124: ^{13}C NMR spectrum (101 MHz, $CDCl_3$, 23 °C) of **8**

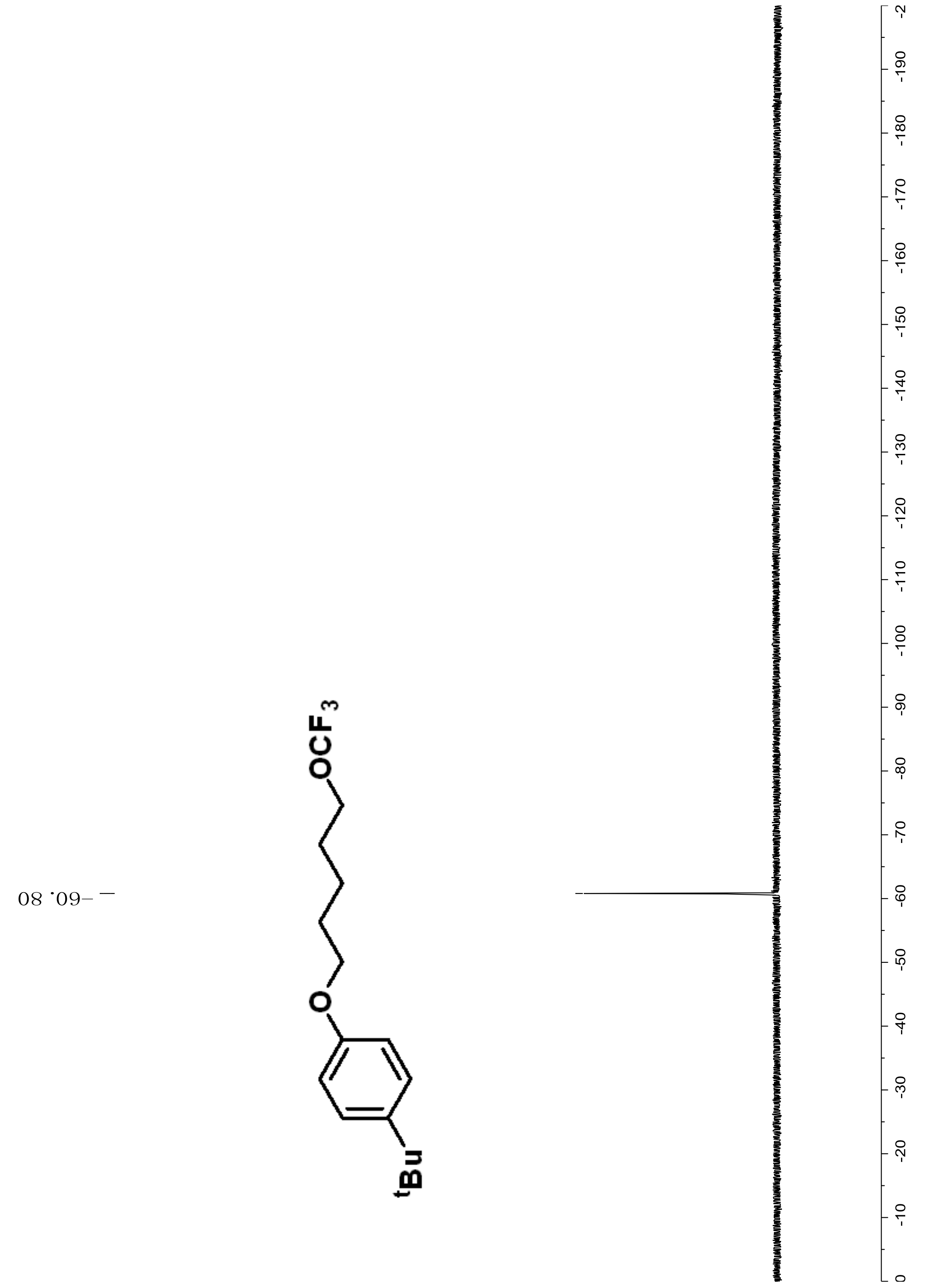

Supplementary Figure 125: ^{19}F NMR spectrum (376 MHz, $CDCl_3$, 23 °C) of **8**

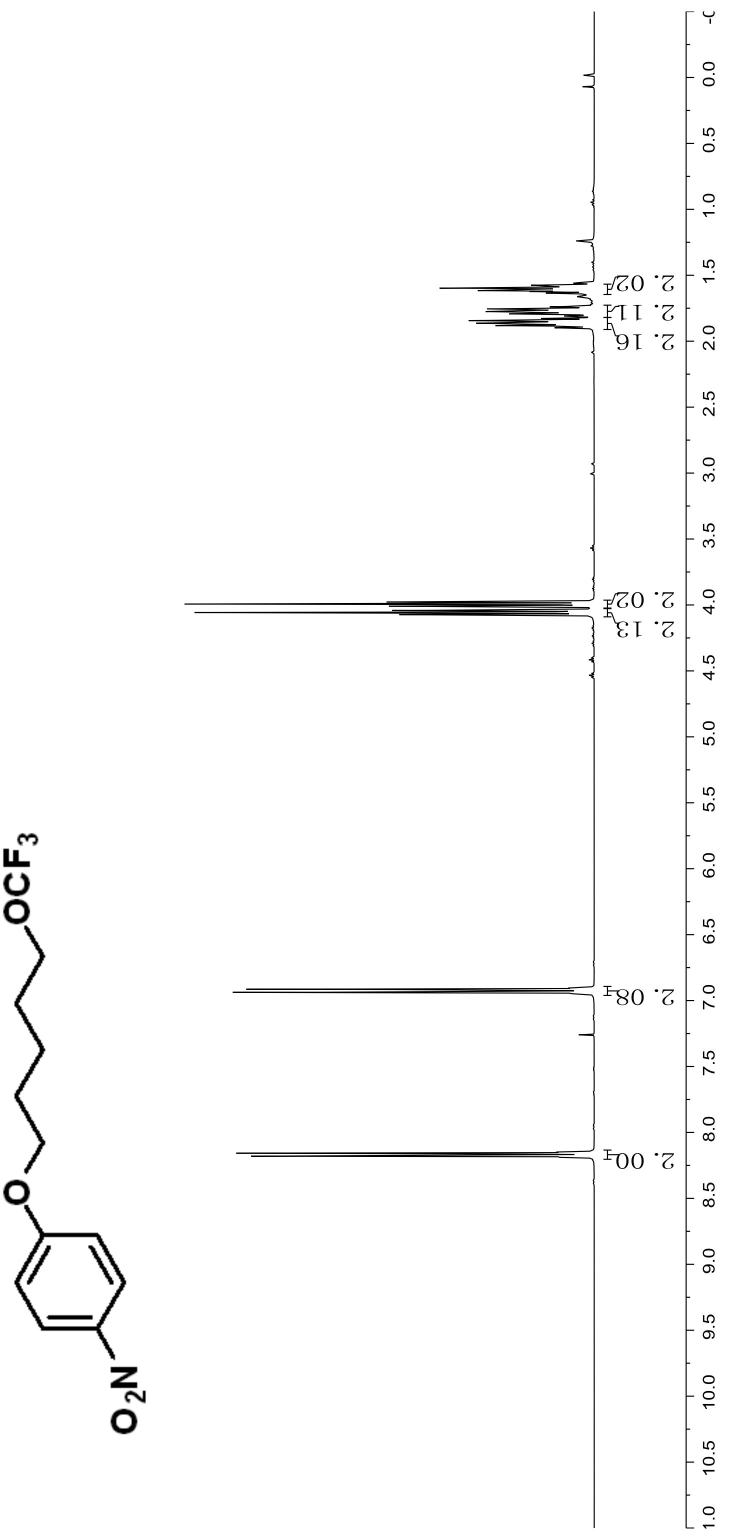

Supplementary Figure 126: ^{1}H NMR spectrum (400 MHz, $CDCl_3$, 23 °C) of **9**

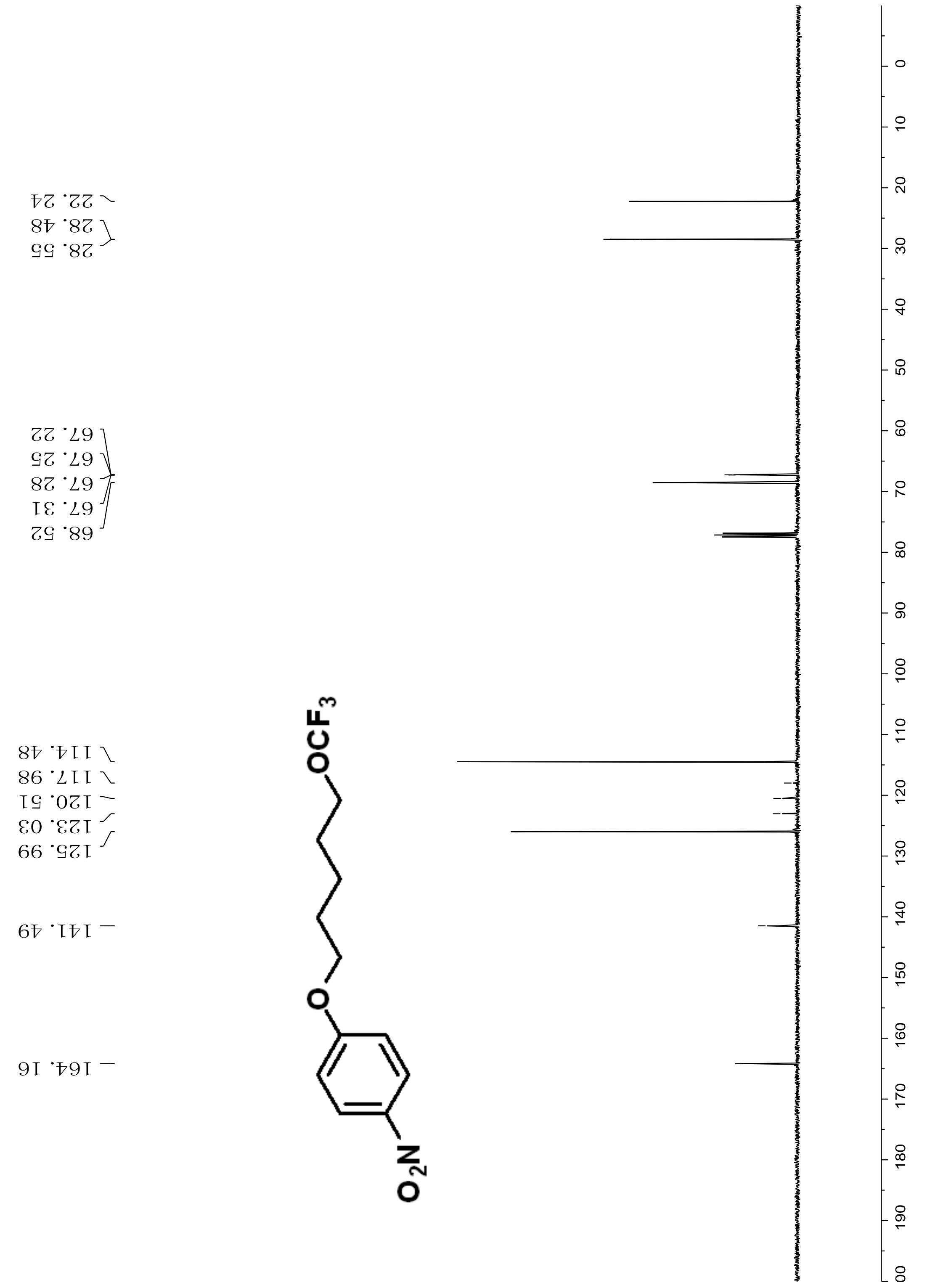

Supplementary Figure 127: ^{13}C NMR spectrum (101 MHz, $CDCl_3$, 23 °C) of **9**

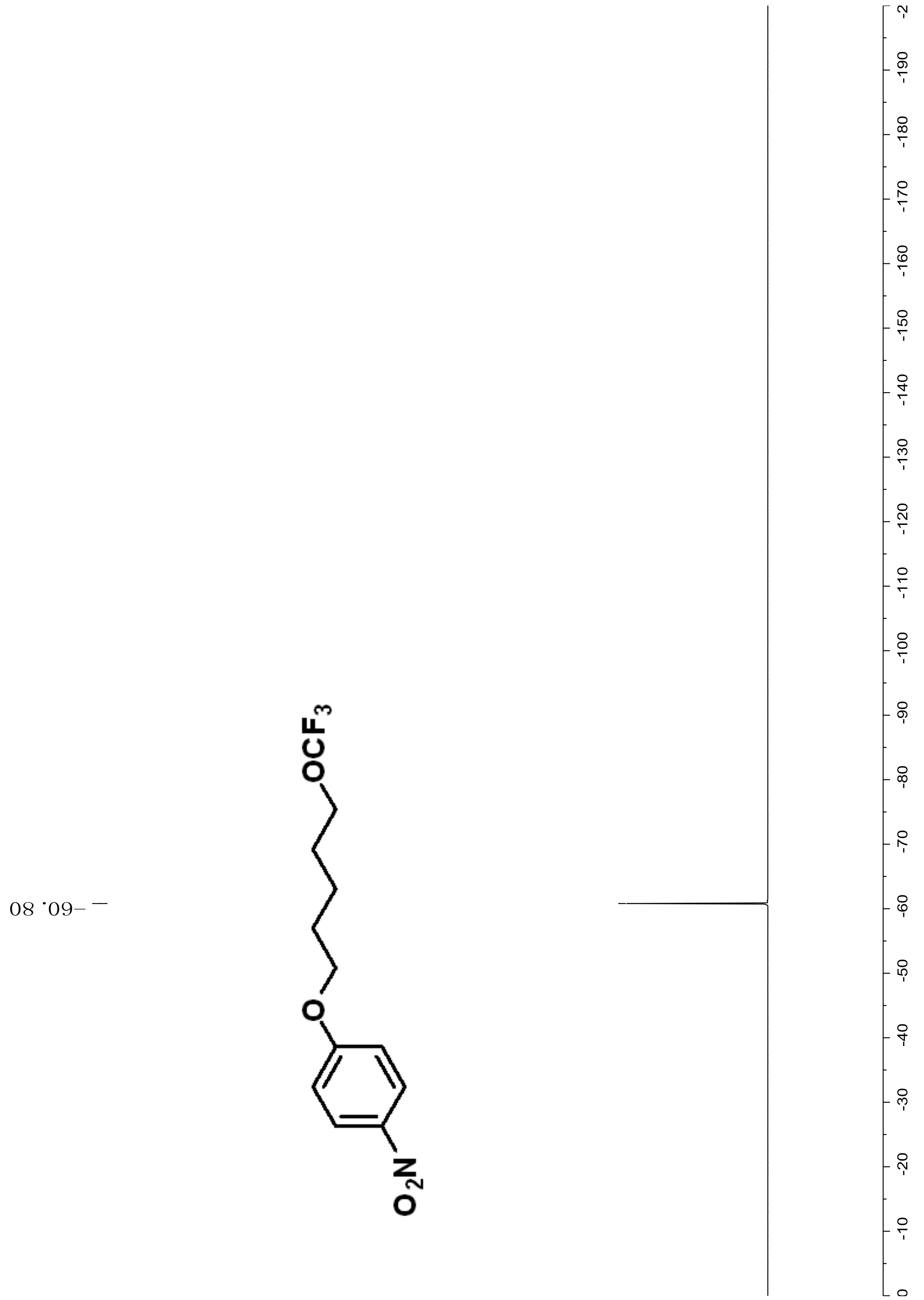

Supplementary Figure 128: ^{19}F NMR spectrum (376 MHz, $CDCl_3$, 23 °C) of **9**

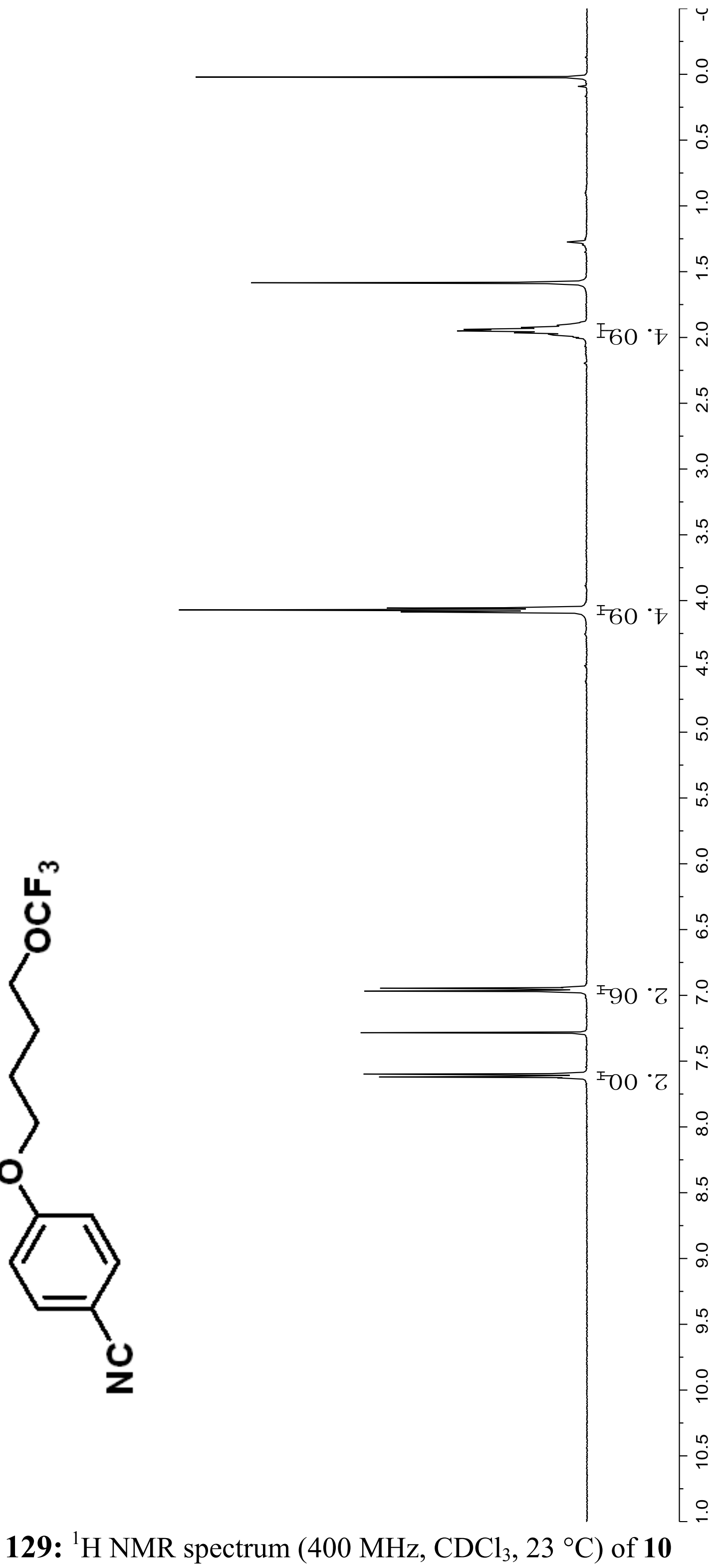

Supplementary Figure 129: ^{1}H NMR spectrum (400 MHz, $CDCl_3$, 23 °C) of **10**

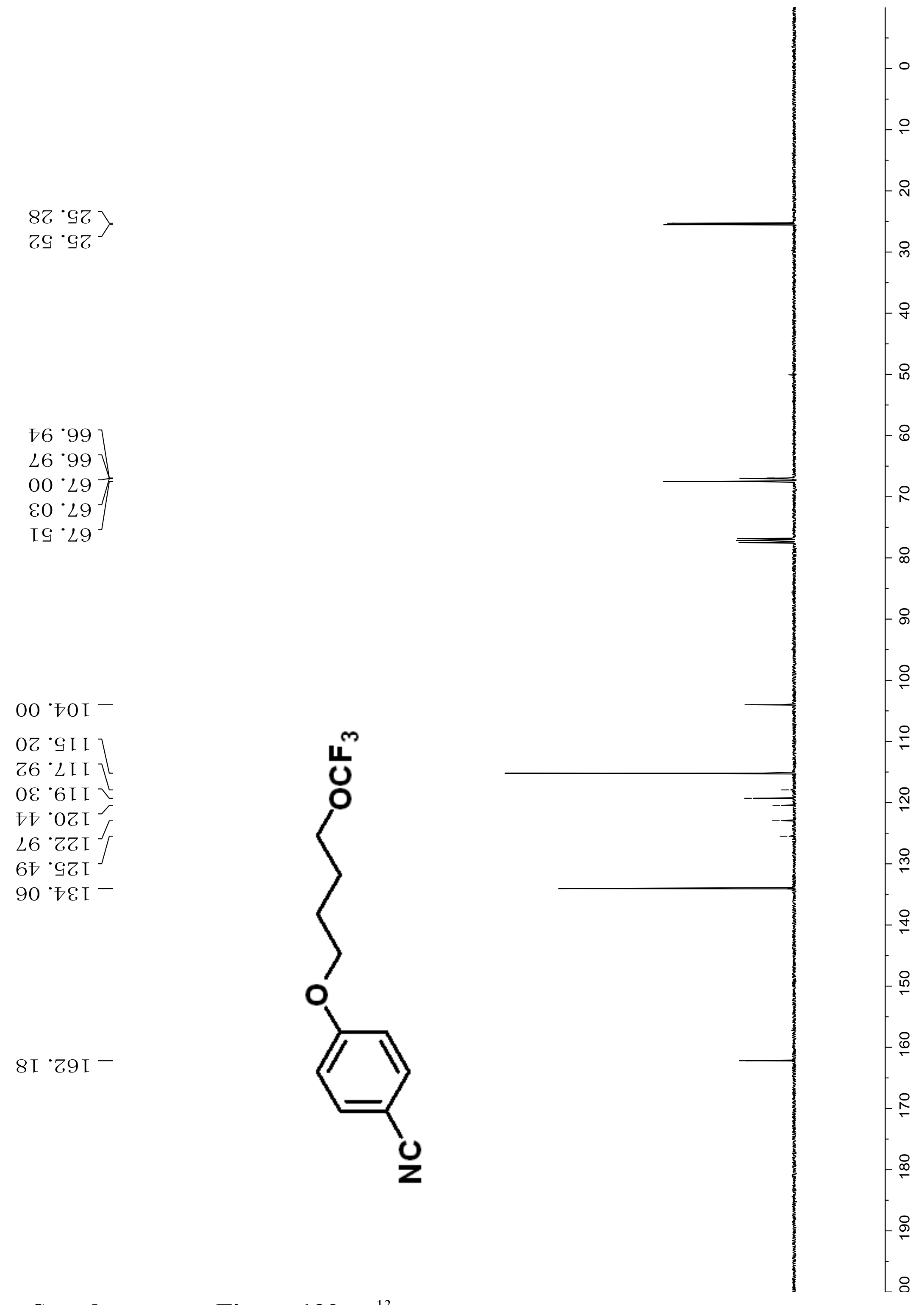

Supplementary Figure 130: ^{13}C NMR spectrum (101 MHz, $CDCl_3$, 23 °C) of **10**

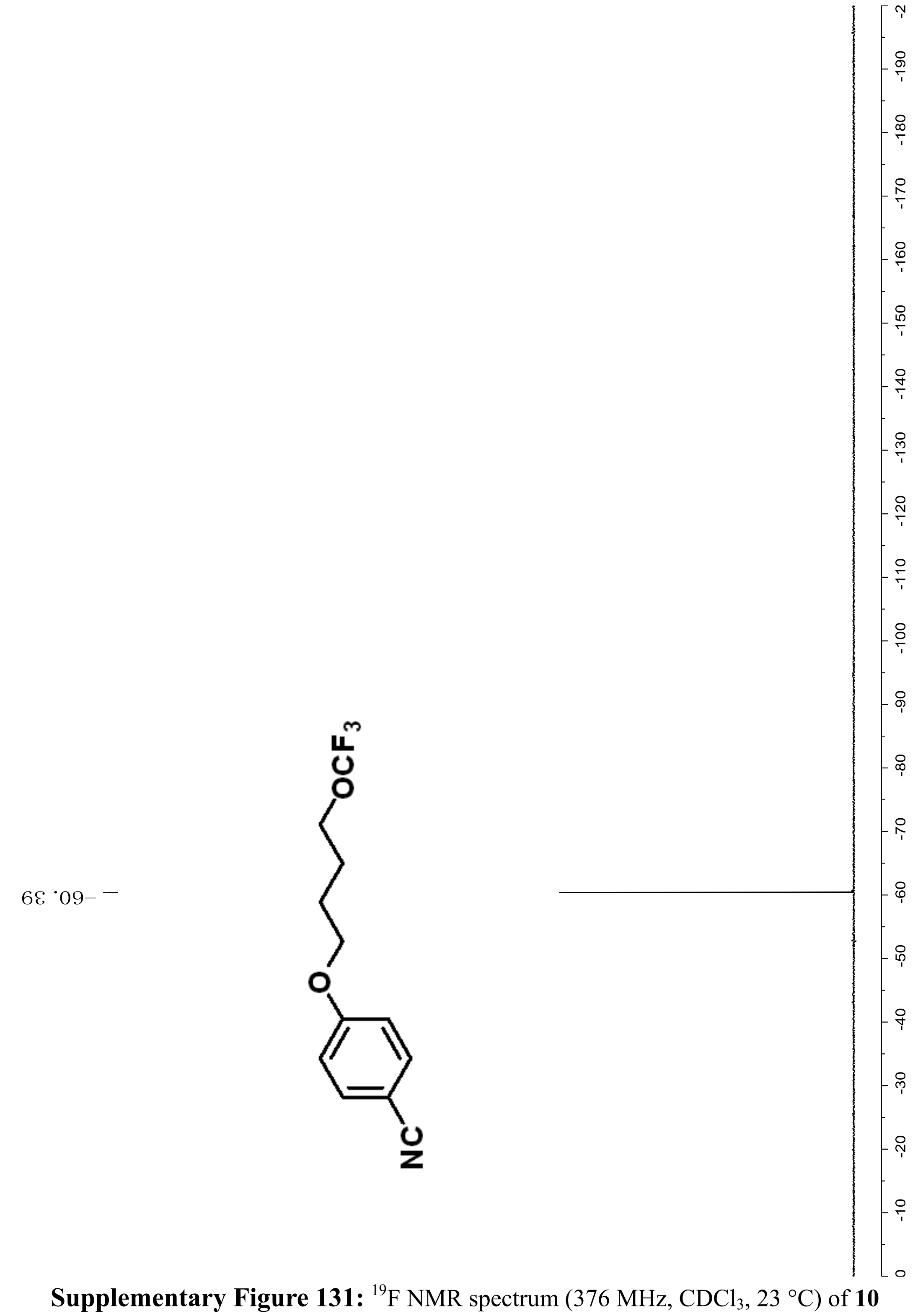

Supplementary Figure 131: ^{19}F NMR spectrum (376 MHz, $CDCl_3$, 23 °C) of **10**

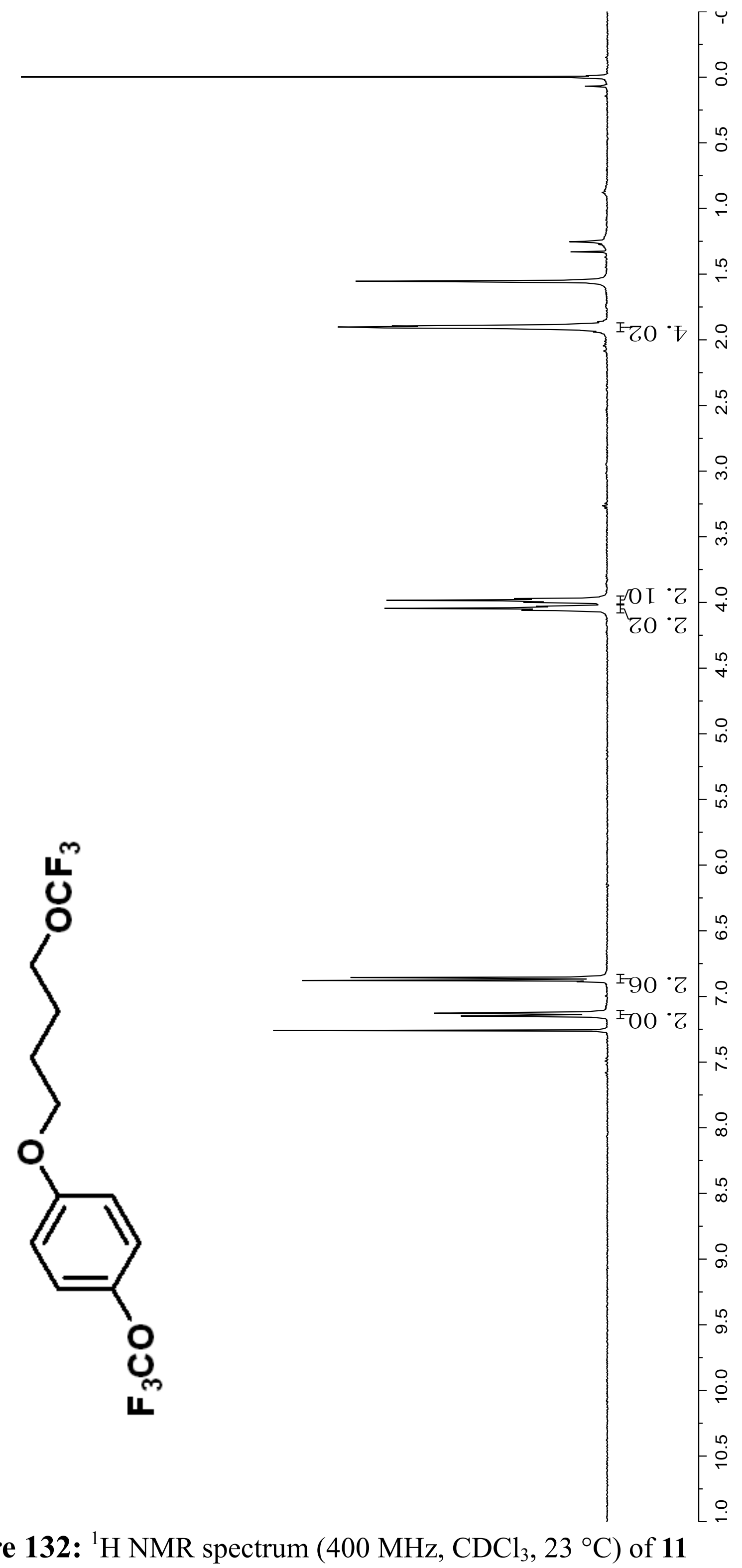

Supplementary Figure 132: ^{1}H NMR spectrum (400 MHz, $CDCl_3$, 23 °C) of **11**

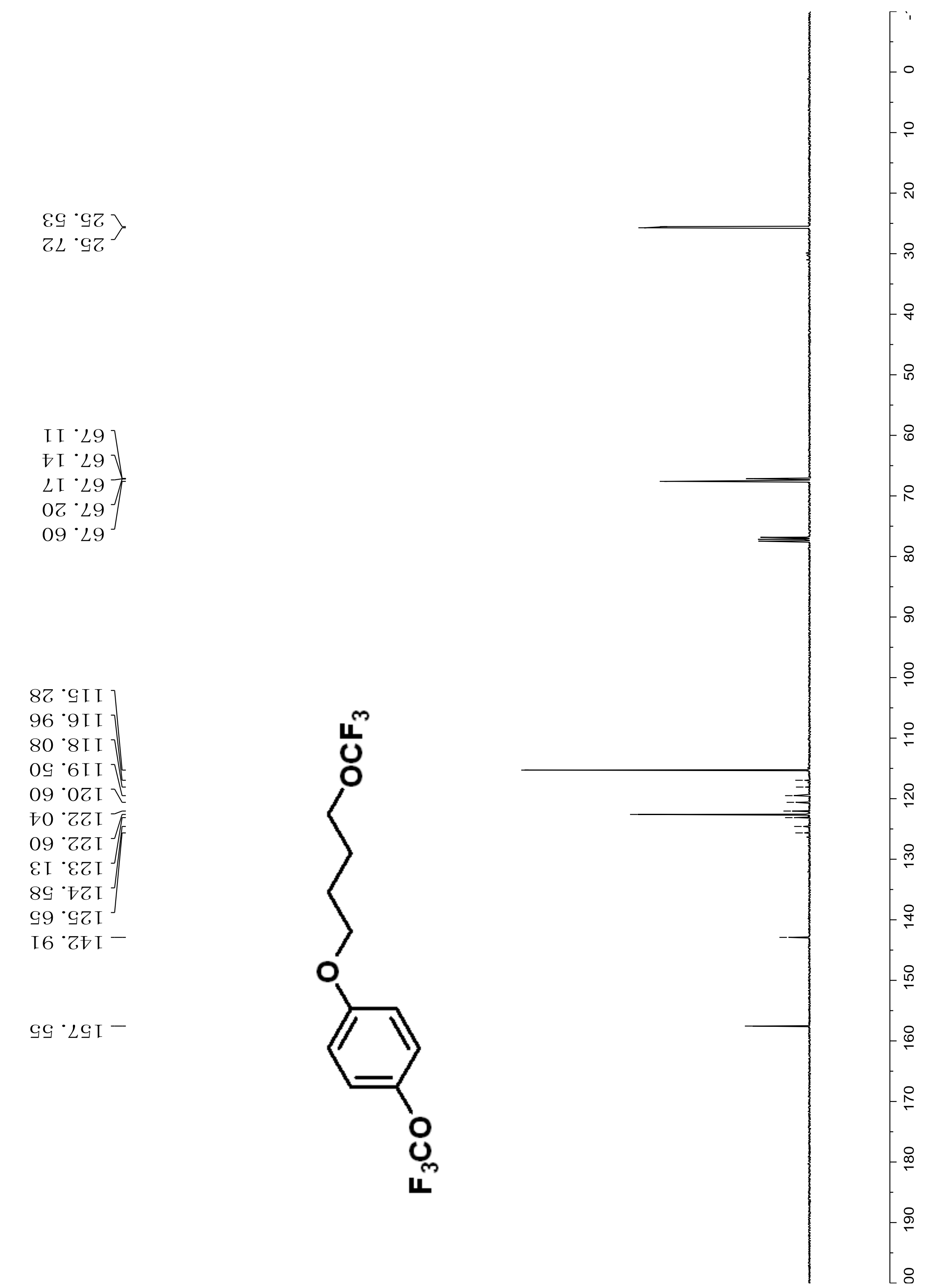

Supplementary Figure 133: ^{13}C NMR spectrum (101 MHz, $CDCl_3$, 23 °C) of **11**

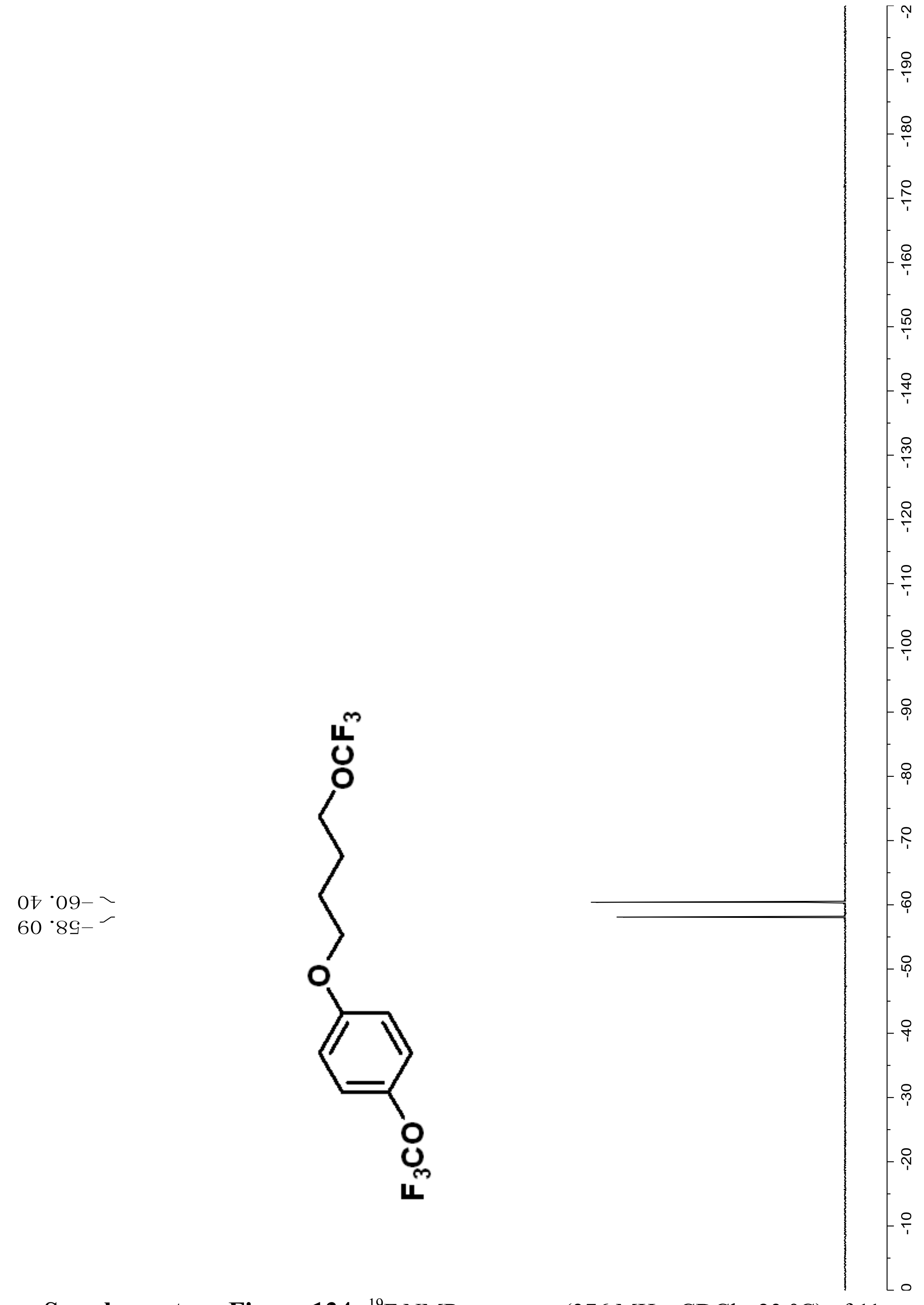

Supplementary Figure 134: ^{19}F NMR spectrum (376 MHz, $CDCl_3$, 23 °C) of **11**

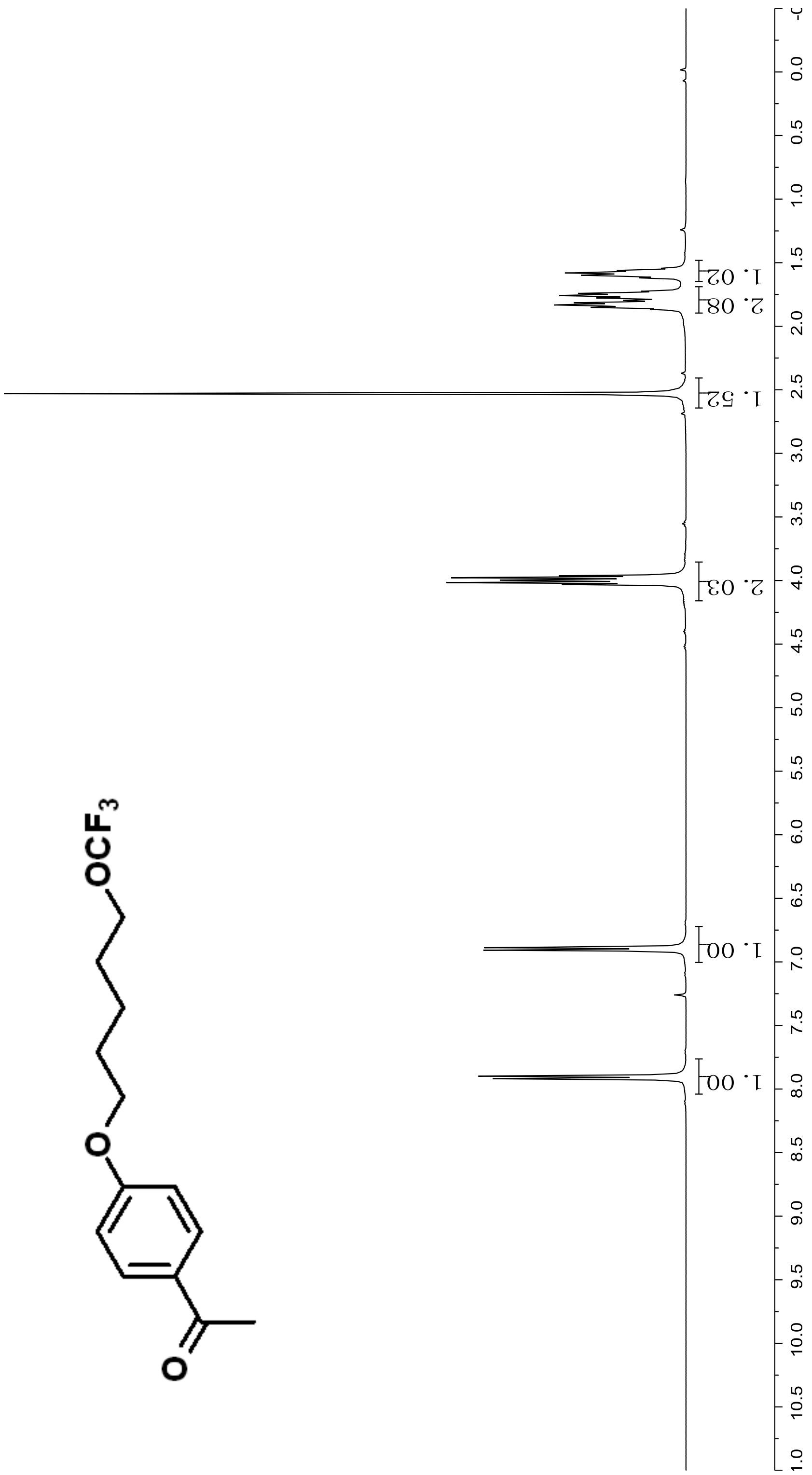

Supplementary Figure 135: ^{1}H NMR spectrum (400 MHz, $CDCl_3$, 23 °C) of **12**

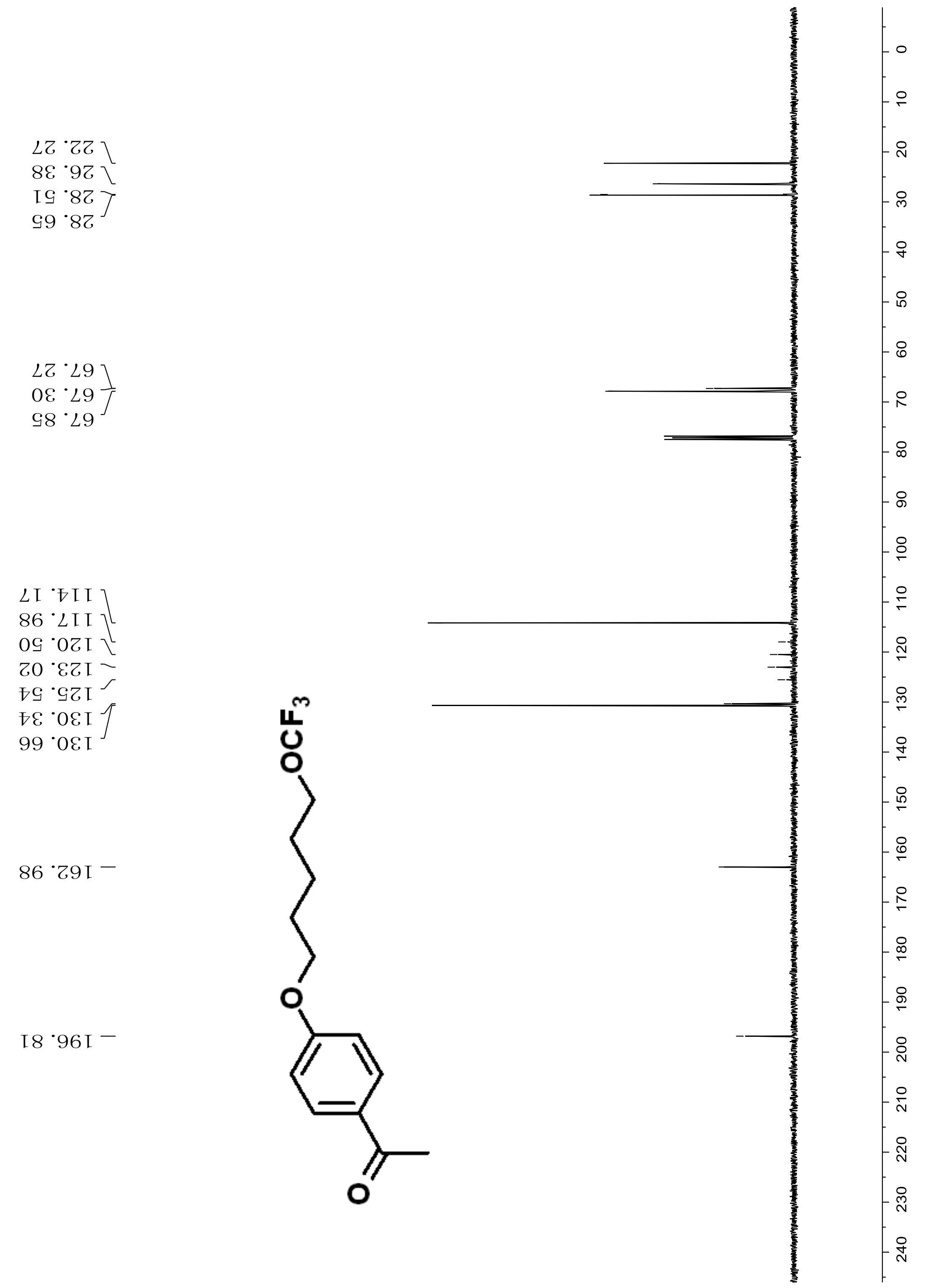

Supplementary Figure 136: ^{13}C NMR spectrum (101 MHz, $CDCl_3$, 23 °C) of **12**

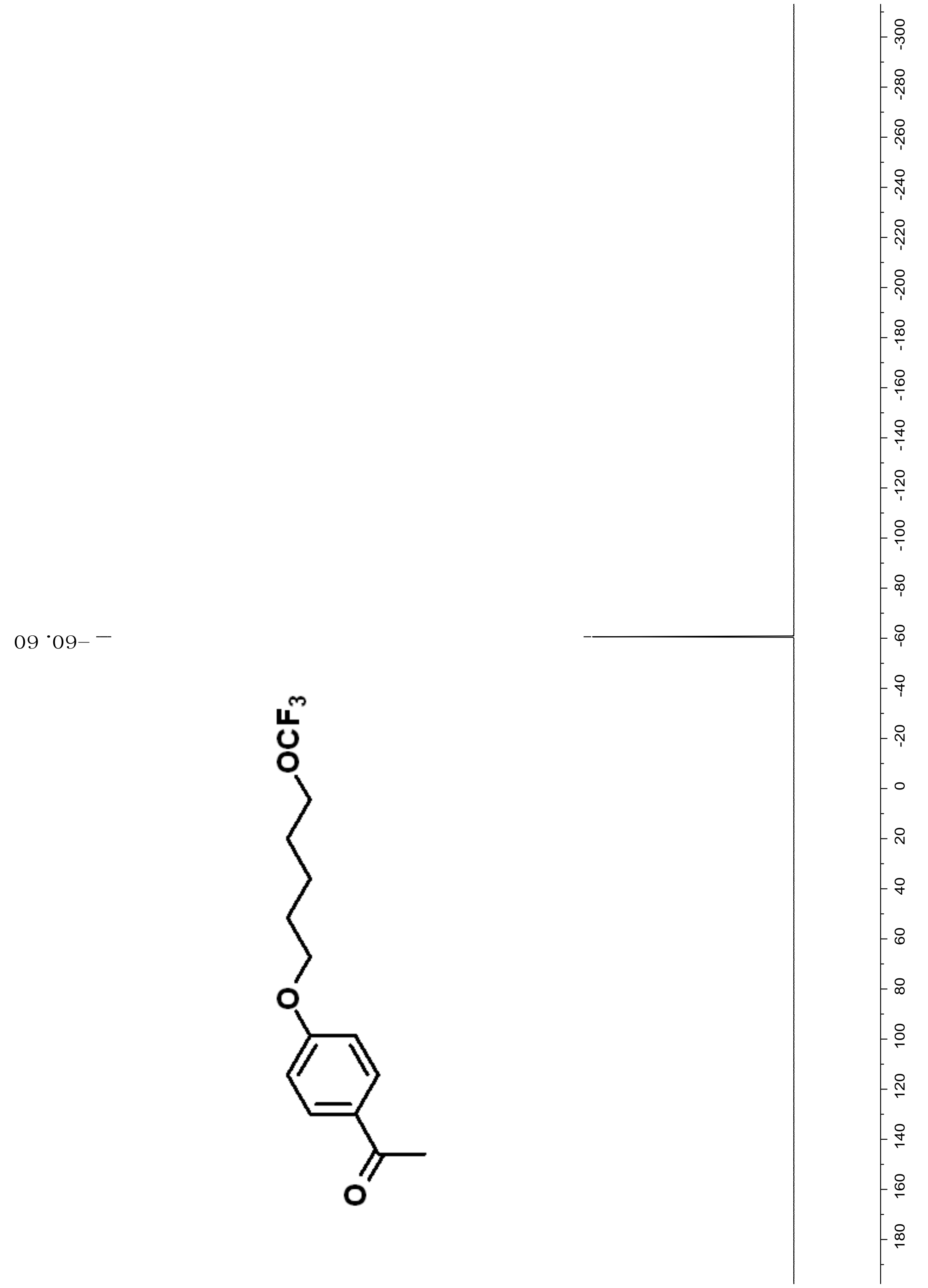

Supplementary Figure 137: ^{19}F NMR spectrum (376 MHz, $CDCl_3$, 23 °C) of **12**

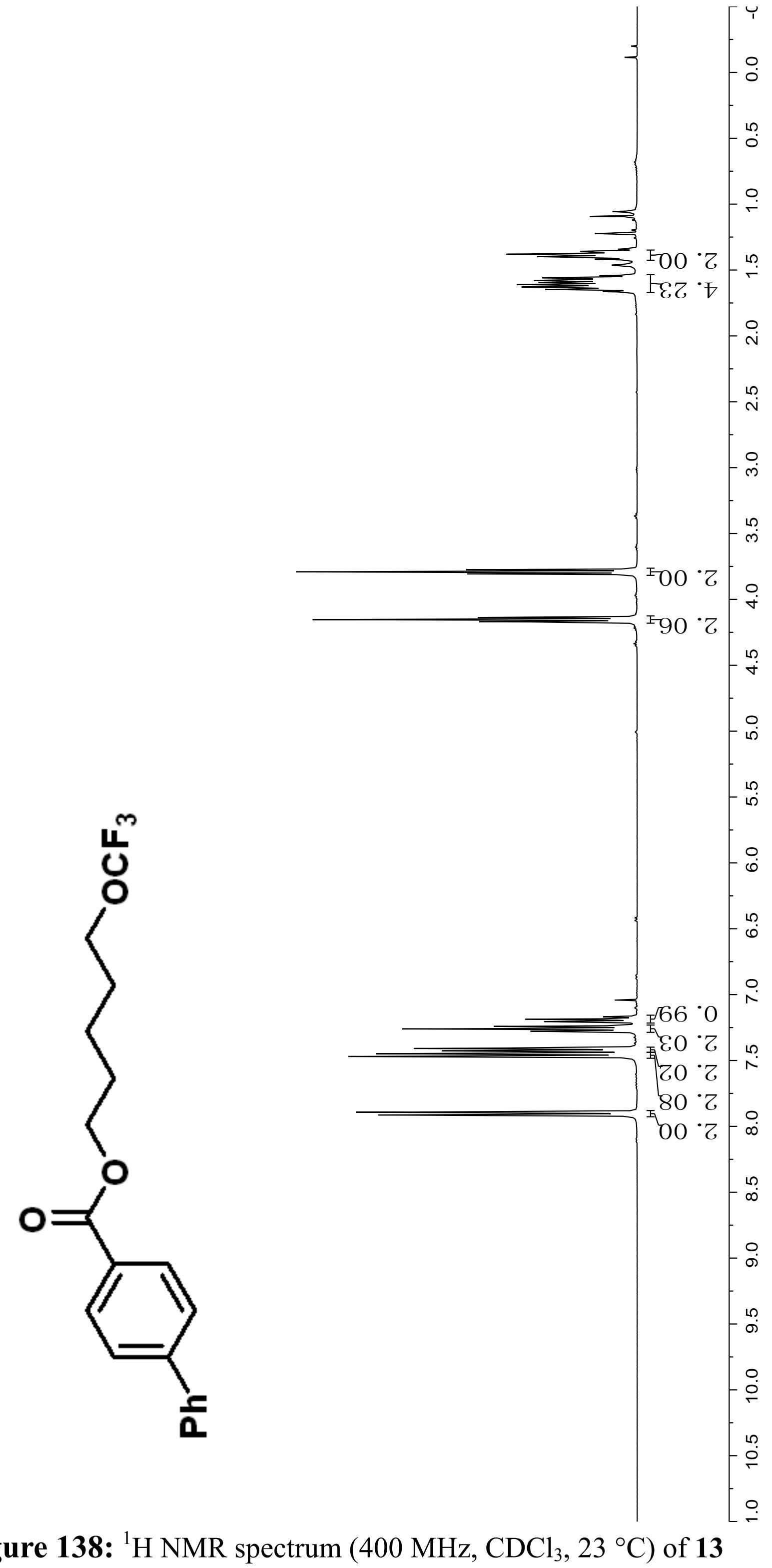

Supplementary Figure 138: ^{1}H NMR spectrum (400 MHz, $CDCl_3$, 23 °C) of **13**

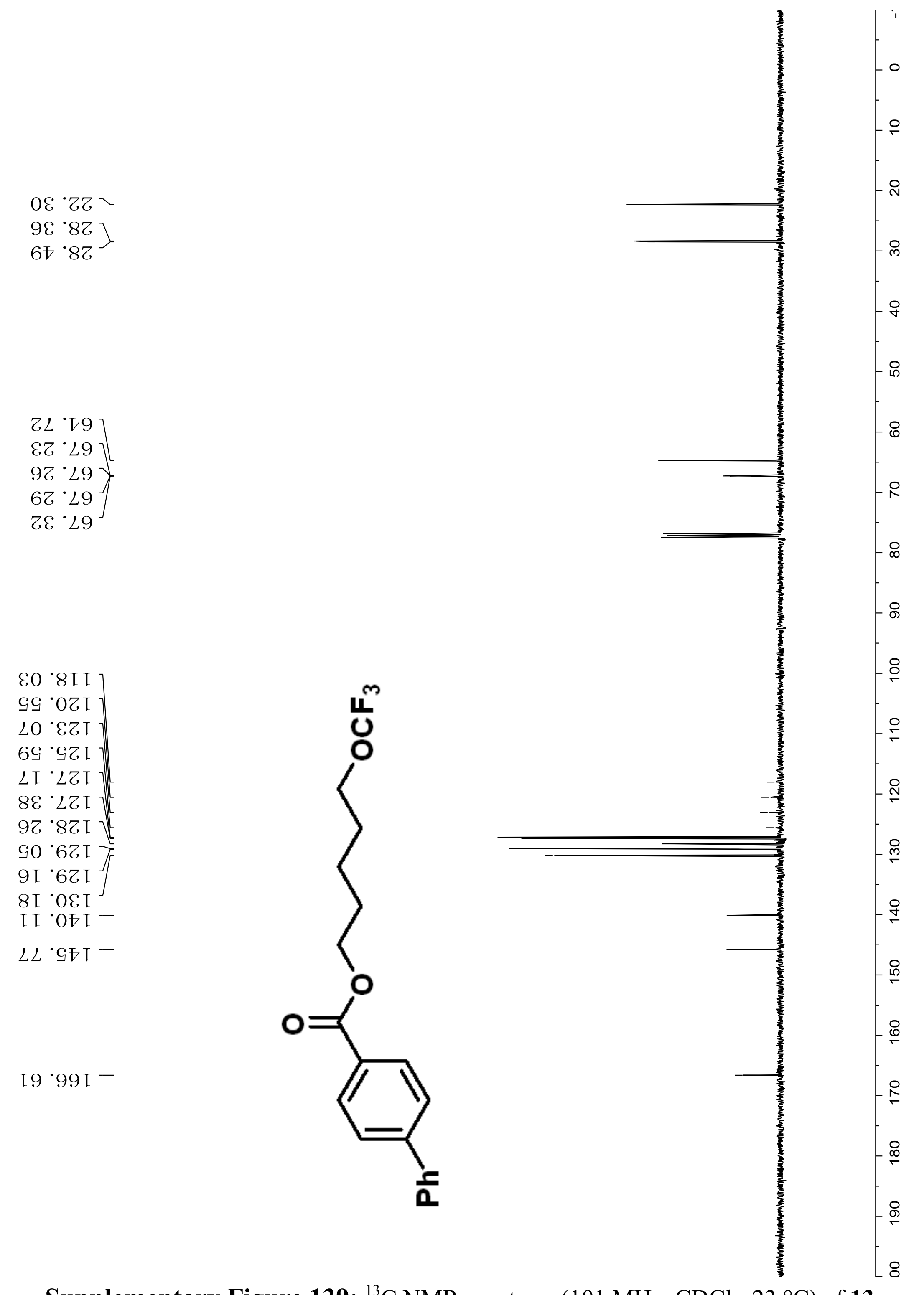

Supplementary Figure 139: ^{13}C NMR spectrum (101 MHz, $CDCl_3$, 23 °C) of **13**

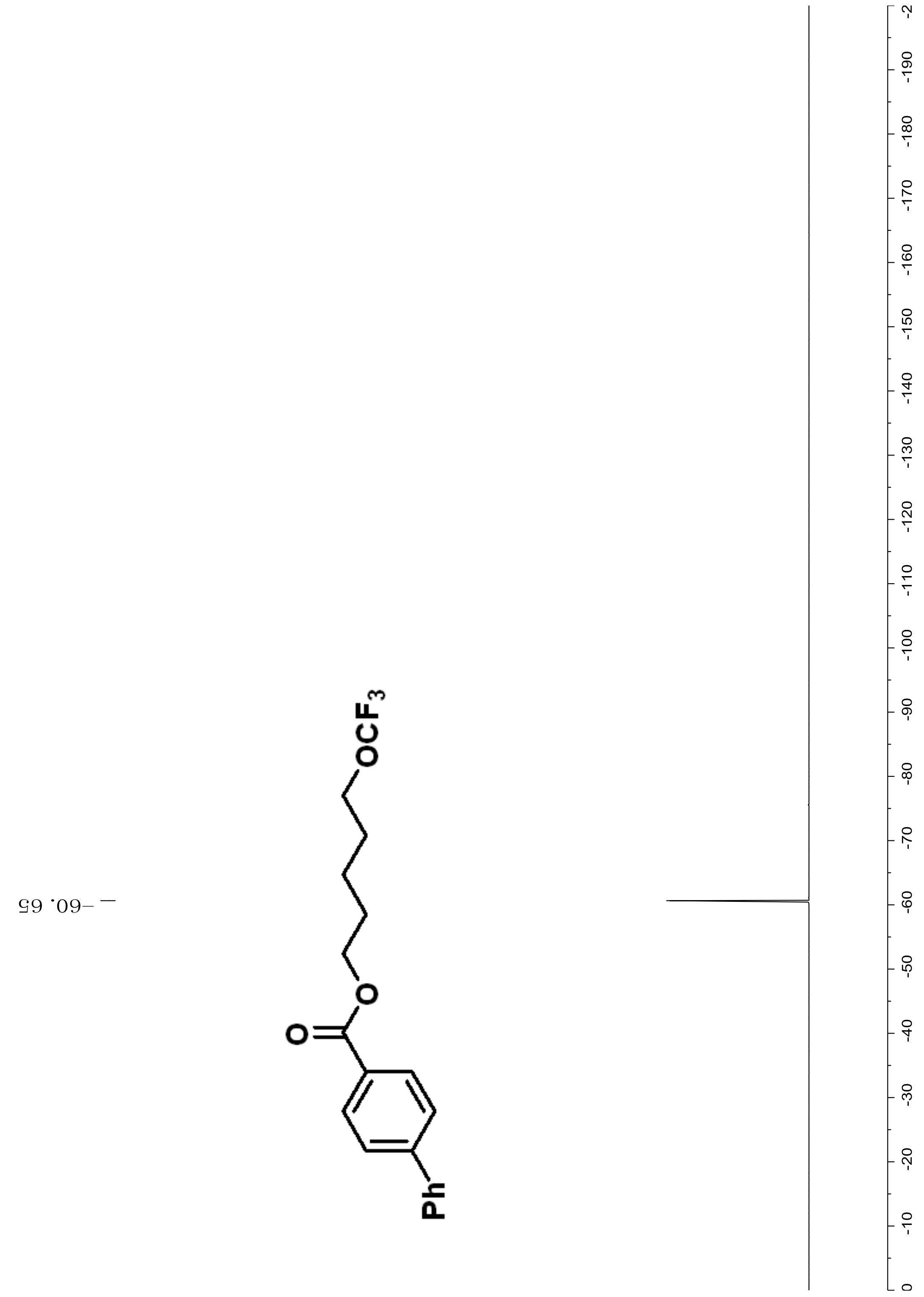

Supplementary Figure 140: ^{19}F NMR spectrum (376 MHz, $CDCl_3$, 23 °C) of **13**

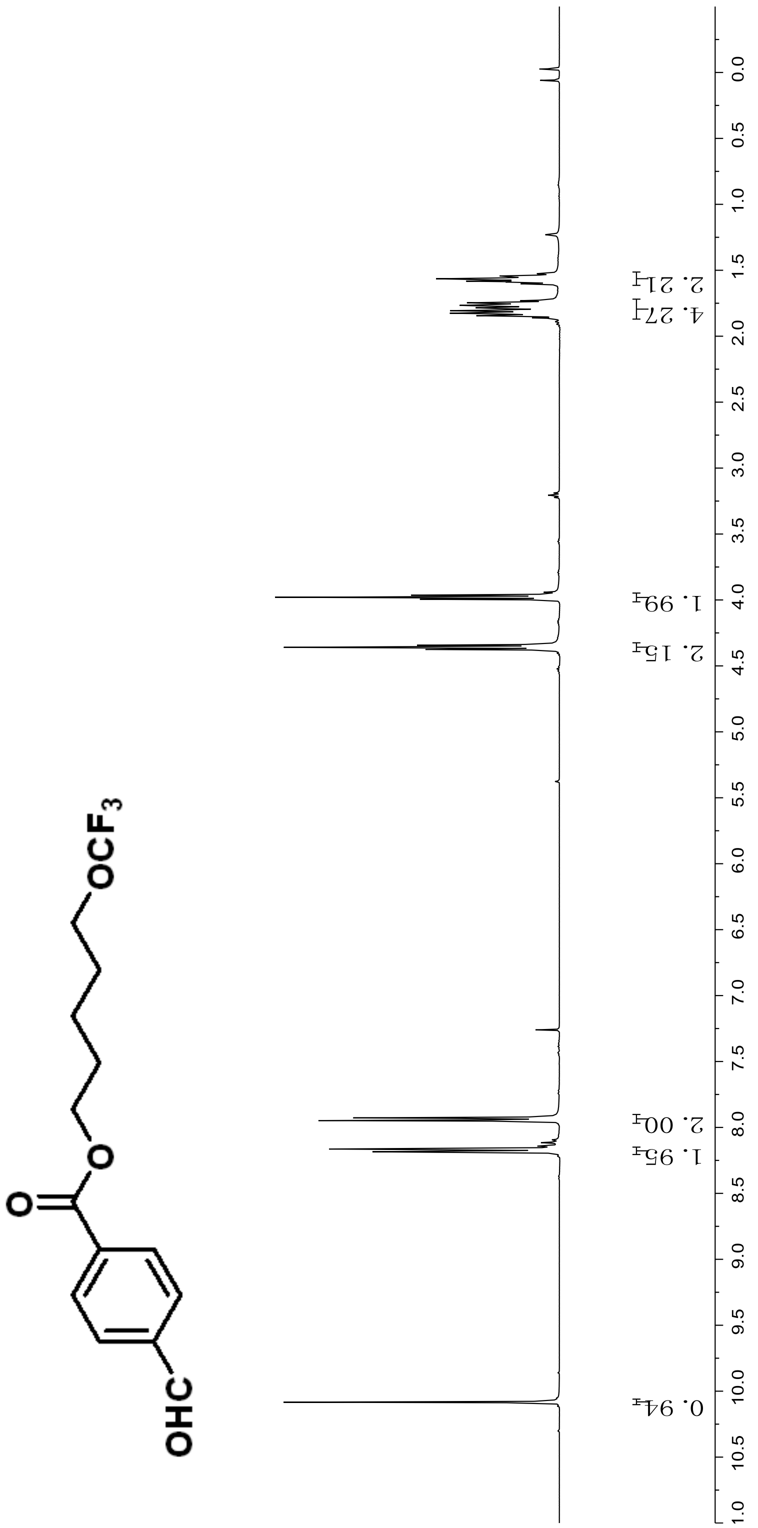

Supplementary Figure 141: ^{1}H NMR spectrum (400 MHz, $CDCl_3$, 23 °C) of **14**

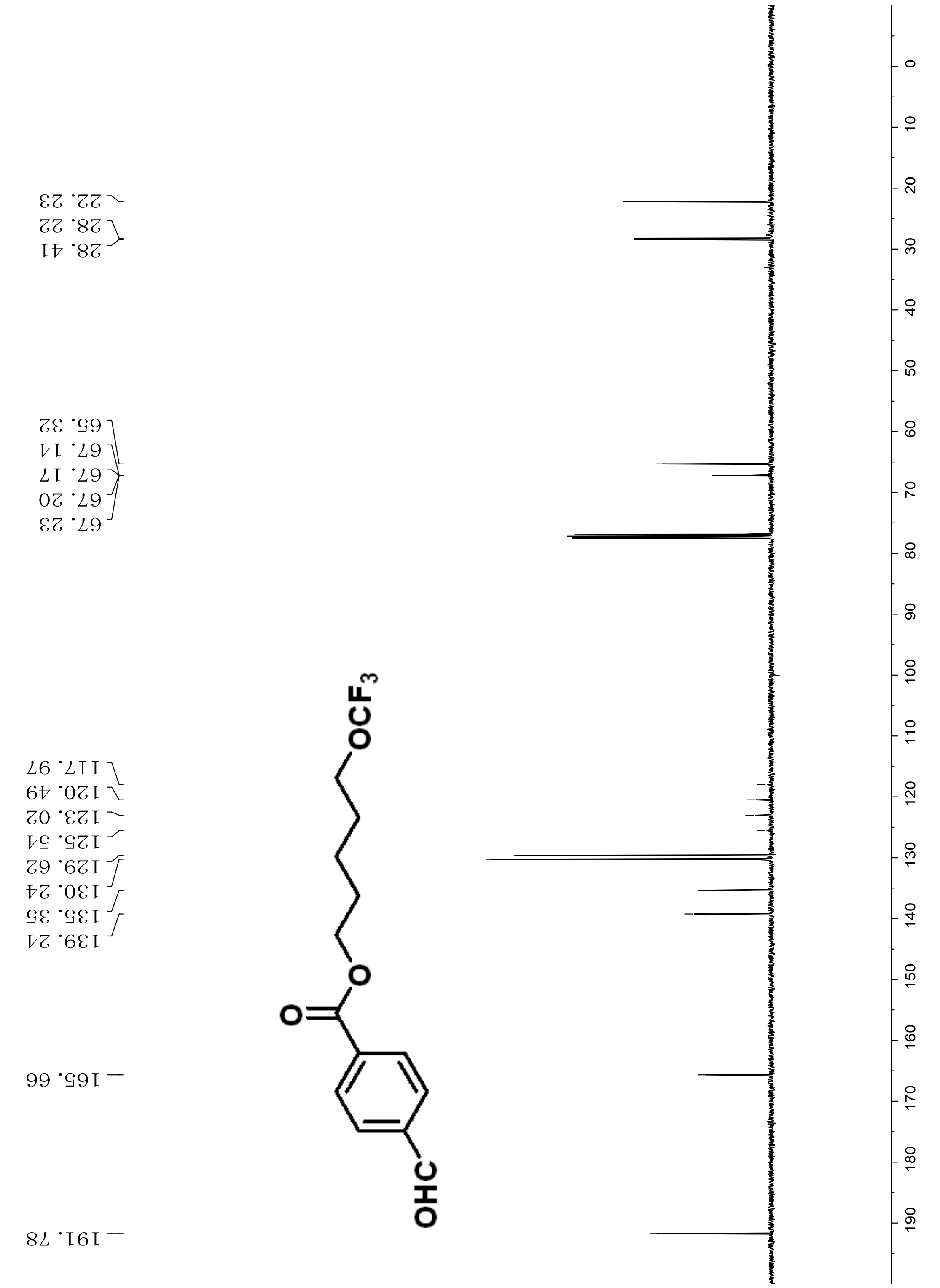

Supplementary Figure 142: ^{13}C NMR spectrum (101 MHz, $CDCl_3$, 23 °C) of **14**

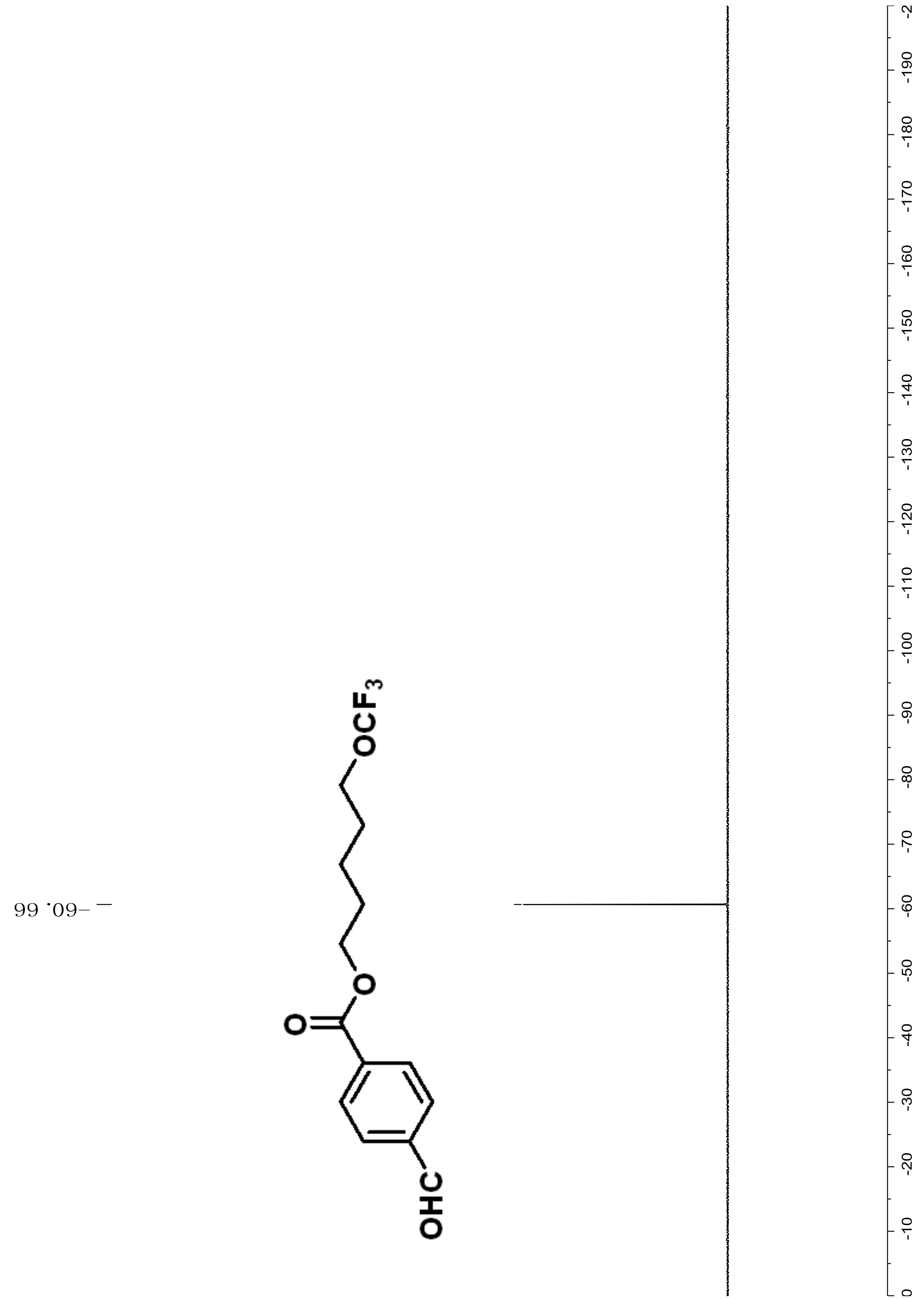

Supplementary Figure 143: ^{19}F NMR spectrum (376 MHz, $CDCl_3$, 23 °C) of **14**

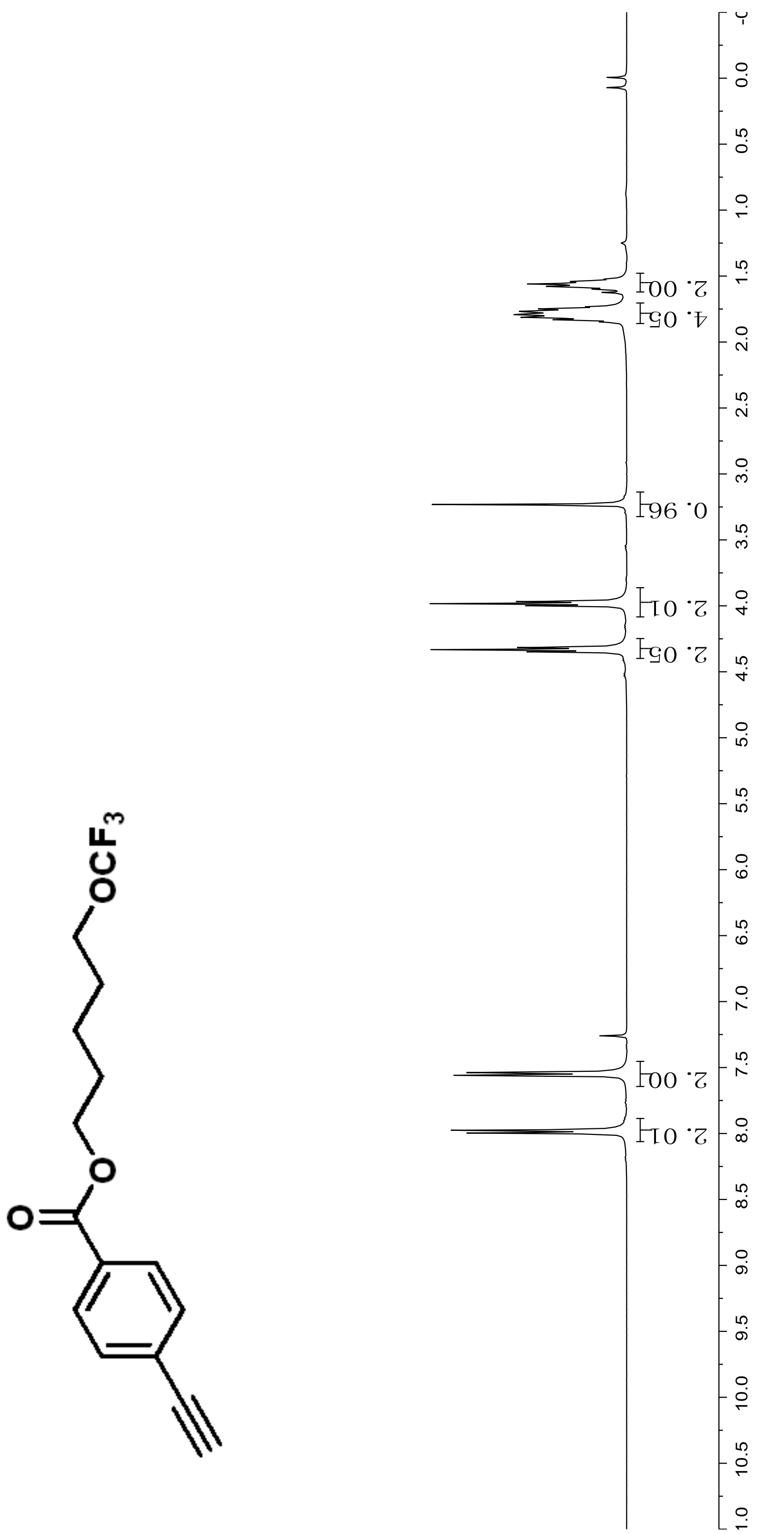

Supplementary Figure 144: ^{1}H NMR spectrum (400 MHz, $CDCl_3$, 23 °C) of **15**

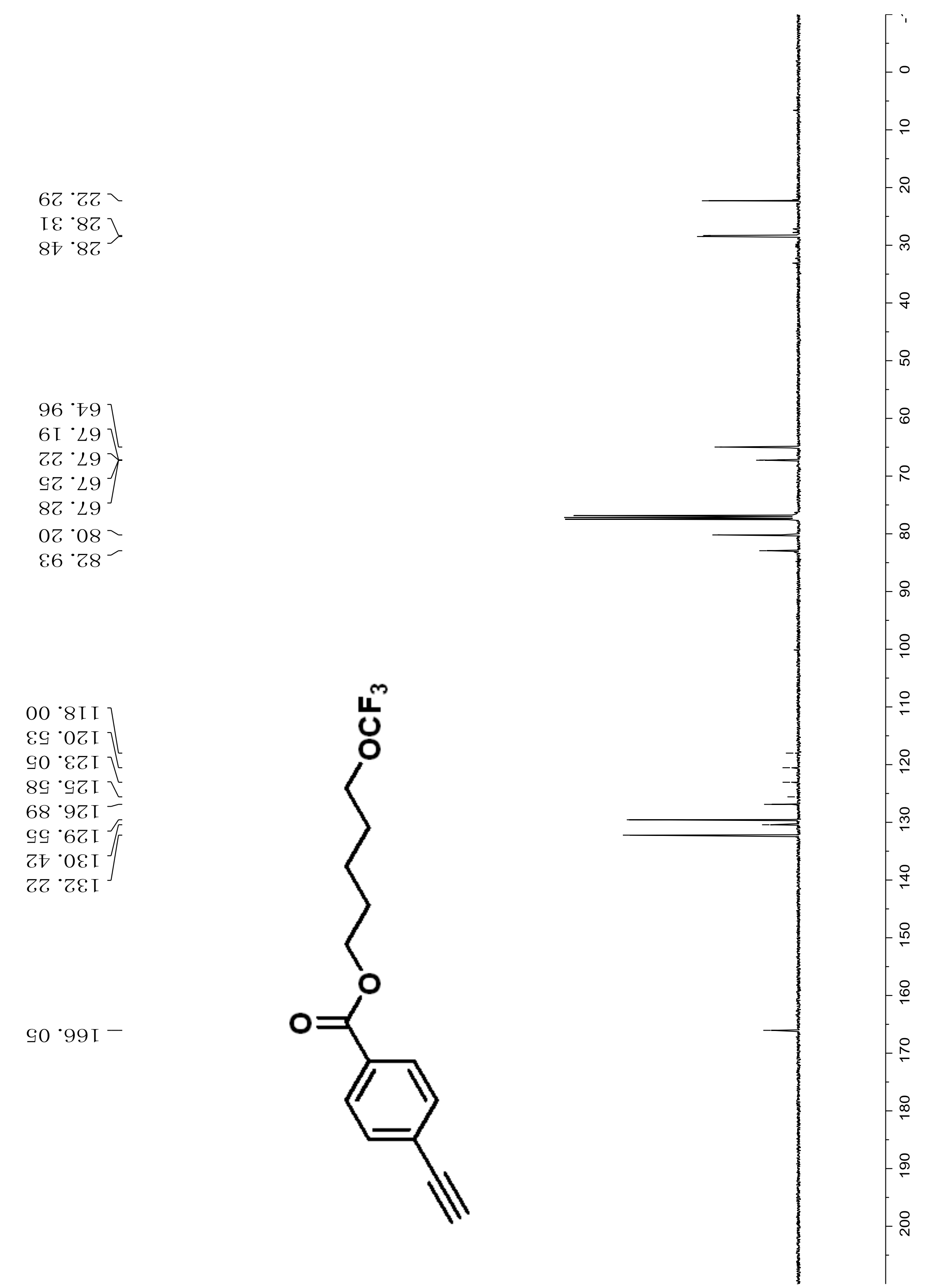

Supplementary Figure 145: ^{13}C NMR spectrum (101 MHz, $CDCl_3$, 23 °C) of **15**

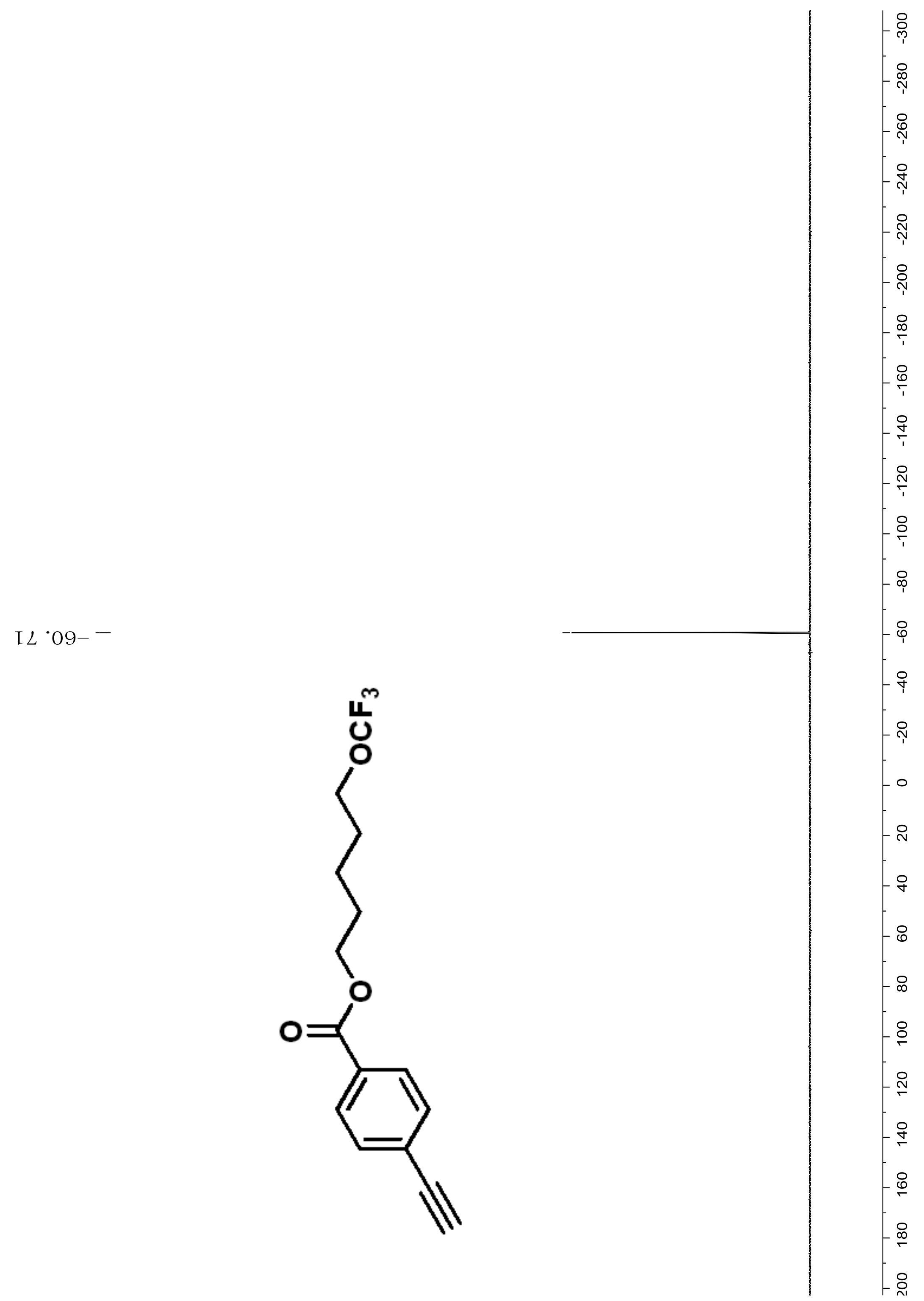

Supplementary Figure 146: ^{19}F NMR spectrum (376 MHz, $CDCl_3$, 23 °C) of **15**

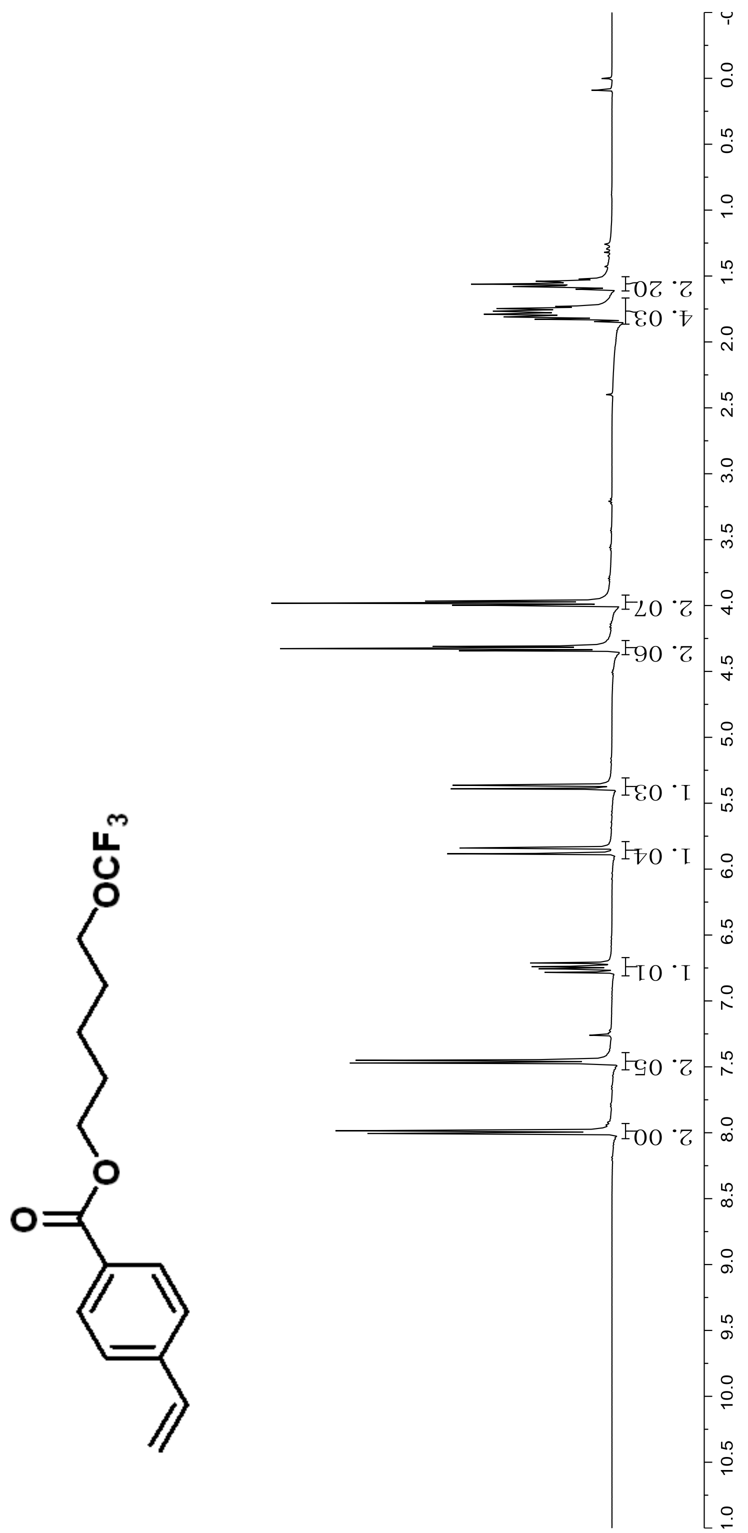

Supplementary Figure 147: ^{1}H NMR spectrum (400 MHz, $CDCl_3$, 23 °C) of **16**

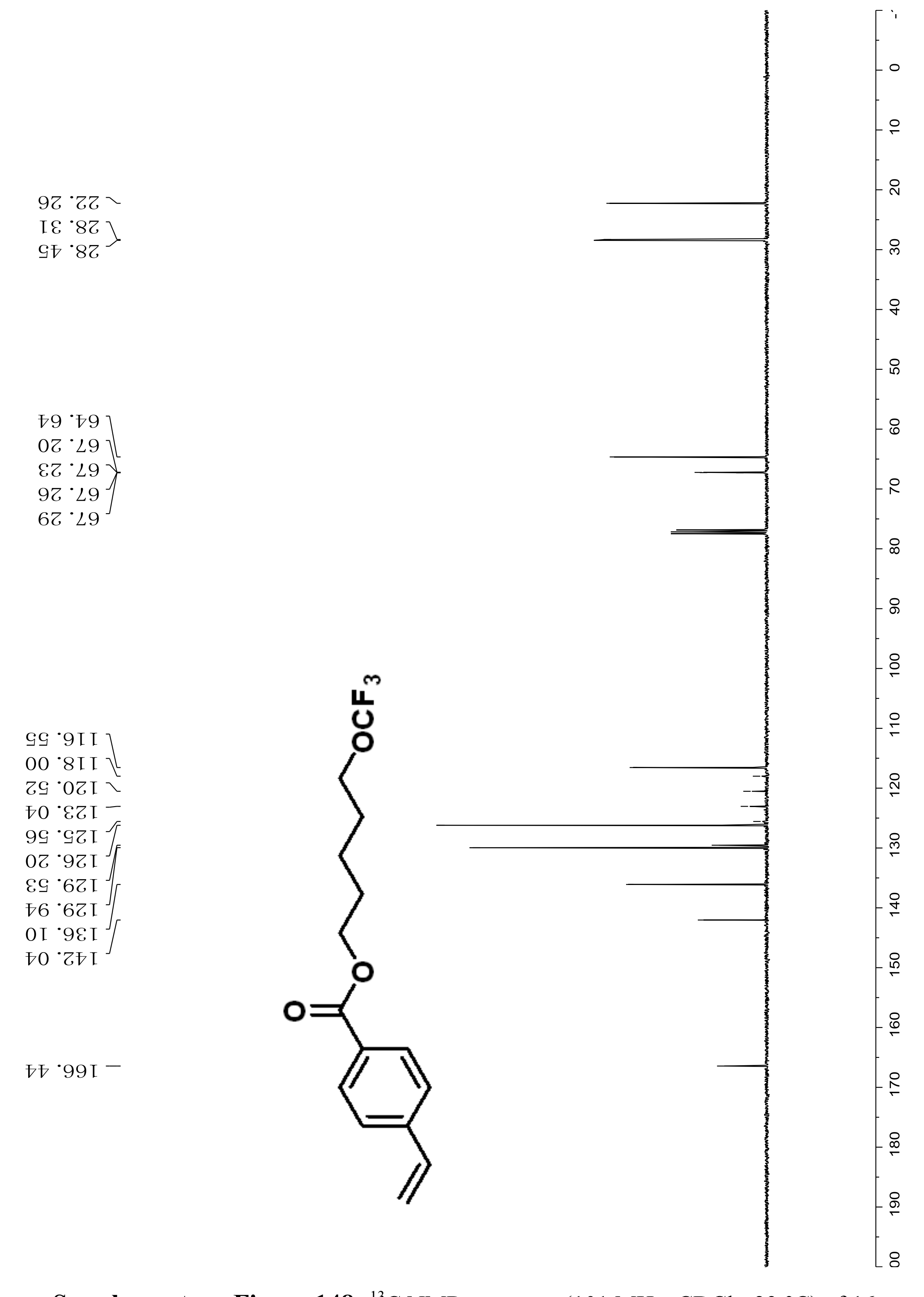

Supplementary Figure 148: ^{13}C NMR spectrum (101 MHz, $CDCl_3$, 23 °C) of **16**

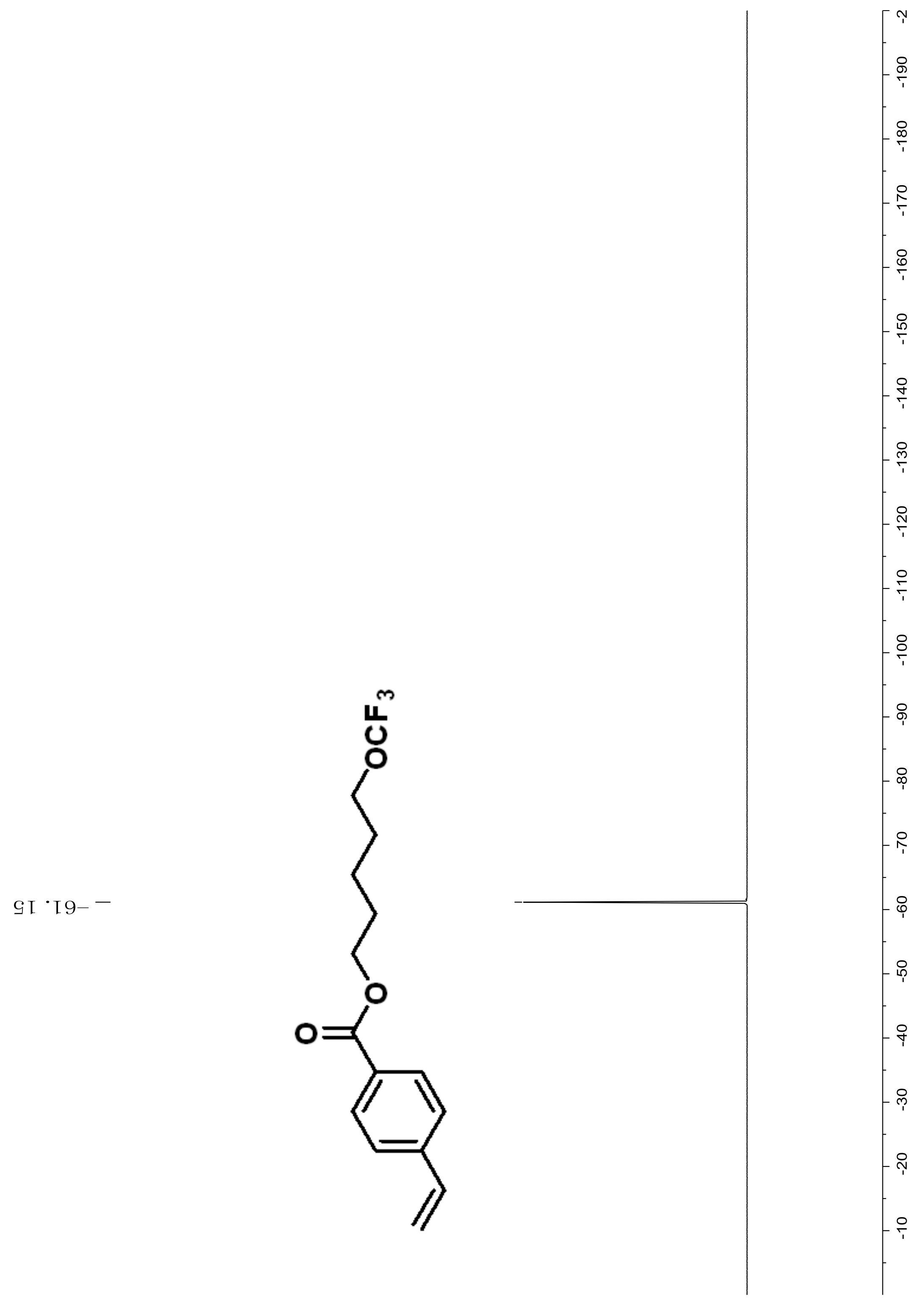

Supplementary Figure 149: ^{19}F NMR spectrum (376 MHz, DMSO, 23 °C) of **16**

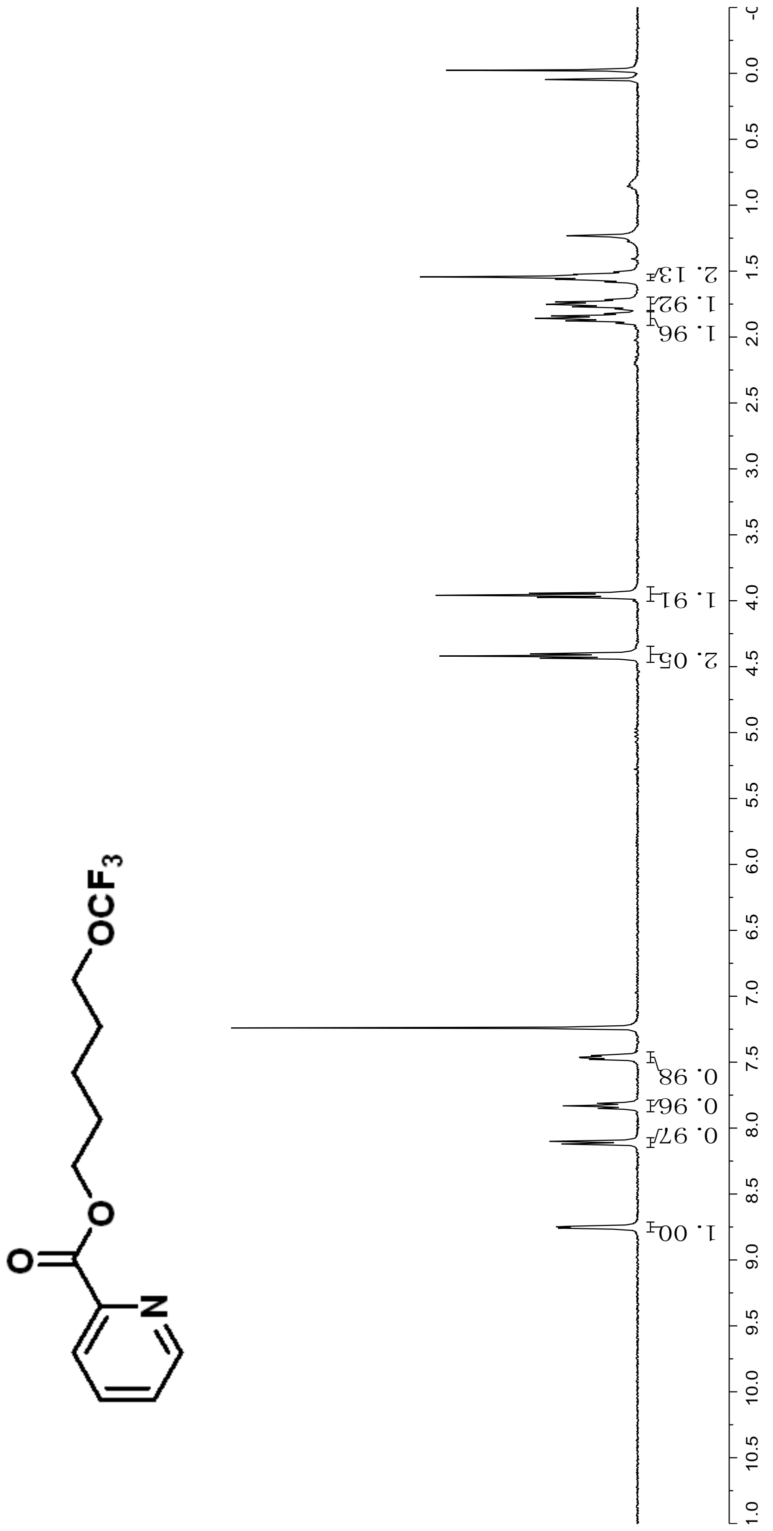

Supplementary Figure 150: ^{1}H NMR spectrum (400 MHz, $CDCl_3$, 23 °C) of **17**

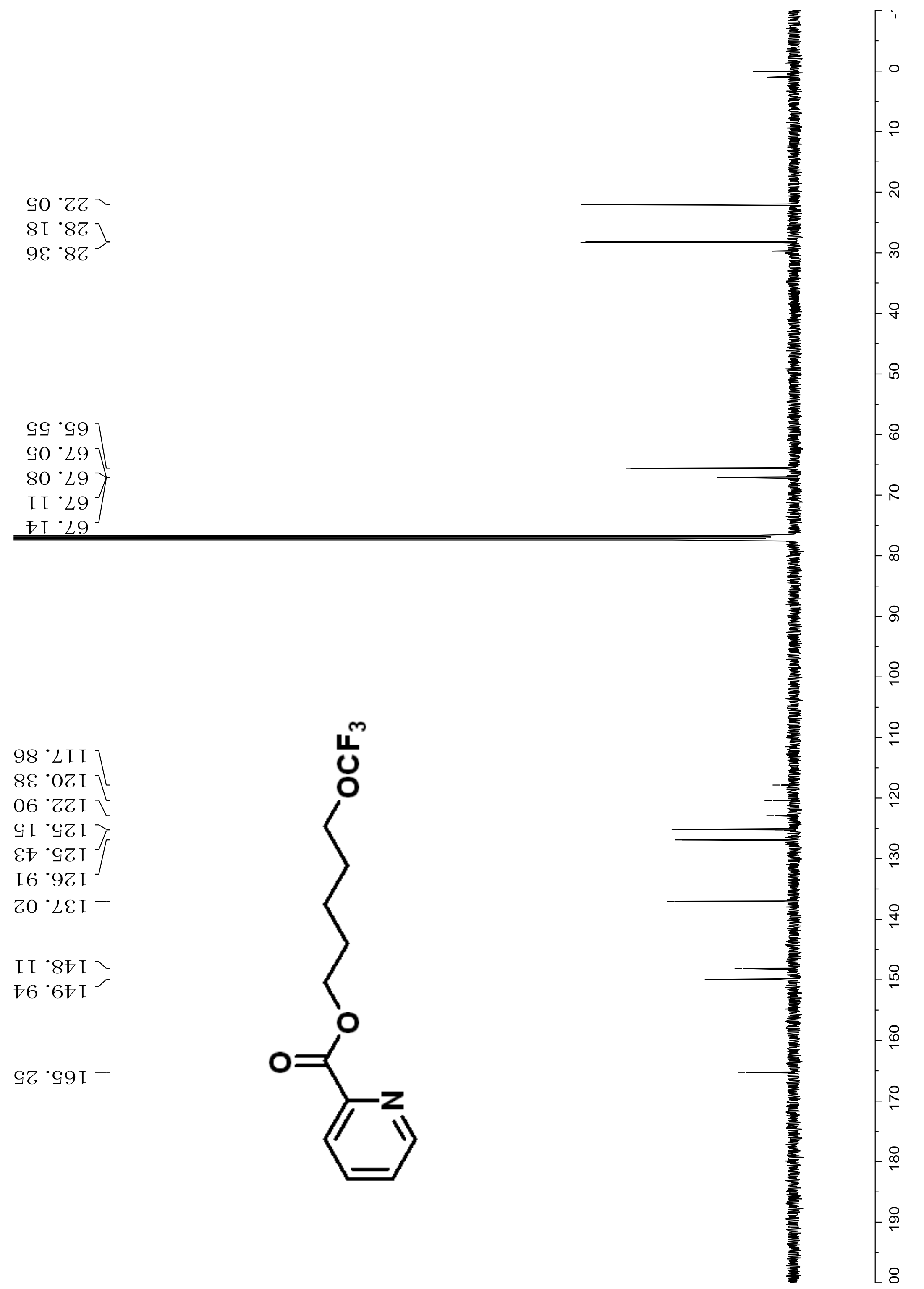

Supplementary Figure 151: ^{13}C NMR spectrum (101 MHz, $CDCl_3$, 23 °C) of **17**

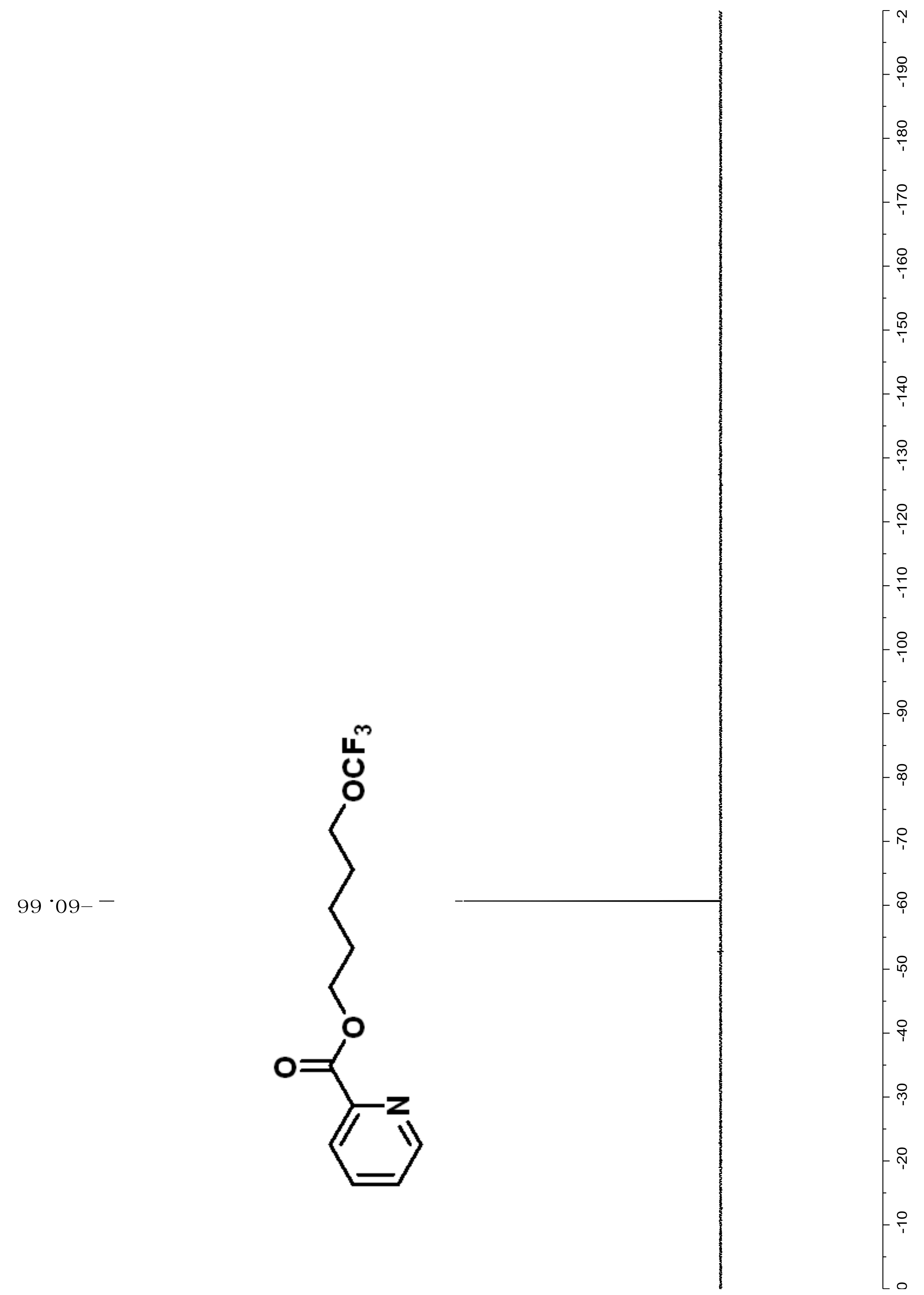

Supplementary Figure 152: ^{19}F NMR spectrum (376 MHz, $CDCl_3$, 23 °C) of **17**

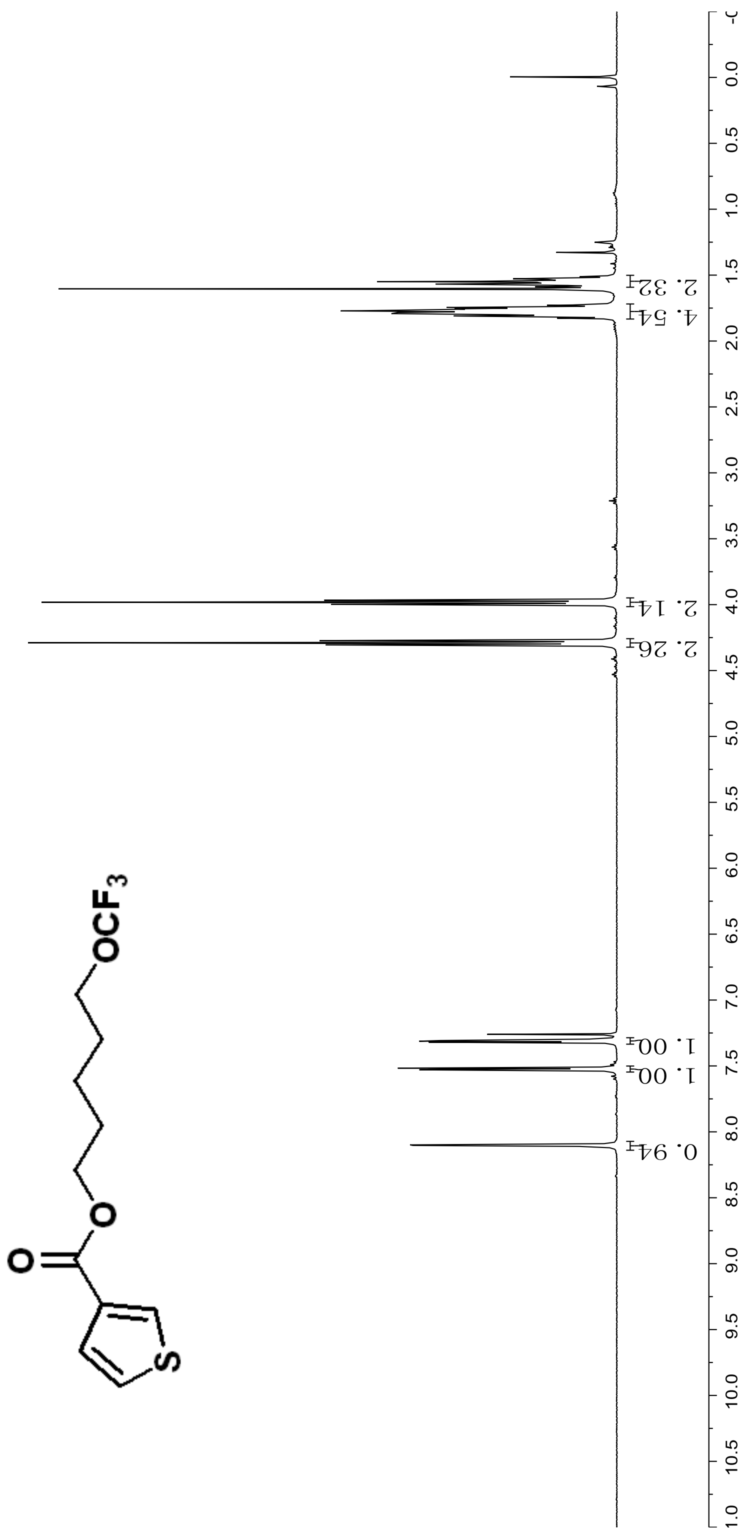

Supplementary Figure 153: ^{1}H NMR spectrum (400 MHz, $CDCl_3$, 23 °C) of **18**

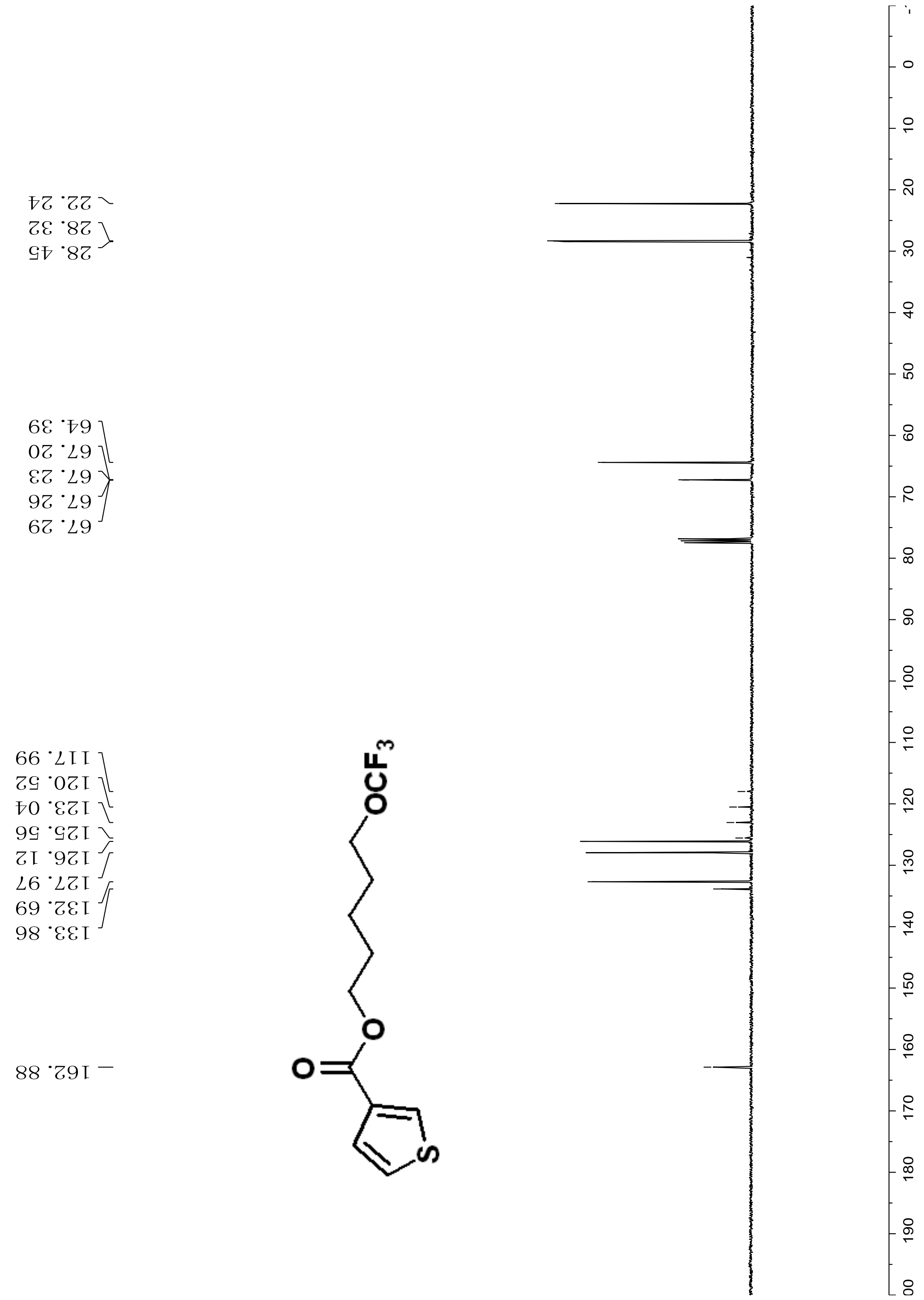

Supplementary Figure 154: ^{13}C NMR spectrum (101 MHz, $CDCl_3$, 23 °C) of **18**

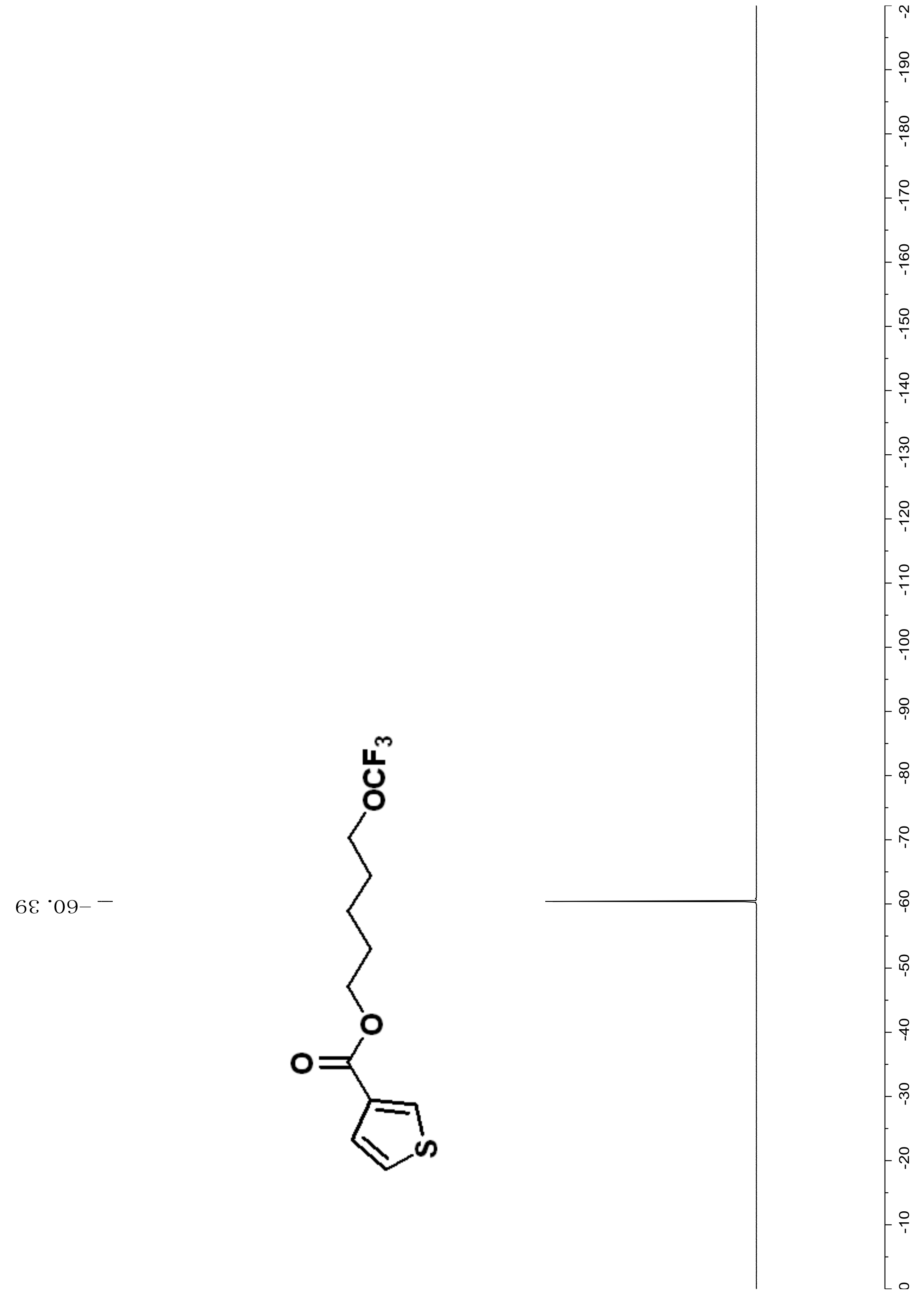

Supplementary Figure 155: ^{19}F NMR spectrum (376 MHz, $CDCl_3$, 23 °C) of **18**

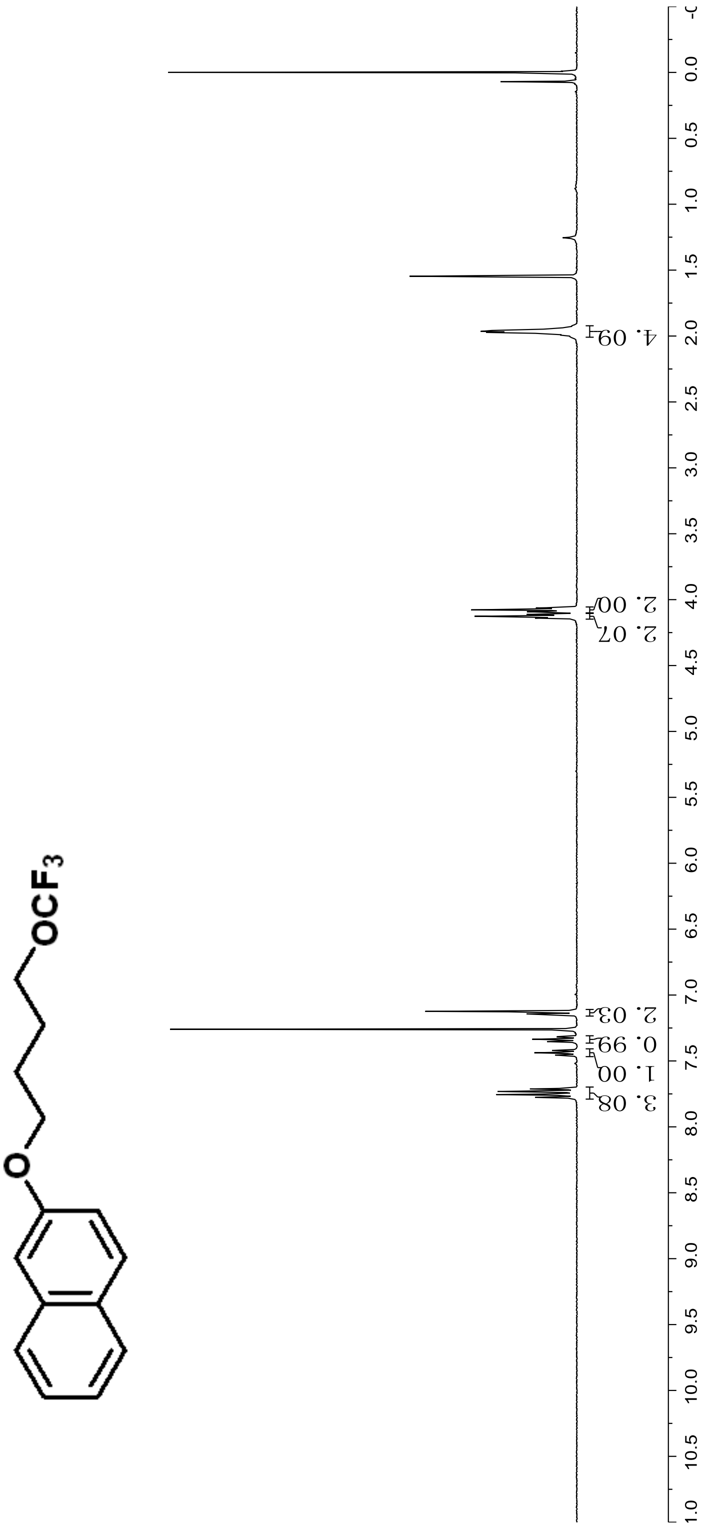

Supplementary Figure 156: ^{1}H NMR spectrum (400 MHz, $CDCl_3$, 23 °C) of **19**

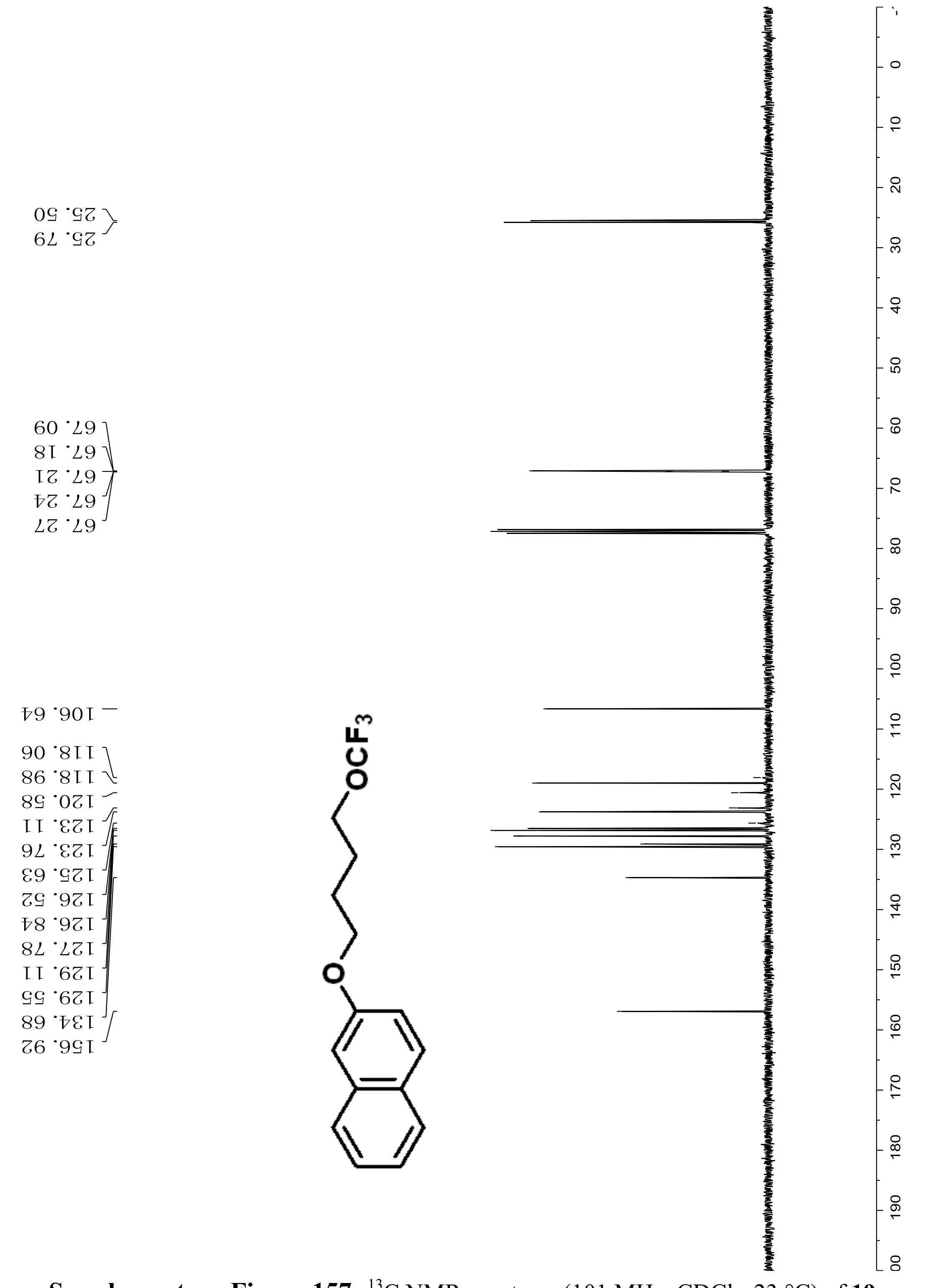

Supplementary Figure 157: ^{13}C NMR spectrum (101 MHz, $CDCl_3$, 23 °C) of **19**

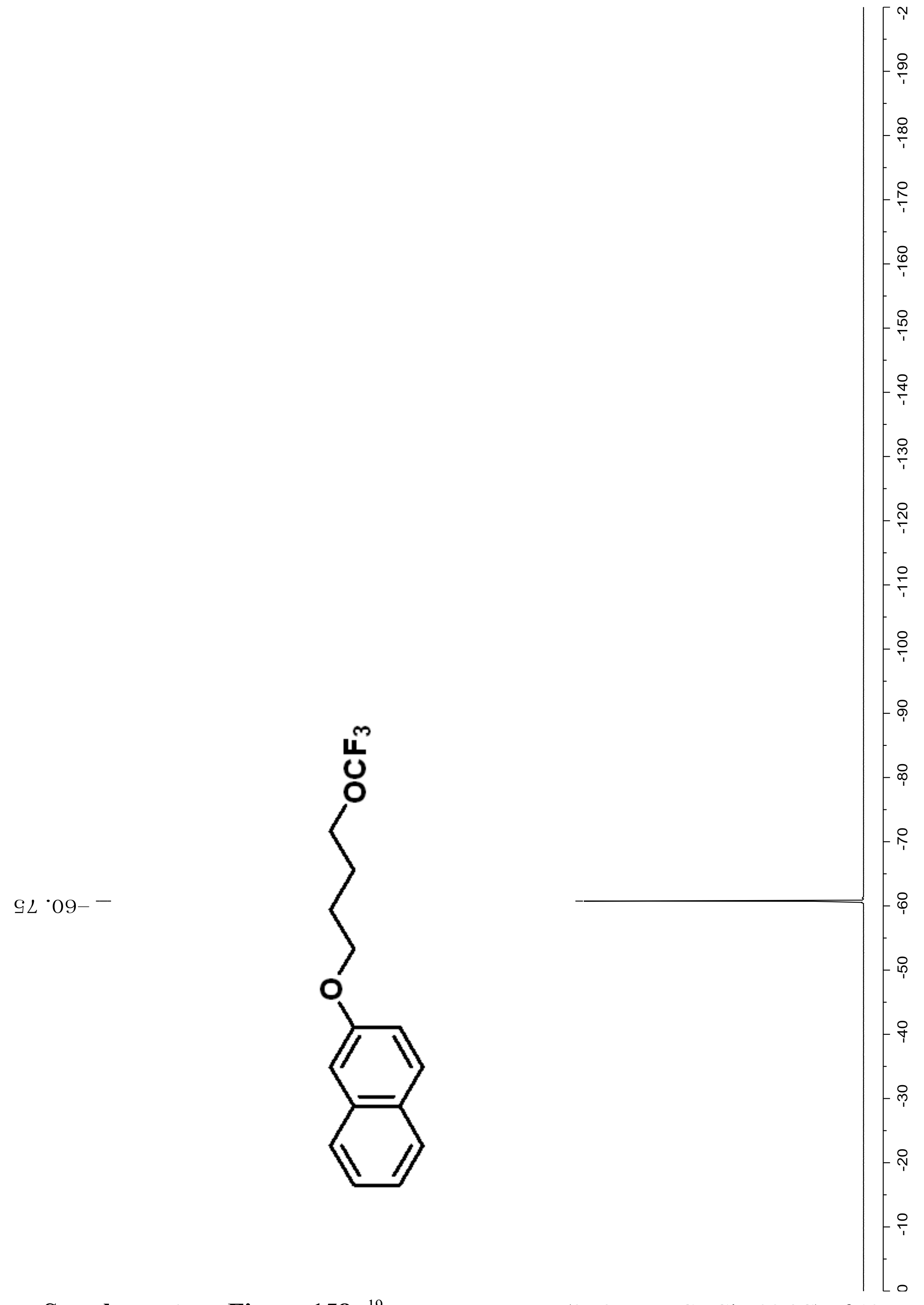

Supplementary Figure 158: ^{19}F NMR spectrum (376 MHz, $CDCl_3$, 23 °C) of **19**

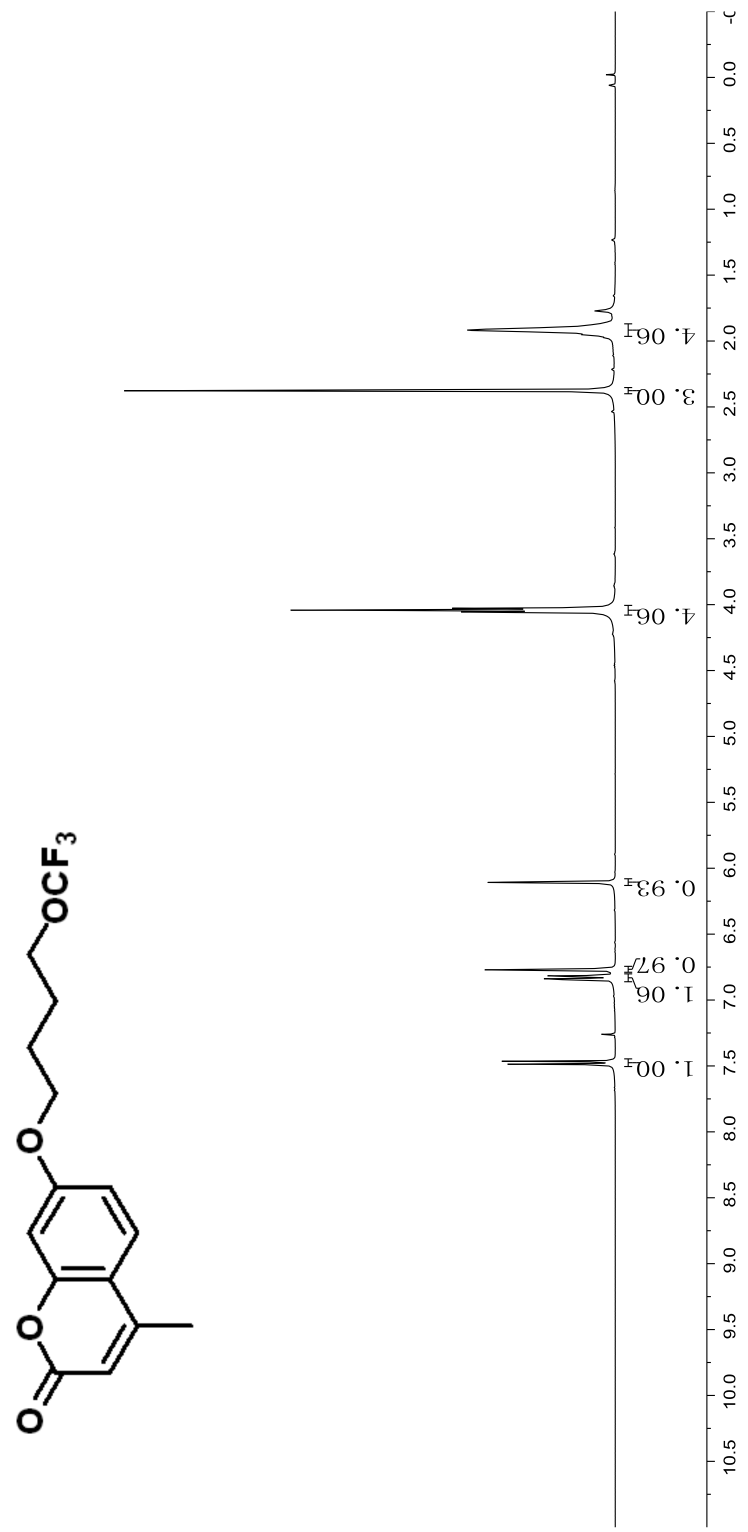

Supplementary Figure 159: ^{1}H NMR spectrum (400 MHz, $CDCl_3$, 23 °C) of **20**

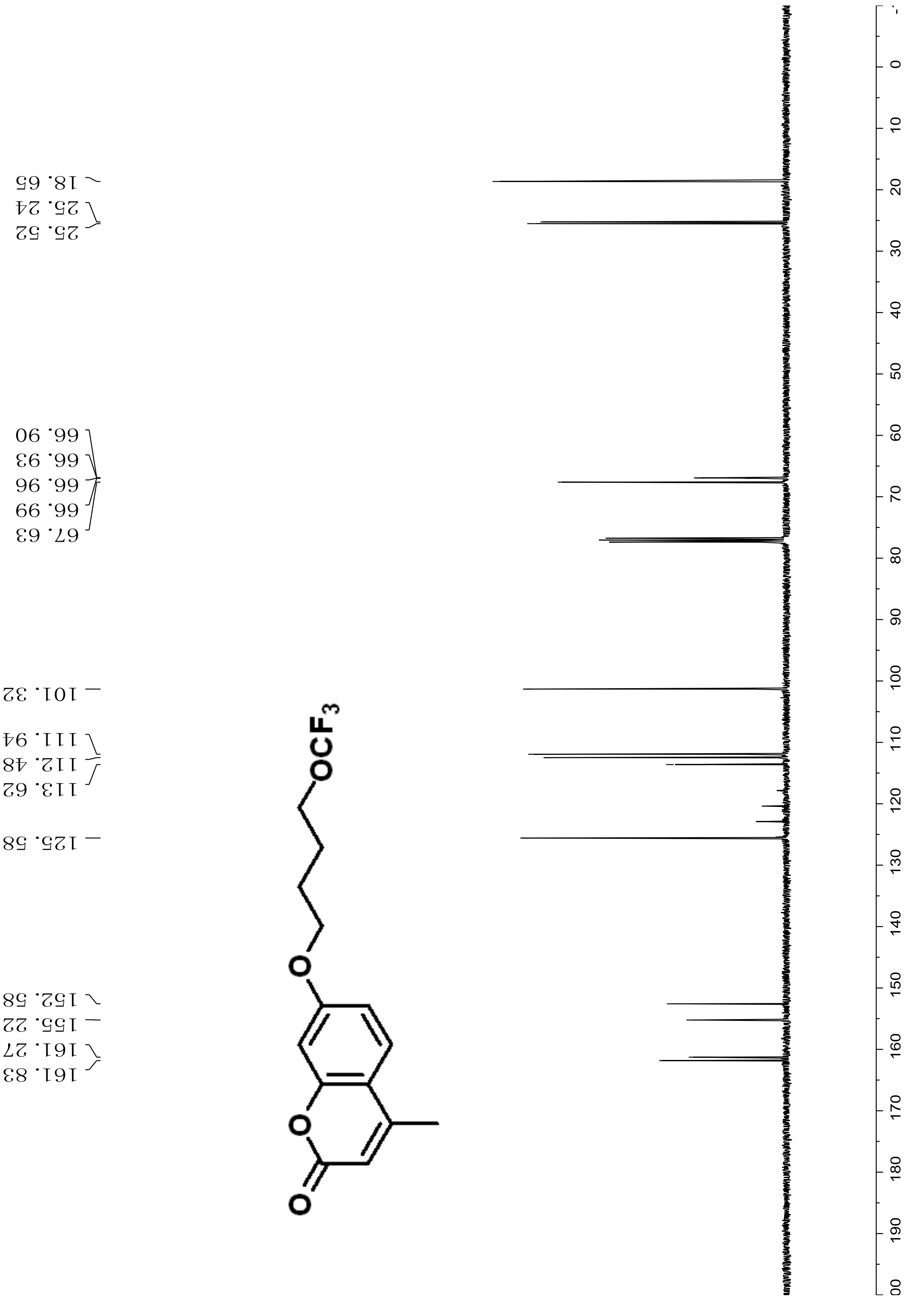

Supplementary Figure 160: ^{13}C NMR spectrum (101 MHz, $CDCl_3$, 23 °C) of **20**

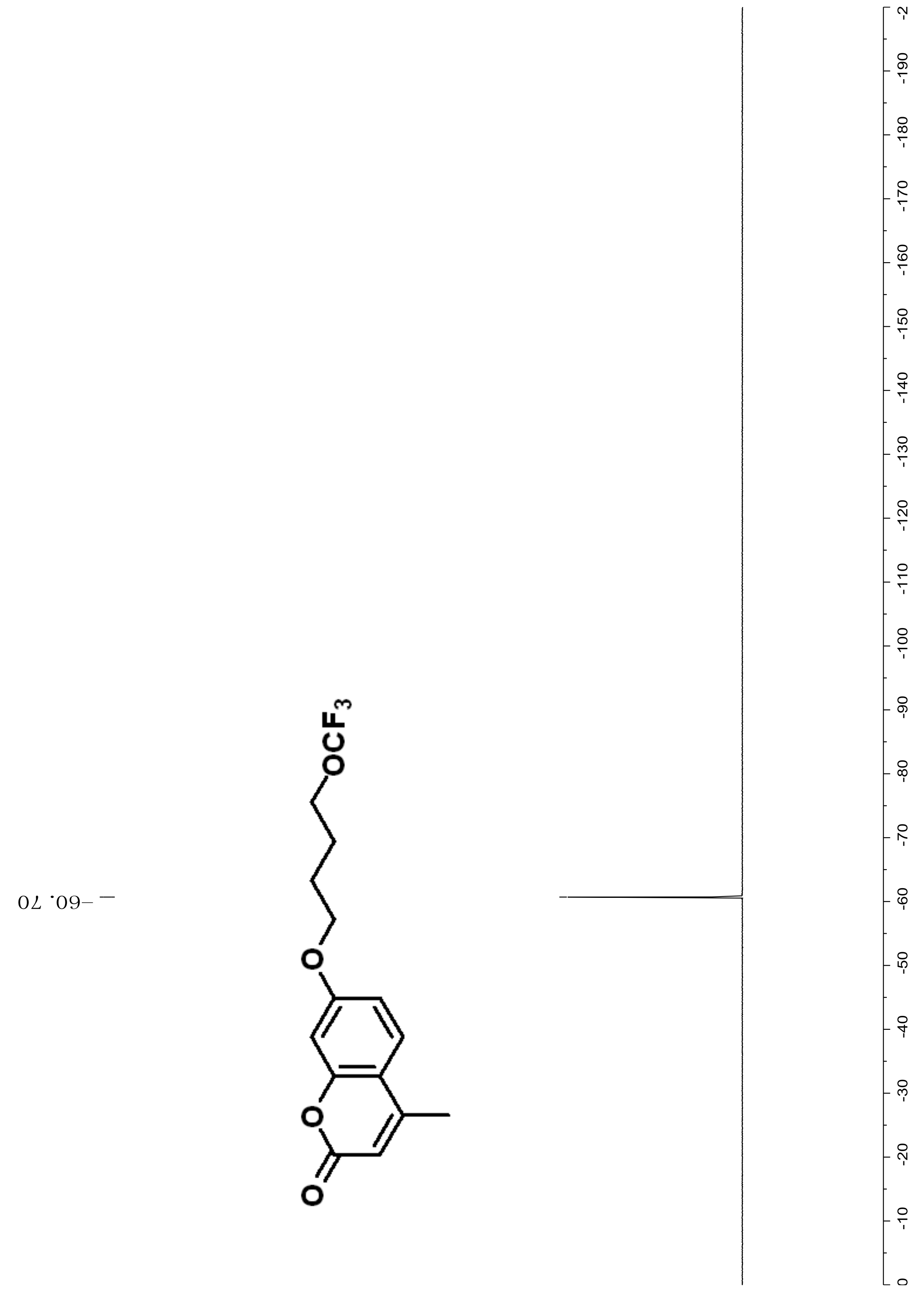

Supplementary Figure 161: ^{19}F NMR spectrum (376 MHz, $CDCl_3$, 23 °C) of **20**

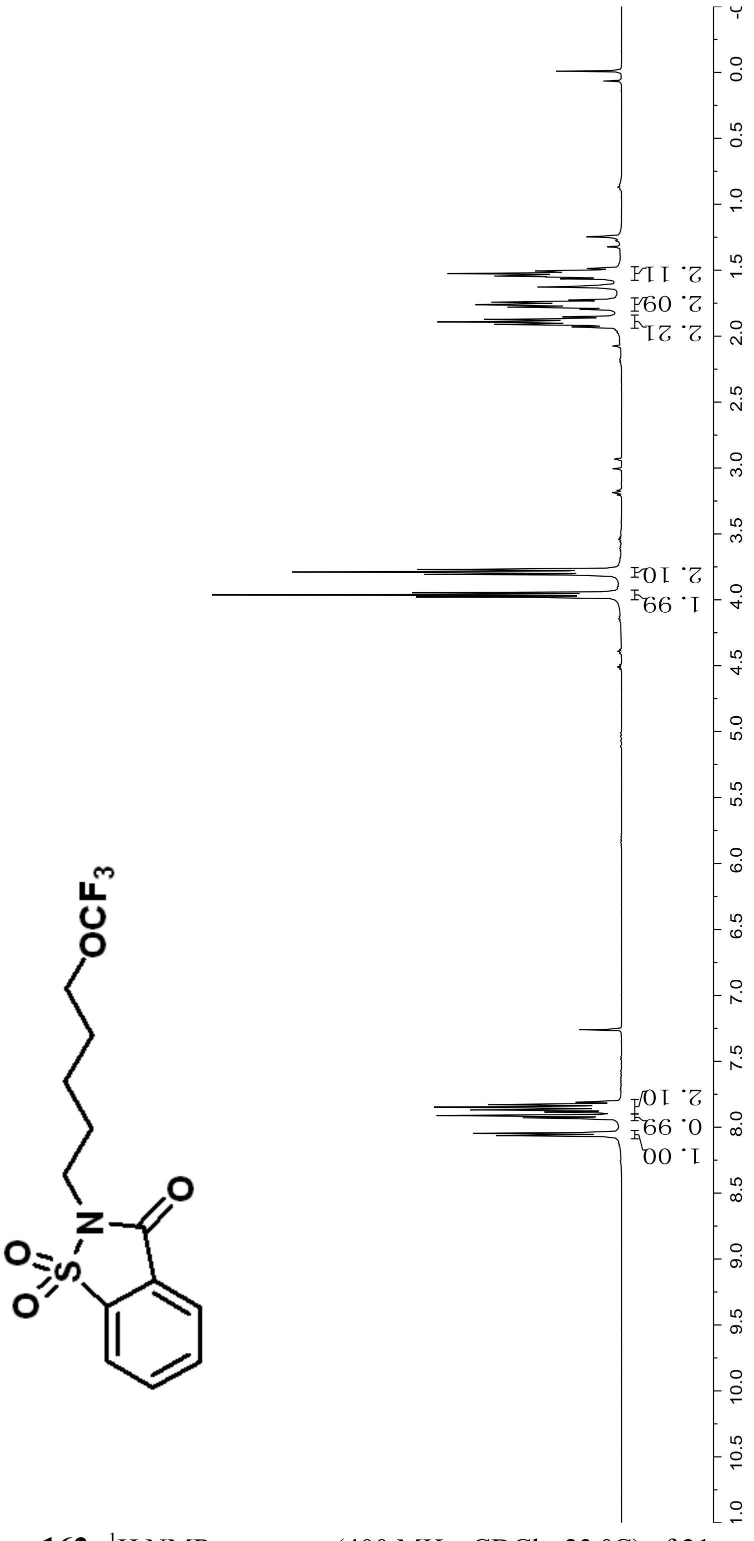

Supplementary Figure 162: ^{1}H NMR spectrum (400 MHz, $CDCl_3$, 23 °C) of **21**

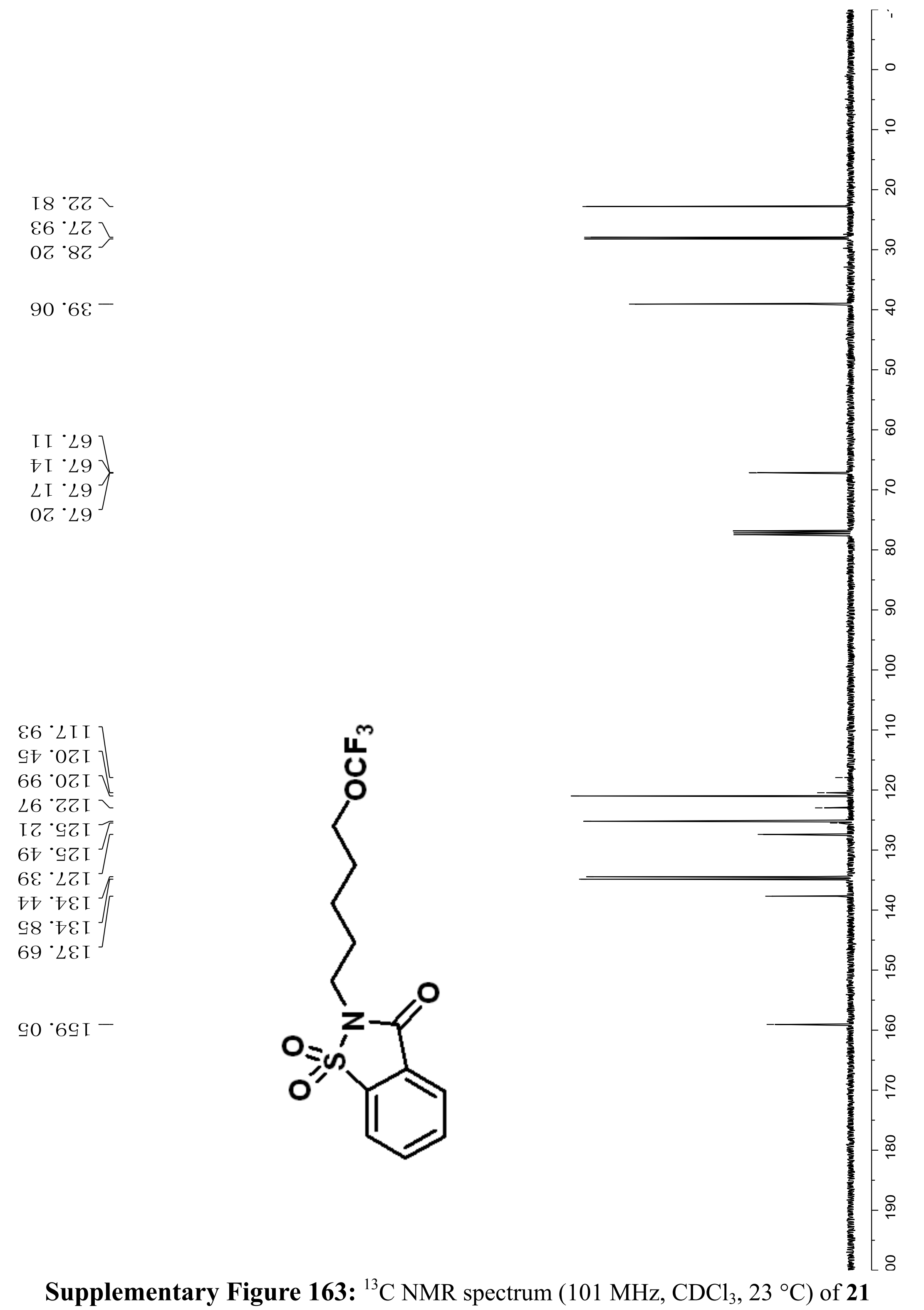

Supplementary Figure 163: ^{13}C NMR spectrum (101 MHz, $CDCl_3$, 23 °C) of **21**

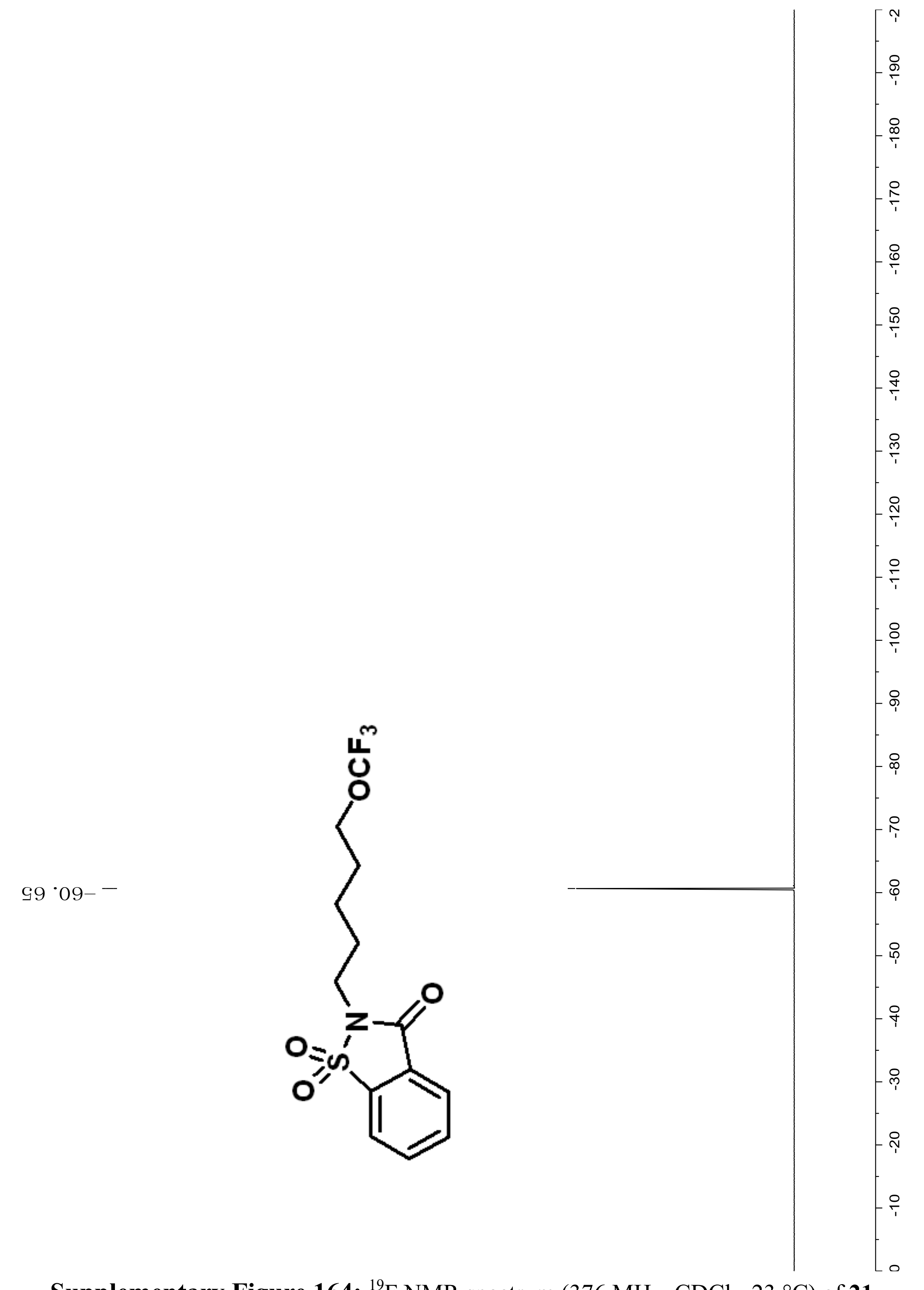

Supplementary Figure 164: ^{19}F NMR spectrum (376 MHz, $CDCl_3$, 23 °C) of **21**

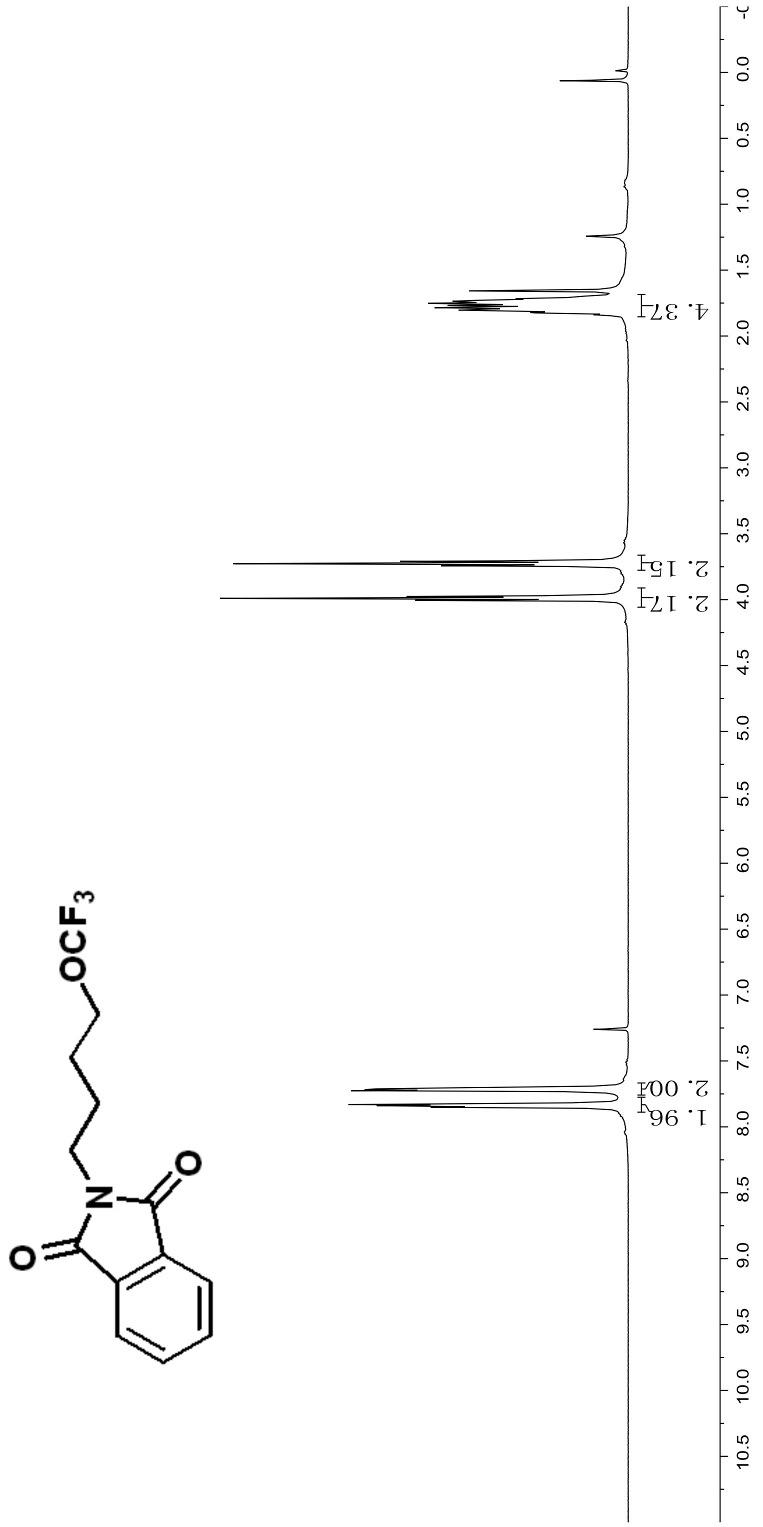

Supplementary Figure 165: ^{1}H NMR spectrum (400 MHz, $CDCl_3$, 23 °C) of **22**

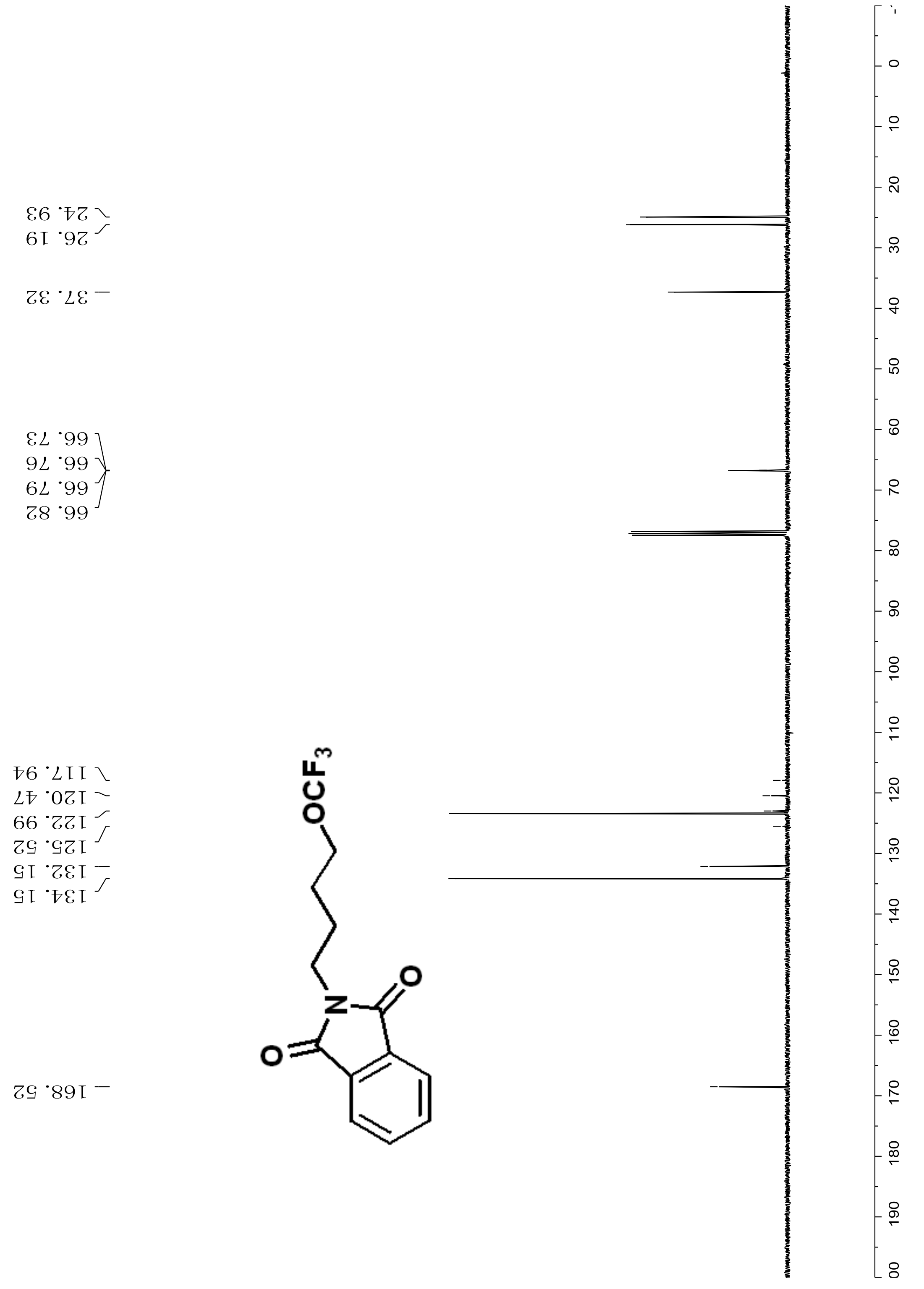

Supplementary Figure 166: ^{13}C NMR spectrum (101 MHz, $CDCl_3$, 23 °C) of **22**

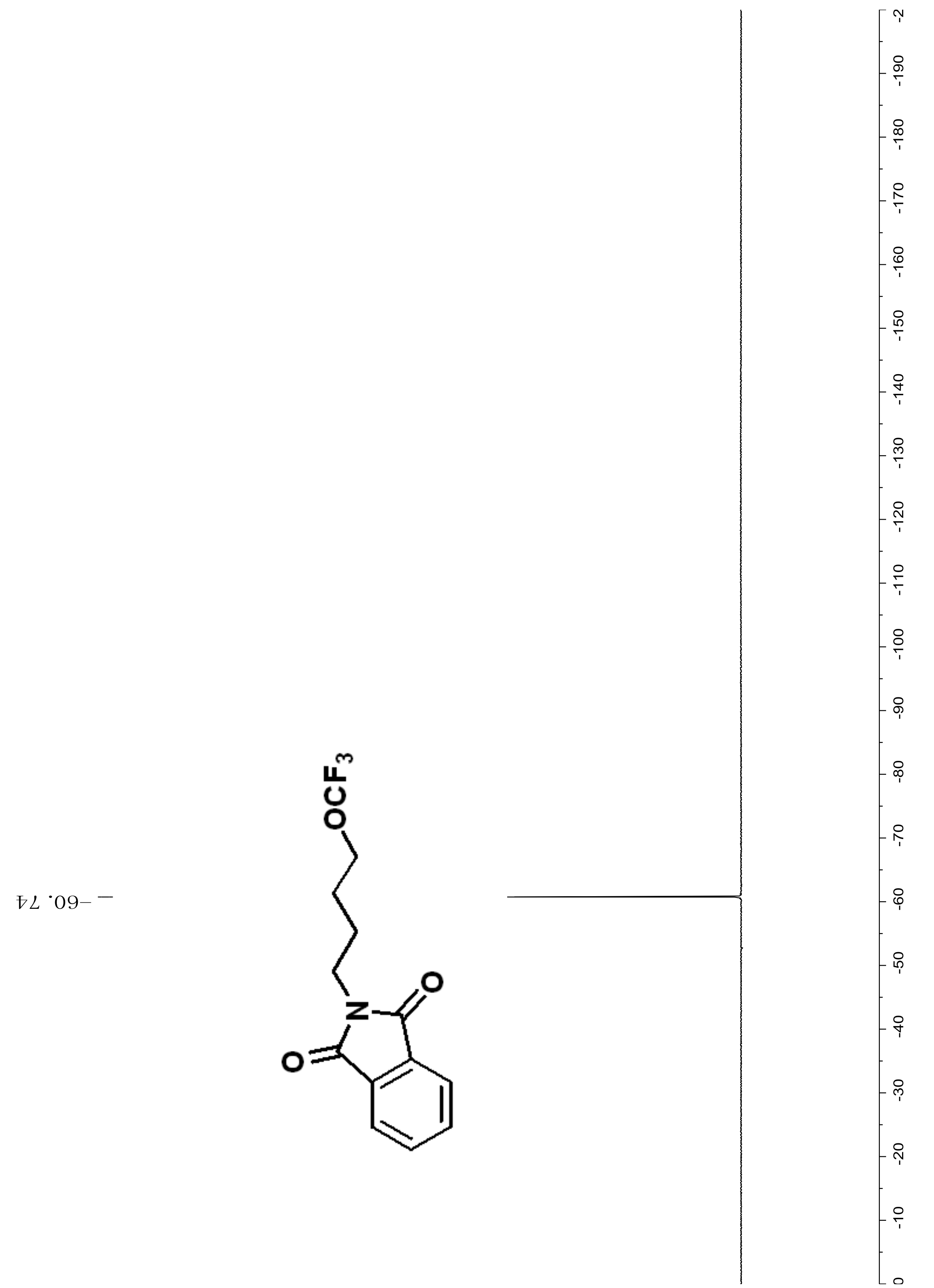

Supplementary Figure 167: ^{19}F NMR spectrum (376 MHz, $CDCl_3$, 23 °C) of **22**

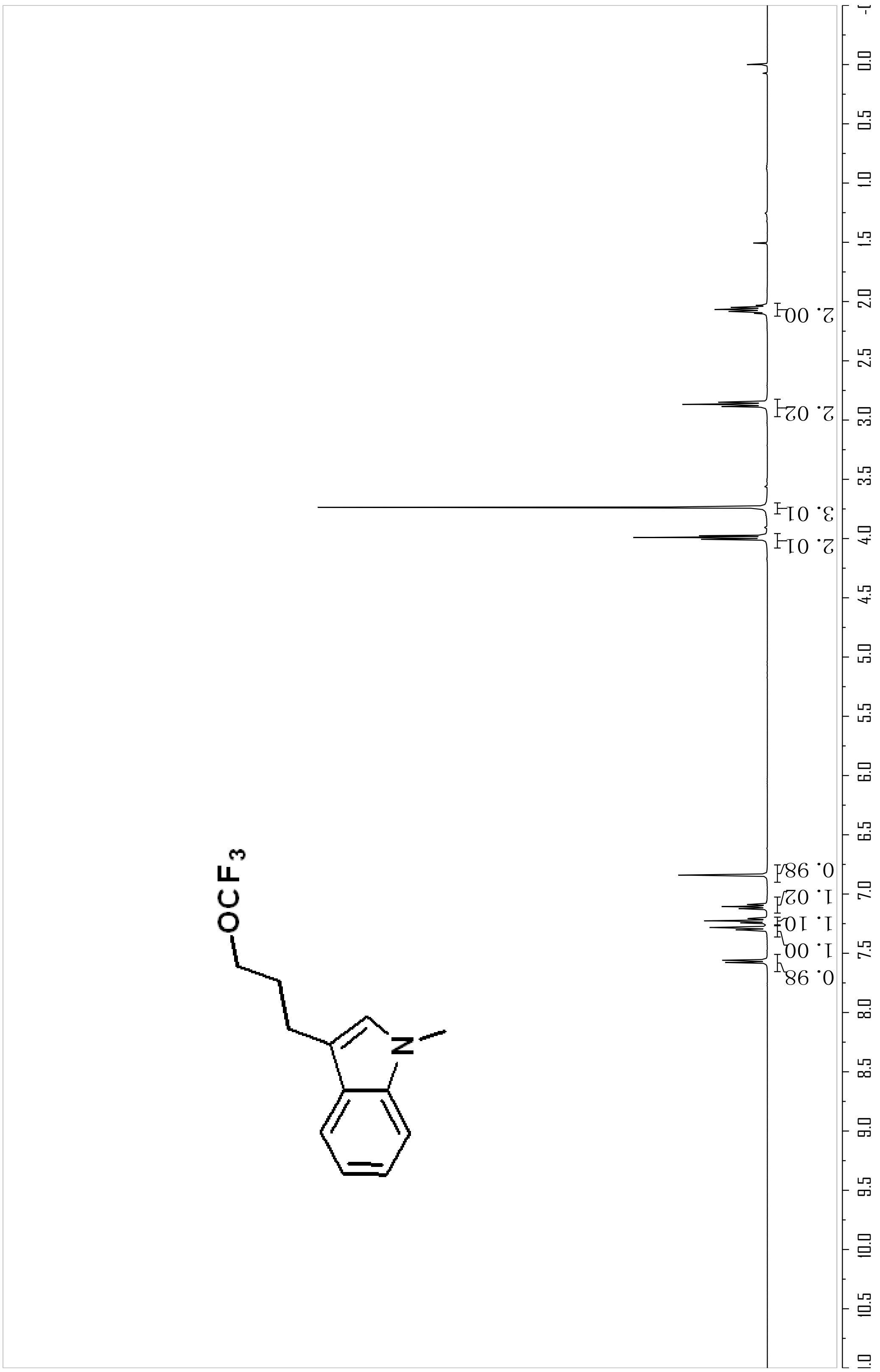

Supplementary Figure 168: ^{1}H NMR spectrum (400 MHz, $CDCl_3$, 23 °C) of **23**

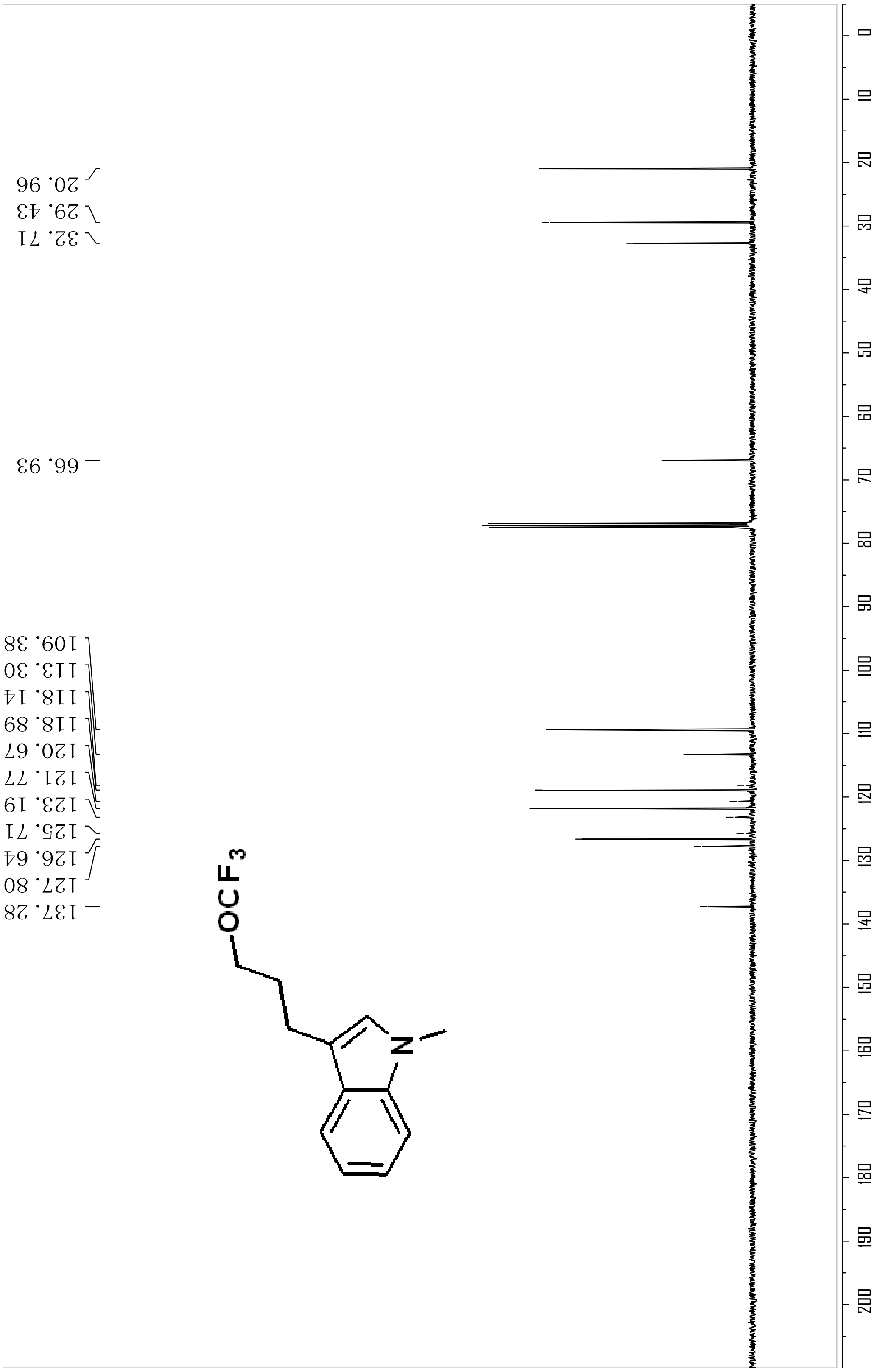

Supplementary Figure 169: ^{13}C NMR spectrum (101 MHz, $CDCl_3$, 23 °C) of **23**

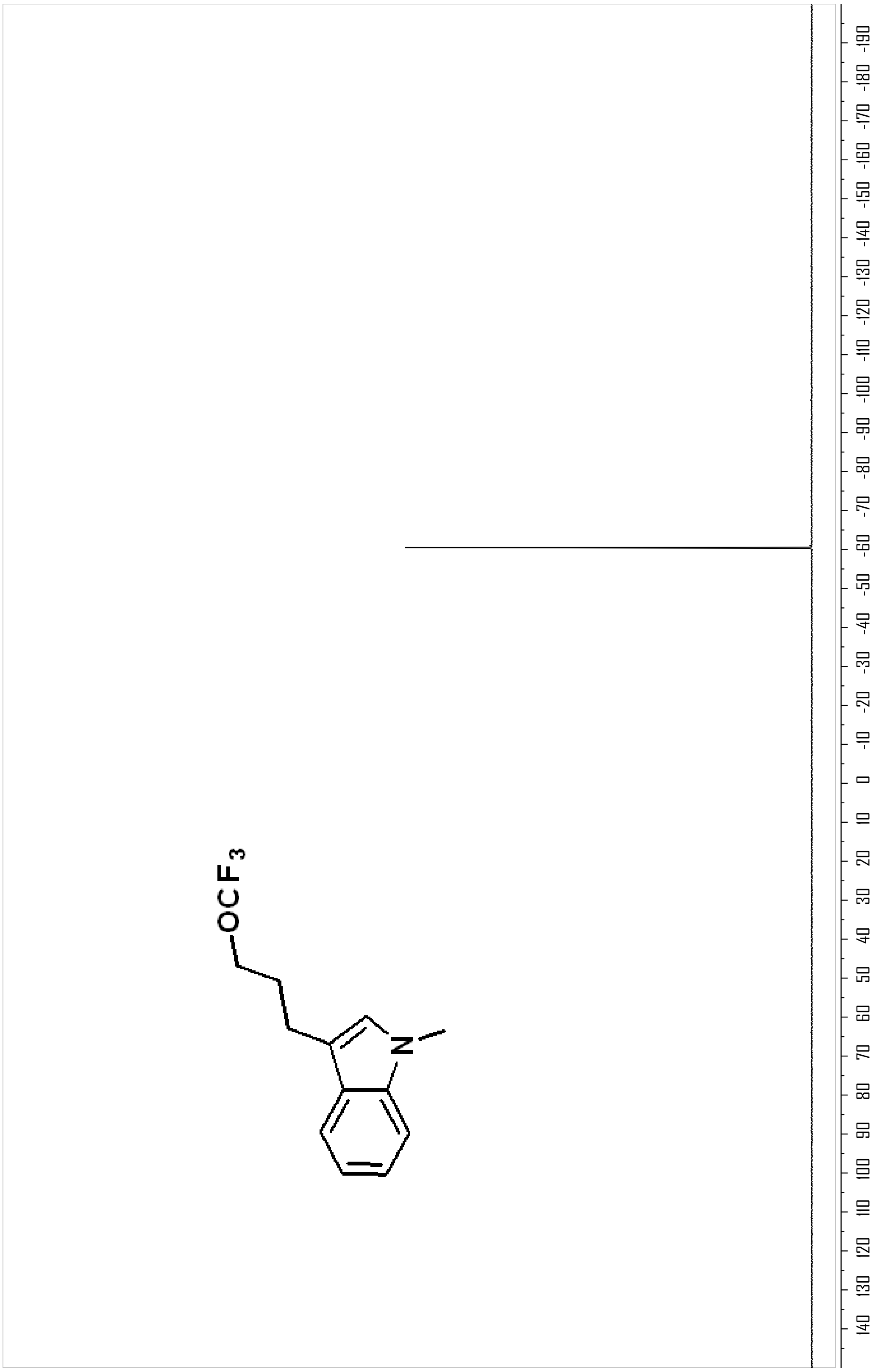

Supplementary Figure 170: ^{19}F NMR spectrum (376 MHz, $CDCl_3$, 23 °C) of **23**

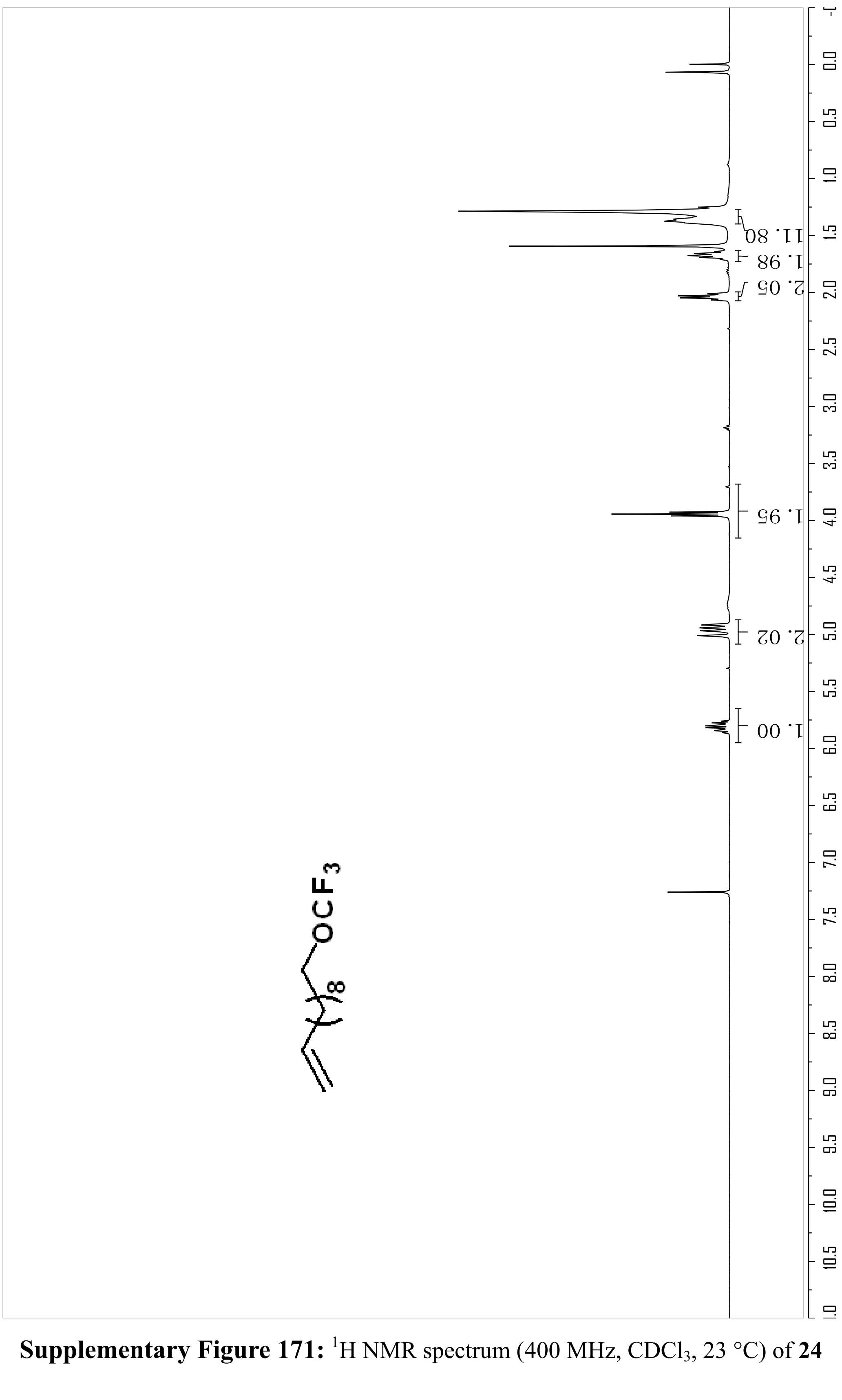

Supplementary Figure 171: ^{1}H NMR spectrum (400 MHz, $CDCl_3$, 23 °C) of **24**

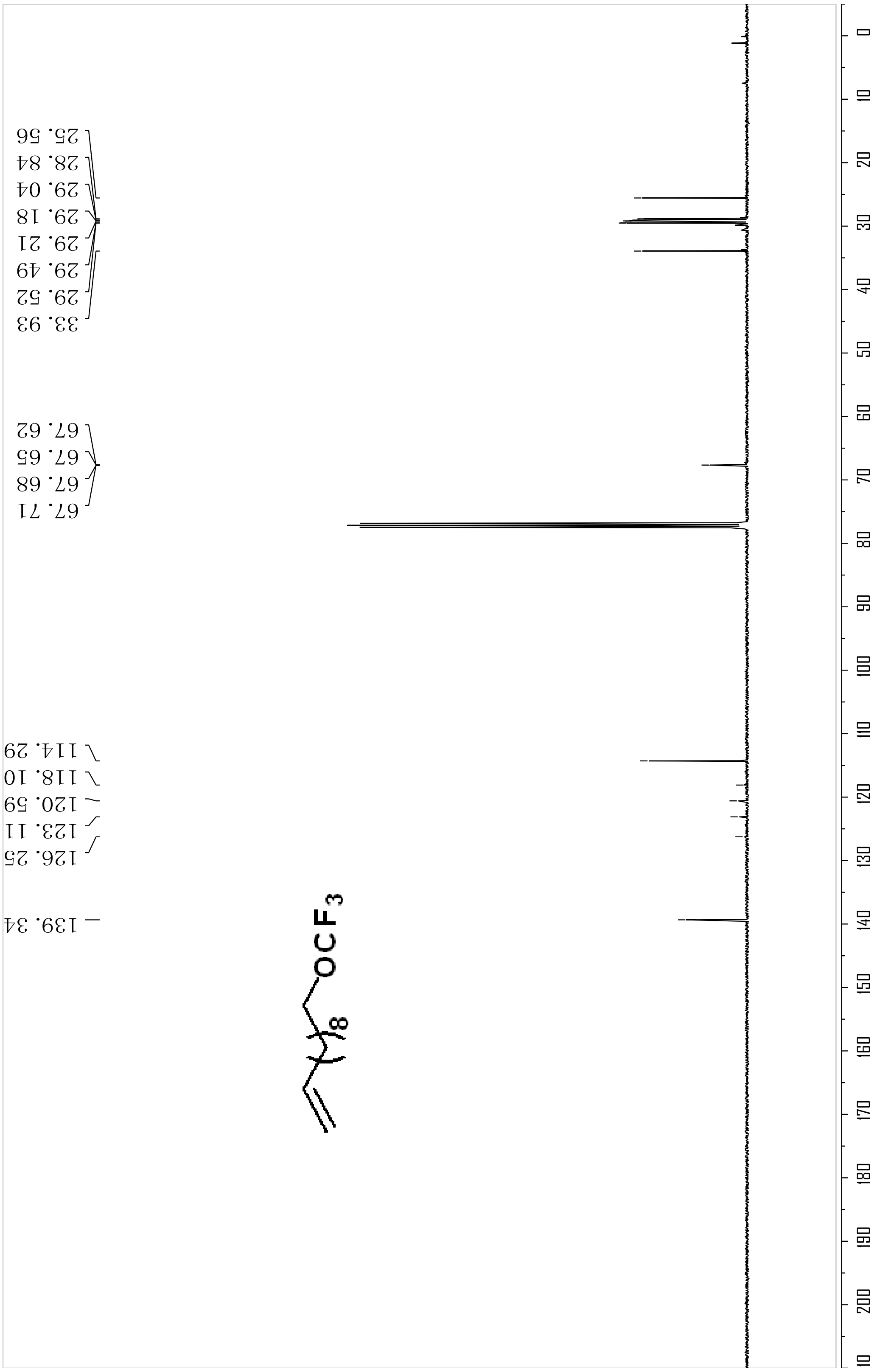

Supplementary Figure 172: ^{13}C NMR spectrum (101 MHz, $CDCl_3$, 23 °C) of **24**

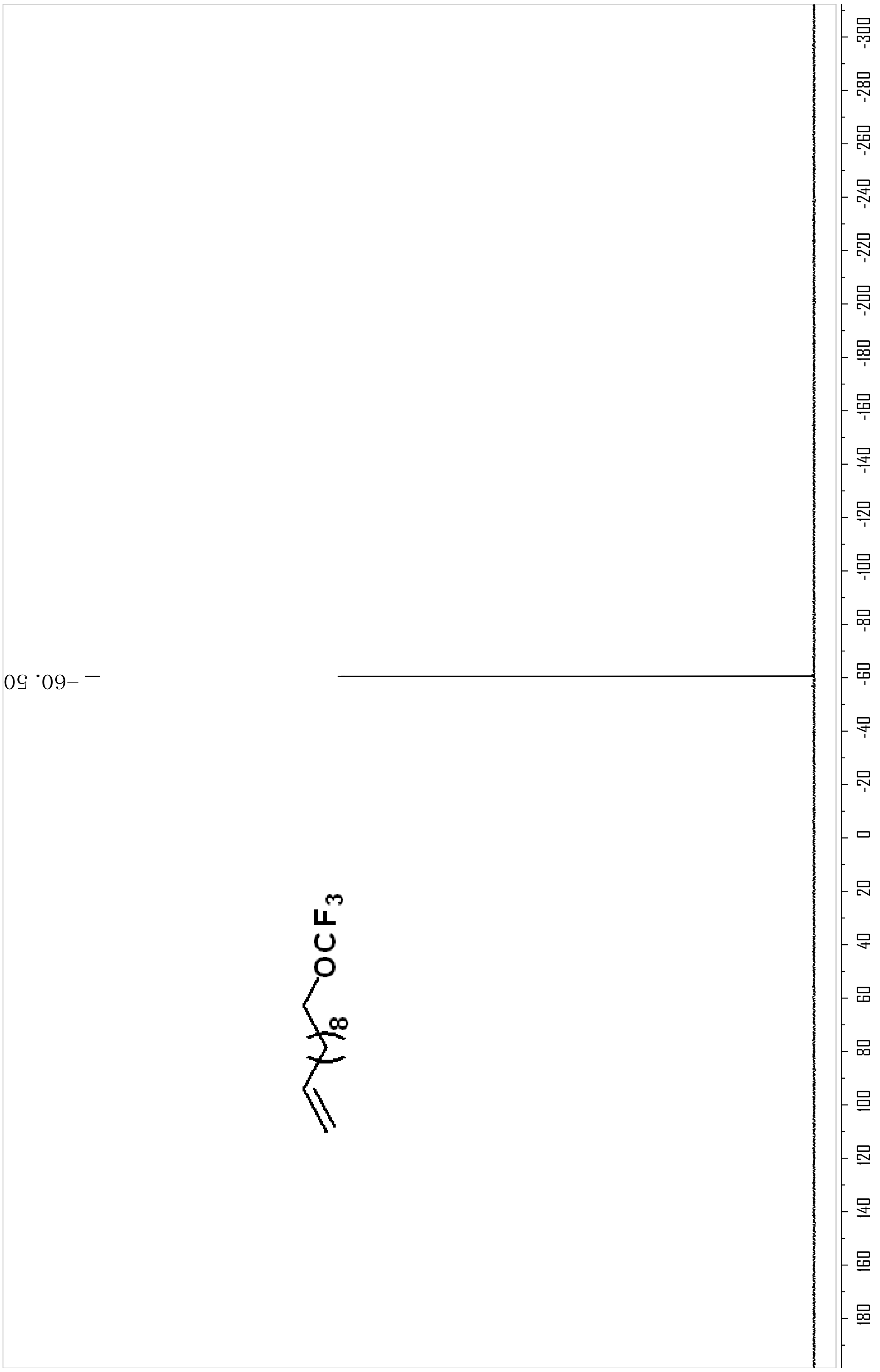

Supplementary Figure 173: ^{19}F NMR spectrum (376 MHz, $CDCl_3$, 23 °C) of **24**

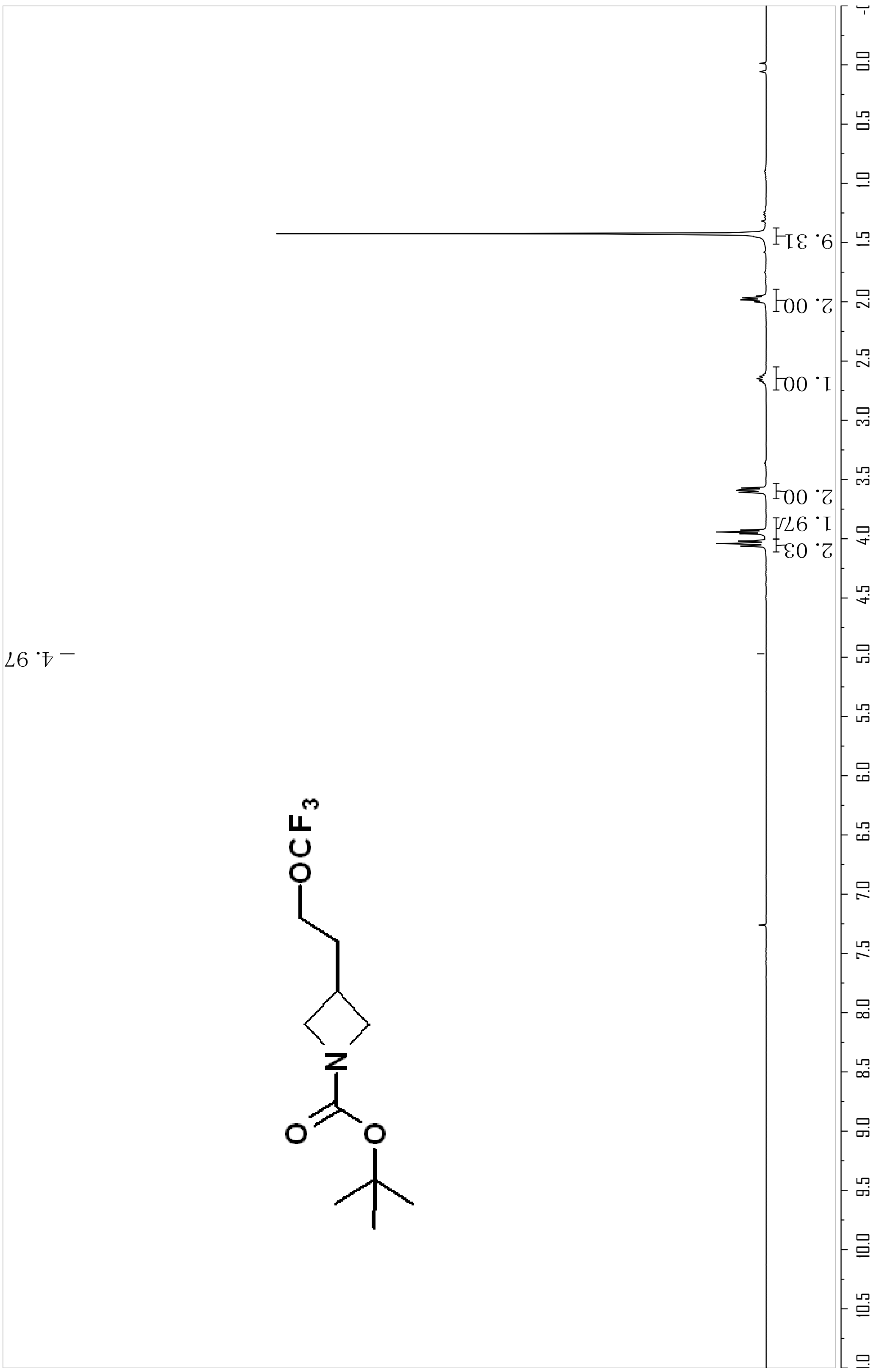

Supplementary Figure 174: ^{1}H NMR spectrum (400 MHz, $CDCl_3$, 23 °C) of **25**

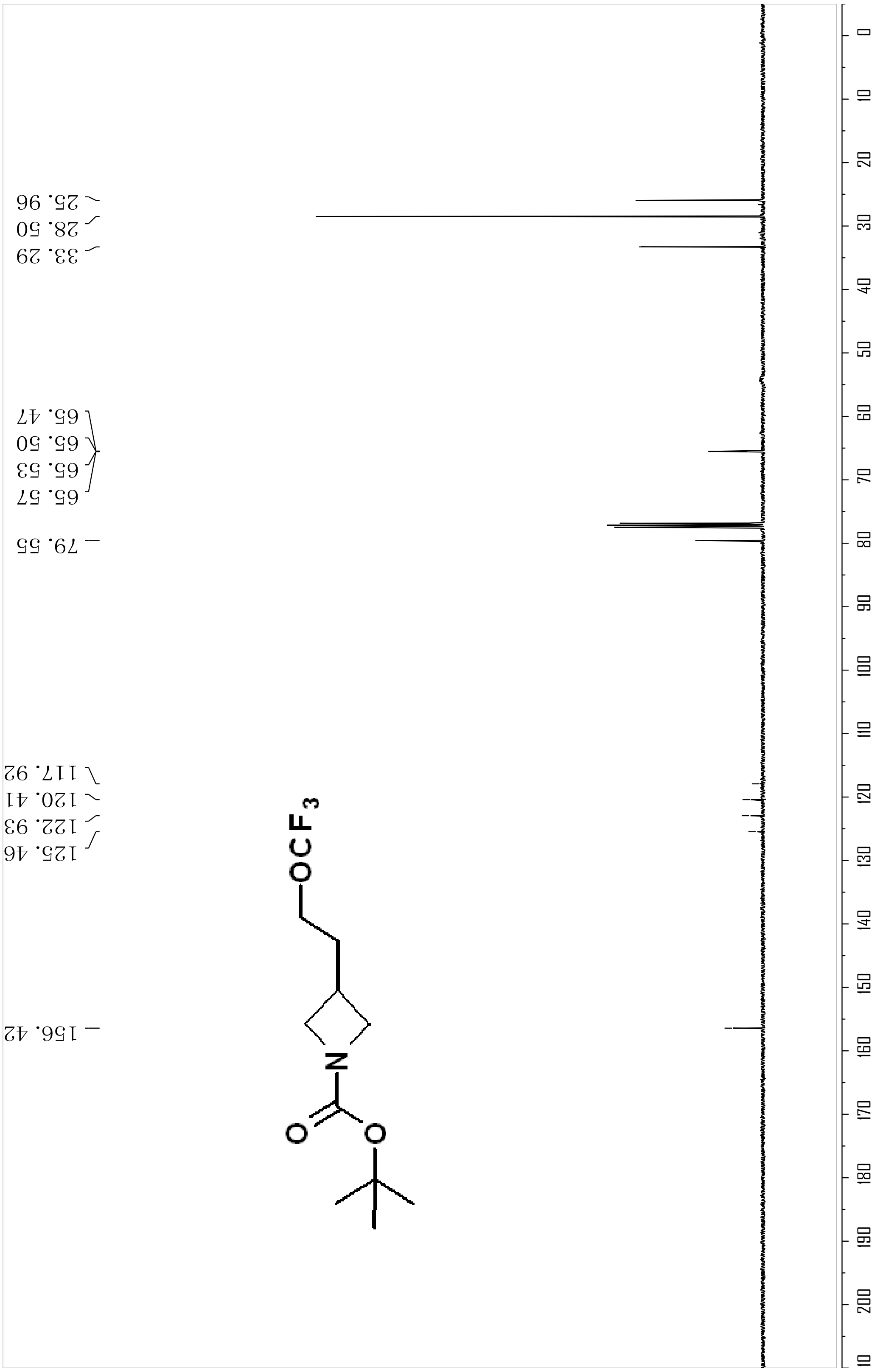

Supplementary Figure 175: ^{13}C NMR spectrum (101 MHz, $CDCl_3$, 23 °C) of **25**

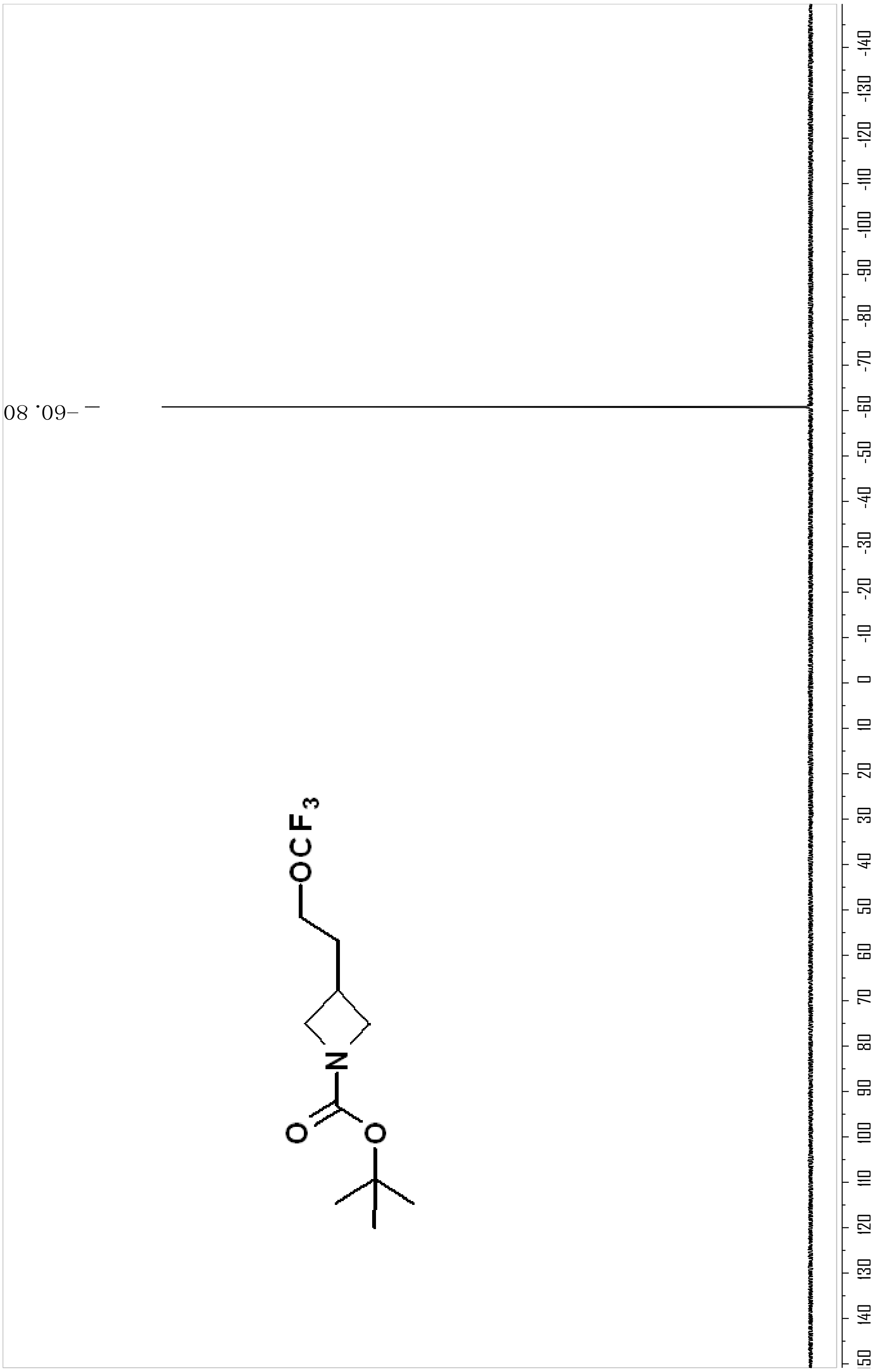

Supplementary Figure 176: ^{19}F NMR spectrum (376 MHz, $CDCl_3$, 23 °C) of **25**

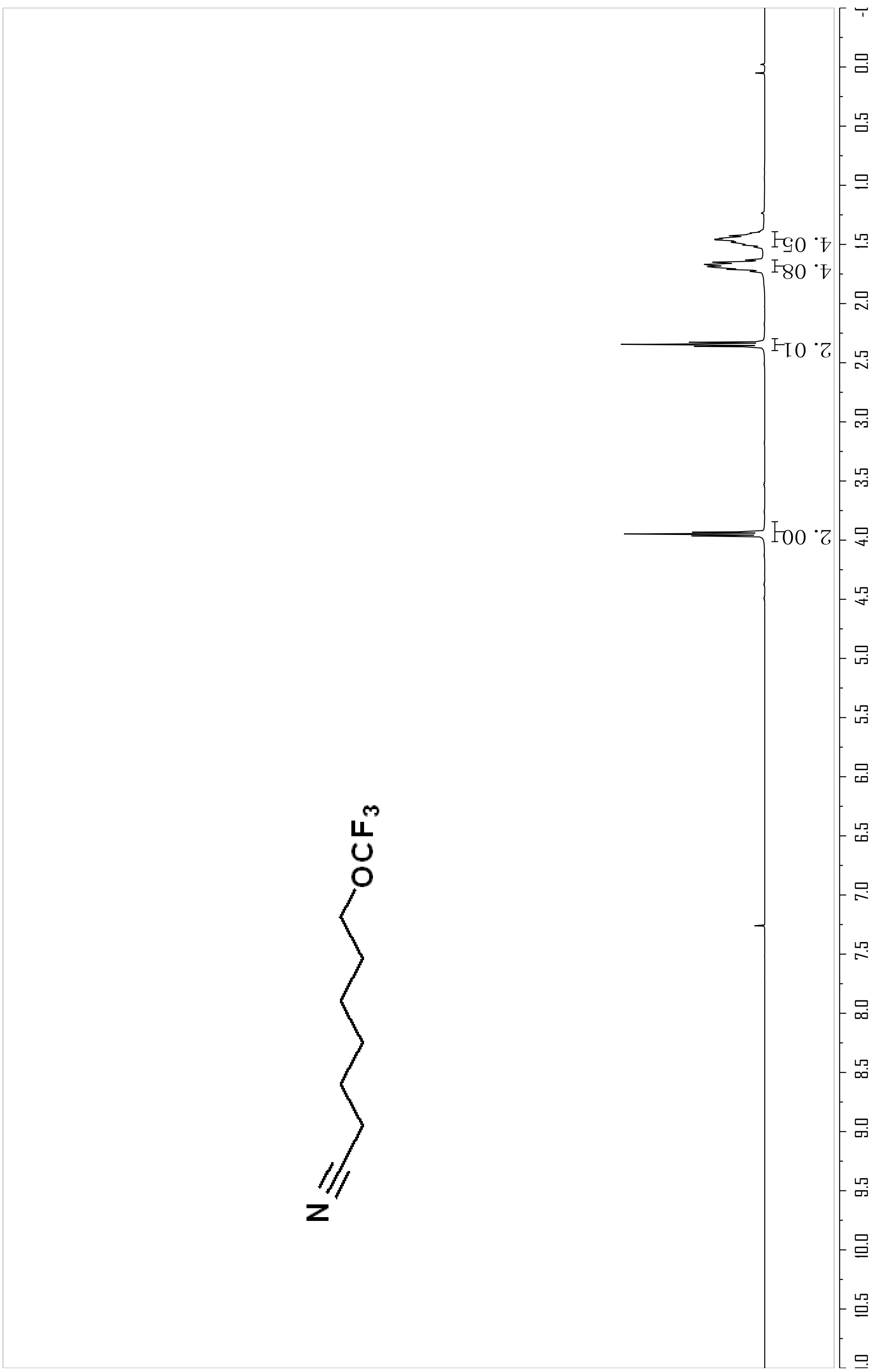

Supplementary Figure 177: ^{1}H NMR spectrum (400 MHz, $CDCl_3$, 23 °C) of **26**

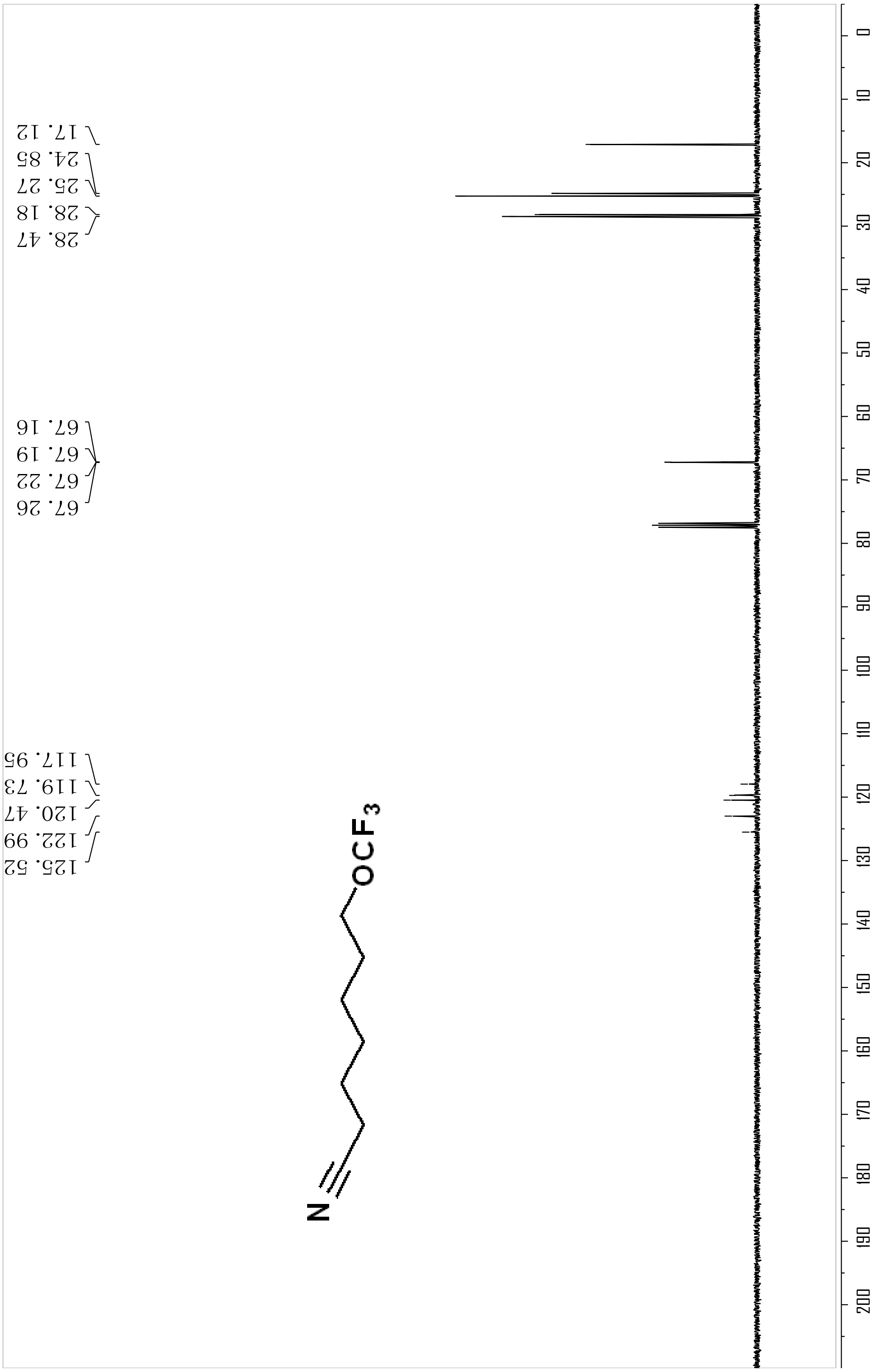

Supplementary Figure 178: ^{13}C NMR spectrum (101 MHz, $CDCl_3$, 23 °C) of **26**

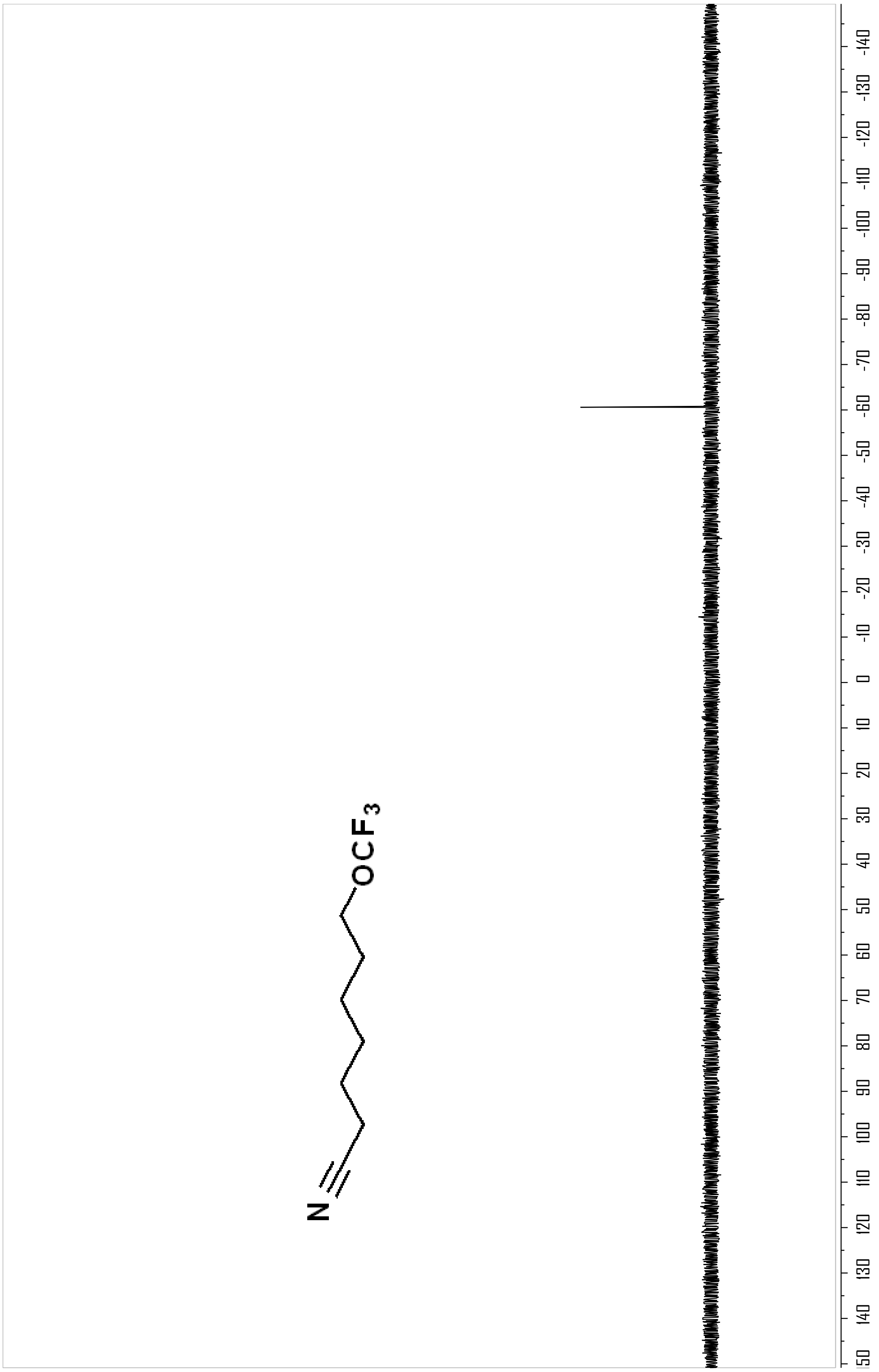

Supplementary Figure 179: ^{19}F NMR spectrum (376 MHz, $CDCl_3$, 23 °C) of **26**

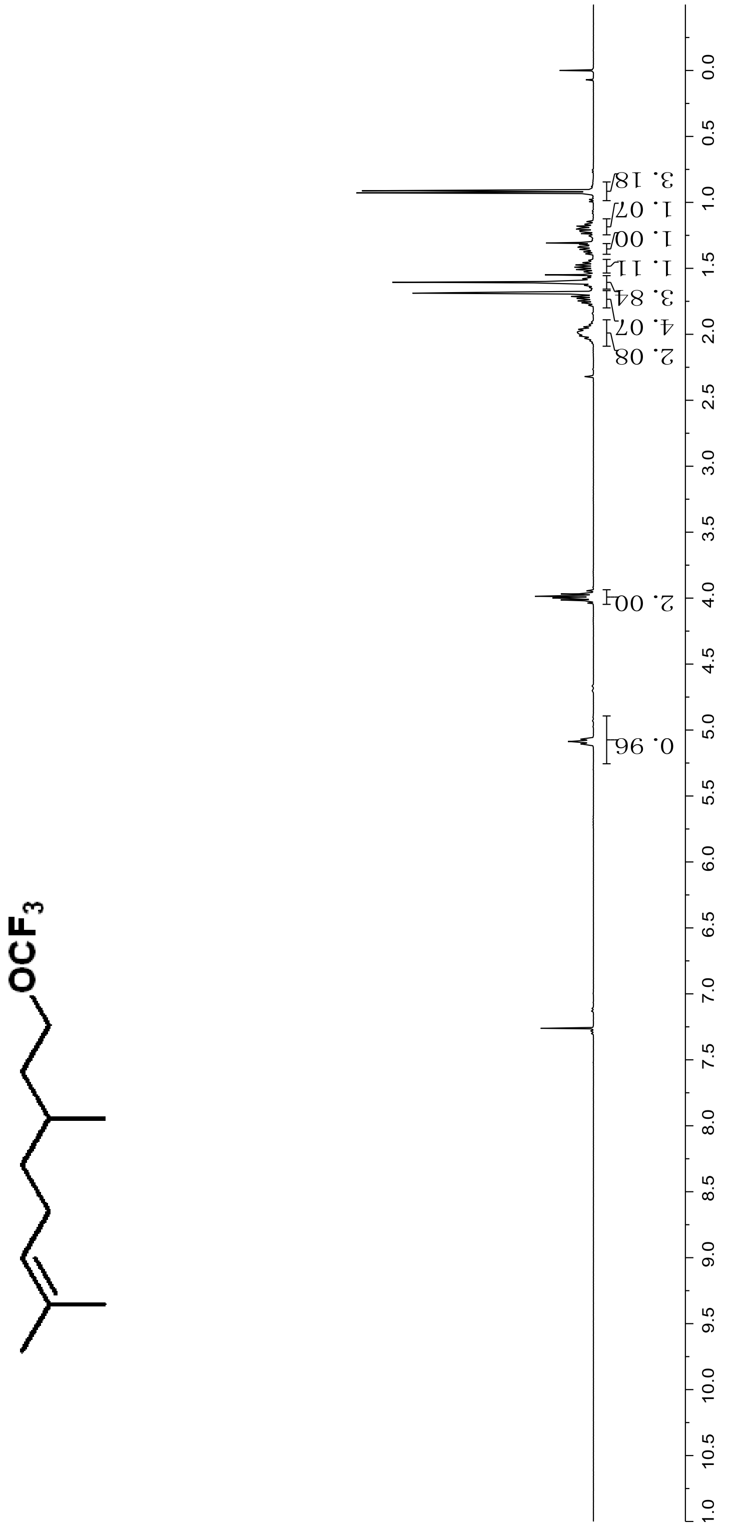

Supplementary Figure 180: ^{1}H NMR spectrum (400 MHz, $CDCl_3$, 23 °C) of **27**

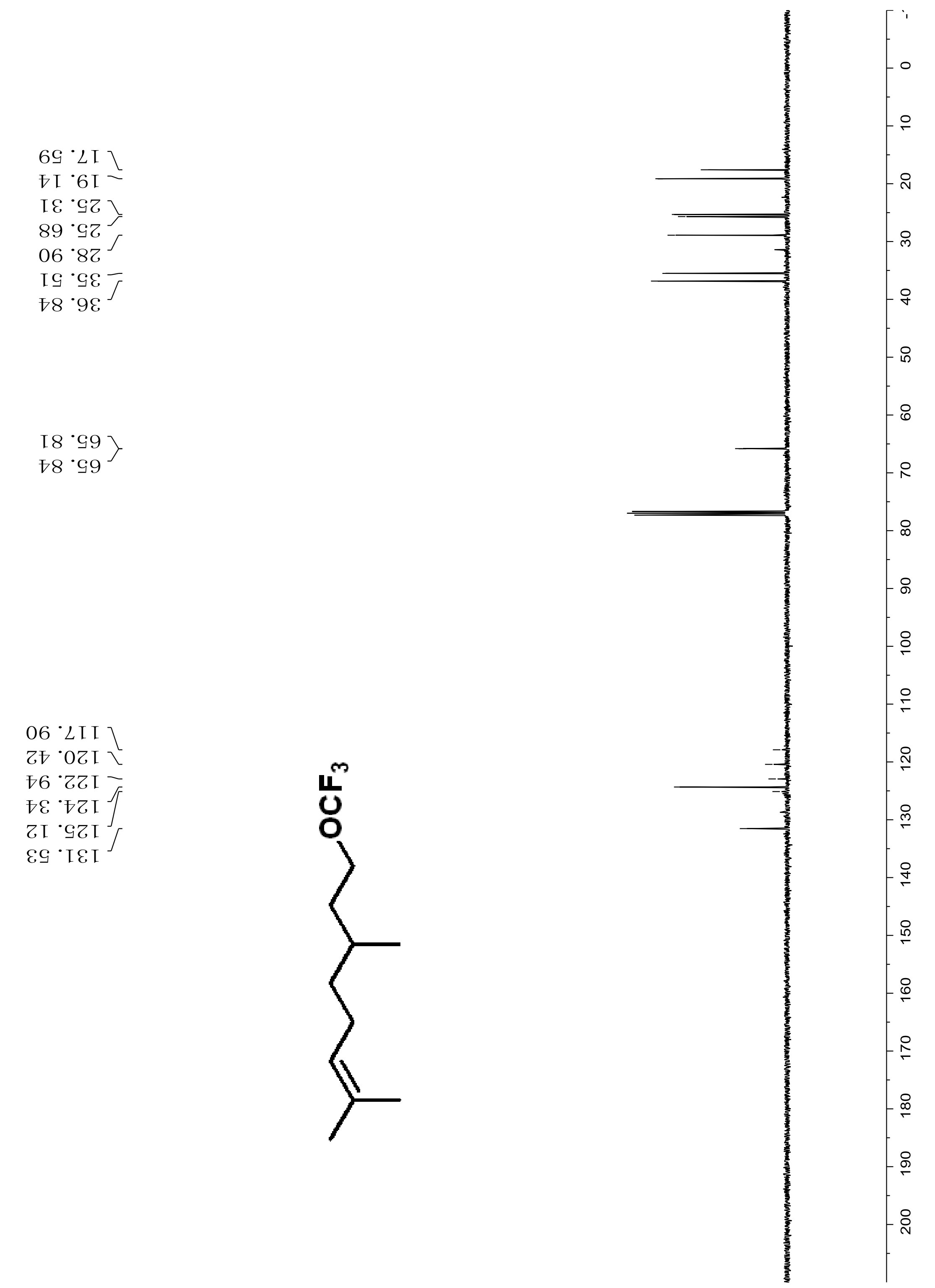

Supplementary Figure 181: ^{13}C NMR spectrum (101 MHz, $CDCl_3$, 23 °C) of **27**

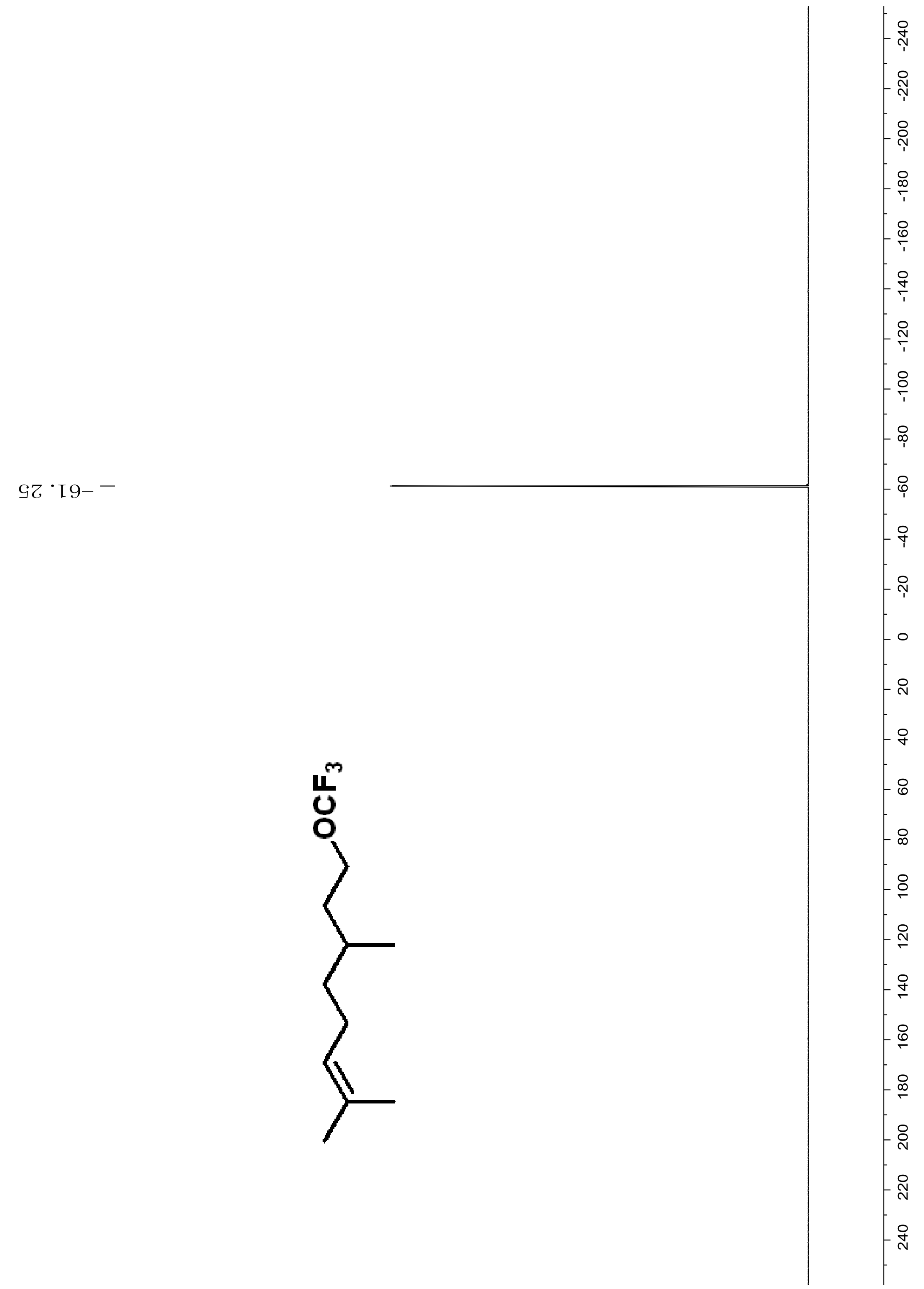

Supplementary Figure 182: ^{19}F NMR spectrum (376 MHz, DMSO, 23 °C) of **27**

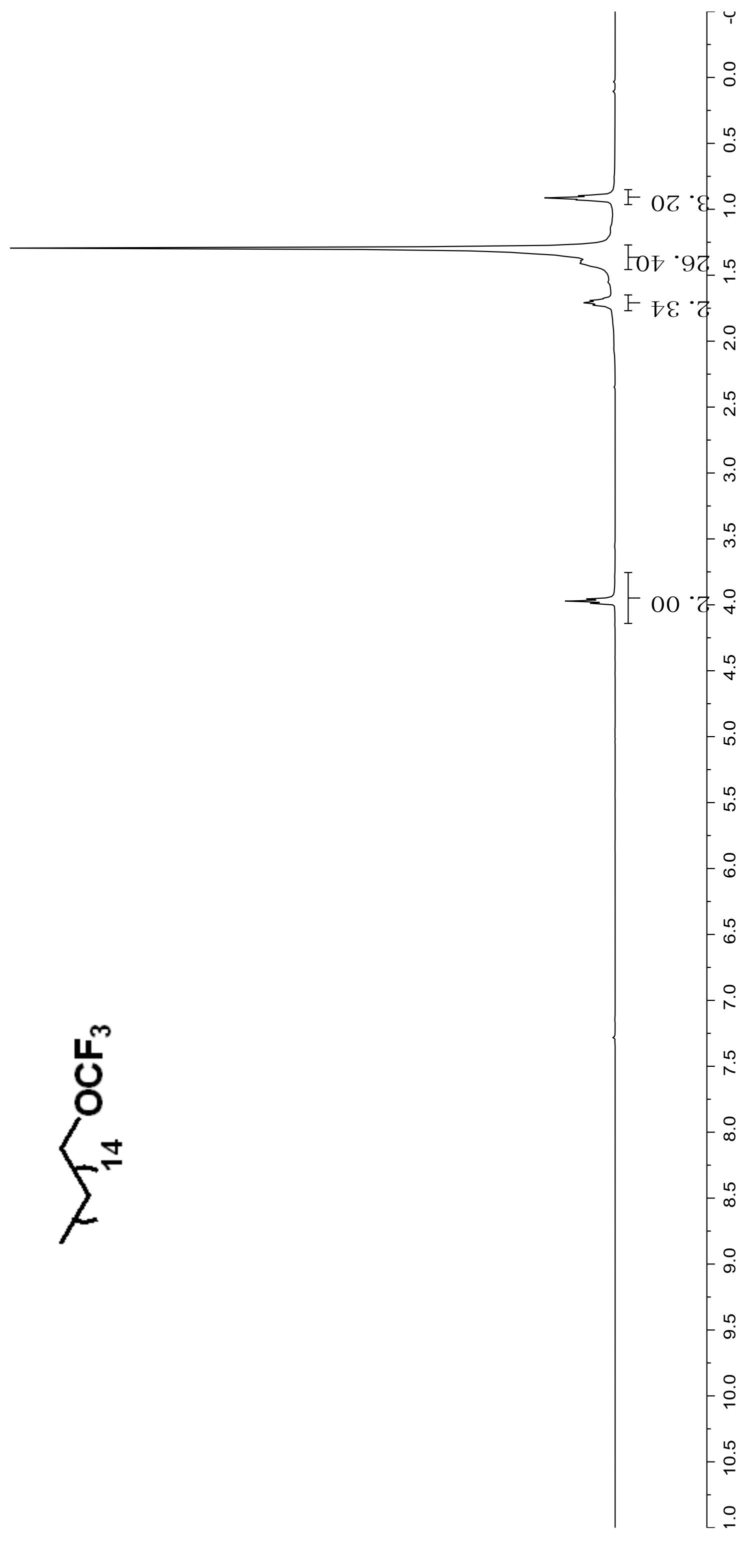

Supplementary Figure 183: ^{1}H NMR spectrum (400 MHz, $CDCl_3$, 23 °C) of **28**

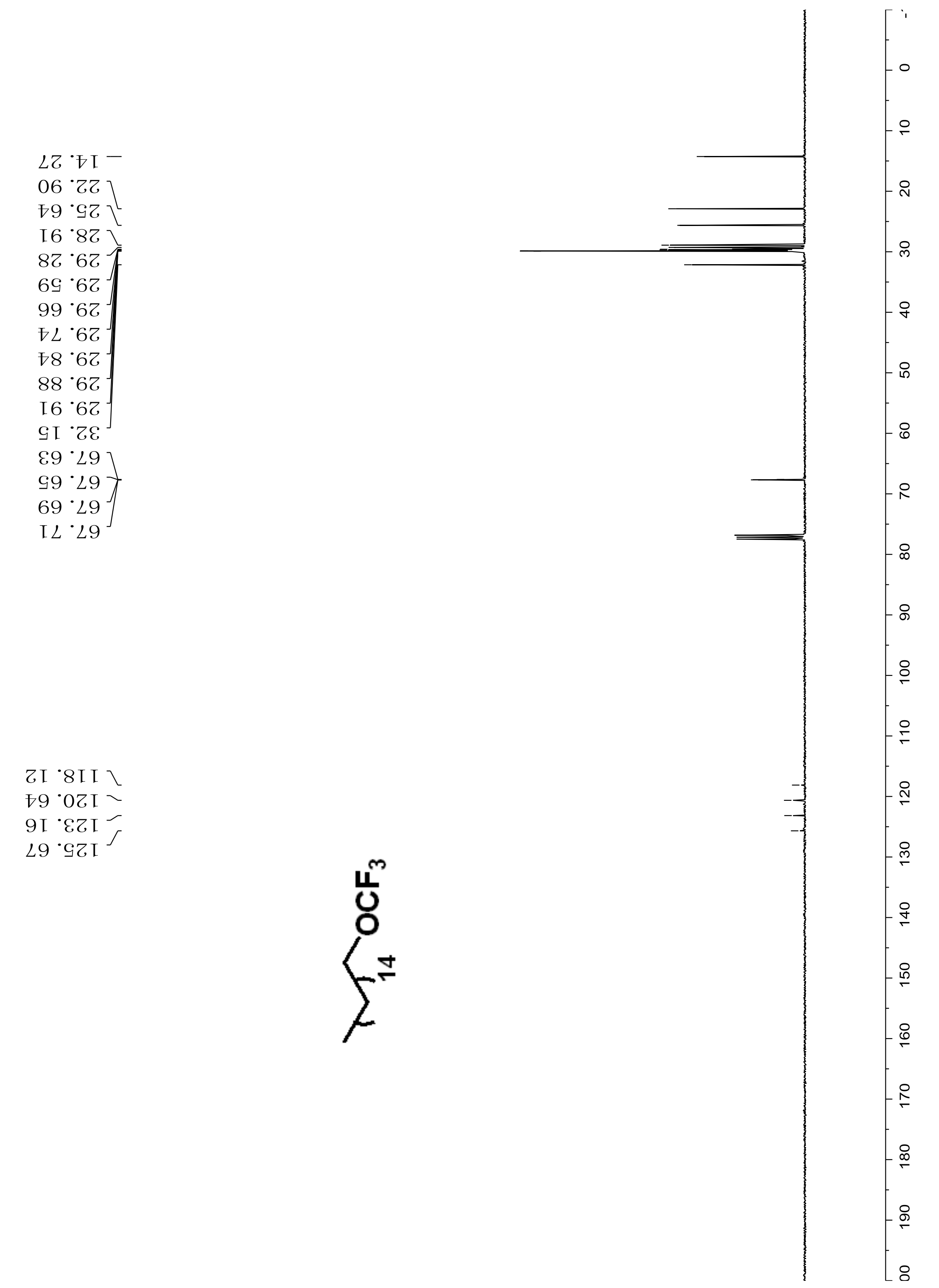

Supplementary Figure 184: ^{13}C NMR spectrum (101 MHz, $CDCl_3$, 23 °C) of **28**

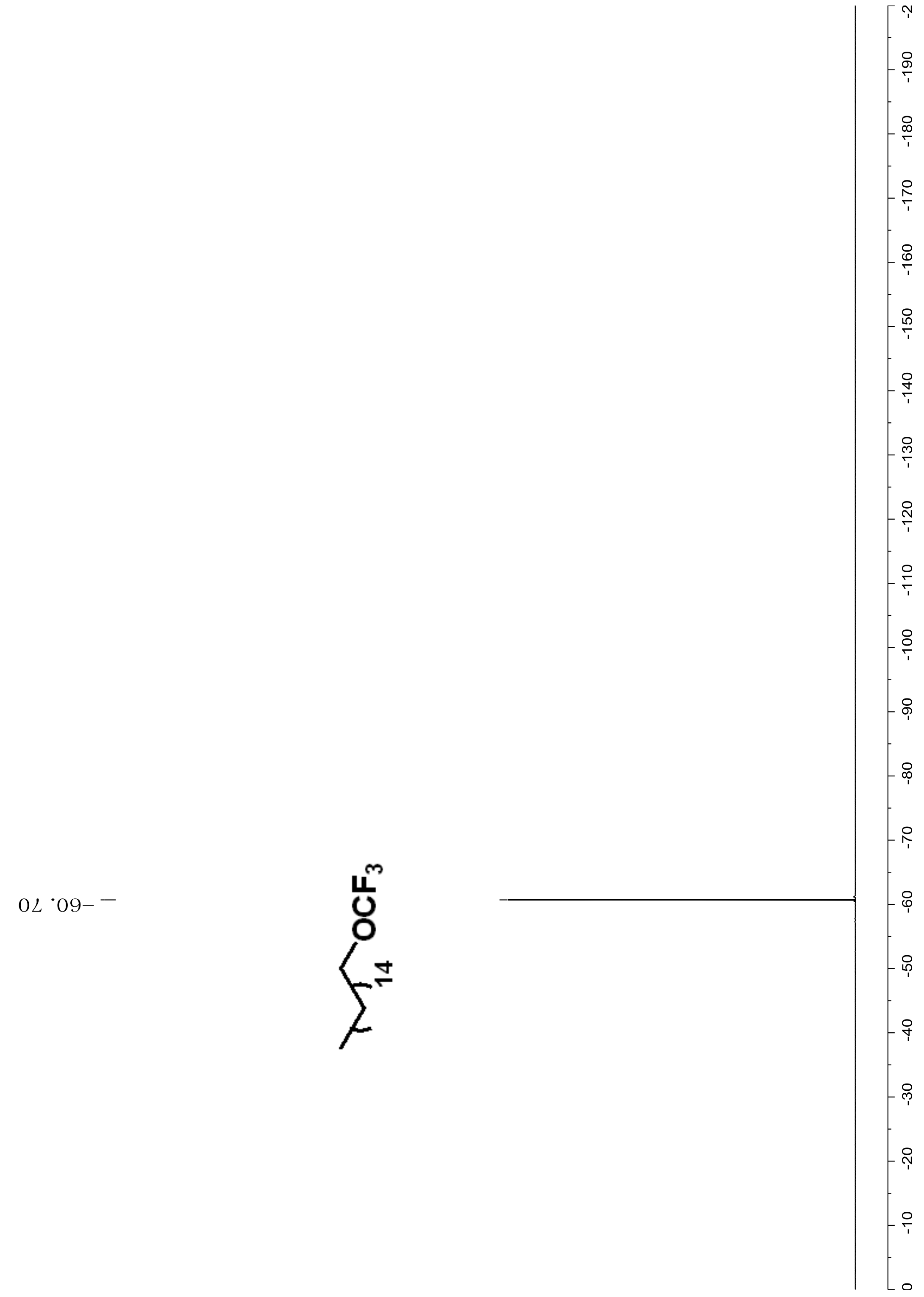

Supplementary Figure 185: ^{19}F NMR spectrum (376 MHz, $CDCl_3$, 23 °C) of **28**

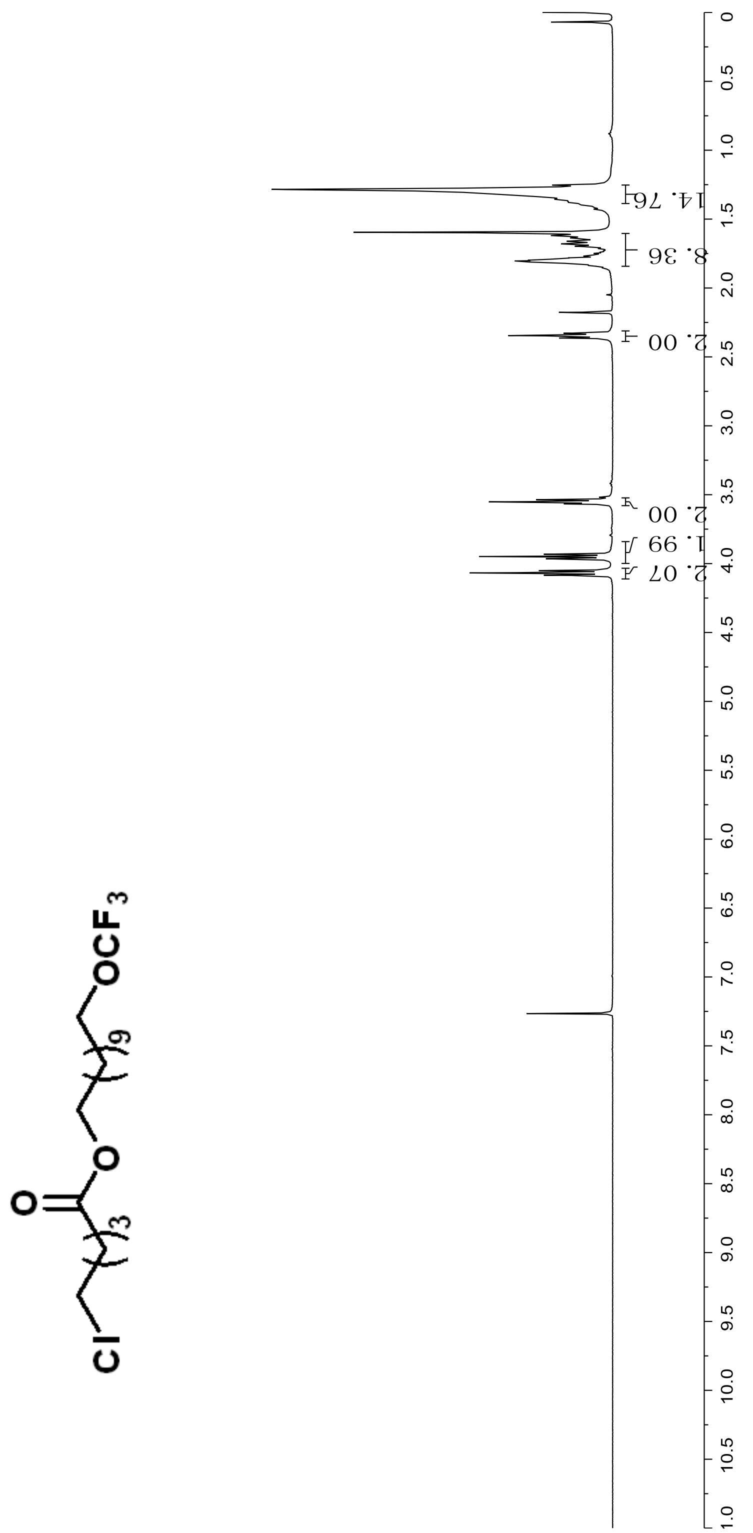

Supplementary Figure 186: ^{1}H NMR spectrum (400 MHz, $CDCl_3$, 23 °C) of **29**

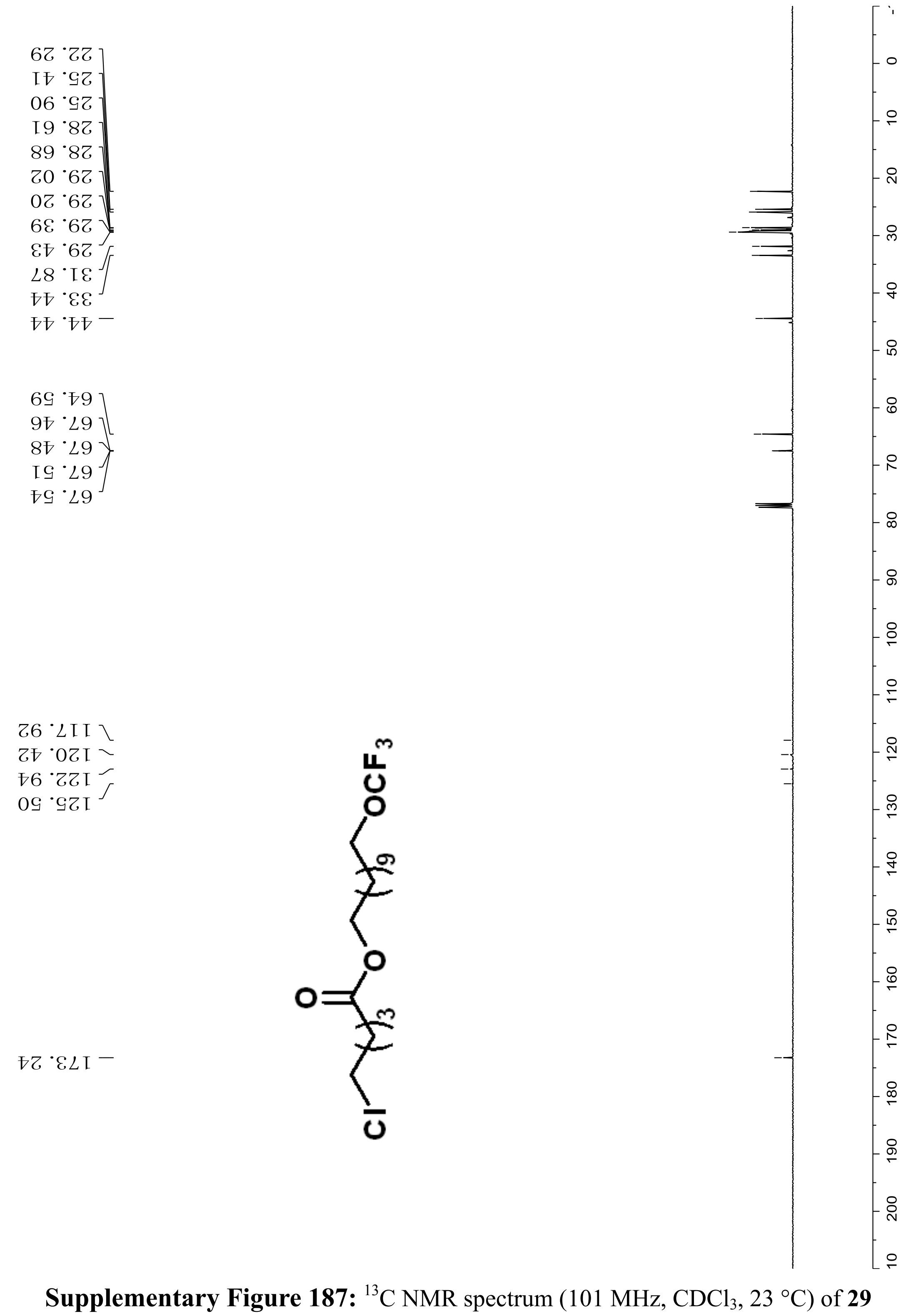

Supplementary Figure 187: ^{13}C NMR spectrum (101 MHz, $CDCl_3$, 23 °C) of **29**

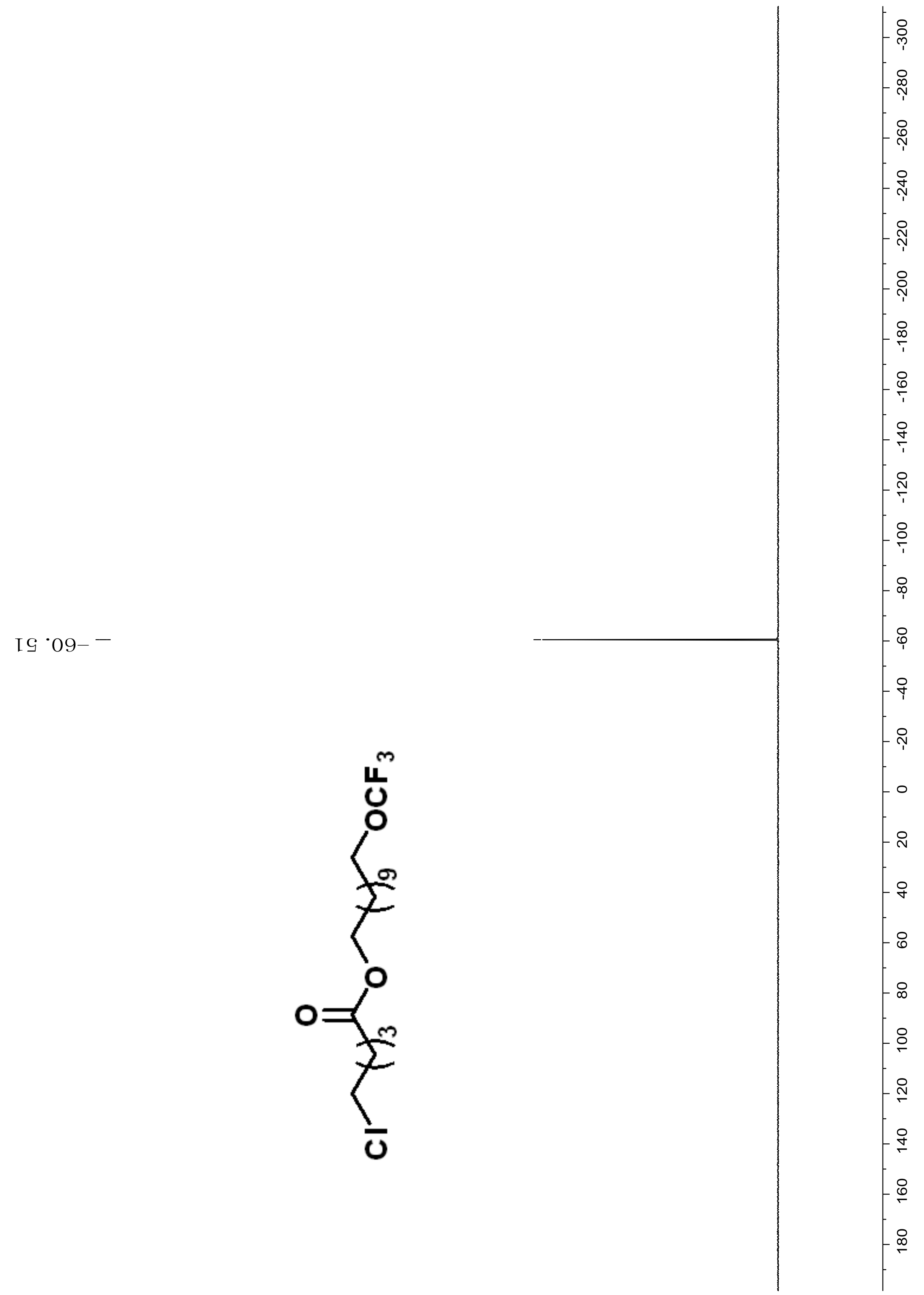

Supplementary Figure 188: ^{19}F NMR spectrum (376 MHz, DMSO, 23 °C) of **29**

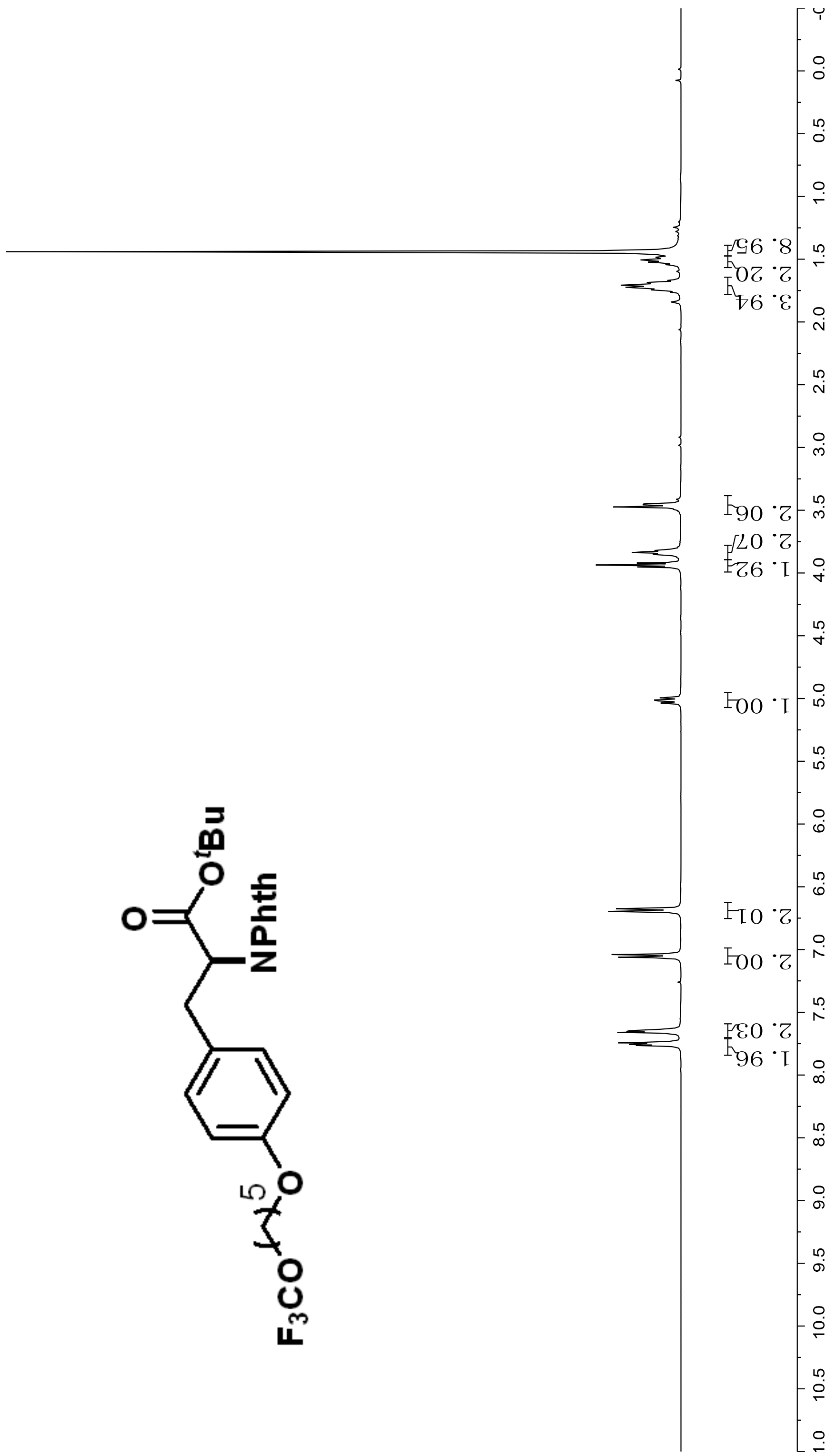

Supplementary Figure 189: ^{1}H NMR spectrum (400 MHz, $CDCl_3$, 23 °C) of **30**

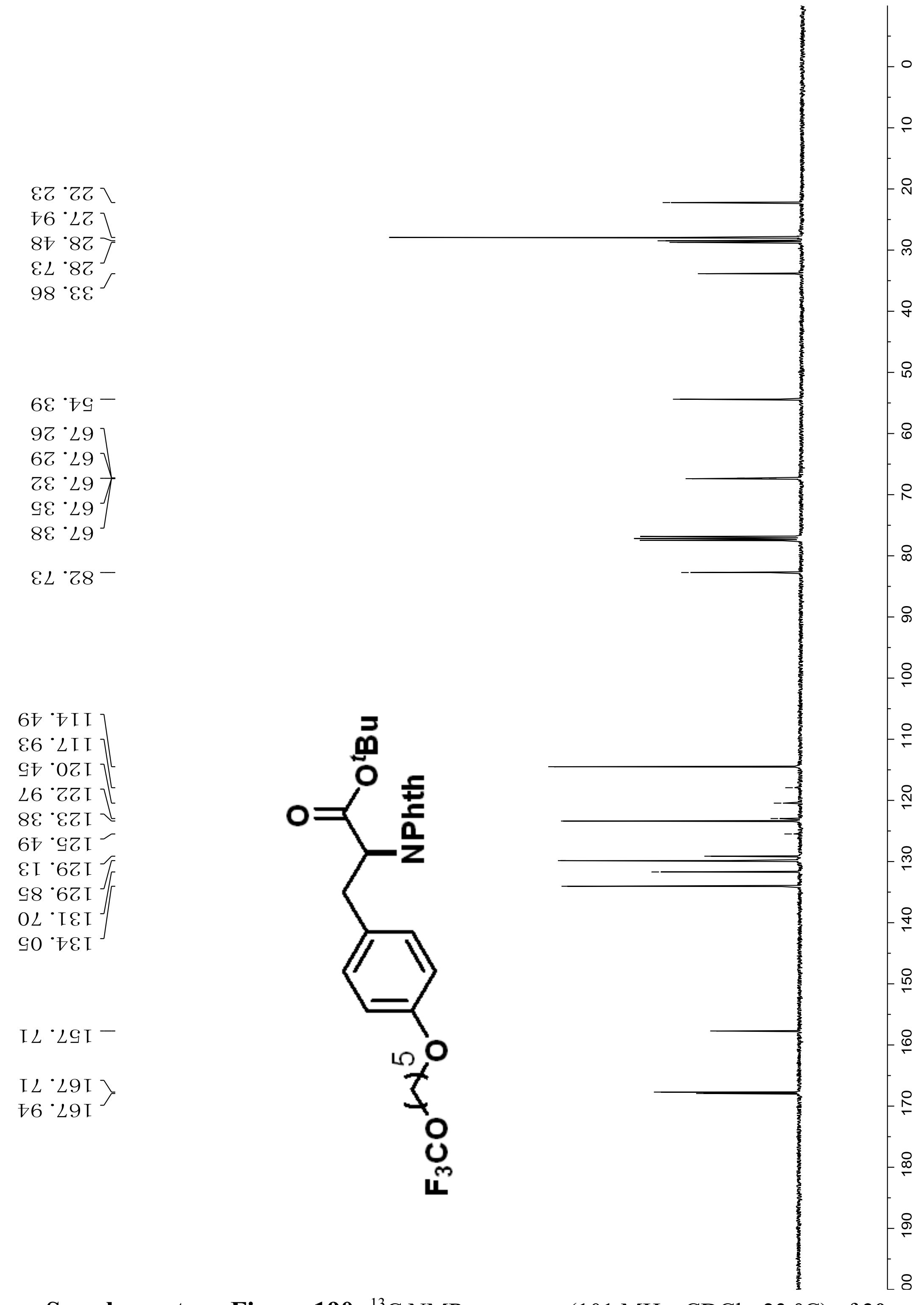

Supplementary Figure 190: ^{13}C NMR spectrum (101 MHz, $CDCl_3$, 23 °C) of **30**

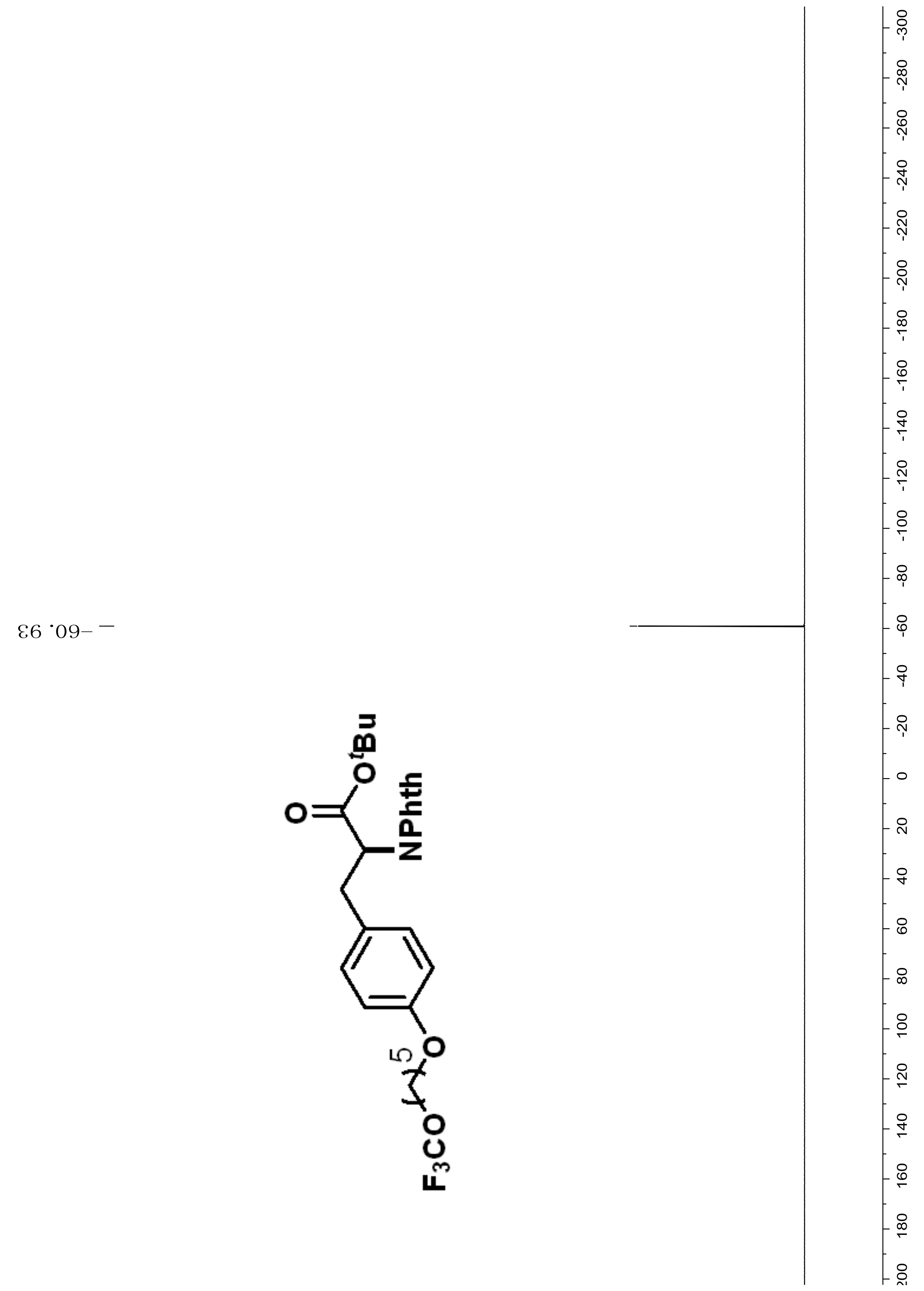

Supplementary Figure 191: ^{19}F NMR spectrum (376 MHz, DMSO, 23 °C) of **30**

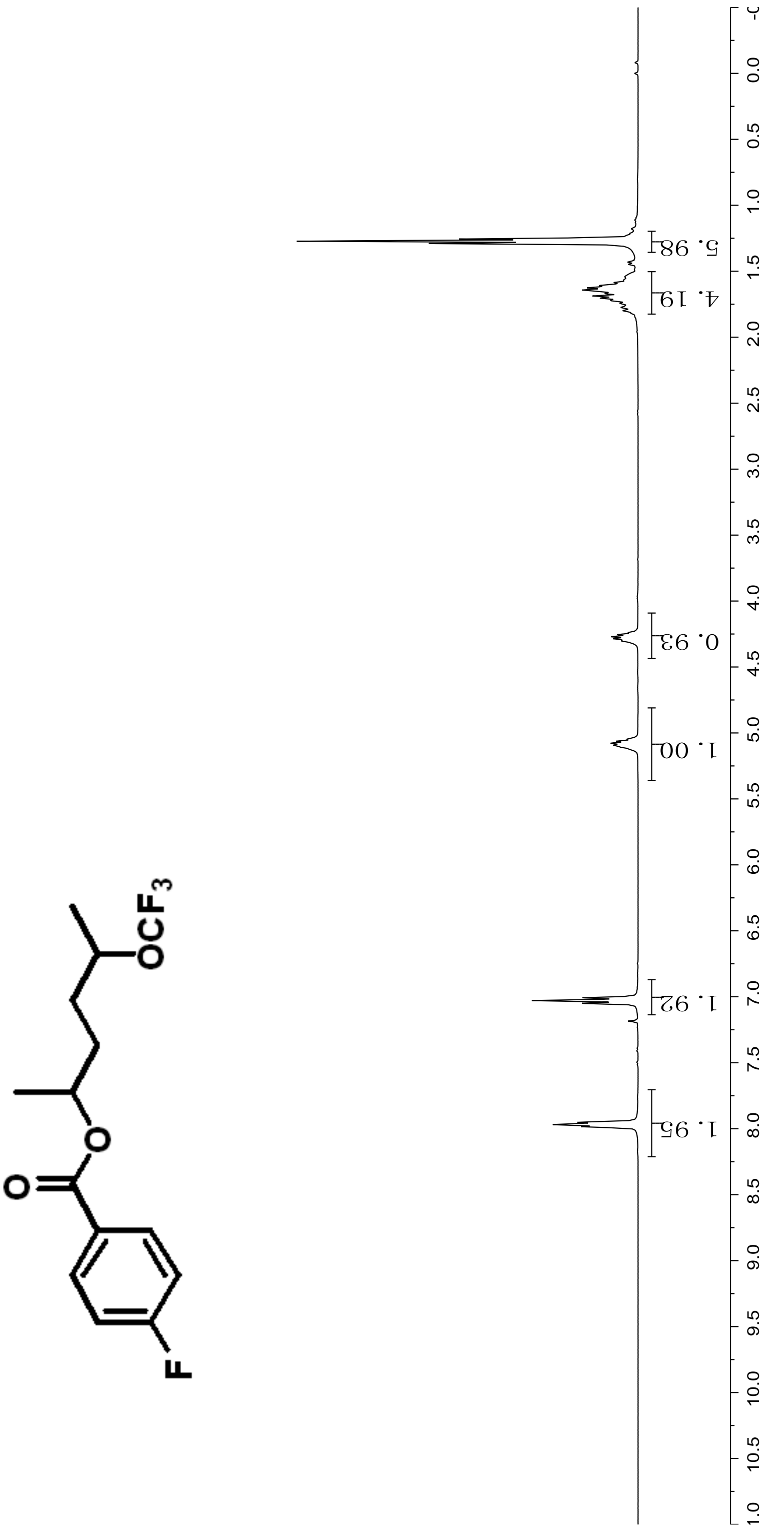

Supplementary Figure 192: ^{1}H NMR spectrum (400 MHz, $CDCl_3$, 23 °C) of **32**

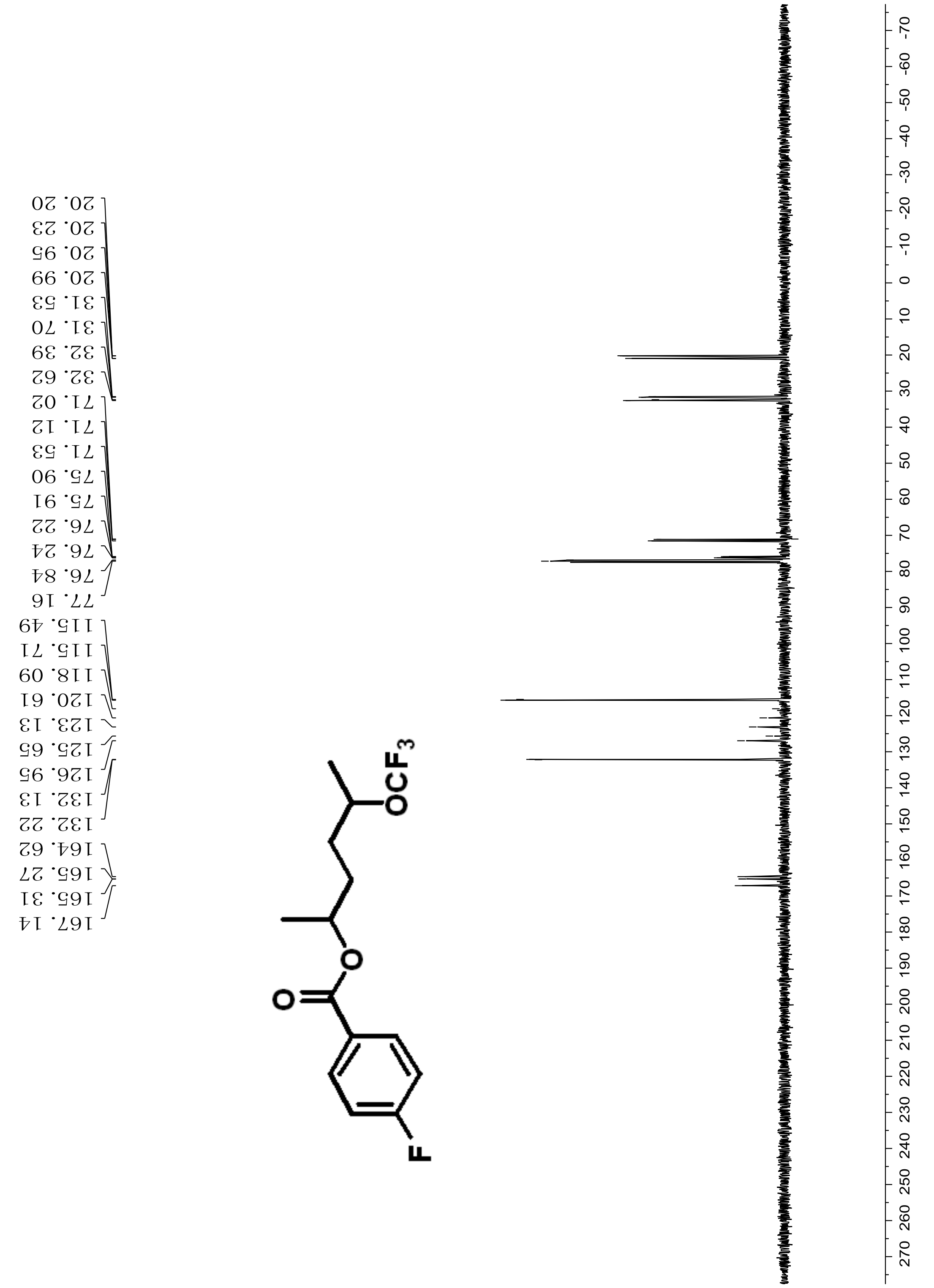

Supplementary Figure 193: ^{13}C NMR spectrum (101 MHz, $CDCl_3$, 23 °C) of **32**

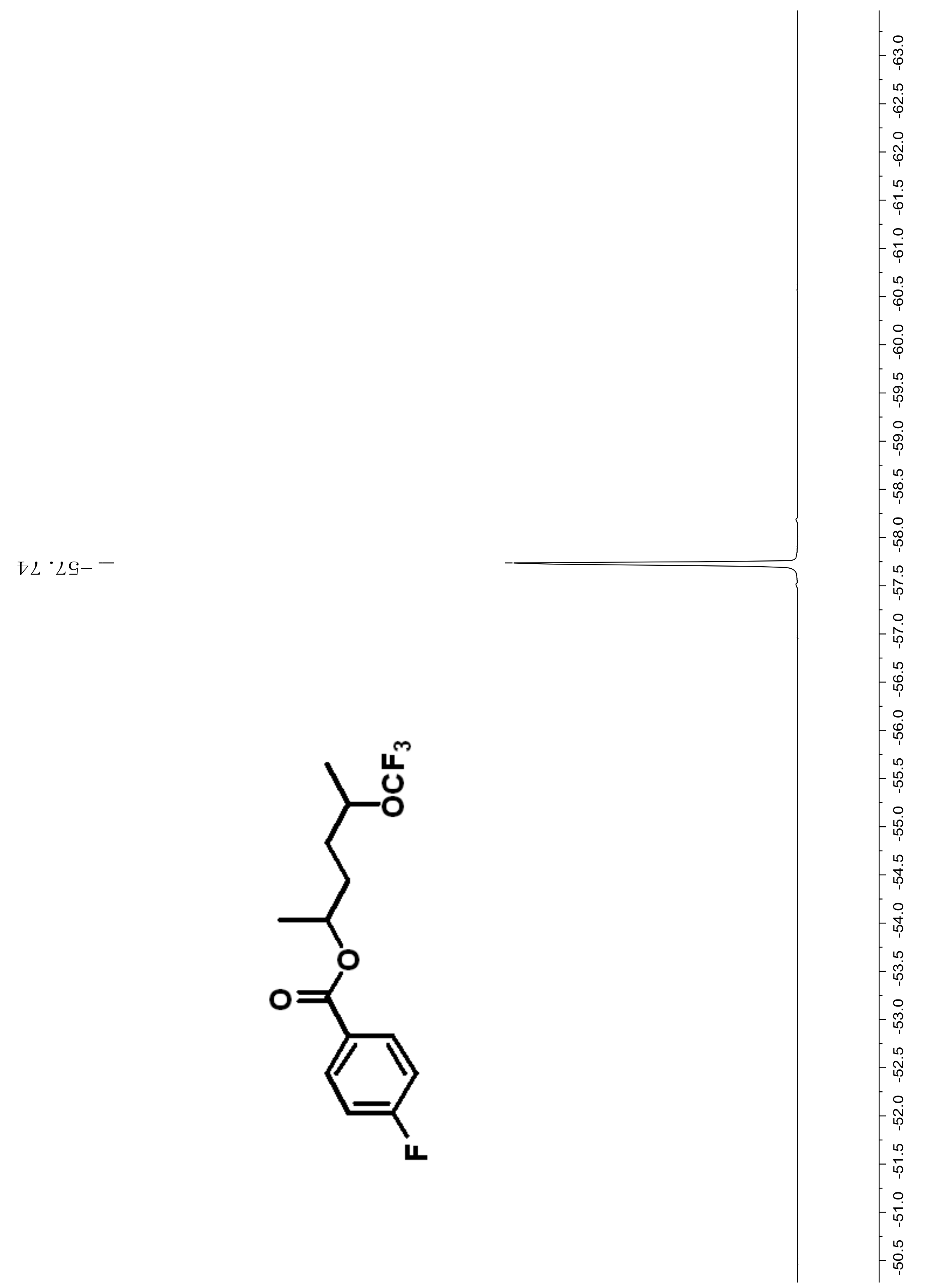

Supplementary Figure 194: ^{19}F NMR spectrum (376 MHz, $CDCl_3$, 23 °C) of **32**

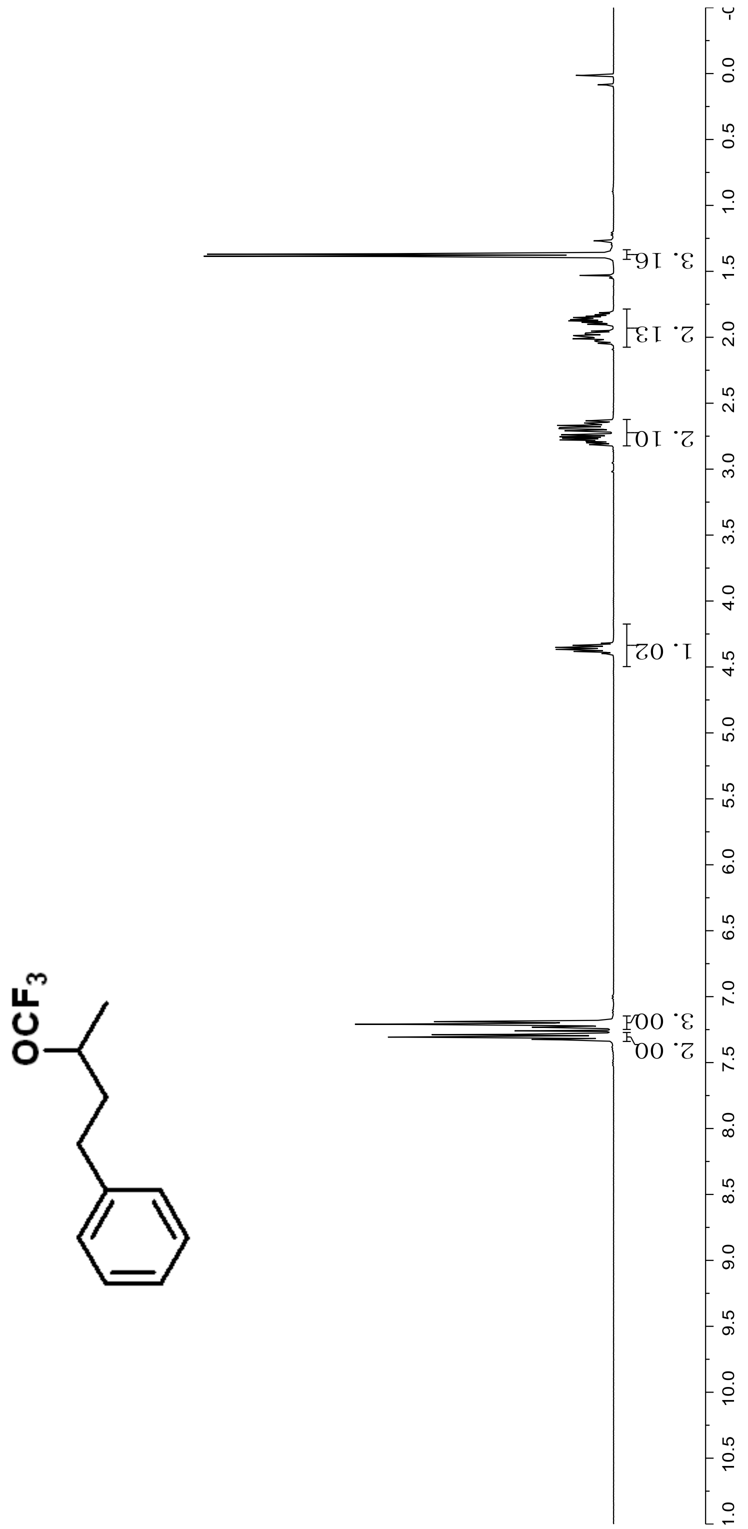

Supplementary Figure 195: ^{1}H NMR spectrum (400 MHz, $CDCl_3$, 23 °C) of **33**

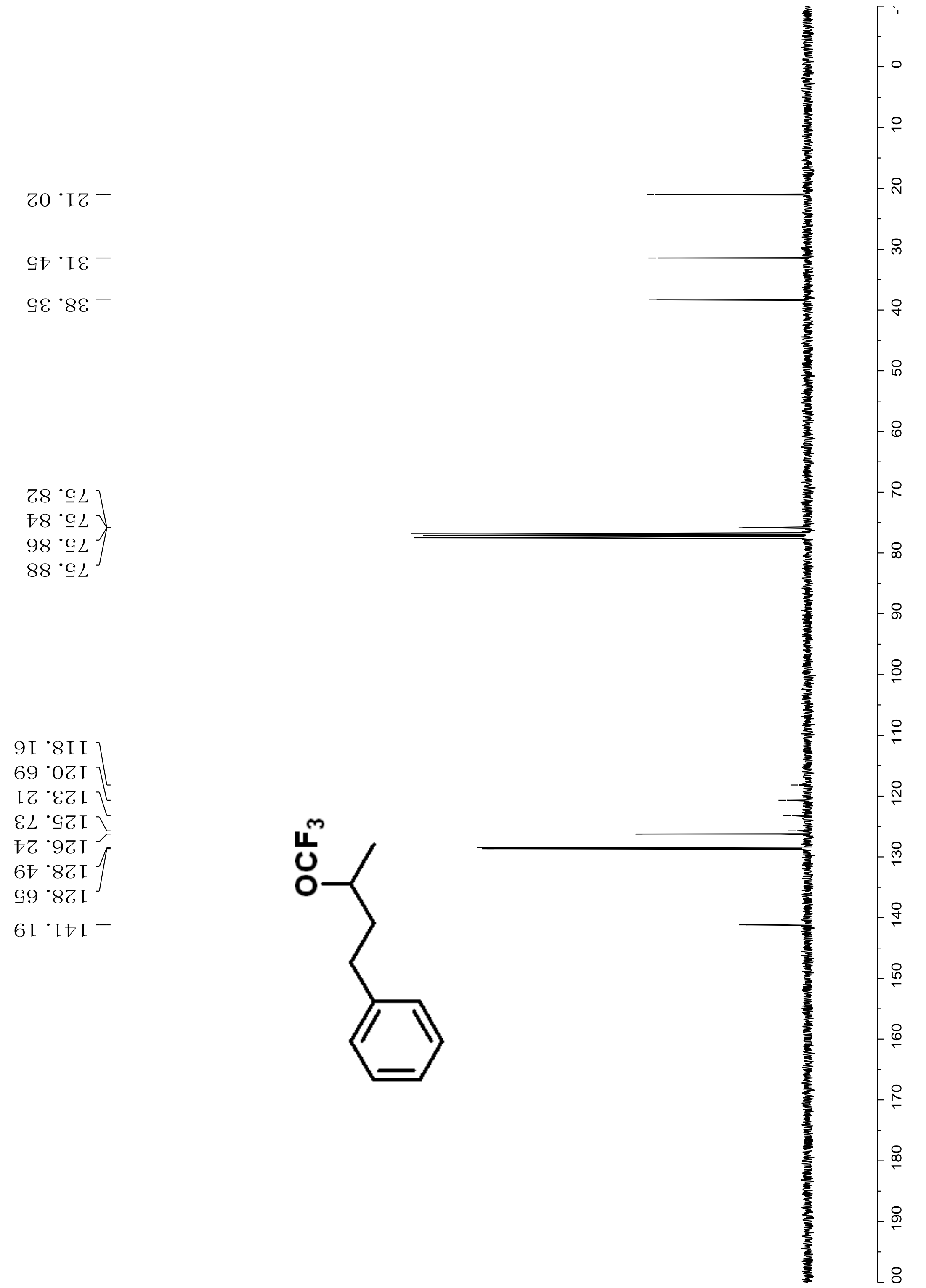

Supplementary Figure 196: ^{13}C NMR spectrum (101 MHz, $CDCl_3$, 23 °C) of **33**

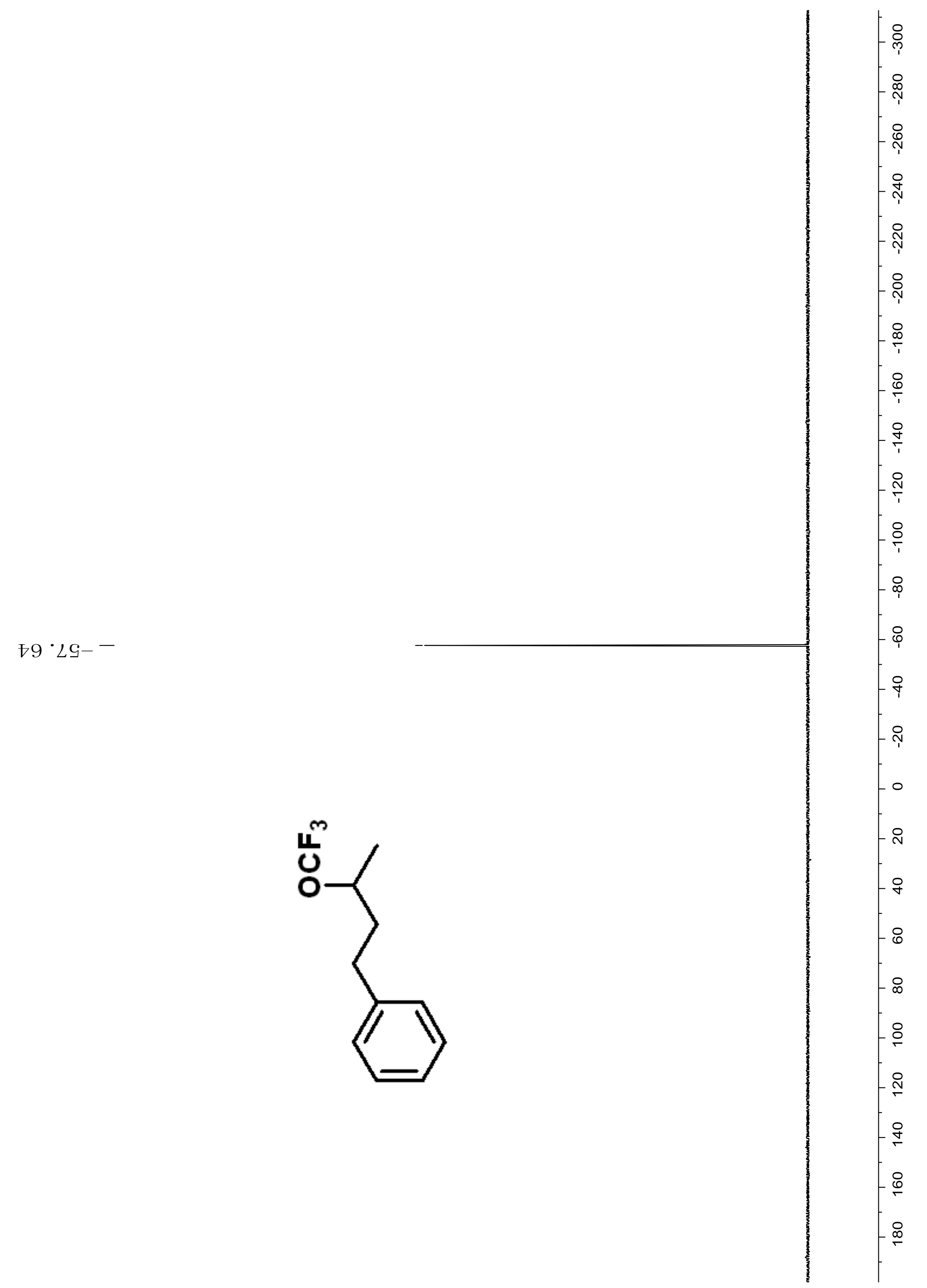

Supplementary Figure 197: ^{19}F NMR spectrum (376 MHz, $CDCl_3$, 23 °C) of **33**

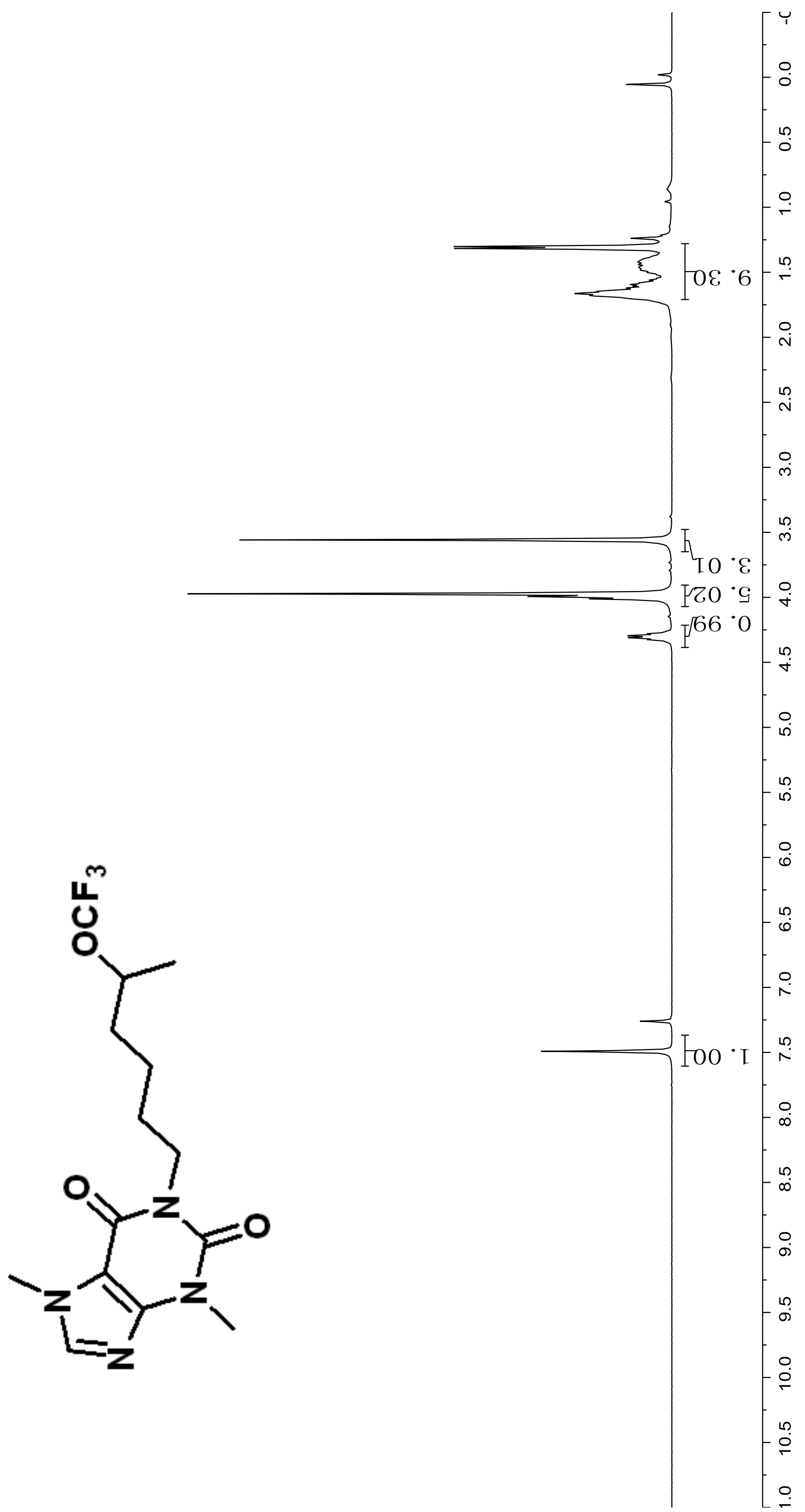

Supplementary Figure 198: ^{1}H NMR spectrum (400 MHz, $CDCl_3$, 23 °C) of **34**

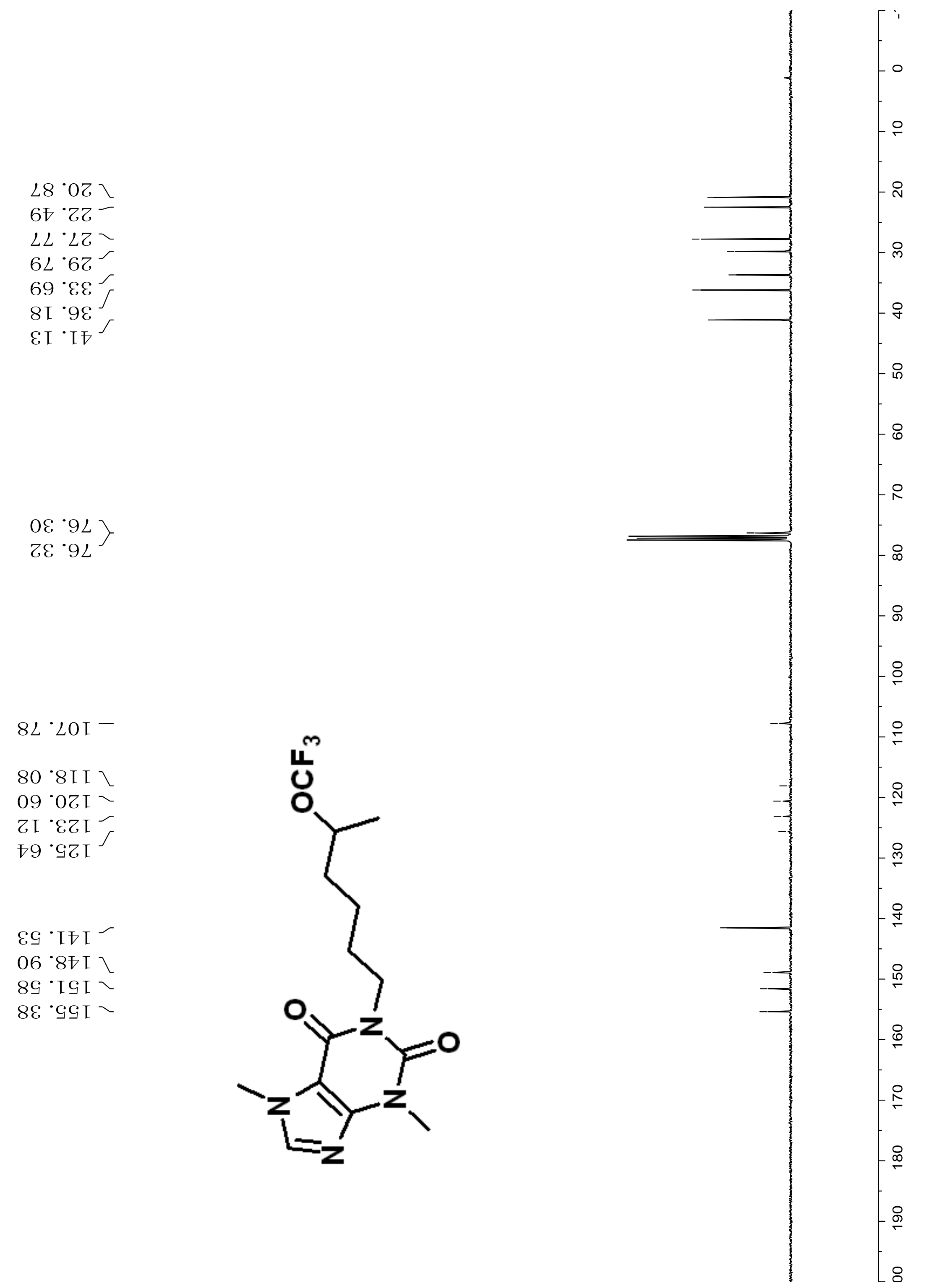

Supplementary Figure 199: ^{13}C NMR spectrum (101 MHz, $CDCl_3$, 23 °C) of **34**

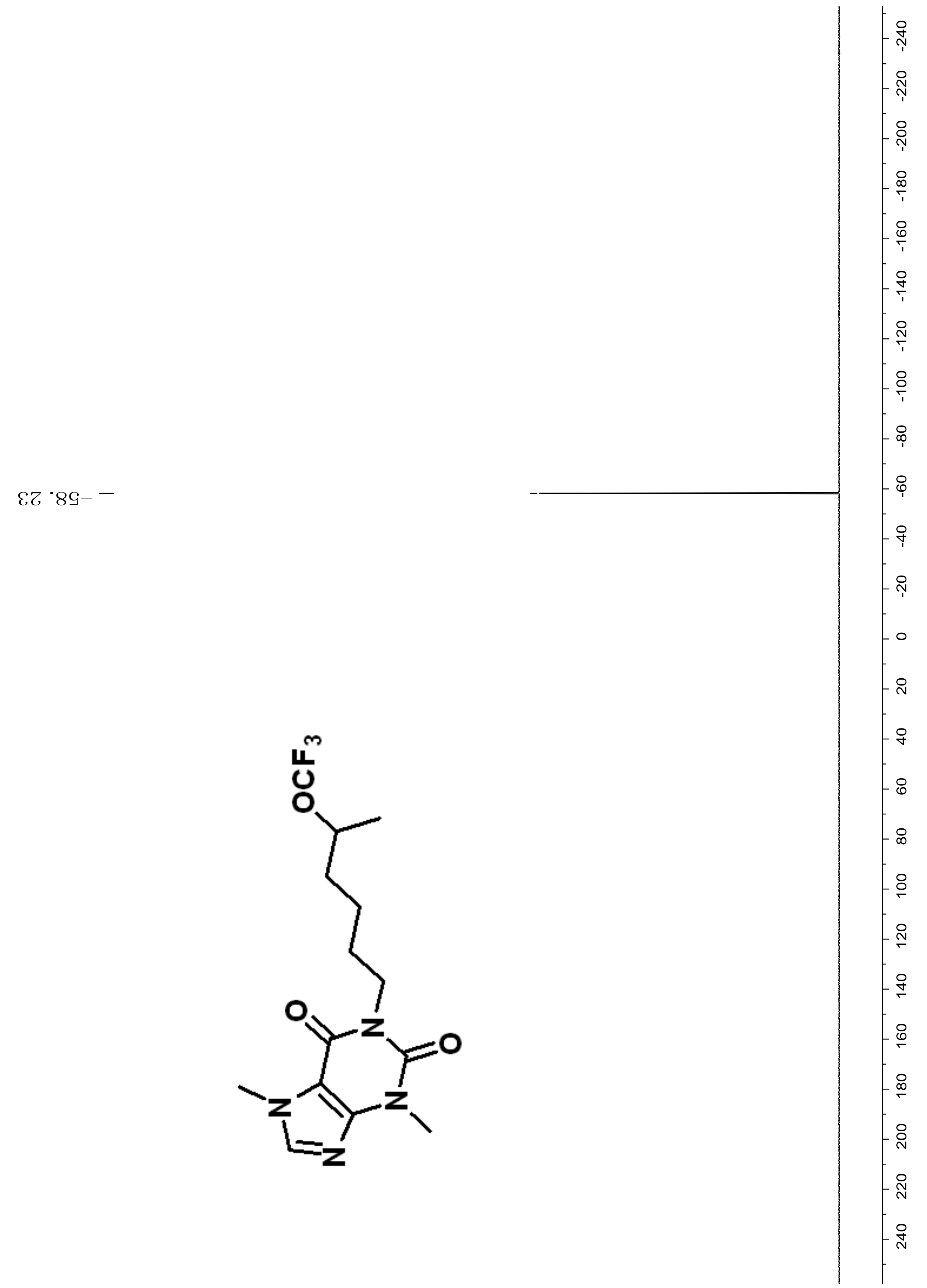

Supplementary Figure 200: ^{19}F NMR spectrum (376 MHz, DMSO, 23 °C) of **34**

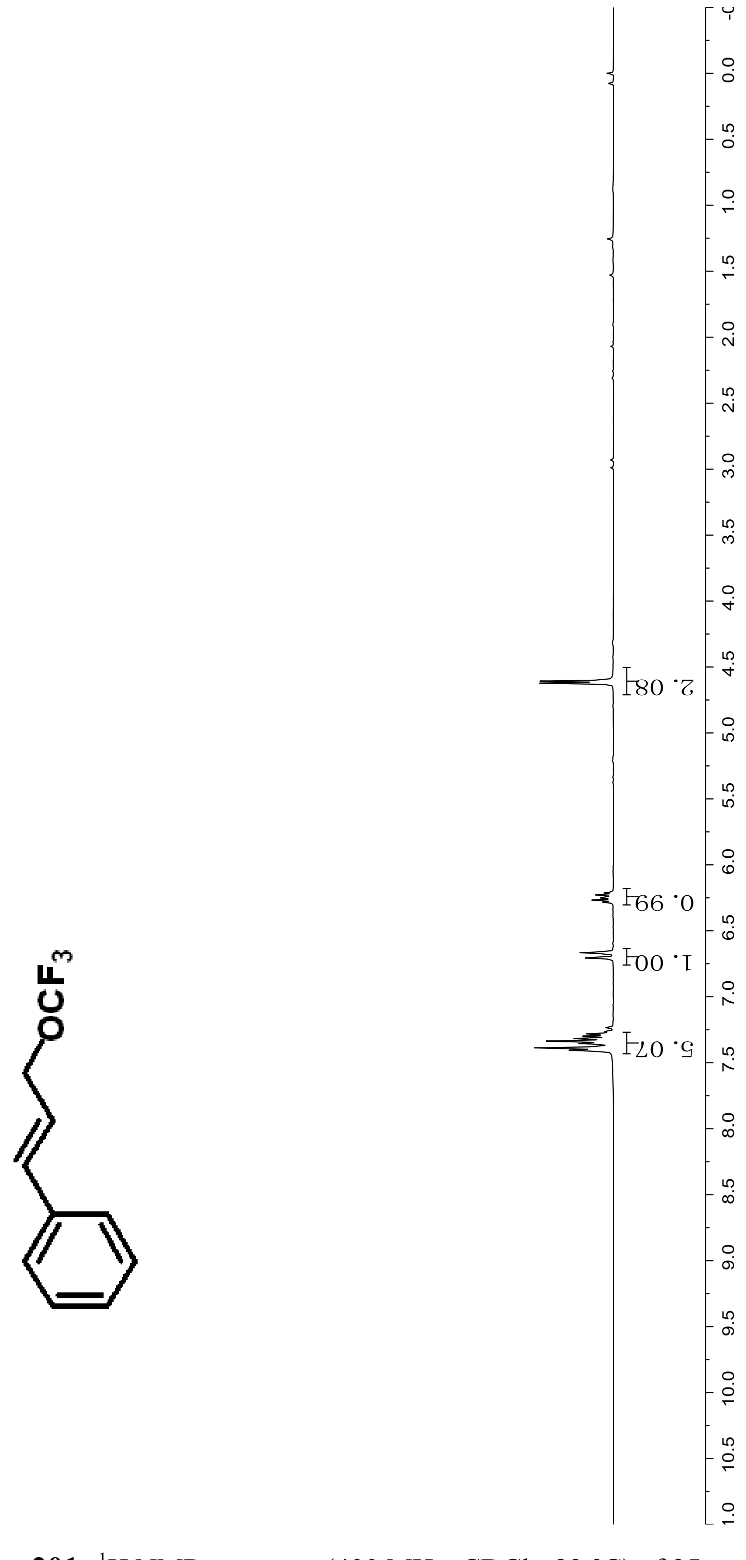

Supplementary Figure 201: ^{1}H NMR spectrum (400 MHz, $CDCl_3$, 23 °C) of **35**

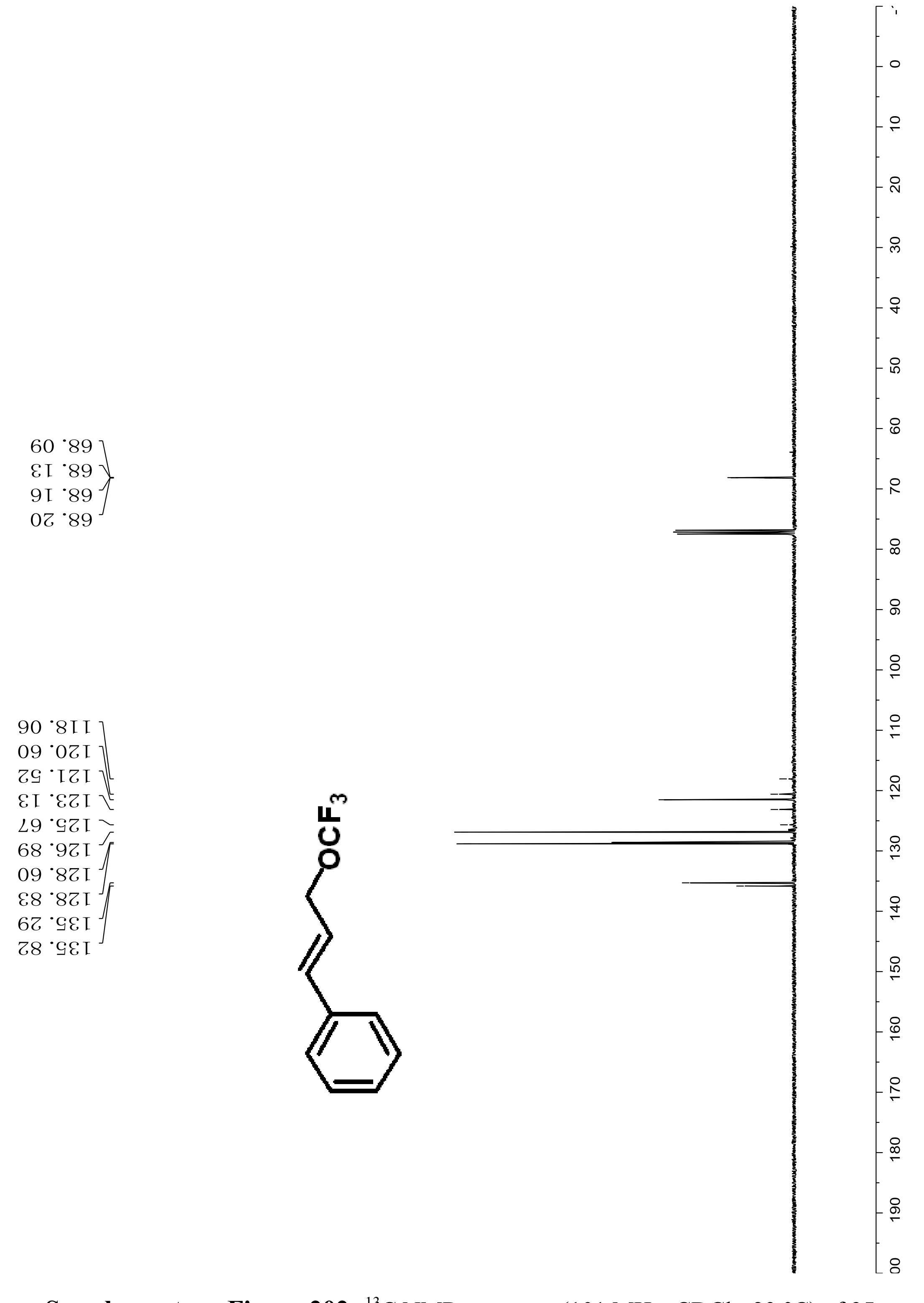

Supplementary Figure 202: ^{13}C NMR spectrum (101 MHz, $CDCl_3$, 23 °C) of **35**

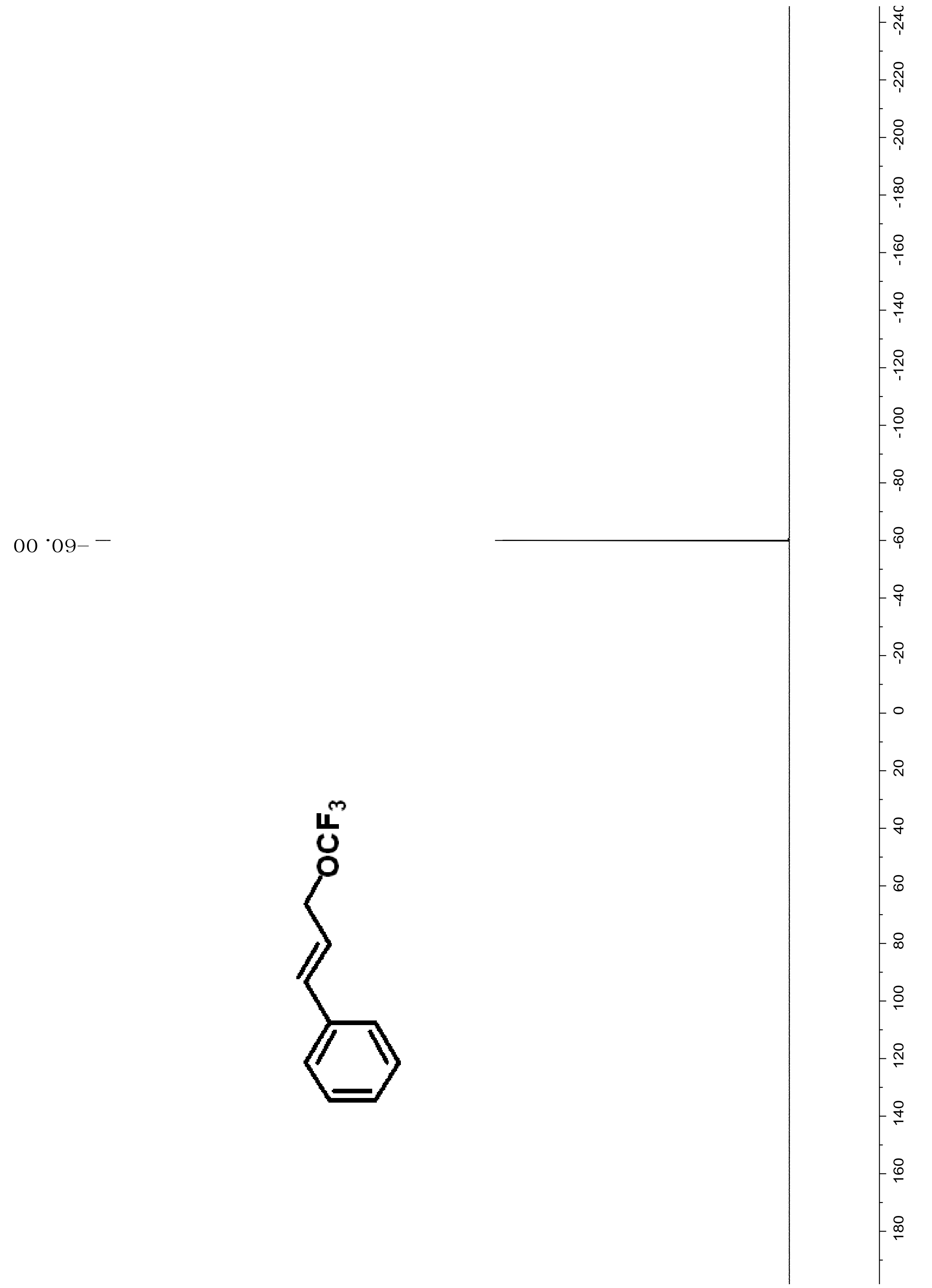

Supplementary Figure 203: ^{19}F NMR spectrum (376 MHz, $CDCl_3$, 23 °C) of **35**

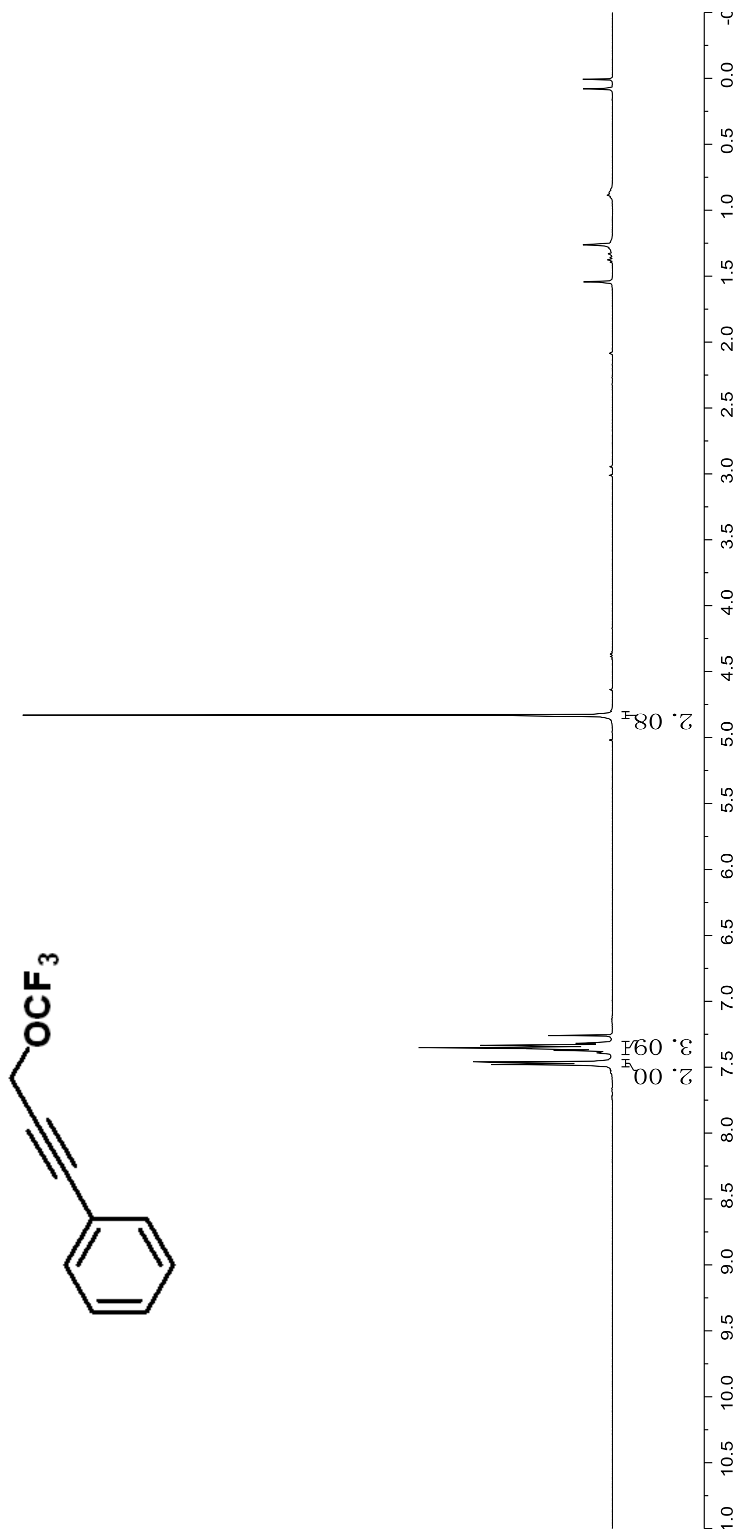

Supplementary Figure 204: ^{1}H NMR spectrum (400 MHz, $CDCl_3$, 23 °C) of **36**

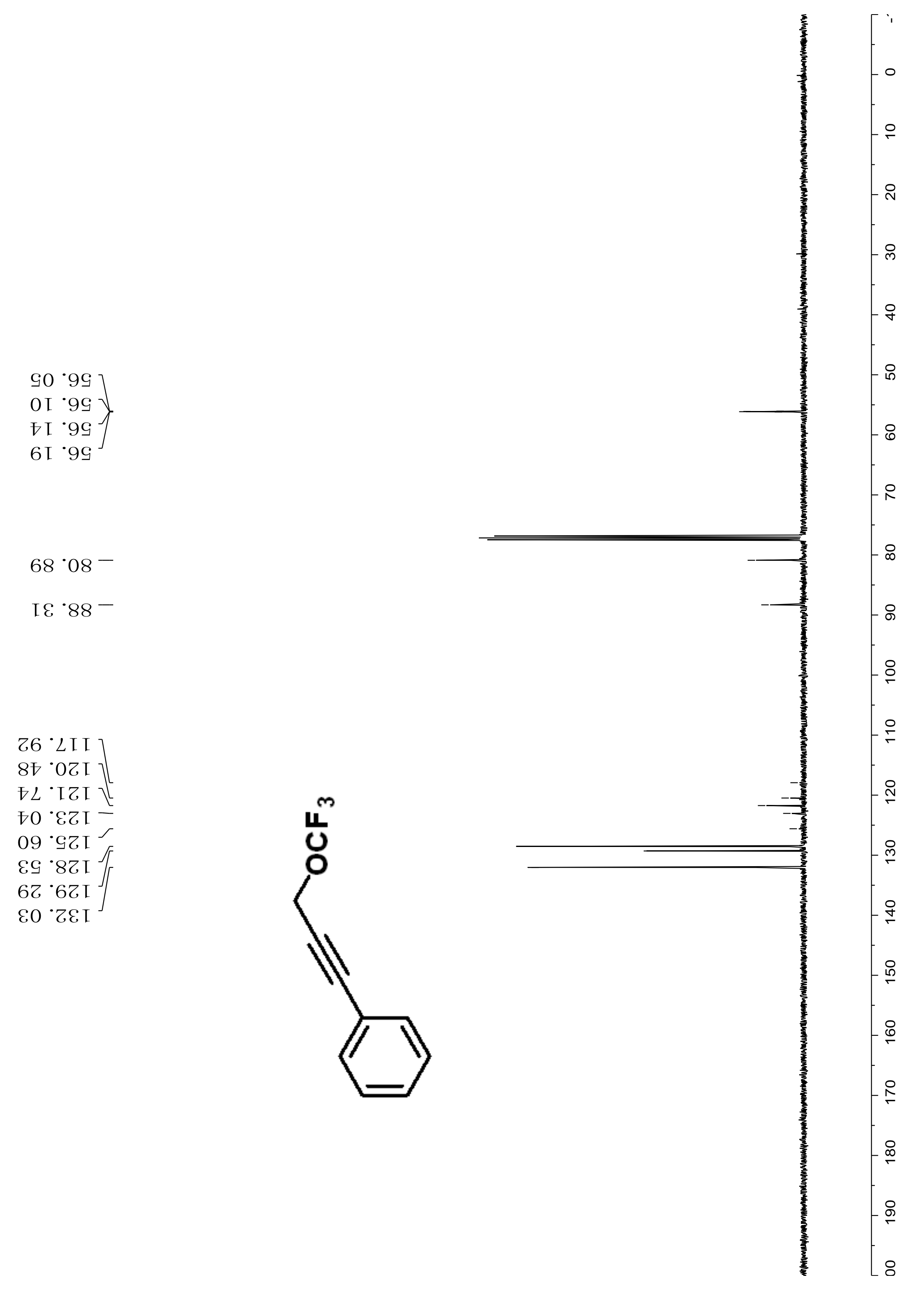

Supplementary Figure 205: ^{13}C NMR spectrum (101 MHz, $CDCl_3$, 23 °C) of **36**

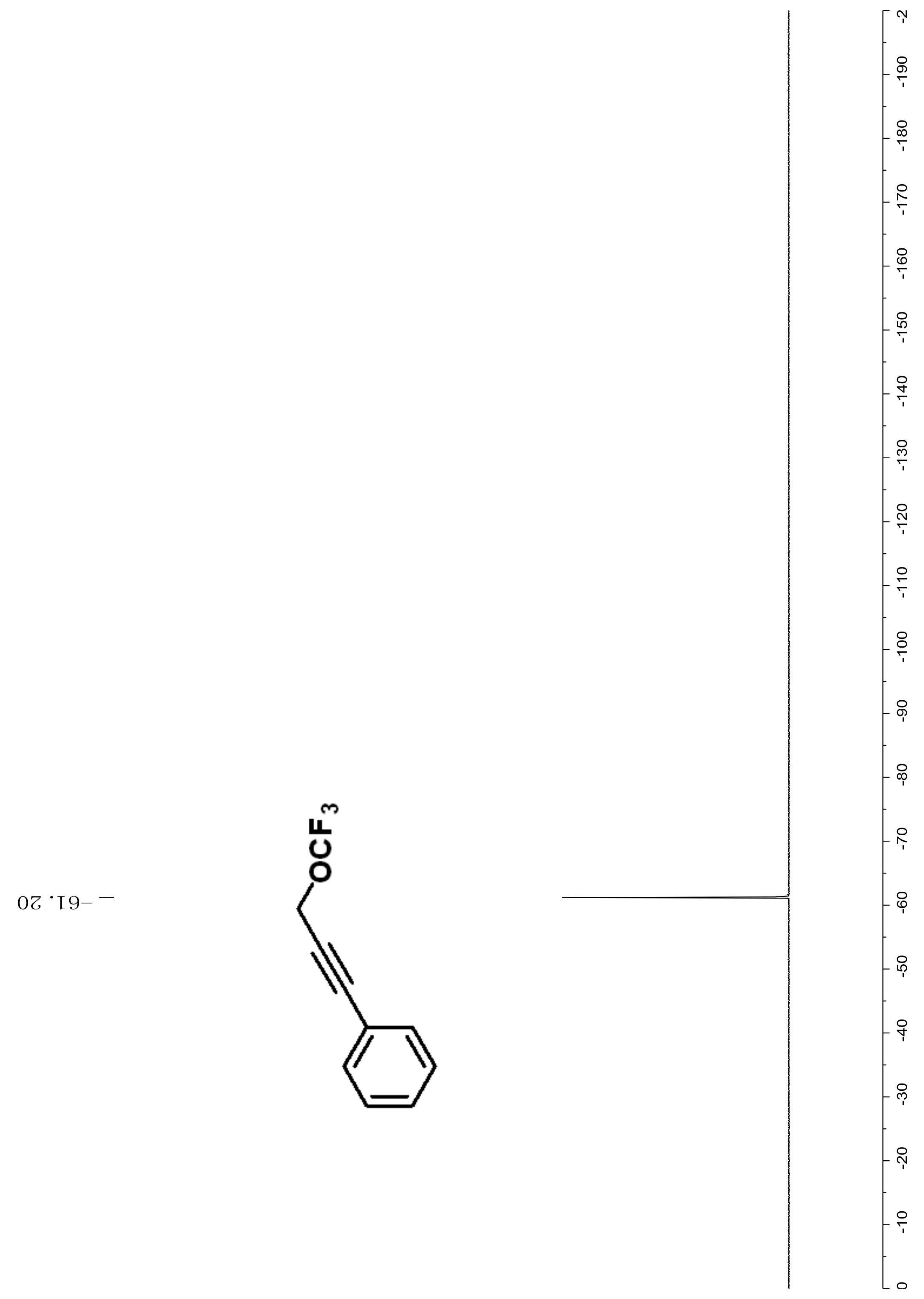

Supplementary Figure 206: ^{19}F NMR spectrum (376 MHz, $CDCl_3$, 23 °C) of **36**

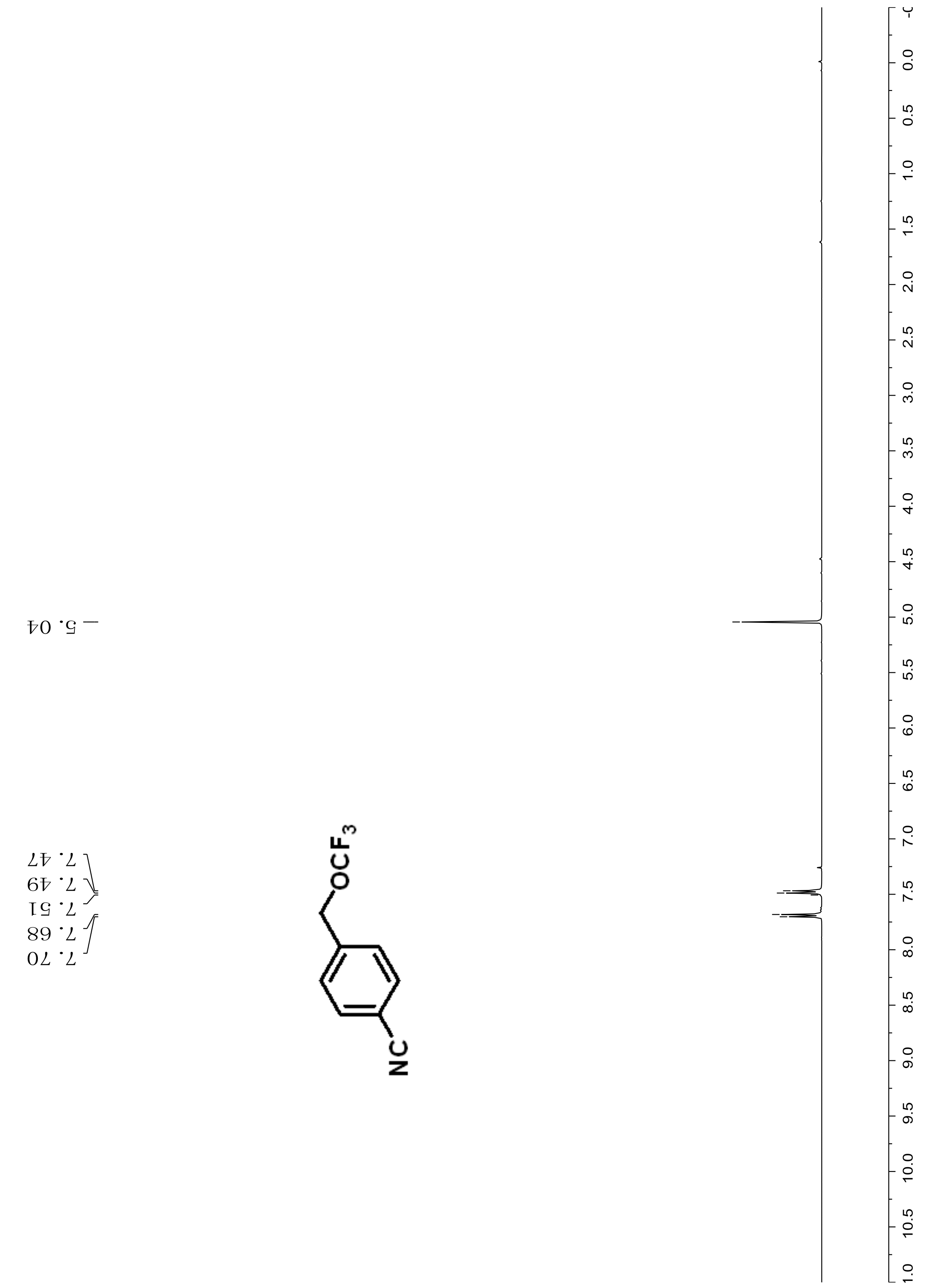

Supplementary Figure 207: ^{1}H NMR spectrum (400 MHz, $CDCl_3$, 23 °C) of **37**

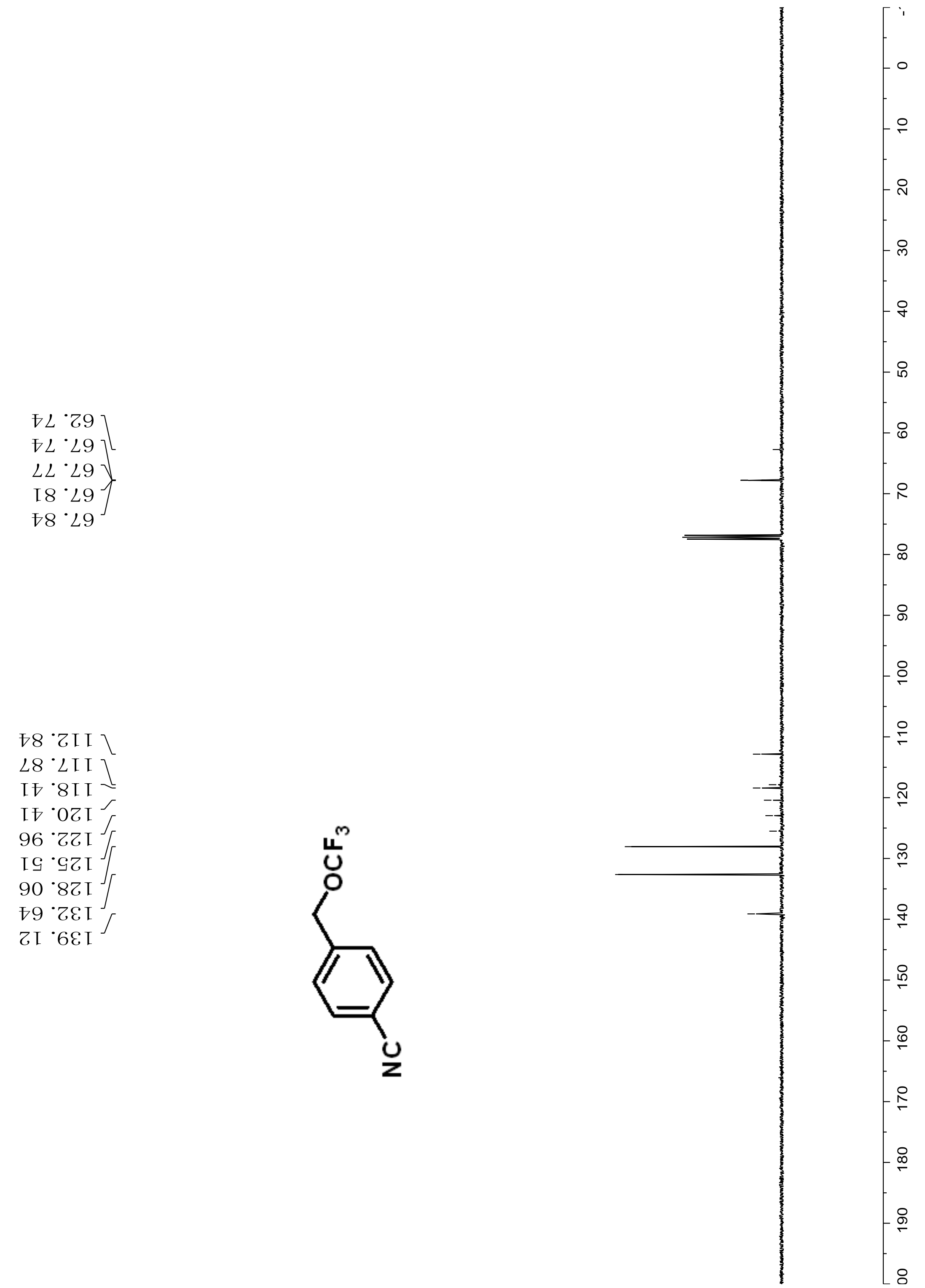

Supplementary Figure 208: ^{13}C NMR spectrum (101 MHz, $CDCl_3$, 23 °C) of **37**

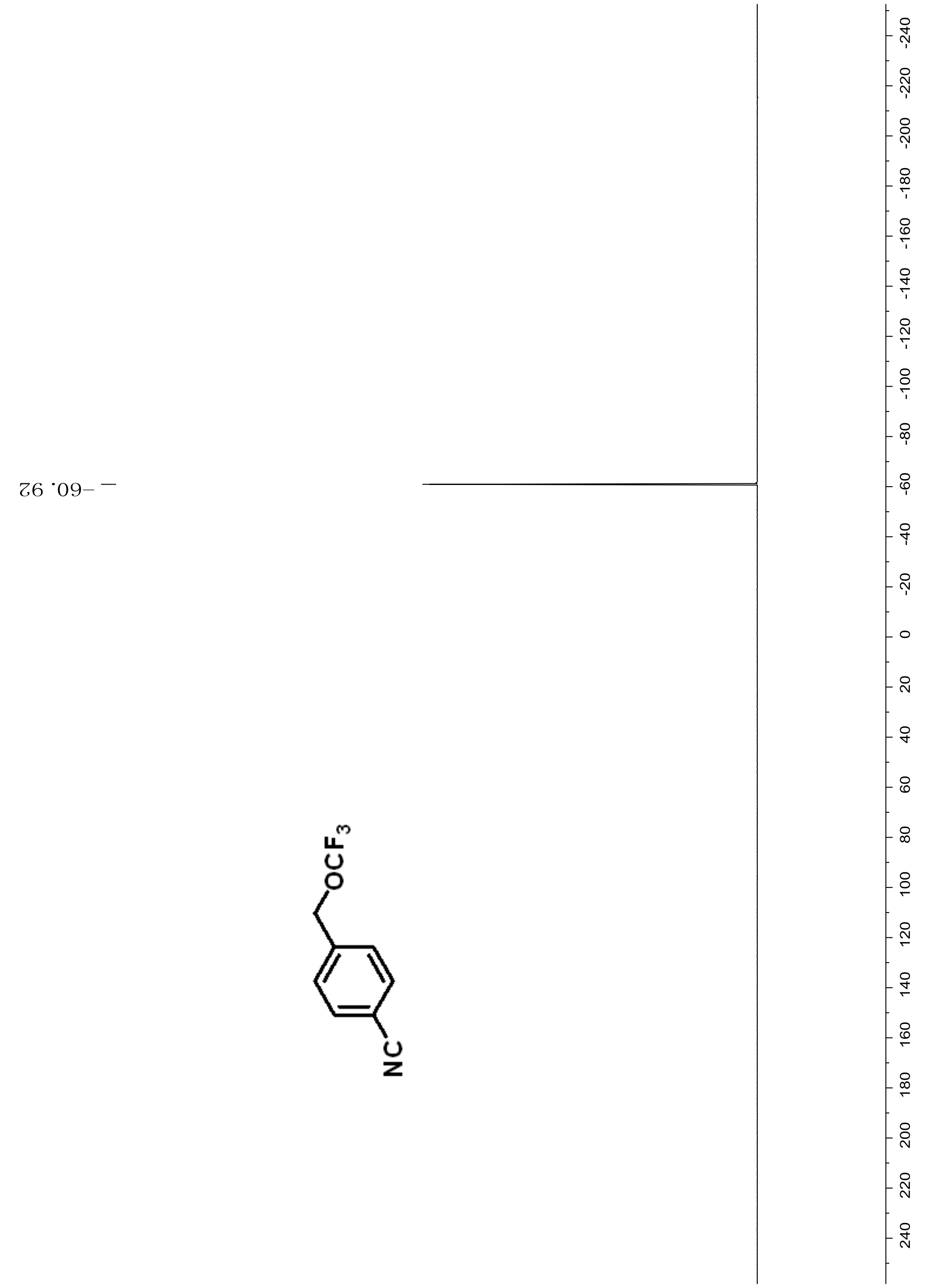

Supplementary Figure 209: ^{19}F NMR spectrum (376 MHz, $CDCl_3$, 23 °C) of **37**

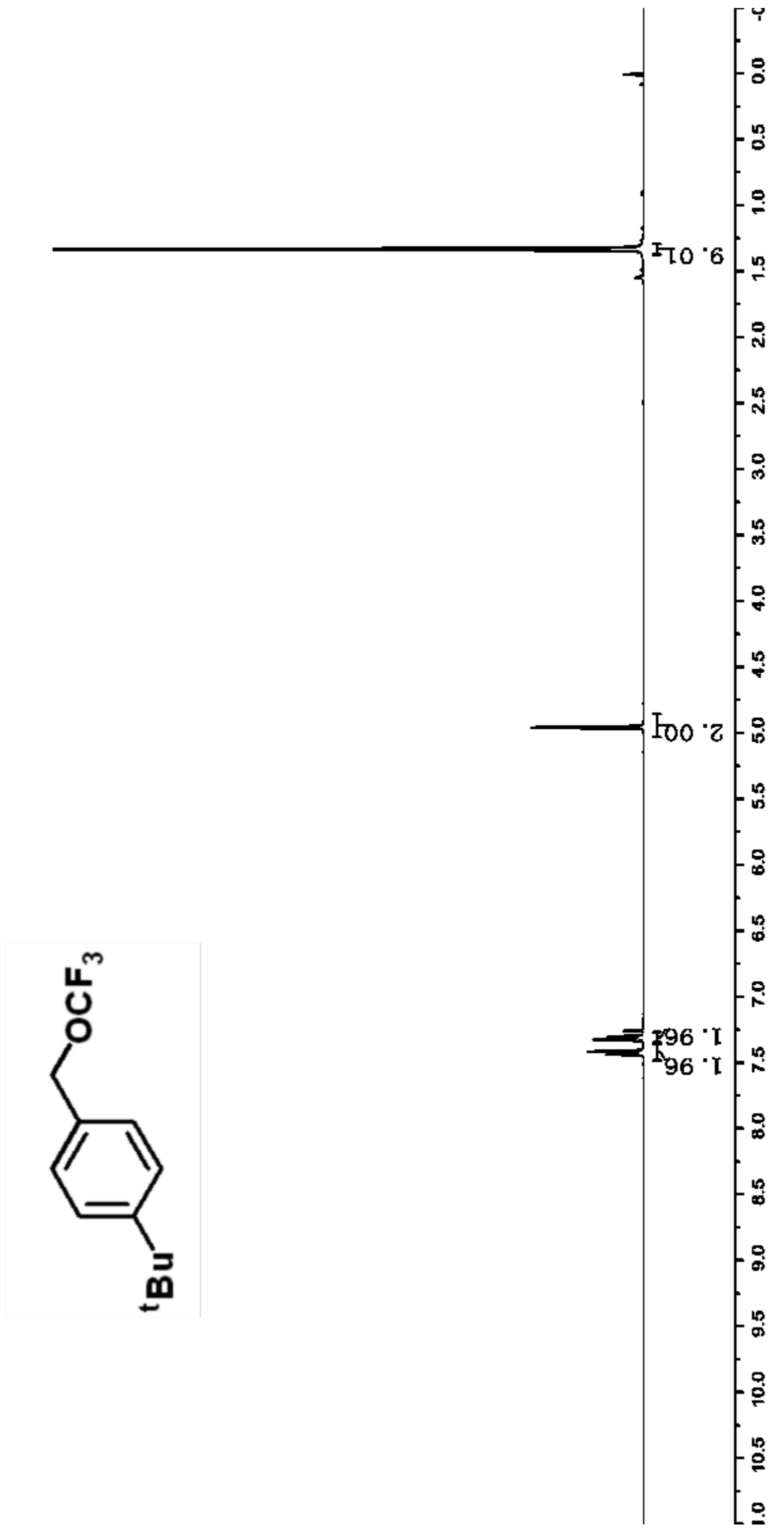

Supplementary Figure 210: ^{1}H NMR spectrum (400 MHz, $CDCl_3$, 23 °C) of **38**

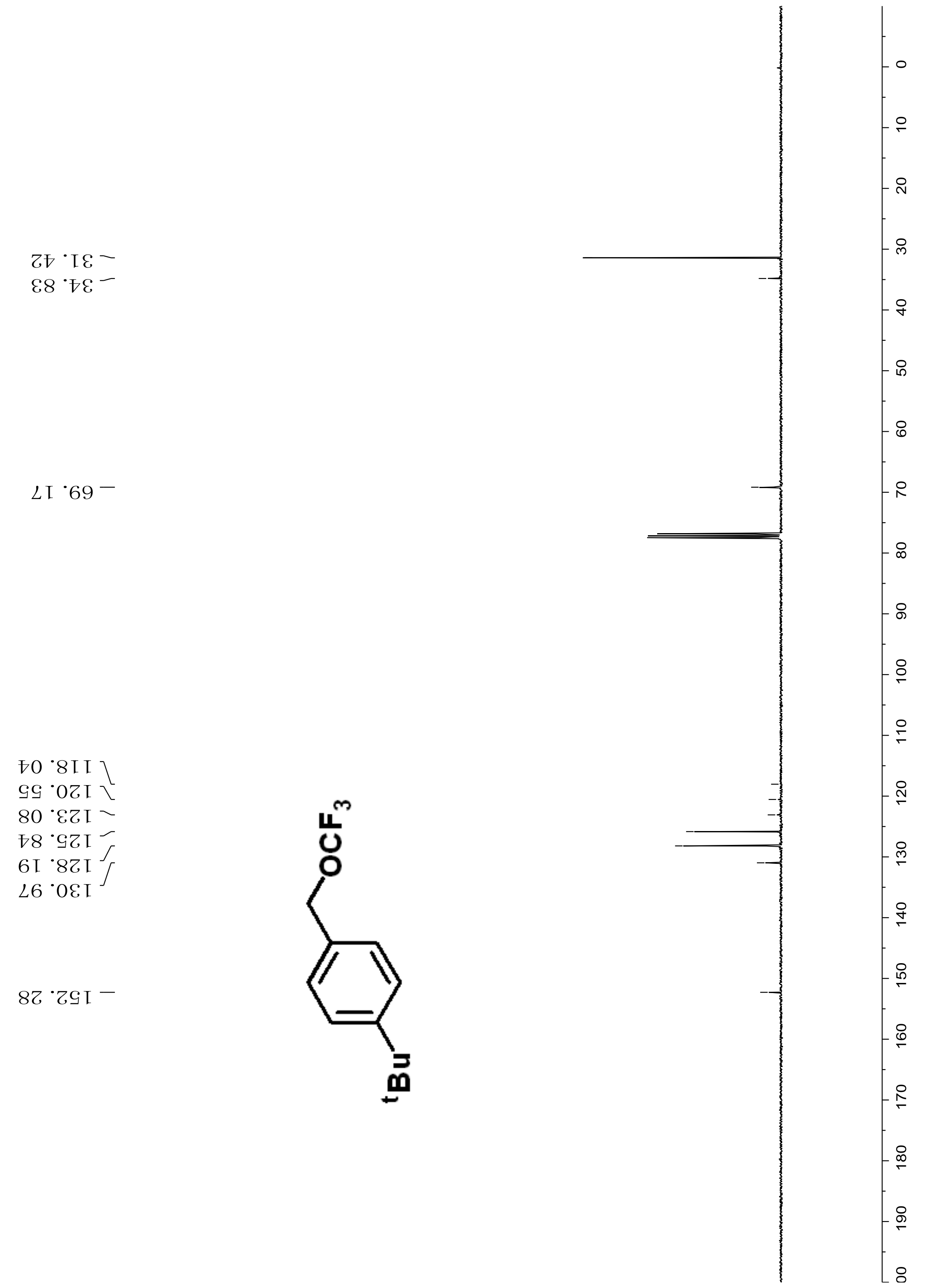

Supplementary Figure 211: ^{13}C NMR spectrum (101 MHz, $CDCl_3$, 23 °C) of **38**

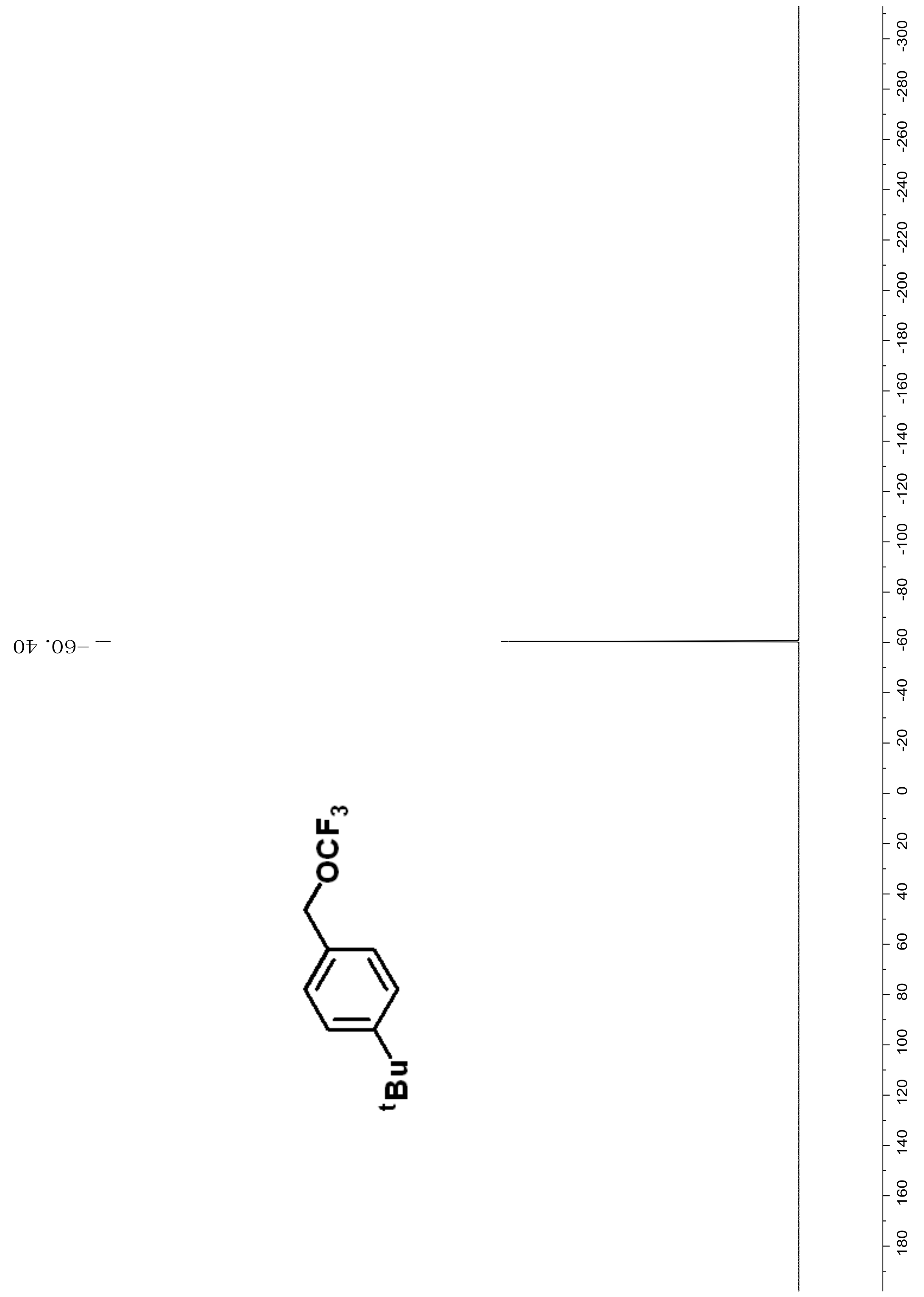

Supplementary Figure 212: ^{19}F NMR spectrum (376 MHz, $CDCl_3$, 23 °C) of **38**

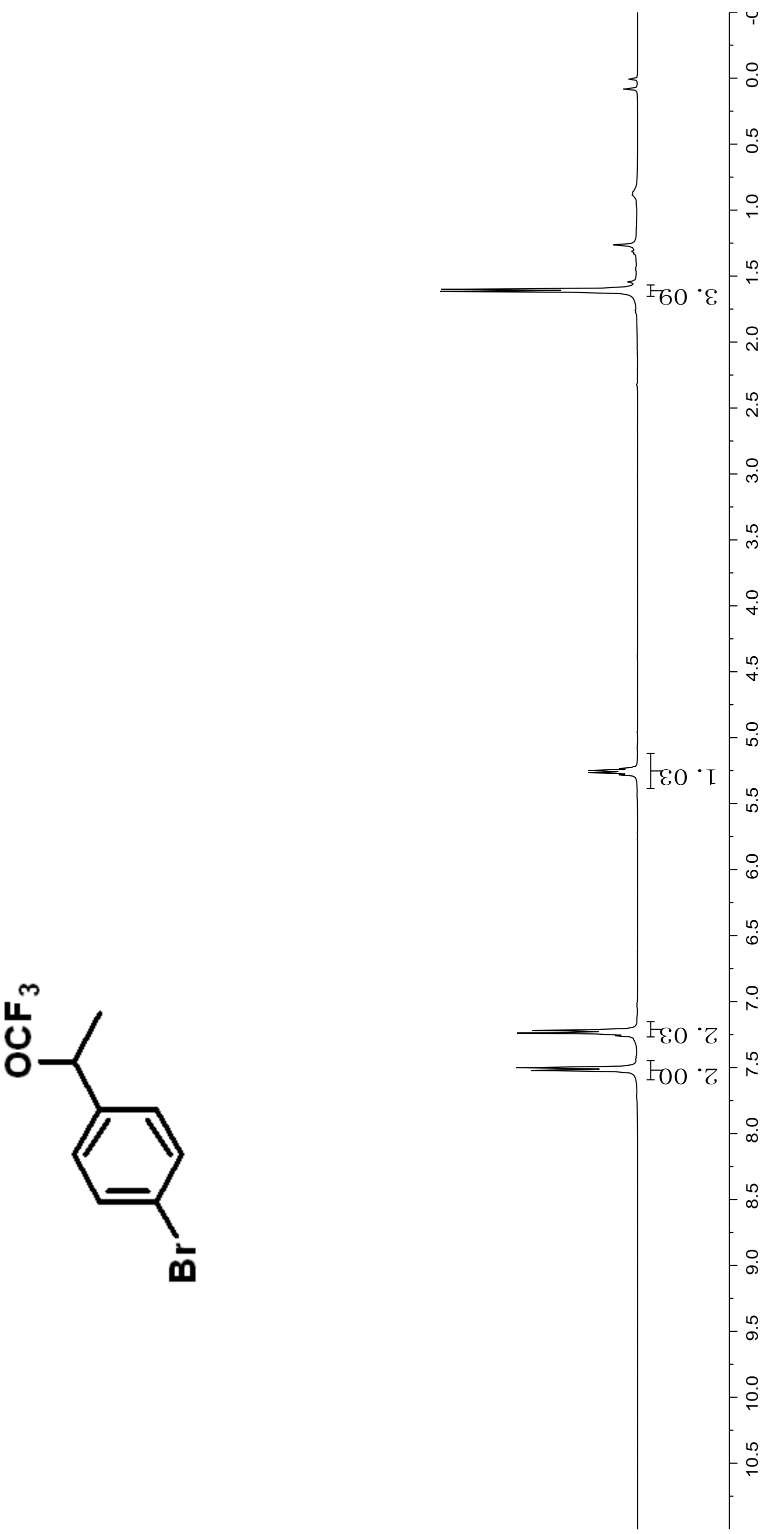

Supplementary Figure 213: ^{1}H NMR spectrum (400 MHz, $CDCl_3$, 23 °C) of **39**

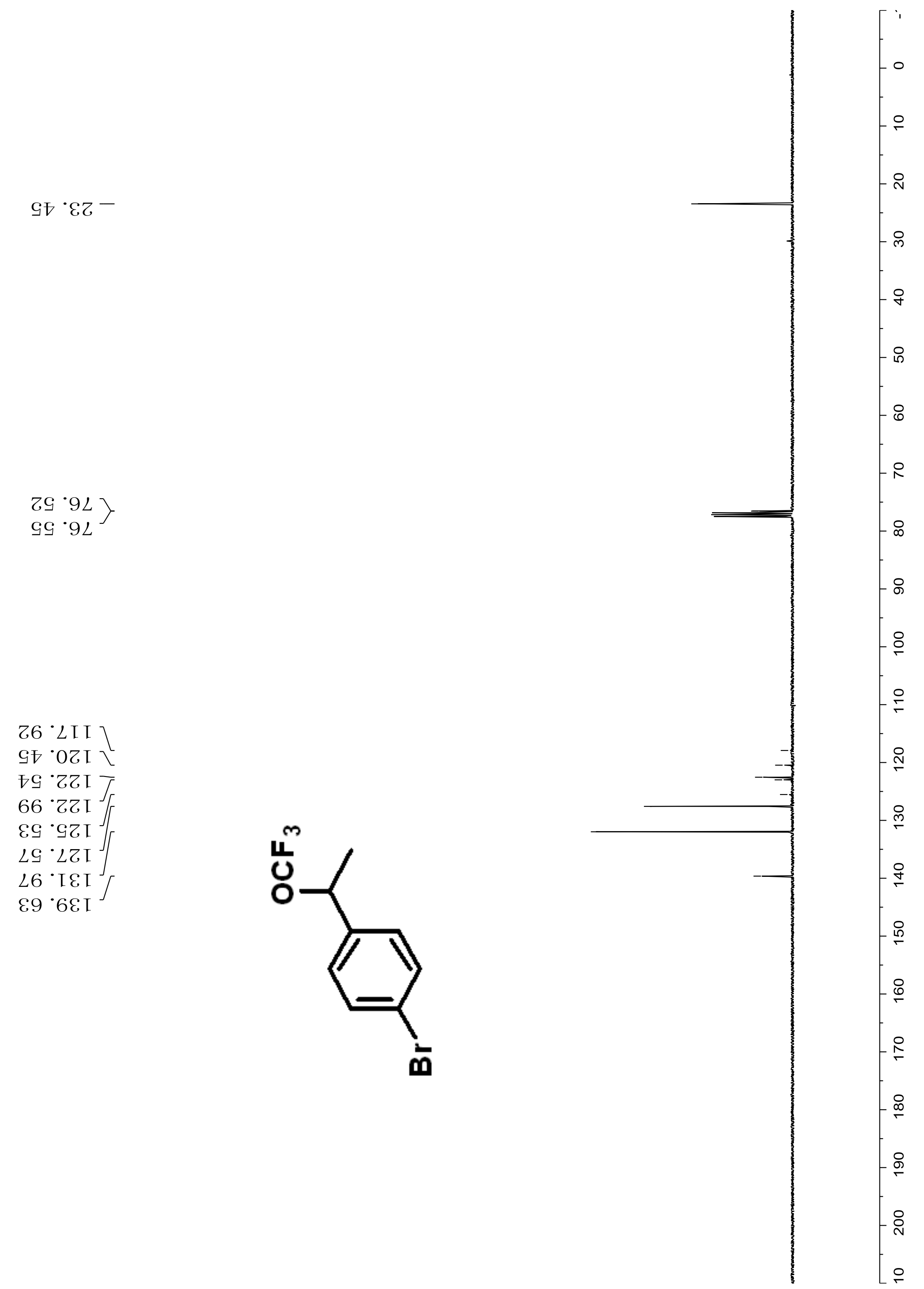

Supplementary Figure 214: ^{13}C NMR spectrum (101 MHz, $CDCl_3$, 23 °C) of **39**

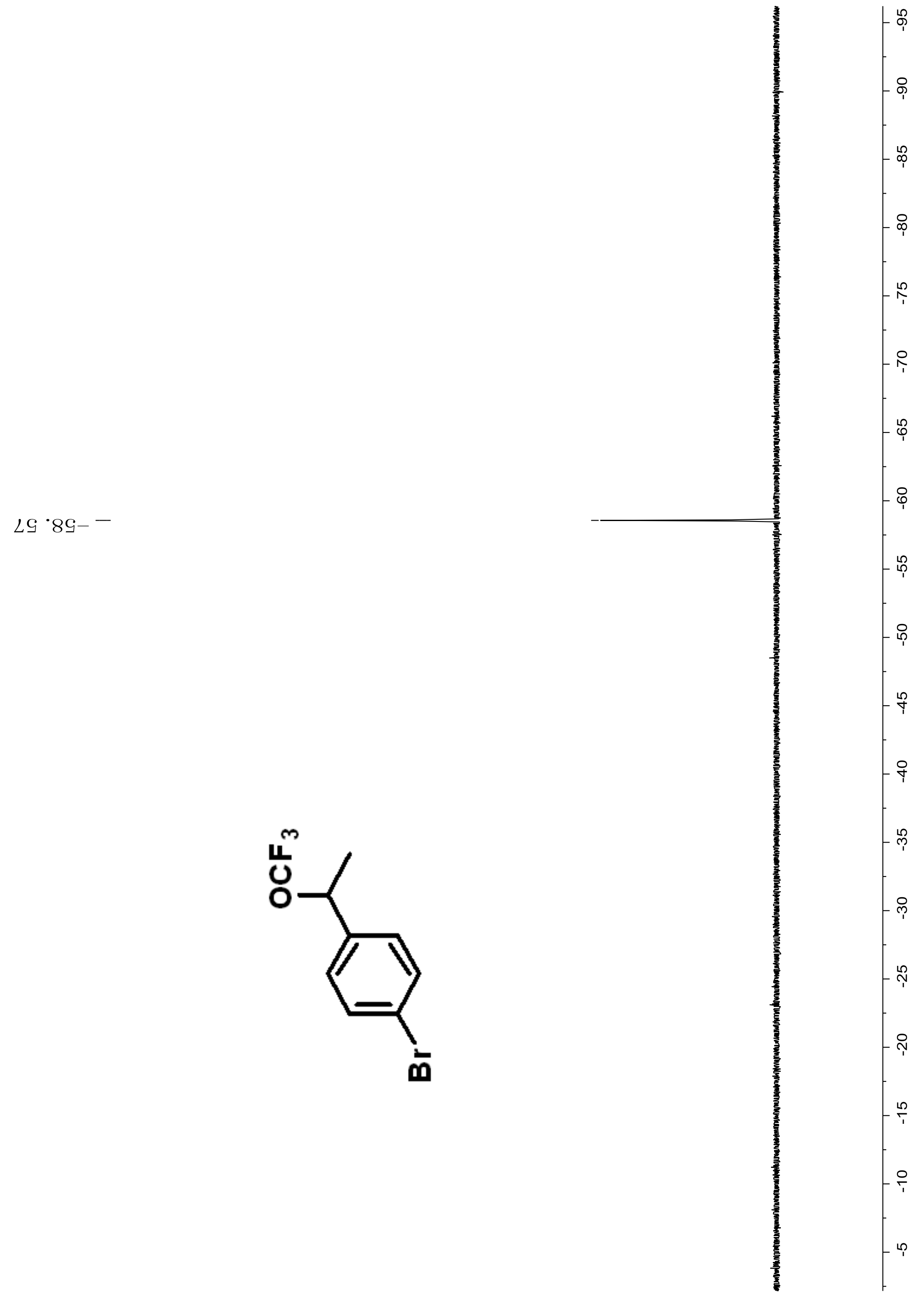

Supplementary Figure 215: ^{19}F NMR spectrum (376 MHz, DMSO, 23 °C) of **39**

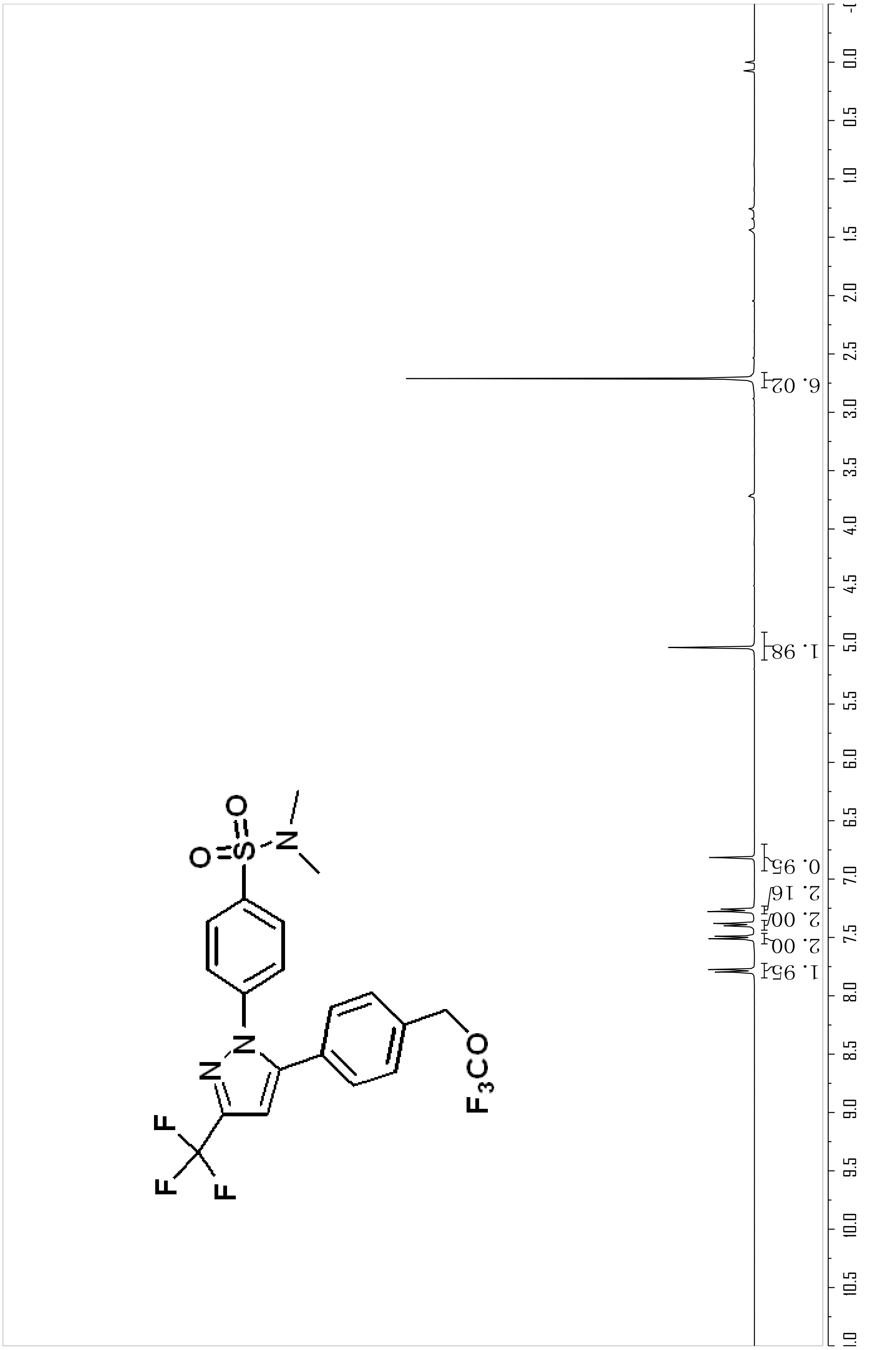

Supplementary Figure 216: ^{1}H NMR spectrum (400 MHz, $CDCl_3$, 23 °C) of **40**

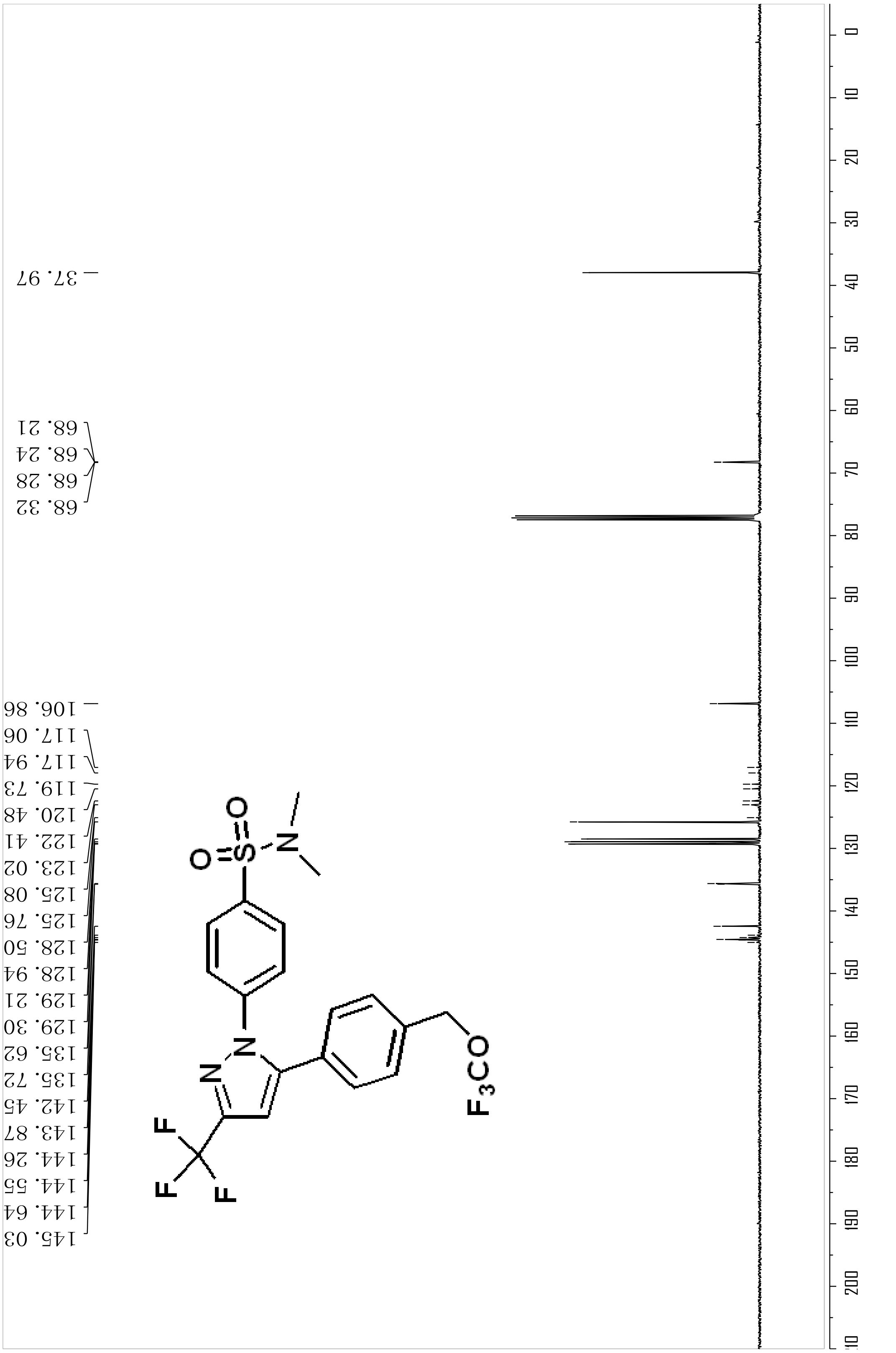

Supplementary Figure 217: ^{13}C NMR spectrum (101 MHz, $CDCl_3$, 23 °C) of **40**

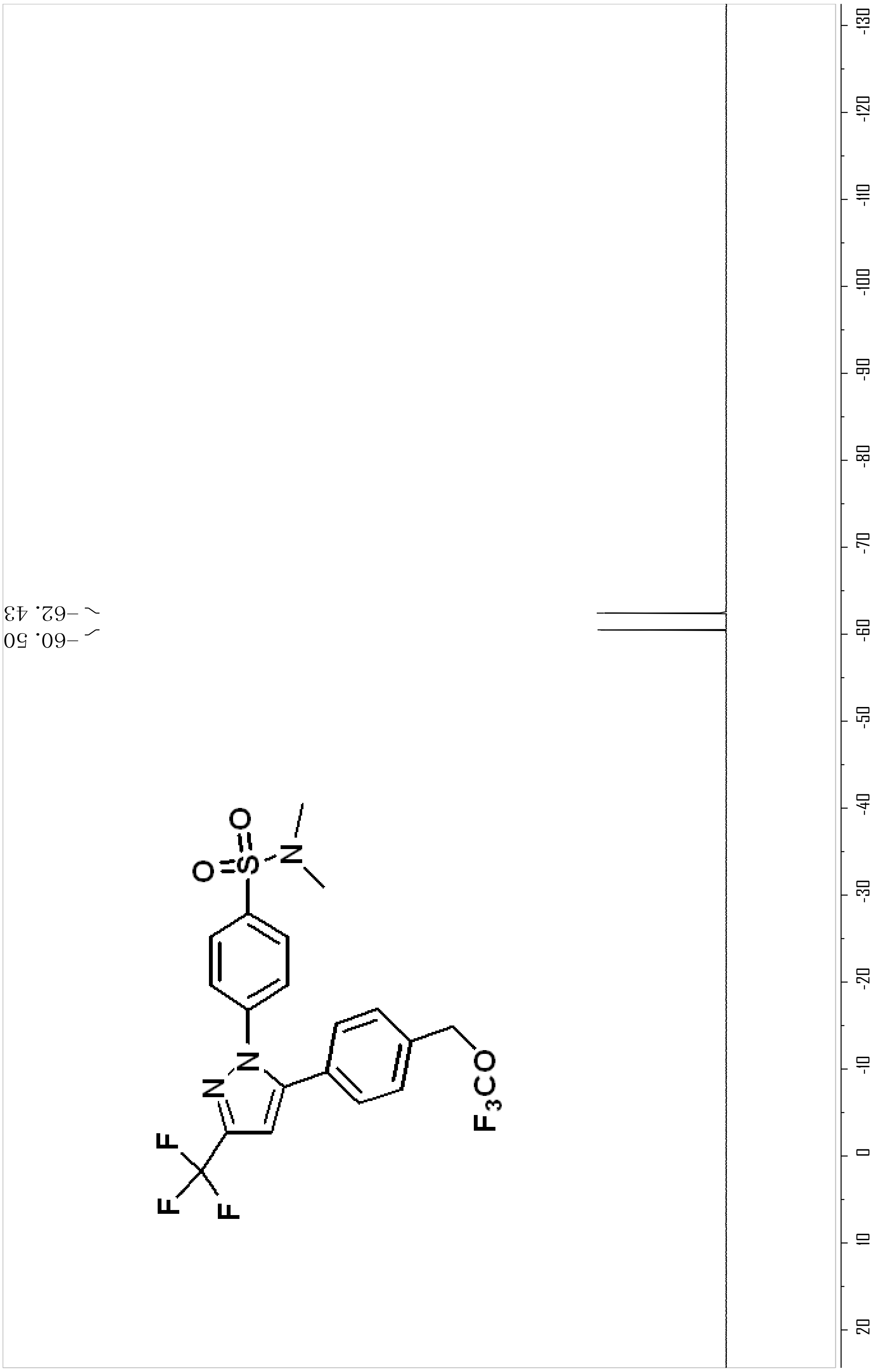

Supplementary Figure 218: ^{19}F NMR spectrum (376 MHz, $CDCl_3$, 23 °C) of **40**

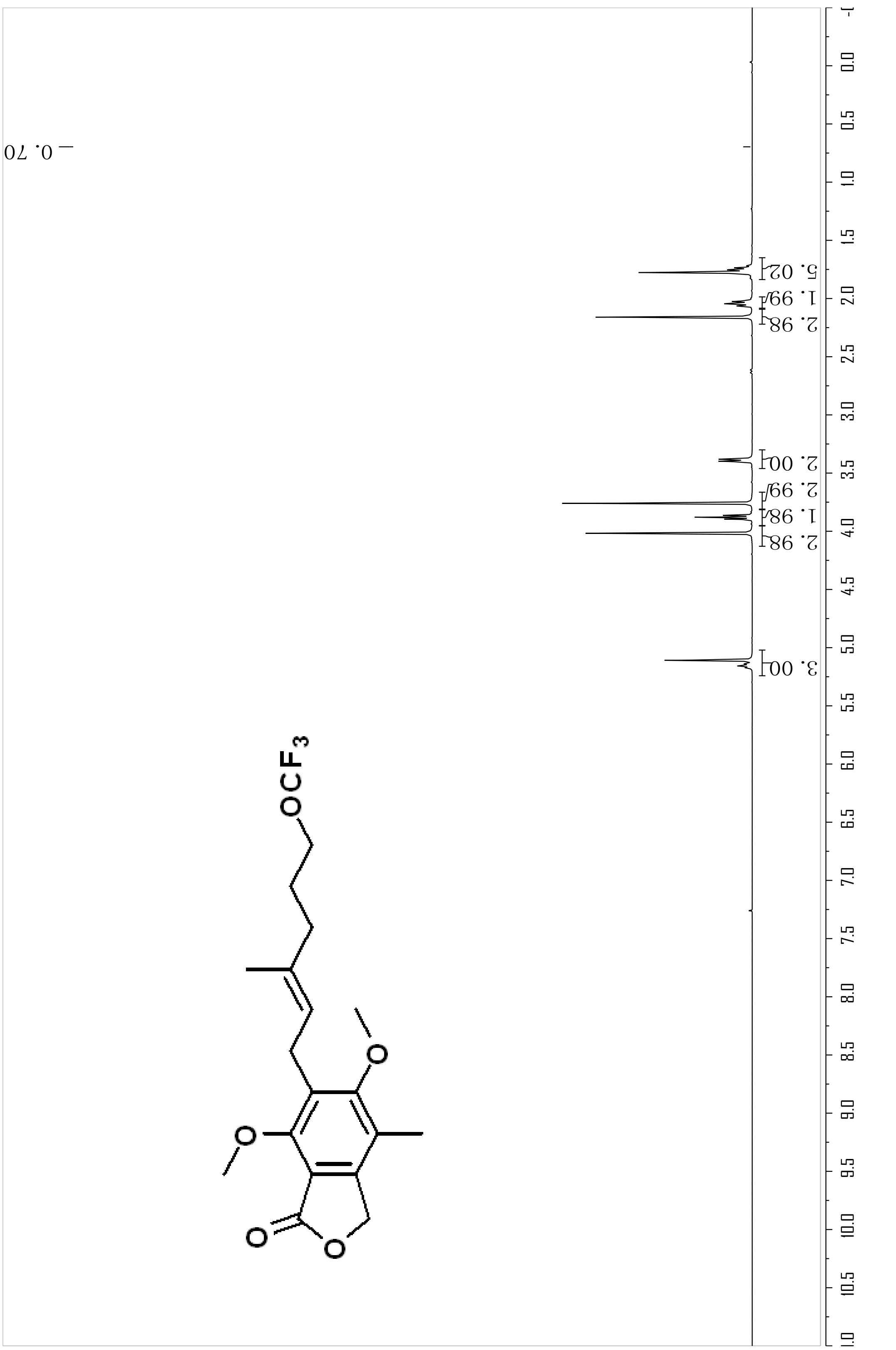

Supplementary Figure 219: ^{1}H NMR spectrum (400 MHz, $CDCl_3$, 23 °C) of **41**

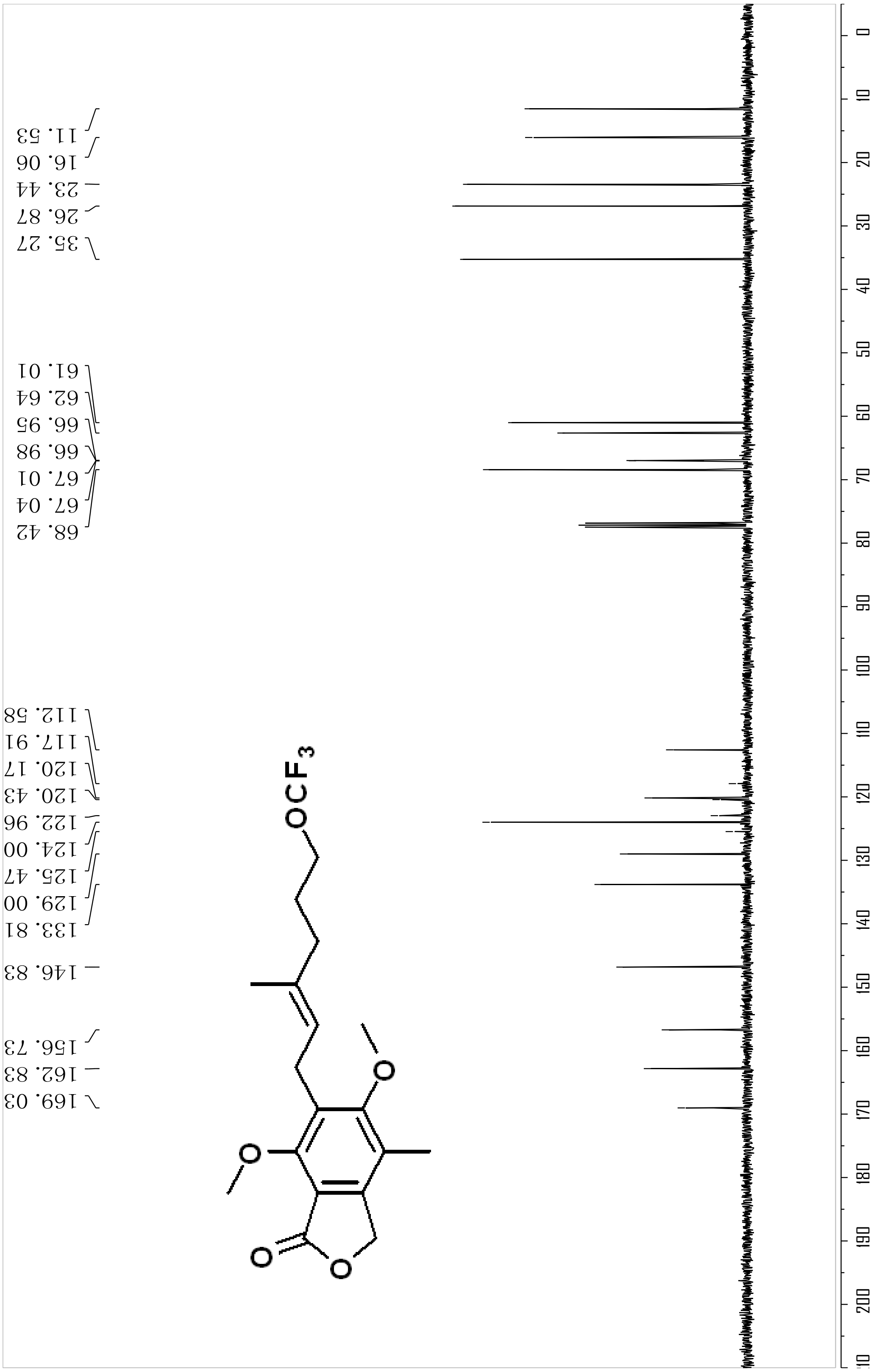

Supplementary Figure 220: ^{13}C NMR spectrum (101 MHz, $CDCl_3$, 23 °C) of **41**

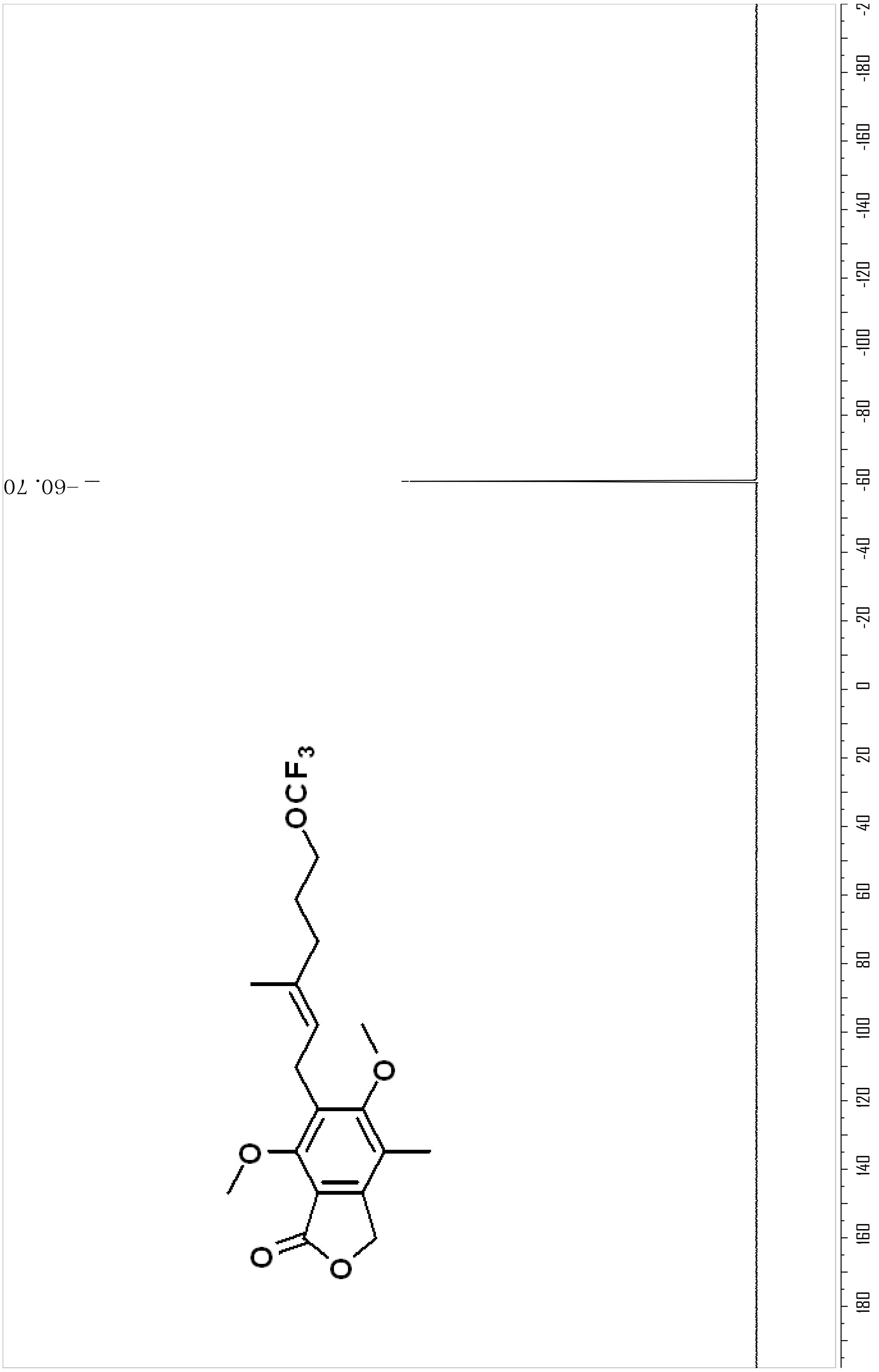

Supplementary Figure 221: ^{19}F NMR spectrum (376 MHz, $CDCl_3$, 23 °C) of **41**

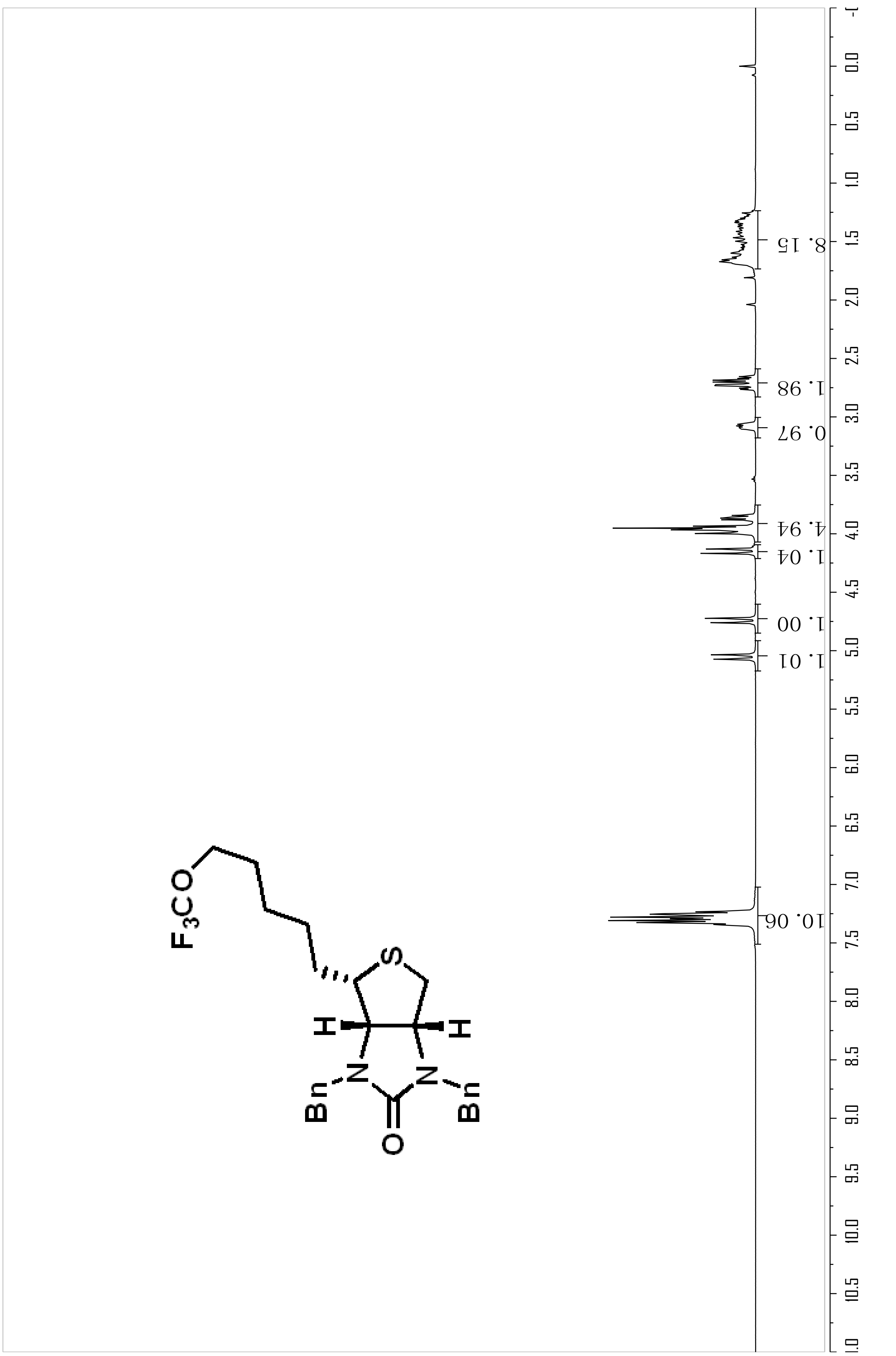

Supplementary Figure 222: ^{1}H NMR spectrum (400 MHz, $CDCl_3$, 23 °C) of **42**

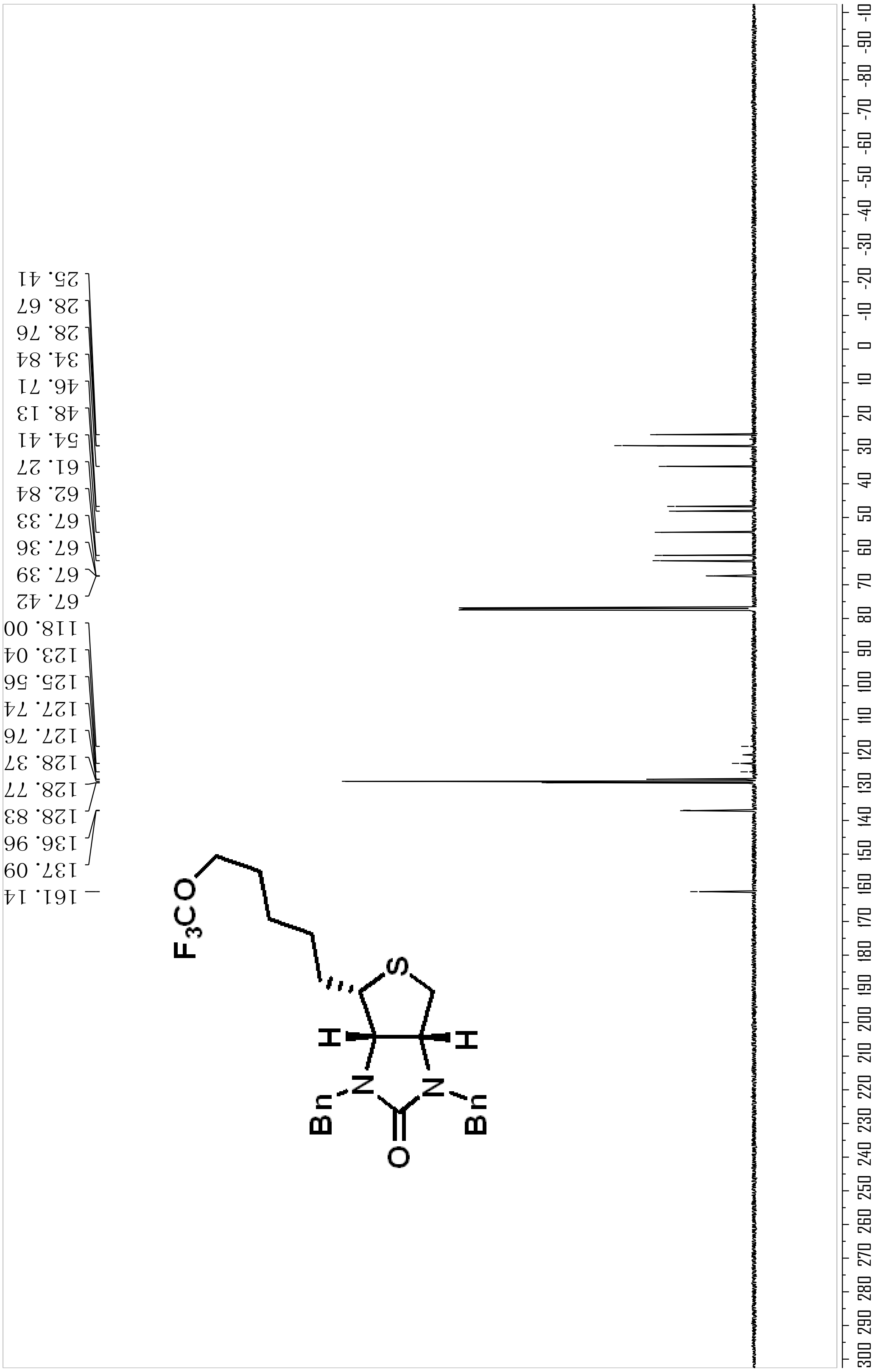

Supplementary Figure 223: ^{13}C NMR spectrum (101 MHz, $CDCl_3$, 23 °C) of **42**

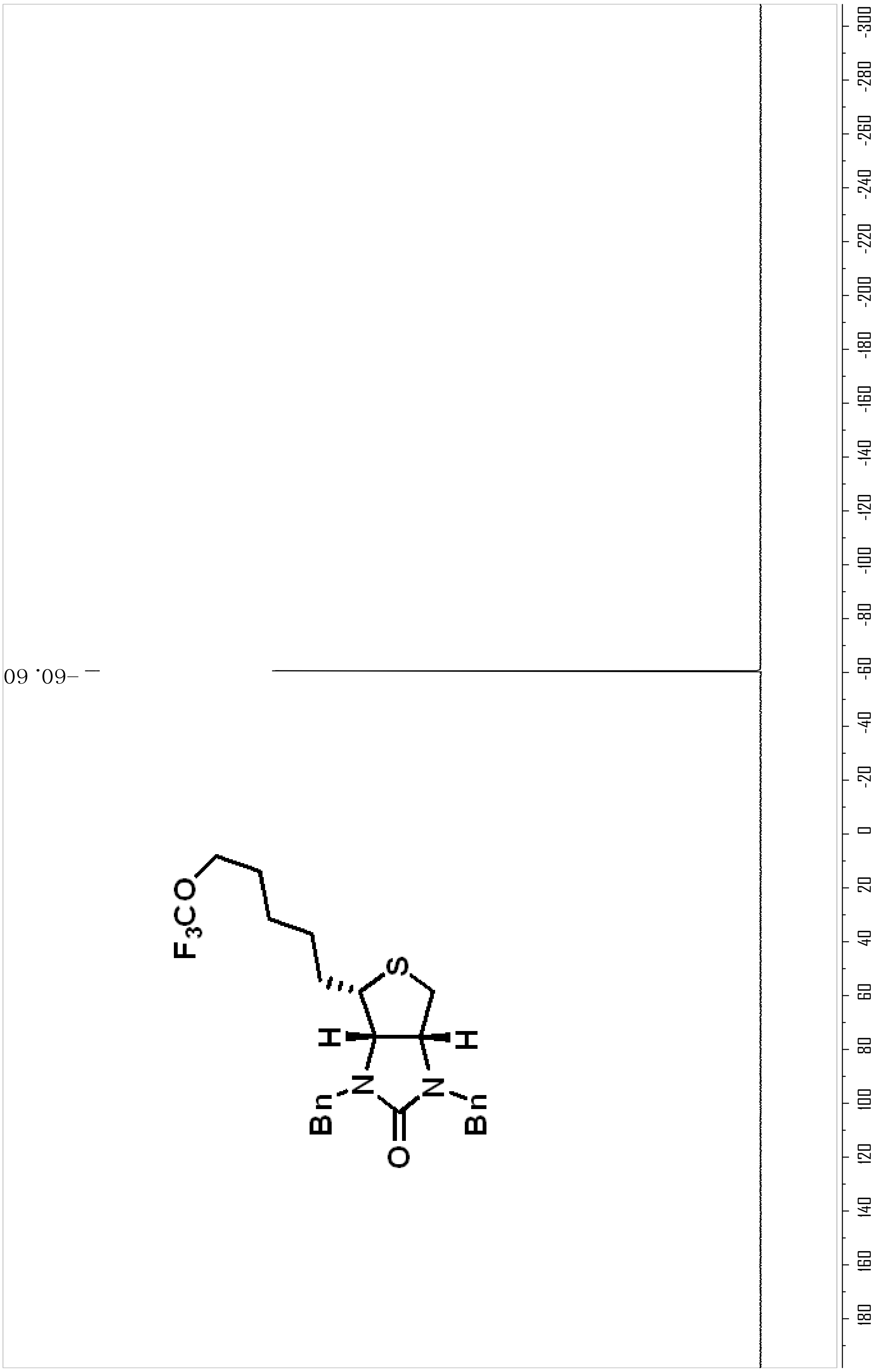

Supplementary Figure 224: ^{19}F NMR spectrum (376 MHz, DMSO, 23 °C) of **42**

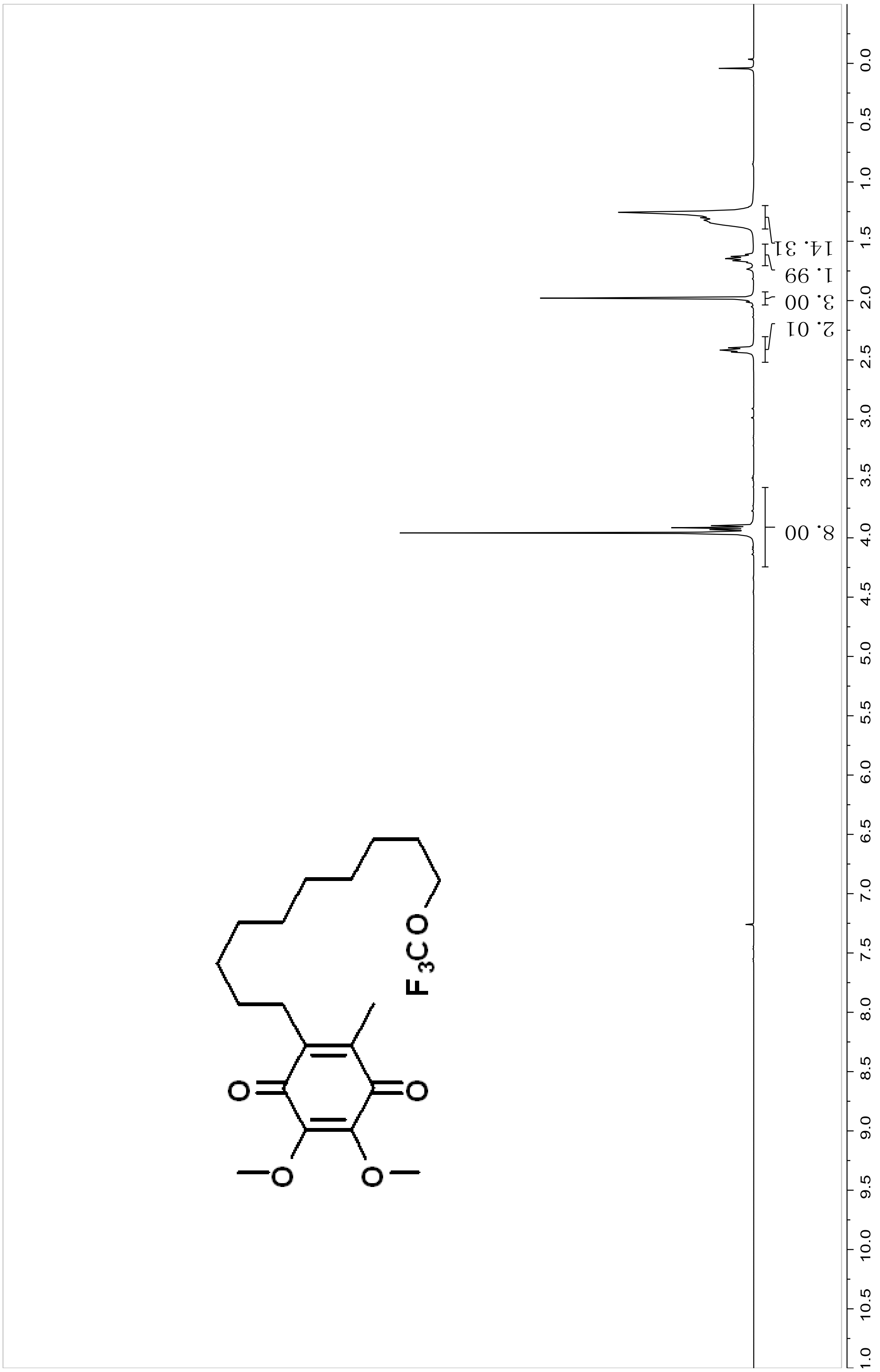

Supplementary Figure 225: ^{1}H NMR spectrum (400 MHz, $CDCl_3$, 23 °C) of **43**

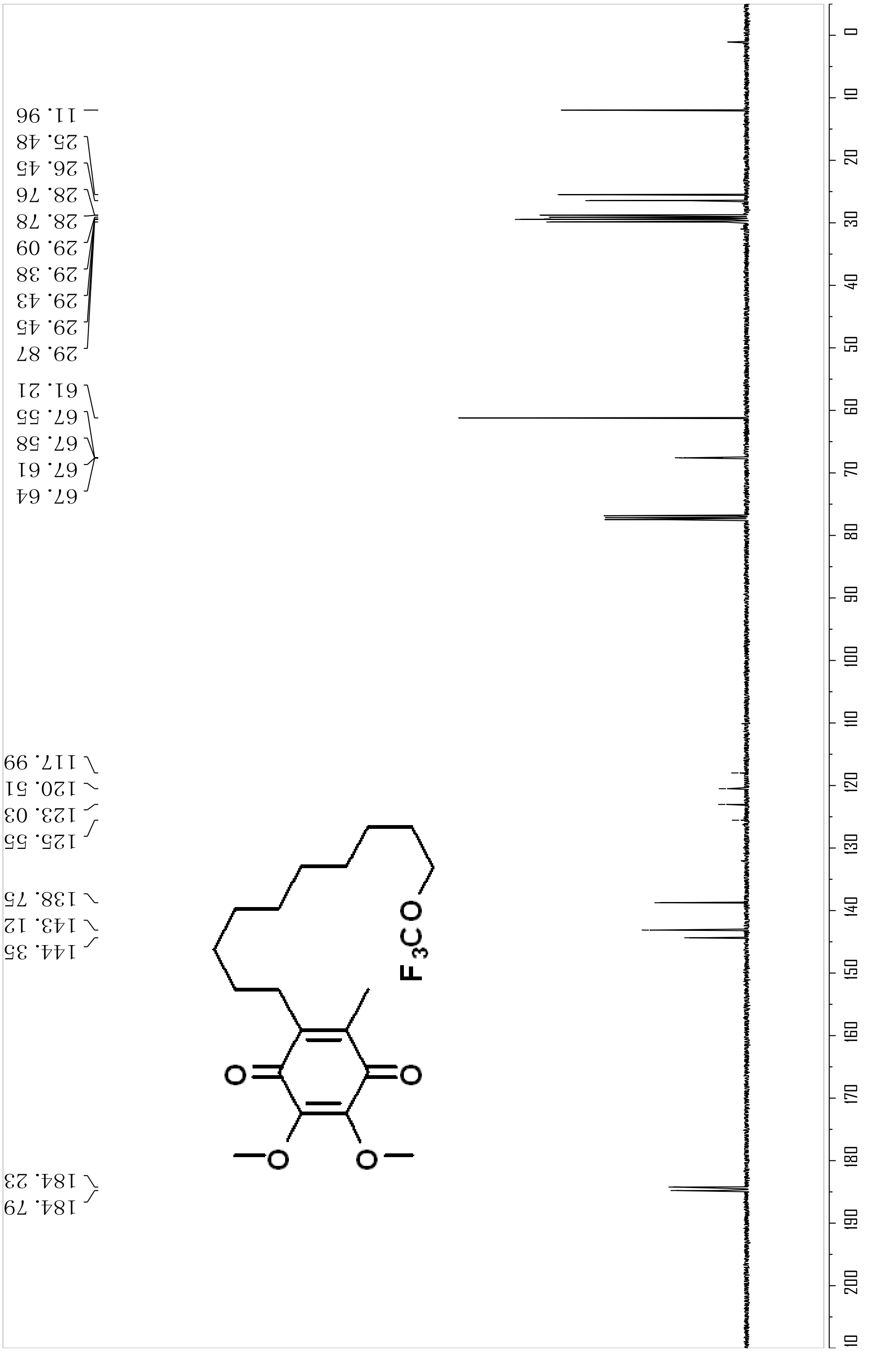

Supplementary Figure 226: ^{13}C NMR spectrum (101 MHz, $CDCl_3$, 23 °C) of **43**

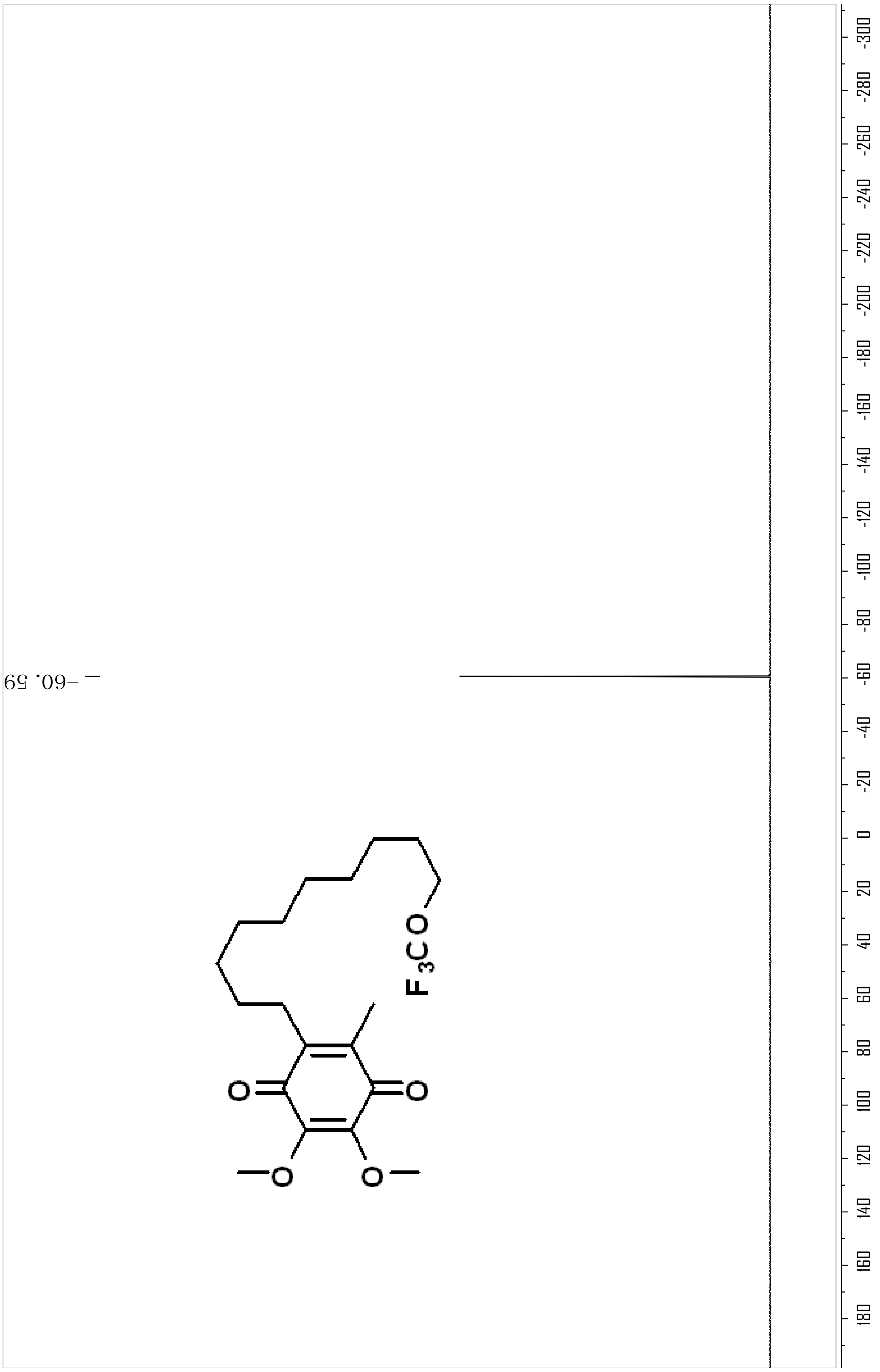

Supplementary Figure 227: ^{19}F NMR spectrum (376 MHz, $CDCl_3$, 23 °C) of **43**

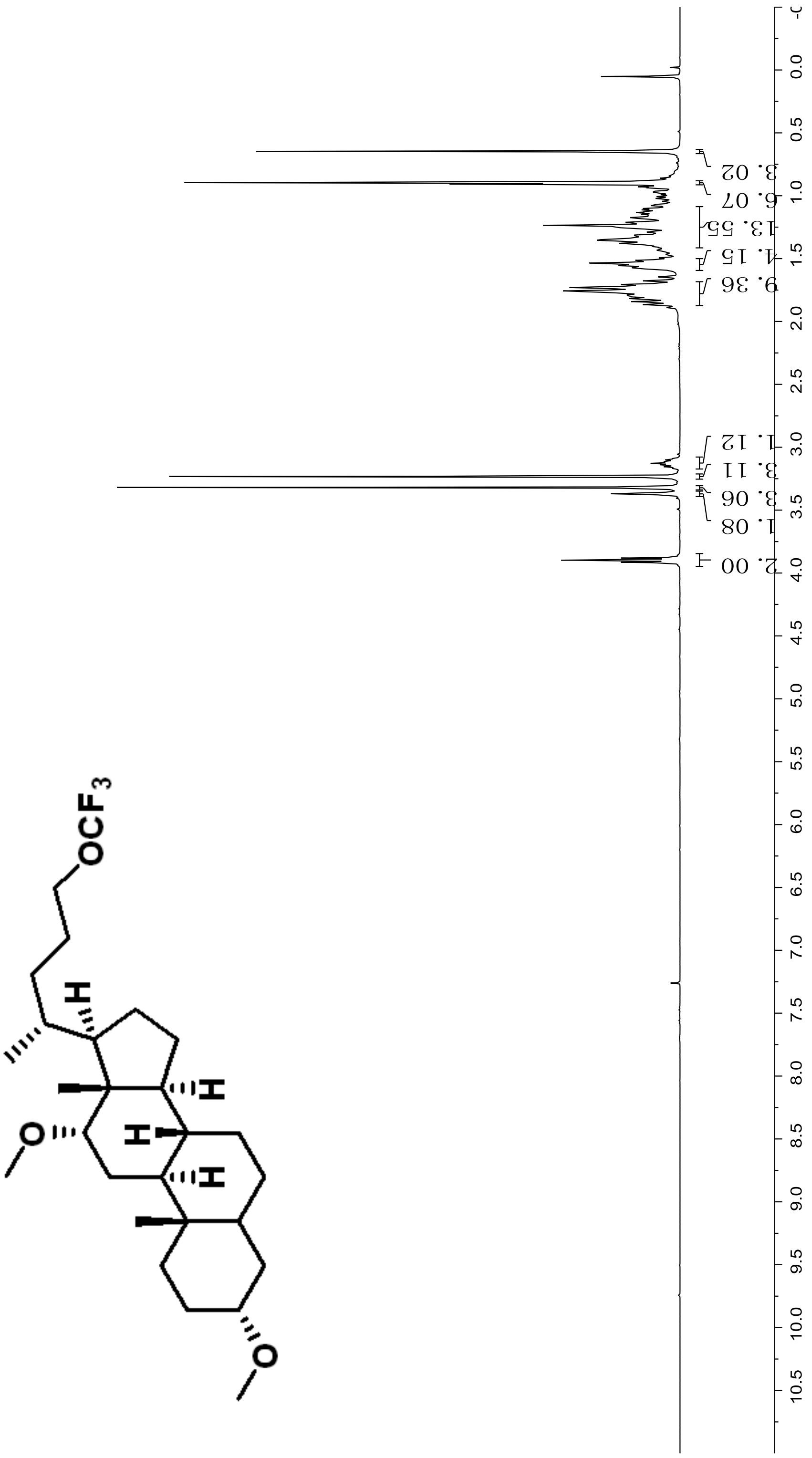

Supplementary Figure 228: ^{1}H NMR spectrum (400 MHz, $CDCl_3$, 23 °C) of **44**

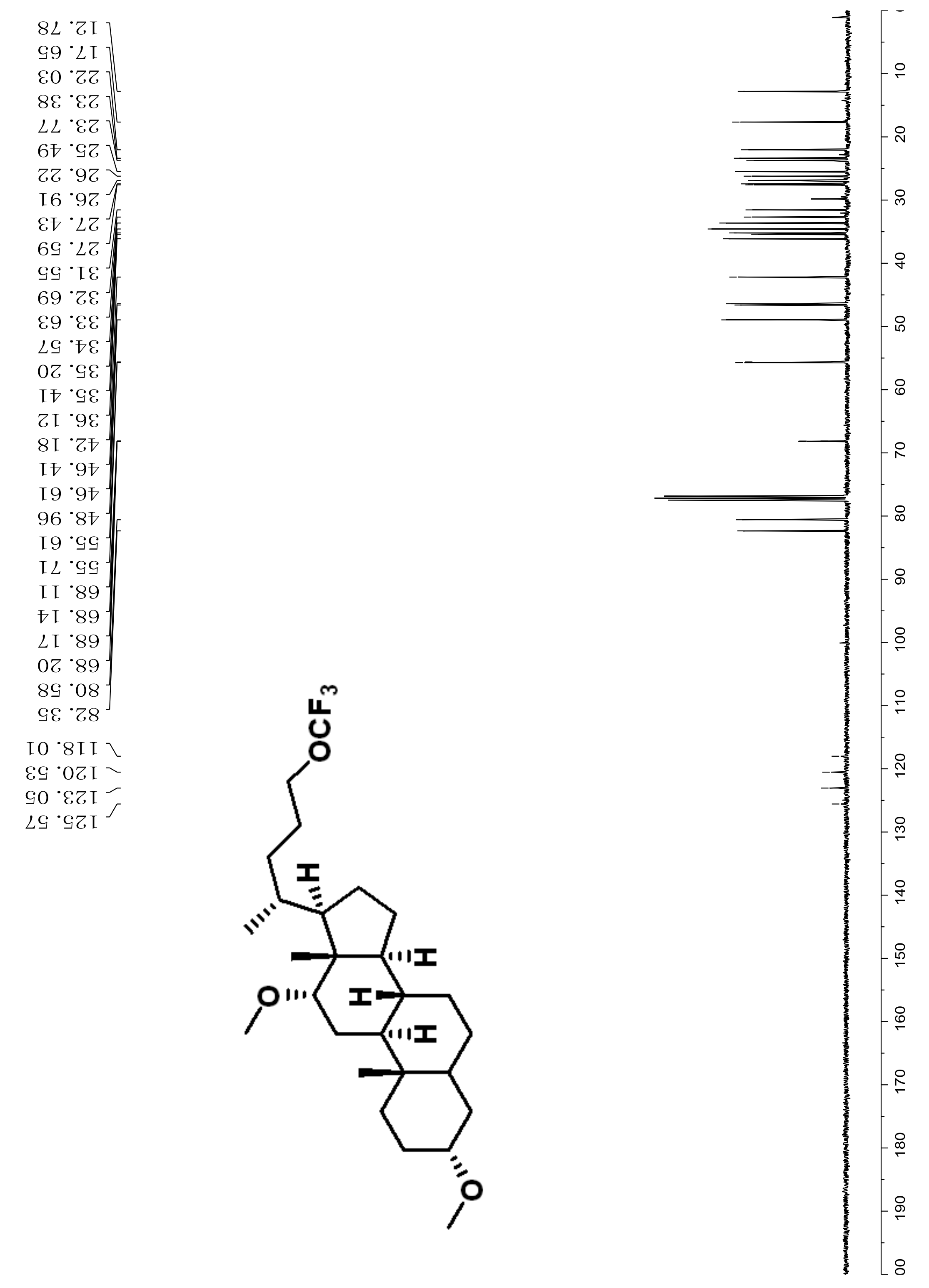

Supplementary Figure 229: ^{13}C NMR spectrum (101 MHz, $CDCl_3$, 23 °C) of **44**

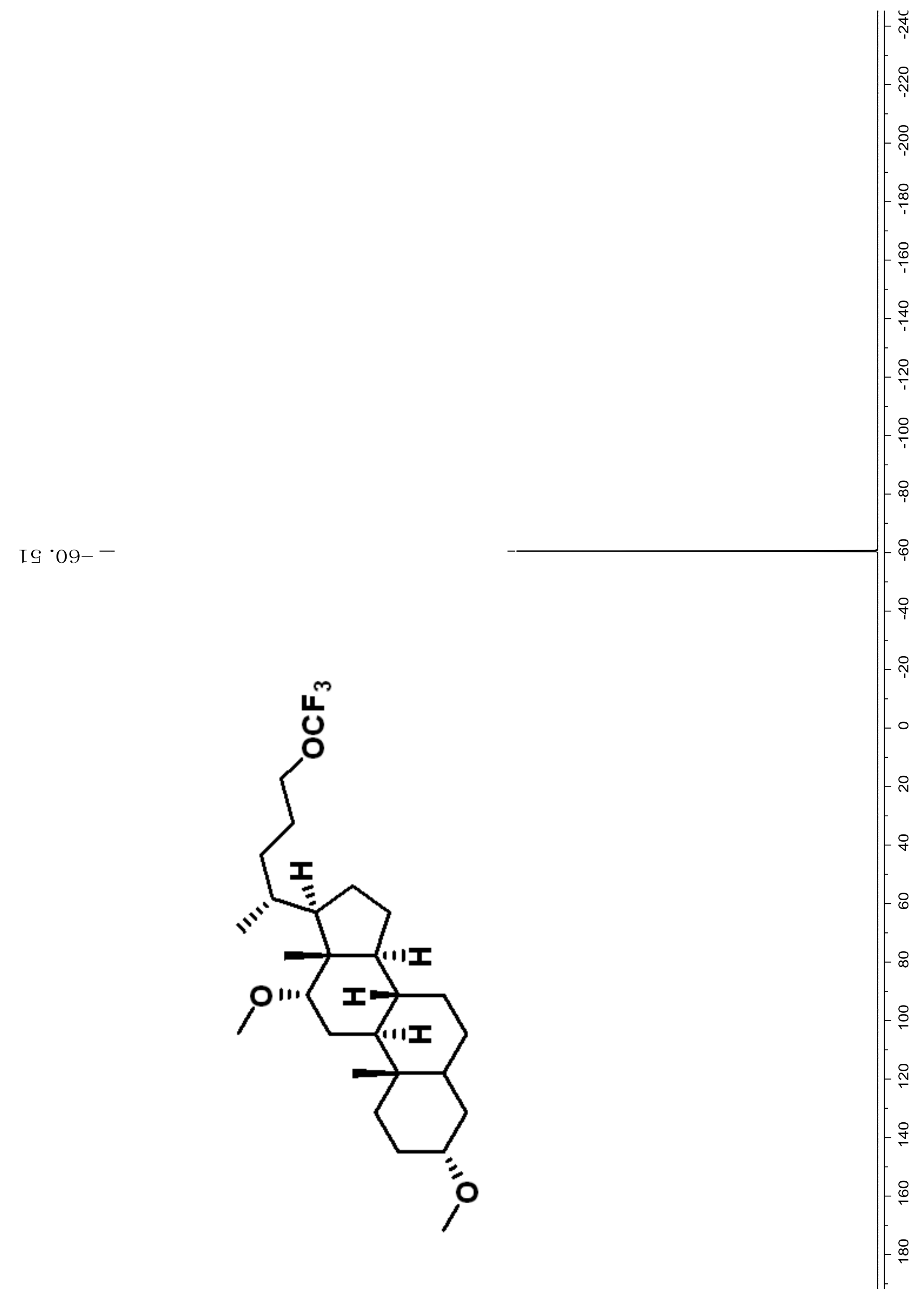

Supplementary Figure 230: ^{19}F NMR spectrum (376 MHz, DMSO, 23 °C) of **44**

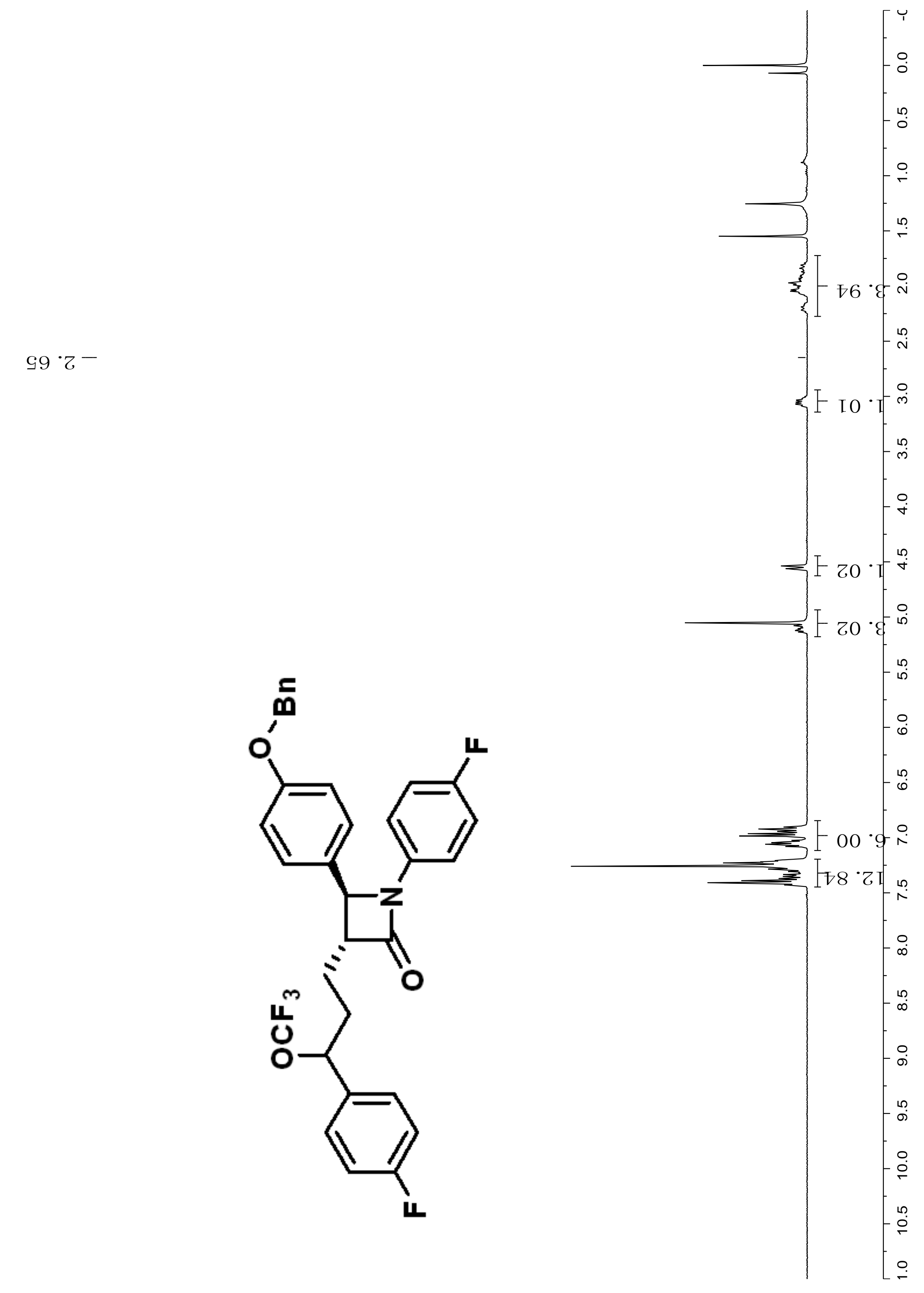

Supplementary Figure 231: ^{1}H NMR spectrum (400 MHz, $CDCl_3$, 23 °C) of **45**

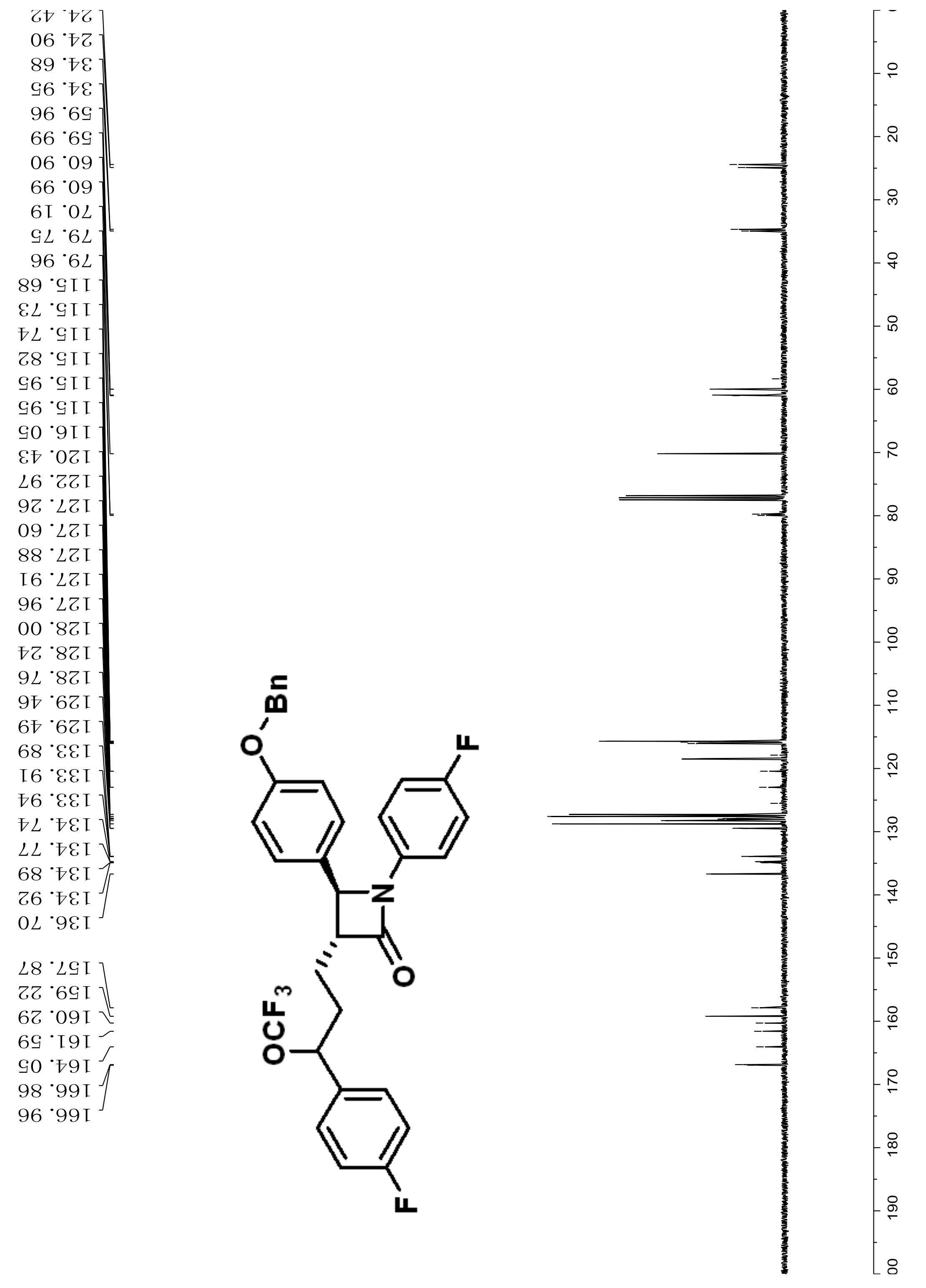

Supplementary Figure 232: ^{13}C NMR spectrum (101 MHz, $CDCl_3$, 23 °C) of **45**

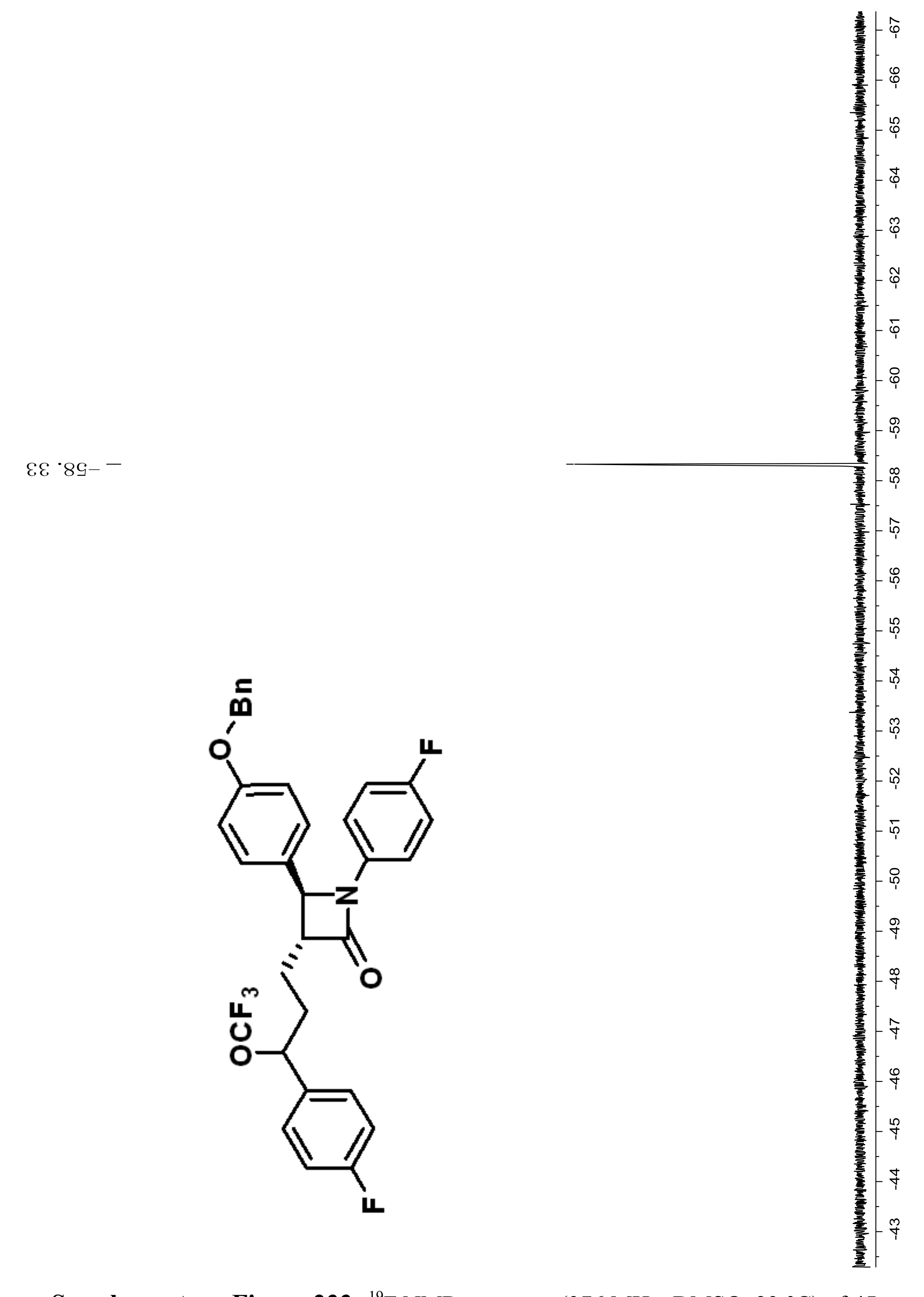

Supplementary Figure 233: ^{19}F NMR spectrum (376 MHz, DMSO, 23 °C) of **45**

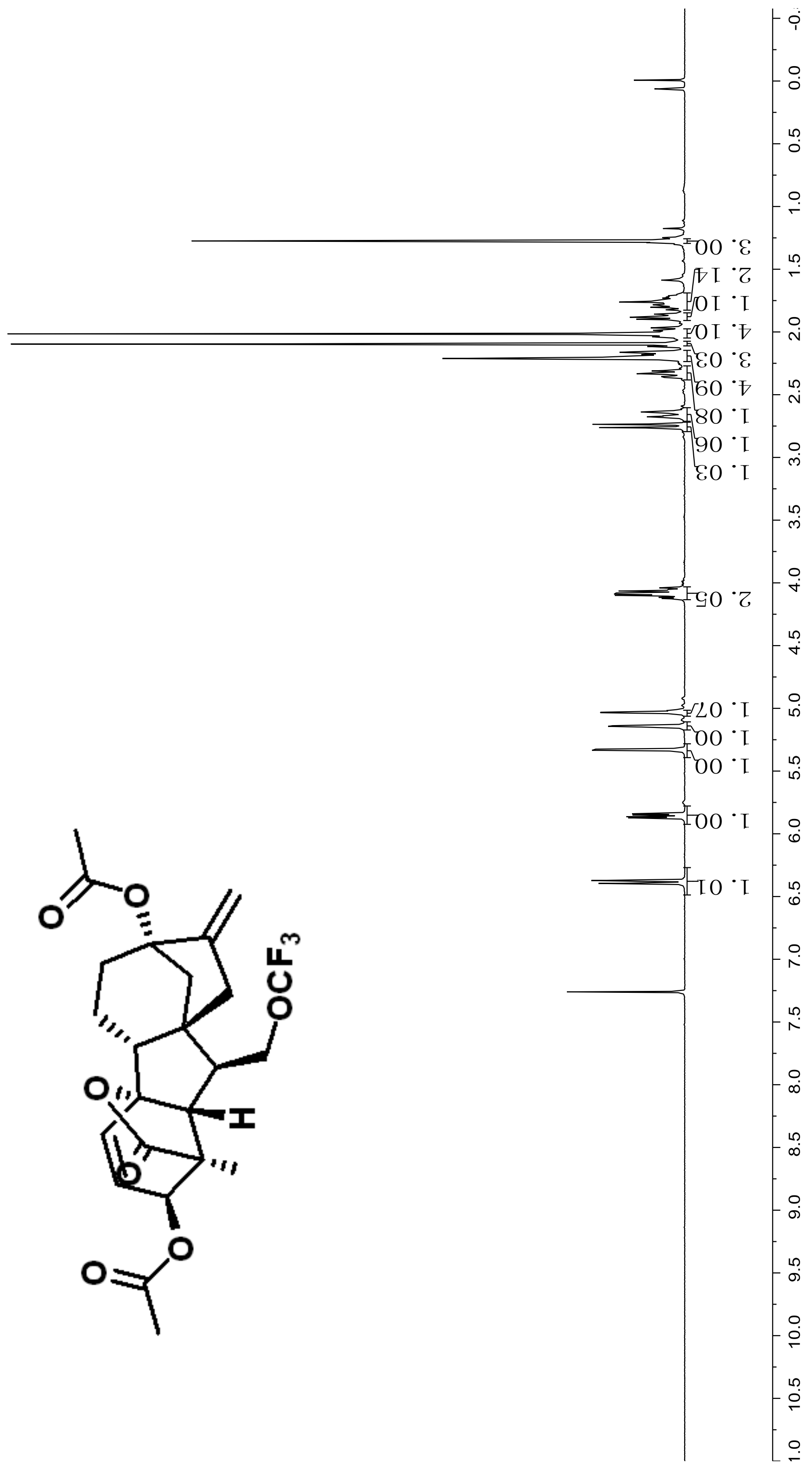

Supplementary Figure 234: ^{1}H NMR spectrum (400 MHz, $CDCl_3$, 23 °C) of **46**

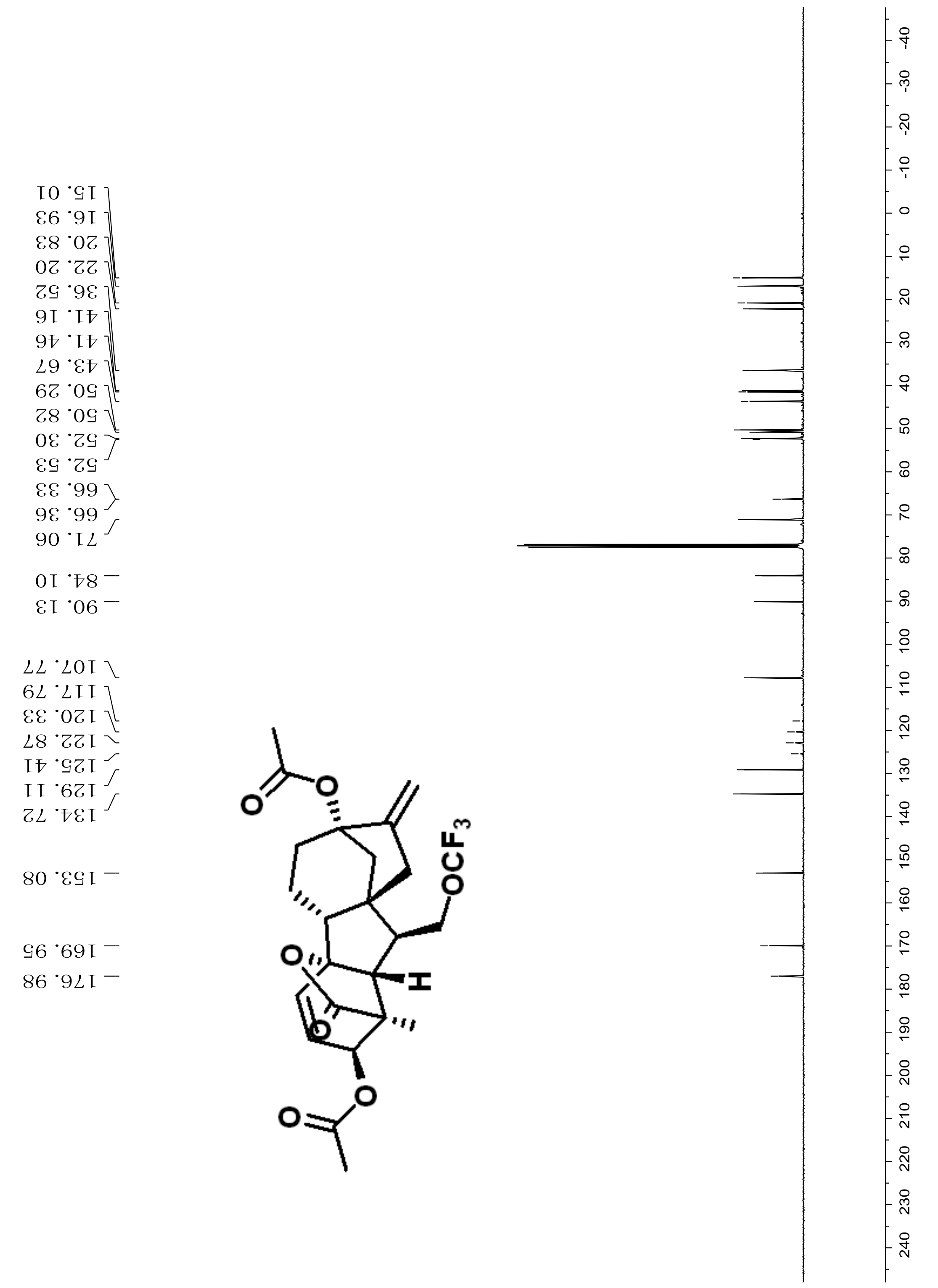

Supplementary Figure 235: ^{13}C NMR spectrum (101 MHz, $CDCl_3$, 23 °C) of **46**

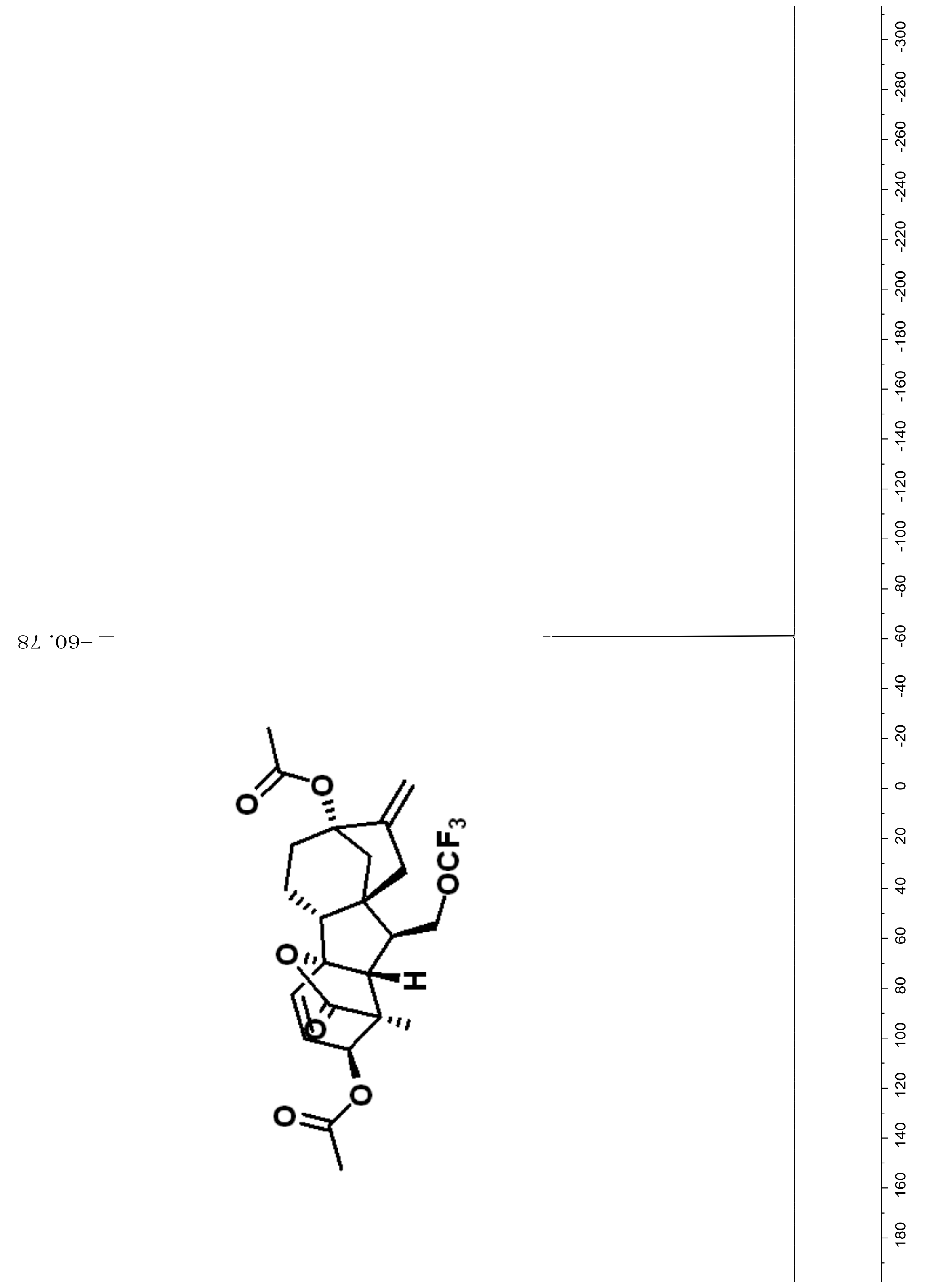

Supplementary Figure 236: ^{19}F NMR spectrum (376 MHz, $CDCl_3$, 23 °C) of **46**

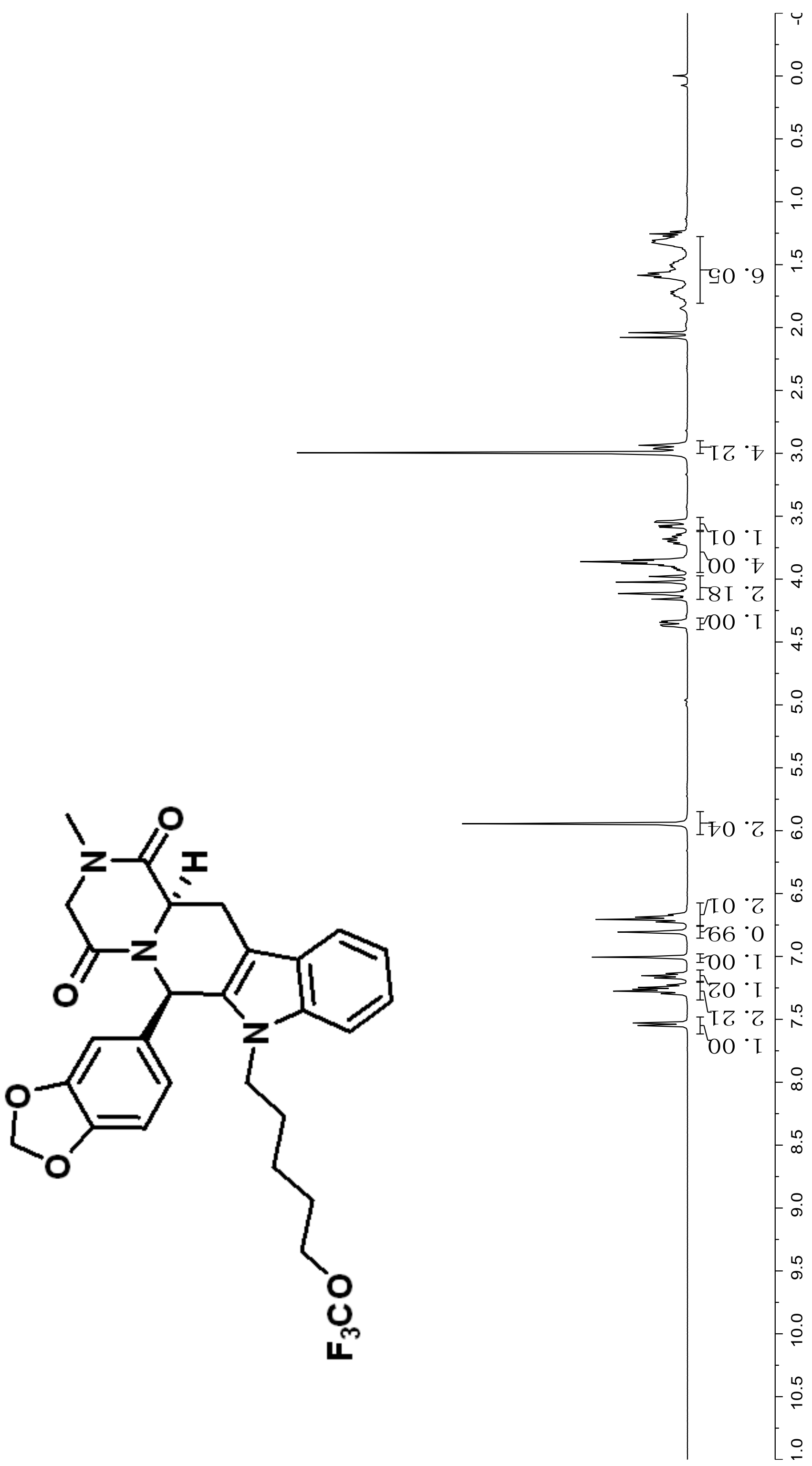

Supplementary Figure 237: ^{1}H NMR spectrum (400 MHz, $CDCl_3$, 23 °C) of **47**

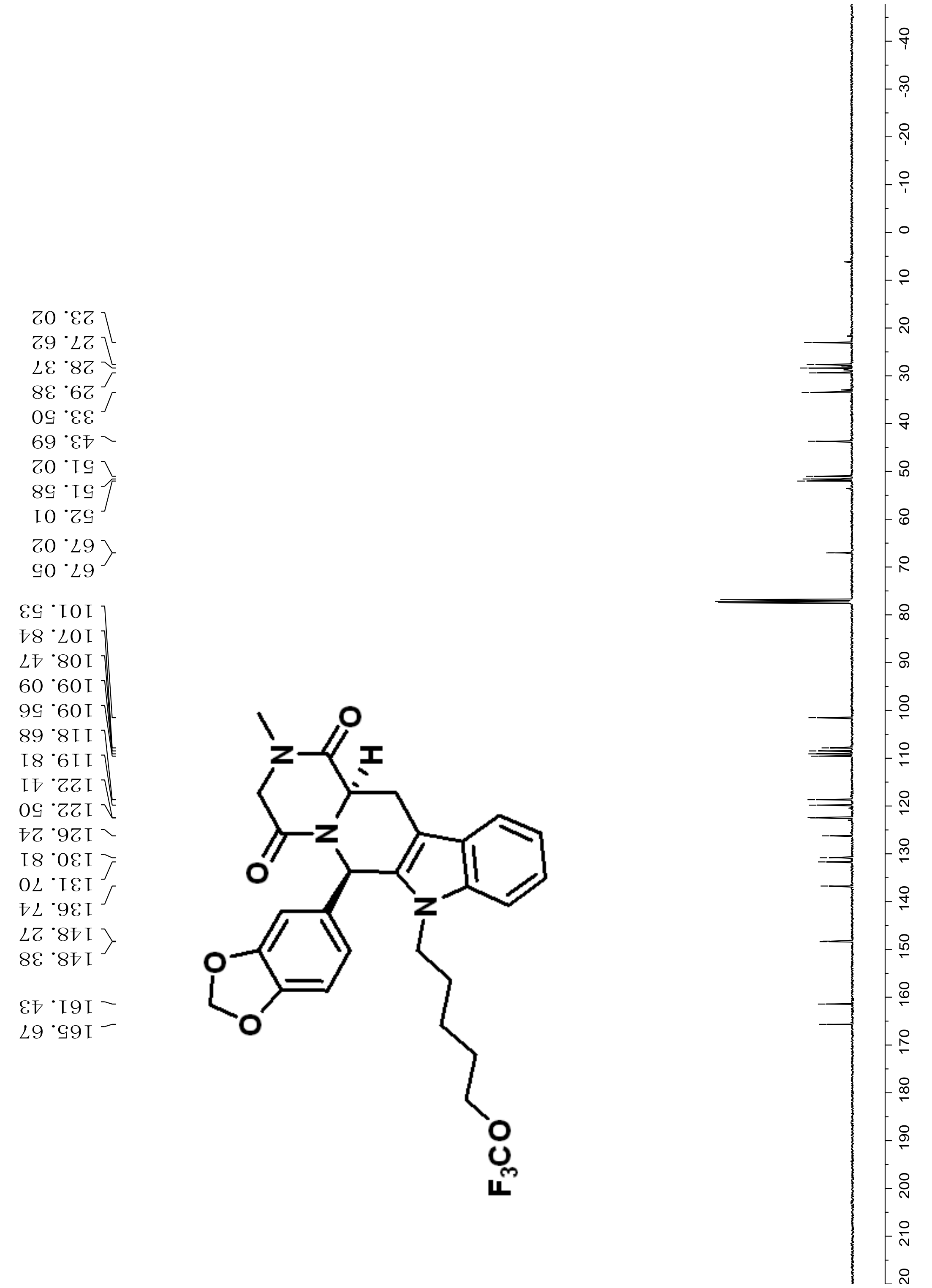

Supplementary Figure 238: ^{13}C NMR spectrum (101 MHz, $CDCl_3$, 23 °C) of **47**

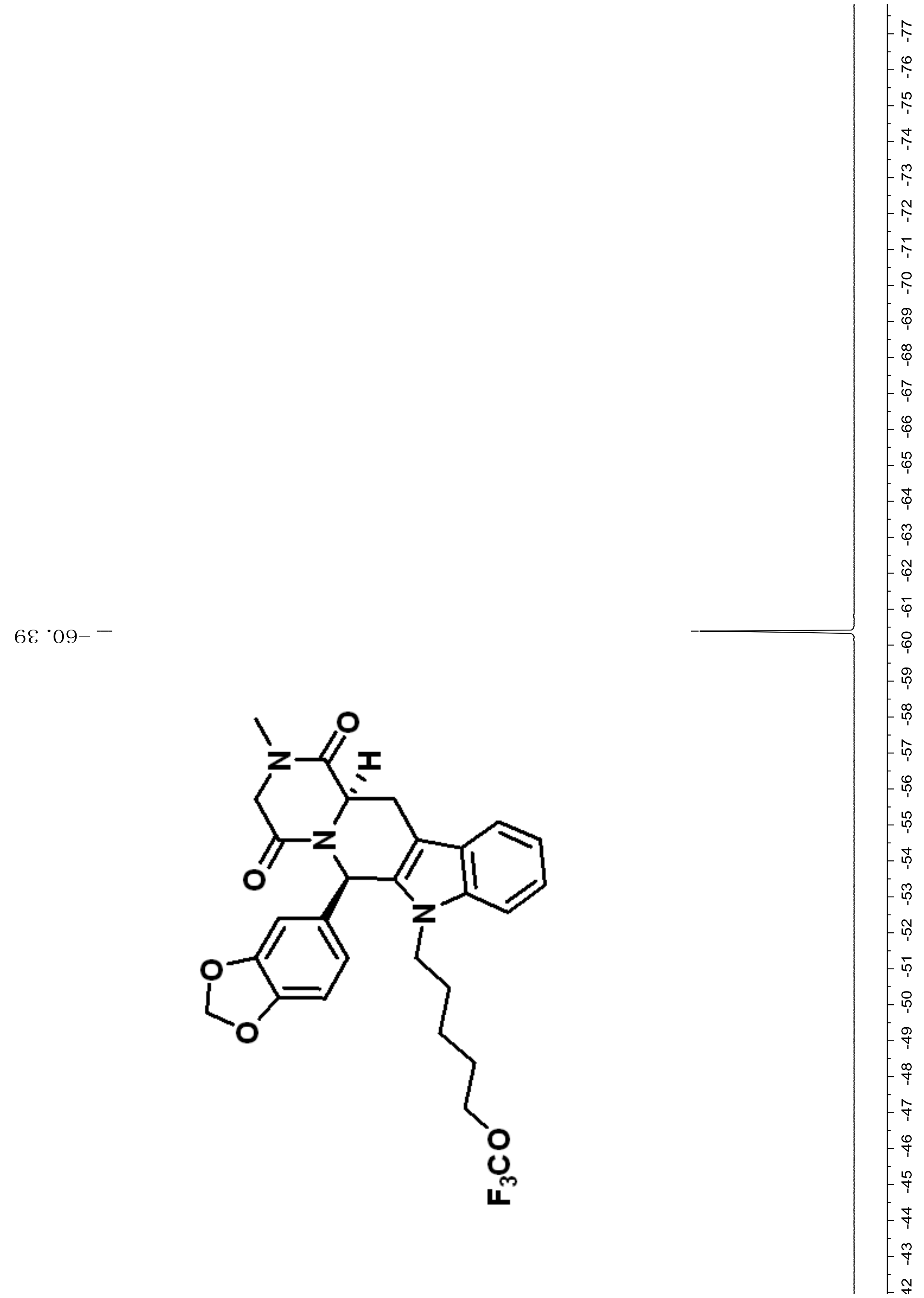

Supplementary Figure 239: ^{19}F NMR spectrum (376 MHz, $CDCl_3$, 23 °C) of **47**

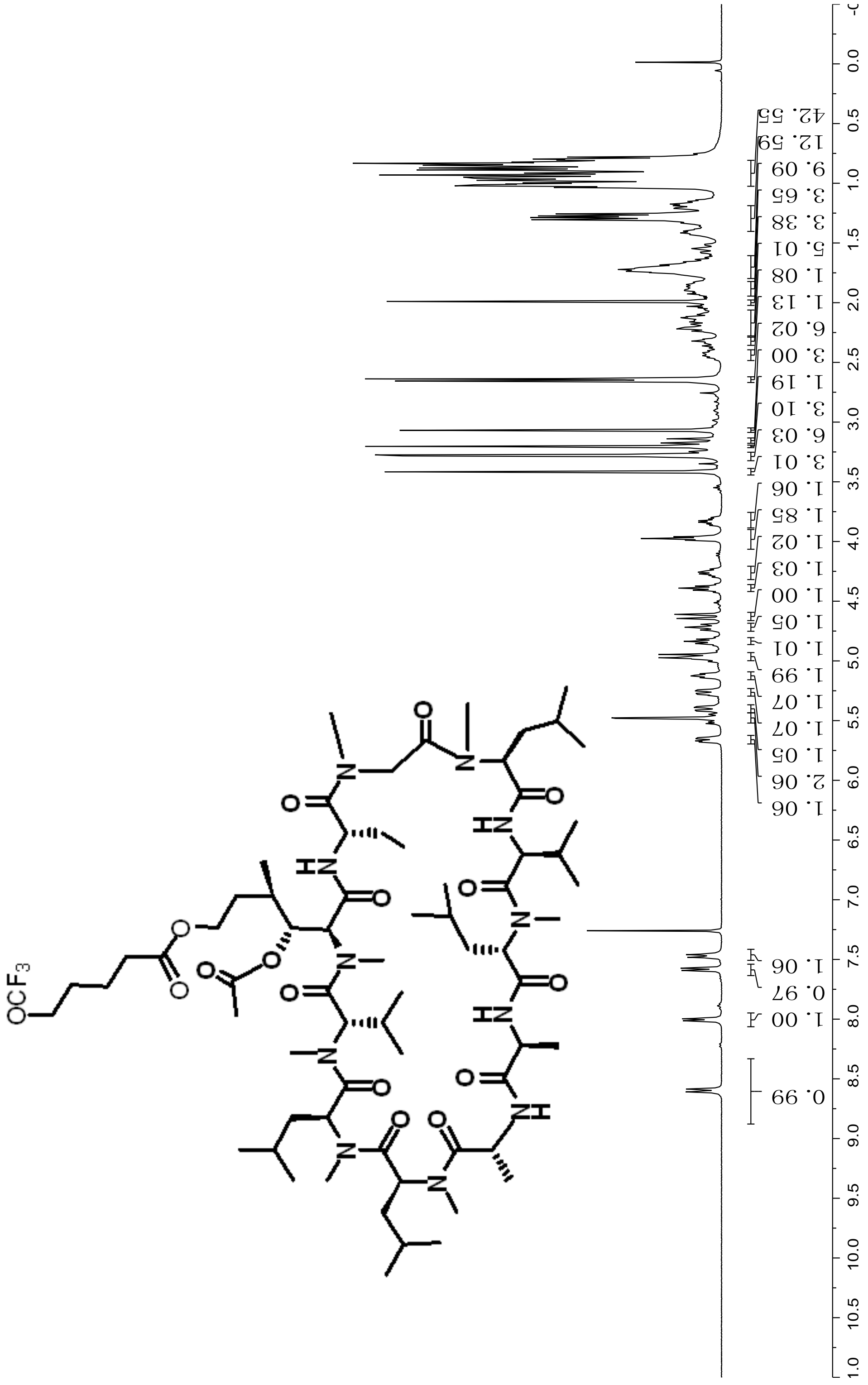

Supplementary Figure 240: ^{1}H NMR spectrum (400 MHz, $CDCl_3$, 23 °C) of **48**

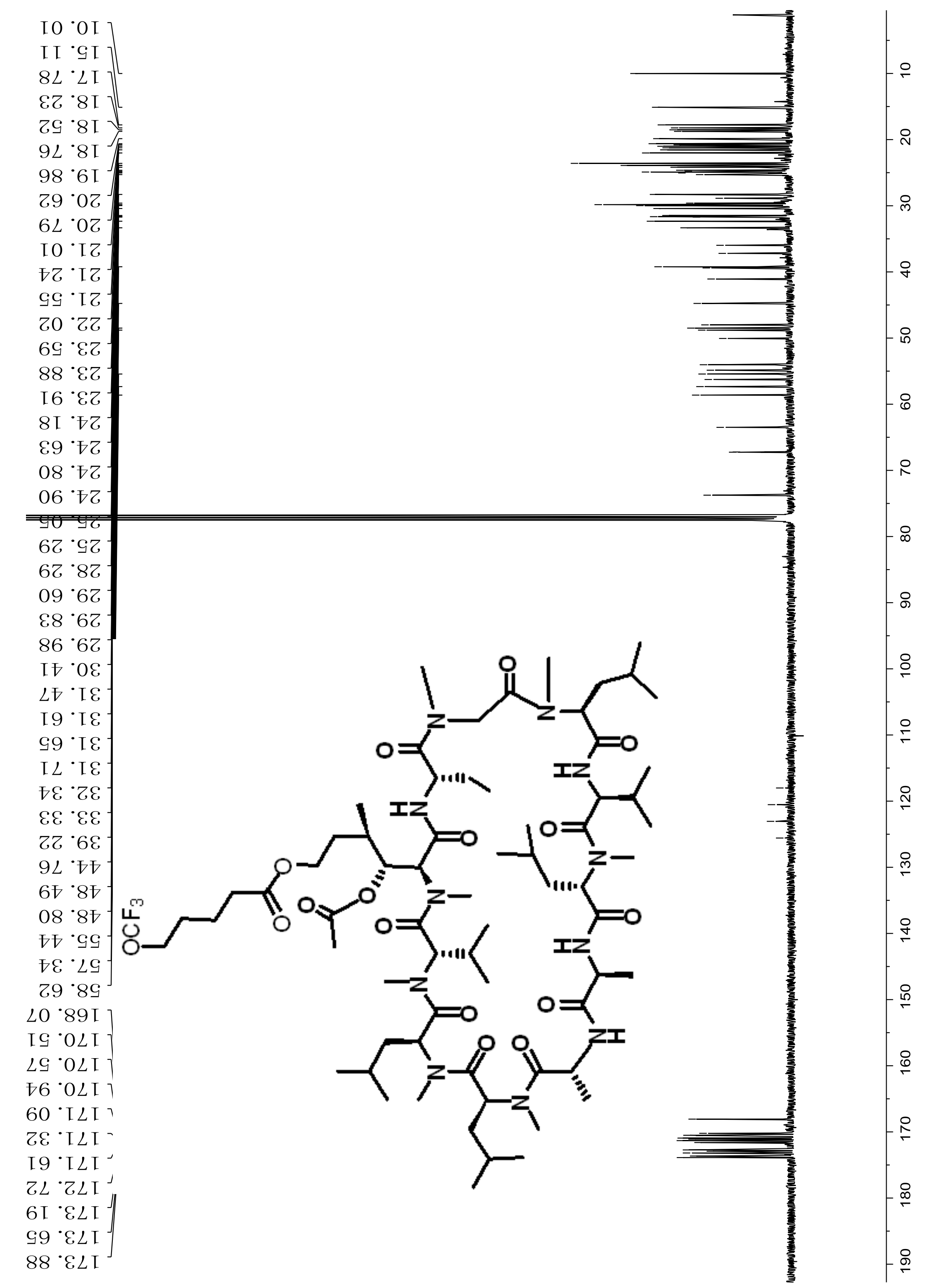

Supplementary Figure 241: ^{13}C NMR spectrum (101 MHz, $CDCl_3$, 23 °C) of **48**

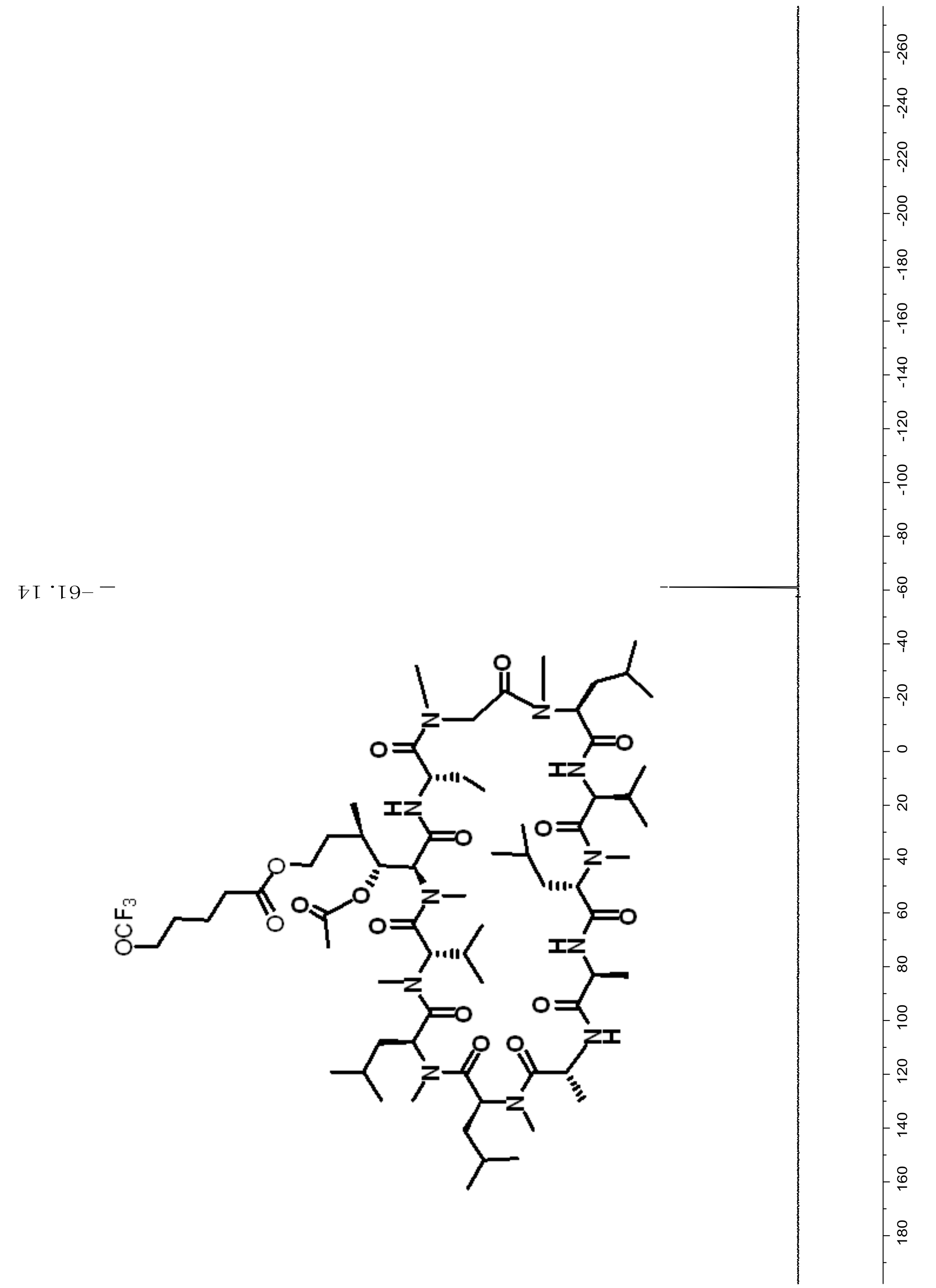

Supplementary Figure 242: ^{19}F NMR spectrum (376 MHz, DMSO, 23 °C) of **48**

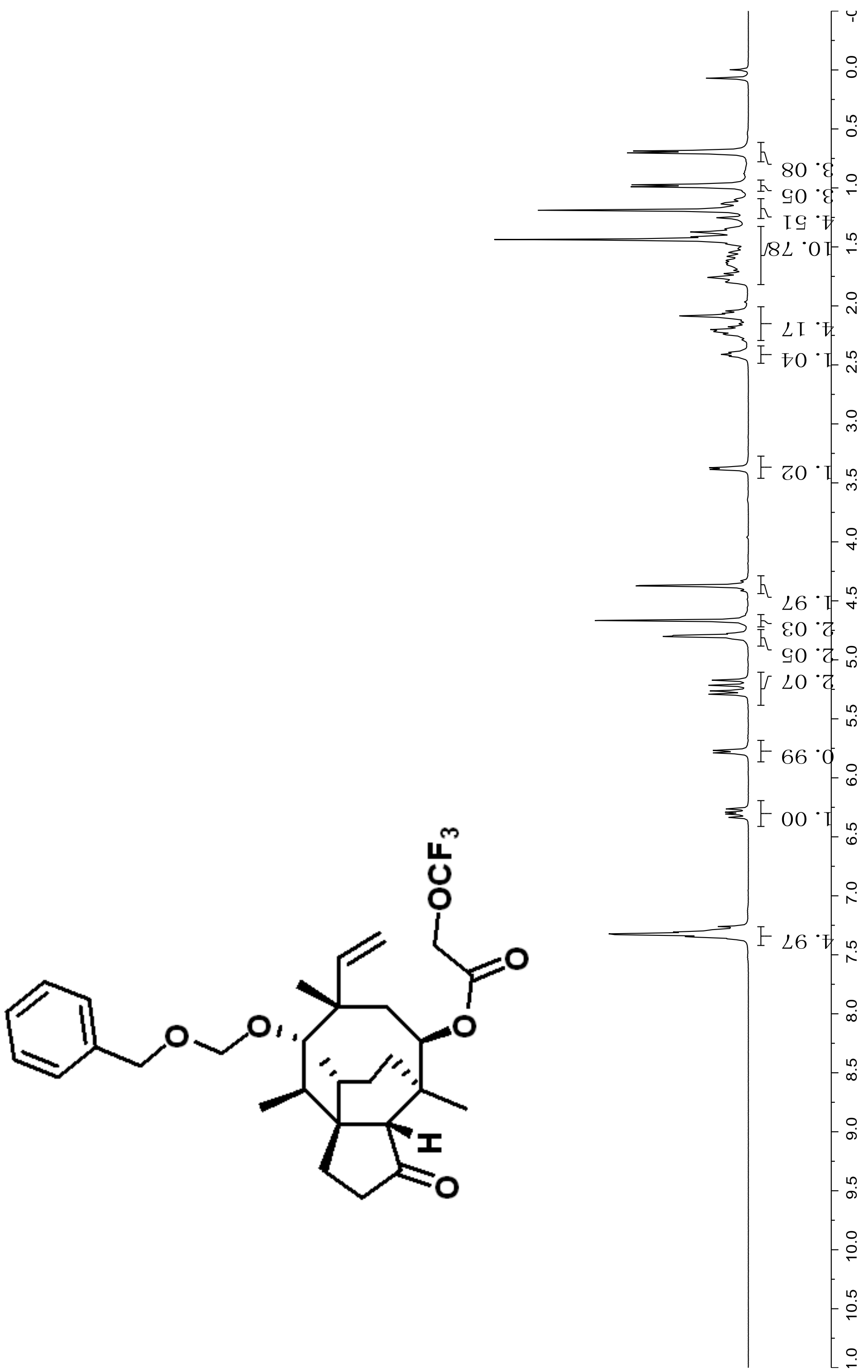

Supplementary Figure 243: ^{1}H NMR spectrum (400 MHz, $CDCl_3$, 23 °C) of **49**

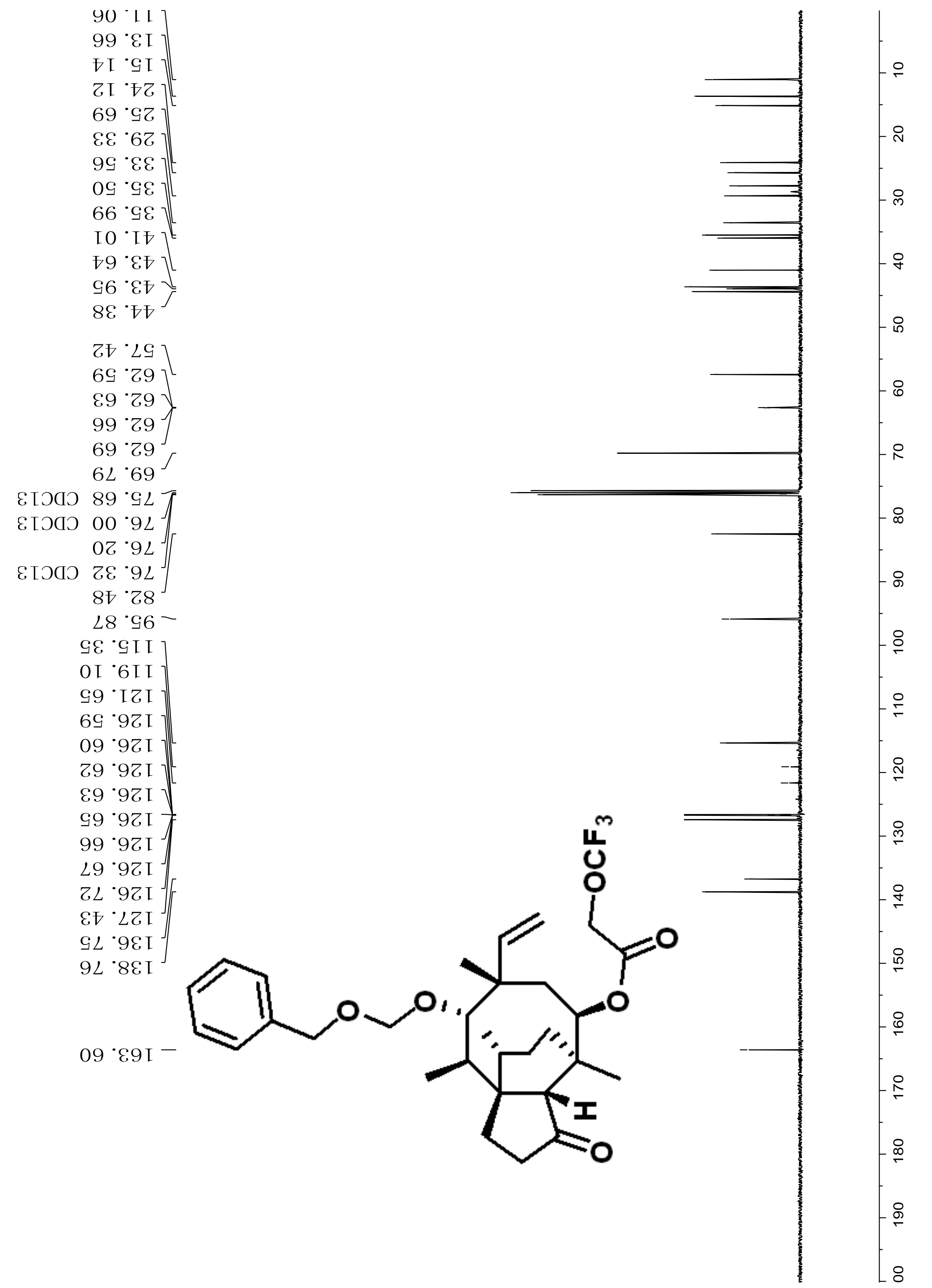

Supplementary Figure 244: ^{13}C NMR spectrum (101 MHz, $CDCl_3$, 23 °C) of **49**

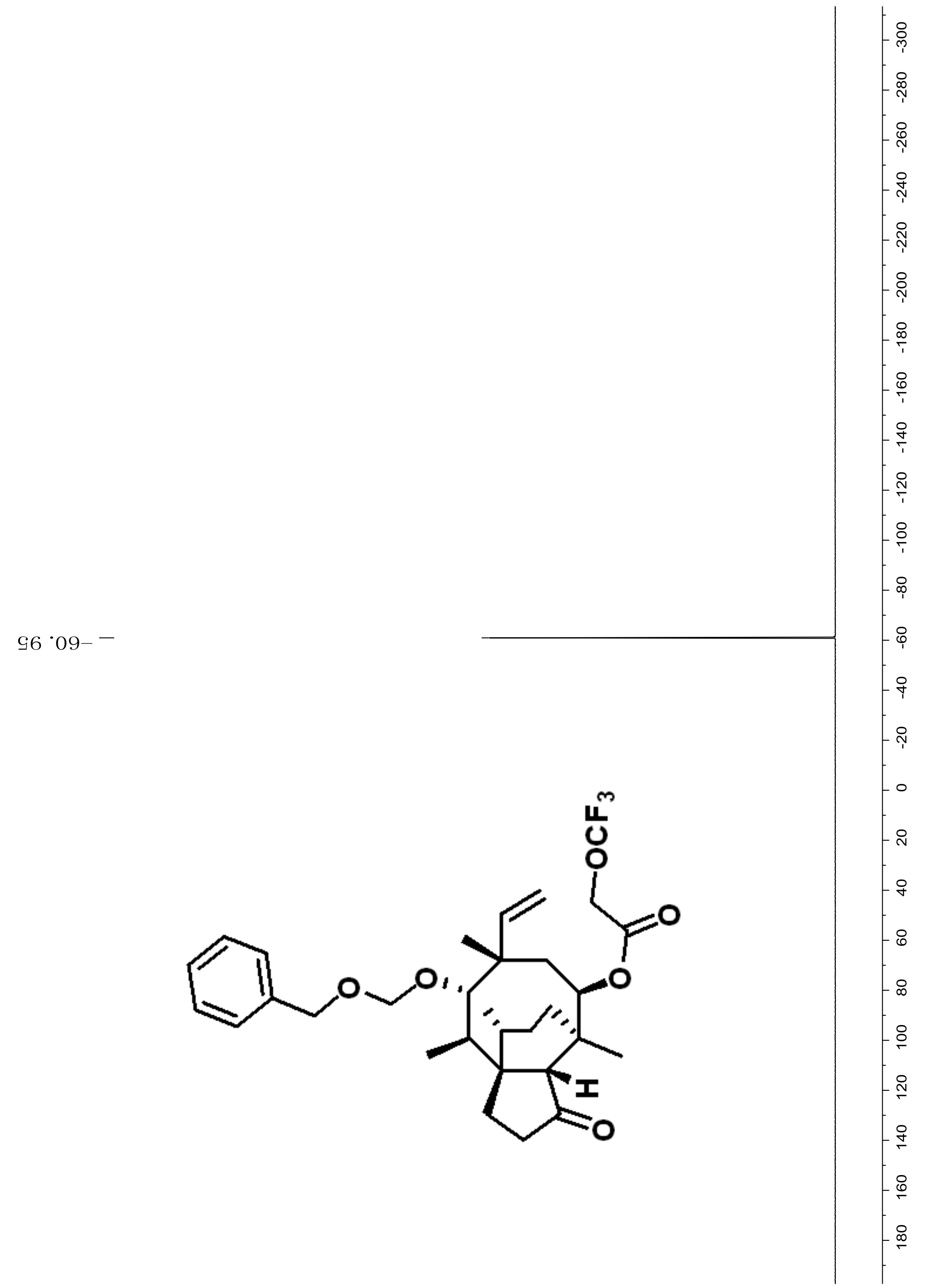

Supplementary Figure 245: ^{19}F NMR spectrum (376 MHz, $CDCl_3$, 23 °C) of **49**

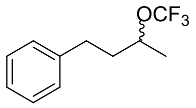

***33,** 8.7% *ee*

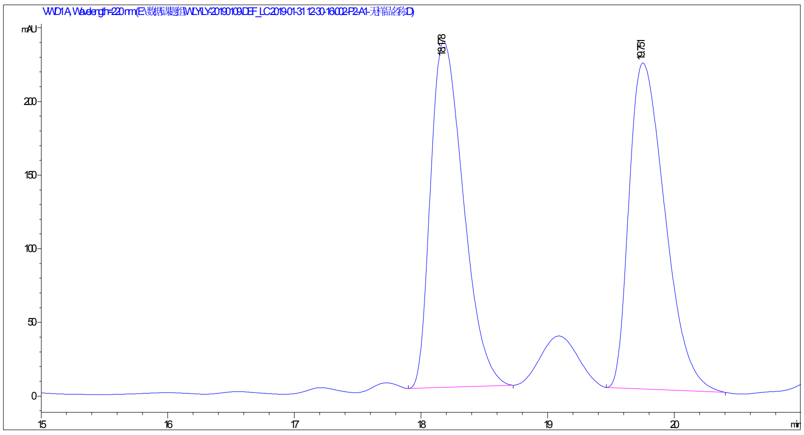

Supplementary Figure 246: Racemic Sample

Peak#	Ret.Time	Area	Height	Area %
1	18.178	4224.00928	234.81500	49.3505
2	19.751	4335.19385	220.98686	50.6495
Total		8559.20313	455.80186	100

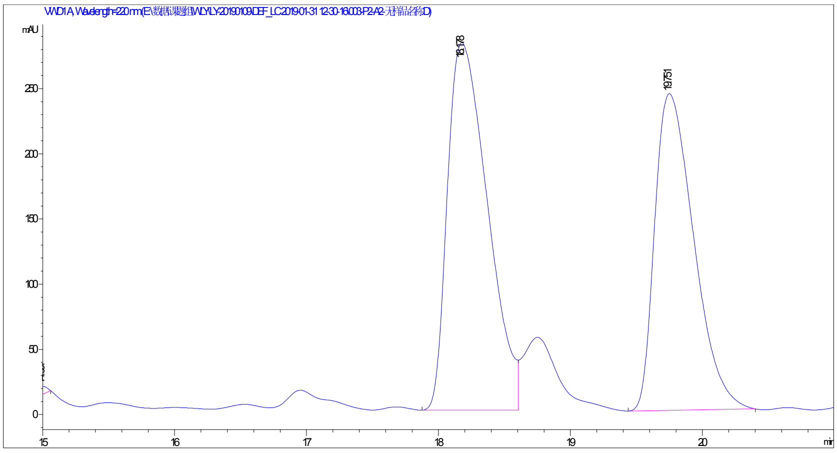

Supplementary Figure 247: Scalemic Sample

Peak#	Ret.Time	Area	Height	Area %
1	18.178	5943.69385	281.90341	54.3723
2	19.751	4987.78711	242.83966	45.6277
Total		1.09315e4	524.74307	100

Supplementary References

1 W. Zheng, C. A. Morales-Rivera, J. W. Lee, P. Liu, M. Y. Ngai, Catalytic C−H Trifluoromethoxylation of Arenes and Heteroarenes. *Angew. Chem. Int. Ed.* **57**, 9645-9649 (2018).

2 K. Oh, An efficient epimerization of biotin sulfone derivatives to 2-*epi*-biotin analogs. *Tetrahedron Letters* **48**, 3685–3688 (2007).

3 A. Fawcett, J. Pradeilles, Y. Wang, T. Mutsuga, E. L. Myers, V. K. Aggarwal, Photoinduced decarboxylative borylation of carboxylic acids. *Science* **357**, 283-286 (2017).

4 M. F. Richter, B. S. Drown, A. P. Riley, A. Garcia, T. Shirai, R. L. Svec, P. J. Hergenrother, Predictive compound accumulation rules yield a broad-spectrum antibiotic. *Nature* **545**, 299–304 (2017).

5 K. Levitsky, C. J. Ciolli, P. J. Belshaw, Selective Inhibition of Engineered Receptors via Proximity-Accelerated Alkylation. *Org. Lett.* **5**, 693-696 (2003).

6 X. Ma, R. Kucera, O. F. Goethe, S. K. Murphy, and S. B. Herzon, Directed C–H Bond Oxidation of (+)-Pleuromutilin. *J. Org. Chem.* **83**, 6843-6892 (2018).

7 O. Marrec, T. Billard, J. Vors, S. Pazenok, B. R. Langlois, A deeper insight into direct trifluoromethoxylation with trifluoromethyl triflate *J. Fluorine Chem.* **131**, 200-207 (2010).

8 X. Jiang, Z. Deng, P. Tang, Direct Dehydroxytrifluoromethoxylation of Alcohols. *Angew. Chem. Int. Ed.* **57**, 292-295 (2018).

9 H. Yang, F. Wang, X. Jiang, P. Tang, Silver-Promoted Oxidative Benzylic C-H Trifluoromethoxylation. *Angew. Chem. Int. Ed.* **57**, 13266-13270 (2018).

10 M. Zhou, C. Ni, Y. Zeng, J. Hu, Trifluoromethyl Benzoate: A Versatile Trifluoromethoxylation Reagent. *J. Am. Chem. Soc.* **140**, 6801−6805 (2018).

11 A. A. Kolomeitsev, M. Vorobyev, H. Gillandt, Versatile application of trifluoromethyl triflate. *Tetrahedron Letters* **49**, 449–454 (2008).
